# Novel and conserved miRNAs in the halophyte *Suaeda maritima* identified by deep sequencing and computational predictions using the ESTs of two mangrove plants

**DOI:** 10.1186/s12870-015-0682-3

**Published:** 2015-12-29

**Authors:** Sachin Ashruba Gharat, Birendra Prasad Shaw

**Affiliations:** Environmental Biotechnology Laboratory, Institute of Life Sciences, Bhubaneswar, 751023 Odisha India

**Keywords:** *Sesuvium portulacastrum*, miRNA, Salinity, NaCl, Abiotic stress, Halophyte, *Oryza sativa*

## Abstract

**Background:**

Although miRNAs are reportedly involved in the salt stress tolerance of plants, miRNA profiling in plants has largely remained restricted to glycophytes, including certain crop species that do not exhibit any tolerance to salinity. Hence, this manuscript describes the results from the miRNA profiling of the halophyte *Suaeda maritima*, which is used worldwide to study salt tolerance in plants.

**Results:**

A total of 134 conserved miRNAs were identified from unique sRNA reads, with 126 identified using miRBase 21.0 and an additional eight identified using the Plant Non-coding RNA Database. The presence of the precursors of seven conserved miRNAs was validated in *S. maritima*. In addition, 13 novel miRNAs were predicted using the ESTs of two mangrove plants, *Rhizophora mangle* and *Heritiera littoralis*, and the precursors of seven miRNAs were found in *S. maritima*. Most of the miRNAs considered for characterization were responsive to NaCl application, indicating their importance in the regulation of metabolic activities in plants exposed to salinity. An expression study of the novel miRNAs in plants of diverse ecological and taxonomic groups revealed that two of the miRNAs, sma-miR6 and sma-miR7, were also expressed in *Oryza sativa*, whereas another two, sma-miR2 and sma-miR5, were only expressed in plants growing under the influence of seawater, similar to *S. maritima*.

**Conclusion:**

The distribution of conserved miRNAs among only 25 families indicated the possibility of identifying a greater number of miRNAs with increase in knowledge of the genomes of more halophytes. The expression of two novel miRNAs, sma-miR2 and sma-miR5, only in plants growing under the influence of seawater suggested their metabolic regulatory roles specific to saline environments, and such behavior might be mediated by alterations in the expression of certain genes, modifications of proteins leading to changes in their activity and production of secondary metabolites as revealed by the miRNA target predictions. Moreover, the auxin responsive factor targeted by sma-miR7 could also be involved in salt tolerance because the target is conserved between species. This study also indicated that the transcriptome of one species can be successfully used to computationally predict the miRNAs in other species, especially those that have similar metabolism, even if they are taxonomically separated.

**Electronic supplementary material:**

The online version of this article (doi:10.1186/s12870-015-0682-3) contains supplementary material, which is available to authorized users.

## Background

The spatial and temporal regulation of gene expression in response to environmental cues are important factors for determining plant survival and adaptability leading to development of an ecotype [1]. Transcriptome studies have shown that plants challenged with an abiotic stress present altered expression levels of a number of genes involved in a broad spectrum of biochemical, cellular and physiological processes, including genes providing resistance to the abiotic stress [2–4]. Alterations to gene expression may occur because of regulatory mechanisms working at several levels. The two most well-known and studied categories of regulation are those involving transcription factors and small RNAs (sRNAs). While transcription factors work at the transcription level, sRNAs regulate gene expression at both the transcription and post-transcription levels. The importance of transcription factors in the regulation of gene expression and their associated mechanisms has been known since the 1980s [5–7] and can be illustrated by the presence of a massive number of transcription factor families and the extensive combinatorial control of gene expression by multiple transcription factors. However, the regulatory role of sRNAs in gene expression was only discovered two decades later after observing the reduced accumulation of gene products (mRNA) upon the introduction of dsRNA homologous to the gene into the tissue [8, 9]. As per our current understanding, sRNAs are typified by a large and growing class of ~22-nucleotide (nt)-long non-coding RNAs that function in association with Argonaute (Ago)-family proteins [10].

In plants, sRNAs exhibit an unexpected complexity and are classified based on their biogenesis and the structure of the genomic loci from which they are transcribed [11]. At present, sRNAs are distinguished as microRNAs (miRNAs) and three classes of endogenous small interfering RNAs (siRNAs), specifically trans-acting siRNA (ta-siRNA), heterochromatic siRNA (hc-siRNA) and natural antisense siRNA (nat-siRNA) [11, 12]. Among these, miRNA-guided post-transcriptional gene regulation constitutes one of the most conserved and well-characterized gene regulatory mechanisms, and it is important for development, stress responses and a myriad of other biological processes in eukaryotes [11, 13, 14]. The involvement of miRNAs increases the complexity of gene regulation processes because miRNAs generally have multiple targets, including transcription factors, and transcription factors also regulate the expression of pri-miRNA [15]. This complexity is further increased because miRNAs have been discovered to regulate gene expression at the transcriptional level [16]. In one of the first records of miRNA involvement in transcriptional gene silencing, Bao et al. [17] showed that mutations in the *Arabidopsis PHABULOSA (PHB)* and *PHAVOLUTA (PHV)* transcription factor genes, which affect the ath-miR165/166 complementary site in the processed mRNAs, presented decreased methylation of the gene downstream of the complementary site compared with that of the wild type. Similarly, Kim et al. [18] reported that the expression of *POLR3D* (DNA-directed RNA polymerase III subunit RPC4) was negatively regulated by miR320 mediated through histone methylation. However, Place et al. [19] observed the presence of a miR373 target site in the promoters of E-cadherin and cold-shock domain-containing protein C2 (CSDC2), and this miRNA induced the expression of both genes. In fact, miR373 represents the first evidence that miRNAs targeting promoters can enhance the synthesis of RNA or RNA activation (RNAa) in a manner similar to that shown by small activating RNAs (saRNA). Recently, Huang et al. [20] showed that three miRNAs (miR744, miR1186 and miR466d-3p) induced the expression of Ccnb1 (Cyclin B1) in mouse cell lines. In addition, miRNAs have also been reported to induce upregulation of translation of target mRNAs [21–23], making these molecules highly versatile components of gene regulation and function.

The regulatory roles of miRNAs in biotic and abiotic responses in plants have also been suggested [11, 24–27]. Sunkar and Zhu [28] were the first to demonstrate the upregulation of miR393, miR402, miR397b and miR319c by at least one of the following stresses: drought, cold and salt. Similarly, Katiyar-Agarwal et al. [29] described a novel class of bacteria-induced sRNAs in *Arabidopsis thaliana*. However, although further work on miRNA responses to biotic stress in plants has been conducted, particularly for pathogen-host specificity, assessments of such responses with regard to abiotic stress have mostly considered test species that are either intolerant or naturally not tolerant to a particular type of stress. To the best of our knowledge, the current understanding of miRNA expression in halophytes, the naturally salt tolerant plants, is limited to three species: *Avicennia marina* [30], *Salicornia europaea* [27] and *Salicornia brachiata* [31]. Deep sRNA sequencing has only been performed for the former two species, whereas high-throughput sRNA sequencing data are available for innumerable numbers of glycophytes.

The responsiveness of most miRNAs to abiotic stresses in glycophytes has been found to be species specific rather than stress specific although they might be involved in stress tolerance [13, 24, 26, 32–34]. It may be that the miRNA-mediated regulatory requirements for stress tolerance could vary with different species. Moreover, the majority of stress-related miRNA in miRNA databases were identified in model plant species, such as *Arabidopsis lyrata*, *A. thaliana*, *Brachypodium distachyon*, *Glycine max*, *Medicago truncatula*, *Nicotiana tabacum*, *Oryza sativa*, *Physcomitrella patens*, *Populus trichocarpa*, *Solanum tuberosum*, *Sorghum bicolor*, *Vitis vinifera* and *Zea mays*, and they constitute 5380 miRNAs out of the 8455 miRNAs from 73 plant species in miRBase 21.0 (http://www.mirbase.org) [35] and 11183 miRNAs out of the 15041 miRNAs from 150 plant species in the Plant Non-coding RNA Database (PNRD) (http://structuralbiology.cau.edu.cn/PNRD/) [36]. The miRNAs identified in the halophytes *S. brachiata* and *S. europaea* have yet to be included in miRBase, whereas those identified in *A. marina* are only partially included.

Because of ambiguities in the available information on the miRNAs involved in tolerance to salinity, which is a serious abiotic stress affecting crops worldwide, as well as the availability of limited information regarding salt-responsive miRNAs in naturally salt-tolerant plants, the present work was conducted to identify miRNAs and their targets and study their responses to salt application in *S. maritima*, a halophyte that grows naturally along the seashore and has been described as a well-suited plant for studying salt stress response because of its ability to grow in the presence and absence of salt [2]. The halophyte *S. maritima* has been exploited worldwide for the physiological and molecular characterization of salt tolerance in plants [37–39]. Recently, the possible involvement of antioxidative machinery in salt tolerance in *S. maritima* was also investigated [40, 41].

The current study was also designed to explore the possibility of identifying miRNAs in *S. maritima* using the EST database for two mangrove plants, *Rhizophora mangle* and *Heritiera littoralis*, to establish whether the transcriptome data of one species could be utilized to identify miRNAs in other species with confidence. In the present study, seven novel salt-responsive miRNAs and several conserved miRNAs were identified and validated experimentally in test plants. This study also investigated the response of these novel miRNAs to NaCl application and/or their presence in taxonomically and ecologically diverse plant species, including two rice cultivars (salt-tolerant *O. sativa* cv. Pokkali and non-tolerant *O. sativa* cv. Badami) and several plant species growing naturally under the direct influence of seawater, such as *Sesuvium portulacastrum*, *Cyperus arenarius*, *Ipomoea pes-caprae* and *S. maritima*.

## Methods

### Test plant species and NaCl application

The seeds of *S. maritima* L. were collected from adult plants growing along a mangrove coastal belt in Bhadrak (21.13°N, 86.76°E), Odisha, India. The seeds were spread on autoclaved soil in plastic pots with holes at the bottom and watered every day, alternating between 1/10^th^ Hoagland’s solution and Milli-Q water. The seedlings were allowed to grow in a growth chamber maintained at 24 ± 3 °C, 70–75 % relative humidity, and a 14 h of light (200 μmol m^−2^ s^−1^)/10 h of dark cycle. After 3–4 weeks, the seedlings were approximately 2 cm in height. At this stage, the seedlings were transferred to soil in plastic pots. The seedlings were allowed to acclimatize and grow for ~3 months under a natural day/night cycle in a greenhouse maintained at 24 ± 3 °C and 70–75 % relative humidity. The individual pots were watered every day, alternating between 1/10^th^ Hoagland’s solution and Milli-Q water except on the penultimate day of NaCl application. For the NaCl application, 500 ml of 85 mM NaCl prepared in 1/10^th^ strength Hoagland’s solution was poured into the individual pots early in the morning. The control pots received only 1/10^th^ Hoagland’s solution. After 30 min, 100 ml of 340 mM NaCl prepared in 1/10^th^ strength Hoagland’s solution was poured into the treated pots at 30 min intervals. Seeds of the rice cultivars *O. sativa* cv. Badami and cv. Pokkali were collected from the Orissa University of Agriculture and Technology (OUAT), Bhubaneswar and Central Rice Research Institute (CRRI), Cuttack, respectively, and then germinated on moist filter paper in petri dishes. The germinated seeds were grown on 1/10^th^ Hoagland’s solution in 200 ml beakers for 7 days in the greenhouse under the same conditions mentioned above. Half an hour before switching on the light, the seedlings were treated with 85 mM NaCl, and then the concentration was increased to 255 mM. After 9 h of exposure to NaCl, the aerial portions of the test plants were excised, preserved in liquid N_2_ and stored at −80 °C until use. The treatment of all the plants continued up to 96 h for the measurement of leaf fluorescence.

The NaCl treatment concentration for the individual test plants was based on lab observations and reports that 340 mM NaCl promoted the growth of *S. maritima*, 255 mM NaCl exerted slight inhibitory effects on the growth of *O. sativa* cv. Pokkali seedlings, and 255 mM NaCl significantly inhibited the growth of *O. sativa* cv. Badami [42, 43]. However, the selection of test plants for the experiment was not only based on their level of salt tolerance. Although *S. maritima* grows along seashores, *O. sativa* cv. Pokkali, which is fairly salt-tolerant, is a cultivated rice that does not naturally grow in saline environments. However, *O. sativa* cv. Badami is a cultivated rice that is not tolerant to salt. Additional plants, specifically *S. portulacastrum*, *C. arenarius* and *I. pes-caprae*, were collected from their natural environments along the seashore. *S. maritima* was also collected from its natural environment for the study. Samples of these plants were immediately preserved in liquid N_2_ for analysis. The taxonomic details of the plants are provided in Additional file 1.

### Measurement of electron transport rate

The effect of NaCl on the test plants grown in the lab was studied in terms of its influence on photosynthetic electron transport in the intact leaves. The parameter was estimated from the fluorescence data obtained using a field model pulse amplitude modulated fluorometer (Hansatech, UK). Before starting the measurement, the plants were kept in the dark for 30 min. The chlorophyll fluorescence parameters were obtained for individual leaves from *S. maritima* and the cultivars of *O. sativa* (control and treated with NaCl for 9 h, 24 h and 96 h) under saturating light pulses of > 6000 μmol m^−2^ s^−1^ and actinic light of 400 μmol m^−2^ s^−1^. These parameters were used to calculate relative electron transport rate (ETR), which reflects the overall photosynthetic capacity in vivo according to the equation $\mathsf{E}\mathsf{T}\mathsf{R}=\Phi \mathsf{PSII}\mathsf{x}\mathsf{PFDa}\mathsf{x}\left(\mathsf{0.5}\right),$ where PFDa is the absorbed light (considered equivalent to the PPFD (photosynthetically active photon flux density) in μmol m^−2^ s^−1^ for comparative study), ΦPSII is the quantum yield of photosystem II and 0.5 is a factor that accounts for the partitioning of energy between PSII and PSI. ΦPSII is given as the ratio of Fq’/Fm’, where Fq’ is the difference in fluorescence between Fm’ and F’ and represent the maximal fluorescence and steady state fluorescence emissions, respectively, under actinic light [44]. The ETR was determined for five leaves from the control and NaCl-treated plants for all exposure durations.

### Small RNA library construction and sequencing

Total RNA was extracted from *S. maritima* tissue using TRIzol reagent (Invitrogen, USA) following the manufacturer’s instructions. Small RNA libraries, one each for the control and NaCl-treated plants, were prepared with a TruSeq Small RNA Prep Kit (Illumina, Inc.) using the Illumina TruSeq Library preparation protocol according to the manufacturer’s instructions. Briefly, total RNA (1 μg) was separated on a denaturing polyacrylamide gel, and sRNAs of 16–29 nt were recovered. Adaptors were ligated to each end of the isolated sRNAs, and RT reactions were used to create single-stranded cDNA. The cDNA was then PCR amplified and separated using 6 % PAGE, and bands corresponding to the miRNA fragments were purified. The final library was quality checked using an Agilent Bioanalyzer DNA1000 chip. Both sRNA sequencing libraries were normalized to 2 nM with Tris–HCl, denatured using NaOH, diluted to 7 pM using pre-chilled Illumina TruSeq Hybridization Buffer and hybridized onto an Illumina Paired-End Flow cell followed by cluster amplification using an Illumina cluster station with a TruSeq Cluster Generation Kit V5.0 as per the manufacturer’s instructions. Single-end sequencing of 36 nt was performed using an Illumina Genome Analyzer IIx with a TrueSeq SBS kit V5.0. Base calling and FASTQ data conversion were performed using the Illumina pipeline CASAVA 1.8 package. The deep sequencing data has been submitted as SRA file at the NCBI (BioProject ID: PRJNA293256).

### Small RNA bioinformatics analysis

The raw reads were filtered to remove low-quality reads, and the reads that passed the quality filter were trimmed to remove the adaptor sequences. Selected reads of 16 nt to 29 nt were then queried against the NCBI and Rfam databases to discard abundant non-coding RNAs (rRNA, tRNA, snRNA, and snoRNA). The remaining unique small RNA reads (16 nt to 29 nt) were BLASTN searched against known plant miRNAs in the miRBase 21.0 and PNRD databases to identify conserved miRNAs. Only perfectly matched reads were considered to be conserved miRNAs. To explore the occurrence of novel miRNAs, the sRNA reads were mapped on the ESTs of two mangrove plants*, R. mangle* (SRX001383) and *H. littoralis* (SRX001410), after de novo assembly of the raw reads available in the NCBI database (http://www.ncbi.nlm.nih.gov/sra/?term=SRP000300), considering that genes expressed in these two plants must be qualitatively similar to *S. maritima* because of similarity in their natural habitats. The secondary structures were predicted using mfold (http://mfold.rna.albany.edu/?q=mfold/rna-folding-form) [45] and analyzed for stable stem-loop hairpins using criteria described in [46] and [47]. Briefly, the criteria included the following: 1) the sRNA sequence matches perfectly with the precursor sequence, 2) the mature miRNA occupies only one arm of the hairpin, 3) the mature miRNA sequence has no more than six mismatches with the sequence on the opposite arm, 4) the minimum free energy (MFE) is less than or equal to −15 kcal/mol, and 5) the minimum free energy index (MFEI) is more than 0.40.

The presence of these precursors was verified in the ESTs of *S. maritima* at Bionivid, Bangalore, India, which agreed to the limited use of the transcriptome data.

### Northern blot analysis

Total RNA was isolated from the tissue samples of the control plants and 340 mM NaCl-treated (9 h) *S. maritima* using miRNeasy mini kit (Qiagen) according to the manufacturer’s instructions. For the Northern blot analysis, 10 μg of total RNA was resolved on a 15 % urea-PAGE gel. The electrophoresed RNA was transferred onto a nylon membrane using a Trans-Blot^®^ SD Semi-Dry Electrophoretic Transfer Cell (Bio-Rad). The blot was air dried and UV cross-linked at 150 mJ using a UV cross-linker (Hoefer™ UVC 500 Crosslinker). Probes designed from DNA oligonucleotides complementary to the miRNA sequences (Additional file 2) were end-labeled with [γ-^32^P]dATP using T4 Polynucleotide Kinase (Fermentas) according to the manufacturer’s instructions. The membrane was pre-hybridized for 1 h with hybridization buffer (Sigma), and then the labeled probe was added and allowed to hybridize for 16 h at 37 °C. After hybridization, the membrane was washed with 2X SSC and 0.1 % SDS at 32 °C for 15 min and 1X SSC and 0.1 % SDS for 15 min at 37 °C. The membrane was air dried and then exposed to X-ray film. When required, the membrane was stripped, re-exposed to the X-ray film to ensure complete signal removal and reused for a second hybridization. A DNA oligonucleotide complementary to U6 snRNA was used as a probe to detect the U6 snRNA for use as an internal control.

### Stem-loop PCR (TaqMan miRNA assay)

A TaqMan miRNA assay was conducted to examine the expression of novel miRNAs and several conserved miRNAs in the test species and to study the relative changes in their levels in response to NaCl application [48]. The TaqMan miRNA assay for the individual novel miRNAs was custom designed at Applied Biosystems (Additional file 2) and obtained as kits, each consisting of 1) miRNA-specific RT primer and 2) mixture of miRNA-specific forward and reverse primers and miRNA-specific TaqMan MGB (minor groove binder) probe. For the conserved miRNAs, the assay kits were available. Total RNA was isolated from the tissue samples of the control and NaCl-treated plants (*S. maritima*, *O. sativa* cv. Badami, and *O. sativa* cv. Pokkali) and from those collected from the natural environment (*S. maritima*, *S. portulacastrum*, *C. arenarius* and *I. pes-caprae*) using miRNeasy mini kit (Qiagen) according to the manufacturer’s instructions. To create cDNA for each TaqMan miRNA assay, 20 ng of total RNA was incubated with 0.15 μl of dNTPs (100 mM), 1.5 μl of 10X reverse transcription buffer, 0.19 μl of RNase inhibitor (20 U μl^−1^), 1 μl of reverse transcriptase (50 U μl^−1^), and 3 μl of stem-loop reverse transcription primer (specific for individual miRNAs) in a 15 μl reaction. The real-time PCR for each assay was set up as a 20 μl reaction containing 10 μl of TaqMan 2X Universal PCR master mix, 1 μl of 20X TaqMan Assay mix that included miRNA-specific primers and TaqMan probe, and 1.33 μl of cDNA. A TaqMan Assay® probe for 18S that was designed according to the homologous nucleotide sequences of *S. maritima* and *O. sativa* was used as the endogenous control for normalization of the Ct values of the miRNAs. The same probe also worked for the other plant species. A LightCycler^®^ 480II (Roche) was used for the real-time PCR with the following cycling conditions: 95 °C for 10 min and then 40 cycles of 95 °C for 15 s and 60 °C for 1 min. The TaqMan assay reactions for each miRNA in a biological sample (cDNA preparation) were performed in triplicate, and each of the PCR setups included a template-free well. Two biological samples were considered for *S. maritima* for the TaqMan assay, but only one sample was used for the other plants. The relative expression or abundance of miRNA in the NaCl-treated plants relative to that in the control plants was calculated using the 2^-ΔΔCT^ method [49]. A paired *t*-test was performed to determine significant differences (P ≤ 0.05) in the abundances of miRNA in the control and NaCl-treated plants, whereas Duncan’s multiple range test was used to determine significant differences in the responses of individual miRNAs to NaCl among the test plants [50]. The results of the TaqMan assays for the miRNA in the plants collected from the natural environment were presented as the Ct values of the individual miRNAs per unit 18S Ct values in each respective species.

### Target prediction and validation

Target prediction of the experimentally validated conserved and novel salt-responsive miRNAs was performed according to the de novo-assembled ESTs of the halophytes *R. mangle* and *H. littoralis* in the lab and using the ESTs of *S. maritima* at Bionivid, Bangalore, India. The target predictions were performed using psRNATarget analysis tool available online (http://plantgrn.noble.org/psRNATarget/) [51]. The maximum expectation value (measures the complementarity between small RNA sequences and its target transcripts), the hsp size (length for complementary scoring) and target accessibility were set at 3.5, 17 and 25, respectively. The range of central mismatch leading to translation inhibition was between 9 and 11 nt. The predicted target transcript sequences were BLASTX searched at the NCBI site for annotation of their probable functions. Primer pairs (Additional file 2) were designed for several of the target sequences to examine their expression by RT-qPCR using cDNA prepared from the RNA extracted from the control and 340 mM NaCl-treated *S. maritima*. A QuantiTect Reverse Transcription Kit (Qiagen) was used to convert the RNA to cDNA. The kit provides an optimized mix of oligo-dT and random primers and gDNA Wipeout Buffer. The RT-qPCR was run on a LightCycler^®^ 480 Real-Time PCR System (Roche) using QuantiTect SYBR Green PCR Kit (Qiagen). Actin served as the reference gene. Two biological samples each from the control and 340 mM NaCl-treated *S. maritima* were used for cDNA preparation. The PCR was performed in triplicate for each cDNA preparation. The paired *t*-test was performed to determine significant differences (P ≤ 0.05) of expression in the genes of the control and NaCl-treated plants.

## Results

### Small RNA sequencing and data analysis

Additional file 3 shows the results from the deep sequencing of the sRNA libraries prepared with the total RNA extracted from the shoot tissue samples of young *S. maritima* grown in the absence of NaCl (control, C) and the total RNA of plants treated with 340 mM NaCl (treated, T) for 9 h before RNA extraction. In both cases, more than 16 million raw sRNA reads were generated on an Illumina next generation sequencing platform, and more than 2.7 million of these reads were unique. Adaptor removal and filtering of the < 16 nt and > 30 nt data, t/rRNA matches, etc. yielded 8981591 and 7923122 clean total reads in the control and NaCl-treated libraries, respectively, and they could be putative miRNAs and/or siRNAs. A redundancy analysis in these sequences revealed 1715999 and 1756751 unique/non-redundant reads in the libraries prepared from the control and NaCl-treated plants, respectively. The data showed a greater number of unique reads per unit value of the clean total reads in the NaCl-treated library than in the control library.

### Identification and categorization of conserved miRNA

Although the results indicated the presence of > 1.7 million unique putative miRNA and/or siRNA reads in both the control and NaCl-treated libraries (Additional file 3), only 126 were found in the miRBase 21.0 database. In addition, eight miRNAs were identified in the PNRD. Four of these were computationally predicted and the other four were found from the Illumina sequencing of the sRNAs from *Setaria italica* (Additional file 4). The identified 134 miRNAs were mostly 20-nt and 21-nt in length, but their length varied from 19-nt to 22-nt (Additional file 4). These miRNAs were distributed over 68 plant species (Additional file 4), of which *A. lyrata* had maximum representation, demonstrating matches to as many as 58 miRNAs, followed by *G. max*, *A. thaliana*, and others (Additional file 4). However, only one naturally salt-tolerant plant, *Avicennia marina*, a mangrove species, presented matches, and it was represented in two of the identified miRNAs, vvi-miR396b and ath-miR396b.

**Fig. 1:**
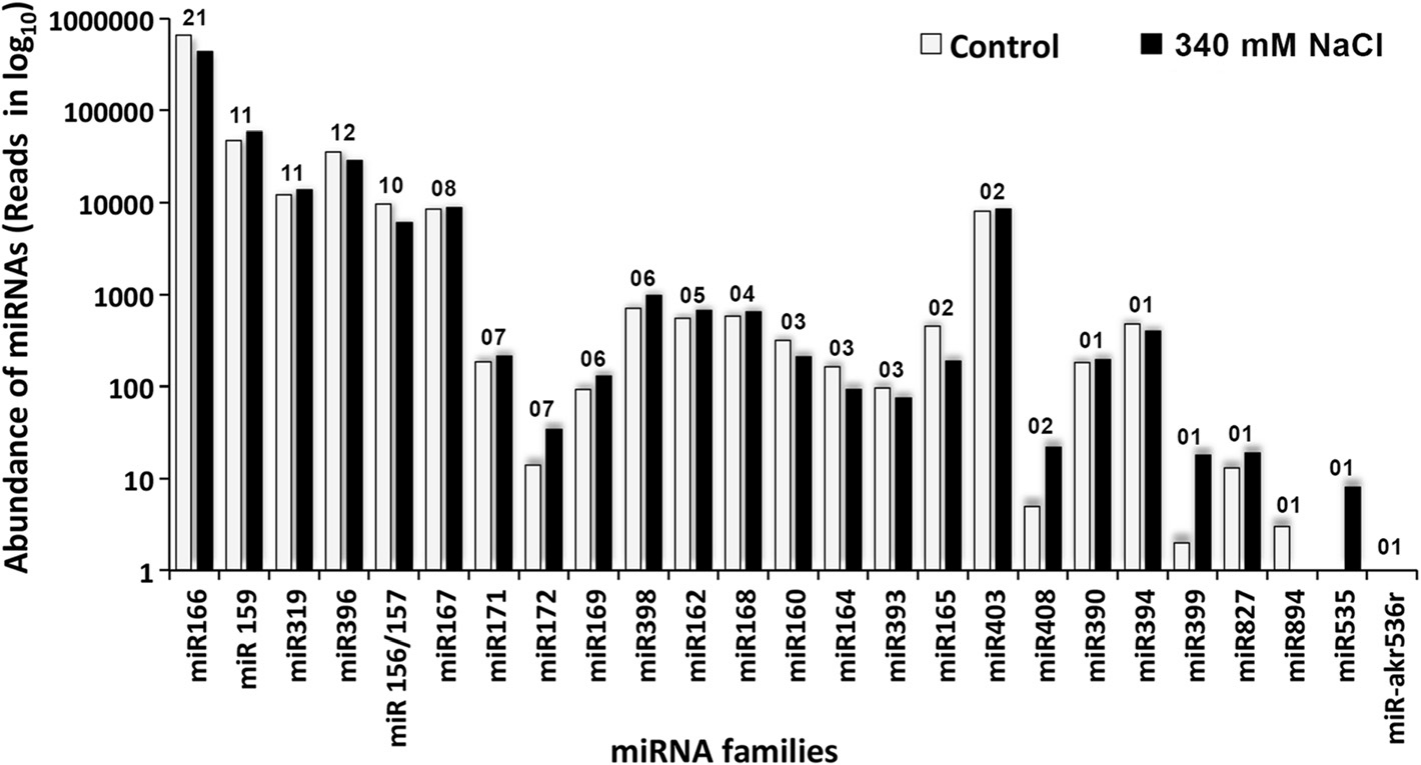
Abundance of the conserved miRNAs and their distribution in the miRNA families. Abundance is expressed in terms of the number of reads in the individual miRNA families in *S. maritima* in the controls and after exposure to 340 mM NaCl for 9 h. The numerical figs. at the top of the bars for the individual miRNA families are the numbers of miRNAs that were found to belong to these families out of 134 known miRNAs identified

The miRNAs identified from both libraries were also found to be highly diverse in nature and belonged to as many as 25 families (Fig. 1). The maximum miRNA representation at up to 21 was observed in the miR166 family, and it was followed by 12 miRNAs in the miR396 family, 11 miRNAs each in the miR159 and miR319 miRNA families, and ten miRNAs in the miR156/157 family. The miR162, miR398, miR169, miR172, miR171 and miR167 miRNA families revealed five or more but less than ten representations, and the remaining miRNA families were represented by less than five miRNAs. In addition to showing the highest number of individual miRNAs, the miR166 family also showed the maximum number of combined reads/abundance (Fig. 1). The NaCl treatment influenced both the number of representative miRNAs and their abundance in the miRNA families (Fig. 1).

### Salt responsiveness of the conserved miRNAs

**Fig. 2:**
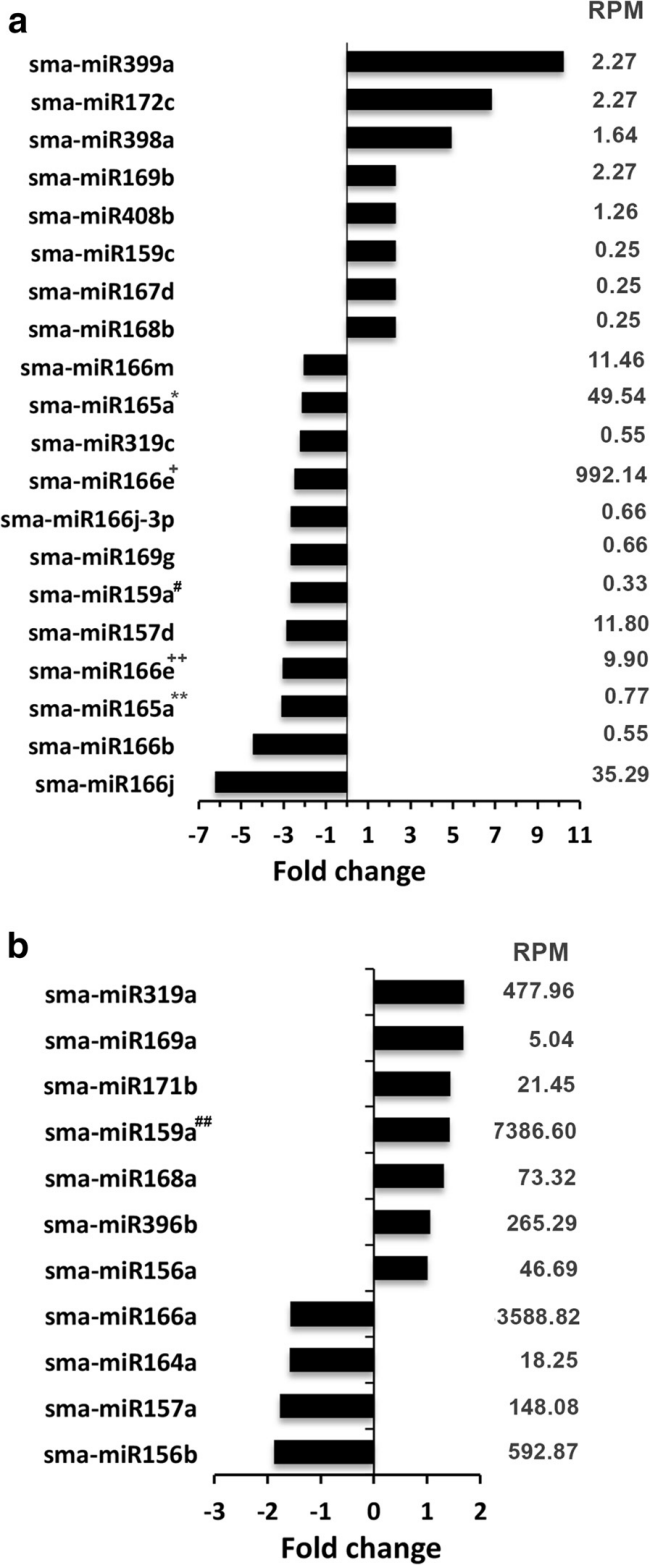
Changes in the abundance of select conserved miRNAs in *S. maritima* in response to exposure to 340 mM NaCl, which is represented as a fold change relative to the control level. **a** The conserved miRNAs that showed twofold or more change. **b** The conserved miRNAs reported to be salt stress responsive. Negative and positive values represent decrease and increase, respectively, in abundance of a miRNA in response to NaCl treatment. RPM- Reads per million; the values are of the control or treated reads, whichever the maximum. The homologous miRNAs of sma-miR165a*, sma-miR165a**, sma-miR166e^+^, sma-miR166e^++^, sma-miR159a^#^ and sma-miR159a^##^ are ath-miR165a, aly-miR165a, bdi-miR166e, osa-miR166e, pta-miR159a and ath-miR159a respectively

To determine the salt responsiveness of the identified miRNAs, their relative abundance in the control and NaCl-treated samples was calculated and represented as the fold change after NaCl treatment relative to the control level (Fig. 2). The results of only those miRNAs have been presented that either showed more than twofold change in abundance in response to NaCl exposure (Fig. 2a) or that showed less than twofold NaCl-induced changes in abundance, but reported to be stress responsive (Fig. 2b). The analysis showed great variation in the responses of miRNAs after exposure of the plants to NaCl, and the abundance of sma-miR166j decreased by more than sixfold while that of sma-miR399a increased by more than tenfold in response to the NaCl treatment (Fig. 2a). Among the miRNAs showing less than a twofold change in response to NaCl, the maximum upregulation was observed for sma-miR319a and the maximum downregulation was observed for sma-miR156b (Fig. 2b). In addition, most of the miRNAs that were present in high abundance with RPM (reads per million) of 50 or more showed less than a twofold change in response to salt treatment.

### Characterization of the conserved miRNAs

**Fig. 3:**
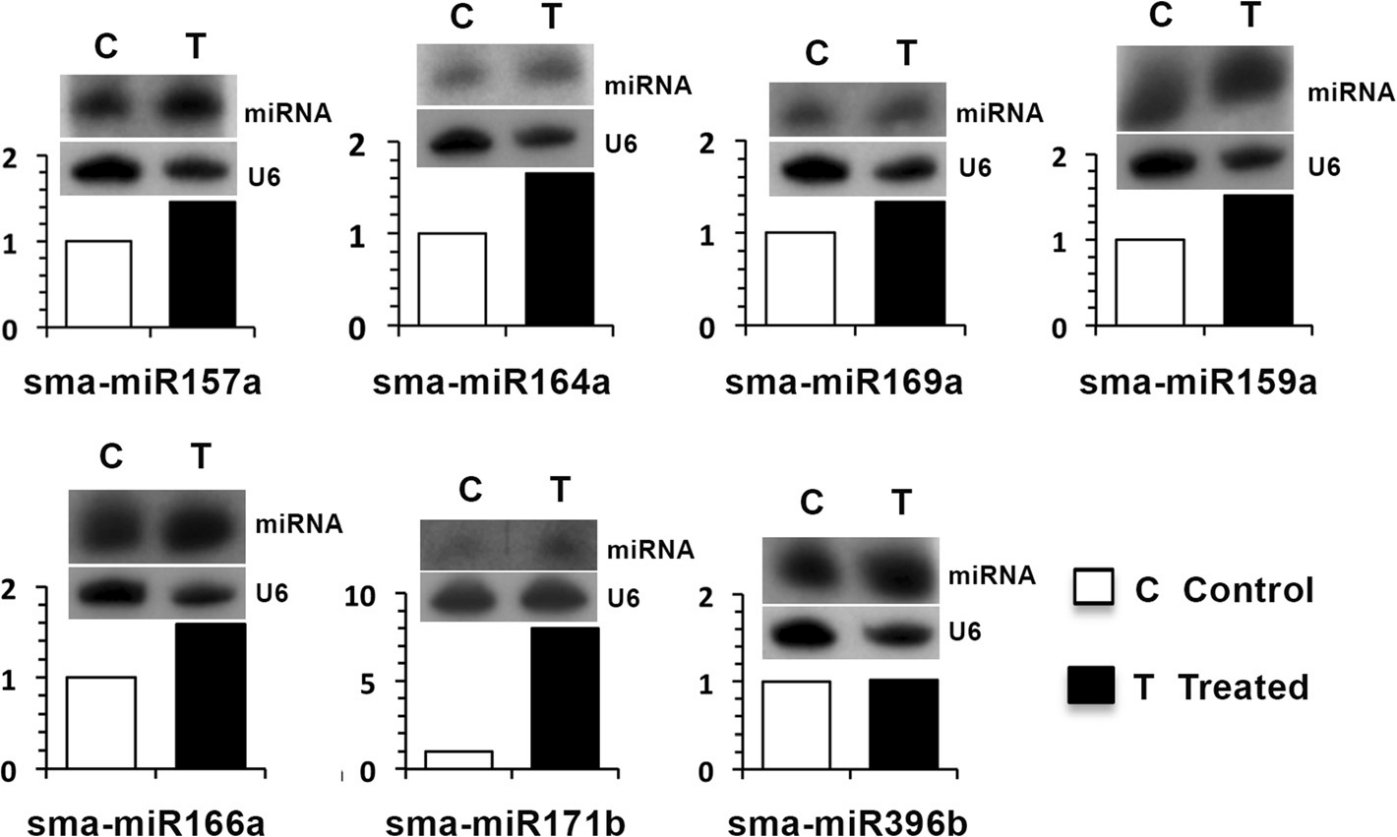
Changes in the expression of select conserved miRNAs in *S. maritima* in response to exposure to 340 mM NaCl, as determined by Northern blot analysis. The upper blot of each panel represents a hybridization signal of the anti-sense probe with a specific miRNA. U6 served as the loading control and is shown in the lower panel for the individual miRNA blot analyses. The signal intensities of U6 and miRNAs were analyzed densitometrically and plotted as a histogram representing relative changes in the hybridization intensities in the NaCl (340 mM) treated sample (filled bar) relative to the control sample (empty bar)

The expression of several conserved miRNAs in *S. maritima*, particularly those showing high abundance in the deep sequencing results, was confirmed by identifying precursors capable of forming hairpins (Additional file 5) in the ESTs of the species (available at Bionivid, Bangalore) and by Northern blot hybridization (Fig. 3). These precursor ESTs could be amplified by RT-PCR (Additional file 5). The details of the precursors are provided in Additional files 4 and 6. The MFEIs of the precursors varied from 0.46 to 1.09 with an average value of 0.77 (Additional file 4). The results from the Northern blot analyses for several of the conserved miRNAs, such as sma-miR157a, sma-miR164a and sma-miR166a, indicated upregulation (Fig. 3), which was inconsistent with the results of the abundance analysis (Fig. 2). However, most of the miRNAs, such as sma-miR159a, sma-miR171b and sma-miR169a, were upregulated in the Northern blot analyses in response to the NaCl treatment (Fig. 3), which is similar to the results of the abundance analysis (Fig. 2) except for that of the miRNA sma-miR396b, which did not present changes in its levels. Although these miRNAs have been reported to occur in a wide range of plant species encompassing monocots, dicots and pteridophytes, only one among them, sma-miR396b, has been reported from a halophyte, *A. marina* (Table 1). These miRNA have also been reported to be responsive to one or more abiotic stresses. In most cases, the presence of all of these miRNAs has been confirmed by sequencing, cloning and Northern blot analysis in the concerned species (Table 1), similar to that in the present species.

**Table 1:** Identification and Characterization of *S. maritima* miRNAs that are conserved in other plants

Family	miRNA	Sequence	Other plant species	Validation method	Stress response
miR156/157	sma-miR157a	uugacagaagauagagagcac	aly, gma, ath, lus, ptc, mdm, mes, cme, ppe, mtr, vvi, tcc, rco, bna, stu, cpa, cca, sly, vun, bra, ahy, bol, gra, han	Cloned, Northern, 3’ RACE, 454, MPSS, Illumina	Drought and biotic stress [70], salt stress [60, 71]
miR159	sma-miR159a	uuuggauugaagggagcucua	aly, gma, ath, ptc, mes, cme, nta, ppe, mtr, vvi, rco, bna, csi, cpa, sly, bra, hbr, htu, ahy, pvu	Cloned, Northern, 5’ RACE, 454, MPSS, Illumina	Drought and biotic stress [70], Mechanical stress [72], biotic and drought stress [70, 73]
miR164	sma-miR164a	uggagaagcagggcacgugca	aly, gma, ath, lus, ptc, mdm, mes, cme, nta, ppe, osa, mtr, vvi, tcc, bdi, zma, rco, bna, csi, sbi, cpa, ssl, ghr, tae, ctr, bra	Cloned, Northern, 5’ RACE, 454, MPSS, Illumina	Mechanical stress [72]
miR166	sma-miR166a	ucggaccaggcuucauucccc	aly, gma, lus, mdm, mes, cme, nta, ppe, vvi, tcc, bdi, rco, bna, stu, csi, aqc, cpa, sly, ssl, dpr, ctr, hbr, ssp, hvu, hpe, pvu, hpa	Cloned, Northern, 5’ RACE, 454, MPSS, Illumina	Drought and biotic stress [73], Drought stress [74], dehydration stress [75], cold and dehydration stress [76]
miR169	sma-miR169a	cagccaaggaugacuugccga	aly, gma, ath, lus, ptc, mes, nta, osa, mtr, vvi, tcc, bdi, zma, bna, sbi, sly	Cloned, Northern, 5’ RACE, 454, MPSS, Illumina	Drought stress [74]
miR171	sma-miR171b	ugauugagccgugccaauauc	gma, lus, ptc, mdm, mes, cme, nta, ppe, osa, mtr, vvi, tcc, bdi, zma, rco, stu, sbi, aqc, cpa, sly, tae, htu, lja, crt, hvu, far, pde, hpa	By similarity	Nutrient starvation [77], mechanical stress [72], drought stress [74], dehydration stress [75]
miR396	sma-miR396b	uuccacagcuuucuugaacuu	aly, gma, ath, lus, ptc, mdm, mes, cme, nta, ppe, osa, mtr, tcc, bdi, zma, rco, bna, stu, sbi, aqc, cca, pta, bgy, bcy, ama	Northern, 5’ RACE, 454, MPSS, PCR	Nutrient starvation [77], drought and biotic stress [70], drought stress [74], salt stress [30, 60]

aly *A* *rabidopsis* *lyrata*, gma *G* *lycine max*, ath *A* *rabidopsis thaliana*, lus *L* *inum usitatissimum*, ptc *P* *opulus trichocarpa*, mdm *M* *alus domestica*, mes *M* *anihot esculenta*, cme *C* *ucumis melo*, nta *N* *icotiana tabacum*, ppe *P* *runus persica*, osa *O* *ryza sativa*, mtr *M* *edicago truncatula*, vvi *V* *itis* *vinifera*, tcc *T* *heobroma cacao*, bdi *B* *rachypodium distachyon*, zma *Z* *ea mays*, rco *R* *icinus communis*, bna *B* *rassica napus*, stu *Solanum tuberosum*, csi *C* *itrus sinensis*, sbi *S* *orghum bicolor*, aqc *A* *quilegia caerulea*, cpa *C* *arica papaya*, cca *C* *ynara cardunculus*, sly *S* *olanum* *lycopersicum*, vun *V* *igna unguiculata*, ssl *S* *alvia sclarea*, ghr *G* *ossypium hirsutum*, tae *T* *riticum aestivum*, dpr *D* *igitalis purpurea*, aau *A* *cacia auriculiformis*, ctr *C* *itrus trifoliate*, bra *B* *rassica rapa*, *hbr* *H* *evea brasiliensis*, ssp *Saccharum* ssp., htu *H* *elianthus tuberosus*, lja *L* *otus japonicas*, ccl *C* *itrus clementine*, crt *C* *itrus reticulate*, smo *S* *elaginella moellendorffii*, ahy *A* *rachis hypogaea*, hvu *H* *ordeum vulgare*, bol *B* *rassica oleracea*, amg *A* *cacia mangium*, hpe *H* *elianthus petiolaris*, pta *P* *inus taeda*, far *F* *estuca arundinacea*, pvu *P* *haseolus vulgaris*, gra *G* *ossypium raimondii*, pde *P* *inus densata*, hpa *H* *elianthus paradoxus*, bgy *B* *ruguiera gymnorhiza*, bcy *B* *ruguiera cylindrical*, han *H* *elianthus annuus*, har *H* *elianthus argophyllus*, ama *A* *vicennia* *marina*

### Identification of novel miRNAs

**Fig. 4:**
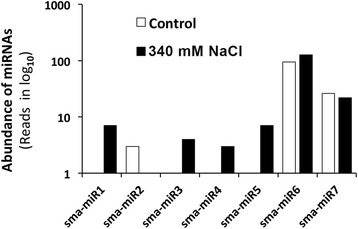
Abundance of novel miRNAs in *S. maritima*. Abundance is expressed in terms of the numbers of reads in the controls and after exposure to 340 mM NaCl for 9 h

A total of 13 potential candidate miRNAs without matches in the database were identified after mapping the sRNA reads on the ESTs of *R. mangle* and *H. littoralis* and hairpin predictions (Additional files 4 and 5). However, the precursors of only seven novel miRNAs could be identified in the ESTs of *S. maritima*, and these precursor ESTs could also be amplified by RT-PCR (Additional file 5). The MFEs of the precursors identified in both *S. maritima* and the mangrove plants, the lengths of the pre-miRNAs, the sequences of the matured miRNAs and other details are provided in Table 2 and Additional file 4. The MFEIs of the precursors of the novel miRNAs identified from the *S. maritima* transcripts varied from 0.44 to 0.74 and had an average value of 0.59 (Additional file 4). The novel miRNAs identified showed great differences in their abundances (Fig. 4), with sma-miR6 the most abundant, followed by sma-miR7. The other miRNAs were low in abundance and present in either the control (sma-miR2) or NaCl-treated plants (sma-miR1, sma-miR3, sma-miR4 and sma-miR5).

**Table 2:** Novel miRNAs predicted through bioinformatics approach

miRNAs	Sequence (5’-3’)	LM (nt)	Precursor accession^b^	LP (nt)	MFE (Kcal/mol)
			(*R. mangle*		
			*H. littoralis*		
			*S. maritima*)		
sma-miR1	AAUAGGUACUGUAACUGGUAUU	22	SRX001383.5101	126	−37.8
			NP	--	--
			*S.maritim*a380087	85	−15.60
sma-miR2	AGGGACCAGGAGAUUGGAUC^a^	20	SRX001383.146244	75	−19.0
			NP	--	--
			*S.maritim*a40845	73	−15.20
sma-miR3	CGGAAUAUGGUAAAGUAGCUC	21	SRX001383.195972	70	−19.7
			NP	--	--
			*S.maritima*1868968	70	−20.10
sma-miR4	CGUGGAUGUUCUUAUUUGGAC	21	SRX001383.88652	116	−30.4
			SRX001410.01205	141	−30.53
			*S.maritima*870097	115	−29.0
sma-miR5	CUUGGUAUGGAAGUUAUGCAUG^a^	22	SRX001383.176415	71	−19.7
			NP	--	--
			*S.maritima*37179	69	−20.10
sma-miR6	GCAUGGCUGUCGUCAGCUCGUG	22	SRX001383.110283	67	−33.5
			NP	--	--
			*S.maritima*40845	89	−21.60
sma-miR7	UUUUCUUGACCUUGUAAGACC	21	SRX001383.115761	90	−40.8
			NP	--	--
			*S.maritima*39969	134	−28.73

Prediction was done after alignment of the putative miRNA sequences with the ESTs of the mangrove plants *R. mangle* and *H. littoralis* available at NCBI database and then with ESTs of *S. maritima* at Bionivid, Bangalore, India. SRX001383 and SRX001410 are the accession numbers of *R. mangle* and *H. littolalis*, respectivey in NCBI database. *LM* mature miRNA length, *LP* precursor length, *MFE* minimum free energy, *NP* precursor not present

^a^mark against a sequence indicates the presence of miRNA*

^b^see Additional file 4 for sequences and other details

### Expression analysis of selected miRNAs by quantitative PCR

**Fig. 5:**
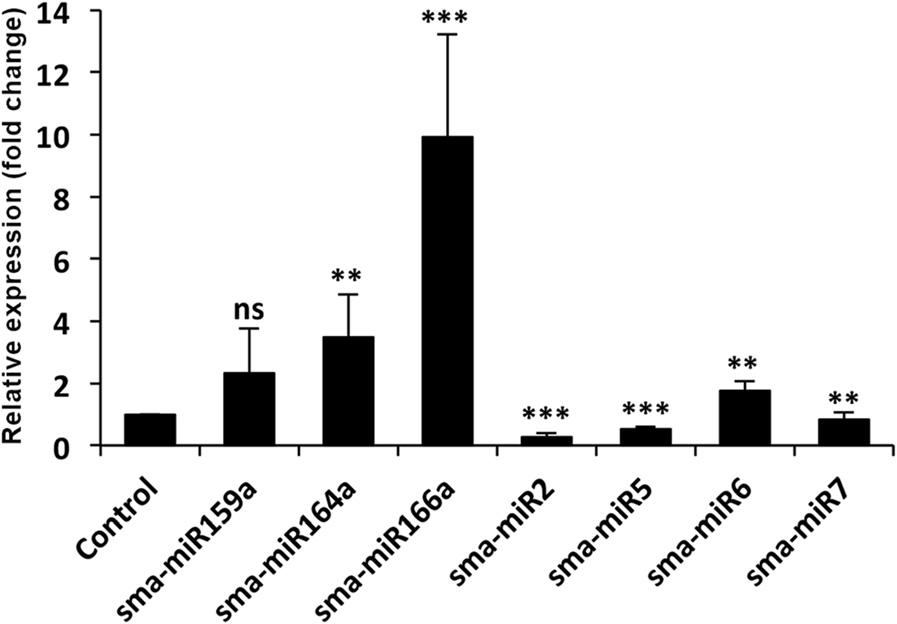
Changes in the expression of select miRNAs in *S. maritima* in response to exposure to 340 mM NaCl as determined by stem-loop PCR. The bars for the individual miRNAs represent fold changes in their expression in response to 340 mM NaCl treatment relative to the mean expression level of the control plants. The data are the mean ± SD of six independent estimations. Asterisks in the individual columns indicate that NaCl-responsive changes in the expression of the respective miRNAs differed significantly from the control expression level at p ≤ 0.05 (*), p ≤ 0.01 (**), or p ≤ 0.001 (***). ns = not significant

To further confirm the presence of miRNAs identified in the test species and their differential expression, a stem-loop PCR analysis (TaqMan assay) was performed using three representative conserved miRNAs and all of the novel identified miRNAs. All three conserved miRNAs showed amplification Ct values < 30 and were upregulated in response to NaCl treatment of the plants (Fig. 5), confirming the results obtained by the Northern blot analysis (Fig. 3). High upregulation was observed for sma-miR166a, which is similar to the result obtained with Northern blotting. Among the novel miRNAs showing Ct values < 30 (Additional file 7), sma-miR2 and sma-miR7 were downregulated and sma-miR6 was upregulated (Fig. 5), which is similar to the results of the abundance analysis (Fig. 4). The stem-loop PCR although demonstrated inconsistent results with that of the abundance analysis for the sma-miR5, it generally validated the results of the abundance analysis for the miRNAs and the Northern analysis for the conserved miRNAs. A paired *t*-test revealed the significant influence of NaCl on the expression of all of the miRNAs assessed by the TaqMan assay except for sma-miR159a (Fig. 5). Three predicted novel miRNAs (sma-miR1, sma-miR3 and sma-miR4) were not amplified in the stem-loop PCR. The traces of progress for one representative PCR for each novel miRNA are provided in Additional file 7.

### Effects of NaCl on the photosynthetic electron transport rates of the test plants

The influence of NaCl on the photosynthetic electron transport of the intact leaves of the test plants was assessed because photosynthesis is a metabolic activity unique to plants, and it is widely influenced by environmental conditions. An analysis of the photosynthetic electron transport rate (ETR) revealed that NaCl only produced significant effects in the rice cultivars, and the ETRs of both Badami and Pokkali decreased significantly during all NaCl treatment periods (Additional file 8). However, the effects were more drastic in Badami than in Pokkali, particularly during the early periods (9 h and 24 h) of exposure as observed from the ETR values presented as the percent of control in the inset (Additional file 8). The halophyte *S. maritima* did not show significant changes of ETR in response to NaCl, even after a long period (96 h) of exposure.

### Analysis of the presence of the novel miRNAs in other plant species

**Fig. 6:**
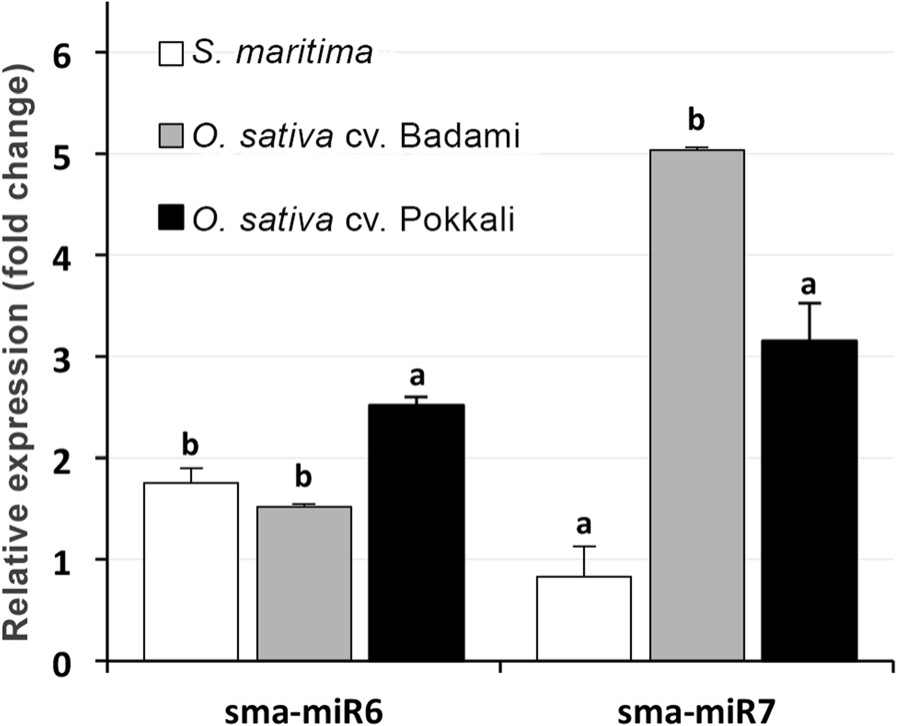
Changes in the expression of sma-miR6 and sma-miR7 in the test plants in response to their exposure to 255 mM NaCl (*O. sativa* cv. Badami/Pokkali) or 340 mM NaCl (*S. maritima*) for 9 h. The bars for the individual miRNAs represent fold changes in their expression in the three test plants in response to NaCl treatment relative to the mean expression level in the control plants. The data are the mean ± SD of six independent estimations for *S. maritima* and 3 independent estimations for the other plants. Variations in the expression of the individual miRNAs in the four plants resulting from NaCl treatment were statistically analyzed with an ‘F’ test and Duncan’s multiple range test. The mean fold change in the expression of an miRNA (bars) of individual species marked with at least one common letter do not differ significantly at p ≤ 0.05

The novel miRNAs showing Ct values < 30 in *S. maritima* were evaluated for their presence in the salt-tolerant Pokkali and intolerant Badami cultivars. Only two miRNAs, sma-miR6 and sma-miR7, showed amplification (Ct ~30 or less) in the two rice cultivars (Additional file 7). However, their response to the NaCl applications was found to differ significantly among the species (Fig. 6). While the expression of sma-miR7 exhibited downregulation in *S. maritima*, its expression increased in the rice cultivars in response to NaCl, and the upregulation was more pronounced in Badami compared to that in Pokkali. Compared with sma-miR7, sma-miR6 was upregulated in all three test plants, with *O. sativa* cv. Pokkali showing significantly greater upregulation compared with the other two plants. To determine why only sma-miR6 and sma-miR7 and not sma-miR2 and sma-miR5 was expressed in the rice cultivars, these sequences were BLASTN searched in the rice databases, and matches were found for the former two sequences with two ESTs (Additional file 4: gi|88967261 and gi|88475521) but not for the latter sequences. Although they were not observed in miRBase, these two ESTs formed the required hairpin structure for Dicer action (Additional file 5) and could be miRNA precursors.

A TaqMan assay conducted for the above novel miRNAs in *S. portulacastrum*, which was collected from the natural habitat of *S. maritima*, showed amplification of all of the miRNAs that were expressed in *S. maritima*, including sma-miR2 and sma-miR5, which were not expressed in the rice cultivars (Additional files 7 and 9A). The expression of sma-miR2 and sma-miR5 was further verified in two additional plant species, *I. pes-caprae* and *C. arenarius*, which grow under the influence of seawater, as well as in *S. maritima* collected from its natural habitat. All of the species demonstrated amplification of the two miRNAs (Additional file 9A). Normalization of the Ct values for the amplification of sma-miR2 and sma-miR5 to the per-unit 18S Ct values in the individual plant species revealed that the abundances of these two miRNAs were in general similar in all four species collected from the natural environment, with *C. arenarius* showing the lowest abundance and *I. pes-caprae* showing the maximum abundance (Additional file 9B).

### Target prediction of miRNAs and study of their expression by qRT-PCR

To understand the biological role of the novel/conserved miRNAs expressed in *S. maritima* as determined by stem-loop PCR or Northern blotting, their putative target genes were searched using the psRNATarget tool. Table 3 shows the putative targets for each miRNA along with other information, such as the miRNA/mRNA pairing, target description and inhibition type (cleavage/translation). Sequences of the target ESTs of both *S. maritima* and the mangrove plants are provided in Additional file 10. Most of the miRNAs targeted more than one gene product, many of which encoded hypothetical and putative uncharacterized proteins. The conserved miRNAs for which the precursors were identified in *S. maritima* and the mangrove plants targeted proteins, such as the F-box family protein, homeobox-leucine zipper, dnaJ, scarecrow-like protein and growth-regulating factor (GRF) in *S. maritima*. In the mangrove plants, the targeted proteins were different from those in *S. maritima*, such as p-ATPase family transporter, RNA helicase, rhodanese-like domain protein, etc. The only target of common function in *S. maritima* and the mangrove plants was an F-box protein targeted by sma-miR159a. The conserved miRNAs that presented precursors only in *S. maritima* also targeted proteins such as lipid phosphate phosphatase, squamosa promoter binding-like protein, F-box protein, ubiquitin-conjugating enzyme 1 and G3BP-like protein. Among the known proteins, the novel miRNAs targeted proteins such as serine/threonine kinase, DEAD-box ATP-dependent RNA helicase, auxin response factor and vacuolar protein sorting associated protein in *S. maritima*. The targets of these miRNAs in the mangrove plants were also primarily proteins with regulatory functions (Table 3). These included serine/threonine kinase, which was targeted by sma-miR5, and auxin response factor and vacuolar protein sorting associated protein, which were targeted by sma-miR7.

**Table 3:** Targets of the novel/conserved miRNAs that expressed in *S. maritima*

miRNAs	*S. maritima* target accession^a^/	Inhibition type/	*R. mangle* target accession^a^/Target description	Inhibition type/
	Target description	miRNA-mRNA pairing	*H. littoralis* target accession^a^/Target description	miRNA-mRNA pairing
Novel miRNAs with precursors identified in both *S. maritima* and mangrove plants				
sma-miR2	*S.maritima*26422/	Cleavage/	SRX001383.184716/	Cleavage/
	Uncharacterized protein LOC104902506 isoform X1 [*B. vulgaris* subsp. *vulgaris*]	::::.:::::: :::::.:	Hypothetical protein [*C. wallichii*]	::::::::::::::::::::
	*S.maritima*1694886/	Cleavage/	SRX001383.195417/	Cleavage/
	4-coumarate--CoA ligase-like 7 [*E. grandis*]	: ::::::.:::::::…:	Transcriptional corepressor/coregulator seuss-like [*P. trichocarpa*]	:::.: :::::::::.:::
sma-miR5	*S.maritima*155512/	Cleavage/	SRX001383.67404/	Cleavage/
	Uncharacterized protein At4g38062 [*B. vulgaris* subsp. *vulgaris*]	:.::: :::.:::::::::	E3 ubiquitin-protein ligase MBR2 [*P. euphratica*]	:.:::::::::::::.::.:
	*S.maritima*714933/	Cleavage/	SRX001383.163041/	Cleavage/
	Serine/threonine-protein kinase PRP4 homolog isoform X3 [*A. pisum*]	::::::.:::::::…::	Serine/threonine-protein kinase TAO3 [*T. cacao*]	: :::::.:::.:::.::::
			SRX001410.05634/	Cleavage/
			Serine/threonine-protein kinase ATG1 [*G. arboreum*]	:::: :::::::::::::.
	*S.maritima*37141/	Translation/		
	DEAD-box ATP-dependent RNA helicase 58, chloroplastic isoform X1 [*B. vulgaris* subsp. *vulgaris*]	: : :.:::::: :::::::::		
sma-miR6	*S.maritima*26981/	Cleavage/	SRX001383.21117/	Translation/
	Uncharacterized protein LOC104904783 isoform *X*2 [*B. vulgaris* subsp. *vulgaris*]	::: :::.::::::.::::	Hypothetical protein [*H. bilis*]	..: : ::::: :::::.::::
	S.maritima41280/	Cleavage/		
	Hypothetical protein (mitochondrion) [*V. faba*]	::::::::::::::::::::::		
sma-miR7	*S.maritima*7497/	Translation/	SRX001383.178145/	Cleavage/
	Uncharacterized protein LOC100160261 [*A. pisum*]	:.::::::: :::::.:::	Hypothetical protein DICPUDRAFT_75074 [*D. purpureum*]	:::::::::::::::::::::
	*S.maritima*41831/	Cleavage/	SRX001383.116751/	Cleavage/
	Auxin response factor 3 isoform *X*2 [*B. vulgaris* subsp. *vulgaris*]	::::::.::::::::::::::	Auxin response factor 4 [*M. notabilis*]	::::::.::::::::::::::
			SRX001410.09075/	Cleavage/
			Auxin response factor 4 isoform 2 [*T. cacao*]	::::::.:::::::::::::
	*S.maritima*39489/	Translation/	SRX001383.156526/	Translation/
	Vacuolar protein sorting-associated protein 26A [*B. vulgaris* subsp. *vulgaris*]	:::: ::: :::::::.::	Vacuolar protein sorting-associated protein 26B-like [*C. sativa*]	:.:: ::: ::::::::::
Conserved miRNAs with precursors identified in both *S. maritima* and mangrove plants				
sma-miR159a	*S.maritima*41732/	Cleavage/	SRX001383.120796/	Cleavage/
	Uncharacterized protein LOC104883236 isoform X1 [*B. vulgaris* subsp. *vulgaris*]	:::::: ::::. ::::.::	Hypothetical protein TRIUR3_29875 [*T. urartu*]	:::::::::::::::::::::
	*S.maritima*43555/	Cleavage/	SRX001383.174703/	Cleavage/
	Uncharacterized protein LOC104908454 [*B. vulgaris* subsp. *vulgaris*]	::::::..:::.::::: ::	Conserved hypothetical protein [*R. communis*]	:.:::.:: ::::::::::::.
	*S.maritima*36986/	Cleavage/	SRX001383.35722/	Translation/
	F-box family protein [*S. vulgaris*]	:: : .:.:.:::::::::::	F-box protein [*M. notabilis*]	::::.::: :::::::::
	*S.maritima*44432/	Cleavage/	SRX001383.108393/	Cleavage/
	Zinc finger CCCH domain-containing protein 5 isoform *X*2 [*B. vulgaris* subsp. *vulgaris*]	::::.:: :::: :::::.:	Double-stranded RNA-binding protein 2 [*M. notabilis*]	::::: .::::::.:.:.::
sma-miR166a	*S.maritima*19497/	Cleavage/	SRX001383.65087/	Cleavage/
	Uncharacterized protein LOC104904626 [*B. vulgaris* subsp. *vulgaris*]	:::::: :.::.::::..::	P-ATPase family transporter: calcium ion [*O. lucimarinus* CCE9901]	:::::::::::::::::::::
	*S.maritima*32700/	Cleavage/		
	Homeobox-leucine zipper protein REVOLUTA-like [*B. vulgaris* subsp. *vulgaris*]	::.:::::::::::::::.		
sma-miR171b	*S.maritima*13778/	Cleavage/	SRX001383.169033/	Translation/
	F-box/kelch-repeat protein At3g23880-like isoform X1 [*B. vulgaris* subsp. *Vulgaris*]	::: ::::..::::::::::	RNA helicase 36, partial [*D. oppositifolia*]	::::::: : :::::::::
	*S.maritima*44824/	Cleavage/		
	Scarecrow-like protein 6 isoform X1 [*B. vulgaris* subsp. *vulgaris*]	:::::::::.:::::::::::		
sma-miR396b	*S.maritima*38649/	Translation/	SRX001383.11076/	Cleavage/
	dnaJ protein ERDJ3A [*B. vulgaris* subsp. *vulgaris*]	.:.:::::::.:: :::::.:.:	Rhodanese-like domain-containing protein 14, chloroplastic [*V. vinifera*]	.:::::: : :::::::::::
	*S.maritima*27747/	Cleavage/	SRX001383.145227/	Cleavage/
	Growth-regulating factor 2-like [*B. vulgaris* subsp. *vulgaris*]	:::::::::::: :::::::	Hypothetical protein PHAVU_009G048400g [*P. vulgaris*]	.:::::: : :.:::::::::
	*S.maritima*36182/	Translation/	SRX001383.205893/	Cleavage/
	CRS2-associated factor 2, chloroplastic [*B. vulgaris* subsp. *vulgaris*]	:: ::::::::: :::::::	ABC transporter F family member 5 [*M. notabilis*]	::::: :: :.::.:::::::
Conserved miRNAs with precursors identified only in *S. maritima*				
sma-miR169a^b^	*S.maritima*467708/	Cleavage/		
	Oxysterol-binding protein-related protein 4C-like [*B. vulgaris* subsp. *vulgaris*]	:::::::::::::::::::::		
sma-miR164a^b^	*S.maritima*27444/	Cleavage/		
	Uncharacterized proteinLOC104894828 [*B. vulgaris* subsp. *vulgaris*])	:::: :::.:::::::::.::		
	*S.maritima*37419/	Translation/		
	Lipid phosphate phosphatase delta [*B. vulgaris* subsp. *vulgaris*]	: :.:::::: ::::::.::		
sma-miR157a^b^	*S.maritima*347871/	Cleavage/		
	Squamosa promoter-binding-like protein 3 [*P. euphratica*]	::::::::::::::::::::		
	*S.maritima*95321/	Cleavage/		
	Hypothetical proteinPRUPE_ppa003644mg *[P. persica]*	::::::::: ::::::::::		
	*S.maritima*700613/	Cleavage/		
	Putative F-box/kelch-repeat proteinAt4g12810 [*B. vulgaris* subsp. *vulgaris*]	::::::::.:.::.: ::::		
	*S.maritima*34755/	Cleavage/		
	Ubiquitin-fold modifier-conjugating enzyme 1 [*B. vulgaris* subsp. *vulgaris*]	::.:::.::::: ::::..:		
	*S.maritima*15309/	Cleavage/		
	Putative G3BP-like protein isoform *X*2 [*B. vulgaris* subsp. *vulgaris*]	::::: :: :.::.::::::		

The targets were identified in *S. maritima*, and also in *Rhizophora mangle* and *Heritiera littoralis*, the mangrove plants as the ESTs of these plants were used initially for identification of the novel miRNAs in *S. maritima*. SRX001383 and SRX001410 are the accession numbers of *R. mangle* and *H. littolalis*, respectivey in NCBI database

^a^For sequence please see Additional file 10

^b^Precursor not identified in the mangrove plants *R. mangle and H. littoralis*

**Fig. 7:**
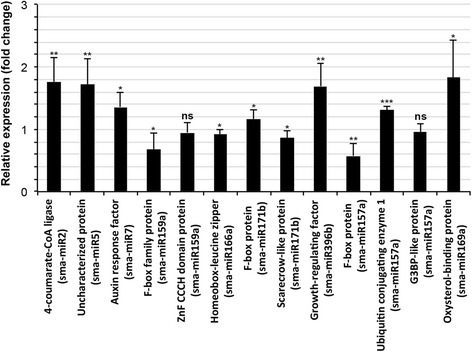
Fold changes in the expression of the *S. maritima* transcripts targeted by the miRNAs identified in the plant after exposure to 340 mM NaCl for 9 h. The data are the mean ± SD of six independent estimations. The values less than one represent decreases, and values more than one represent increases in the expression/abundance of the target mRNAs in the NaCl-treated plants relative to the non-treated (control) plants. The details regarding the statistical significance are as in Fig. 5

Changes in the expression of most of the target mRNAs of miRNA that presented precursors in *S. maritima* were verified by RT-qPCR in the plants grown in the presence and absence of 340 mM NaCl. The expression of the targets of the conserved miRNAs in responses to NaCl exhibited varied results so far as expression of these miRNAs are concerned. Most of the targets, such as the F-box protein and zinc finger CCCH domain proteins, G3BP and F-box family protein member, homeobox-leucine zipper protein and scarecrow transcription factor protein, were downregulated in response to the NaCl application (Fig. 7) in agreement of the NaCl-induced upregulation of the miRNAs sma-miR159a, sma-miR157a, sma-miR166a and sma-miR171b targeting them, respectively (Fig. 3). However, despite NaCl-induced upregulation of the miRNAs sma-miR171b, sma-miR396b, sma-miR157a and sma-miR169a (Fig. 3), several of their targets were also upregulated, such as F-box protein, GRF protein, ubiquitin conjugating enzyme and oxysterol-binding protein, respectively (Fig. 7), although these were predicted to be cleaved by their respective miRNAs (Table 3). The putative targets of all of the novel tested miRNAs (sma-miR2, sma-miR5 and sma-miR7) were upregulated (Fig. 7), i.e., opposite to and in agreement with the changes in expression for the miRNAs (Fig. 5) in response to NaCl application.

## Discussion

The identification of 134 conserved miRNAs (Fig. 1, Additional file 4) from nearly 17 million total reads (Additional file 3) in the current study is comparable to the identification of 115 conserved miRNAs from nearly 15 million reads in *Cunninghamia lanceolata* [47] and 210 conserved miRNAs from nearly 13 million total reads in *S. europaea* [27]. Most of the miRNAs identified belonged to the glycophytes such as *A. lyrata*, *A. thaliana*, *G. max*, *O. sativa*, *P. trichocarpa*, *Z. mays* and additional species for which the miRNA database is relatively large compared to the other plant species, suggesting the need to create miRNA and EST databases containing as many diverse groups of plant species as possible. The family diversity of miRNAs (Fig. 1) was similar to those reported in the halophytes such as *Avicennia marina* (193 conserved miRNAs distributed to 36 families) and *S. europaea* (210 conserved miRNAs distributed to 51 families), with miRNA to miRNA family ratios of ~5.3 and ~4.1, respectively [27, 30]. In contrast, studies have shown much lower miRNA to miRNA family ratios in a range from 1.5 to 2.0 [52–56] for terrestrial plants other than halophytes. NaCl-induced changes observed in the miRNA abundance in several miRNA families, particularly miR172, miR408, miR399, miR166, miR165 and miR535 (Fig. 1), is indicative of an altered metabolism in the test plant in the presence of salt, which was also observed in other studies, including halophytes and non-halophytes [27, 57].

The expression of several of the miRNAs such as miR166b, miR166e, and miR166m that exhibited salt-induced decreases in abundance in *S. maritima* (Fig. 2a) revealed increases in abundance in rice in response to salt treatment [58]. The reverse was true for other miRNAs, such as miR156a, miR159a, miR159c, miR168a, miR169a, miR169b, miR171b, miR172c, miR319a and miR398a (Fig. 2a, b) [58]. However, the expression levels of at least nine out of the 12 miRNAs found common in *S. maritima* and *S. europaea* [27] were comparable, with five (miR166a, miR164a, miR165a, miR166e, miR165a) showing downregulation and four (miR168b, miR169a, miR171b, miR396b) showing upregulation in both of the species. These observations suggested that the response of miRNAs to environmental change might differ with the plant species and that the level of difference would depend on the difference in their metabolism.

None of the novel miRNAs identified in *S. maritima* was identical to those identified in the halophytes *S. europaea* [27], *S. brachiata* [31] and *A. marina* [30], suggesting that the database of plant miRNAs is not yet saturated and that there is a need for further miRNA identification in more halophytes. However, the unavailability of genome/transcriptome information often limits such attempts. To overcome this disadvantage, Khraiwesh et al. [30] used the Mangrove Transcriptome Database (MTDB - http://mangrove.illinois.edu), whereas Singh and Jha [31] relied on the genome databases of species other than *S. brachiata* at NCBI. In fact, identification of the precursors of as many as seven novel miRNAs in *S. maritima* out of the 13 novel miRNAs identified using the ESTs of *R. mangle* and *H. littoralis* that met all the criteria (Table 2, Additional files 4 and 5) revealed that the ESTs of one plant species can be used in the computational predictions of miRNAs in another species, even taxonomically separated species, if they were derived from similar habitats. However, metabolism may be more important than habitat for determining similarities in the miRNAomes of two species because while all seven miRNA precursors in *S. maritima* were also present in *R. mangle* and both species accumulated high levels of Na^+^ in their tissues [42, 59], only one of the precursors (sma-mir4) was present in *H. littoralis*, which accumulated low amounts of Na^+^ in its tissues [59].

Most of the novel miRNAs identified were low in abundance (Fig. 4) and expressed only in response to NaCl (sma-miR1, -miR3, -miR4 and -miR5), unlike the conserved miRNAs. Feng et al. [27] described the low-abundance novel miRNAs in *S. europaea* that were expressed only in the salt-treated plants as species-specific miRNAs that could be important determinants of salt tolerance in the plant.

The influence of NaCl on the expression levels of miRNAs considered for Northern analysis (Fig. 3) has been reported to vary with species [24, 32, 60], indicating a species-specific response of these miRNAs. However, because of the presence of these miRNAs in a wide taxonomic group of plant species and their responsiveness to a wide range of abiotic stresses (Table 1), their involvement in protecting plants against stress cannot be ruled out. Similarly, the salt responsiveness of the novel miRNAs, particularly of sma-miR2 and sma-miR7 showing downregulation in response to the NaCl treatment (Fig. 5), indicates that their metabolic functions are important for salt tolerance in the plant. A significant decrease in sma-miR7 expression in *S. maritima* showed no changes in ETR, whereas significant increases in the miRNA expression level in the rice cultivars showing decreases in ETR in response to NaCl (Fig. 6, Additional file 8) provide circumstantial evidence of involvement of sma-miR7 in salt tolerance.

Most of the targets of the conserved miRNAs were downregulated after NaCl treatment (Fig. 7), which is consistent with the increased expression of the miRNAs after NaCl treatment, except for that of sma-miR396b (Fig. 3). The decrease in the expression of at least a few of the targets might be important in the context of salt tolerance. For example, the decrease in the cellular level of lipid phosphate phosphatase (LPP) might lead to acceleration of the phosphorylation signaling [61], which, in turn, could function to accelerate biochemical processes linked to salt tolerance. Similarly, the decreases in the cellular levels of ubiquitin-conjugating enzyme (E2) and F-box proteins might function to slow the degradation process of the cellular proteins [62–64] associated with salt tolerance in the plant. Hence, the upregulation of these miRNAs in response to NaCl application could be of great ecological significance in terms of adaptations of *S. maritima* to NaCl.

Functional annotation of the targets of the novel miRNAs (Table 3), most of which were upregulated (Fig. 7), revealed that these targets and the associated miRNAs could be important for plant adaptations to adverse environments, a possibility that could be particularly true for sma-miR7, which was downregulated in *S. maritima* and upregulated in the rice cultivars (Fig. 6), and targeted ARF family proteins in *S. maritima*, *R. mangle* and *H. littoralis* (Table 3). Although the reports of involvement of auxins in salt resistance in plants are contradictory [65], their involvement is supported by the greater expression of sma-miR7 in the salt-sensitive Badami compared with that of the moderately salt tolerant Pokkali in response to NaCl application, along with the salt-induced downregulation of sma-miR2 targeting SEUSS-LIKE (SLK) protein in *R. mangle*, which is required for a proper response to the phytohormone auxin [66]. Moreover, the downregulation of seu-miR160 and seu-miR5 targeting ARF genes in *S. europaea* in response to NaCl application [27] further supports a possible role for auxin in salt tolerance in plants.

The possible involvement of sma-miR7 in salt resistance is further emphasized by the occurrence of another common target of the miRNA in *S. maritima* and *R. mangle*/*H. littoralis*, the vacuolar protein sorting-associated protein (Table 3), a constituent of ESCRTs (Endosomal Sorting Complexes Required for Transport), which are involved in mono-ubiquitination mediated endocytic trafficking of plasma membrane proteins to the vacuole followed by lysosomal degradation [67]. Moreover, this miRNA also targets the RING finger protein E3 ligase (Table 3), which serves as a unit of the ubiquitin/26S proteasome system. The biological role of the ubiquitin/26S proteasome system has been investigated in relation to the abiotic stress tolerance of plants and has been found to be a positive regulator of stress tolerance [63]. The suppression of the expression of the miRNA (seu-miR396h/I) that targets ubiquitin-specific proteases (UBPs) in *S. europaea* also strongly suggests a possible role for the ubiquitin/26S proteasome system in salt tolerance in plants [27]. Nevertheless, the activity of both plasma membrane proteins and cytosolic proteins, might also be influenced by regulating the abundance of active protein, which is modulated by the phosphorylation process as indicated by the downregulation of sma-miR5 in response to NaCl application (Fig. 5) and the presence of the target serine/threonine kinase family protein in *S. maritima* and both mangrove plants (Table 3). The importance of protein phosphorylation in salt tolerance has also been indicated by Feng et al. [27] in *S. europaea*.

The salt tolerance process in halophytes is mediated by many proteins in various ways [3, 27, 68]. Feng et al. [27] stressed the important roles of the proteins such as squamosa promoter-binding-like protein (SPL) and nuclear factor Y subunit A (NF-YA), among others, in the morphological adaptation of the halophyte *S. europaea* to salt stress, which also seems to hold true for *S. maritima*, because the miRNAs (sma-miR156b, -157d and -169g) targeting these transcripts (Additional file 11) were downregulated in response to NaCl (Fig. 2). Moreover, salt application downregulated the expression of miR-169g targeting NF-YA in the halophyte *S. brachiata* [31]. Feng et al. [27] further related salt tolerance in *S. europaea* to the increase in protein turnover that occurs as a result of the miRNA-regulated upregulation of the F-box protein. Salt application has also been reported to increase F-box protein transcripts in *S. maritima* and *S. fruticosa* [3, 68], and the increase in *S. maritima* could have been caused by the downregulation of certain miRNAs, such as sma-miR157d and sma-miR156b (Fig. 2), which target the mRNAs encoding F-box proteins (Additional file 11). These results suggest the important role of the ubiquitin/26S proteasome system in maintaining the required protein turnover demanded by the metabolic requirements in the presence of salt [27]. In addition, Sahu and Shaw et al. [3] indicated accumulation of glycinebetaine as an important component of salt tolerance in *S. maritima*, and the salt-induced downregulation of sma-miR169g (Fig. 2) supports the same because the miRNA targets phosphoethanolamine N-methyltransferase (Additional file 11), which is involved in glycinebetaine biosynthesis. Nevertheless, the overall salt tolerance process in halophytes could be under the control of regulatory kinases [27, 68], a possibility that is also supported by the current study in which NaCl induced the downregulation of sma-miR157d and -miR5 (Figs. 2 and 5), which target serine-threonine kinase (Additional file 11, Table 3).

Although the regulatory role of miRNAs cannot be generalized, the expression of sma-miR2 and sma-miR5 only in plants growing in saline environments, such as *S. maritima*, *S. portulacastrum*, *C. arenarius* and *I. pes-caprae* (Additional file 9), does suggest that these miRNAs might be regulating certain biochemical processes common in the plants that are important for their survival in the given saline ecological conditions. In this regard, it is important to note that sma-miR2 targets 4-coumarate-CoA ligase in *S. maritima* (Table 3), an enzyme that plays a pivotal role in the biosynthesis of plant secondary compounds with essential functions in plant development and environmental interactions [69].

## Conclusion

This study identified 134 conserved and thirteen novel miRNAs in *S. maritima*, a halophyte. More importantly, the study demonstrated that the transcriptome of one species can be successfully used to computationally predict miRNAs in other species inhabiting similar environments, even if they are taxonomically distantly related. However, metabolism could be an even more important determining factor of similarity in miRNAomes of two species than the habitat. This study further revealed that a high ratio of known or conserved miRNAs to the number of families to which they belong could be considered a potential indicator of further miRNA identifications in a species, a result that is supported by the lack of identities among the novel miRNAs identified in the three halophytes, including *S. brachiata* [31], *S. europaea* [27] and *S. maritima* (this study). Furthermore, the responsiveness of many of the conserved miRNAs and most of the novel miRNAs in particular to salt suggested their important roles, in the metabolic adjustments to facilitate growth in a saline environment. However, any role for miRNAs in salt tolerance in halophytes may not be straightforward because several metabolic pathways could be acting either in parallel or in sequence during the process [3, 27, 68]. Of course, the miRNAs specifically expressed in plants growing under the influence of seawater, such as sma-miR2 and sma-miR5, could be important for the plants’ survival in a saline environment. Although miRNA-mediated molecular mechanisms for salt tolerance in plants have yet to be established, the target prediction of novel miRNAs provided an indication that ubiquitin-mediated alterations in the abundance of plasma membrane and cytosolic proteins and phosphorylation-mediated alterations of their active states could be important miRNA-regulated biochemical processes for salt tolerance in halophytes.

## Additional files

Additional file 1:
**Taxonomy of the plant species used in the study.** (DOCX 17 kb) Additional file 2:
**Probes and primer sequences.** (XLSX 262 kb) Additional file 3:
**Summary of sRNA reads.** (DOCX 17 kb) Additional file 4:
**Pooled reads of the conserved miRNAs identified from the sRNA reads generated by sequencing of the sRNAs libraries and details of the precursors of the novel and conserved miRNAs validated experimentally for their presence in** ***S. maritima.*** (XLSX 42 kb) Additional file 5:
**Stem-loop secondary structure of precursors of the novel and conserved miRNAs validated experimentally for their presence in** ***S. maritima.*** (PPT 4355 kb) Additional file 6:
**Details of the precursors of the conserved miRNAs present in** ***S. maritima*** **and/or** ***R. mangle.*** (DOCX 19 kb) Additional file 7:
**Progress of the Stem-Loop PCR for the novel miRNAs in control and NaCl treated** ***S. maritima*** **and the rice cultivars, and in** ***S. portulacastrum*** **collected from its natural saline habitat.** (PPTX 4 mb) Additional file 8:
**Changes in the relative electron transport rate (ETR) in the leaves of the test plants determined after their exposure to 255 mM NaCl (** ***O. sativa*** **cv. Badami/Pokkali) or 340 mM NaCl (** ***S. maritima*** **) for 9 h, 24 h and 96 h.** (TIFF 8 mb) Additional file 9:
**Study of the expression of sma-miR2 and sma-miR5 in** ***S. maritima,*** ***S. portulacastrum,*** ***C. arenarius*** **and** ***I. pes-caprae,*** **which were collected from their natural ecological environments.** (TIFF 20 kb) Additional file 10:
**The target ESTs of the conserved miRNAs represented in Fig.** 2 **.** (DOCX 242 kb) Additional file 11:
**Targets of the conserved miRNAs mentioned in Fig.** 2 **.** (DOC 283 kb)
