# Supplementary material for: Novel and conserved miRNAs in the halophyte Suaeda maritima identified by deep sequencing and computational predictions using the ESTs of two mangrove plants
Source: BMC Plant Biol. 2015 Dec 29;15:301. doi: 10.1186/s12870-015-0682-3 (PMC4696257; doi:10.1186/s12870-015-0682-3)
Supplement: Additional file 1: — Taxonomy of the plant species used in the study. (DOCX 17 kb) [file 12870_2015_682_MOESM1_ESM.docx]

**Additional file 1**

**Novel and conserved miRNAs in the halophyte *Suaeda maritima* identified by deep sequencing and computational predictions using the ESTs of two mangrove plants**

Corresponding author E-mail: sachingharat113@gmail.com

**Taxonomy of the plant species using Tropicos database (http://www.tropicos.org/):**

1. ***Sesuvium portulacastrum:***

Class: Equisetopsida C. Agardh

Subclass: Magnoliidae Novák ex Takht.

Superorder: Caryophyllanae Takht.

Order: Caryophyllales Juss. ex Bercht. & J. Presl

Family: Aizoaceae Martinov

Genus: *Sesuvium* L.

1. ***Cyperus arenarius*:**

Class: Equisetopsida C. Agardh

Subclass: Magnoliidae Novák ex Takht.

Superorder: Lilianae Takht.

Order: Poales Small

Family: Cyperaceae Juss.

Genus: *Cyperus* L.

1. ***Ipomoea pes-caprae*:**

Class: Equisetopsida C. Agardh

Subclass: Magnoliidae Novák ex Takht.

Superorder: Asteranae Takht.

Order: Solanales Juss. ex Bercht. & J. Presl

Family: Convolvulaceae Juss.

Genus: *Ipomoea* L.

1. ***Suaeda maritima*:**

Class: Equisetopsida C. Agardh

Subclass: Magnoliidae Novák ex Takht.

Superorder: Caryophyllanae Takht.

Order: Caryophyllales Juss. ex Bercht. & J. Presl

Family: Amaranthaceae Juss.

Genus: *Suaeda* Forssk. ex J.F. Gmel.

1. ***Oryza sativa* cv. Badami and cv. Pokkali:**

Class: Equisetopsida C. Agardh

Subclass: Magnoliidae Novák ex Takht.

Superorder: Lilianae Takht.

Order: Poales Small

Family: Poaceae Barnhart

Genus: *Oryza* L.

Species: *Oryza sativa* L.
