# Supplementary material for: Novel and conserved miRNAs in the halophyte Suaeda maritima identified by deep sequencing and computational predictions using the ESTs of two mangrove plants
Source: BMC Plant Biol. 2015 Dec 29;15:301. doi: 10.1186/s12870-015-0682-3 (PMC4696257; doi:10.1186/s12870-015-0682-3)
Supplement: Additional file 3: — Summary of sRNA reads. (DOCX 17 kb) [file 12870_2015_682_MOESM3_ESM.docx]

**Additional file 3**

**Novel and conserved miRNAs in the halophyte *Suaeda maritima* identified by deep sequencing and computational predictions using the ESTs of two mangrove plants**

Corresponding author E-mail: sachingharat113@gmail.com

**Summary of sRNA reads**. Adapter ligated libraries of sRNAs from *S. maritima* grown without NaCl (control, C) and that exposed to 340 mM NaCl (treated, T) for 9 h were prepared and sequenced on Illumina platform. The sequences were processed to get the putative miRNAs.

| **Read categories** | **sRNA reads in the library control sample** | | **sRNA reads in the library NaCl treated sample** | |
| --- | --- | --- | --- | --- |
|  | **Total reads** | **Unique reads** | **Total reads** | **Unique reads** |
| Raw reads | 17819399 | 2861588 | 16000000 | 2787243 |
| Reads after adapter removal | 14492970 | 1958683 | 12101621 | 1966579 |
| Reads after filtering by sequence length (<16nt to >30nt) | 12734769 | 1798344 | 10831030 | 1841964 |
| Reads after filtering low-complexity sequences | 12734517 | 1798133 | 10830740 | 1841724 |
| Reads after filtering invalid sequences (Low quality read) | 12671722 | 1770579 | 10768978 | 1809841 |
| Reads after filtering t/rRNA | 8981591 | 1715999 | 7923122 | 1756751 |
| Reads corresponding to putative miRNAs and/or siRNAs | 8981591 | 1715999 | 7923122 | 1756751 |
