# Supplementary material for: Novel and conserved miRNAs in the halophyte Suaeda maritima identified by deep sequencing and computational predictions using the ESTs of two mangrove plants
Source: BMC Plant Biol. 2015 Dec 29;15:301. doi: 10.1186/s12870-015-0682-3 (PMC4696257; doi:10.1186/s12870-015-0682-3)
Supplement: Additional file 5: — Stem-loop secondary structure of precursors of the novel and conserved miRNAs validated experimentally for their presence in S. maritima. (PPT 4355 kb) [file 12870_2015_682_MOESM5_ESM.ppt]

## Slide 1
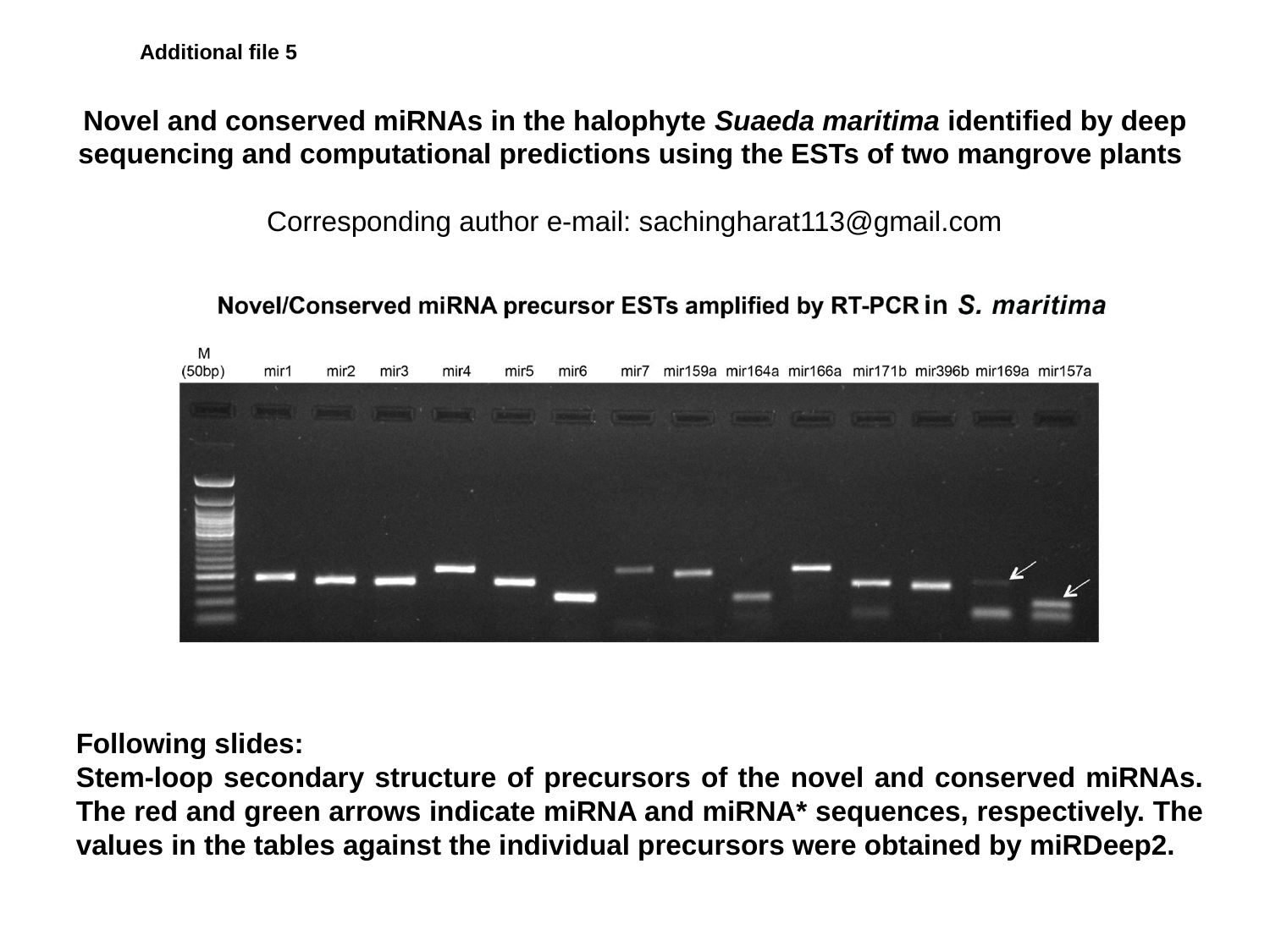

Additional file 5
# Novel and conserved miRNAs in the halophyte Suaeda maritima identified by deep sequencing and computational predictions using the ESTs of two mangrove plants    Corresponding author e-mail: sachingharat113@gmail.com
Following slides:
Stem-loop secondary structure of precursors of the novel and conserved miRNAs. The red and green arrows indicate miRNA and miRNA* sequences, respectively. The values in the tables against the individual precursors were obtained by miRDeep2.

## Slide 2
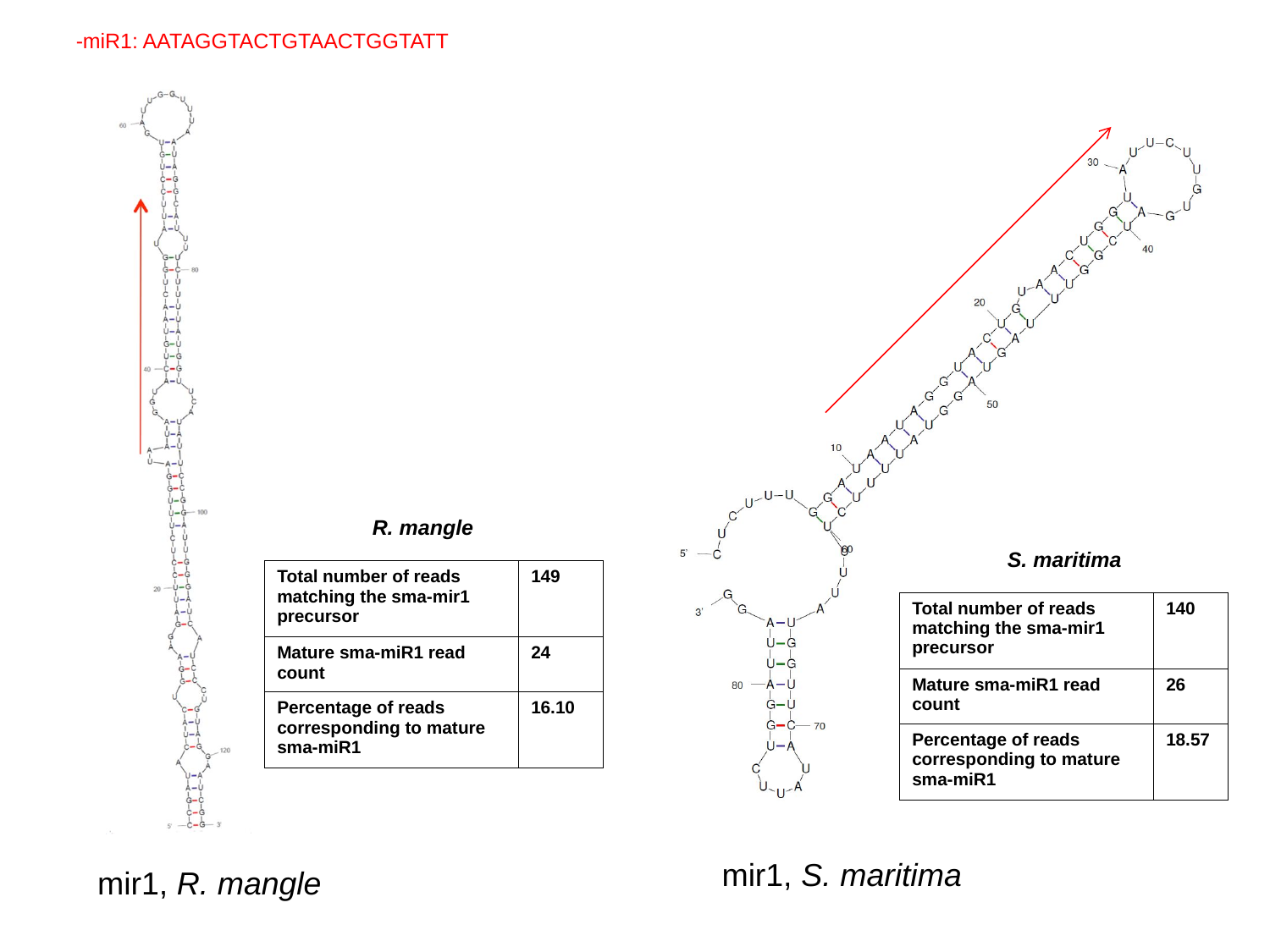

-miR1: AATAGGTACTGTAACTGGTATT
R. mangle
S. maritima
| Total number of reads matching the sma-mir1 precursor | 149 |
| --- | --- |
| Mature sma-miR1 read count | 24 |
| Percentage of reads corresponding to mature sma-miR1 | 16.10 |
| Total number of reads matching the sma-mir1 precursor | 140 |
| --- | --- |
| Mature sma-miR1 read count | 26 |
| Percentage of reads corresponding to mature sma-miR1 | 18.57 |
mir1, S. maritima
mir1, R. mangle

## Slide 3
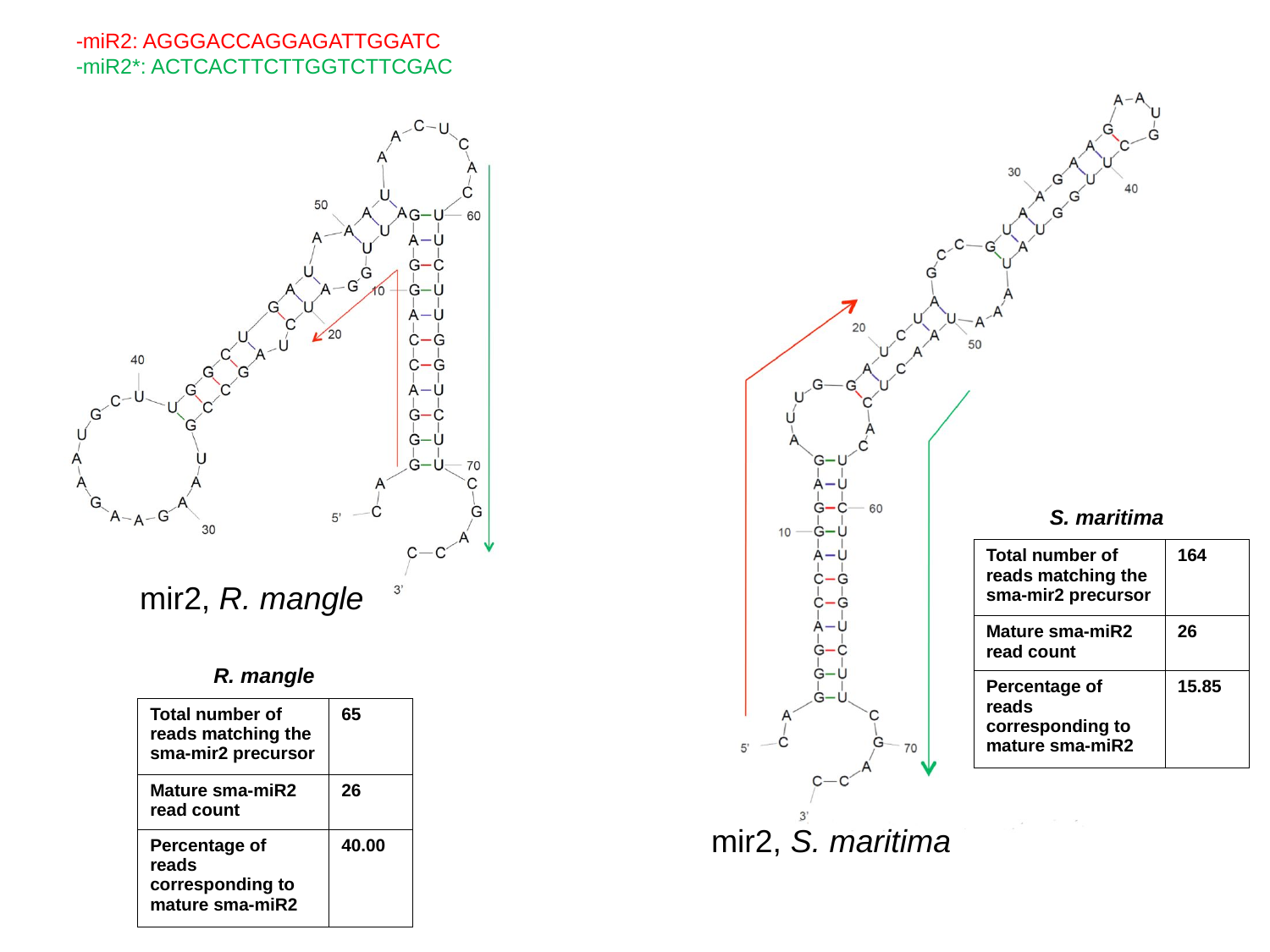

-miR2: AGGGACCAGGAGATTGGATC
-miR2*: ACTCACTTCTTGGTCTTCGAC
S. maritima
| Total number of reads matching the sma-mir2 precursor | 164 |
| --- | --- |
| Mature sma-miR2 read count | 26 |
| Percentage of reads corresponding to mature sma-miR2 | 15.85 |
mir2, R. mangle
R. mangle
| Total number of reads matching the sma-mir2 precursor | 65 |
| --- | --- |
| Mature sma-miR2 read count | 26 |
| Percentage of reads corresponding to mature sma-miR2 | 40.00 |
mir2, S. maritima

## Slide 4
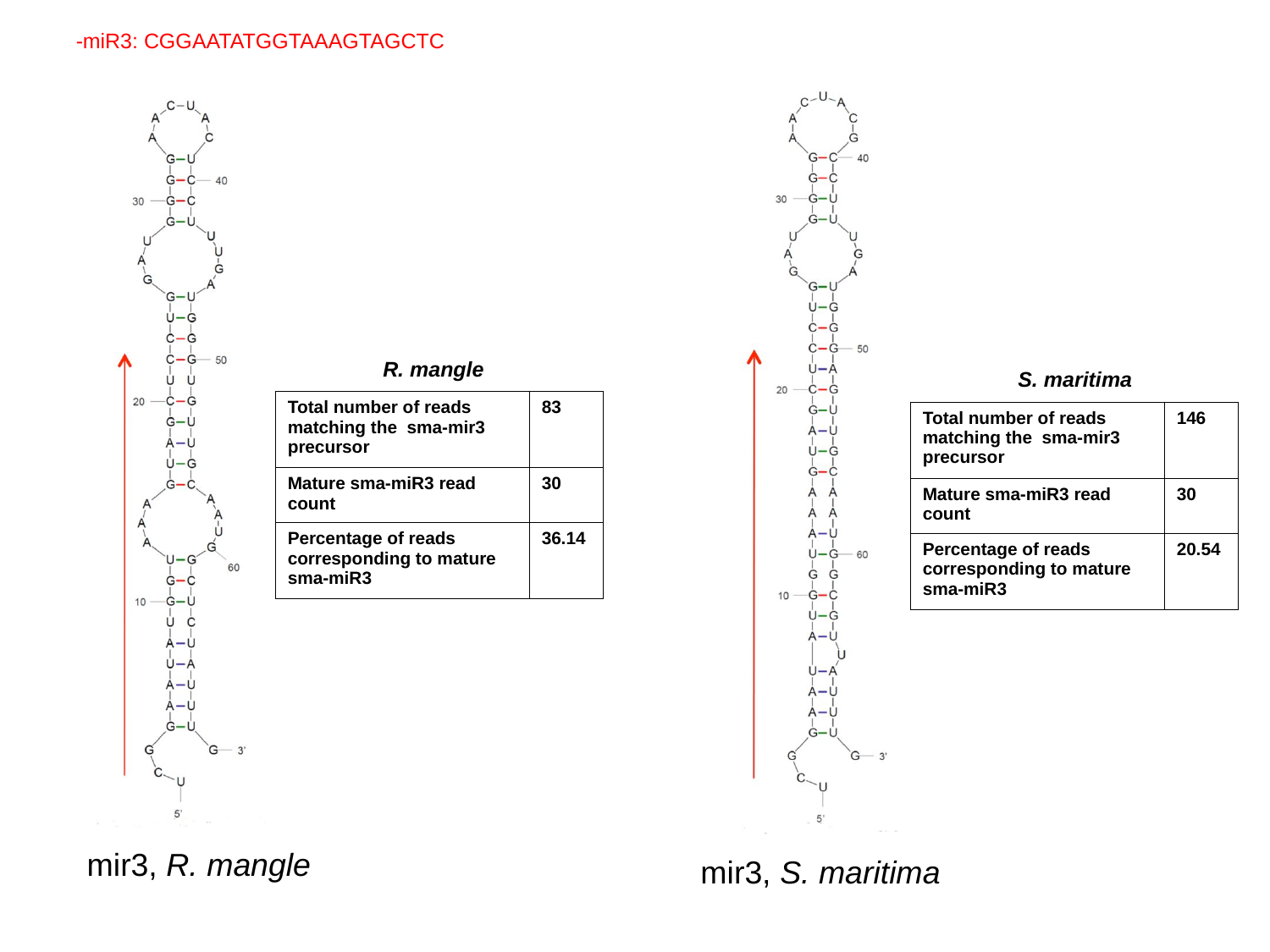

-miR3: CGGAATATGGTAAAGTAGCTC
R. mangle
S. maritima
| Total number of reads matching the sma-mir3 precursor | 83 |
| --- | --- |
| Mature sma-miR3 read count | 30 |
| Percentage of reads corresponding to mature sma-miR3 | 36.14 |
| Total number of reads matching the sma-mir3 precursor | 146 |
| --- | --- |
| Mature sma-miR3 read count | 30 |
| Percentage of reads corresponding to mature sma-miR3 | 20.54 |
mir3, R. mangle
mir3, S. maritima

## Slide 5
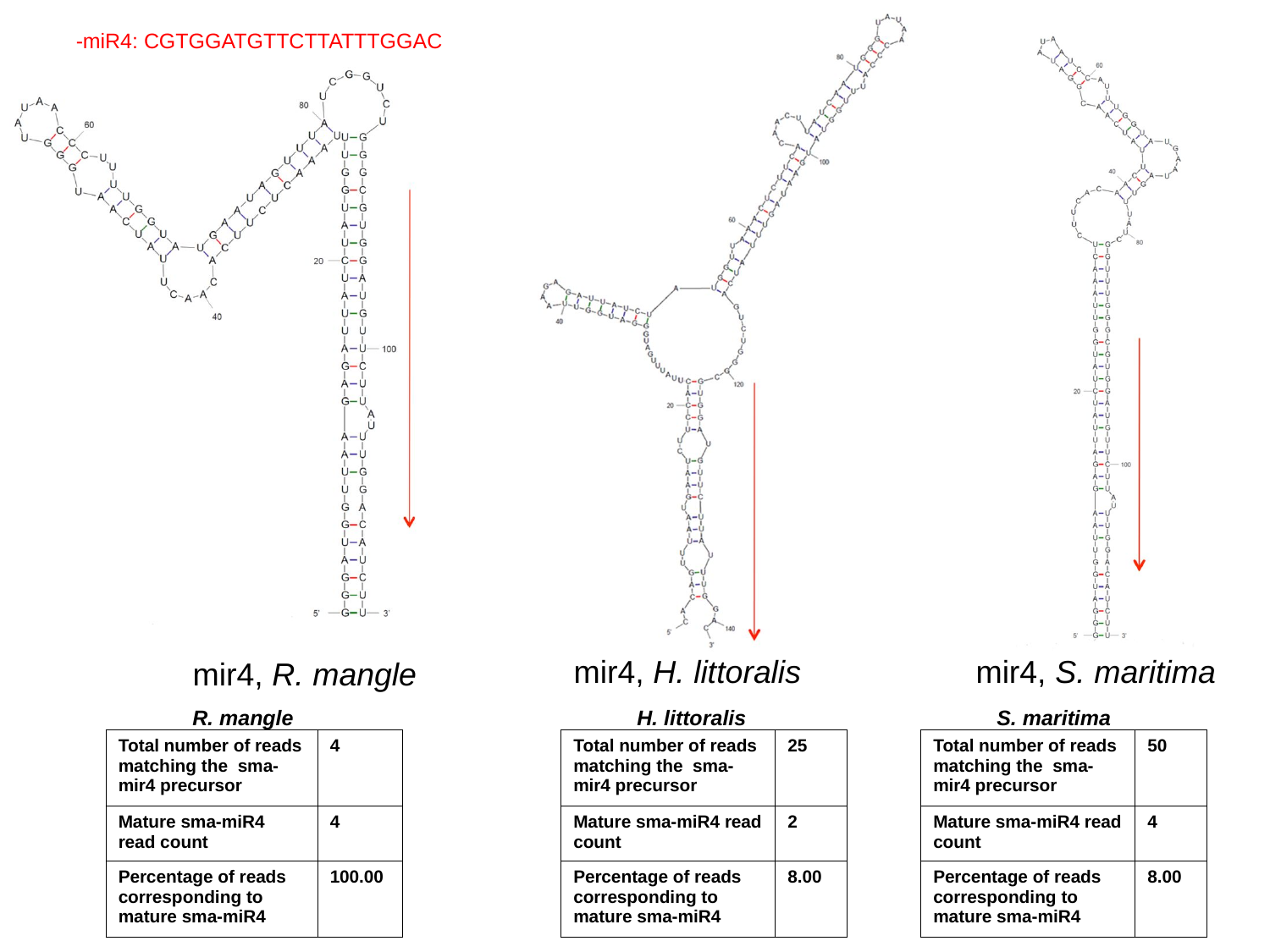

-miR4: CGTGGATGTTCTTATTTGGAC
mir4, H. littoralis
mir4, S. maritima
mir4, R. mangle
R. mangle
H. littoralis
S. maritima
| Total number of reads matching the sma-mir4 precursor | 4 |
| --- | --- |
| Mature sma-miR4 read count | 4 |
| Percentage of reads corresponding to mature sma-miR4 | 100.00 |
| Total number of reads matching the sma-mir4 precursor | 25 |
| --- | --- |
| Mature sma-miR4 read count | 2 |
| Percentage of reads corresponding to mature sma-miR4 | 8.00 |
| Total number of reads matching the sma-mir4 precursor | 50 |
| --- | --- |
| Mature sma-miR4 read count | 4 |
| Percentage of reads corresponding to mature sma-miR4 | 8.00 |

## Slide 6
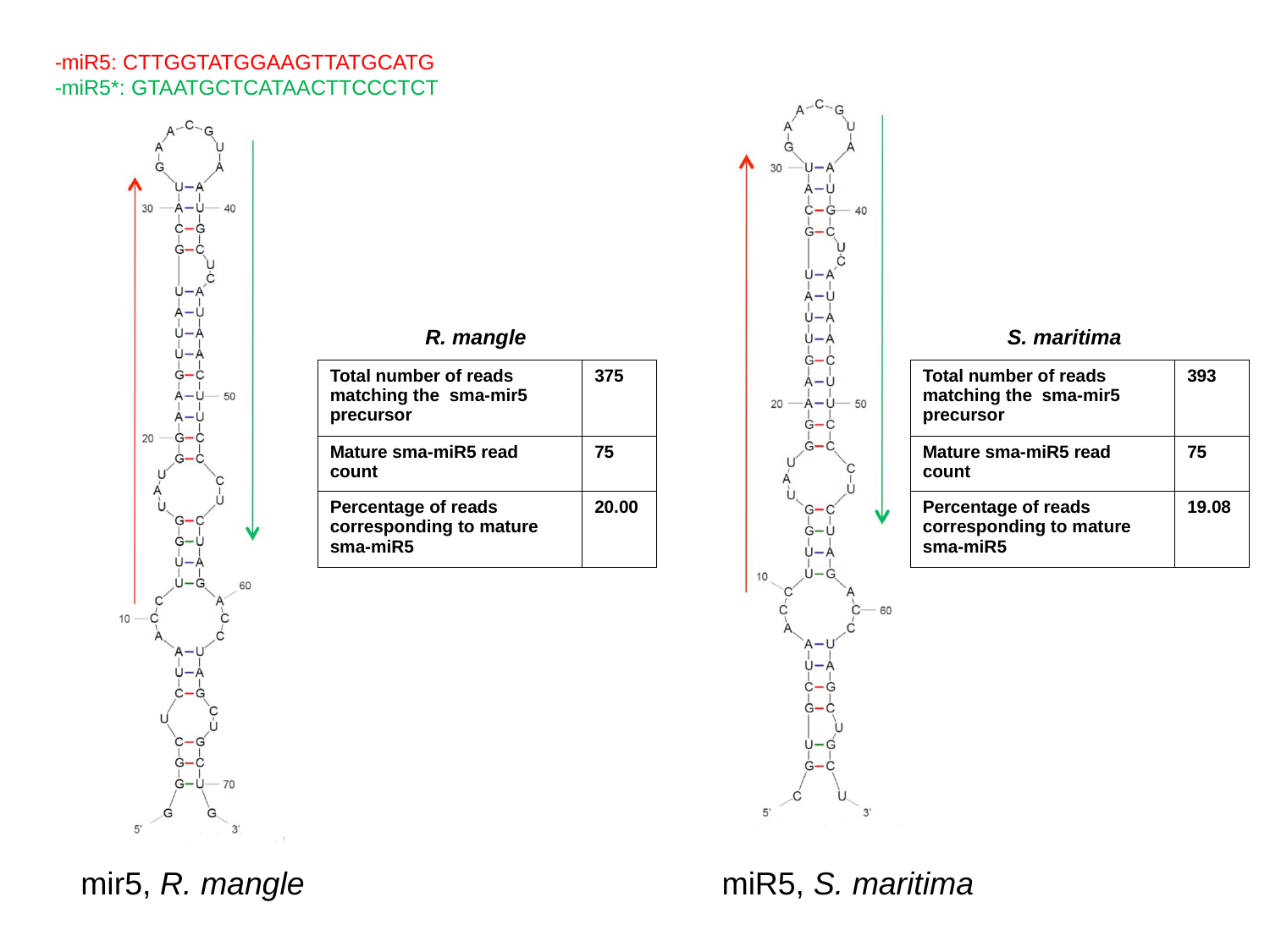

-miR5: CTTGGTATGGAAGTTATGCATG
-miR5*: GTAATGCTCATAACTTCCCTCT
R. mangle
S. maritima
| Total number of reads matching the sma-mir5 precursor | 375 |
| --- | --- |
| Mature sma-miR5 read count | 75 |
| Percentage of reads corresponding to mature sma-miR5 | 20.00 |
| Total number of reads matching the sma-mir5 precursor | 393 |
| --- | --- |
| Mature sma-miR5 read count | 75 |
| Percentage of reads corresponding to mature sma-miR5 | 19.08 |
mir5, R. mangle
miR5, S. maritima

## Slide 7
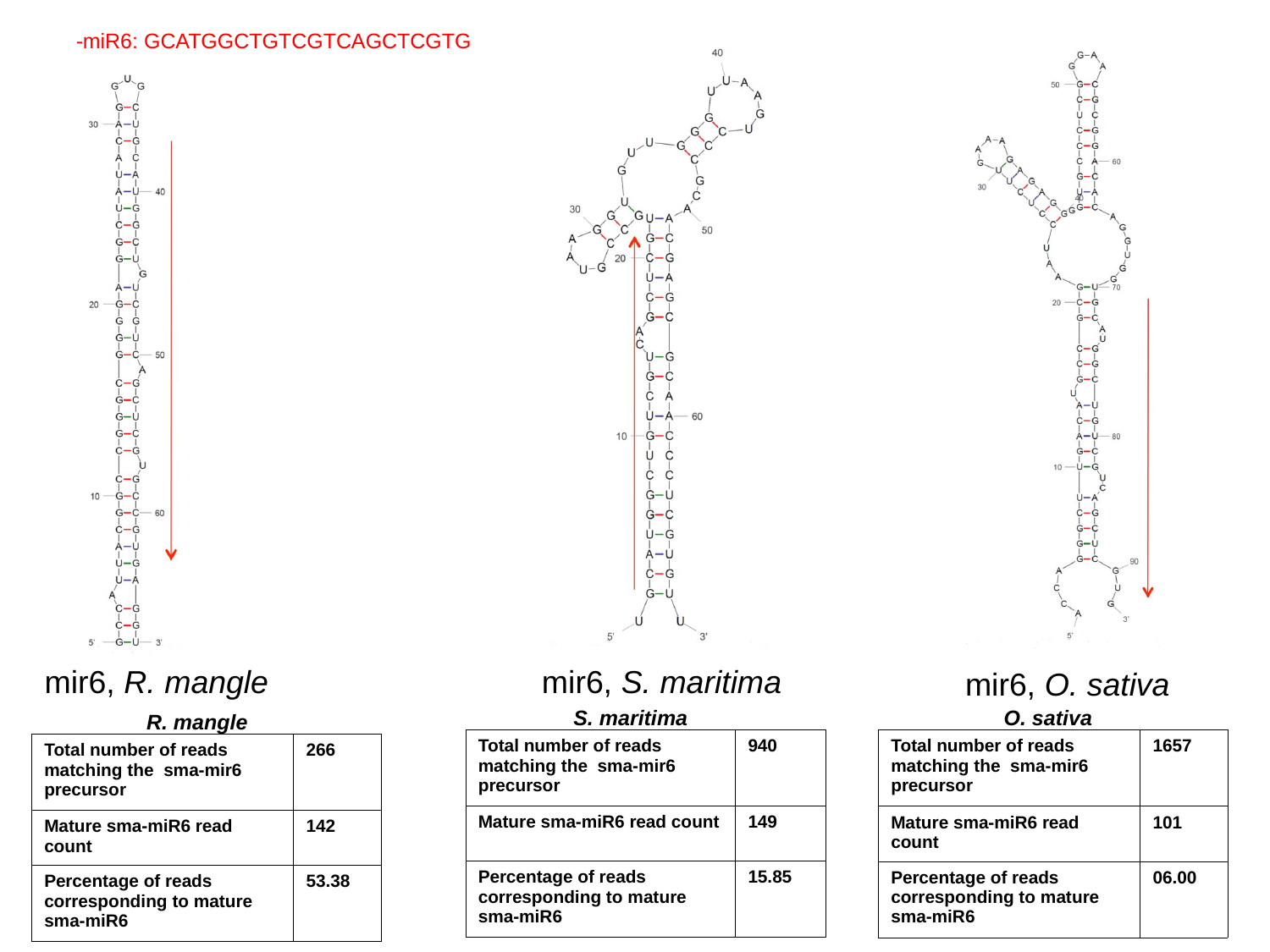

-miR6: GCATGGCTGTCGTCAGCTCGTG
mir6, R. mangle
mir6, S. maritima
mir6, O. sativa
S. maritima
O. sativa
R. mangle
| Total number of reads matching the sma-mir6 precursor | 940 |
| --- | --- |
| Mature sma-miR6 read count | 149 |
| Percentage of reads corresponding to mature sma-miR6 | 15.85 |
| Total number of reads matching the sma-mir6 precursor | 1657 |
| --- | --- |
| Mature sma-miR6 read count | 101 |
| Percentage of reads corresponding to mature sma-miR6 | 06.00 |
| Total number of reads matching the sma-mir6 precursor | 266 |
| --- | --- |
| Mature sma-miR6 read count | 142 |
| Percentage of reads corresponding to mature sma-miR6 | 53.38 |

## Slide 8
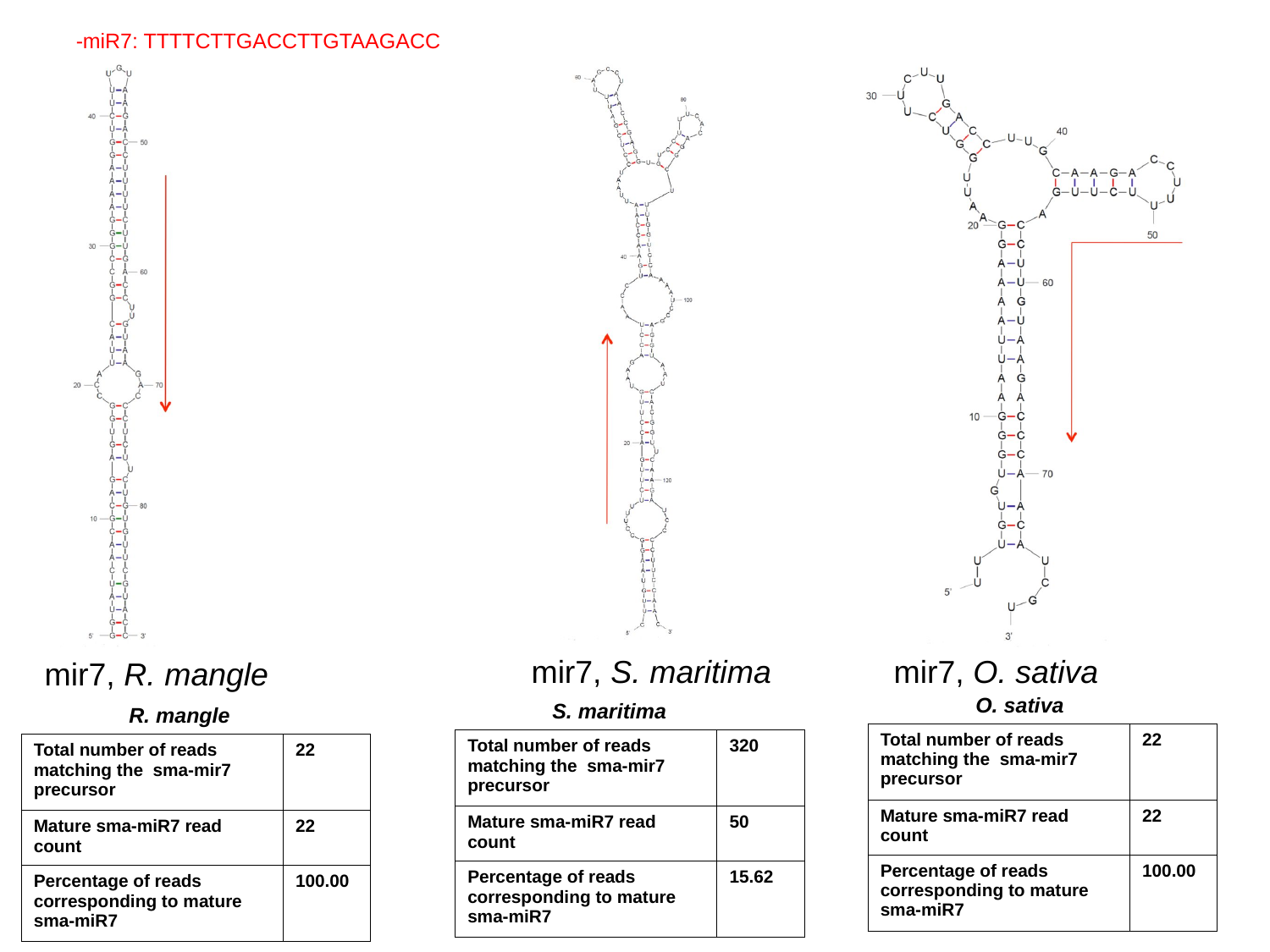

-miR7: TTTTCTTGACCTTGTAAGACC
mir7, S. maritima
mir7, O. sativa
mir7, R. mangle
O. sativa
S. maritima
R. mangle
| Total number of reads matching the sma-mir7 precursor | 22 |
| --- | --- |
| Mature sma-miR7 read count | 22 |
| Percentage of reads corresponding to mature sma-miR7 | 100.00 |
| Total number of reads matching the sma-mir7 precursor | 320 |
| --- | --- |
| Mature sma-miR7 read count | 50 |
| Percentage of reads corresponding to mature sma-miR7 | 15.62 |
| Total number of reads matching the sma-mir7 precursor | 22 |
| --- | --- |
| Mature sma-miR7 read count | 22 |
| Percentage of reads corresponding to mature sma-miR7 | 100.00 |

## Slide 9
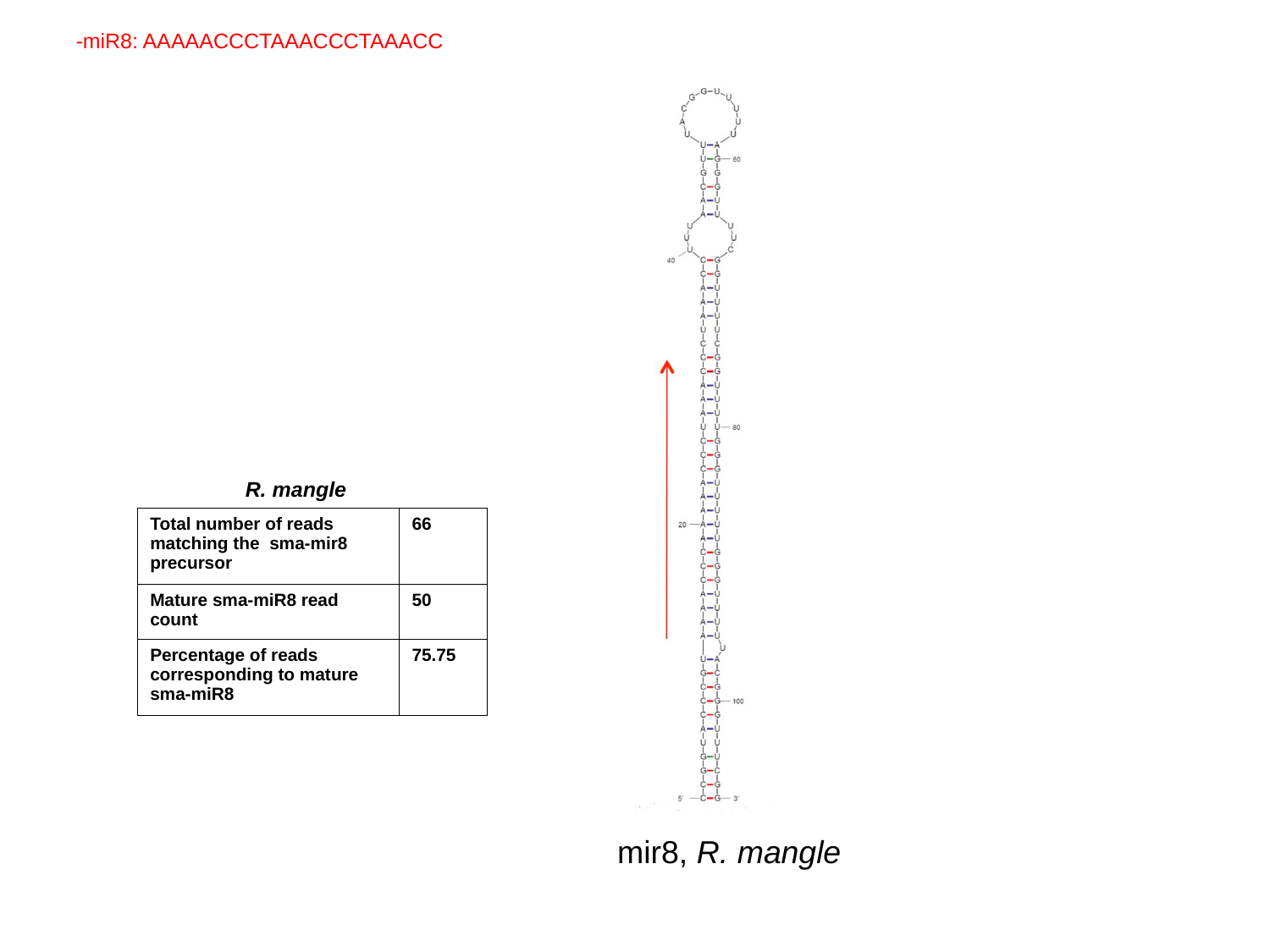

-miR8: AAAAACCCTAAACCCTAAACC
R. mangle
| Total number of reads matching the sma-mir8 precursor | 66 |
| --- | --- |
| Mature sma-miR8 read count | 50 |
| Percentage of reads corresponding to mature sma-miR8 | 75.75 |
mir8, R. mangle

## Slide 10
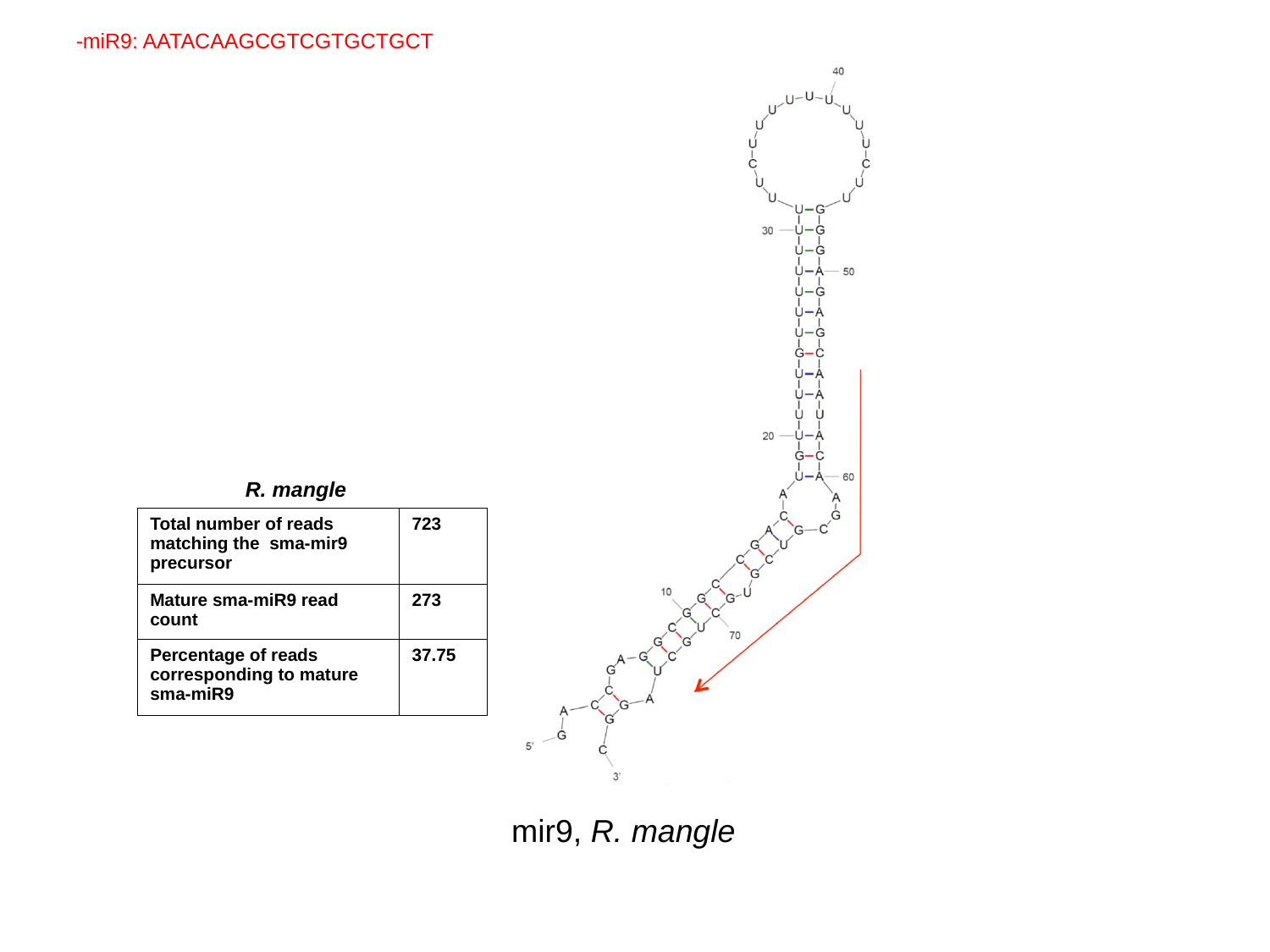

-miR9: AATACAAGCGTCGTGCTGCT
R. mangle
| Total number of reads matching the sma-mir9 precursor | 723 |
| --- | --- |
| Mature sma-miR9 read count | 273 |
| Percentage of reads corresponding to mature sma-miR9 | 37.75 |
mir9, R. mangle

## Slide 11
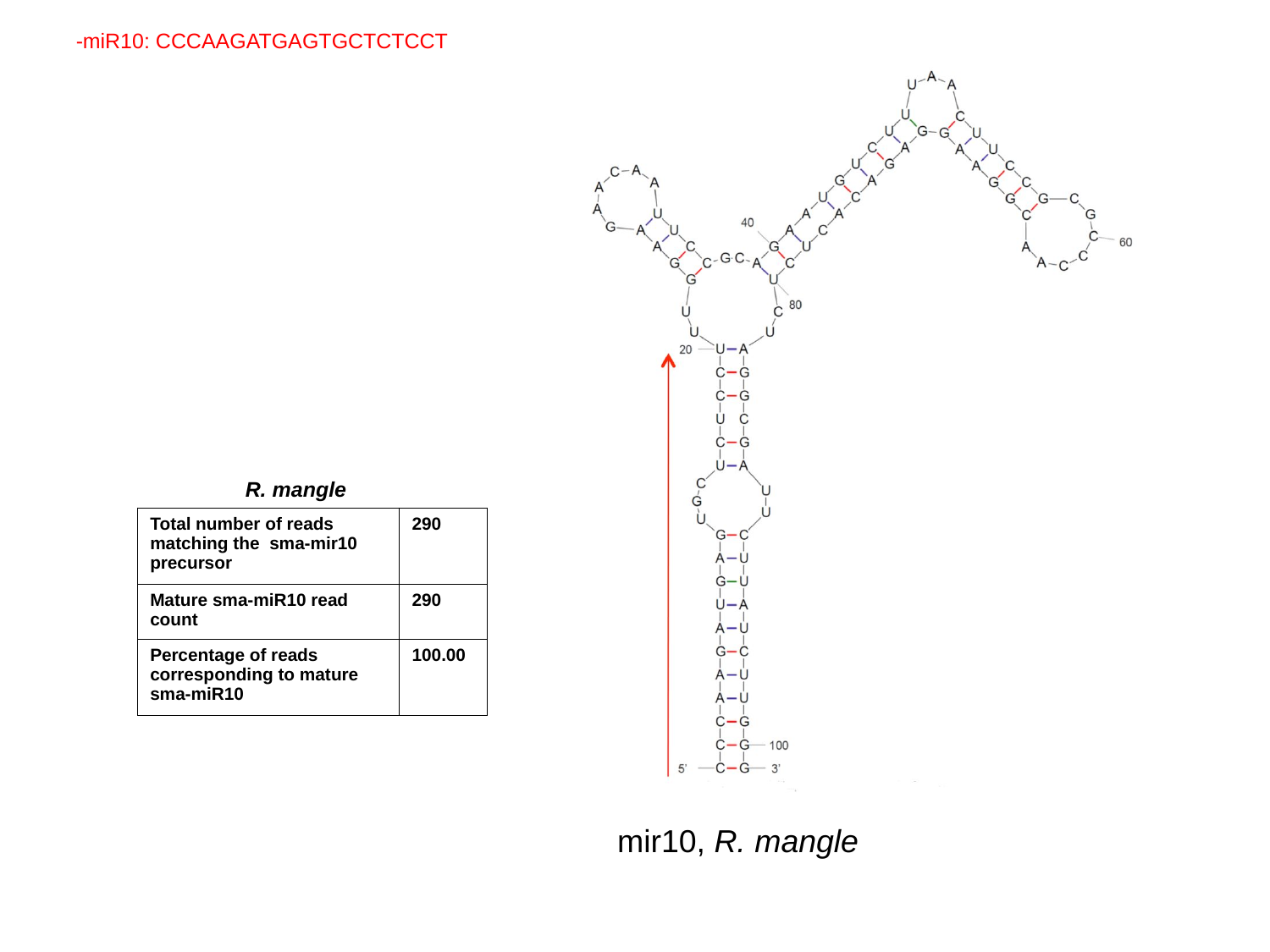

-miR10: CCCAAGATGAGTGCTCTCCT
R. mangle
| Total number of reads matching the sma-mir10 precursor | 290 |
| --- | --- |
| Mature sma-miR10 read count | 290 |
| Percentage of reads corresponding to mature sma-miR10 | 100.00 |
mir10, R. mangle

## Slide 12
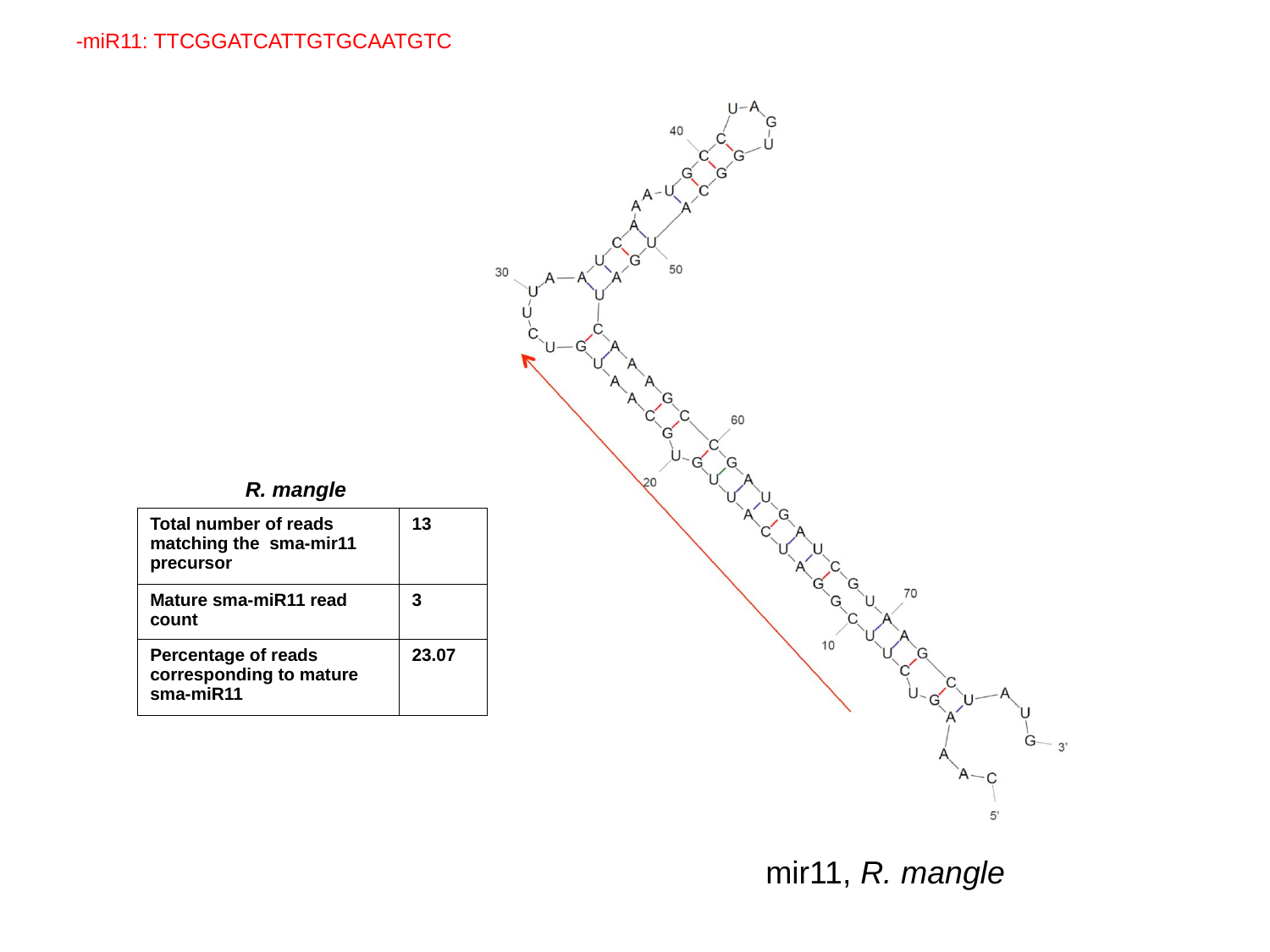

-miR11: TTCGGATCATTGTGCAATGTC
R. mangle
| Total number of reads matching the sma-mir11 precursor | 13 |
| --- | --- |
| Mature sma-miR11 read count | 3 |
| Percentage of reads corresponding to mature sma-miR11 | 23.07 |
mir11, R. mangle

## Slide 13
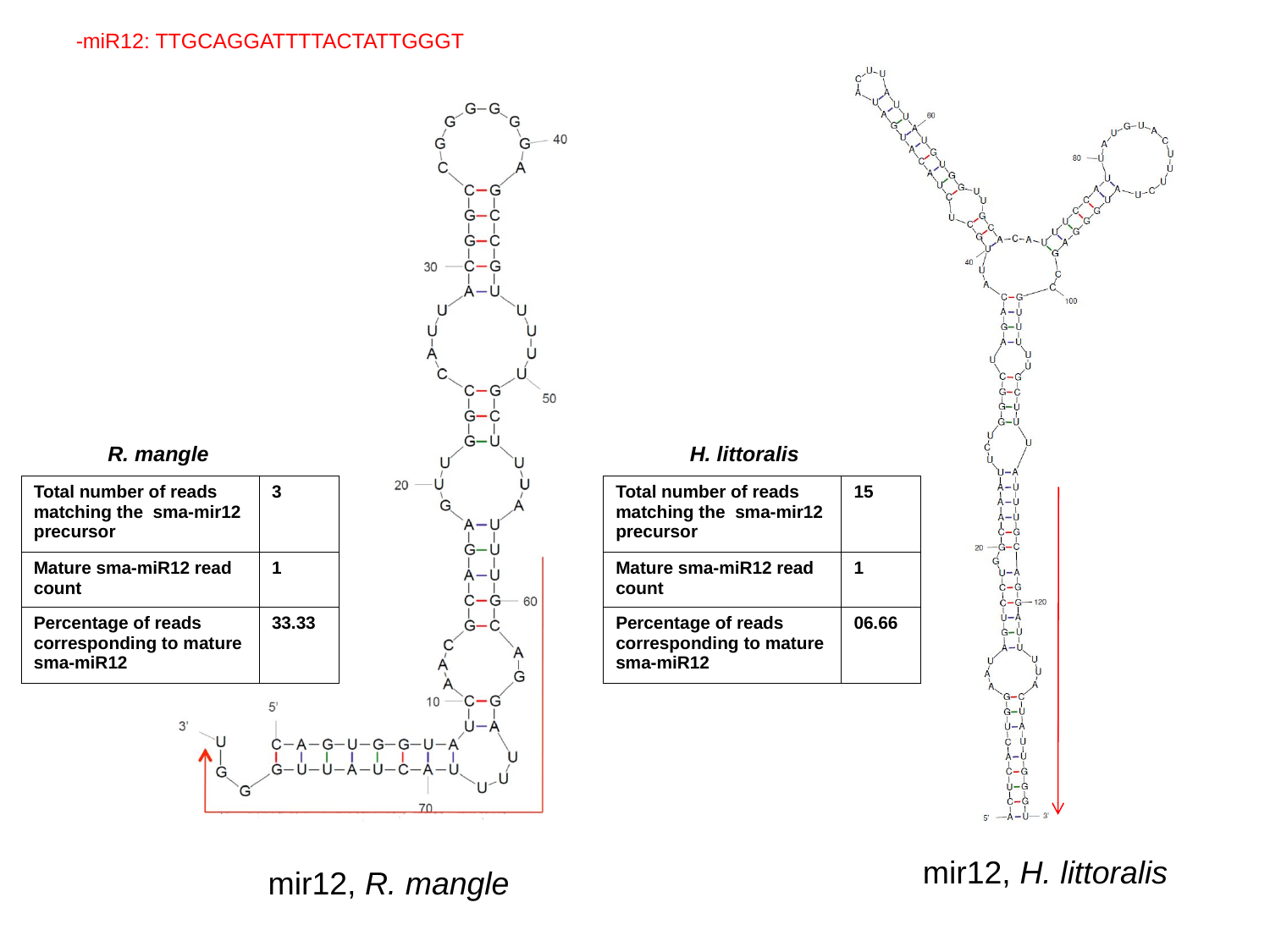

-miR12: TTGCAGGATTTTACTATTGGGT
R. mangle
H. littoralis
| Total number of reads matching the sma-mir12 precursor | 3 |
| --- | --- |
| Mature sma-miR12 read count | 1 |
| Percentage of reads corresponding to mature sma-miR12 | 33.33 |
| Total number of reads matching the sma-mir12 precursor | 15 |
| --- | --- |
| Mature sma-miR12 read count | 1 |
| Percentage of reads corresponding to mature sma-miR12 | 06.66 |
mir12, H. littoralis
mir12, R. mangle

## Slide 14
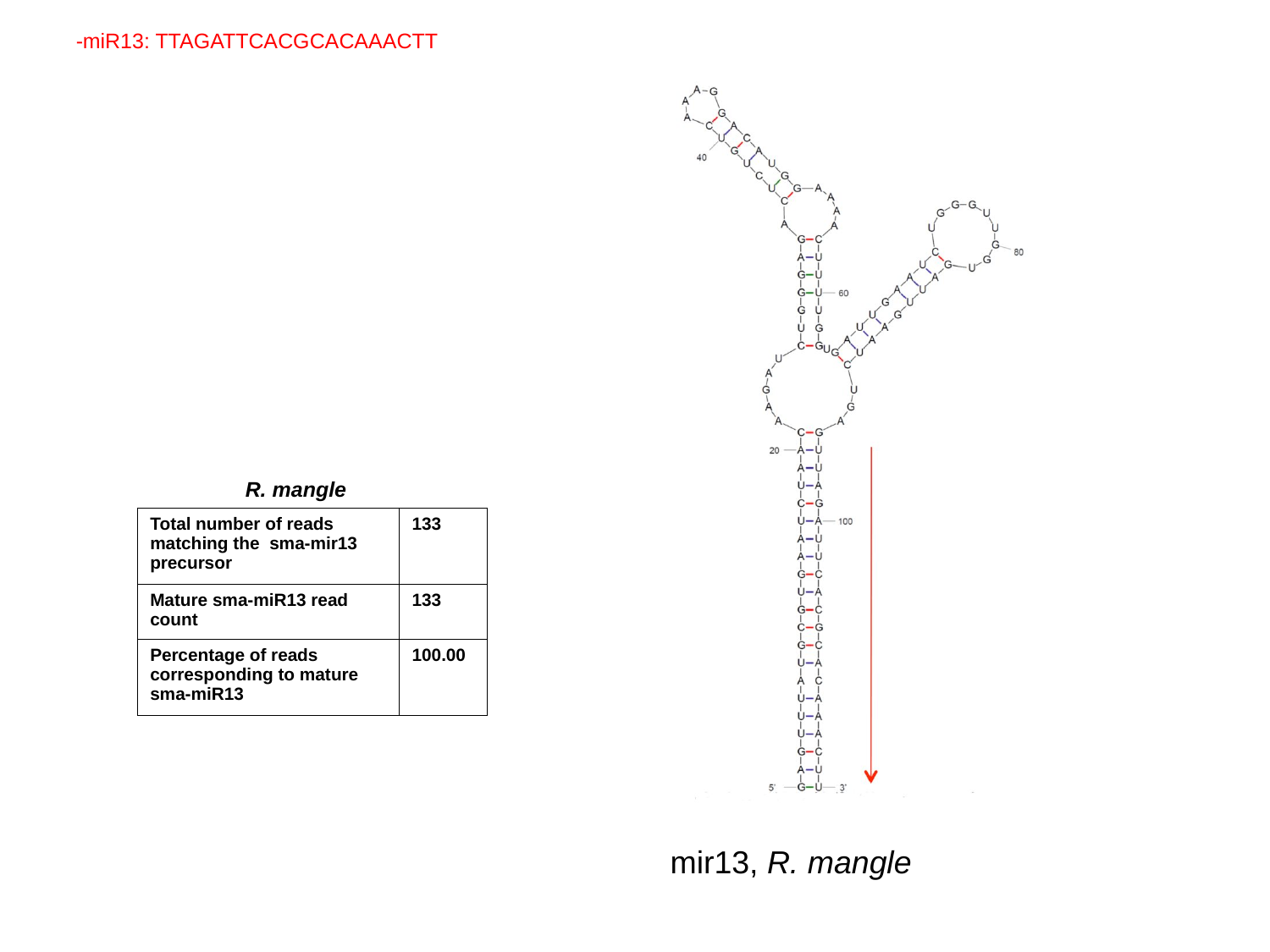

-miR13: TTAGATTCACGCACAAACTT
R. mangle
| Total number of reads matching the sma-mir13 precursor | 133 |
| --- | --- |
| Mature sma-miR13 read count | 133 |
| Percentage of reads corresponding to mature sma-miR13 | 100.00 |
mir13, R. mangle

## Slide 15
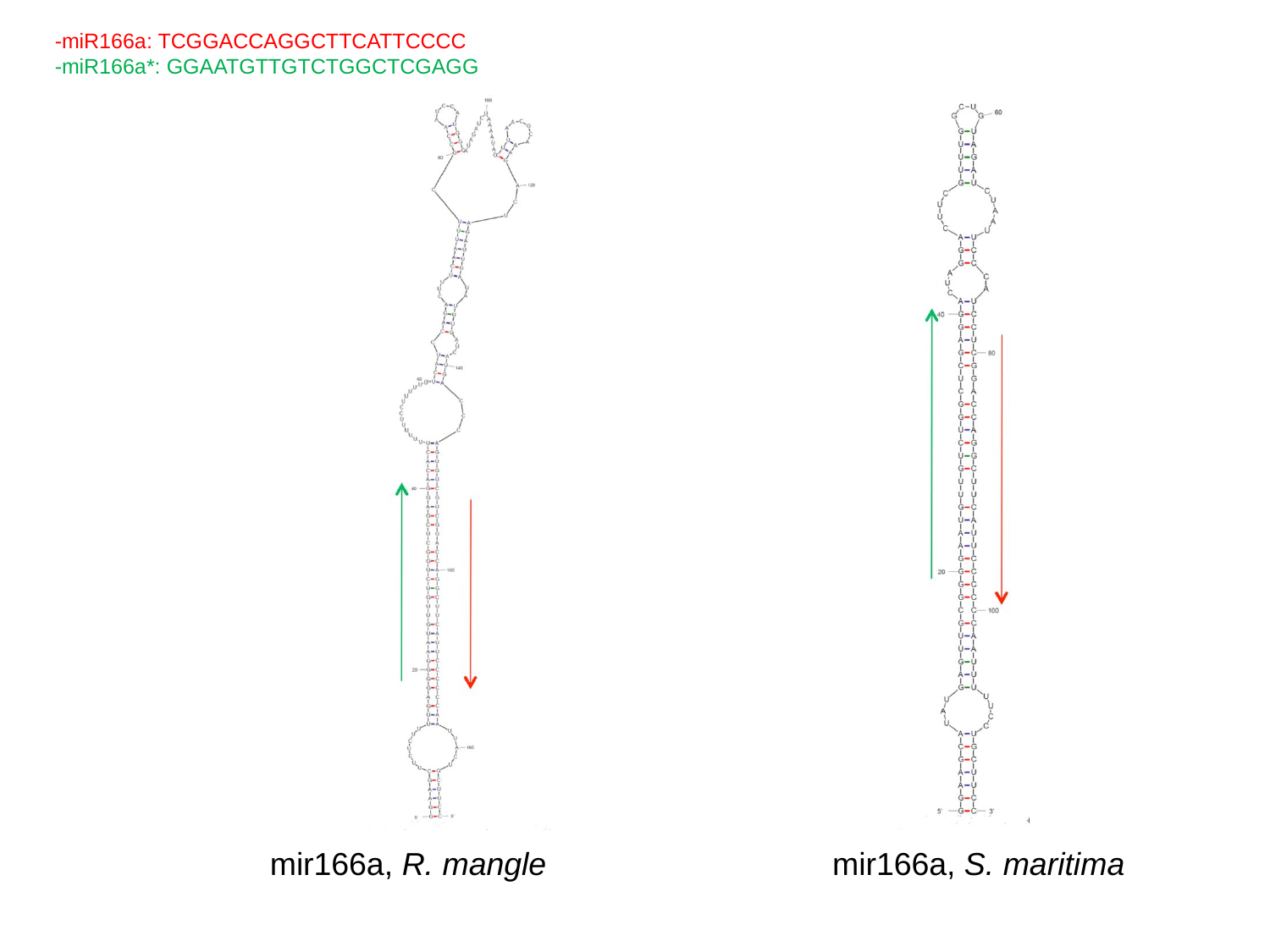

-miR166a: TCGGACCAGGCTTCATTCCCC
-miR166a*: GGAATGTTGTCTGGCTCGAGG
mir166a, R. mangle
mir166a, S. maritima

## Slide 16
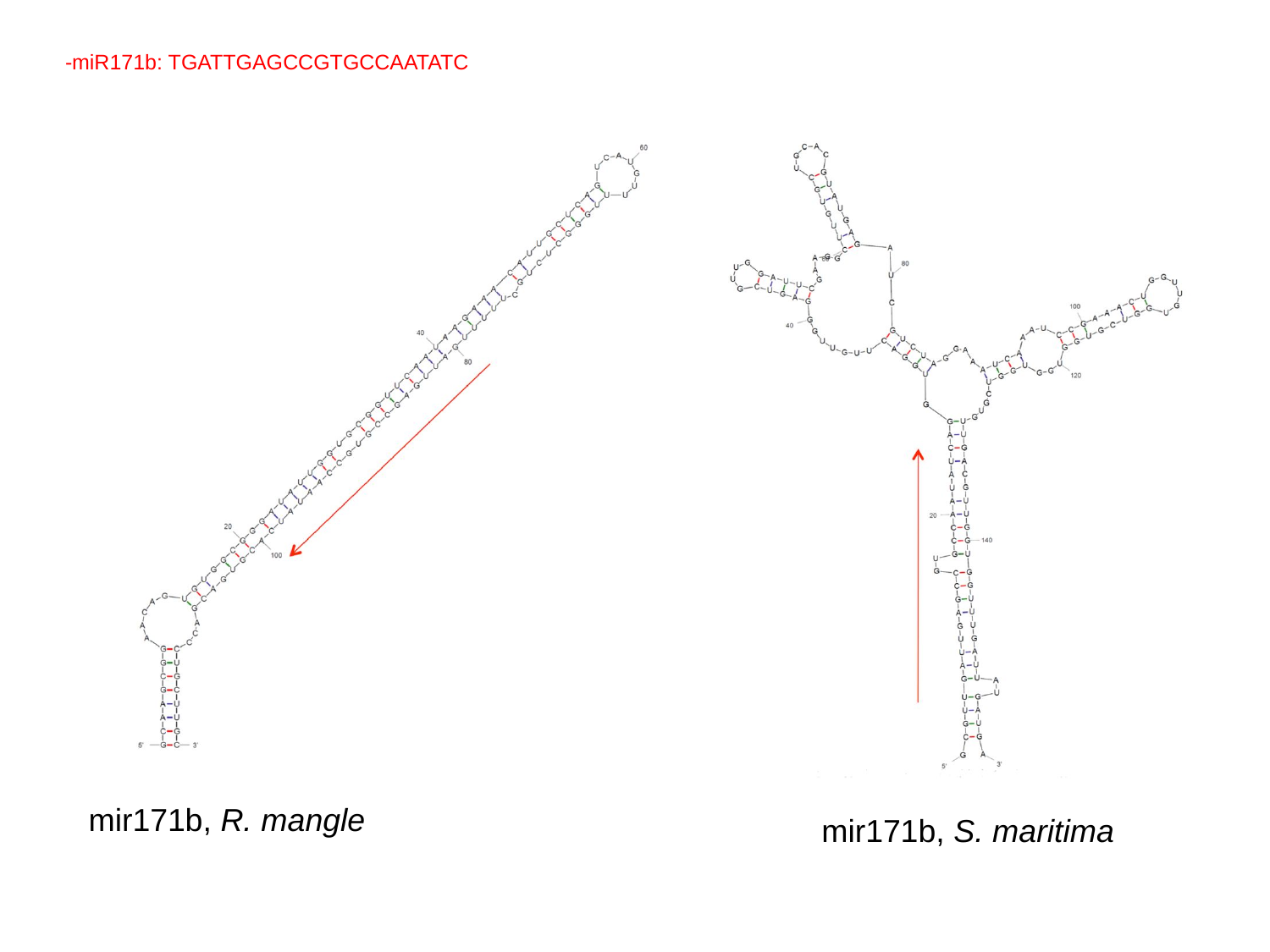

-miR171b: TGATTGAGCCGTGCCAATATC
mir171b, R. mangle
mir171b, S. maritima

## Slide 17
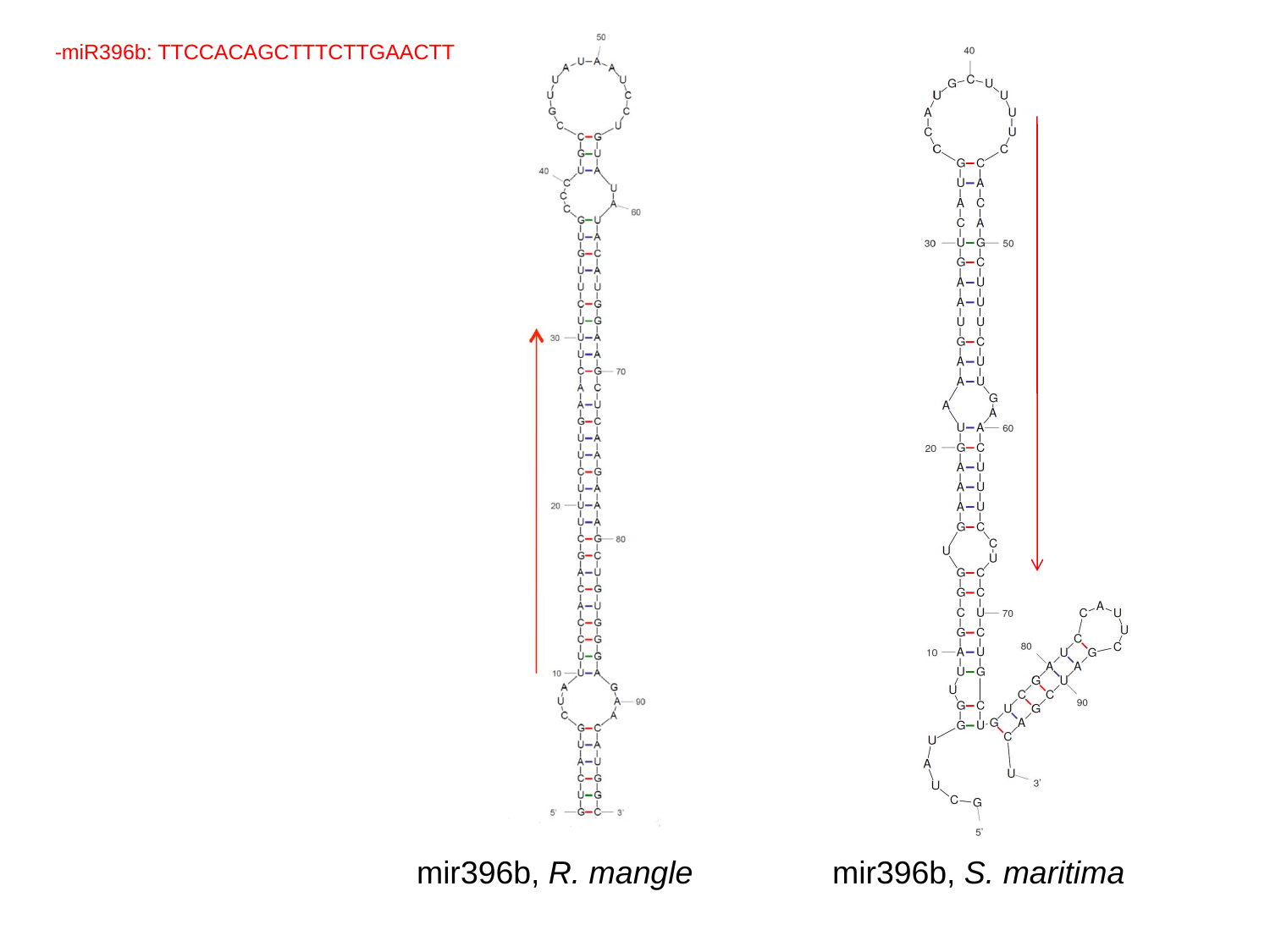

-miR396b: TTCCACAGCTTTCTTGAACTT
mir396b, R. mangle
mir396b, S. maritima

## Slide 18
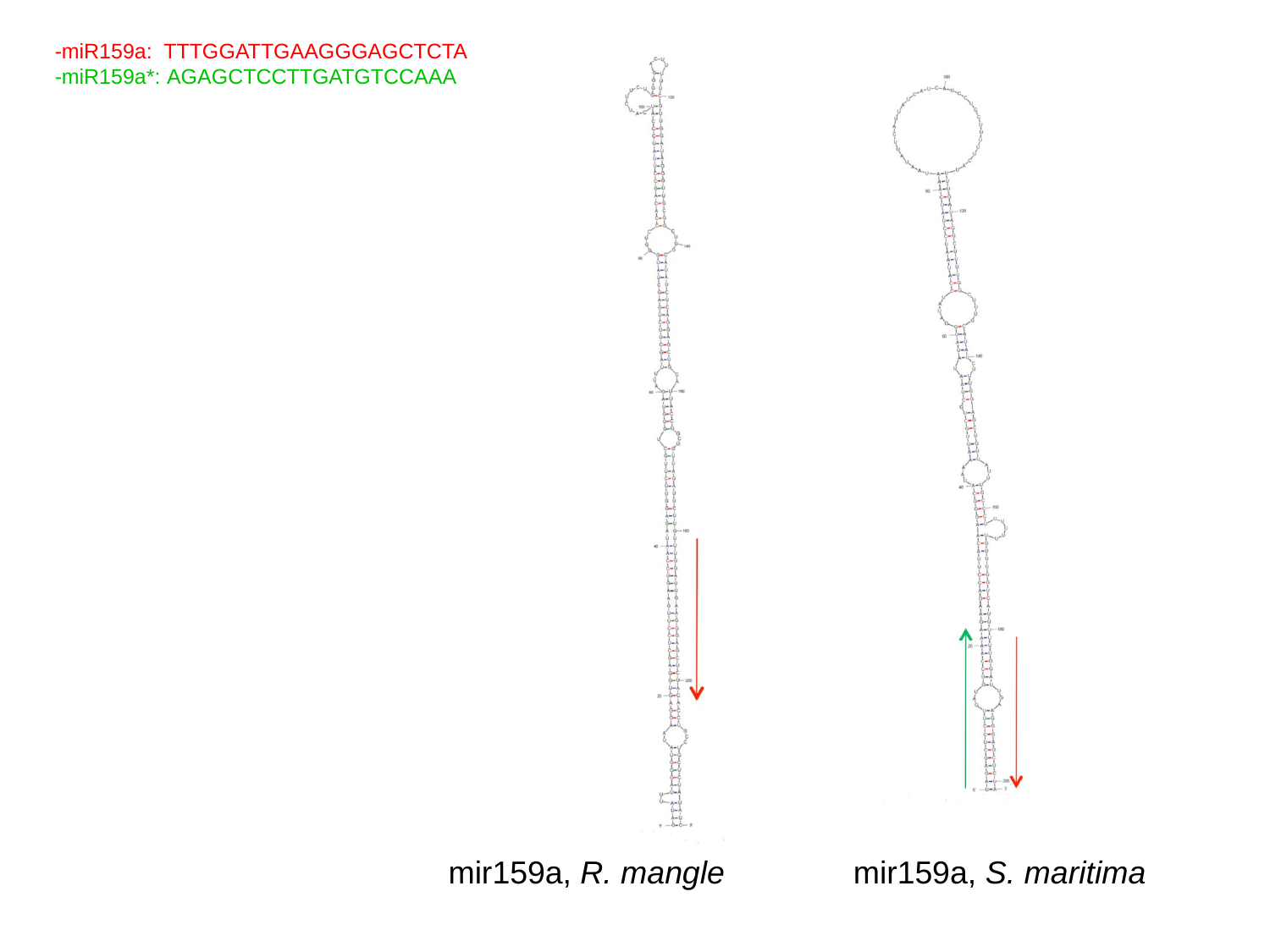

-miR159a: TTTGGATTGAAGGGAGCTCTA
-miR159a*: AGAGCTCCTTGATGTCCAAA
mir159a, R. mangle
mir159a, S. maritima

## Slide 19
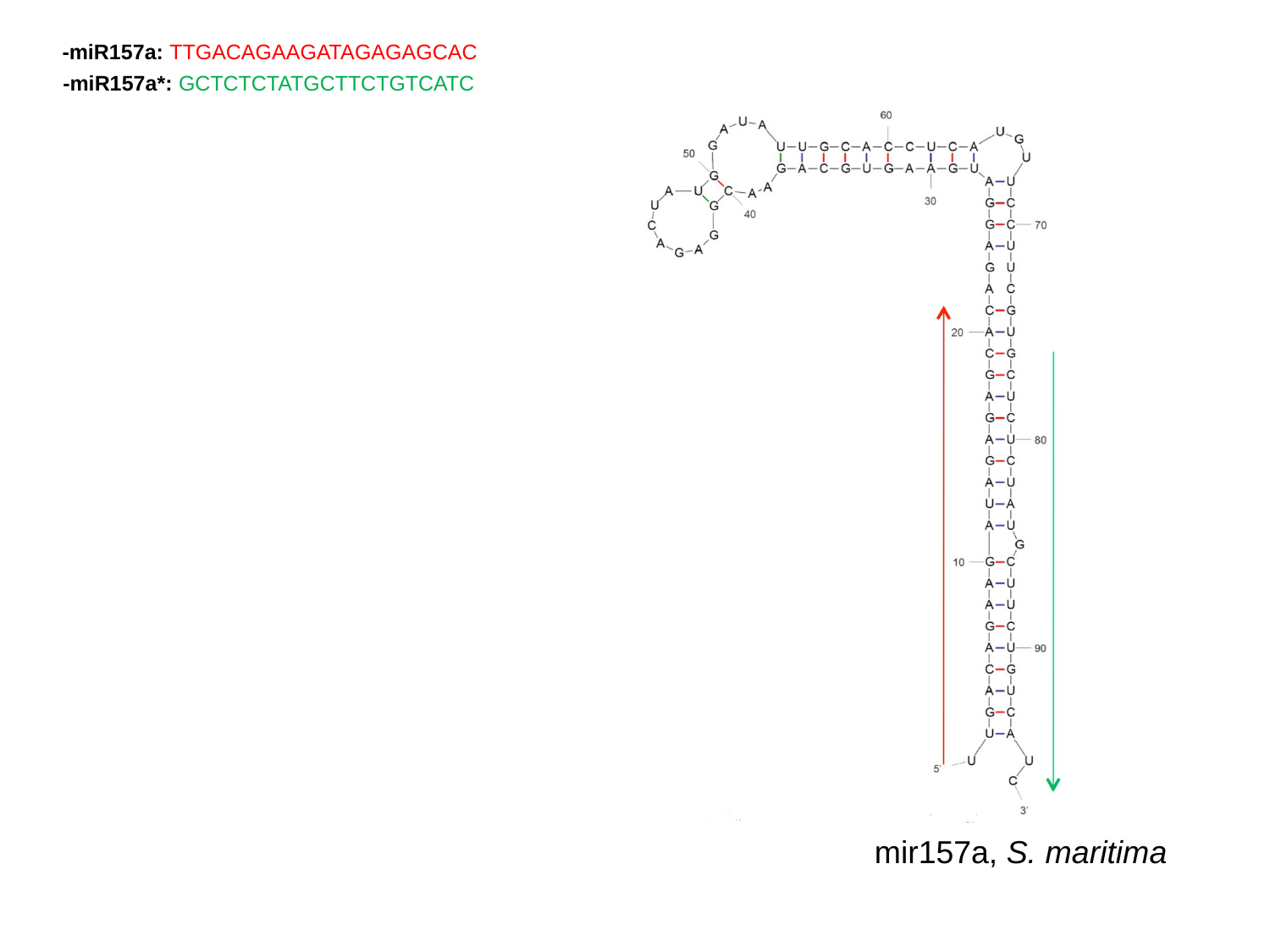

-miR157a: TTGACAGAAGATAGAGAGCAC
-miR157a*: GCTCTCTATGCTTCTGTCATC
mir157a, S. maritima

## Slide 20
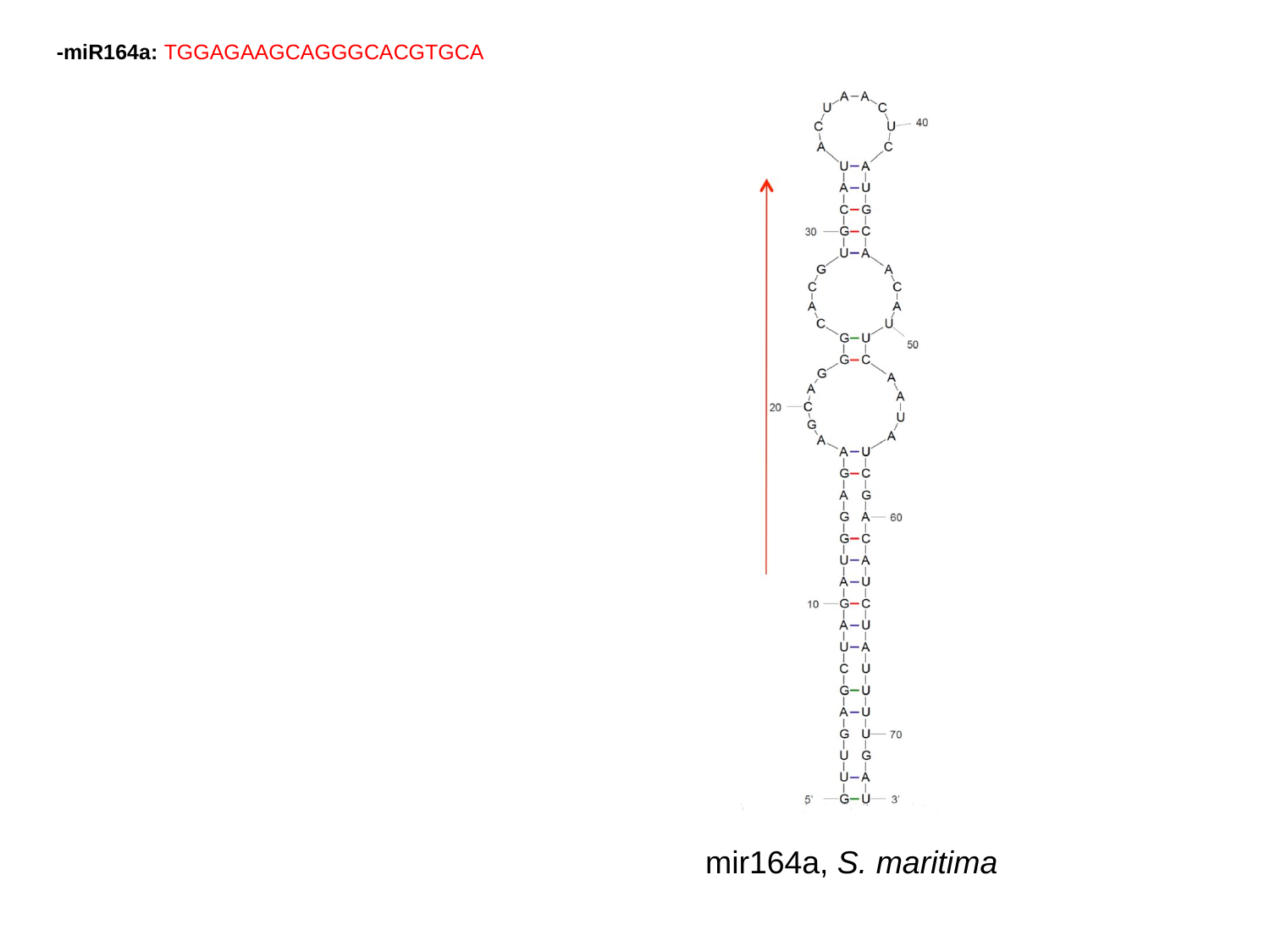

-miR164a: TGGAGAAGCAGGGCACGTGCA
mir164a, S. maritima

## Slide 21
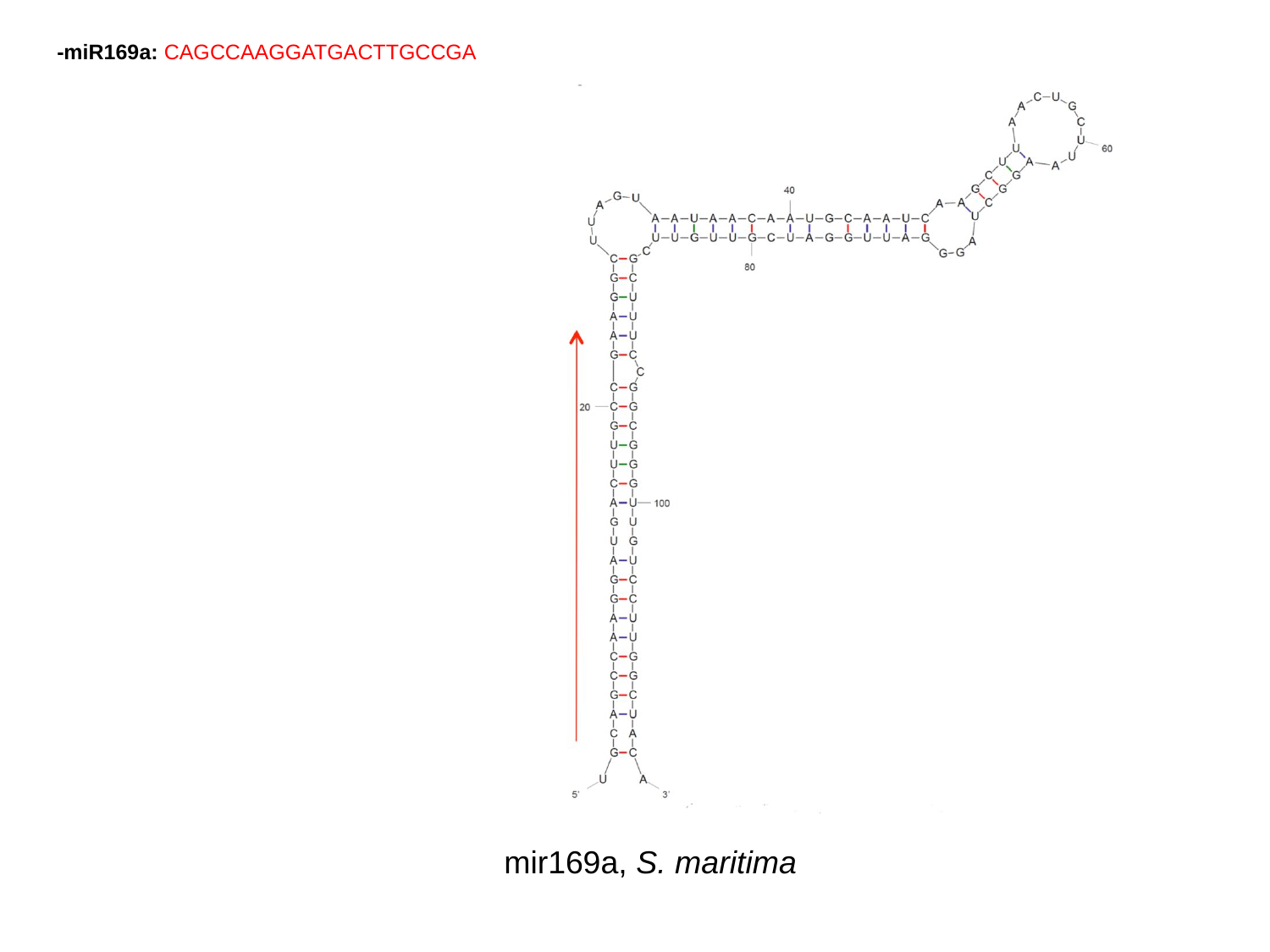

-miR169a: CAGCCAAGGATGACTTGCCGA
mir169a, S. maritima
