# Supplementary material for: Novel and conserved miRNAs in the halophyte Suaeda maritima identified by deep sequencing and computational predictions using the ESTs of two mangrove plants
Source: BMC Plant Biol. 2015 Dec 29;15:301. doi: 10.1186/s12870-015-0682-3 (PMC4696257; doi:10.1186/s12870-015-0682-3)
Supplement: Additional file 6: — Details of the precursors of the conserved miRNAs present in S. maritima and/or R. mangle. (DOCX 19 kb) [file 12870_2015_682_MOESM6_ESM.docx]

**Additional file 6**

**Novel and conserved miRNAs in the halophyte *Suaeda maritima* identified by deep sequencing and computational predictions using the ESTs of two mangrove plants**

Corresponding author E-mail: sachingharat113@gmail.com

**Details of the precursors of the conserved miRNAs present in *S. maritima* and/or *R. mangle***. LM- Mature miRNA length, LP- Precursor length, MFE- Minimum Free Energy, NP- Precursor not present. *mark against a sequence indicates the presence of miRNA* sequences. SRX001383 is the accession numbers of *R. mangle* in NCBI database.

| **miRNAs** | **Sequence (5’-3’)** | **LM**  **(nt)** | **Precursor accession****  **(*R. mangle***  ***S. maritima*)** | **LP**  **(nt)** | **MFE**  **(Kcal/mol)** |
| --- | --- | --- | --- | --- | --- |
| sma-miR166a | TCGGACCAGGCTTCATTCCCC* | 21 | SRX001383.65087  *S.maritima*472472 | 188  117 | -58.83  -55.40 |
| sma-miR171b | TGATTGAGCCGTGCCAATATC | 21 | SRX001383.222397  *S.maritima*24592 | 118  160 | -59.9  -36.44 |
| sma-miR396b | TTCCACAGCTTTCTTGAACTT | 21 | SRX001383.43452  *S.maritima*30680 | 97  83 | -42.3  -15.10 |
| sma-miR159a | TTTGGATTGAAGGGAGCTCTA* | 21 | SRX001383.55766  *S.maritima*440067 | 220  201 | -95.9  -72.04 |
| sma-miR157a | TTGACAGAAGATAGAGAGCAC* | 21 | NP  *S.maritima*1861596 | --  96 | --  -48.00 |
| sma-miR164a | TGGAGAAGCAGGGCACGTGCA | 21 | NP  *S.maritima*10099 | --  73 | --  -16.10 |
| sma-miR169a | CAGCCAAGGATGACTTGCCGA | 21 | NP  *S.maritima*467708 | --  114 | --  -51.70 |

*mark against a sequence indicates the presence of miRNA* sequences.

**see Additional file 4 for sequences and other details.
