# Supplementary material for: Novel and conserved miRNAs in the halophyte Suaeda maritima identified by deep sequencing and computational predictions using the ESTs of two mangrove plants
Source: BMC Plant Biol. 2015 Dec 29;15:301. doi: 10.1186/s12870-015-0682-3 (PMC4696257; doi:10.1186/s12870-015-0682-3)
Supplement: Additional file 7: — Progress of the Stem-Loop PCR for the novel miRNAs in control and NaCl treated S. maritima and the rice cultivars, and in S. portulacastrum collected from its natural saline habitat. (PPTX 4 mb) [file 12870_2015_682_MOESM7_ESM.pptx]

## Slide 1
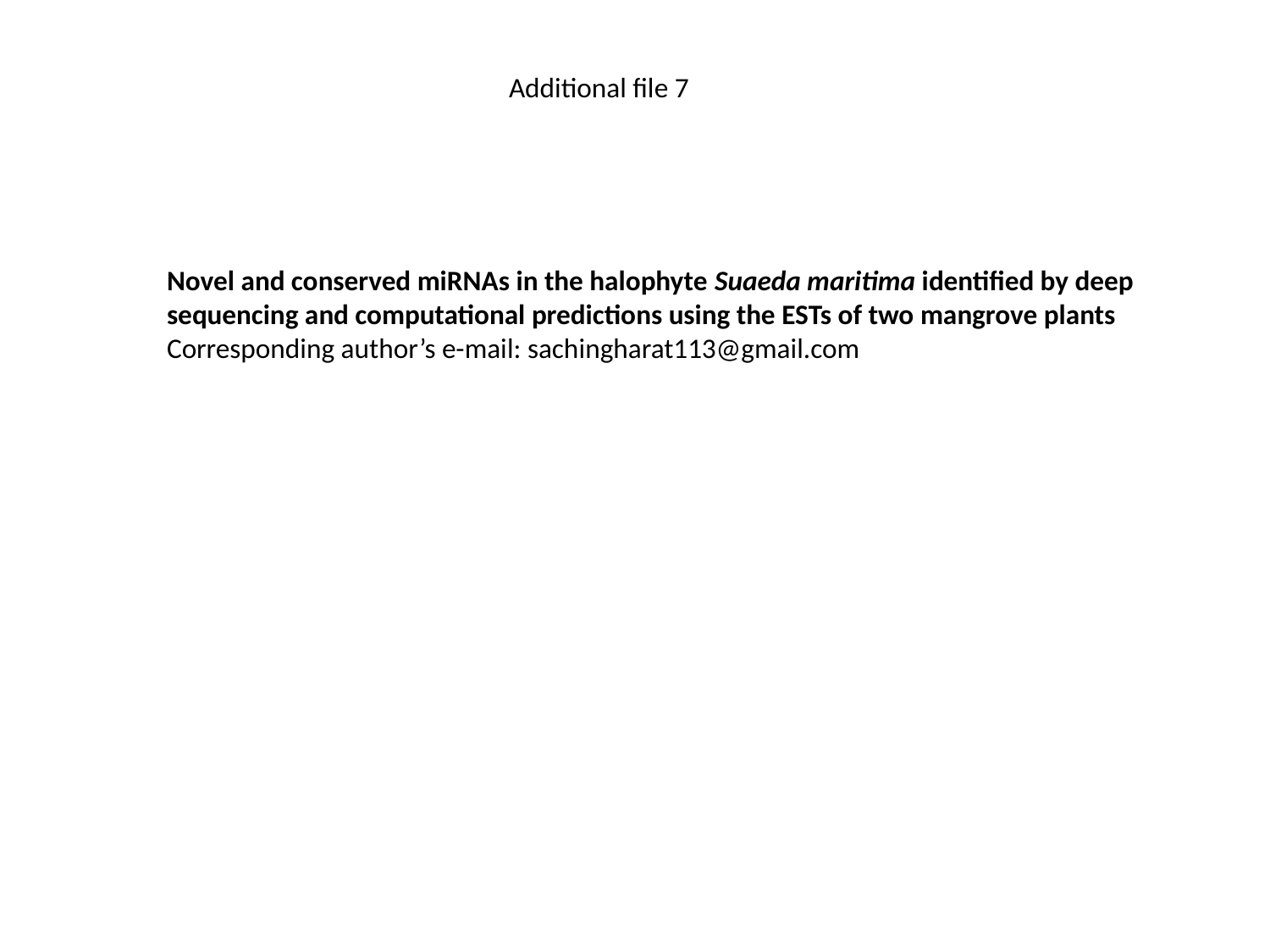

Additional file 7
Novel and conserved miRNAs in the halophyte Suaeda maritima identified by deep sequencing and computational predictions using the ESTs of two mangrove plants
Corresponding author’s e-mail: sachingharat113@gmail.com

## Slide 2
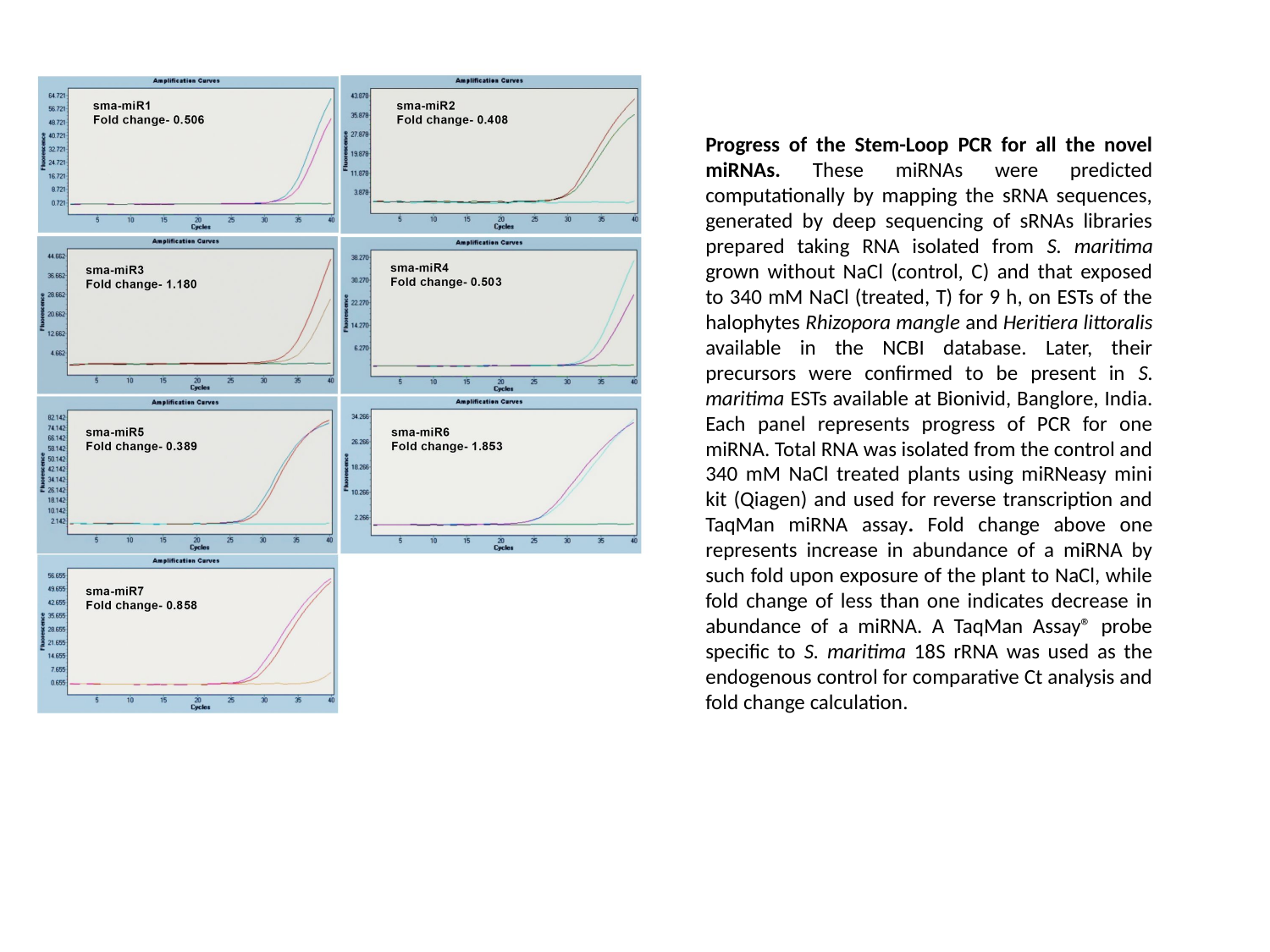

Progress of the Stem-Loop PCR for all the novel miRNAs. These miRNAs were predicted computationally by mapping the sRNA sequences, generated by deep sequencing of sRNAs libraries prepared taking RNA isolated from S. maritima grown without NaCl (control, C) and that exposed to 340 mM NaCl (treated, T) for 9 h, on ESTs of the halophytes Rhizopora mangle and Heritiera littoralis available in the NCBI database. Later, their precursors were confirmed to be present in S. maritima ESTs available at Bionivid, Banglore, India. Each panel represents progress of PCR for one miRNA. Total RNA was isolated from the control and 340 mM NaCl treated plants using miRNeasy mini kit (Qiagen) and used for reverse transcription and TaqMan miRNA assay. Fold change above one represents increase in abundance of a miRNA by such fold upon exposure of the plant to NaCl, while fold change of less than one indicates decrease in abundance of a miRNA. A TaqMan Assay® probe specific to S. maritima 18S rRNA was used as the endogenous control for comparative Ct analysis and fold change calculation.

## Slide 3
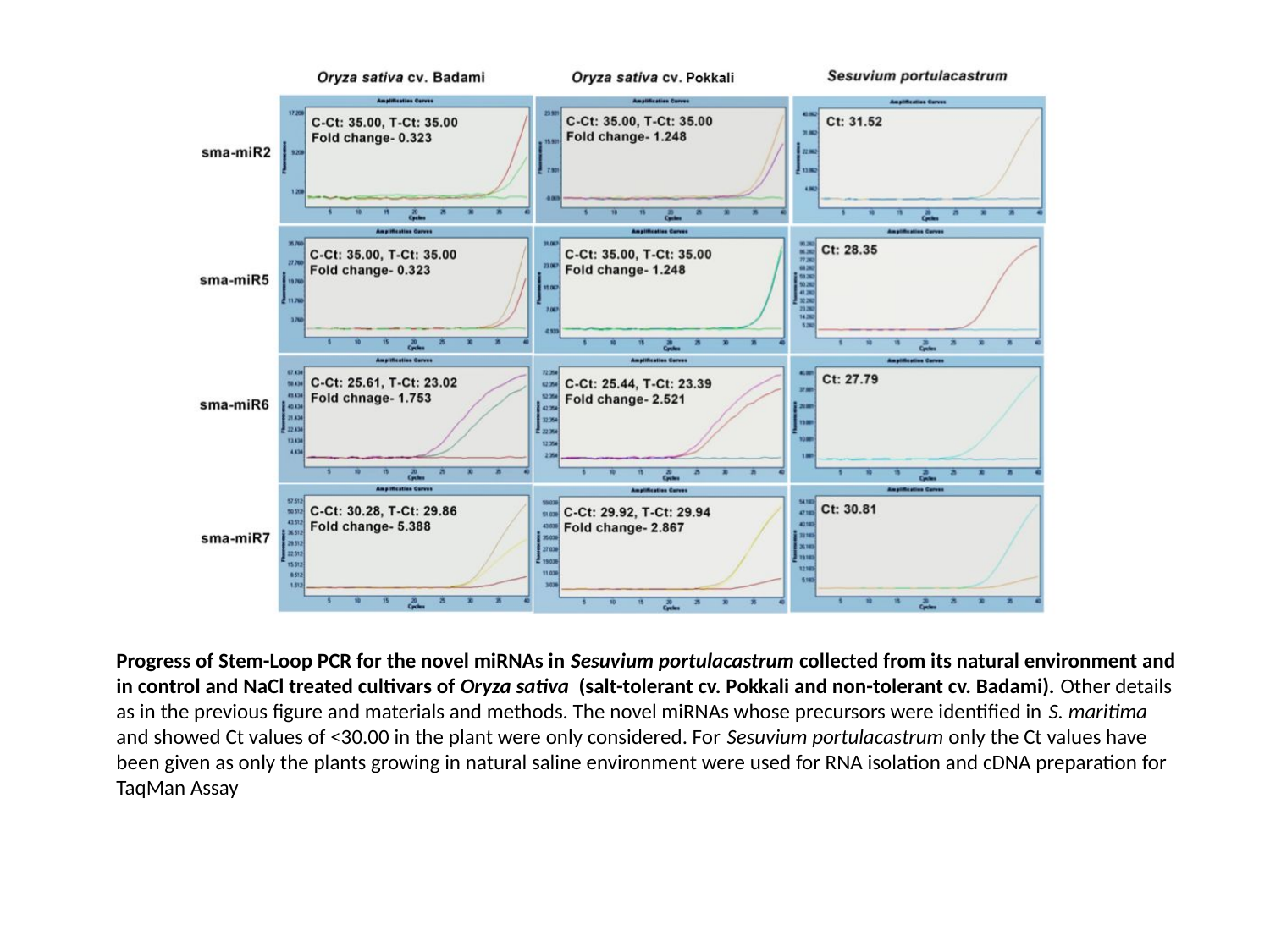

Progress of Stem-Loop PCR for the novel miRNAs in Sesuvium portulacastrum collected from its natural environment and in control and NaCl treated cultivars of Oryza sativa (salt-tolerant cv. Pokkali and non-tolerant cv. Badami). Other details as in the previous figure and materials and methods. The novel miRNAs whose precursors were identified in S. maritima and showed Ct values of <30.00 in the plant were only considered. For Sesuvium portulacastrum only the Ct values have been given as only the plants growing in natural saline environment were used for RNA isolation and cDNA preparation for TaqMan Assay
