# Supplementary figures and images for: Novel and conserved miRNAs in the halophyte Suaeda maritima identified by deep sequencing and computational predictions using the ESTs of two mangrove plants
Source: BMC Plant Biol. 2015 Dec 29;15:301. doi: 10.1186/s12870-015-0682-3 (PMC4696257; doi:10.1186/s12870-015-0682-3)

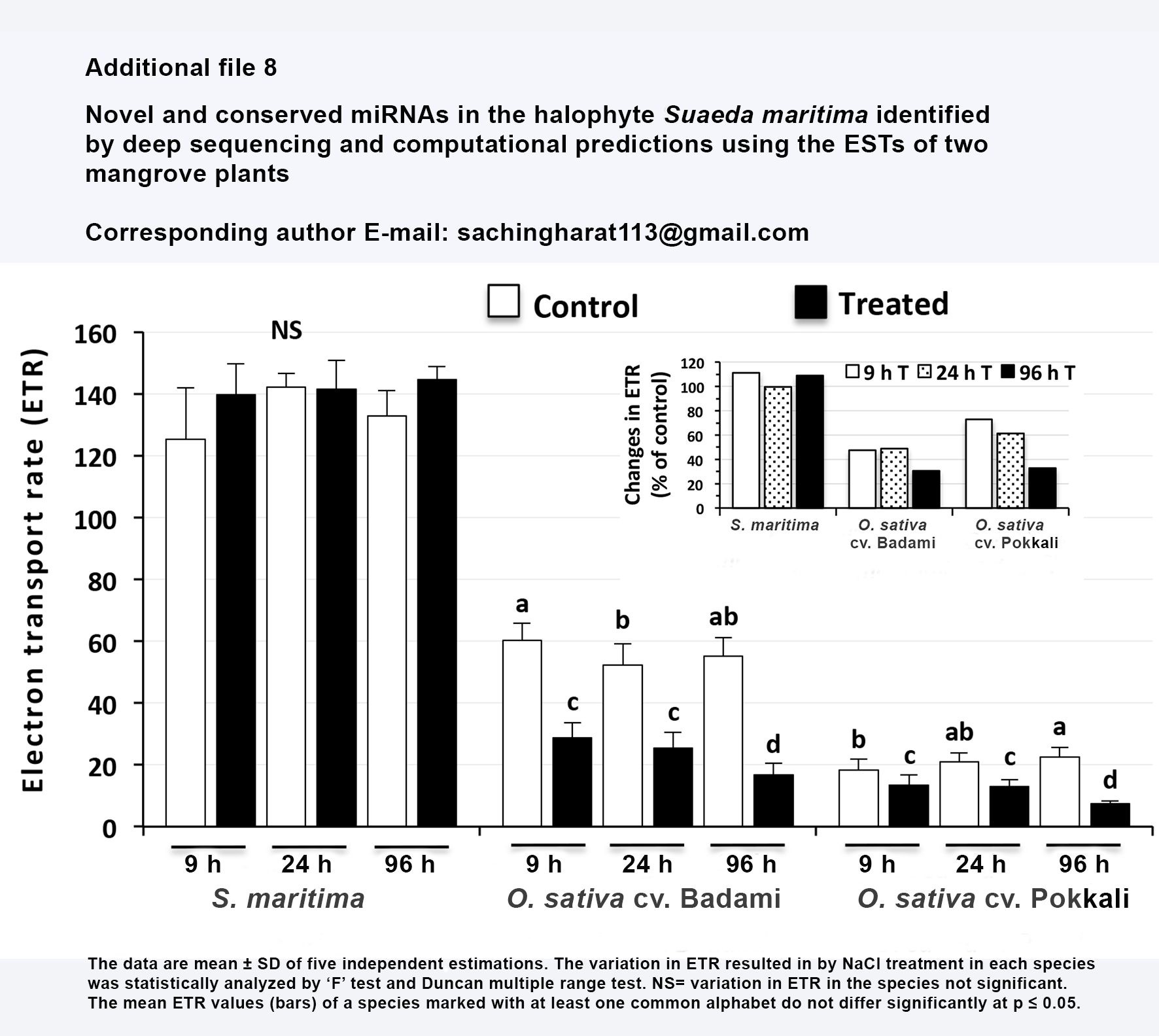

Supplement: Additional file 8: — Changes in the relative electron transport rate (ETR) in the leaves of the test plants determined after their exposure to 255 mM NaCl ( O. sativa cv. Badami/Pokkali) or 340 mM NaCl ( S. maritima ) for 9 h, 24 h and 96 h. (TIFF 8 mb) [file 12870_2015_682_MOESM8_ESM.tif]

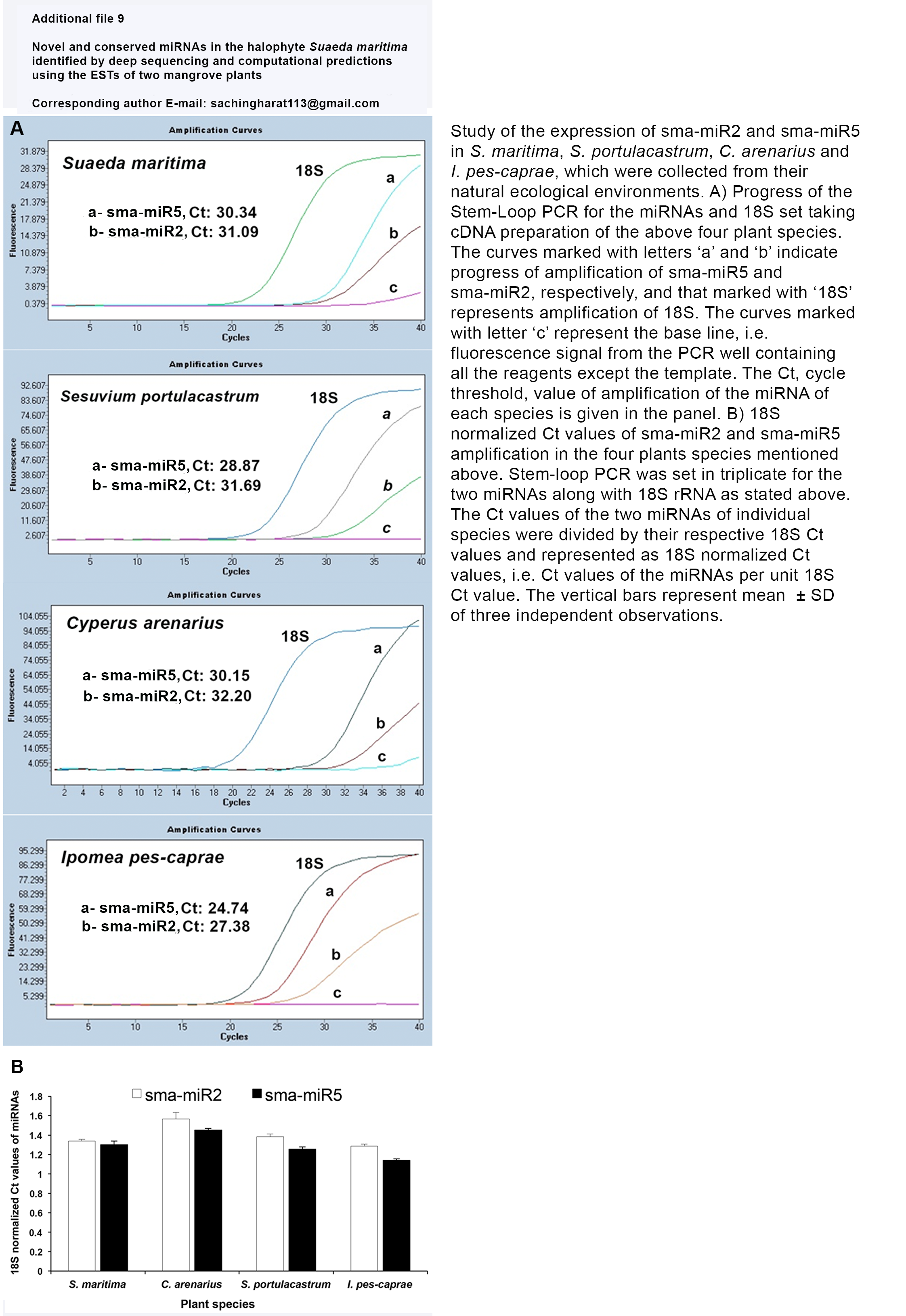

Supplement: Additional file 9: — Study of the expression of sma-miR2 and sma-miR5 in S. maritima, S. portulacastrum, C. arenarius and I. pes-caprae, which were collected from their natural ecological environments. (TIFF 20 kb) [file 12870_2015_682_MOESM9_ESM.tif]
