# Supplementary material for: Novel and conserved miRNAs in the halophyte Suaeda maritima identified by deep sequencing and computational predictions using the ESTs of two mangrove plants
Source: BMC Plant Biol. 2015 Dec 29;15:301. doi: 10.1186/s12870-015-0682-3 (PMC4696257; doi:10.1186/s12870-015-0682-3)
Supplement: Additional file 10: — The target ESTs of the conserved miRNAs represented in Fig. 2 . (DOCX 242 kb) [file 12870_2015_682_MOESM10_ESM.docx]

**Additional file 10**

**Novel and conserved miRNAs in the halophyte *Suaeda maritima* identified by deep sequencing and computational predictions using the ESTs of two mangrove plants**

Corresponding author E-mail: sachingharat113@gmail.com

**Mangrove target ESTs:**

SRX001383.184716

AAGAGACCTTCCCGGCCCCACCTTAGACGCTCTAAGATCCTTTTTCAAACCTGCTCGCTCCCATTTCGAGTCAAGAGTCCCATTTCGAGTCAAGAGATAGATAAATAGACACATCCCATTGCACTGATCGGGGGCGTTCGTAGTGACTGAGGGGGTCGAAGACCAAGAAGTGAGTTATTTATCAGCCAAGCATTCTTCTTACGGCTAGATCCAATCTCCTGGTCCCTGCGGAAAGGAAAAAGAATTTCACGTCTTCCTTTCGGGAAGGGAGGGTTAGGAAAATACC

SRX001383.195417

AAGGCAGTGGTCAACGCAGAGTTGGTTCCTTATTTACGGCCGGGGCTGATTGCTGCTAGCAGTGGTGTGCCTAGTGTAAGTAATTCTCTCAATACCGCATCTGGGTCTACCTCTACCAATGCTATTNTGGGGCTGCTGCACCAGAACTCTATGAACTCAAGACAAAATTCTATGAATAATGCAAGCAGCAGTCCTTATGGAGGGAACTCTGTTCAGATTCCTTCTCCTGGTTCCTCTAGTACAA

SRX001383.67404

AAGCAGTGGTATCAACGCAGAGTTGGCCATTACGGCCGGGGAGGTTAATGAAACAGAAAAAGTACACGGCTGTTACAACAGAATCCCCCTCAGACGTGGAGCCTTGTTGTATCTGTCAGGAAGGATATACAGATGGGGATGATCTCGGAATCCTAGACTGCGGGCATAACTTCCATGCCGACTGCACCAAACGGTGGCTAACTCNCAAAAACCTGTGCCCCATTTGTAAGATGACCGCCCTGCCTACTTGAGGGCGAAAATTTGTC

SRX001383.163041

AAGAGGCATAGCTTTCATGCCAACAAACATATACTCACAGTTTTTCTTACCTTGAGTAGTGACAAGACGA

TAAGCATGGCCAAGTGCAAGAGTATCAGCAGGCCTAAGAAGCTTAACACGAGTGAACTGCACAGTCTTCT

GCTCCTGGTTGTCCTCCCCAGACACTGGCAATGGTATAATCAAAGAAACATAATGGCCTGGGTTCATCCT

CATGACTTCACTGGCTGTAATAGGCCAA

SRX001410.05634

TTGTAGAaCTTCTGGTGCCAtATACAATGGGGACCCACATACTGTCTGaGCATACTtGCCTGGATCTACACTTCTTGAAAGACCAAAATCAGCAATCTTCAGCaCCAAATCATCCATGgAaCCCGAAAGTAGAATGTTcTCTGGTTTCAGGTCTCTATGAATGATATGATGGGATTGCAAAACTTCCATACCAGCTCCAAGTTGCTGCATGAAACTTCTGGCTATTTTCTGTTGAACTCTTCCATGATGCCAAATATAAGAAGCTAGATTTCCTCCATCACAGAACTCAAGCACCAGGAACAGACAACTTTCaGACTGAAGGACATGGAGaaGGCG

SRX001383.21117 AACNCTTTTTCACGGCCTCCACCACTCTCCTAGTTAATACCTGCTATAAAACTCAACAATTGTGTCTCCT

AATGCCATCTGACTACAGCTATGCCTGAATGTGCAGCAATCACTGCAGCTTAATGCATCAAGCTTCTGAA

AATGGCCCCCCGGCCCGTAATGGCCACTCTGCGTTGATACCACTGCTTCTGAGACACGCAACAGGGGATA

GGCAAGGCACACAGGGGAGTAGGNN

SRX001383.178145

GGAGGGATAGACAAGGAAGAAGAAGATGAGTTCAGCGGAAACAGGACAAGAAAGTACTCCAACTTTATATCACAAGATTCTGATATAAAGTTCCTGAAAACACACAAAATAGAAGGTAGGAAGACAGAACGAGGTCTTACAAGGTCAAGAAAAGGTCTTACAAGGTCAAGAAGAACACTAAAATGTAAATAGAAAAGAGAAATGAAATGTTTAAAAAAAAAAAAACGGTAAAAGGTAATAAATAAAACGCTCGTAAGGGTAAGGTAAGTACGACCCAACCCTTTAAAGTTTTTCGGACCCTAAACCCTAAACTAGTCGCCCGTACCCGTACCTAACGGTAAACGGTTAACGTTTGG

SRX001383.116751

AAGCAGTGGTATCAACGCAGAGTGGCCATTACGGCCGGGTCATCAAGAACGAGTTTCTCCATGGGACATTGATCTATCTGTTTCACTCCCGCCCTTNAGTATTTCACTCTTCCCCCGACGAGTGAAGAAACTGCGGCCTGGCCCACAGGCAACCCCACCCCAATAACCCCCTAAACTGGTCAGGGTAGGGTTCGTTAGACTTTGTGGATTCCTATGAGATCCTCCCAACGGTCTTGCAAGGTCAAGAAAATAAGAGGTTTCTACTGCCACTCCTTTACTTAATTC

SRX001410.09075

GGgATGTGATACCGTAAACCACCCACTAGATTTTGAGATGCAATCTCCGGCACATCAAAaTCTTGTGTCAACAGGAATTGGAAAGACTAATATTAGTGAGCTTATGAGGGCTCGCTCCACTGCTTACACAGGCTTTGCGGAATCTAaTAGGCTTCCGAAGGTCTTGCAaGGTCAAGAAATTtGCCCATTGAGATTCCTGACACAAAAGG

SRX001383.156526

AAGCAGTGGTATCAACGCAGAGTGGCCATTACGGCCGGGCGATCAAAGGTGATTTTGACTAGAGTTTTAG

GATCTTGATTGAAGATTCTAAACAAGCAACATGAATTACCTCATTGGAGCTTTCAAGCCGCCATGTAATA

TTTCGATCATGTTTACGGTGGAAAACTCGAAAGCAGGTCCCAATGAAGAAAGAAAATGGGCAGACTCTTA

TGGTTCCACTTTTTCAAAGTCAAGAAAATAATTGCTGGAAAGAATTACAATAGAACCAC

SRX001383.120796

AAATCTGCGGATAAAAGAGGTAAGTGGCGGAAGCTAGATAGGTACATAAGCAAATAAAGAGTGTACATAAAAAACATTTCAAGATCTGAAACAGATCTACCATTAAAAGAACGAATTGACCCCACTAAAAATACGAGATATAGAGCAGGCAGGTGTAGAGCTCCCTTCAATCCAAACAAGAATCTAACAGCAGGTAATGCAGCTCCTGAGATATGCAAGCCGCAGCCCTATCCAACGAATAAGTCCCCAGAAGATG

SRX001383.174703

GCACCTGTGAAACCTGTTTTGCAATGGAATTAAGAATGGACTTCCACCTAGACACATATGTGATTCTTGGTGTACAGCTTGTAAAGCTCTCTAATTCATTCAGCTGGAGTTCTCCTTCAATCCAAGCTTTTGTGCATATGTGGTCTCCTCAGAAAGGTTCAACTGCTTTCCAAAGTATTCAAAACCTTCCAGAAGGTCTGAAGAGACAGAGCCAACAGAGCTCGATTCAGAGTTTGTATCCACTATAC

SRX001383.35722

TTTTGTTGAAATATGATTGCAGAGCTCTAGCTTCCTACAATCCAAATTCTGGAACATTCAGAGATATTTC

AATTCAAGGGATGCCAAATTGGTTTGAAGCCGTAGTTCATGTGGCTTGCTTCAGTTCATTAGACAGCCTC

ATCGGTATATAAAAAATGAAGTGATAGCCTCTTTTTCCTGAAAGCTTCCTGCTTGATTGGGGTTTTTCGT

AAATACTGGCTTTAGTTTCGTTGTGTTCTTCTCTTGTCCTCGTTTCTAGAATTAA

SRX001383.108393

TTTTCTCTGCACCACCACTTCCTCCACCATCTTCCCTTCCCCAGAAAATGGTGCGAGCACCACCTTTTCGAGTTGCTCCACCAGTCACCATTAGGCAGGCGATACCAGTATATGCTATACCTCCAGTTCAGAAAGAAGAGCCTCCTTCAGTTCGAAAAGATCCTCTAGCTGTTCAAAAAGAGGATCCTCAAACTGTTCAAAAGGAGCTTCTGTCTGTTCAAAAAGAAGTCCCTCTGAACGATCAAAAAGATCTCCCAATTGTTGTTTCCCCC

SRX001383.65087

GAAAAATCTCTGTGTGGAAGCAGTAATTGGGGGGAATGAAGCCTGGTCCGACGACACTGGGTCATGATCAAATATCAATCTAGTCTTTGCGTTAAGGTATTTTAGATCTATGCCATGGATTGGCGAAATTGAAAGTCTGGATGAAAAAAAGGAAAAAAAGTGTCCTCGAGCCAGACAACATTCCCCTCAAAAGAGAAGCTTCCTTTGCAAATCTTCTTCAATTCTTCCGAAGGAGATTGAGAACAAGAGAAGGGTACAGAGAGAGAGAG

SRX001383.169033

AAGCAGTGGTATCAACGCAGAGTGGCCATTACGGCCGGGGGTATCAGATTCTGCCATGATATTTGTATCAACTTGCAGAACTTGTCATCTTCTAAACTTATATTGGAAGAGCTCAATCAGGAAGCTGCAGCACTGCGTTCATTCAAATCCCAGTCTTTAAGACTGGCTGCGTTGCATTCGATTCAAATCTGGACAAGCTTCTATATTAATTGCAACTGATGTTGCTAGTCG

SRX001383.11076

CAGAAGCAGAATTCAAAGAGGCTCATCCACCTGGTGCTGTAAACGTGGAAGTATATAGGCTTATAAAGGAGTGGACAGCCTGGGACATTGCTAGACGTGCTGCATTTGCATTTTTCGGCATCTTTGCTGGCACAGAAGAAAACCCCGAGTTCATGCAAGCTGTGGAATCAAGGATAGACAAAAATGCGAAGATAATAGTGGCTTGCTCTGCCGGGGGTACTCTCAAGCCGTCCCCAAATCTTCCTG

SRX001383.145227

ACTGCTAGACGTGCTGCATTTGCATTTTTCGACATCTTTGCTGGCACAGAAGAAAACCCCGAGTTCATGCAGGCTGTGGAATCAAAGATAGACAAAAATGCGAAGATAATAGTGGCTTGCTCTGCCGGGGGTACTCTCAAGCCGTCCCCAAATCTTCCTGAAGGTCAACAGTCAAGATCACTTATAGCTGCCTACCTGTTAGTCCTCAATGGCTTTACAAATGTCCTCCACTTAGAAGGAGGGCTTTACAC

SRX001383.205893

TAACTTACAATAAAAGACAAAATAAGTTAAATAGAGTTGTGGAAGTTAAAGACTGCAAGTTACAGGACTATGCTGGAGATTACAATTATTACCTCGAGAAAAATCTTGATGCAAGGGCAAAGGAACTTGAACGTGAGGCAGAGCTTGAGGAAAAGGCTCCCAAAAGTGAAAGGACCAAAATCCAAAGAATAGTCAAAGGCTGAGAAGGAAGCTCGAAAGAAACAAAAAATGCAAGCATTCCAGGCTGCTAAACAAAAAATCAAAAGGGCTGAAGAATTCAAAGAG

***S. maritima* target ESTs:**

*S.maritima*26422 CTATTGTAAAATTCACAAAGTACACTCATAGTTTTCAAAATTCACCAAATACGGAATACCCTTAACATATTGTGAAACTTCATAAAATGCCCTCAATGAATAATGAGTGAATAATAACGTAACTTTACCATCACTAGGAGACTTTATTCATGAAATATCCCCGAGGTTTGTTTAAAACGCACAAAATGAAGTCAAGCTATTCCTCTATTCAAAACATACTAAAATGAACTCAGCCAATGATTCAGAATTTCAGATGGGTATTTTCTGAGAAAAATCAAAACCAAATATACAAAAGGTGCTACAGGTAAAGCATTGAAGGGGAAAACAAAAAAGAGAATCACAAGTTAAAAATCAATCCAGCATTCACCAGTATATTTATTGCATATTATTGTGAAAAAAATGCAACCACGGAAATTTGGAAAAGAAAACGAAGTTATAAGTTCAAAGCATATTTCAGCATAAATTGCCAAAAGTCATAGACTCAATGCCATCCTATATGTAGCCAATAAACAATCTTGCCTATCATTCGTTCTTCCCGATTCCATGAGACATGTTATAGATCCCTTTACCAATCATAAACATTGATGTAGCGGCTAGAGCAAGAGGAATGGCAACAGAAGTGATCTTATCTCCAGGTCCTTTTAGATATGTATGCTTGTGGATATTTTGGAAGAACTTCTGCCTCTCAAGAAGCTTTTCCCTAGGGTAAAATGGTGCCTCTTTTTCTGCCATCCTGAATTTCGAGTAGATCAGATGAAACTCTGGAGGTGTAGAGAAACAAGAAATGGTTTTCCGATGAAAGCTACGGCGGGCAACGCAAAGTGCAGATATGGATGTTAATGCTTTCTAAATTTTGGTTTCCTTTTGGTTACCCTATAATTTCTGTTATACGAGTAGAAAGAGAGGTAGAAAGATAGGGAACGGTGACGATATCCACATAAAATTACTTATTTATC

*S.maritima*1694886

CGGGGTAAGATTTTTTTCTTCTTCTTCGGCTTGCTTCAAACGATATCCATGTGCTTACTCTGATATGGGTTTCGTCTTCGGTCGCTTGCTCCAATTTCCTGGTTTTTCTAGGGTTTCCACAAAATGAAATCACTCAGTTTGGAAATTACTCCACCTCCCAATTTGTTCATTCATCAGCATACTTTGCTCCTCGTCGAACTCAGAATGAAACTGGCTCAATTGGAATGTTGGTTGCTGGTGTTGAAGCTTAGATTATCAAAGTAATTAGCTCAAAGCCTCATCCTTCAAACCAAGTGCGTGAATTATGGGTTCATGGTCCAAATATGATGAAAGATTTTTTTTTGGTGTTGGTGG

*S.maritima*155512

TATCAGTTTACTCATACAGATGAACAGAACAAATTCCTGTTACAATTAGGTTTCTGACAACAACATTGTCTTACCAGTCCAGTAACTAACATAGCCAAAGCACTGAAAGAATGACAAATTTTCATATACGAAGCTGCTAATCCGATAATTTTCGTATAAGAAGCTGCTAATCTACTATTGGTTAATCTCTCTTAATGGGTATCTGTCAATGTTTACGTCAACTTCCTTCTTTACATCCTTTTGCAAAAGATTAGAACTCTTATATGATGTTACATCATCTGTGTTAGAATTTTGCAGCATTTCCACCAACATTCCTTGAAGTTTCACATCTTCCACACAGAACTCACCAAATTGGTCACAGAATCCTTCCATACAAGTGAGCAGATTGTCCCTTTCAAAAGACAACCTTTTGATATTGGCTACCAAGGCCTCCTTCTCTTGCTCAAGATCTTTCATCGATTTATTCAACTTTGCTTGTTCCTCCCTGCATTGGTTCAGCAATTTCTGAGTGTCCAGCTTCTTAAACTCAATCAGAGCCTCAAGCTCTTTAACTGATTGTTCAGAATGAAGCAAAGACTTCTCTTGCAGCAGCATTCTGCCTTGTAGATCACTGATTTCCTTTTTCAGTACATTAATTGCGTTGTTTTTCCTTTCCATTTCAGTGTTCATGCAGACTTCAGACTTCTCAAGAGTATCTTGTAGGGCAACTATCTTAAACATATCTTCTATACCTTGTGAGAATGAAAAGAGAACCACTTCCACGAAATTCTGTTCTAGCGATGAAGCAAATTCCTGAATGCACCTTATCTGCCCATCTTTCTCTGCTGTAATGGCCCTACACAATTCTTCCTTTTCAAGTTCAAAATTGTTCTCTGCTTCAAGCCTGGCAGCTTCAGCTGATGCTACTTCCCTTTTCATGGATTGTTCCTCATTTACTTTTATTTCTTTCTGAAGGTTTTGAACTATAGCATCCTTTTCATTAATGATGTTGACATAGTTCTCAGCTTTCTTATATGCTTCAACCACCTCCAACTTCATTGCCTCTATAGCTGCTTCATTCTGTATTAACTTATGTTCCACAGAATCGGCCTTTTGCTCGAGATCCTTAATTTTCTGATCTTTTTCTTTCATTTTGCATTGATAAGACTCATTCTCTTCTCTGAGAACCTTCTCTGTCTCAACCTGCTCGAGCAGGGATGTTTCCATTTGCTTACATACCTCTTGACATTGTTCAAGGCATGTTTTAAAGCTTTCTGCTTTTGATTTCCATATTTGCAGTTCCATTTTTGTCTGTTTAGCCTCATTTATCTTTGCTTCCAATGTAGAATTTGATTTTTCTAGAGCACTAGACATATTCTTAAGAGCAACTTCCATTTGGCTAAGTTGTTCTCTTGAGCGAAGTTGACAGGCTGACGATTCTTCAAGCATCTTTTTATGCTCCACAAGTGCACTTTCTAAAGAGGTACTACGCTCTTCCAATGCCTTTATTGAATTCAGCTTTTGCGTCAAAATTGCAATCTTCTCATGGGCCTGCTTGGCATGAACTTTGGCATTCGATGGAGAGCAATCTCCCATCACCTCGAAACTCTGAGCAGAAGAATCTGTATTCATCTCGACTCTTTCCTTGTTGGATATTTCAACCTGATTCTCTGTCTTGAACAGCTTTGAGTAAGCATCAGAAAGTTCAGATTTCATCATCAAGAGGAGTATGGAAGTCTCTTCATCTGAAACTTCCATGGCAGAGTGACAATTATCTAACTGCATCTTCAGTTGGTCTAATTGTTCACTTTGTGTCTTTATATCAGATTCATAGCTTCTCATATCTCCCAGTAGCTTCTCCATTTTAGAGTTCCATTCAACTTCTCTTTCCTTTAAAGCCAGAGAACAATTGCTGTGCACCTGCTCCAGATCTTTAAGCTTGTTGCGAAGCTTATTAAGAGAAGGATCCACTTTTCTTTTGTTTATTTGAGCTTCCCGTAGCTCCTTCAAAGATTCAAGCAACTCCTTGTTCTCTTGCTCCAGGTGGGTAACCTTGTAATCCAATTCCTTCATGAGTATATCTTTCATACCAAGTGAATTCCTCATATCTGCAATATCCTCATCACGTTTTGTGTTGAAACTTTCCAACTTCGATTTCGCTTCCTCATACTCTGCTAAAACATTTTCAAAGTTCTGTTTTGATTCTGACAGTTGAACTTCCAATGTTTTCCTTCGGCTTTCTTCATGTGCCAGAGCTTGATTGCACATCTTTAGCCGAGATTCAAGGCTTTCAGTGACTCTCATTTGAGAATCCAATTTCAGCTGCAAAGCTGTCATCTCTTCCAACATACCCGATTTTTCAGATGCCCATTCCTTTTTACTTGACTGAAACAAGTCTTGCAGCCGCCTGTGAGCTTCTTCAAGATGTTCAAACTGTTCTTTCTTCCATTTCAGCTGGTCTTGGACACTCATGCTTTGCTCTTCCAATTTCGATATAACTTCCTCTCTGTGTCTCAATTCCCTTCCTTCTCGGGCCTTTTCTTCTGCTTCAAAACACTTCTTTTGTGATTCAGATAGAAGGTTTTTCAAGCCTTTAACCTCTTTATTGCAAGCGCAAAGCTTCGCCTCCAACTCATCATTTTTTCCTGTTGCTTCCTCTATAGCTGACACCAATTTTCTGTTTTCCTCCTCCAACTTCTGCACCTTTTCACCATTATCAATGCGCATTTTTTCATTCACAGAACTGACTTGCTTAAGAGATGATTCCATTTCACGCAATTTAGACGTGACCTCTCCATACAGCTGCCTCACTTCAGCCAACTCCTCTGATTTCTCATTTAGTTCCTTAGCCTGCTTTTCTATCTGCAATTTTGCTTCTTGATTTTTCGATATTTGCTCAATATGAGCTCTCCTTAGACTTTCGGAAAGTTCTGTCTTGATTCTGTATTCATCCTTCAACTTCTCCAATTCAGCTTTTAATTCTTCCAACTCCTTGTACACTCCATCCATTTTGCTGAAGCAGCAACAGCAACTTAGCAAAGAGTAGTGATGAATCTTGGGAGAACAGAGAGAAACAGAGAGAGTGGAGGAGAGCGGGAGGAGTGTTTGATTTGAATTTTGAGTTTGGGCCCTTTTTTGCAACTGCTCTCCTGAGTCCTGGTATTGGCCTCTTTT

*S.maritima*714933

TAAGAGGTTCAAAAACCATACATAAATGTTGTTTGTGAAAAAAGTGACGGTATAAACGGAGACAATGAAACCTATCGTCAGGATCAGAATCATTTAACCGTTTAAGAATTTCTAATTCTTTCAATCCTGTCTTGTGCATAATTTCATTGTTTCGTATAATCTTAACAGCCACATCTTGATTATCGCGGGCCGCATCTCTGGCACGAACGACATTAGAAAATACTCCTTGCCCAGTATATCCATAGACAGTATAACGTGAATCCATAGTTTCACCAATTCTTACGCGATAATATCCTTCTGCATCATCCCAGTTATCAGTTAAAGAAGGATTTTCTGGTACACTCTTGTTTCCATTTGATATACCATCTGGTTGAGGAGTATGTTGTGTAGCATGAAATGAATCAGCTTCTGCAAACATATCCCATTTGCCTTTTATTAAATCAGGATTTAATACATGATCAGGCTTAGTGGTCTCTTTATCAGTTGCTGAATCAGAATCATTTAATAATCTATCAGTTTCTTTAGGAGATGGAATTTCTGGAGAATGCTGAACAGATGTTTCAGTTGCATAGCTTCCATGTGGAGAAGGATTCATAGAATCTTCACTATGCAAATTACTATTATTTGCTCCTAATCTCTTCAATAATTCCTCCCTCTGCTTTCTTCTTTTTTCAATAATTTCTTCTTCGTTTTCATCTTCCGCAATATTTATATTTACATCAATGTCAGAATCTGAAGATGACTTATCATCACGCTTAAGACCTTCAGAAAAGCTGTCTTTATATTTATCTCCTCTATCTTTTCCTCTATGAGATCTAGAATATCGTTCTTTACTATGTGATCTCCTATCATATTTTCTATTATATCTCTTATCTCGGTCTCTGTCTCTGTCCCTATCTCTATCTCTCTCTCTATCACGATCACGGTCACGATCACGATCGCGGTCCCGATATCTATTAGCACCAGGCGGACTTCTTGCACGGTCTCTATTAGGTATAGGACTTTTAATACGATCTCGTCTCCGTCTTAATTGATCTTCGCGTTGCTTGATCTGTTCTTCTCGTCTTCTGACTTCTTCTGTTCGTCTTTTTGCTTCTTCTTCTATTTCTTTTATTTCTTCATCTCGTCTTCTTTGTTCACGCCTCCTTAAATCGTCTTCTCTTTTTTTAATTTCCTCTTCTTTTCGTCTGATTTCACGTCGCTTTTCATCTTCCAAATGTTTAATTTTTCTTTGTCTATCTTTATCTTTTTCTCGACACCTTTCATGTTCCTTATCACGATCTTTTTCTTTTTCACGTTCTTTTTCTTTTCGCATGCGATCTGACTTTAAATGAGTTATGTCTTTACTTACACTTCTATCTCTTGTTTTCTTAATTTCTTGACTGGAACCATGATAACGTTTTAATGGACTTCTTACACGTTCACTATTCTTGTTTTTATATTCTTCATCTGACTCGTCATCAATTAGGTTAATGGTTTCAACTGATTTTTTAGCACTTGGCTTTTTTTTTTTATTGGTTTTATCAGCTTCTTCTGATTCTGATAAATAAGCTCCTAATCTGGCTTGAAGTAAAGCCTTTTGCTTAATTAGTTCTTCTAAATTCATTTCTTCTTCGTCCAAATTGATTTCTGTTATTACTGGTCCTTCAACTTTTTTCTTTTTACTATTTTCAGGTGGTGGTGGAGATGGAGGTCTTTTTTGTTTACCATTCTGAATATGGTCGCCATTCGATTTCTTCTTATCTTTTAATTTGGTCTCTCCATTTTCTTTTTTTGTTTTTTTATGTTTTCGTTCACTTCTATGCTTGTGTTTCTTTTTCTTTTTTTTCTTATCTAATCGAGTGTCATCGATTACCTTAACGGAGTCAGCAGAAGAATCATCAGACAAACTGTCAACTGCCATGTTTGTTGACGAAAGAAAATAATAAACAATAAACGGCTCAGAATTGTTTTTAGCAGTTCCTGAAAGAGATCGTCATTTCACTTTCATAAATTAATTAACTTACACACAATTAAATCACAGTCACACAAACAAATTACAACAATAATAATAGTTCCTATCTCGTTGACATTTTGAACACGAACAAAATCAAAGTATCAAACCCAGTTTTTTATGTTATTATAGAGTTGAGCCGTCAGACCTCTCACCAGAGAAGTATGCGTGCACAGTATGTGCTTT

*S.maritima*37141

ATTACGAATGAGCCACATAGAAGTGCTCAATTGGCCAAAGAAAAAAGTGCAATTGATGAAATGGACATAAACAACAGGAACTTTCTATTTCTTTGTCAGATTTTTCTGAAAAAGCATTTCATATTAGAAATTTGCACCCCTAGTAAATTAAGAAGGACAATTTGGAACTGTAAAGCCCAAAATCAAACATAAACAATATACTAGAGGCAAAAGCAAGATACGAGAATACATCTGTATATTAATTGTAAAAATCAAAGGGAGATCTCTTCACAATGAAATTTCAACTCATGTTCAAAACGTTGTAAAACAAAATGTTCCTCTGGAGCTATAATACTGGTGACAATGCATTTATCATCTGAGAATGGTTTCCTACCTGCTCTACCAGCTCTATGTAGATAGTGCACTGCACTTTTTGGCAAATCAAAGTTGTAGATGTGTGTAGTCTCCGGCAGGTCAACTCCCCTTGCTGCTACATCCGTCGCCACAATAAGATAGTGACTACTCTTTCTAACCTCCAAAAGAGAAGCTGCTCGGGAATTGAAATTCTGGTCTTCCTCCAGAAGGGAAATGTTGGAAAATTCCTTACACGAGTTTTTTAAAAAATCAATCAGCAGAGTAGTTGTCGGCATATCACCTGTCCTTTTTGACTTCTCAGACTGCTCATTGACAAAAAGTATGGCTGATACGGGCAAGTCGATTTGCAACATAGATAAAATAGCTTGAAATCTCTGATTTCTTGGACAAATCATAAATCTATGATGCAAGCATGAAGGCATTGGCTCAACTGGATTAACATGAACATGTACCACATTGCTCTTGGTCCATTTCTGTTGCACACAATCATATAGAAAGTGTCGATGCTGAGGAATAGATGCACTGGCAAAAACAGTTTGACGAGAATGGCTTGATGAATATGATGTCAACAACTTCCGCAGAGAACTAACTTGTTTGGAGGAGTTAAACATGAAATCAACCTCATCAATTATTAGCACTCTCAGTGCTCCAAGATCTAACACGCGCTTCTCAAGCATTTGACACAAACTTGCCACCGTAGCCACTACTATGGTTGGGGGATCTGCCTTTAGCCAAGATCGGTGCCTTTTCAACATTCCTCCGTCTAAAAGAGCCATAACTGTGCATGACTTTTGCTGCTCATGTCCAAAATCTGTGGACTTTGGAGCCAACATTCGTGCAACTTTCGTAACTTGCATACCAAGTTCCCTTGTTGGCACCACAATCAATGCTTGAACAGCAGATCTCTGAATATTGATTATTGAAAATACTAAAAGGAGGTACGTCAAGGTCTTCCCAGAACCTGAGCATGCAGCACACTATCACAACCGGAGAGAAGTATTGGAAGGCCTTGTTTCTGCACCTCTGTTGGAGTCACAAATCCATTTTCTTCCATCCTGCGAAGCACATGTTCAGGCACGTGACCTTGGCACAGTTCCCGAAGAGTTGCAAAAGTCCGAAATTGAGCAACAGAATCTCCATTTGCAGTTGCAGTTGTATGATGCATAGAAGATGCAGTTGTTTTGCTAAATTTTGGGAATGAGCTTCGAAATTGACTTGCAAAAATTGAATAATTAGGTTTACATTTACGATTAGGGTTTTGAGAGGTGAAAATGGGAGAGAGATTATAGTTCAATTTTGGTGAGATGGGGATATGAGGGAGAAGATGACGACTACAGAAGGAAGTAGCCATTACAAGTTTGCAACAACAAACACAAGATTCTTTGTCAA

*S.maritima*26981

TGTCAACAACCAAACAAACCTTAACAAATGTCATCACTACATTTGTGTCACTGTCTTCTACTCATCCCTCCTTTTTAATGCTCCTAACTACAAATATCAATTTCACTCAAGCTCTCAATTTCCAGCATTCATATTAAACACACCAATTTTTATTCATAAACCCTTTTCCCCCTTTTTGTTTTTCCTTCAATTTTAACCTCTATTTTCTTCTTAATTTTATGGATTTCTGATTCAAACCATCAATTTCTCATTTGGGGATAGAGTAGAGGCATGTGTGATCCGGAAAGAACCATGGCTGTTGAGGGAAACCAACAGAATGATGTCTCCGAGGTGAATGGTGTTTTTGTATCTGGAAGCGCAATGAATGACGATTCTGAAATTAGGAACAGTGTTATTGATGATGAAGCTAGGGCAGCTGACAATTCCAAAGCAATATCTCCTATGAAAGACGAGATTGATGTTTTTGAAAGTAAAAATGTTGCCGTCAAGGATGAAGCGGAAGATCATGATGCTTCTAAACTCAATTCTCCTAAACAAGATGATGATGCTGGTTTGTCCAATTGCTCAGCTGTTGAAATGTCTGCTTCTTTGAAGGTCGGCAGTAATGTTTCTGAACTGAGAAATGATGTTGACACCCTTAAACTGGTTTCACCAATGGAAGAAAAGAAAGATGCACCTGAAATTAAAAGCAATATTGTTAGCAGTGAAGCCAAGGGTTTTGATTTCTCCAAAGTGATTTCACCCCTACAAGATAAAAATGATGCGCCTGAAATTAGAAATGATGCTGGCAACGGAGGAGGTGAGGGTTTTGACCCTTCTAATGTTATTTCACCCATGGATTTCACCAGTGGAGCTAAGGGCCTTGACTTATCTAAAGTTATTACACCCACACAAGATAAGAACGATGCTTCTGAAATTAGATACAATGTTGTCAACAGTAGAGCTGAGGGTTTTGACTCCTCCAGAAGTACTTTTCCCATGGAAGTCACCAGTGGAGCTATAGAACTCAAGAATGTTGCTTCTGAAATTAAAGACAATGATATTAACAGTGGAGCTGAGGGTTTTGACTCTTCTAAAATAATTTCGCCCATGGATGTCACCTGTGGAGCTGAGGGTTTTGATTCTGTAAAAGGGATGACACCGAGAGAAGACAAGAATGATGCTTCCGTAATTAAAAACGTTGCTGTCGACAGCGGAGCCAAGGGTTTTGACTCTTCCCAAATTGTTTTACCTGTTGGTGTCATCAGTGGAGCTGAAGTGGACTCTTCTAAAGTGATTTCACCCATGGAATTCAATAATGCTGTGTCTGAAGTTAGAAGCTATGCTGTCAACAGTGGAGCTGAGGGTTTTGATACTTTCCAAATCATATCCCCCATGGAAGATGAGATTGATTCGTCTGAAATTGCAAATGATGTTGTCAGTAGTGGAGCTGAGGGTATTGATGCTCCTAAAGTGGTTTCCTCCATGGAAGGTGAAAATGTCGCGTCAATAGTTGGAACTAATGGTGTCAGCACTAGAGCTGAGGGTGTTGATGCTCCTAAAGTGATTATCTCTACAGAAAGTGATAATAGCGCAACAAAAATTGGAATTAATGTTGTCAGCAGCCAAGGTGAGAGTTTCGATGCTTCCAAAGTGATTTCTCCTATGCAAGGCGATAGTGCTTGCCTGGTCAAGTGCTCAGCTACTGATCAAGTTATGCATTTGACTATGGAGAATGGTGAGGAAGACAAAGAGTCTAAAAAGAGTGCTGAATTGAGGGAGAGAAAAAAGAGCAAGTATTTGTCCCCTCCTTATGTGAACTTGGGCAAGGAGGCAAAAGGTTTGTCTAATTTGAAGGATTTAGAAACAGAAAGAGCTGATTTTCCTGTAGATGGGGAGGGAAATGTATGTATGTCTGACAAGCTGGCTTCACCCTCTACGGTCGGAAAGACTGGAAGCAAAAAGAGAGGGAGAAAGCCTTCAAGAAAACCTGTTTCTGCCTCTGCTAATCTACAGGAAATAAGTGCATCATCAGCTGAATTACTATCAGAACTACAATTGGTGGCTTTAGATTGTCTGCATCCTTGTGATAATAAAAATTTTGATCCTAGCGAGATCTTCTTTACCAGCTTTCGGGCTTTGGTATATCGTGGTGAAGAGAAAACTGATGCTGCCGCTGGGAAGGGTGATAATAGTCAAATTCTTCAGGTAGGAAGCCCAGATGAAGGTAAATCTGGAGCAAAAAAGAAAAATAGAGACGAGAAAACAACAGGATCTAGTGAAGGTGGACCAAAAAAGAGAGGAAGAAAGAAAAGTTCGGCAGTTGGATCTACAAGCGTGCTTCCAAATGTAAATGCCTCAACTGGTTCATTCACTATAGAAAATTATCCAGCAGCCAATATACCTCAACAGATGCGAAATGAGCAAGCGACTGGTCATGTCACCGAAGTGCCTATTCAGACAGCTGCAATTACTCTGGATTTCTCTCGGGAGACTATACAGCCACTTCCATCGTCTCTTGATCCCTCTCAAGGAACTAATAAGACAAACATTTTGTGTTTTGAGGGCAAGGAAACAGTTCCCATTCCGGGTTCTGTCCCTGTTGGTATTCCTGGTCCTAAAAAGAGGGGAAGGAAGAAAAAAGGGGAGAGCCCTGCAAATCCAAGTGCAAGTGCTGTTAATGCTGATGGTATTCCTGAGAAGAAGAAGAGACGGAGGAGGCGTAAGGATGGGACTTATTTTGGGGATTCAATGACTACTGCCCTTTTGAGCCCTACTGGAACCAATGCTAAACCAATTTCGTTGGAAGTATGTTTGCAAAATGTGGGCCCACGATCAACAGTTCCACCACCGACAGCAGCTTGTTTGAACAGTGGAAACAACAATCAAGGGTTGACTCCGCCTAAGCCAATGCAGCCTGATAATGGGACTGATTCCTCTTCTCTTGCGAAGAATCCTGGTACTCCTGCTATTGAGGCCCCATCGATAGATCAGATAAGGAAGAATCTGGAGATGATGACAGCTATGCTTGAAAAATCAGGGGACAATCTGTCTTCTGAAATGAAAGCAAACTTAGAGATTGAGATCAAGGGGCTTCTGAAGAAAGTTTCTGCCGCTCCAGGGTCATCCCCGTCTTAAGTGATCAGTAATTGTAACATATGTAGGTGCTATAGTGAAGACCAGTCTAAAGGGCTTTAACAACTCTTGCTAAGGTTGTTTCCCTTTCACCATTTGGTCATTAGGTTGATTTATGTAGCTCCCTGCTCAGCAAGTCTCGTTCCATCTCTCCCCTCTCCCTAACCGACTTTTGTTGCCCCTTTTGCTTCTCTTGTGCCTCACTTTCCTGTCTCGTTTGCATTGTTGGTGAACATGAACCAAAGCATTGAATTATTTTTTTGGAAGTTTCATGTATTTGGCACAAGAGAATTGTAGTGGATCGCTGTTGAGACTTGGTTCTTGTTATTGGTTATGCCTATGAGCCATTAATCTATAAAACTTGATAGCATGTCTCGCAACGTTGATATAAGC

*S.maritima*41280

CCCAGTCGAAGACCCCACCGTGGTATGCGCCAATAAGACCACCAAAAGCCTTTGTGGCACTAGTGGTACACAGAAGTCATGGGTGATCATTGGTCCGATGCTTCGGGCGAAACCAATTCCCAGGGTGTGACGGGCGGTGTGTACAGGGCCCGGGTACATATTCACCGCGGCATGCTGATCCGCGATTACTAGCGATTCCAACTTCATGTTCTCGAGTTGCAGAGAACAATCCGAACTGAGGCAATCTTTCCGGATTCGCTCCGCCTTACAGCCTTGCTTCCCATTGTAATTGCCATTGTAGCACGTGTGTGGCCCAGCCCATAAGGGCCATGCGGACTTGACGTCATCCCCACCTTCCTCCAGTATATCACTGGCAGTCCCTCGTGAGTGCGGCACGCACCTTTTTGTTTGTTTCGGAGCCGTTTTGGCGGAGCGTACTAAACCCACTACGTACCGCACCACCGGGCGGCTCGCCCGAACGCCGAGTCTTTCTCCGCCGCCAACTCGACGTCGTCGTCACCTGCAAGATAAGGCCAAAAACTTGACTTTACTAAACAAGCGAAAAAAGCCCTTTCTATAAAGCCTCAAGTCCTTATTGAAAACTAAAGCGCACACTAGAAAGTACTTCGAAAGAAAGGCGCCGGCTCCCTTCTTACTGACAGCACAGCTACGTGCTGGCAAAAAATGTTGAGCGCTGGCACGTCACTCGGCTCCTTGGCTCACGTCGGTTGCAAAGACTTTCTCCTTAAGCGCATGTCTCAGCAACACAAAACGAGGGTTTCGCTCGTTATAGGACTTGACCAAACATCTCACGACACGAGCTGACGACAGCCATGCAGCACCTGTATGAAAGTCAGTACCATCCCGTTAAGGACAGGTTTTCTTGTTCATATGTCAAGGGCTGGTAAGGTTTTGCGCGTAGTATCGAATTAAACCACATGCTCC

*S.maritima*7497

ACTATTAAAAACACAACCAATTAATAGTAATACTAACTATCCTGGATCTTATCAACAGCAATTTCCTATTCAACAAAATTTCAATCAAAATCCTCTATCAATATCTCAACCAATTGATAGTAATACAAACTATCCCGGATCTGCTCAACAACAGTTTCCTATTCAACAAAATTCTAATAAAAATCCATCAAGGACCAATCCTAATGGATATGCAAATCCAGAGCAAGGAACTATACCATTTTCAAACGGTCAGTCAATTGTTGAAGGTCATCAATTTTTACCAAATCCAAATAACTACGGAATACCCAATAACTATGGTAATGTTGAAGAAACACAGAATAATTATTTATCTGAACAAGAACAAGAACAAATAAATCAACAAAAAAATAAAGTACGATTTCCATCCCCGTATCCATATCCACAAAACAGTTATAATGGAAATCAAAAACCAGTATCATCAAATCCTTCTAATGTTAATCCTAATTCTTGGATTCGAGGACAGCTCAAGGTGAAACAAGGTTCTAGTTTAAGCTCTAGTTCTAACACAAATGAACAATCAGAATATGAAGAATTTCAAACACCTAATAATCAATTCAACAAACCAGCATCAACGTTTGATTTAGGTAATAGACAACAAACAGAGCACAATAAACCAAATAATTATGATTCATCAAGAGATGCAAGTCAAACTCACATAGATTATGGGCAACAATCTTCTATACCAAATGGTCAAGGTTATAACTCAAAATCTTCACCAGATTATTCAGCTCACTCCATGGTTAATGGACATGCACAATCACAGTATTCAAATCCAATACCACAAGATAGACGAACTAATGGAGACTCAAAAATGAATAAACAATTTAAAATAGTTGTTCCTAATATTTCCAAACCTATTGATAACATACAACAGTCAAAACCAAACATAGAATTGAGTTTTAACAGAGATTCTGCTGGTAGAAGATTACCAAATAATTATAATGGTTTACAATACCCTAAGCAAAAAGAAGATTCAGGTAAAATTCAAAATCCTAGTTACACAGATAATATTCAATCTCCTTCACAAGAATCTCCAGTATACTCATCATCAACTACTAATTCTAAGTGTCCAAATGGCTTCAGTGGTATTAAACCACATCCTACAGAATGTTCCAAATTTCTGAGTTGTGCAAATGGTAGAACATTTGAAATGGACTGTGGTCCTGGAACATTATTTAACCCAACTATATCTGTATGTGATCACCCGTATAATGTGGAATGTAATCAATTAGTAAGACCTACATCAACCGCAATAGAAGAAGATTATATTTATACTACACCAACCACAATACAAGAAGATTATAATCCACCCATTGATATGAGACAAGAGTTTGATCATGAAACTAGTAATAGTGATTTGGTAACTGAAACTGAGCCACTAGAAAATCATAATCAAGCAGTGTTAGAAACTTTGCCAAGCGAAAATAAACAGTCAAAAATCTTAAGAAACCCAACATCTATTGATTTAGCAGATAACTTTTTGCAAAACACTTCTATTATGCATACACCCCCTAAAATTGTAAACAACAGAGTCAACAACAATGTAGCAGTGAGAATTGATTTGAAACCAAATAGTACTCAGTCTATTCGGTTACGTGGAGGTCCAAAAAGCTCCGAAGGATTTTTACAAGTTCAAGGAAAGCCTTTTCAATGGGGTGTAGTTTGTGATGAACTCAACTCATGGACAATCGAGAAAGCAGATATTGTTTGTAAACAACTTGGATTTAAGAGAGGAGCAGAACAAACATGGCAAGGTTTAACTGTGACTACTGATAATCCTACTAGATTGTTAAGAAATATAGGAGTTACTAAAGTATCATGTAATGGCCAAGAAAGTGTATTTCATAATTGCAAGCTTCAAAATGATAAAGCTTGTAATGTTGAAAGAGACGCTGTCTGGGTGAAATGTCGATCAAATTCAGGCTCTGAATGTCAACCGGAAGAAGTGTCGTTTGATGGAAAATGTTATAAATTATTTGTTCCTGTACTAGAAAAACAAAGTAAAACTCAAGACATTGGCTATAGCAAAGCTGAAGCATTAGAACACTGTCTTAAACGGGGAGGAAAGCTTTTAGATATTAGCTCACAAAAAGAAAATGATTTCATATCTGAATGGTTATCTAGACAGAAAACAGAAGGCCCTATCCTAACATCGGGAGTTGGTGTTTCATTATTAGGAAGTCCCATATGGATTTGGGAAGGCACTGAAAATCCATTTGTGTATCAAAATTGGTGGCCAGGCTGGGAATTCAGAAAAGCTGTTTCACCCAATATACAAACAAACCGTGCTCTATGTATTGTTTTACAAAAGTCATTCCCTTGTCCTTCAAATCCAAACAGCACAAAATTATGTGACTCTGAGTACTATCACTGGGAAGCAATTGATTGCGGAACAAAGACTGATAGACTACCTTATGTCTGTGAACGAGATGTTGATGACATTGGTTGTGTGAATGGGGCTGGTTCGGATTACACTGGTTCAGCGAATACAACTGCCTCTGGAAATGCCTGTTTATTGTGGGAAGATCAACAAGTGTTAGTAGCTATGAAGTACAGAGTATCAGAAAAAACACGAAGATCTTTATTAAACAAACACAACAAGTGTAGGAACCCTGATGGTACTGATTTACAACCTTGGTGTTATGTACAAACTTCAAATGGAATAGTGCGAAGTGAATTTTGTGATATACCAGTTTGTAATGCTGCAGCTAAATCACCTAAAGTCAGTCGAATGATTGAAGAACCAAAATGTGATGCAGGATTTTTTGAATGTCAACCTAATGAATGTATAACTCAAGCTTGGGTTTGTGATAATCAAGCAGATTGTAGTAATGGTATGGATGAAAAAAATTGTTCTAATATTATGGACAATTTCATTAAAACTTCTGAAGCTCTATTAACAAACCATGAAGCTGAAAAATGGCTTCACACTACTGTTAACACTTGTGCTAACCGTTGCATGCAAGCAGATGGTTTTGTTTGTCAATCATTTTCTCATAATAAAAATGAACAAACTTGTATATTGAATGAAAAAACAAAAGGGGATAACACAACAATTTTAGAATCTGATAAAGATTGGGATTACTACGAAACCGATAAAGCTCTCTGTATAGGAAAGTTTATTTGTGAAAATGGAAATTGTGTTGATAAATCAAAGGAATGTGATGGACATAATGATTGTGGAGATCGTAGCGATGAAACTAAATGTACCAAAGATATGATGGGATATGAGATTCGACTGATGGGTGGCAACACCACTAATGAAGGACGTGTTGAAGTCAAAGTCCTTGGTGAATGGGGAGTTATATGTGATGATAAATTTGATTTGAGAGAAGCTAATGTTATCTGTCGAGAACTTGGTTTCCTAAGTTCCGTTGCTGTAAAACCAAATTCCTATTATGGAATGCCAAATAAAACAAGATTTGTGTTAGATGATCTAGACTGCAATGGAACTGAAAATTCATTATACTCTTGCCAATTCAAAGAGTGGGGAGTTCATGATTGCAACGCTCAAGAGTCAGCTGGTGTGGTGTGTAGAGTCGCTGGCGGGAAAGCTTGTTCAAATGACGAATTTGAGTGTAAATCTGGAGAATGTGTTCCAGTACGTTTCTTGTGTGATTCATTTGCAGATTGTACTGACGGATCGGACGAAACACCAGAACGTTGTAATGTGAGTGTCGCTACTACAACAATAATTCCAACTACTAGTACAACAACTAAGGCTCCAAAAAAATTTATTCAGAAAATAGCTCCAAAGAAAAAAGGATAATTTTG

*S.maritima*41831

GTTGTGGCATGCTTGTGCTGGCCCACTAATCTCTTTGCCTAAAAAGGGCAATTTGGTAGTTTACTTGCCTCAAGGTCACCTTGAAGAACAGCAACAACAAAACCATCATCATCATCTTCATAATAACAATAATAACAACAGTAATAATACTAATAATTTTGGTGAATTTGATTTTTCTTCATTTGGGGTTAATAATTCTTTTGATTCCAACCAAAATAATATTCCTCCTCACGTGTTCTGTCGTGTTCTTGATGTTAAACTTCATGCTGAAGTTGCAACTGATGAAGTTTATGCTCAAGTTTCTCTAATCCCAGAAAGCCAGATTGAGGAGAAATTGAAAGAGAGTGAAGCTGAAATTGAAGGGGATGATGAAGATTTTGAGGATGTTGTCAAGTCTACAACACCACACATGTTTTGCAAGACTCTCACTGCCTCAGATACCAGCACTCATGGTGGCTTTTCTGTTCCTCGGCGCGCTGCCGAGGATTGCTTCCCTCCATTGGATTACAAGCAGCAGAGGCCATCTCAGGAGCTTGTTGCAAAAGATCTGCATGGATTAGAGTGGAGGTTTAGGCACATTTACAGAGGTCAGCCGCGCAGGCATTTGCTCACAACAGGGTGGAGTGCTTTTGTGAACAAGAAGAAGCTAGTGTCTGGGGATGCAGTGCTCTTCCTCAGAGGTGCTGATGGAGAGTTGAGGCTAGGAATCCGAAGAGCGGTTCAACTGAAAGGGCTTGCCTCTTTTCCTGCTCTTGCTAACAGACCTGTGTCCAACAATAGTGTTGCGGCTGCATTTGGTGCCATATCATCTGGATGTGTTTTCAATGTCTGCTATAACCCAAGAGCAAGTTCGTCCGAGTTCATAGTGCCATATCGGAGGTATTTGAAGAGTTTGAATACTTCATTCAATGTTGGAATGAGGTTTAAGATGCGCTTTGAGGCAGAAGATGCTGGTGAAAGAAGGTACACTGGAGTAGTTACTGGAATGGCTGAGTTGGATCCTGTTAGATGGCCTCGTTCGAAATGGAGATGTTTACTGGTAAGGTGGGATGATGTAGAGGTGAACCGTCATGGCAGAGTCTCTCCATGGGAAATTGAACCAACTGGTTCATTTCCGAGTCCCAGCAGTGTAATGGCCTCCGGCCTGAAGAGGAGCAGAATTGGTCTGCCGCTTACAAAACCAGAATTTCCAATTCCAAGTGGAGTTGGAATGCCGGACTTTGGGGAATGCTTAGGTTTCCGAAAGGTCTTGCAAGGTCAAGAAAAAATGGGTTCTCGTACTGTCTGTGATGGTAATGAGAACCCTCACCCTCATCTCACCCCATCAGAACCAGGAAGATGCTTTCCAAGCTCCATTCCCTCAGGTGATTTTGGGAGGACTGCCAAAGGCATAGGCTACAGGGAATCATTCCGATTCCAAAAGGTCTTGCAAGGTCAAGAAATACGTCATCTAAGTCCGGCTAATGCAGACGGTCTTACATCATCAGCCAACCATAGGCGTGACGGTGGTGGTGGGCCCCCTGAAATCTTCGAGGGTGTACAAGTGCCTAACTGTGGAAATGGATGGCTCAAGGCACAAGTATCTTCACCATCATCGGTATTAATGTTCCCATCTACTAGTATTCAGCAGGCTCGAAACTTCAGTTCTTCTGTGTGCCAACCCCGGTTAATGAATACGGAACGCGTAAATTATCCTGCCACACCTCCATACACCTCTCTTTCCGAAGCTGGTGATGAGCTAGTTCCAGCAACTAAAAATAGTTGTAGGCTGTTTGGGTTTTCTCTCACCGAGGGTGAACATGGAGGGCATAAAGAGCGAAAGCATGGTTCTTTTCCACCTACCGAGTCAGCATTCTGGCCTCTTGTTGAAGGACAGCCTTGCTACAAGTCTCCATCCGTGACCAAAGTCGTGAGCAATTGCCCCGGACCAAATGACTTGTATGCTGTAAGAGATATGCTTCTCGATATTGCTCTTTAATGGTATGAAGTTAAAAACTTGTTTAATTAGACGTAGACAGACATGCTTTGGGGTTTCGAACAAAGAATGGTTCATTTTACCCCAGATGACAGTAGTAGGGAAAATCTGCAGAATGCTTTGGTTGCAAATCTGCATTATTTTGTTTTTAAAGTCAGATATGAAGAAAACTATCTCCAAATACATCTCTTGTTTGATTCAAGTTGTATTAAGGAAAACAAAAGAATTGTAATCCTCTAACTTGCTTCGTAGTGTAAACAAGATCTTATGAAGCATGTATGAATTTTTTCCTCGATCGAAAGATTTGATTGTATTGTAATATCAATCTTGACTTCAGATACTTAATGTCATGCATTCATTTGTGCTAGCAAAATTGAACTGCCCATTGAAAA

*S.maritima*39489

ACACTTACTTCTTATACGTACGTACATCTACTTGGAATTCACTAAAAATTTGACAATAGACGTCATGTGGCCCACTCTATTACGAGTCGACTTACGAGCACTTTGCTGTTACTGAGTTCTTCATCGTCGAAATTCCGTTTCCTTAGATCTCTTCGTCAACTCATCAATTGATCTCCCCATTTCTTTCAACGATTCAAATCTTCTCAATCCCTAACCCTAATTTTCTTCGAATTAGGGTTTCTCTTACCTAAATCCTTCCATCAATTTCCACTTCTCCTCGGCGATCGTTTGTTAGTGAGTTTTTGCAGTTTAATCTGAGGAATTTGGGGATTGTATTGGTGTAATTCTGAAAAGTATGAATTACATCCTTGGAGCTTTCAAGCCTGCTTGTCATATATCAATTACGTTTGCGGATGAAAAGAATCGAAAGAAGGTTCCAATTAAGAAAGAAAATGGTCAAACTGTCATGGTTCCACTCTTCCAAAGTCAAGAGAACATTGTTGGGAAGATCTCTGTAGAGCCAGTTCAAGGGAAGAAAATTGAGCACAATGGCATAAAAGTTGAGCTTCTTGGACAAATAGAGATGTATTTCGACAGGGGAAACTATTATGACTTCACTTCTCTAGTCCGTGAACTAGATGTCCCTGGTGAAATATATGAAAGAAAAACTTACCCCTTTGAATTTTCCACTGTTGAAATGCCGTATGAAACATACAATGGGGTGAATGTGAGACTTAGGTATGTCCTCAAAGTAACAATCAGCCGTGGTTATGCTGGAAGTATAGTGGAATATCATGACTTTGTGGTTCGCAACTATTCTCCACTACCGTCAATCAACAACAGTATCAAGATGGAAGTTGGAATTGAGGATTGCCTACATATTGAATTTGAATACAATAAAAGCAAGTACCATTTAAAGGATGTTATCATAGGGAAGATATATTTTCTTCTTGTAAGAATCAAGATTAAAAATATGGATCTTGAGATCAGGCGACGGGAATCCACCGGTTCTGGGGCAAACACTCATGTTGAGACAGAGACACTTGCTAAATTTGAGCTGATGGATGGGGCTCCAGTCAGAGGTGAATCAATTCCTATTCGACTGTTCCTAAGTCCATATGAGCTGACCCCTACACATCGCAATATCAATAACAAATTCAGTGTGAAATATTATCTCAACCTTGTTCTTGTTGATGAAGAGGATCGTCGGTATTTCAAGCAACAGGAAATGACGCTTTACCGCCTTTCTGAGAATTCTAGTTAGTTATCTTTTGGTTTAATCTATTTCGATGACAAGCTATATAGATGGATTCACTGATTCTGGATAGGGGAGAAAACATCTGTTGCGAGATCTGGCAGTGGTGTCAGTCTGTTTTTCCCCCTCTTTTGAAAAAGGTTTGGCCATAATGATGTATCTTTGAATCTTCGTGGTAAATCATCTTCTTTCACTTTTTTTTTTTTTTTAAAGTTTCATGATTCTATTAGTAATCTTGCTTGTACCGAGTTGTAAATCAGGTTTGTGATTGTTTGATGGTTTAGTGCAAATGCTGTCTTGATAATTGTATCCCCAAATTTCTTGCTATCGGTTTTGCATTTCTGTAACAGTGTTTTGTTTGGTACGAGCATTTGTTGTGATACATCCGGGCATTGTTTGCGATATATTGGAGTGTGGAAAAAGAAATTGTTGGTTATTGGACTCTTTGTAGCAGGATTTCAAGTAGAATCTAGTTCTTTCAGATTCGTCCGTGCACTTGTGATTGAGCACATGAAAATTTTTAAAGGTTCAGGTTGTAAAGTTGCTATGGATTCAATTGTAACAATCACATCGTGCG

*S.maritima*41732

ACCTTCATCATCATCATCATCATTCGCTTCGTTCTTCAGTTTTTGCTTCTCACTGTAATTCAGCCATGCCTCGCAACAAGGGAAAGGGTGGTAAACGTAAGGAGGACAAGTGGACATTGAAGTCAACTTCTGGTGGATCATCAATGATGGAAAGTGCATCAACAAATGAGACAGAAGCCATTATTGGTGCATTTAGTGGTTTAAATATATCTCATCAGAGTATGCAAAGTAAGAGTGCAATTGCACCAGGACAAAAGGTTGTTTGGAAGCCTAAATCATATGGAACTTTGAGTGGTGTTAAAGGAGACGAAGTTGAAGCTCAAAGTAAGGCAAAGGAAGGTGAATTTTCTTCAAGTGTAAAGAACAGTTCCCAGATTAGTACATTATTCAACGGGAAACTGTTGGAGAACTTTACTGTTGACAACTCTACTTATTCACATGCACAAATAAGAGCTACCTTTTATCCGAAATTTGAAAATGAAAAATCTGATCAAGAGATTAGAACGAGGATGATTGAAATGATTTCCAATGGCCTTGCCACCTTAGAGGTATCGCTCAAACATTCTGGATCTCTCTTTATGTACGCAGGACATGAAGGTGGAGCATATGCAAAGAACAGCTTTGGAAATATATACACTGCAGTTGGTGTTTTTGTTCTTGGAAAGATGTTCCGTGAGGCTTGGGGAACCCAAGCAAGTAAAAAGCAGGCAGAATTTAATGGCTTTCTTGAGAGAAATCGCATGTGCATATCAATGGAGTTGGTAACTGCTGTGTTGGGTGACCATGGTCAGCGTCCTCGTGACGATTATGTTGTTGTGACTGCTGTCACAGAATTAGGAGCTGGTAAACCCAAGTTCTATTCTACTGCTGAAGTAATTGCTTTTTGTCGGAAATGGCGGCTACCAACAAATCATGTCTGGTTGTTCTCAACAAGGAAGTCAGTGACATCGTTTTTTGCAGCATTTGATGCTTTATGTGAAGAAGGAACAGCAACTCCAGTATGCAAAGCATTGGATGAAGTCGCAGATATCTCTATCCCAGGATCAAAAGATCACGTTAAGGTGCAAGGTGAAATTCTGGAAGGTCTAGTTGCTCGTATTGTAAGCCCAGAAAGCTCAAAACGGTTGCAGGAAGTTTTGAGGGACTGCTCTCCTTCAGCAGTAGAAGAGGCTGGGCTCCACTTGGGTCCAACACTACGGGAGATTTGTGCTGCAAATCGGTCTGATGAGAAGCAGCAAATTAGAGCTCTTCTAGATGAAATTGGTTCCTCCTTTTGCCCCAGTTTTGTAGATTGGTTGGGAAAAGAGGCAGATGATGTTAGTTCCAGGACTGCTGATAGATCTATTGTCACTAAGTTTTTGCAAGCTCATCCGGCAGATTATTCAACTAAAAAGTTGCAGGAGATGATTCGTTTGATGAAAGAAAAGCGGTTGCCAGCTGCCTTCAAAAGTTACTTTAATTTTCACAAAGTGGATGCCATGTCTAAGGACAATATACATTTTAAGATGGTCATACATGTCCACAGTGATTCTGCGTTTAGGCGATACCAGAAAGAAATGAGGCACAATCCAGCTTTATGGCCATTATATCGAGGATTTTTTGTTGACATAAATTTATTTAAGGGAAACAAGCAAAACACTGATGAAGCTGGGAAAATCTTGAGTGATCTAAACAATTTAAATGTTAGTGATGATGCATCTGGAGGTGATTCACTTGCTGATGAGGATGCTAACTTAATGGTCAAATTGAAATTCCTCACTTACAAGCTACGAACTTTTTTGATCCGGAATGGCTTATCAATTCTGTTCAAAGATGGCCCTGCTGCTTATAAGGCATACTATTTGAGACAGATGAAGATTTGGGGCACCTCTGCAGCAAAGCAAAAAGAATTGAGCAAGCTGCTAGATGAATGGGCTGTTTACATAAGACGAAAGTGTGGCAACAAACAGCTGAGGTCTGATGTTTACCTCAGTGAAGCAGAGCCTTTCCTTGAACAGTATGCGAAACGGTCCCCTGCAAATCAAGCACTCATTGGTGCAGCTGGAAATTTAGTGAGATCTGAAGATTTCTTGGCTATTGTTGGAGCCATTGATGAAGAGGGTGACCTTGAGAAAGAGCGTGAGATAGAATCTACCCCATCATCTTTTAGCACTAAAGCAGCTGGGAGAAAAGAAGGCCTAATTGTGTTCTTTCCAGGAATACCGGGTTGTGCTAAGTCTGCACTTTGTAGGGAACTGCTGAATGCTTCAAGTGCTTTTGGAGATGATCGTCATGTCCAAAGTTTGATGGGTGATCTCAATAAAAAACGATACTGGCAGAAAGTTGCCGATCTGTGTAAAAGCAAGTCTGACTGCATAATGCTTGCTGACAAAAATGCACCAAATGAGGAAGTATGGAAACAGATTGAAGGCATGAGTCGATTTAGTGGTGCAGCTGCTGTTCCAGTTGTACCTGATTCTGAAGGCTCTGATTCAAATCCTTTTTCTTTGGATGCGTTATCTGTTTTTATGCTGCGTGTGATTCAGCGAGTTAATCATCCGGGGAATCTTGACAAGGCATCGCCAAATGCAGGCTATGTACTTCTTATGTTTTACCACCTGTATGAGGGAAAGAGCCGCAGAGAGTTTGAAGCCGAGTTGATTGAACGTTTTGGCTCAGTTGTGAAAATGCCTTTGCTCAAACCTGAAAGGGCTAGTTTACCTTTTCCTGTGAAATCTATGTTGGAGGAAGGGATAAATCTATACAAACTACATTCAAACCGGCATGGAAGATTGGAGCCAACTAAAGGGTCATATGCGAATGACTGGGTGAAGTGGGAGAAGCAGATGCGAGCGACTTTAATGAGTCATTTTGAGTATCTCAACTCGATTCAGGTTCCATTTGAGAGTGCTGTTAACAGTGTGTTAGAGCAGCTTAAAACAATCACGAAAGGCGACTATATCGCTCCCAGTACCGAAAAGAAGCGGCTTGGAGCAATTGTTTATGCTGCTGTCAGTTTGCCTATTACTGAAATTCGTCATGTCGTCAATGATATTGCTGTCAACAACTCCAAGGTGGAAGTTTTCTTCAATGACAAGAATTTGGCAGACACCCTTAGGAAGGCTCATGTCACTCTTGCTCATAAGAGAAGTCATGGTGTCATAGCAGTGGCTAACTATGGTCAATTCCTAAACCGTAAGGTTGATGTTGAATTGACTGCTTTGTTGCTATCTGATAACTTGGCTGCGTTCGAAGCACGTCTTGGCTCAGTTGATGGTGAAACGATCAATTCCAAGAACGAATGGCCTCATGTTACGTTGTGGACAGCACCAGGAGTTCCAGCCAAAGAAGCAAGTACCTTGCCCAGGTTGGTGTCAGAAGGGAAAGCATCTCGTTTTGAAATTGATCCACCTGCTGTTATAGACGGTGAAGTGGAATTCTTCTAGTTTATCTCCTTTTATATTAGTTTGATCAAAGAATTTGTTCTTTGAGCTTTTGCCTTGTGAATAGTTTTCTTCCTGCGGCGTACACATGTAGTGTGAATGTTGAGGGCTAGCTGAAATTTTTGGTAGGAATATGAAAGGTTTTGTATTTAGTTGAATCAATGGAATAAAGAAATTAAAGAATCTCTGGCCTGCTTTGGTACTTCCATGGTTCAAGAGATAGGGCGAGCCGAAGAAGCAGGTAAAAACTTTTAGAAAAGGGCAACAATGCCTGTAATTGAAGATAGATGGGTAGTTTGGCTAAAGCAACCATCTGTTTAAAACTTGTATTAATGTTGCAATTGACTCAAACGCAGCCAAGTCAAGGCACGTCTTACGTTCAAGTCAAGGCATATTGCTCCCAAAAACTATAGCAAGTTCATTGGATTTTCTTTCGAATCAACAATACTAGCTTTAGAAGCCATGTTCAGAGTACAAAAATGTGAATGCAACATTTCACAGTAGATGAGGTGTATAGGAATATAGGATTATGGATTTTAGTTGGTTTATGGCG

*S.maritima*43555

CGGCCATGATCCATAGATCTCAACTGCTCGAGCATAAATTCGACCACAAAAGGATCAGAGAGGTCCATATACACTTCAAAATCGAGCCAAAGCCACCATAACCAGAGTAAACTCCATGGCTTATGGTCCTCGTCAATCTATTTGTACTTCCACTTCCGAGTTCTACCAAACATAGTCTAAAAGACTCCCAACTTTTCACTAACTACCCTCGTTATCTGAATATACAAGGGATATGAAGAAGTAACAAGTTACTCAGATGACGGGGATATATACCGGATTGCCATCACTCACTGAAATCACCCGGAATCTGATATTCGTGCACTGTATGCAAACCAGTTAAAACTAAAGCAGATTCTTCAAATGAATAAACAGAAATGAGATAGAAAATGTTGATAAAATCCCTAACTTCCAACCAAAAACAGGATATTTGGTAAGCTCAAAGTGACATCAAAACAGAAGCTGTAAGAGCGCGACTGAGATCTTATTGACACCTGAAATTACATCCCAGCATTTCGCTAATGTTTCCCTATTTCCAAGTTCCAAGACTCCCAACGTTTGTATGTTTCCATAGAATCAGTCCGTCCTTAACATGAAACTACAGAATCAGATGTCATTATGCCAACACCGTTAATGATTAATCTGAGCACGTTCAGCCTCAGTAAATCCTTCTAACTATTGATAAAAAAAAATAATGGAAAACCAAATAAGGGAAATGTGATTTGGTGATGAGGTTCCAAAAATCTAATCCTAGTTGAATAATTTTAAAGCTAGGGGAAAACCTCCTGCACTTCACCATCAGCTAGAGCAACACCGAAATTTGCTTGAGCATCAGCATCAGAATTAAGTTCCCTTAAAACATGAGAGATCTTGAAAGAAGAGAACTGTTTCTTCAGTTTCGCCGCCTCTGCATGCAAGTTCGACATGTTTTCATTCTTGACTTTCCATAGACCCTGAAGCTGCATGCAGACAAGTTTGCTGTCACCCTGCGCTTCAATACCTGTATAACCCATCTCAAGAGCTTTCTTTAATCCTAAAATAATAGCTCGATACTCAGCAGCATTGCAGGTTGCAGTTCCCAACCCTTGACGTATTCTACAAATCAAGTTTCCACTAAGGGTTCGAAGCACCACTCCAGCACCAGCTTTTCCAGGATTTCCTTTTGAAGCACCATCAAAATGAAGAATACAGGTTTGAGTATCGGAGGGCACGTGGTCAGACTTCTGAAGCTTGCTCAATGGATCAGAAGTGGTTGAAAGCAACCCCACATCATCCTCATGAGGCCTCTTCTGTGGTAAATCTATGCTGGATGTTTCACCTGTGGCAGATGCTGGTTCCTAAAGAGCAATGGAAACACAT

*S.maritima*36986

TGATGATCAAATGCGGACTAATCAACAAGTTATATTGATTTTTTCAAGTGACGCAAAAATATATGCAGAATTATAGAAAACATGTATCAAACATATAGCAGAACTATAGGGTCATAATAACATATAGGCATATACTTTCTTTCAATCCAAAACACATTTGACAAAATATTACAGCCATAGAAATGGAGAGCTGAGGTCAATCTACCATCTAAGAGCGAGGCTACTATTCAGAGCTGCCACTAGTATACTTCTGAAACATATGTTACCCTTGCATTACTTGAGGCCCTTGGAAAGTTTAATAATTGCTTCACAAACTTCAGCTCATTGCTAGCCGCAAACTTTTTCTTGCAGGGAAGGGGAAGGATTACCATCTTTTCCAAGGTGACAGAATTTTTGAGCAAAAATTCCGCTAGTTGAAGCAGGCTAGGACAAGGTATTGTGTAGCCACGGATGTCAACAGTTTTCAGTTGATGCATCAAACAAGAAGGGAGCTCCGGGGACAGCGCATTGGTGTCATGTTTTCTATCCGAAATATGGTGTGAGTCATAGTACACTACAAGTTCTTCCAAGTGAGGTGAACTTCTCAAAACTCTACATATGCCTAAGAGTTGATCACCAAGAAGCAAGGACTTAATATGTAACCGTTTCCATCTTGTTTGAGGAAAATCTTGCTTCTTAAATATTGACATCTCAACCATGGCAAGTGATAATTTTATTAACGATGGCAAGTGGACTTGACGTAGCTCATCGATTCGACAACATTGAAGGTTCAGTGTAACAAGAAACTGACTTGTGAACACACGCATTGGCAAAATAGAACCGTCATTCTCAACAGTACTTGTAGTATCTCCGTCCACGAAATGTTGAACGATTAACTCCTTGACTTGTCTGTCTACTGCCACCCTTAACCATGTTAAAATTTCATGGACAACTCCATATTCACCAACATCGTGGAAATTAAGCTTAAATTTATCAATAGGGGTCTTTTTATGAACGATTAGCACATTGCGCACAAAATTGGCGAAACGAGAATTATACTGGTCGTAATTAGATATATCGGATTCAGACCAATCAGGCCAAAAGTCAGACTCATAAAATTCAAGGGAAGGAAGCGAAGTCCAAAGATCACCAAATCTTCGAATTAAGACGGTTCTAACAGCATCAACAATTGGCAAAAAGCTAAGAATGTGTACCAGAATTTCATCGGGTAATTCGCTTAACCTATCTCTCCCTGAAGAAACCCTTTGCTCTTTTGAAATGGAATTCATGATTTTTGAGGTGGTAGTTCTAATTGGTGTAGTTGAATTCCTGAGTAGTTAGTAATTTTGATTTTGGGTTAAATGTTGAACTGAAGTAAAGTAGATTAAGCGTTGGTCAAAGACCCATTGAAAATTGAAAATTGAAATTCTTCTGAGTTGCTTGAATACTTTCGCAAAACAAGAAATTGGAAGGGTTGTG

*S.maritima*44432

CTTCCCTCAACCAACGCAGAAGTGAAACCTAACACAAGGAAAAATAAAGCAAACTTTACTCATTCAACGAAGGCGGAAGAGAAGCCAGAAGGTCATCAAAAGAAATGGAGAAAATTACAAAGAGAAGAATCTGATGCATTACAAAGGGGAGCTCTTGATAAATTTTTTAAGAGAAGAGTAGGGGAGCCTTCGGATGAATTGGATCTTGACAATAGAGGAAGAAGAGTAAGCTCAGGCGATGATTCATTCAGCAAGAGGATGTCATGAATTCCAGCAATAACAATGGCGACGACAACAACATAGCAGTGATGGGTAGGAAGGAGAAAAGGAAGGCGATGAAGAAGATGAAAAGGAAGCAATTAAGGAAGGAGATAGCGATCAAGGATAAACAAGAGGAAGAAGCTAGATTAAATGATCCTCAAGAGCAAATTAAAATGGCTCGGTTGCAGCAAGAGGAGGCGGAGAGAATGGAGAAAGAGCGTATCGAGTTTGAGGAGAGAGAAAAACAGTTTCTTCAAGCTTTGGAGCTCAAGAAGTTGCAGGAGGAAGAACAACTCAAGCAATGCAAAGATTCTCTCAATATAATGGTTAATGAAGATAATGCTGAAGAGAAAAATGAAGATGATGGTTGGGAATATGTAGAAGAAGGGAGGGCAGAAATAATTTGGAAAGGAAATGAAATAATCGTTAAGAAGAAAAAGGTCAAAGTGCCCAAGAGATCTCTGGAAATTCAAAAAGAAAACAAGGAAAGTGAAAGACCAATCTCAAACCCTCTTGCCCCCCAATCTGAAGCCTATGAAAATCATAGGAACGCACAAGATATTCTACAAAGTGTTGCACAACAAGTTCCTAATTTTGGAACTGGCAGGATAAAGAACATTGCCCTTTCCATCTCAAAACTGGGGCATGTCGCTTTGGAACACGCTGTAGCAGAGTTCACTTCTATCCAGATAAAGCTTGTACATTACTGCTGAAAAATATGTACCATGGGCCAGGCCTTGCATGGGAGCAGGATGAAGGGCTTGAGGTCTGTTATTGAAGCCATTGGGTGGTTCCTCACCTGGGGAATTCTATAGGTTCTCAAGATGGATAACTGAAGCTTCAGCACATTACTATCATGATGGTTTTCTTTCCATTGCTTTCAGCCACACGTTGCATTGTCAGAACTTTTAAATTATATCTTAGAAAGTTGAAGTATACGGATGAGGAGGCTGAACAGTCTTTTGAAGACTTCTACGAGGATGTTCACACAGAATTTCTGAAATTTGGAGAAATTGTGAACTTCAAGGTGTGCAGAAATGGATCTTCCCATTTACGGGGTAATGTTTATGTTCATTATAAGTCACTGGAATCAGCTGTGTTCGCATACCAGTCAATTAATGGTCGCTATTTTGCTGGCAAGCAGCCAGCTCATAATCAAATGTTAGACCAAAGCTGTCACAACTGAATTGGTTGAAGGATTCTATTGCTAGAAAAATTTCAAAGCGTCTTCTACATCTTTTGATAATAGAGCATAAATTAGGGGCAAGCTCCCTATTCTCAATGTTGAAATACACAGTTAACCTGTGAATTTGTTAATGTGACAAGATGGAGGGTTGCTATCTGTGGGGAGTATATGAAGTCAAAGCTCAAGACTTGTTCTCGGGGATCAGCTTGCAATTTTATTCATTGTTTCCGAAATCCTTGTGGAGACTATGAATGGGCTGATTGGGACAAACCTGCCCCACGGTGCTGGCTGAAAAGTATGGCTGCTTTATTTGGATACAGTGGAAACAGATTCACAGATGGCGATAGTCCTGGCTATCACTCAAGAAGTTCAAGGTTCAGGGCTTTAGGCAGCACTCATGAAGATGATGGTCACAGGAGACGGAGCCTCTGGAGGCACAAAGATGATGACAGAAAAGCCAACAATTGTGCTGAAGTGATACAAGGAAGAAATGACATGCATCAAGATCATCATTTTACCAAGCACCGAAAAACTGATGATGGTGATAGATCGAATGGGAGCACTGGTGGTCAGAGATCTTTCATTGGCTCGAAAAGAAGCTCGAAGAAGAGGGGAAGAGAACCTGATCCAATTGAACAAGACAGCAAGTGTGGAGAAGGTGATGCTATATCTGCCAAAAATGATTCTGTAGAGAGAGGAGTAAGTGACAGATACCTTGGTCACAATAGTAGAAGCTCAAATCAGCAGAAGAGAGCTGCAGAGTCAGCTCATGAACATTGCCAGAAAAAAGATGATACACATGCGCAGGAGAATACTTGTGCTGAAAAGCATTATAACAGATTCCATAAACACAAAGGAAAATATTTCCTGCAGGAAAGGGAAGGGTTGGATTCCTCAAACATTGAGGCCCATCATGACAGTCTGGATAGCGAGGAAGATGAACACTATAGGCATAGTAGGAAAAACCGGAGAAGGATGAAGAATGAAGCAGATTTATCTAGCACTGCTTCTGATGAGGATGGGCTTTGCAGGGTTAAGGACAGGAGCCAAAAAACCAGGAGAAAAAGTTCAAAGTCTAAAAGCAAGAGAAGTGTCACAATTAGGAGCGATGAAGATGGTTTGGACTGTTGTGAAGATAGGCAACATAGATTTGTTGAGAAAAACTCGAGATTGAAAACAAATATCAAAGAGAGTTTTGATGGTAGAGATAGACTTGGTAGGGGTGAAAAGAGGCAAAGAAAAGTTTTAAGCCTAAAAGATCAGTCAGTCACAGACATGGAGAGTGACAGAGATGAGGCCAGGCGTCATAGACACCGTAAAAAGCACTCAAGACAGTAAACATGATGCAGACTCGTCAGAAAGTGCTTTTGAGAGTGATAGATCTGGCAGGTATGAGAGAAGGCCCCAAAAGAGAAGTACAAGTTTCAAGTCCTGTAAAGAGTCAGACGTTACTGACTTAGCGAGCAGTAGAGAAAAGGGTGTTGGGAAAGAGAGACGTCGTAGACATAGGAGTAGTGCCAAACATCATGTGAAGGTGCGTGGCTCGTCAAAAATTGATTCTAATGCAGATAGCCGGTTCAAGGTCAGAGACACGGCTGAAGACAAGGATGAAGCAGATGGTGGCAATTTTGATACTTATGTCAGATCCAGAAGTTCTTGTGCTGATCTAGACCAGAATCATCCTGTGCAGGAGAGTATTGATTATCAAGGAGGGATATTATCGAGATTTAGTGACACTGAGAAGTGCAATGACTCAATTGATGGGGTTTTGGTATGTCGAAGGGATATCAAAAGGTGTGGCAAAGATCATATGTTACAGAGTAATAAAAGCGATGAAGGGGCTAATGTGTCAACAGAGGCGGAGACTTGTTCAGTGCCCACCATTTACCAAGACTTTGATGCTGACACAGAGGCTGAACATGAAAAGGCATGCCGCCATGCGGCTTTAAAGAAATTGGAAGAGAAAAAAAAGAACATGGATAGTCAGGCTGTCCATGGCAGGGAGTCTGTGGAAAGCTTCAGATTTTGCTCCCATGAACATACAGTTGCAATGGAATCTGGTTCAAGTAAATTGGGAACAAATGTTGCTTCAATACAAAGGAGAAGGATTTAATTGTCATGCAAAGTGCTTCTATATCTATACAAAGTAAATGTGTCACGACTTGTAACAAGTAGTAGTTCTCAAAGCTAACTATGGTTTTGAACTTATCAGTGCTGTAGTTTCCTTTGAATGTTGTACATTTGTTTGTGTGTGTTTTGGTGCTTGAAAACCTGAAGCATCAAGCAGATAATCCCCGATAATGACTACCAGAGGACTTATCATAATTTAATTATTTGACATAGTTGCTAAATACAGCAGTTCTCTCTGTAGCAAAATAATAGAGAGCAAGTTTTAGAAGATTGGGAGTCTTTACTGAGTTTGGTAAGTAAGTGAGTGAGTAGTCCTTTAACTCAAAAGTTTGTTTTCAAGTTGATCATCGC

*S.maritima*19497

TGTGGTGGATGACTACACGGAATAATCACTTTGGTTTATGTAGCCGACCCCATTTTTGGGAATAAGGCTTGGTTTGATTGTTGATTGATTGAATCTTTGTACCGTAAGTATTATTATCTTACTACTTATAGAAAGATGACAGTCCATCACAAACCAAGCTTTATTCCCATAAATGCGGTTTGCTACATGATATAAAAGATGACAATGCTACATAAAATGTTGTTATAACTTGAAAACCCAAATCAGTACAAGTTATTGTTTTTTCAGGTTAACCCAAATCAGTGACAGCTAACAGGTATTGTTCTTAATTCCCCATAAACATAAGATGTGCGGCTAGGTCATCATAGTATCATATGACACTCTTCATTTCCTTACTGTCACTGTTTTTGTTGTCTAGTTTCACAGGTTGTGATTTTGTGGATGCAAAATCTGAAACAGCAAATGAAGCTTTTGGAAACCCCAACAACGATAGGTGAAAATGTGAAATTTTGTAAAGCTTGTTCTTCAGTCATATATTCTGCTGCTGAATTGAAAGTTGATGATTCTGCAACCAATAGAATAAATGATGAGCTTTTATATGATGAAATGGGGAGCAAGAAGCAACATCACAGCTTCTAAATGATAGACCATCATCAATATCATCTTGTTTTGAATGTGATGTTTACAACTCTTTTGCGCCATTGCGTTGCTGATGATGTGCAACTGTAATATTGCTGCACCTTGCACCTCTTCAACAATTCAATCGCACTCATGACTTTTGTAACTAGTTTTTGGTCCTTTTACATCAGATGAGCAAACTAATGCAGCAAATGGAGGGTTCAAGCCTAAAAAATGTTAGTATTCTTGTGCACTCTATTCTCTAACAATATCCCGGGATGGTGTTAGTTATGATTTTAAATCTCGGCTTCGGTCGCCATCACGATAACGACTGCGGAATCATATTCACGGGCACATAGTTTGCGGTTGTTGTGGCCAAATATCTTGGTTGCGATAAGCTCAAAAAGCTTTACAATGCAGTTGTGATGCACTGACGTGACCTTGATTTAATACCTTTAGTATTCTTGTGCACTCCATTCTCTAACTATAATATGGGATAGTATATTGATTTCC

*S.maritima*32700

GAGGAAAGAGTCATCAAGATTACAAACAGTAAACAGGAAATTATCAGCTATGAATAAATTGTTGATGGAAGAGAATGATCGTTTGCAGAAGCAAGTTTCTCAGTTGGTCTGTGAAAATGGGTTTATGCGCCAACAACTTCATACTGCACCAACTGCGGCCACTGATGGAAGCTGTGAGTCTGTGGTCACCACTTCTCAGCATTCTCTTAGAGACACCAATAACCCTGCTGGACTCCTATCTATTGCGGAGGAGACATTGGCAGAGTTCCTTTCTAAGGCGACTGGGACTGCTGTTGATTGGGTCCAGATGCCTGGGATGAAGCCTGGTCCGGATTCGGTTGGGATCTTTGCCATTTCACATCGCTGTAGCGGAGTGGCAGCCCGAGCCTGCGGTCTCGTTAGTTTAGAGCCTACTAAGATCGCCGAGATCCTTAAAGATCGTCCATCATGGTTCCGGGATTGCCGGAGCCTTGAAGTGTTTACTTTGTTTCCTGCTGGAAATGGGGGAACAATTGAACTTGTTTATACACAGATTTATGCTCCTACTACTTTGGCCCCTGCACGGGACTTTTGGACTCTGCGATATACAACAACTTTAGAAAATGGCAGTATTGTGGTTTGTGAGAGATCTCTATCTGGTTCTGGACCCAACACTGCTGCCCCTCACTTTATAAGAGCTGATATGCTTCCAAGTGGCTATCTGATTAGGCCATGTGAAGGTGGAGGTTCAATTATCCATATTGTAGACCATCTTAACCTTGAGGCGTGGAGCGTGCCAGAGGTTCTCCGGCCATTATACCAATCATCAAAAGTTGTGGCCCAAAAGATGACCATAGCAGCCTTACGCTACATACGACAAATTGCACAAGAGATGAGTGGGGAGGTTGTATATGGCTTGGATAGGCAACCTGCTGTTTTACGTACGTTCAGTCAAAGATTGAGCAGAGGTTTTAATGATGCTATAAATGGCTTCACTGATGATGGTTGGACTTTGGTGACCTGTGATGGTGCTGAAGATGTCGTAATTGCCTGTAATACTGCCAAGACATTCATGGCTACTTTAAATGCTACCAGTTCTCTCCCAATGATTGGTGGTGTTCTTTGTGCTAAGGCATCTATGCTACTACAGAATGTACCTCCAGCAGTGCTTATTCGATTCTTGAGAGAGCATCGTTCAGAGTGGGCTGATTTTAGTGTTGATGCCTATTCAGCAGCTGCTTTGAAGGCAAATGCATATGCATTTCCAGGAATGAGGGCTACCAGATTTACTGGTAGTCAAATCATCATGCCTCTTGGTCATACAATTGAGAATGAAGAGATGCTTGAGGTGATTCGGTTGGAAGGTCAATCTCTTACTCCGGAAGATGCTTATATTTCTAGAGACATCCATCTGTTACAGATATGCAGTGGAACAGATGAGAATGCAGTAGGAGCCTGCTCAGAACTTGTTTTTGCTCCTATTGATGAAATGTCTCTAGATGATGCTCCCTTGCTTCCTTCTGGGTTCCGTGTTATTCCTTTAGATTCAAAACCGGAAACTGCAACAAATCGAACACTTGACCTCACTTCGAGTCTAGAAGTGGGTACGTCCACCAACAATGGATCTGCTGATGCATCATCTTCTGGCAAGAATCGCTCTGTGTTGACAATAGCATTCCAGTTTCCATTTGAGAATAGCCTGCAAGAAAATGTTGCGACGATGGCCCGTCAATATGTTCGCAGCGTGATTTCTTCAATTCAAAGGGTTGCCACGGCAATATCACCTTCAGGGACAAATCCCGCAGTGGGTCCCAAATTGAGTCCAGGCTCTCCTGAGGCACTTACCTTGGCTCATTGGATATGTCAAAGTTACAGTTACCATATGGGAACAGAACTTCTTAGGACTTCATCTGCTGGTGATGATACAGTCTTGAAGCATCTATGGCATCACCAGGATGCTATTCTGTGCTGCTCATTGAAGGCTCTTCCGGTTCTCATCTTTGCCAACCAAGCAGGGCTTGATATGCTGGAGACGACTTTAGTGGCACTGCAGGATGTGACCTTAGATAAATTATTTGATGATACCGGGCGAAAATCATTATGCTCCGACTTTCCTAAGATAATGCAGCTGGGTTATGCTTGCTTACCTGCGGGAGTTTGCATGTCAACAATGGGCCGTCATGTTTCATATGATCAGGCAATAGCGTGGAAAGTATTTGCTGCAGATGAAACTACCGTTCATTGTCTTGCCTTCACTTTTGTCAATTGGTCTTTTGTGTGAAAGATACTTTAGGTAAAAATATTTAATCAGTTGCATCCTTAATTGTTTCTTTGCAAGATGAAAGAGCTAAAAGGATGATTGACAAATAGATTTGGGCAGTTAATGTGCAGATATTCAAGGGGGAAAAAGATTTTTTTGTAATATTTAACATGACAGGCTTGAATTTTATTAATTTATTTTCGAAAACCCATCGAATTTAGTTTAGCAATGTATCTGTTCATAGAAATTTGTTGTCCAAAGCAAGCAGCGATAGAAGATCAGTTACAGCGGATGTCTGAGAGAGACTGTTTTTGGCGCTCCCTTTGTTAGCGCTCATTTTTTATGTTTCATCTACAAAGAAG

*S.maritima*13778

CTTGTATTAATCTTGTTTCTTAATTCTGTATTCAACCTTCTATTTCTGCTGTTGCGATTCTTTCTCCCTAATTCAGCTGTAGGAGATTCCATGTCGACAGAGCTTTTACATGATGATCTACTTGCAGAGATTCTTATAAGGTTACCAGCAGAATCTTTGCTCAAGATCAAATGCGTTTGCAAGTCTTGGTATTCCCTTATCAGCAGTTCCAGGTTCATCTTGGCCCATGTCTGTCACAACAGATCGAAAAACCCTCGTATCCTTTTGCGAAGTTTTAACAAGGCAGACAAGAAGGTCAGTTATAAACTGTGCCATGATAACGAGTATTTGGATGGTATTATGACTATTGATCCCCCATTCATGAGGCAACGTAATGATTTTTTAAGGATGGTGGGTTGTATAAATGGACTTGTATGCTTGTCAGATGACACTGTTGAAGTGACGGATAGTGTGATATTATGGAATCCGGTGATTAGGAGATTTCTACCTCTTCCCAAACTTGAATTAAATGTTGACTCAACTGATTTGGGCCGATCAGTTTTTGGTTTCGGCTATGATTCTACTAACAATGATTACAAGGTTATCAAGATTGTATACCGTAAGAATCCAGATTTTGAAGCCCGCCAAGTTGAGGCTTCAATTGCAATTTATAGACTAAGCTCATGTTGTTGGGAAGTCAATGGGTCTGCTTCAGTCCCTTTGCTTGATTCCCGACAAGCTTATGTAAATGGAGTTATCCATTGGTTGGCTTATAATAAGCTAGTTGTGGGGTTTGCTGTGGAAAGTGAGGCATTTAGTGACACCATGCTGCCTGAAACCTTGCAAAATGCCAATATTAGTGATTTAGCAATCGCCTCATGGTGTGACTTGCTCTCTGTGTTTCAAAATGGGTTTTGGTCTGGCAGACTTTGTTTATGGGTTATGAAAGATTATGGCGTGGCTCAATCATGGGTTAAACAGTTTGTGATTGAATCCTACGTGATGGTGAGAAGTCTTAGAAGGAATGGCTGCGTTATACTCGAAAATATTGGTGGTAAGCTAGTTTTATACAACTCAAAGACCAATCAGTTTGAGGAGTTCAAAATCCATGTCGAGGGTTCTATAAGAGGTTTTCATATGAAATCATACGTAGAGAGCCTAGTTCTATCAGATCGGTTGGATGCAAAATCTATTCCCTAAAAATCGAGTATGTATACTTAACTTTAGTTTGCAATTAATTGCAGGATTCCCTCCCGTCTCCTCTCTTTTTCCGGTTTGTGCGTGGATGCATGTGCCTTTGTTTCCAATTGTGCTTGTAGATTATAGTTTCTCTATCCTTGTATAAATTCTGTATAAATTCCGGGATACTCATGAACCTAGAATTTCTCTTCTACCTTCGATGAGGTGAAACTCTGGAAGTAGTTAAACTGGGAGATTTTCAATTTCACTGACCCTGTATTACAAAAACTCATTTCATGCTGTAAGTATAAACTGTAATGCTCCAAATGTATCAGCTCACATTGCAAGTTAATCTGATGCTAGAATTCTGTTGTACATGTGATTGGATTGATGATTTCGGC

*S.maritima*44824

TTTTGTATAAACAAAAAAAAAAAACCCAAAAAAAAATTAAAAAATCCCAAAATTCATATGAAGAAGCAACCTTTTTTCCTGTAGAAGAAGGGGAAAAAAAAAGAAGATAAGAGGTTGAAATGTTGGTGCAGAAAAGAATAGATTTTATGGCACAACCTTTAAGCATCATGATTATGAGTGGAGATAATTTTTGCTCTGTTTCTTTCTTCTTCTTCAACCTTCTTCCATTCTTTCTTCTGTGAAATGGGATTTTAGCTTCTGAAACTATGATGCGAGCTATGCCCTACAATTTGCAAGGCAAGGGTGTGGTAGAGGTTTCAGGTTTAATTTCACAAATCTCTTCTTCAGTTCCTCCAAAGTGGAAAAATATAGACAAAATTCAACAACAAAATCAACAATTTGCAGCAACAACAAGAGCATCGTTTGAAGACGAAGAAGAGTTTGAAGAAGAATTACAAGTAGTTAGTAAAATCTCAGGTGTTAAAAGAAGTAGTTCAATTGACAGCGAACCCACATCTACTCTGGATACTACCACTAGGAGAAGCCCTAGTCCTCCTACTTCTACCTCAACTTCTACCCTTTCCTCATCTTTCAACAACAACAATACCAAAGATAATAGTATTGCTAACAATACTGCCGCAACAACAACAACAACAAGTGCTGGTGCGGCTGCGGCTGCGTGTGCCGGTGTGGCGAACCCTGGAGCATGTGTGAGGAAAGAGGATTGGGGTTCGGAGCTCCACCATCATCAAAGATCTGATGAGAATGAGAATGTTGCCACTGCCGCTGTCGCTACGGTTGGAGTTGGTGTTGGGGGTGATAGATTGGTGGAGAGTCAGAATACAACAAGTGGTGGATTGGAAGATTGGGATACAATGTTTCCAACAGGTGAAGGAGCTTTGCTTCCTTGGATCATAGGAGATGCTGAGGACCCTAGTTTGGGTTTGAAGCATCTTTTGCAATCTGCTTCTACTCATGTTGTTGATTATGAAGGGAATGCTGGTTTAGGTGTTGTTGATCAGGGTCCTGGTTTTGATGCTCATTCTCATAGTCATAGTCCTCTTGCTGTTGTTGGGTCTCAGGCGACCGAGGCCGGTTCCAATTCCGGGTTTCTTGGGTCTGAATTTGGGAATAATGGAAAGATTGGGAACTTGATTTCGCCGAATTGTTCAACTGGGGTTATGGATAATACAAAGGTTAGCTGTTCTAACAATGTGTCAAATAGTCTGCTTTTGGGTTCAATTCCTGCTAGTTTTTCTCAGCAATATGAGTTTGGTGATGAGAAGCCACAGATTTATAACCCACAGTTGGTGATGAACCAACAACAGGCTCAGAGCCTTGCTAACCCTAGCTTCTTGATGCCACCATTGGGGTACTGTCAGTTGGACCAACATTTGGGGTTTCAGCCACCCAATAAGCGGCATAACCCCGGCGTTGTTTTGGACCCGAATCTTGGTGTGAAGAACCCTTTTATTGATCAGGGTCATGACCTGTTGTTGAGGAAACAGCAGCAACATCAGGTTGGTGGGTATCAGCAGCTGCCCTTGGGGTTGCCACCGCAGCTGGTGCCTCCCCATCTGCAGCAGAAGCCGATGATGTCAACGAAGCAAAATAACCACCAGCATCAGCAGCAGCATTTCCCATTGCCTATGCTTCAGCAACAGCAGCAGCAAGAGCAGTTCATAAAAGAAACGCTCTATAAGGCGGCAGACCTGATACAAACTGGGAATTTCTCACTCGCGCAAGAGATATTGGCGCGGCTCAATCACCCGCTTTCCCTCCCTGCAAAGCCCCTCGATAGGGCGGCTTTGTATGTGAAGGAGGCTCTACAAATGCTCCTTATGATGGGCAACCCAGTTGCAGCTCCTCCGTCGTCTAAGAACCTCACCCCTTACGATGTTGTTCATAAGATGAATGCTTATAAGGTGTTTTCTGAGGTCTCTCCTATTACTCAATTTATGAATTTTACTTGCACACAGGCTATTCTTGAGGCTCTTGATGATTCTGATGCTATTCATATCATTGACTTTGATATTGGTTGTGGTGCTCAATGGGCTTCCTTTATTCAAGAGCTTCCCTTGAGGAAAAGGGGTGTTCCGTCCCTCAAAATTACTGCCATTGTTTCGCTTTCTACCACTCAACCCTTTGAAATCAGCCTCATTCGTGAAAATCTTGTGCAATTTGCTAATGATATTGGTGTTCCTTTCGAGCTTCAAGTTGTTAATTTAGACTCGTTTGATCCATCTTCATGCTCCATGCCTAATTTCAGAATCTCTGAGGAAGAGGCTATTGCTGTTAGTATCCCGGTTTGGTCATCATCCAATCGGCCTTCCATCCTCCCACCGGTCCTTGAATTTATTAAGCAGTGCTCCCCCAAAATCATCGTCTCTTTGGATAGAGGCTTTGATCGCTATGATGTTCCATTCCCCCAACATCTTGTTTATGCCCTTGACTCCTGCACCAATTTACTGGAGTCACTAGATGGTCATGTAGCCTCAGATATCGTAAGCAAGGTTGAAAAGTTTTTCGTCCAGCCTAGGATTGAGAACACCATATTGGGACGCGTCCATTTTTCTGAAAAGATGCCACATTGGAAGAATCTGTTTGCCTCAGCTGGCTTTTCGCCTTTGCAATTCAGCAATTTCACTGAAACTCAGGCGGATTACGTGGTGAAGAGAACTCCGGGGAGAGGATTTCACGTGGAGAAGAGGCAGGCGTCTTTGGTACTAAGTTGGCAGAGACGGGAACTTGTGGCAGCTTTGGCGTGGAGATGTTGAGGTGAACATGACGCCGTATCCATCAAGCTAGGAGGTTTGCTTCTCCATATTGGTCACATTGCATTTCCAGAGGTTTTTTTATAAATTATGTGATACATGATATTGTCAATAACTAGAAGCTATGGTGATGATGATGATGACACCTCGCTTTGTGGCTTTAAACTGTTTATTATCTCCTTGCTTGTGAGCATCCCTCACTCATCGTGCCTTTCAGGTCGGCGTATTATTTTGCTATGTACAACCAAACAATTAACTTCAAAATTCAGTAGCTTTTATGTCTTTCGACAATCTAATTGTTAAGTATCATATTTTTCTGTCCATGATTTGATTCACTGTCAAATTTGCTCAGCTGCTGAAAATGTCTTATTCACATGTCTGTTATCTATGTGATTACATCCGAGGATTAGAAATGAAGGAAACGATATTGTTGGTCCATGTATGCACTTACCGTGCTT

*S.maritima*38649

TTTCGATTACAACTTTGATTCAAGTTAGTTTACACACTTGTTTTAGTACAACTTAATTTGCGATGGAAATAACATACCATTACATTGCTAGATTCTTTAAAATTGTTCCATCCAAAAATAAAATTTATGGCAAAATATTACGTAGTACTTTAATTCTTGGCAATAAGTAATCAAAAAATCTCCAGTATATTCACAATTCACAAACCTTTCTCCTTGATTAGTTACTAGGAATTTGCTGCTACTGTAAAAGGATGAGCAGCTCGAACTTGATTCTCATGGAGCAAGCATACTGAAAGTCCTTCAATATTTCCAGAAAATATCAGACCATTGACGGACTCGTGTTTTATCCCAATTCGCTCTAAACGGAACCATTCCTATAATTCTAAACTCTGATTTCGCACACCCAGATTTTGGTGGGTGATATCGGTCAAAACTATTTTCTATGTACACTGGGTTTTACAATTCTATTACCATTAAGGTATCACATTCTCTCTACATGATCGCTGGTTTTTGCCGTGTCTTGCTGAATAGTACATCTCCACTCAGGACTGAGCCGATGAACTTCTCTACTTCTTCAGCGTTTAAACTCCCTGTATATGCTGCATACCTCCCTTTCCTTGGCTTGTAGGCTACCAAAAAGCTATTTGATGACTTGAATCCAGATTTTTCGAAAGCATCCAGGAAAGATTGTTGCTTTGCCGCATCTACCAGAGTGTATGAGATGGAATCCTTGGAATCAGAAAGACTTCTTCTCCTTGATAACGATTTCTGAGAAACCATTGACAAAATTGATTCAAGTTTGTCTTTTGCTTTTGATGATCTAAAACCACCAATTATGCAAACAGGGTTTTCATCTCCACAAAAAGCATGGTAGTTCGATGTTGTCAAAAGCGGTACTTTTTTGCTAGATGATTCACTCTCAGACTTCTTGGTCTGACTTGAAGCAATTTTGTTCTTCTTTTCAAAACTGCCAAGCAATGCACCAAGTTCCTGAACAGCTGACTCTAAATCTCTCACAGATATCCCAGCTTTTAGAATTTGTTTTTCACCATTTGACAACCATCCCACTATAGCTGGAAGTGCGTTAACTCCTAATCTCCTGACCTCTGGATCAGAAACATCATGAACCTGTGTGTCATAGAAGACAAATCGTTTGCGATACAAACCACTGAGAGTACGCCAGATCACTGGGGTATCTTTCTTTGTTGAAAGAAGCATGACTCGAGGTAACTTTTCCCCAGCACCAAACAAAGGCTGAAAGTTATCCAGGCTAACACGTCTTGAAAATCTCGGCAAATGTTCCTGACAGGAACTTTTCAAGCTTTTGACATTCAAGTCATCATTATACTCCACAAACGAACCACTCTCGCTAGTTATGTAGGAATATACAAACAATCTGGGTGCTCTACGAGGATGTATTCCATGTTTCTTGCAGAAAGAAGATTCATTCTCACAGTTTAATTTACCAACTTTCACAGCTCCATTCAATGAGTTATTAATCTCCTCCAACATTGATTCATACTGCTGGACTGTCATCATTGAGGGAGTGTAAGATAGCAGAAGCCAAGTAATGCCCTTTCCATACATCTCATTCTCAAATACTTGTGAACTCACAGCTCGAACAGACTTGGTGGGCCTCTTGAAACCAGATTGTGATCCAGATCCAGCAGAACCACCAAAACCACCAAAACGAGAACCACTGCCAGCATCACCACCAAAAAAGTTTGAAAAAATATCATTGATACCAAAACCAAATGGATTTCCACCACCAGATCCACCAAAGGAAAAGGAGTACGACCGACCACCCCCACCCATATTCTGCCACTGTCCTCCTGACCCACCACTTGTGAAGTGGGAGTAGCCACCATAATCTCCTGTACTCCCACCGTCAAAGGATGGACCACCTCTCTCATCACCATATTGGTCATAGTTTTTCCTCTTTTCTTCATCAGATAGAATTTCATAAGCATTATTGATCTCCTCGAATTTTGCTTGAGCACCTTTGTTCTTGTTCTTGTCAGGATGATATTGTAAAGAAAGCTTGTGGAAAGCCTTCTTAATATCACGTTGACTAGCATTCAGATCAACACCTAGAACCTTATAAGGGTCTAACCCCTTTTTAGCTTCAGCAAAAAGGATAATTAATGATACCACAGTAATTAGAACGAGCTTCAATCGGACACCCATTATTGAATTTTCCAGGACTTTTATGTGAATTTCAGTTTCTTTTCTTTAGTCAGCGGGAGAAAGTGAGGAACAACAGAAAATAAAAATAGAAGTTAAATAAAGTTTGGAGAAGATTGTGGAAAGTATAAA

*S.maritima*27747

TTTAAAGGTGATAAGAGATGCAAGAAGTGCTACTATAAGAAGGGATGATATAAACCTAGTTATATTACTTTCAAGTGCAACATCATCATCATCATCATCAAGTGCTGGAAGGAAAGCCAAAGTGTATTGGATCTGGGTTAATGAAGCAAGAAAGATCCGACCCATTTGGAGATGATGAAAATAATAGGGGTTCAAAAATGGCGAAAAGAGTTGAGGATTTGGGTTTGAGATCTGAGAATGCACTTCCAAAGCAGCAAATGCTAAGCTTTTCAACTTCTCCTAAACCCCAACAACTCAACTTTGTATGTGGTAAAGATGTTGAATTAATTGCTGATAAAAATGCCCAAAATCTTGTCTTATCTTATTTTCAGCAACCACCTCAATCTTCTTCTGCTTATTCCAGAACTCCTACTGGCTTTAATTCTGGAAACATGAATGGGAGCATGCATGGGTGTTTTACAGGGATCAGAGGACCATTTACACCAGCTCAATGGATTGAGTTGGAACATCAGGCTATGATTTACAAGTACTTAACTGCTAATGTACCTGTTCCTCCTAATTTGCTCCTTCCGATTCGAAAAGCCTTAAGCTCTTCTGGGTTTCCTGGCTTCTCTATTGGATCTTATTCTCCCCATTCATATGGATGGGGTGCTTTCCATCTAGGATTTTCTGGCAGCACTGATCCTGAACCTGGGAGGTGTCGCCGGACTGATGGAAAGAAATGGCGGTGCTCTAGGGACGCAGTTCCCGACCAGAAATATTGTGAAAGACATATCAACAGAGGTCGCCATCGTTCAAGAAAGCCTGTGGAAGGCCACACTGGCCAAGCTGCCTCTGGACCCACTAACACGAAGGTGGTTCCAGCGATTTCTGCTCCTATGTCGTCGCTGGTAACATCCAATGGTGGTGCCACCAACAGTGTCACGATTGCGCATCAACAACACAAGATCAAAGGGTTGCAGCAGCAAAGTGGTGCCACCAATACTAATGCTGATTCATTTGTTAACCGCTTCCATGATGAGCAAGGTCTCTCTGTGATGCCTCAAGCCATCAATTTGAAAACCAATGACAGCCCGTTTTCAATTGCGAAACAACACATTCCTCTGGATGAATCCTCTCAACCAGAATTTGGAATTGTATCTACTGACTCTCTCCTCAACCCTACGCAGAAAAGCGCATATTTGGGTTGTAAAAGCTACAATTCTCCTATATTGGAATTCAATGGTCAGCAAACTCAAGATCAATACCCACTTCGACATTTCATGGACACTTGGCCCAAAGATAATTCTAACAGGCCCACCATCCCATGGTCTGAAGACTTGAAATCAGATTGGACTCAGCTCTCAATGTCGATTCCCATGTCATCAGCTGAGTTCTCATCAAACTCTTCACCCAATCAAGAGAAAATCGCTACTGTTTCACCATTGAGTTTGTCTCGTGAGATTGACCCATCTCTGATGGGTTTGGGTGTGGGGACGATCATGGGTGAAACAGGTATGAAGCAAAATGCCTGGATACCAATTTCATGGGGGAATACTACAATGGGAGGTCCTCTAGGGGAGGTACTTAATAATACAAGTAATGTGACACCAGCCTTAAATCTCAAGGGTGAAGGGTGTGATGGGAGCCCTCAGTTTGGGTCATCCCCTACTGGTGTTCTGCAAAAGTCTACTTTTGTTTCTGTTTCAAATAGTAGCTCTGCTGGTAGTCCGAGGGGTGAAAATAAGAAAGCCCCCGAGAGAGCTAGTCTATGCGAAGATATCCTTGGTTCAGCAGCTCATGCTAGTTCTACATTCATCCCTTCAATGTAAACTATCGGAAGAGCTCGACGAGGTAAGAGAGGAGAGTGATTCCGGAGATTATGTGACTTTCTTCATATTTATGAAAAAAAAATGTTCGAAAACAAGTCTTACTGGTAAGGCCTGTTTAAGAATTAAAATATGCTTAATCTGTTATTTTAATTGAGTTTTGCTGAACAGTTGTGGGAGGTTCAAAACTTAGAGCAGGTGTTTATAGCTCAACCTATTTTTTGGCTATGAGTTGTGAACTTGTGACGACCTGTTCGTCGAATGATCACAGTGTATTGTTTGGCTGTTGTCATTACTCTGTATCTATCAATGACCATTTTGTATCTCCTTCATTCTCCATTTGGAGGGTTAATCCAAACTTTCAAGAATTTTAATTGAGAGAGAGTTGGATTTGAAATCAAAGCAATTTCATCTTGGACTA

*S.maritima*36182

ATGTGATGTGTTCTTGTTACATGAACTTCCAAAAAATGTTTTTACATTGTAGAAGAAACAAATTTGTGCATATAAATTCAAGAAACGTGTGGAATACTGGAATTCTATTTCATTACGTAGATGAGAGATACCATAATTAGTTATTTTTGTCATTCATATGATCATATGTATGTACAAATTGTAACATTGTTTTTCTACATGACCAATAATTTACATTGGTATAAACGATTACGGCATATAATCAGTATTCATCGTGCTCACTAAGCCGCTTTGCAAGAACATCAACTGGCAGTGAGCCTAGAGGACCTGGGTTTTCGGCATCCTCAAATGGGTCATCCTCAGAATCTACTTCAAACTCGTCTTCTTCATCGGTGGAATAATTATCAAACTCTTCGTCATCTTTGAAAAATGCGTCTTTTGATTCTGAATTCGACATGCTACCCTCACCTGAATGAACTAGAGCAGGATACGAGTGCTCAGTAGCTTGTGTCATCCTGTCGAATTCCTCCACCCTTTCTAGAAGCTCGTCTGGACCCAGATCAGTCTCGTCTAATATAGCTGCTTTGTTTGACTCTAGTGCGCGTTGCCATAGTGACATCATTCTTGGGCTTGAGATCACTTTTTTAGTTTCACCTGAATTAGAGTTGTCAAAACCATTTACAGAAGCACTTGAAGTCAACAAAGATAGAGTGTCTGGGTACATAGACTTCCACTCATGTCCCCTCCACATCAGTATCTGTTCATCGTCAAAAGACAATAGTACACAGGGAACCAGCTCCTTTAGCTTTGCTCCAATCTTTTTGTAGTCACTAGGTTCTAATCCTCGACAATCAATTTTGACAAGTTCACTTCCTTCAAAGGCTTGTCTCACTTCATTTACTAGATTGATGTAGACTCCATTTTTTGCTAGTTTACATATGGCCAGAAGGCTTTTCCCTTTTTGTCTAAACTCATCAGCCTTCTGTTTAGTCAAGCCTTCAGGAGCATCTTGAATAAGTTTTGGATAAACCGGGGCAGCTGGCTTCCAAAGCATTATAGGATATTTTGGGCGAGTGCGGTAGTTATAATTCCGGCCACGGAAAAGGTATGCTATACCACCAGTACGATAGATAATCTTACCCCCTGCTTTCTCCTCGATACATCTGCAGACATTATCCATGTCAACAGTTGGAACACCCTTGCATTTCACTTTACACACCGGCCGTCTCTTCCAATGAGAATGTATCAACTCCAACATATTGTGTGTTAGCCCATCTCTCCCAAGATTAACTTGACGATTATCCTTGATCATAGGTTGAACAAGAGCCTTAATCTCAGCTCTAGACAAAGGTTCCCCAAGAATCTCCTTTCTACTCCTTCCATCACTAGAATACTCCCCAAGTTTCCAAGGACTTTTCATATCAAACTTCTTAACATAAGGATTAGAAGAATCAAACAAAGGAACATTCCTCTTTTTCAGCAATTTCTCAGCTTTACTCTTCAGTGGAGCTTTACCAGTCCAAGGACGGGGCATAGAAGGAGGAGCAAAGGGCAAAAAGGGGGGTTCCCTAATAGCTAAAGGTTTGGCTTTCGGGGTTTCGGAGTAACTGAATTTGAACTCAAATGGAGCTCCGGGGAGAAGGTAGGAAACCCCGGAATCACCAATGACAATGGTTCGGTCATCATCGGATTGGGATTCAAAATCTTCTTGAACGGTGGAAGGAGGGGAGATGAATTGTGGGGTGGTGGGAGAAATGGGTTTGTAGTATTTGGTTTTGTTGTGGGGGATTTTGGTGAAAGCAGGGTGGTTTTGGGGAGATGGGGTTTTGGAATTTTTGGGTTTAGGAGGGTATTTGGGAGGTGGGATTGGAGGAGAGAAGGAAGGTGGTGATTGTTGAGTGGTGGTTTTGGGGGGTTTTGGTAATGAGGTGAAGAGGTTGTGGTTTGGAAGTGAAGCTACAATTGCCATTGAAGAAGATTTGAAGATGATTGTAAGAAGTTGTGGTTGTATCTTGTTTGTTAAGTGCCTTTTTTGTTT

*S.maritima*467708

CCAAAGATTGGGATAAAGCAAGTAAGGCGAAGAGATGTTTGCAAGAAAAAGAGAGAGAACTTGCGAAGCAAAGGAAATCTCTAGGAGAAGATTGGAAACCTAAGCATTTTAATTTATCTCATGACCCTGAATTTGGGTGGGATTGTTCACCTAAACAAAAGACCGTCCAACCCGCTCCAATTTATTTTCCGATTTAAGTTATTATGATTTTCGTTATGTCTTAGTATTATTATTGCGCCATTTTACAAAAACTGGACTCATAATGTTGCAAAGAAAAGAAGATAGGTAGCTAAAAGAAGTAGTGTATGAACTTAATATTTGTATGTACCGTCCATTATATAGCTAGTGTTGCATTGATGATCGGAAAAAAAATGTAGACATTAGACAACGTCGATATAGGCTGGCATGAGAAGAAGAGAGCCAAATGTAGCCAAGGACAACCCGCCGGAAAGCGAACAACGATCCAATCCCTAGCCTTAAGCAGTTAAGCTTGATTGCATTGTTATTACTAAGCCTTCGGCAAGTCATCCTTGGCTGCAATTCCACTCTCCATACTTCTTCATGTCAAACTCCTATATAGACCAATTTCATTTTTATTTTTTTT

*S.maritima*27444

ATTCATTCCTTCATCAAAAATTCAAAATTTTTTTTCCCGTTGCACTCATCCTTTCTTCTTCGATCATTCACCTCTTCTTCAATTTTGAATTTTCAAAATTTGAATTTAAATTACTGCTTTAATCTTCTTCTTCCTCCTCCTCCTTCTTCTCCTTCTTCTTCTACTTCTTCTACTTCTTCTTCTACTTCTTCTTCTTCTTCAAGTATTGTTAATTGGGTTGAACTTTTGGTATGGTAATCATCTTCCCGAATTTGTTCCTCGGTGGAAATTTGCTTCGTGAGTTGCCTTTTAATTTCTTGTTTTTCAAAGTAGCTTTTTTTTTTTTATTGAATATATTGAAGTAAATTATTAGAATTTTTTATTTTTTTGTTGTTTTTGTTACTCGTTATTTGCGCCACTCAATCAATCATGATTAGCAATTTAGTCTCTGTTTAAAAATTTACAATCAAACTTTTTCATTCAAATTATTATTTTCTGGTTTCAAATTATAATTATTCTGTTTTTTTTTTTTGGCGATATTAATAATATTATATTTTCGTTGGAATAAATACTAATCTCCTTCATTTGAGGAAAAAAAACAAAAATCAACCAAATTTGTGTTATTTGTGGATTTGAGACTTGGAACTTTTCTTTTAACAATGGCTTCCCTAAACTTAGTTGGAATGACAAAGAACCCATCAATTTTCCTCATCAAAAACCCAGCAAAATCGTACAAAACTCCAAACTTTGCTACTTTTGGTGAGAAAAAACACAGTTTTGTGAAGTGGATCAATCTCAAAAGGGAGATTAGAGCATCAGCTACAAATGGGTCTCCAGGACTTTACTCCGCTAAGCAATTTGAGCTCTCAATCAAAAACGTTGACATGATTTTGGAAGAAGTAAGGCCTTATCTAATTGCTGATGGTGGAAATGTGGATGTTGTTTCTGTTGATGATGGAGTTATTACTCTCCAACTTCAAGGAGCATGTGGGACTTGTCCGAGCTCAACAACCACCATGAAAATGGGTATTGAACGAGTGTTGAAGGAGAAGTTTGGCGATGCAATAAAGGATATCCGACAAGTCAATGAGCAAGTAAATGAAACAACTGTTGAGGCCGTGAACAGTCATCTAGACATACTTAGACCTGCTATTAAGAATTATGGTGGAGAGGTGGAAGTATTAACCATCCAGGGTGAAGATTGCACAGTAAAATATGACGGACCAGAGACTATTGGCTCTGGAATAAAAGCAGCTATCAAGGAGAGGTTTCCCGACATTTCAAATGTAATCTTTACTAGCTAGGCGATGCTCAATTGCTCACCTTTCTGAGTTCACTAGCTCAGTATAGTATACACGAGTTAGTTGTAGGGTTTGCATTGTGTATGGGATACAATGGTACATTCTTGTATAATTTTCAAACCTCCTTAAATTCATGTAGTTATATAATGATTGCTTCAAAACTGTATGCATACATCGTACTCTTATAGATAAACAATGTTTCCTTGGTTCAACAAGGTGATACATTCATCCACTGGAAAAAAAATTTTGACATCCAAACTCTGTGGACCTGTTATGTTCTTCTGGCTTCCACATGCAACTGCATAATGTATTCATGCCCCCTTTGCATCTCTAGCAAGAACTGATTTCAGCCATCGCGAAAAATTGATCAAAAAAGGAGTGTATATGAAATAGCAAGTAATGGTCCAGCTGACAATTCCCTCGCCAAAGACTTGTAGGTTTGCTTGAGGATAGTACCATAGATGGAAAAATTTGATGAGTGGTATCTCAGCCAGTGGACAAGCAATGCCTACAATGCAGGCAAGGGCAAACCCAAACCATGTTCTATCAGCGAGAAACCATATGAACTCTGCTGCTGCAAACAACGCATAGGCTTCAATATTGGCAGGTACGCCTGATTTATACATCTCAGCACTCAATTCTATGAACAGCACCAATGATATTAATGAGATAGCTGTTTTCTTGAAGCTTCCTTCTGGAGTTGTATTTGGGGATGCTCTTTCATCCAAGTAGAGTTGTAATATTCCAACAGTGCAGTAGAATAGTCCTAGGAGAAAAGGTACCCATATATTGGTATGCAAAGGACCAATGTTGATGGAACCGCTCTTGTACACCACAAGGTTAACCCGCGAATGGAGACCATCGATAAGGGGACCTAAGACGAAGCCTGAACCAAATAGCGAGAGAGCAACTGATGGCCATGCTGTTTTAACTCTTGCAGTGTCCTGCTTCTTCAGGCTGCAACACCAGGGTTTACTCGCGACATTGACTGATCTCCTTTGTGGAATTCTATGGCTGCGTAGAGATGGCAGGCCACTAGCACAAGGAGCTATGTGATGTTGTATTGCTGCTTTATACATTTCATACTCAGATTCTCAACAATGTATTGAAGTGTCGATGTTGAGACACTTCTATTTGTCTGTGGTTTCAAAGTGCAGATATTAAT

*S.maritima*37419

AAAGTCTAAACCGTCAAAATGGCGCCACCTAGTTCGTTAAAGTATACCATGGTCCAATTTCCTCAAAATTGACTCGTTCTTCAAACCACCATCAATGGAGACCACAATCTTTCACTAAATCACTAATCACCCATTTCACCCTTTGATTTTCCATTAAAAAAAACAATAAACCCCCATTTGAATCAACGACTTCAAACATCGCAATTTAGGTCAACATTATGTCAATTTCATTTTTTATTTTTAACATGAGGAATTTTTTTCTGGAGTACTTTTTCAGTTGACAACTAAATCCAACCAATTTTTGAGTAGTAATTACAAAATTGAAGATTCTGATTAGATCCCCAAAATCAATTTTAAAGGGTTTTGTTTAGTATCAGAGGAGAGAGAAGGAAGGTGAATATAATTGCAAAAGATGGCTGTTTGGCAAGCTGCAACATTGAGTGGAATTGTAGCATGGATTTTGGTATCATCAATTTTGGAAATTACTCTAAAGATTAGATCTTTTTTACAACCATGGGTGATTCATCATGTTCAAACTAGCATTCCTCTCATTCTTAAGATCCAGAAATCACAGCACAAATTCTTGGATTATTTGTTTTCTGTGGTCTCATGTGTTGTTTCAGTGCCATTCTACACTTGTTTCCTTCCTCTACTTTTTTGGAGTGGTCATGGAAAGTTGGCTAGACAAATGACACTTTTAATGGCATTTTGTGATTATGTTGGCAATTGTGTGAAGGATGTAATTTCTGCTCCAAGACCAAGTTGCCCACCTGTAAGGAGATTGACTGCTACTAAAGATGAAGAAGAGAATGCACAAGAATACGGGTTACCTTCATCCCACACCCTCAACACAGTCTGCTTATCTGGATACCTTCTGTATTATGTTCTTTCCTTTAATCCAAGGAGTAATGGACCTGAAGTATTGGTTGGCGTTGTTTTGGTTTGCTTGTTTGTTGGCCTTATAGCCACAGGAAGAATCTACCTGGGCATGCACAGTTTGATTGATATTGTTGGTGGGATTGTCGTGGGAGTGGCTATCCTTGCATTTTGGCTCACAATCCATGATTATGTTGATACGTTTGTAACCTCTGGGAAAAATGTTACAACATTTTGGGCTGCTCTTAGCTGCTTATTGTTTTTTGCTTATCCAACACCCGAGGTTCCAACACCAAGCTTTGAGTTCCATACTGCCTTCAATGGTGTTGCATTTGGAATTGTTGCTGGCGTACAACAAACATATCACCAGTTTCACCACGAGAATGTGCCCCGCTTCTTCACTCCTGAACTTGGAACTCCTGCCTTTATTGGCAGGATTTTAGTAGGTATTCCAACCATCCTACTGGTGAAGTACTGCAGTAAGGCCTTGGCAAAGTGGATTTTGCCCGTGGCATTGAACACAATGGGCGTCCCTATAAGGTCAACTACTTACCTTCCTGCTCTTAATGAACCAGCAACTGGTAAAATGTCGAACAAAACGAAGCAGCAATCTGGGTACCTTCATAAATTTTTCTTTTCATCTGGTGAGGATTCTTTTGATATTGATACAGGGATAAGATTTATCCAATATGCTGGCCTCGCATGGTCTGTGGTTGATCTTATACCATCACTATTTTCAAAACTCGGATTGTGATTAGTAAATTTTTGACATGATTCGGTAGAGCATCTATTGTTGTTACCCCCTTGCTTAGCGAAAAGTTTATCTGAGTATGTAATTCCTAATTATTGCTCGTATATATATTCCCTTGATCGCCTCTCATTACTTACTCGTATAGCTATCTATATGTTCC

*S.maritima*347871

TCTAAAACAAACTTATCTCAGGTACTATTTTTTACTTTCTCTTGCTTAATTTCAATTCAATTCTATTTTTTAGTTTTGCATTGTTATTAATTTCTAATAAGAAATCAAATACAAGATAGAAAATGGAGGGATATAATCATGATGATGATCAACAAAGAGGTGGATCACTTCAAGCTAGTACTATTGTGTACTGCCAAGCAGACAATTGCACGGCTGACTTGACCGAGGCTAAGCGATATCATCGTCGTCACAAGGTTTGTGAGTTCCATGCCAAAGCTCCGGTGGTCATCGTTAACTCGATTCATCAACGATTTTGCCAACAATGTAGCAAGTTCCATGAACTATCAGAGTTTGATGACACAAAGAGAAGCTGCAGGAGGAGGCTAGCAGGGCACAACGAAAGGCGACGGAAGAACTCCTTTGATGTTTCTGCAGAGAGCTCTTCAGGCGGCTGAGTGAGGCCACCGGAGAAAGAGAGATAAACAGCGCAATCAGAGAAAGAATATGGATTGATTATTAGAGATAAAACCATAGATTAACCCCCTTGAATTGCTTGAATTGAGATAAGCTAATTAACCTTGATTGATGAGTTGATCATATTATAATGAAGGTGTAATTAATGGTTTTATCACTAATAATGATCCATTATTATTCTTCTGAATGCTCTCTATCTTCTGTCAAACTCATGC

*S.maritima*95321

TTGTCAGCAATGTAGCAGGTTTCATTTGCTGGGTGAATTTGATGATAGTAAGCGAAGCTGTCGTAAACGACTTGCAGGCCACAATGAAAGGCGGAGGAAACCTCAAGTGGGCTTAAGTAGTAGAAGTGGGAGATCATTTCACTCATACACGGGAAGCAAGTTTCAGGGGTTTACACCGTCAGCATCTTATATTTGTCGAGACATTCTTTTGAGAGCCACAACCTCTGCAGAAAAATATGGTTCAAATGATTGGATTAAACATGTAAAGCTTGAAAATGTGACAGATTTTGTTCAAGAACAAGCGTATGATTGCATATATGGACAGCTTCAACCAAAATCTAATTTTCCCTCTTACCATTTTGAGAAACATTTCCCATTTGCTGACAACAAAGATGACACTGTCTCAAAAAGTCTTGTTCACGGGAATGTCACTCAACACGTACCTCAAACAGTTTCACCAAAAAGTGAAGACTTGACACTTTTGGACGCAGCATCAACCGTTAATGGATTACCAAGAACATCGGAGTCCGGTTGTGCTCTCTCTCTTCTGTCATCTCAACCACAGAATTCTTCAGGTCATTCATCACAAATGCCGGAACCTGGAGTTTTAATGATACCAAGCACTAGTCCACACTACAGTATTAATGAAGACTCTGAAAAAGTCTTTGGAGTTCAAAACAAAAATGCTTTGTGCATGATCAGTTCTATGGGAAGAAATCAACCAAGTTCAATGTTGACATATCTTAGTGATAATACCTCACATTCTGACATTGGAAATGCGATGCACCACAGTTCCAAATTTATGAACATCAAAGATCAATTGTCGTGTGATATTGGGACAACTATTGATTTGCTTCAATTGTCATCTCAGTTGCAGAGAGTGGAAAACCAGTGGCAACCTCACCCTCTCAAGCAGGAAACTGAAGGTTCTTGCTGCCTAAGAATGACATGAGGGACCATACAAGAGGATAATAATAATATGAATGCGGGCAAGGGGCAACAACCATCAGAGAACTTGGACCCTGCTAATGCAATCTTCACTACCGCTCAGCTAACTGCTTAGTACTTGTAGGCTTGTAGCTCATTTGTGGAGACGGATTAATATCTCTGCTCTGGAACTAGTATATATGTCCTAAGCTTTATAGAAATTTTTTATTCTATGATATTAGCTCATTTTTAGCCTTGAAAATTCTGAAATTTTGAAGAGCCTGATGCTACATTTGACTGGCGTTTATCTTCATCCTAATATTTGCAATCGTATATTCTTGTTTT

*S.maritima*700613

GGCAATGGCTGCTCTCTGTTTTTTCTCAATTCTATTGATTGATTAAATATGCACTCTTTGAACTTTCACATTAATCGAAGTATATGTTATGTTTTTTGGAATGTTCAGATGGCTGATTGGTCGATGCTTCCATGTGAACTTCTTGGTGCAATTGCTCTAAAGTTGGACACACTTGAAGACTTCATATACTTCTCTACCGTATGTCATTCATGGAATTATGCTTCTTCCTTTGTTAAACGTGAGCGAAATGCCACCAACATGATGCCTTGGCTACTGCTAGGCGAGAACACTAATGACAACCCTACTCACCTCAGGAAAATATACAATCTCACCAACAATAAGTGTTACCAGTTGAGTATGCCCGAGACCTTTGGGGCGCGCTGCTGGGGTTCATCCTATGGCTGGATTGTCATCCTAACAATGGACTTCCAGGTGCAATTGTTTAATCCATTAACCAAACAGCGACTGAGCTTACGGTCAATACTAAATCTGCCAAAATTTCCATTAAACCCTGATGAAATAGATGATCCTGACCGATATTGGAGTTGGCGTGTCATAAATTTTGTGGTATTAAAGATGACTTCAACCAATGACTTGCTTGTTGCCGT

*S.maritima*34755

GCATCTGCTTATTTGAAACGCAGCAACAAAGTACGGACTTTGTCGTACATCACCTTCGATCTCTCCCTTCAGAGCCACCATTGATCTACCTTGCTGTTCTTTATCTCCTGTTGAATAAGCAAGGTGGTGAAACAGAAAGGGGAGAAATGGAAGGATTGGACACAACGACAAAATCGACATTGACAAAAATCCCCCTTTTGACGATAAAAGCAGGACCTCGAGATGGTGCAGCATGGACACAGCGCTTAAAGGAGGAGTACAAAGCTTTGATTGCATACACTTCCATGAACAAATCCAATGACAATGATTGGTTTAGAATTTCAGCTGCTAATCCTGAGGGAACTCGTTGGACTGGCAAGTGTTGGTATATTCATAACCTTTTAAAGTATGAATTTGATCTCCAGTTCGATATTCCTGTCACTTACCCGGCTACTGCTCCTGAACTTGAACTCCCCCAACTTGATGGCAAGACCCAGAAGATGTATAGAGGAGGGAAGATTTGCTTGACTGTGCATTTCAAGCCACTTTGGGCTAAAAATTGTCCTCGGTTTGGCATAGCGCATGCCCTATGTCTGGGGCTTGCTCCATGGCTTGCAGCAGAAATTCCCGTTCTTGTGGATTCTGGAATGGTCAAGCACAAGGATGATGATGTTGCCACTTCCAGTGACAGCTAGTTAGTCTAAAGTCTCATGCTACAAAATTGGATTAGGTAGAGGAATGAAGAAGTAAACTAATTATATGTTTTTCTTTCAGATAAATGTTGAGGCACTAAAATGTATTTGTGTTCTTCTAATGCGCTACTGTTGAGAGTTATCAACAAATGCAGTTCTCATTCTTGTTGCTACTGTCCATTGTAAGTCTGCAGCACTCTTGCTTCCCTCCATGACTTTTGCTCTTGTGCAGTTTGAGTTTGTGGTTTTGGACTCGGAAGTCCAAGGGATTTTGTCAATAAATTCCAATCTATTTAGACAAGTTCAAGTTAAAGCTCTAAGAGCATCTACAATGGCAAGCTAATTTGATAATTAGCTTGCCATGC

*S.maritima*15309

TCATCCCCCCAGAAAAAACCCAAAAAAAATTGCTCACTCACTCACTTATCTCCCCTTTCTCTCTCTTCTCAACTTCAACTTGTGTTTGTCCACTGATTTCTCTCAGATCTAGGGTTTCGTTCTGAAACCCTAGAATTTCCCCCCTTTCAGTTTTTCCATATTTACTTTTAACTTCCCTCTTTCTTCGCTCGTCAGCAATGGCGACTCCATTCATCACTGCTCCTCAGGTGGGTTCATACTTTGTAACTCAGTATTATTCGATGCTTCAACAAAGACCAGAGTTTGTTCATCAGCTTTACTCTGATTCTAGCACTATGCTTCGTATTGATGGCAACACTCGTGAGGCTGCCACTGCAATGCTGCAAATTCATGCGCTTGTTATGTCGATGCGTTTTACGGCGATTGAGATAAAAACGGCACATTCTCTTGAATCTTGGAATGGTGGAGTAGTTGTGATGGTCACAGGGTCTCTTCAGATCAGGGATTTTAGTGGGAAAAAGAAGTTTGCTCAGACTTTTTTTTTGGCTCCTCAGGAGAAGGGTTTTTTTGTTCTCAATGATATTTTTCACATCATTGAGGAGGATCAAGTTCATCCATATCCAGCTTCACTTATAGGGCAAACCAATACTAATGTAAAGTTGAATGCTCCTTCATCCATACCAGAGGCAGTGCCAAACTACATGATGAGTGGAGCAATGCAGGCCAGGGAATACATGCCTCCTGCTGACGTCAAAGAAAACGGTACTGTTGACAAGTTTTCAATGCCTGAGCAAAGATTGCAGCAAGCTCCTGTAGTTGAAAGTATCCTGGAAGATAATTCTCGAGAACAGACAAATGGTTTTCTTGAGAGCCCGATGAACCCTATACAAGAAATCCCACCAGCACAGATAGAGGAGCCTGTTGAGGAGCCACACAAGCACACCTATGCTTCTGTTTTGCGTGCTGCCAAAGCACAGTCTGCATCATCTGTTGCGCCTTCTACTTCGTCTAACAAGGCTGTGGCTCCTGCTTCAGAATGGCAGCATTCTCCACAACAGAATGATCAGCAATCTGCTGCCCAACAATCCCAACAACAGTCTAATTCGGCACAGGTTGCAAATGAAAGCTGGGCACCAGATGTGGGTGATGACGTTTCAGCTGCTGATGATAGAGGTGAAATAAAGTCTGTCTACGTGCGAAACTTGTCTCCAACTATATCAGCTTCTGAAATAGAAGAGGAGTTCGTGAAATTTGGTCAACTTACTGCTGAAGGGGTAGCCATCCGAAGACTGAAGGACACTGATGTTTGTTATGCATTCGTTGAATTCGAAGATATTGCTTCTGTCCAGAGTGCAATTAAGGCTGGTTCAGTACAAATAGCTGGACGTCAAATATACATAGAAGAGAGAAGAGCAAACAGCAGCTTTGCACGTGGAAGGAGAGGGAGAGGCAGGGCTTCATATCAACCTGATATTTCGAGGGGAAGGTTTGGTGGTCGTTCTTATAGCAGGGGTGGTGGTCAAGTTGGAGGTGAGTTCAACAGACCGAGAGGAAATGGCTATTATAGGCAAACAAGCCGCCAAGACAAAGGGTATTCAGGGAACCAAGTATGATGAAATGGATAAACCATACCAGAGTGAAAATTAGGGTGACGGTGAAAAGAAATAGTGAAAATGTTAGTAATTCAGTTTAGTTTTGGCAAGTTAAAAACATAAATATATATATTCAATATTTTTGCAGTTGTAGCATTTGAAGAAATGCATTCTTTGTTAGTGGCTTCTGTGCATTTTTTTTGGTCGGTTTTCGCCATTCTGCTCACTTTTTTTTGTCAAGAGCAAAAATGCAAAATTGTAACTTGAATGCCATCTTCTGTCAAAGCATTCATTTTCATCAACCTTTGTTGAACTTATTGCATAAAGGTGTGCTCTGATGACATTGTAGTACCTAAACTGAATTGTGATGGGACCAAACATGAACTGTATTGGAC

*S.maritima*29157

TAATGTTATTTTAATTCGCCGCAAGTATTCAATTATTTCAAATCAAATCATCATCTTTGGCGGTGGGTGAATGGATGGGTGGGTGAGTGAGTGAGTACTTAATTTCCGGTTTCCATATCTACCTTCAGTTGCATAAATGTCGCATTAATATAGAGAGTTTTAAAATAATTTCAACTTTCAAGTCAAGTCAACTTATAACAGAAATTCTCATTCATTACCTATTTATTAAATTAACAAGTCAAACGGGCAACTCCGGTAAGCAAGCAGCAGATCCGGCTGAACGGTTCGCAATACGGCAGGAAAACGGCCGTTTGGACGAACTGAGGAAGTGACGACCGACGAGTCACCAGTGCGACGATCACCGATTGTTCGAATTCTCGAGTAGCAGAGGCCTGATTTTTTTATACGGGAGGAAAGATGGAGAACTCGTTGTCTGATCAAAGCTTTAATTATATTGAGAGTGATGGTGAAGAAGATGCAGAAAATTATTTACATTATGATGGAAACAATTCTGATTCTTCGCAATCTTCAATGGAAATTAGACAGCACAGCAAACCCAATTCATATAATACCTCATGGCCACAGAGTTACAGGCAGTCCATTGATGTGTATAGTAGTGTGCCATCTCCAAGTATTGGTTTTCTGGGCAATTCTCCTTTGTCAAGGTTTGGTAGTTCATTTCTGTCTTCATCATTAACTCAGAGGCACACCTCTGAAGTTAAAAAACCTCTCCTACCAACACCCGCACAGGAACATCAAAGGCGTAGTTCTCACTCTCTAATTCCACCTGTTCCATCACGAAAGTCTTCTACTTTAAAAAAAGGAGATTTTGAGCACAAACACTCTGTAATTTCTCACGAGTGTTATGTTTCTCGAGATAGCACATTTGCACATGCTGTAATTAATGGTGTGAATGTTTTGTGTGGAGTAGGAATACTTTCTACTCCTTATGCTGTTAAGGAGGGAGGATGGCTTAGTCTTGGTTTACTACTTATCTTTGCTATTCTCTCTTTCTACACTGGACTTCTTTTACGTTATTGCTTGGACAGTTCTCCTGGGTTGGAGACCTATCCTGATATTGGTCAAGCTGCATTTGGCAAAGTTGGAAGATTTGCAATTTCGATAATTTTATACATGGAATTATATGCATGTTGTGTGGAGTACATAATACTGGAGGGTGATAACTTATCATCCATATTTCCAAATGCACAATTGACTTTGCTTGGTTATACGCTGGATTCCCATCACTTGTTTGCCATAGCAACCACTCTTGCTGTACTTCCGACATGTTGGCTGCGTGATCTTAGCCTTTTGAGTTACATATCAGCTGGCGGTGTTATTGCATCAGTATTGGTTGTGTTGTGCTTGTTTTGGATTGGCTTAGTGGATCAGGTGGGATTTGTTAGAAGTGGATCATCAATTAACTTATCCACTCTTCCTGTTGCTATTGGTCTTTATGGCTATTGCTACTCTGGACATGCCGTCTTCCCCAACATTTATACTTCTATGGCAAATCGAAACCAATATCCAGCTTTTCTCCTCACTAGCTTTGCTATTTGTACTTTAATGTGTGCGGGAGCTGCTGTTGTTGGATACATGATGTTTGGTGAATCAACAGAGTCACAGTTCACGCTTAATATGCCCTCAAATTTAGTTGCTTCGAAGGTGGCCATTTGGACTACGGTGGTCAATCCATTCACAAAATATGCTTTGACTATGTCACCGGTGGCTATGTGCCTAGAAGAGTTGATACCATCTCGCCATCTCAAATTTCATTTATACCCAATTCTTATTAGAAGTGCTCTAGTGATATCAACTTTATTGGTTGGTCTCAGTATCCCTTTTTTCGGTCTTGTAATGGCATTGATTGGATCATTACTTACAATGCTTGTTACTTTGATATTGCCATGTGCTTGCTACCTCAGCATCATGAGGGACAAGGTGACTTGTTATCAGGTATCTGCTTGTGTTCTTGTTATGATTGTTGGGGTTGTGTCATCGTCAATCGGAACAACTTCCGCTCTCTCTAAAATTATTGAGAATTTGTCATGAGGTCCCCCTCTTTTTGATGGTTTAGAGCATAATCAATGCTCGGGCACCCCAAATCATTGCTTTCATATTCTCCTTTTGACACATAATACACTAACTTTTTTATCTATCCTACTCAATTTTAAGCTTATTTTTTTTATAGAAAATGGTAGAAAATGAGTTTTTTTTTTTTCATTTTTATAAAAAGTATGATAATATGGTACTTAAATTTTAGAAAATAAAAAAATATAGAATTGTTG

*S.maritima*32186

AATGATAAAAATAATTTTTGGTCCAAGTTAAAATGTAAACAACTTTCAAGAGCGAATGAAGTACTTGGTAAGTAGTATCCCCTGCAAAAATGTAGTAGGTATCAAATCTTTAGAAATATTCATCATTGAGATGTAATTGCCGCGCAAAATGGGAGTCTCGGAAATTGAAGTCACACGAAAAAAGAAGAATGTAATCAATATGAACCAAAGTATAAAAGAAAAACAAAACTGTAGTTGTGCAATGTGATGCACAGGATTCAAGCTCTCTTCTTCCTACTTGGCACACTACTCTTTTTAGCGACAGCTCCAGTTTTGCATGATGATGGTAACATGCCACGTAACACTGCCAACAACGCTACGCCCATGAACGGAAGGTACACCGTCTTCAACTTATCTGACGCTTTGCCGGAATTAAAGAGTTCAAAAAGTATTGCAACCATGGAAGTGGTGACGAATGATCCATACATGAGGCAAGCTTTCGAAAACCATGATTTAGAAAAAATGAGACCAAAAAGATTTACCAATGATAGTGGCCAAAGAACAAATAACTCCATCCAAACAAGACCCACAAAGAAATGTGGCTTCTCAATCACCAAATAATCCCCATACTCATTGCTATACCATGAGTTTAGATCTAACAAAGCTTTGGGGTAATACTCTTTTGGCAAACAATTTTGAGCATCAAACAATGGTATTATGATTATCATTAACAAGAAAAACACAAATAGGATTGAGTCTAAGAGTTTTACGACACAACCCATTTTTTTTATTAAAAAATGAAATGCTGGTATAATTTTGTGAGGGGGTTTTGAGACAAGTTTGATTGATTGATTCACTCCTTTCTTTCTTGTTTGTTTGTTTAAAGGTTTTTAACAAGATGTGAAAATTGAGATGTTTTTCG

*S.maritima*37741

GTAGTAGTAGTAGTAGTAGTAGTTGGTGATGAAGCCTGAGGCTGAGGAGGTTTCTTCTTCTTCATCATCTACTTTGATGGGGGAAGATGCCTCCTCCTATTGTGATGCTCCACTATCCAAAATCGTTGCTGTTAAGGTCAAGGATTCCTTGTGTAATACACAGAACGACACCGATAACTGTTGCGTTAATGTTCATCACCAAACTCAAACTCAAACTCAAATTGGTGAGGATGGCTTAATTTCAAGCTCCCATTCTGAAACCACTAGCGTTAATGGCGAGGAATCCCTGCCATCTGATCCTCATTCACCTGCTCCTGTCGTGGATCGTGTTCAAGTTCAACAATCTCAGATTAAAGTTGATTCTAACAAGCTGGATTTAAAAGCTGCCTTAGAGCCTCCTATTGGAGATGTTTTGGCTGTGGCTGAGGATCTTGATGTAGAGCGTAATGAAGCACGCGGAGATGGAGTCAAACATGATAAGATTGTCCGAACAATGAGTAGCCAGTGTGCTTTGGATGTCGAATCTGTGCCGCTCTGGGGATTTACATCAATATGTGGTAGAAGGCCCGAGATGGAAGATGCTCTTGCTGTTGTGCCCTTTTTTCAGAGACTTCCTGCTAAATTGTTGATGGCTGAAAATATTTTGAATGGTATCACTGAAACTCTAAGCCAGGTTGTCCACTTCTATGCTGTATATGATGGCCATGGAGGCTGTCAGGTTGCCAACTATTGCCGTGATCACCTGCATCAAGCGTTAGCTGAGGAAATTGATGTTGTCAAAGAGAGTTTTTTAACCAGAGGTTATGAGGAGAATATGCAACAGCAATGGGAGAAGGCTTTCTCACGTTGTTTTCTTAAAGTTGATGCCGAGATTGGGGGACTAAAGCAAAGGTCTTCCGAAGGAAGCAGCAATGTCACTGAAGCTACTACGGAACCTATTGCACCAGAAACTGTTGGTTCAACTGCAGTTGCAGCTATCGTTTGCCCAACTCATATCATAGTTGCTAATTGTGGTGATTCAAGAGCTGTTCTTTGTCGTGGAAAAGCACCCATGCCACTCTCTGTAGATCATAAACCTGATCGAAAAGACGAATGGGATAGGATAGAAGCTGCTGATGGCAAGGTTATACAATGGAATGGCTACCGTGTTTTTGGTGTTCTTGCAATGTCAAGATCAATTGGTGATCGCTACTTGAAGCCATGGATCATACCAGACCCAGAAGTGACAATAGTTCAGCGAGCAAAAGACGATGATTGCCTAATTCTTGCTAGTGACGGACTATGGGATGTCCTGACCAATGAAGAAGTATGTGATGCAGCACGAAGGAGAATTCTTCTTTGGCATAAGAAGAATGGTCCTAATATAGCAGCAGATAGAGGCAACGGTGTTGATTTGGCAGCTCAAGCAGCAGCAGATTACCTATCCAAGCTTGCCCTCCAAAAGGGAAGCAAAGACAACATTACTGTCATTGTGGTTGACTTGAAACCCAATAGAAAGTTTAAAACCAAAACTTGAAAGGTTCACAACTTCACATCCATTTTGGCAAAATGTTAATTCTTTTTCTTCTTTATTTTTATTTTTTTTTAAAATTTTTTTGCCTCCTTTGACCTGTAGTTGTATGAGGTAGAAATATGTGTAACTCAGCAGCACTCAGCACCCAGCAGCAGCATTACATAATCATGGATTTGTAACATAATATTTTTAAAAGCCCATCTTTTTAAAATGGGCTGATTGTAGCTCAAGAATTTGTATAGTATATTTTTCAGAGTATCCTTTTTAGACCAGACCCCAGAACCAACAAGAATATACTAGTCATCTATGTTGGCATAAAGTCCTAACTAATAACATTTTTATGAACAGATAATTCCTTGTCTTGCAATTGTGAAGAATGTGTGTTTTTGTATGATGGTTT

*S.maritima*6020

GCAAGAAGATCAGATAATCCCCCCAACAATAACATCTCAGATTTTTTGGTTCTTTATACAAAGGTCAAATTTTTAGGGTGGGTATTCCAACAATTACAAGAATAATGGTTTTGAAACCCTAAAACTGATTGAAGGAAGTAATGGAATTGGATGATGCCTTTCACCTGCAAAATGGCTACATTTGCCATACATTGAGTCGAACCGGGTCACGGGGCATCTTCTTTGGCGATGACCCGATCCGTTTTTCCATACCCGGATTGCTGCTCCAGCTCTCTCTCATTTCTGTTTTCACACGTGCCTCACATTTTCTTCTCAAGCCTTTTGGCCAGCCTTCCATTGTCTCTCAAATTCTCGGAGGCGTAATTCTTGGACCTTCAGTCCTAGGCCACAACAGAAAGTTTCTAGCAGAAGTTTTTCCTGCTAAAAGCAAAATTCTGTTAGATAACTTATCAATATTCAGCTTAATGCTCTTTATATTTCTTTTAGGGGTGAAGATGGATTTGGCATTAGCCTTAAGGTCAGGGAAGAAGCCAATTGCCATTGGATTATTGGGGTTTATAGTTCCTTACGCGCTTGCTAGTTTGGCTGCATTCCTCTTGCAACACTTTGTATCATTAGATCATGACATCTTAAGTATACTTCCTTTTGTAGTTGAGACACAGTCCATGTCTGCGTTTCCTGTTATCGCTTGTTTTCTTGCTGATCTTAAGATCCTTAACTCGGAGATTGGGAGATTAGCTTCTTCCTCTTCTGTGATTTCAGATGTTTTTCAGTGGACTCTTATGACAATTAGGTTCGCGGTTAGGATAGCCAAAGCTAAATCATTTGAATCGTCTTTAGGGTCATTCTTGTCATCTGCTCTTTTCATTGTATTGGTCTTGTATGGAATCCGCCCTGCAGTTTTGTGGGCGATCAGGAAAACCCCTGAGGGAGAACCTGTAAAAGAAAGGTACATTTTCGGGGTTCTTGTGACTCTTTTGGGGTGTGGATTCATAGGTGAAGTTATTGGACTTAGTGCGGTTATAGCTTCTTTTATTGTTGGACTTGTTATACCAGATGGACCCCCTTTAGGAGCTGCTCTATCAGAAAAGCTTGATTCCTTTGTGTCTGTGCTTCTTATGCCTATCTTCTTCGCCATTTGTGGCCTTCAAATGGATGTGTTTGCCATTCAGAAGTTGAAAAATGTAGGTGTGATTCAGTTGGTGGTGTTTGTTGCCTTTGTTGGGAAGATCGTAGGGACGATTGCACCCCCTCTCTTTTACAGGATGCCATTTCGAGATGCCTTTTCTCTCAGCCTCATCATGAATGCCAAAGGTATCATTGAATTGGCCTTGCTGAATCATAGAATCAAGGAAAATGGCATGAGTGATGAATGTTTTGCAGTTATGATCATATCTGTGGTGATCGTCACAGGAGCTCTGTCACCAATTGTGAAGGCCCTTTATGATCCTTCGAGGAGGTATGTGGCATTTAAGAGGAGAACCATAAGACATCAGAAGCGTGATGAAGAGCTTCGAATATTGGCTTGTGTCCATTGTGCAGATAATATTAAGATAGTCATGGATGTCCTACACTTCTCTACTCCCACTACACAAAGCCCTATAAACTTAACCGTCATGCATCTCATCCGGCTAATAGGTCGTTCTTCTTCCCTATTAGTTGCTCATCATCCTCGGGAAACATCATCTCCCTACCCTAGTCAATCTGAGAAGATTTTCAACACATTCAAAAAGCTGGAAAAACAATGCCCTGGTCTTTATACAGTTCACTGTTTTAAGGGTATTTCTCCTTTTGCATCTATGCACAATGATGTATGCTCCATTGCTTTAGAAAAGAGGAGTTCAATCATCATAATCCCTTTTCAAAGGCAATGTGGCGAAAAAGAGATAGTAGAACCATCTTTTGTTTATAGAAACCTTAACAAGAAAGTTCTAGATAAGGCTCCTTGCTCAGTTGGAGTCCTTGTTGACCATAGAGGATCGCATAAGAATAGGAAAATTAGGCTTTCAACACCAGAGCCCTCAATGTATCAGGTAGCAGTGCTTTTTATCGGGGGCAAAGATGACAGAGAGGGGCTAGCATATGGCATGAGAATGTCGGAGAATCCTAGAGTAATACTAACCCTTATAAAATTCACAGGCTCTAATTCTGAAGACATTATAAATGACAACGAGCAAAGCAGACTGCTCGATGGGGACATTCTCAGTGAATTTAGGCTCAACACTATGCACAGAGAAGATAGAGTTTTTTATGAAGAGATGATATTGACAAATACGATGAGTTTGGTAACTCTAATAAAATCTATGGAGAGTTCTAAATATGACTTGATTTTGGTAGGAAGAAGTCATAAAAGATCTCGGTTTTTGACTGAACTTGTACAGTGGAGTGACTCTTCCGGGGAATTAGGGCCAATTGGGGAAATGCTCGCTATAAATAGTAAAATCAAAGCTTCTATATTGGTGGTGCAACAACAATCAAGGGTCTGGGGTCTGCAAGACCCTGAAGGTTCCACCCATCTAAGACGAATGGATTTGTAGTAACGATCTTGTTTAGATTAGACACATTATCTTCAATGAAAATATATACTGATCTCACATTTTGCAACTACTTGAAGCAAGCTTTTGAATGAATTATCTGT

*S.maritima*13022

TTTTTTTTTTTCAAAATTCAAAAAGAAGCTTTTATTCTTTAAGAAAAAAAATGTTTTACAAGTTGTTAGGTCGACCAGTTAGGTGCAGAACCACCTCCCCAGTTGGTATTGGCTGCACCAGTAGCAGGCGCTGCATTCCATTCTTCAGCCGCCCAGTCAAGAGCTGGAGCAGGAGTAGCTGCAGCTACTGCTAATGGTGGCACAGGAGTTCCATCTTCAGCCCAATTTTGTGGAGCAGTAGCTGCTACTGCACCCTCATTCCAGTCAGCAGCTGTATTGGACAATGAATAAGCTTCTTCCACACCAACAACCGTCTTAACAGTTGAAGCTGCAGGCAATGCTTCTTTGGCAGCAGCTTCTTCTTTTTCAACATCTTCAGGATCTCTGTAGAAGAACAAGTCAACAACAACATCCCATTTACCATCACGAGGAATAGTTCCCCTAAGACGAAGTACTTCACGGGCCAAGAACCACCACATAAGTCCAATAGAATGAGGAGACTTATTATTACACGGAATGGCAATGTCAACATATCTTAATGGAGTATCTGCATTACATAAAGCAATAACTGGTATGTTGACATAAGAAGCTTCAGTGATTGGCTGATGATCAGTGGCTGGATCGGTTACAACAAGAAGTCTAGGTTCACGGAAAGCAGCTTGTATCTGATTAGTAAAAGCCCCTGGAGTGAAACGACCAGCAATTGGTGTGGCACCAGTGTAAGCTGCAAACTTCAATACAGCACGTTGTCCATAAGGCCTAGATGAAATTACGAAAATTTCTGATGGATGTTCAACAGCAACAATAGCTCTAGCTGCTAACAACAGTTTCTCCCATGTCTTGTTCAAGTTGAAAATGTTCAATCCGCGGCTGTTTCTCTTATAAGCATATTGTTCCATTTGAAAGTTGACATTTTCAGCACCCAAGTGGGTGGTGGCAGCCAACATCTTGGTGACATCGTCTTCTTTCAAGCCCAATACATCTAGTCCTCCCGACATGTTGCAGAGTTGGTCAGACGTCTAATTCAACAAGTTTTATTAAAAAAATTAACCAGAAATTATAAGACTTGAAAAGAGTCAACGAAAAAGAAACATTTTACAGAACGAGAGCGCCGCGTGAATAG

*S.maritima*33760

ATCACAATGAATGCTATGATCCTATGAAAGTTCCATTGATTTCTTACATTTTATAAAAAGAAATGTGATTGAATGATTTGATGAATGTCTGAATAAATTTGGAAAAAAAAAAAAGAAAAATGAAGGAAAAAATAACAGAACATCATCATCTTCTTCTCAAGGTAGAAGATGAAAGTAATGATGTGGGTCCAACAGTACCAACAATGGAGGATTCATCATCGCATTCGATCACTCCTGTGTTGCTTCTCTCTGCTTTCGTTGCCGTTTTGGGTTCATTCTCCTTTGGCATTGTTATGGGTTATTCATCACCTGTGCAAGCTGAAATCATGAAGGAACTTGATCTTTCAACAGCTGAGTATTCTCTGTTTGGATCAATGATTAATATTGGAGGGTTATTTGGTGCTATACTATGTGGCAAGATAACAGATTATCTAGGTCGAAGAAATGCATTGAGACTAATAGACCTGTTTTACATATTTGGCTGGATTTTCATATCAGCTAGCCAGGCTGTGTGGTTGCTCGACCTGGGTAGATGGATGTTGGGTTTTGCTGTAGCCGTTACTGGTTATGCTGTTCCCGTATATGTGGCAGAATTCACTCCTAAAAATGTTCGGGGAGGATTTATGGGTTTGCATGTGTTAATGCTTACAGTAGGAGCTTCAGCAGTATTTCTCATTGGACTAGTCGTCTATTGGCGTTCTTTAGCTCTCATTGGACTTATTCCAAGTGTCATACAGTTAGCTGGCACATTCTTCATTCCGGAATCACCTAGATGGCTGATGATGGCGAATAAAAACAACAAGGAGTTTGAAGCCACTTTGCAACGCTTGAGGGGAGGTTCTGTTGACATTTCCCAGGAAGCAGCTGATTTAAGGGATTGTGTTGAAGCTCTTCATGAAATGGAACGCGTTAGCATCCTACAACTATTTCAGAAGAAGTTTGCTTATGCACTCACAGTGGGGATTGGCCTTTCAACACTTACTTCCCTTGTTGGTTTAACCGGGATCATCTTATATGCCAACTCTATATTCGCTTCAGTTGGTATTTCAGTGACGGTGGGAACTGTTGTATTGGCTCTTTTTCAGATCCCTAGTGTGGGTTTGGGTATGCTTTTGATGGATAAATGTGGGAGACGCCCTCTTCTTATGATTTCAGCTGCTGGGCTTTGCTTTGGTTGCTTGCTCACAGGCTTTGCATTTCTGTTTGAGGAGCATCATTTGTTGGATGGCTTTTGTCCCTATATTGGACTCATTGGCGTATTGGTCTATTTTGCTTCATATCCATTAGGTATGGGCGGAGCACATTCTTTGGTAGTTTCTGAGATATTTCCCTTGAATATAAAAGGGTCAGCAGGTAGTCTTCTTGCTATAATATCCGAAGTAGTAGGATGGATTGTTGCTTATGCTTTCAACTTCTCCATGGGGTGGAGCTCTTCAGGAACGTTTTTCGTATTAGCAATTAATTCTGCATTGGCACTGCTGTTTGTTGCTAAGTTGCTGCCTGAGACAAAGGGGAAGACACTTGAAGAAGTTCATTTGTCGATAAGTAAGATTTGGCAATGATATACATATACTCCGGTTTTTAATCAGCTGCAATTTCTGTTTATTTGCATTTTTTCATGATGCAAAAGCAATTATCATTACATGTAAAAGTTGGCCTACATTGTGAAAGGTTTATATTCAAAACAGAGAAACTAAGCTCTGTATTAAGGTGAAATATTGTGAGTGTAATTAAG

*S.maritima*11130

GGGTGAAGTTTGGATATGAAAAATTGAAAACCACAGCCCAAAAATCAAAAACACAAACAAAAACAGACAACTTTGTTTCTTCTTCTTCCTTCACTCACTCTCATTTCATATCCAAACTTCACCCACCTTATCTCCCTCAAACACACACACTAACACTCTCATTTGACCTCATTCTTCGTCTTCAGCAATGGCAGACGAAGAAAACGAAGATGAAAAACAACCACCACCCCAAGAAACTGAACAACCCGAAGAAGAAGAAGGAGGCTACAAATCCGACAGTACCTCCAACTACTCTTCTGACTCAGAAGACGAAGAGCTCAACAACGAAGAACAACAAAAACTCAAAGACGAGCAGCTGTACTACACAAGAGCAGGCGAATTCGAGGACATTCCTAAAGAAGACAACACTGAAGCCGCAAACATTGAGAGATTCAACAGAGTATTCGAGTCACAGAGGTATAAGAGAAAATTGGAAGAAGAAGAGAGAAGAATTGAGTACATTGAAGACTTGTATAATTTCCCAATTGATAAAGAGAATTGGAGAGAAGAAGATCTTAAGGAGTTATGGAGTGATGCACCATTAGAGATGAGTAAACCTGGGTGGGACCCAGCTTTTGCTACTGATAAAGATTGGCAAGTGATTGAAGAAGAAGTTGATGAAGGTCGTGACCCTCCAATTGCTCCCTTTTACTTGCCGTATCGAAAGTGTTATCCTGTTATACCTGAAAACCATCATGATATTTCTAACCCCAAAGCTGTTATTGAGGAATTGGATCGAATTGAGGAGTTTCTTAGATGGGTTAGCTATGTTTTTAAAGATGGAAGCTCGTATGAAGGCACTGTATGGGATGATTTGGCTCATGGAAAAGGTGTTTATGTTGCTGAGCAAGGCCTAGTCAGGTATGAGGGAGAATGGCTTCAGAACAACATGGAGGGGCACGGGGTTGTTGAAGTTGATATACCTGATGTAGAACCTATTCCTGGTTCAAAACTTGAAGCACAAATGCGTGCTGAAGGGAAAATCATAAAGAGAGATTACATGAGCCCAGAGGACAGGAAGTGGCTGGAGATGGATATTGAAGACAGTGTTCGTCTCACTAATGGGAACTATGAAATCCCTTTCTACGAGAAAGATATTTGGATACAACAATTTGGGGAGAAGCCGGAAAAAGGCCGGTATCGTTATGCTGGTCAGTGGAAGCATGGCAGAATGCATGGCTGTGGTGTTTATGAAGTCAATGAGAGGCCCATTTTGGGTCGATTCTACTTTGGCGAGCTACTGAACGAATCTGCTGGTTGTACTCCTGAAATTTCTATGATGCATGCAGGTATAGCTGAAGTTGCTGCAGCCAAGGCTCGAATGTTTATCAACAAGCCAGATGGAATGGTTAGAGAAGAGAGGGGTCCATATAGTGATCCACAACATCCCTATTTTTACGAGGAAGAGGATGTGTGGCAGGCTCCAGGCTTCATTAATCAGTTTCATGAGGTTCCTGATTATTGGAAAACTTATGTGAGTGAAGTAGATCAAGAAAGGGAAATGTGGCTGAACTCTTTCTATAAAGCACCATTGAGGCTGCCAATGCCTGCAGAGCTCGAGCATTGGTGGGAAAAAGAACAACCTCCACAATTTGTTATTTTAAACAAGGAGCCGGAACCTGACCCTAATGATCCTTCCAAGCTCATTTATACTGAGGACCCTGTCATACTGCACACGCCAACTGGGCGAATAATCAATTACATTGAGGATGAAGAACATGGCATTCGCTTGTTTTGGCAACCACCTCTGAGTGAGGGTGAAGATGTTGACCCTACGAAGGTCACATTTTTGCCTCTGGGGTTTGATGAATTCTATGGACGGGGAACAATGGAGAAGAAAGAGGGCCTTTTTAAACGTATTGTGTGTTCAATAGAGAACACACTTAAACCCTGGCTTGATAAGCTGGAAAAATGGACTGAAGAGCAGAAGAAAGAGAGTGAACTTAGAAAACAACTCTTAGAACAGGAGCTTGAATTGGTAGAGGCTGAATTGACCTTGGAAGAGGCCCTTGAAGACATGGAAGAGGCCTTGAAGCAGCAAGAGAAGGAGGCAGAGAGTAGAATGGATGTTGATGCCGAGGAAGAGGATGTATCAGCATCTCCTCCTTCAGATGTTGGTAAGGAGGTGAAAGTTTCTTCCAAGGAGGAAATCAAAGGTGATGATGAAGAGGATGAGGAAGAGGAAGAAGAAGAAGACGATGATGGTAGTGGTTTAGCACCGTCAAGTTTTGGTTCTGTTGCAGATCCAAAGGACAAGAAAAATGGACCTGGGAAGTCTCCTTTCTCTACACTTTCATTTGCTTCTGGTTTGCTTTCAACAGTTCCTTCAATGTTGGAGAATTCTTTTTCAGCGTGGAAAAAGGAAAGATCTCCGCCAAAAGTTCCACAAGCATCATGCAGCAGTACCTCAAGCTGTCAAGAAAATGCTAATTTGGTCTCTTTTTGCCAGTCATTACCAGAAAATATGAGCTTGAGAGCAAAGGCCTATCAAAAGATTCAGGTAAAACGAGGTAGAAAGCTGCGCACTGCATGTCATACAACTAGTAGCCAGTCAACATATTCCAAAAATGAAGGCAAGGGGCATAGATCTTTGGAGTTTAATTGGATGTGTGAACCACCAGAGAGAAAGTCAGATATGATATTGTCCTTGCACATACCAGTTGAAAACTTGGATTTGTGCATGGGATAATTCATGTTATAAAATATGTACGCTGTGTACCCATTTTGTAGTTTATATATCTTGAAGGCATGATTTTGTAATCCAAGATGAGTTGAGCTGCTGATTCATTTGCTGGCATCTGGATGTTTTATAAACAATCACTAGTTTGTTGCACCATTGTTCTGTATCAGTTGAAACGAAACAAATAACAATGCAAGTTGATTAGTTGATTGCGATTTCGTTTTTTGTTTTAACTGTCTGCTATAGTTTTAAAGCCTTGTACATGCTATTGTGTTTGTATTGCTTTTAGAATGTATGCTTTTGTTTAGACAAAGAGTCACAA

*S.maritima*35771

TTTTTATTATAGTCACTGGGGGATACCTAGGTTTCAAAACTGGATGGGTTGGCTATGAGCTCCCAAAAGGGTACTTTCCTTTTGGCATAAATGGTGTGCTTGCTGGATCTGCTACAGTTTTTTTCTCTTATATTGGCTTTGATGCTGTTGCAAGTACTGCTGAAGAGGTGAAGAACCCAAAACGAGATTTACCATTGGGTATAGCCTTTTCATTGTTAATATGCTGCACATTGTACATGCTGGTATCTGTTGTAGTTGTTGGCCTGATTCCATATTATGATCTTGATCCGGACACTCCTATTTCTTCTGCATTCTCTAGTCAAGGGATGCATTGGGCAGCGTATATAATCACTATTGGTGCATCAACAGCTCTATGTGCATCTCTGCTGGGTTCATTTCTTCCACAGCCTCGAGTCCTCATGGCAATGGCTAGAGATGGGTTGCTGCCCTCATTTTTCTCAGACATCAACCAACGGACACAAGTCCCTGTGAAGAGTACCATCGTGACAGGAGCTTTTGCTGCAGTTTTAGCCTTTTTCATGGATGTTTCGCAGCTGGCAGGAATGGTTAGTGTAGGAACTCTTTTGTCATTCACAATTGTTGCCATATCTATACTGATAATCAGATATGTTCCACCGGATGAGGTACCACTTCCAACTTCTCTTCTGGTGTCAACATGTGTAACTTCATTGGAATATGATGGTAATGTCGAGGAGGTTGATGGGGCTTCTAGTGAAAGTCATGTTGGTACCGCCGAGCATGAAATCCAACATCTACTTCACGACAGACACTTATCTGCTGAACATCCTCTGATTTTGAAGCAATCAGTTGACTGCAGTAGTAAGTGTAAAGCATCCTAGATTTGCATTTAATTTCAGCCCGCCTCCTACCAGTCACCAGACAGTGAGCAATGGAAAGAAGCATTAACACATTAACTGCCAGACCTTGAAATATTAGTGCAGACTACACAAGTGCATTGGTTCAACCTTAAGCTTACAAGTGCTGTAATCATCTTTTTGGTTGTGCAATTTTTCTCCATGGCATATGGGTTCCATAGTCTTAGATCTATGAGTTTATGAAATCCAAACTCTGACATGCTAATTTTAGAAATCGC

*S.maritima*35771

TTTTTATTATAGTCACTGGGGGATACCTAGGTTTCAAAACTGGATGGGTTGGCTATGAGCTCCCAAAAGGGTACTTTCCTTTTGGCATAAATGGTGTGCTTGCTGGATCTGCTACAGTTTTTTTCTCTTATATTGGCTTTGATGCTGTTGCAAGTACTGCTGAAGAGGTGAAGAACCCAAAACGAGATTTACCATTGGGTATAGCCTTTTCATTGTTAATATGCTGCACATTGTACATGCTGGTATCTGTTGTAGTTGTTGGCCTGATTCCATATTATGATCTTGATCCGGACACTCCTATTTCTTCTGCATTCTCTAGTCAAGGGATGCATTGGGCAGCGTATATAATCACTATTGGTGCATCAACAGCTCTATGTGCATCTCTGCTGGGTTCATTTCTTCCACAGCCTCGAGTCCTCATGGCAATGGCTAGAGATGGGTTGCTGCCCTCATTTTTCTCAGACATCAACCAACGGACACAAGTCCCTGTGAAGAGTACCATCGTGACAGGAGCTTTTGCTGCAGTTTTAGCCTTTTTCATGGATGTTTCGCAGCTGGCAGGAATGGTTAGTGTAGGAACTCTTTTGTCATTCACAATTGTTGCCATATCTATACTGATAATCAGATATGTTCCACCGGATGAGGTACCACTTCCAACTTCTCTTCTGGTGTCAACATGTGTAACTTCATTGGAATATGATGGTAATGTCGAGGAGGTTGATGGGGCTTCTAGTGAAAGTCATGTTGGTACCGCCGAGCATGAAATCCAACATCTACTTCACGACAGACACTTATCTGCTGAACATCCTCTGATTTTGAAGCAATCAGTTGACTGCAGTAGTACTTCACATGATGAGAAGAGGCGAAAATTAGCTAGTTGGACCATAACACTGACATGTTTAGGGGCATTTATTCTCACAGTTGCAGCTTCATATGTAGAGCTTCCAAGGTTCGTAATCAGAAGTTTGATATTATTGGGATAATGTAGTCAACTTATCATACTTAACTACTGCAGCATAATTAAAGAAAAAACAATTTAAATTAGGGTAAAGGTTAGACTTCTATTAGACTGCTGCCTAGGTTAAATGGTAATTATAGTCCCTGAACCAGTGATTGTATTAGTGCCTACATGAACTAACATACTTATCAGGACATTATTCTGATTCTAATTTGGTGCCTTTTTCACCAGTATCCCTCGCTTCACATTATGTGCAGTAGGTGTTGCTCTTCTTTTATCTAGTTTGATAATTCTCTTTTGCATTAACCAAGATGATGCGAGGCACAGATTTGGCCACACAGGAGGTTTTATTTGTCCATTCGTTCCGCTTCTGCCTGTTGCTTCCATTCTGATCAACATATACTTGTTGGTAAATCTTGGAGGAGGCACTTGGGTTCGTGTGTTGATATGGATGACAATCGGAGTGCTTGTTTATGGGTTGTATGGGAGGACACATAGTACACTGCGGGATGCCGTTTATGTGTCTGCTGCAGATGCAGATAAGATCTTTCGTGGATTGTGTTCACATGATTAACATGACTAGGCAGACGTTCATACACTTAACATATCATGCAAATTCGGATACTGGAGTCATTGTTCTTGAGTGTGGAAGCTCTGTTATCTTTAGGTGTAGCATTTTATAAAGTTGCCAATTCGGTAATTGCATGATTATGTGTATGGGGAAACTTTTTTGGTAACACCATGTTTTAGATGTATATATTTGATAGTTACAAAAGAAAATGAGAAATTTAATTTTGTACCACTGTACTTTGCACTTTTTATCAATACACATTTTTTTTCT

*S.maritima*31043

ATTAGCCAACAATTGAGACCCACACCACAAACCCCATAACCTGTTTCCTTTGTGGAGTAGTACACTATACTATACAGTGTTGCAAATCCCCTCTCTTTCGTTTTCACTCTAACTTTCTTCTTCTCTTCTTCACAACTGCGAGTGGGTTGCGGGCGGCACAACCACACACCACCACGTTGCCACACCAGCTCCACCACAACCACCCATTCGCTGAGGAATTACAGTTTGTAATCTGTCTTTGGCACGCAACAGCAACATTAACAATGGCGGAAACTGATGCAAGCCAGTTAATATGCTCAAATTGTGACAGCCAGGGGTTATACGTCGATGACGACGGTCATTTCTACTGCTTAAACTGCAATTCTCAGGCCCAAGACATCGTTGATGGAGGCAATGCGGATGAAGATTTTCTAGACAAAGGCAGTCAGGGCGGCGTAGCTCTTTACACTGCGGCACATAAGCGCAATGTTATTCAACCCTCATCTTTTGTACCTGATTCCCAAATTGGTTGCGATCATATCGGTCCTGTTGAACCGGCTGATTTTGGGGTTAGTCCTAAGGGGGTTCATTTCAAAGAAGAGGATTATTATAATGCCATTCGTTTGAGATATGTTTTGGGTGTTCAGTTGATGATTCAGTATCAGTGTGAAGCTCTTGTTGAGGAGTTTGGGGTTACTCCTCTGATTTGTGGCATTGCTACTTCTATCTGGATGCGCTTTGTTGCTGTTACCAAGGTTTTCGAGGATGGTTGGGCAGACAAAACTGTCTTTGACTCTGAAACTGCGGAGAAAAGGTACCAAGTAGGAAAACATGTGCCAAATGAGCCTCGAAATATGCATAATGAACGTATGGTAATAATATGGTTGAAATCTTTGAAAAAGATGATACCCTTATCGCATTCCCTAGCGATTTCATATTTGGCTTGTCATGTTGCTAAAGAACCAATTCTTTCAACTGACATTATAAAGTGGGCTATTGAAGGAAAGCTTCCTTATTTCGGCGTTTTTTCTAAGATTGCGGAATCCATCAAAGAGATTGAGAAAGACAACAAACATGTTTCGGTTGTATGTCCATTGAAAGCAAGTTCTATGTTTAGGCCTTCAGAACTTCCTTTGCAGAAGTTGGAGTCAATGGCGGCGTCTATTGCTCACTCTGTAGGGTTGAATTTGCCTCCCGTAAACTTTTACCGTATAGCTTGCCGTTATCTAAAGCAGTTGTCCCTCCCTGTGGAAAAAATCATTCCATATGCATGCCGCATCCAAGAATGGTCCATGCCTCCTAATTTATGGTTGTCAACAGCAGAAAAGCTTCCTACTCGTGTTTGTGTTATGTCAATATTGATTGTTGCAATAAGGATACTATTTAATATTAATGGTTTTGGCAAATGGGAAGCATCTCTATCTACTCCAATGGGCTCCTGCTCCAGGGATGAACAGGCTGAAACTCCTGGCAAATCTAATGCAATGAATGGTGCTACCATACCCCCTAAAAATCCAAGTGATGGACCTGAAGCATGTTCATCATATGTTCATAAATTTGAGTCGGACAGCACAGAGCTTTTGCGGAACCTTGAAAAGAGATATGATGAATTAAAAGATAACTTTGAATATGCTGAGGATTTACCAACATATCTTCGATACTGCAAGGGTGTTGTATTTGCGGGCTTAGAACCATTATACAAGGATCCTACTTTGATAGAAGAACTTTGGGATTTTTATGAGAAGAATCGAGAGCACATTAATACATCTGAGGAATTTGAAACTGGATCAAAGCAGAAAAGGTCTAGGGATTATGACAAGTTGTTCAGTACCCCGTCAAAGGATAGCAAGAAGCATAGAGATGAAGAAATTACCAGCTCTTCATCACCCGATAGCAATAGTGTCAGGCCCAAAATGGCTGATAGTGCTGAACAAAGTCCACAAACATGTCAAATTCCAGCTATTGATGAGTTAGGTGAAGCATCGTCTCAAAGTTATGAAGATATAGCACTTAAGCATCTCAAAATAGACATGGAAGAACATATCTTCACGTACATTCCGCCAAGGGTGAACATCAAGAGACATGATCATCTGCACTATATTCGAAAGAAGGGTGATGGTTCTTTGGCATATGTTGCTCATGCTGATTACTATATCCTACTTAGAGCTTGTGCTAAAGTGGCCCAAGTTGATGTTCGTGTTATGCATTCAAGTGTACTCCGTTTGGAAAGAAGATTAGCTTGGATTGAGAAGTGGATCGATCATAGTTTGATGGGAAAATGTTCCAGTGGTCCTACAAATGATGATGCTGACTTGGATGCTGATATGGATGATGAATCTATTAATTTATCGGATCTAAGTATATGACTTTTGATTTGCACTTGCTAGCCTTTGTCTCATTGTATGTATTGGTGGTACCTTCTTTTCTTTTTTAGTAGACAAAAGTGCTTGAGTTTGGGACTTGATTGTCAAATGAAATTATCTACTTCTGTCTAGAAACAGTGATTCACATGGTCATCTAGTGGAGCTTCTTTTCTGGGTAATGTTCAGAAGGCTGATGTTTCTATGAAAAAAAGCTTCTTTCTATGGAAATTTGGATAGTGAAGACTATAATCAGGTGTTTTGCGCAAAAGATAGAACATGCAGAATGTGGTTTCCTGATGTTGTTACGGGTTATTATTTCAGATTTCCGTGGATGATGTAATGGAGCGCCAGGAAAGACTTGCTGAAATGCGAAGTCTTGTTTTCCGTCATGAATTAAAGGCTA

*S.maritima*13729

CTACTACTACTACTACTACTATTATTACCACCTGAGCAGTAGGAGTAGCAGCAGCATCAGCAGTAGCCGACGTCTCGCGGTGTCGTCCGCGTACGGATAAAAAAAAAAATAGAAATCTCCTCTCTGGTTTAGTTTTCTCATTTAATTCAACTTGCCCTTCGCAACCCCCGAGTCCCCGTAATAACCCGCACGCACCACGTCCGATTTTTCCTTTGACCGTGTCACCGCCAAACTGGTAACAATTTCGCACCGCCACAGCTACCCGTCGTAGTAGTAGCCACGGCACCCAAACGACCGCCGCCGTACCGCACGACCGCCGGCCGCAGCCGCCGCCACTGTTGCACCACTGCAGCGCGCCTTAACGACGACGGACAGCCGCACGGCTGCACCAGACCGCACCAGACGCACCGCTATGTCGCATCATTTCGCCGCCGCCGGAGACGTCGCTGGAGTGCCCCTGAAATCGACGGGGTCCATGCGACCGATTATCACAGAATTGGATAACAACAAAAAAGGATCCATACGAACAGATGTTGCCGATCTTGTTATGGTCAAATACAAATGTTCATCGAACGGAGTACCTATTACACAGACCAATGGCTCGACGCTTCCGCTGGTATCAAGTACAAGCAAAGATGCCGAATTCGGTGGCTACAATCCGTTCGACCATCGGACGGTCCAATACCCTACTACGGATATGGAAACATTTATACATCTGCTGAAAGGGAGTCTGGGCTCAGGCATTTTGGCCATGCCTTTGGCTTTTATGAACGCTGGACTAGTGTTTGGGCTCGTTGCAACCGCTGTAATTGGATTCGTCTGTACATATTGTGTCCATATACTGGTAAAATCTTCTCATAAATTGTGCCGTAGAATGCAAGTACCAGCATTGGGATTTGCTGATGTTGCAGAAGTTGCCTTCCTCGCTGGACCTCCAGCCTTCCATAAATTTTCCGGCCTCTTCAGAGGCCTTGTTAACACGTTCCTAACCATTGATTTGCTTGGTTGCTGTTGTGTTTATATTGTTTTTGTTGCAAAAAATATTAAACAGGTGATGGATGAATATGTATTAGAAATAAGTGTTAGATGGTACATGTTGATGATGTTGCCGTTGGTAATCGCCATGAATTTAATACGTAATCTGAAGTACCTGGCTCCATTTTCAATGATTGCAAACTTTTTGGTTGGTATTTGCATGACCATTACATTCTGGTACGTGTTTCAAGACATGCCATCCCCAAAAACTGTACCTTACATTACGGATTGGCACAAATGGCCACTCTTCTTTGGCACTGCCATCTTTGCGTTGGAAGGGATTGGTGTTGTAATGCCATTGGAAAACAACATGAAAACACCACAGCATTTTATTGGTTGTCCCAGCGTGCTCAACATTGGCATGGCCATTGTTGTGATTTTGTATTCTACTGTTGGATTGTTTGGTTTCCTGAAGTATGGGGATAAGACTGAGGGCAGTATTACGCTGAACTTACCGAAAGACCAATTGTTGGCGCAATCCGTAAAGCTCATGATAGCTGTGGCCATTTTCCTTACATACAGTCTTCAGTTTTACGTACCCTTTGAGATCATCTGGAAAGGCTCCAAGCATCGGTTTACATCACACCCCGTTCTTTTTGAATATTTGCTCAGAGTGTTCTTGGTTATATGTACGGTATGTGTTGCAATTGCTTGTCCCAACCTTGGCCCTGTCATATCGCTCGTTGGAGCATTATGTCTGTCATTTTTGGGTCTAATCTTGCCAAGCTGTATCGATCTAGTTACAAACTGGGAAGAACCTGGATTGGGTAGATTATACTGGCGTCTATGGAAAAACGTGGTCATCATAATGTTTGGCATTTTGGGATTGGTTACCGGCGTTTACTGTAGTATACTTGACATCATCGTACAGTTCAATCAGTGAGCGTGCGCGCAAGTAATGATTTCAATTACAAAAGGACGGCAGTGACACAACTCTCATACAGACAGAACAGCAATATTAGCGTTGTCAATTACAGCTATTAGTGTCCAGTAAAATTGTTTTTAAACAAAAAAAAACGACAATAACTATTTTGTACTGATTAACTAGGGTGCAACAGTGAATTTTGAAAAAAAATTTAAATACAAAATCACGG

*S.maritima*31909

CTGGGTGACAGAGTGAGACTCTGGCTCAAAAAAAAAAAAAAAAAAAGGTTTGAAGTTGTCGTAAAATTAGGTTTAATGTGAGTAAAGACTAAAGAGTAACACAGTAATCCAATTCAATCGAAAACGTCTTTTTCCAACTTCAATCTTATTTCAATCACCGTCATCGATTGGCCTGCAAATTCGATTTAATTTTCTAAGTGAGCTAAGGGGTGGAAAGCTCAGTATAGTTTTGTGTGTGTAAAGATGCTTCAAATCCGGCTTAATAAAGGTGTGTCTTTAGATGGAAGCACTGGAGCTAAGCCATTGCCGGGGGAGACTGTTACTGTCGCATGCCCTGACCATCTTGTTCTTGCTGACCTTCCTGTTGCCAAGGGTCTTGGTTCTGCAACTTCTGCTTCAATGGTGAAGATTGTTGGTCGTAGATCTCGACGACAGCTTGCAGAGCGAGTCCACTTCTGTGTCCGTTGTGATTTCCCAATTGCCGTCTATGGCCGTTTGAGTCCATGTGAGCATGCCTTTTGTCTTGATTGTGCTAGGAGTGATTCTATCTGTTATCTATGTGATGAACGGATCCATAAGATACAAACAATCAAAATGATGGAGGGAATTTTCGTTTGTGCTGCTCCTCACTGTCTCAAATCATTTCTAAAAAAGTCTGAATTTGAGTCTCACATACATGAAAGCCATGCCGATTTGCTTCAGCCTAATATGAAGAAGGAAGAAATGAGTGAGTCAGAAGTATCAGGCTCAAAGCAGTCTTCAACATTAGATACAACTGTTCGAGGTCCGCAAAGATCAGGTTTTCCACTTAGCTCAAATGATCGTGATGATAAAGGTCGGCGCCAACAACCTAGAGAGCAACCTCCACTCAGACCACTTATGCCGCCCAAGCTACCCCCGTTTTATGGGCAAGCACATCCTTCTGACCCTCAACCTGACAACCGAGCAATGGGTCTTGATGGGCCAGGCCAAACTCGGATGCAACATCAAAATGTTGAGGCACAGGGTGGAACACAGCAGGACTTTGGACAGTTTTCAGACAAACAGCAGCAAGGGCTTATGCCCGATCCTCATCGTGAGTATCCACAAATGTACCCCCAACAACCACCTAATTTCCCGATGCCTGGTAATGGTAACACCATGATGATGGGTGCACCTCCATTTGGATTCCCTCCTTTCCAGTCTGATGGAGCTCCACCCTTTTATGGTGCCTCGAATACATATGAAGTAGCACGATCAGACTCCATTGGGGAAGGTGGGTCAGAACAAGGATCAGTGTTGGGCTTTGCACCAGGACCAGGTGGGCCTGTTAATTATCCTGATGGTTTTCCTCGCCCTTGGAATGGGGGACCAGCAAATGTTCCTTTTGAAGCTGCTCAAGGCGGGCAAGGTTTGATGGCACCTCCACCACCTCCACCAGGACCACCTCCTCCTCACTTGATGCAGCATAAACATGGATACTATTCAGGTGATTCGAGTAATGATGGAAAAAGCTATGGATGGCAATCAGAGAGGCATGACGGATTCGGAAGTAGCCAGGATTAGTGCAATTTTCTTTGGCAGTAATGTTGCATTTTTTGTACTCTTTACCTTTTCAGGTGTTGTGTAACTTTATCAAATGATCGGTTTCCTAGTAGTACAAAATATTTAGCAATCCTTTGGAACATTTTCCGTTCGCTGTTTGGCCTTAAATGCTTTACTGTGTTCCTGTTGGTGATACATGCTAGAATAGAAGAACTGCCATGAATGCAATGTGATATATTGTATCAACTTCAGAAAGTAAAGTTTAAACTGAAAGAGTTATGTTTTTGTAAGCAGGAAGCAGGAGAAATAATCTAAA

*S.maritima*1239142

CCCCATATAAAAAAACAAAGTACATTGGAACACAAAACAGTTTTTTAATCATTATTTGATACTATATTCAGGTACTCCCAAGAAATGCAAGCTAATGAATCCTTCAAGGTTTGGCTGGAGTGGCAGGCTTAGGGAGTTCTGGCATTGTCGGTTTCGGCAACTCAGGCATAGTGGGCTTGGGAATTTCAGGCATTATTGGTTTGGGCAGCTCTGGCACAGTGGGCTTTGGCCACTCGGGCATTGTGGGTTTTGGCATTTCGGGAATTTTGGGTTTTGGCATCTCAGGCAATGTGGGCTTAAATGGTTTGGGAATTTCTGGCATTGTGTGCTTGGGCAATTCTGGTTTTGGCACCTCAGGCAATTTAGGCTTGGGCATTTCAGGAACCTTGGGTTTGGGCATCTCATGCAATGTGGGCTTAGGAATTTCAGGTACTACCAGTTTGGGAATTTCAGGCACTGTGG

*S.maritima*41386

AGGCAACTGTACTCTGAACCAATTTACCCAGATGAAACATACACTTAAAAATTCACCAGTATTGGTAAAATTACAAAGAAAATTCTTCCTACAAGGTTGATCAATAGTCATAGATTTTGCGTTGGCTCAAAAATAACTCATACATGTTGCACTTCTTAATTTTTAATTAAACAAACTAAGCAAGTAACTAATAGACAAAGGGCTAATAAAGAAGCCAAAAGGTTACATAGAAATCCCAGAATTTGTTTCTGGCAAAAGATATCAGAGACCTGCTAATGTCATTTACAGATTAATGTTGTAAGTGAGAAGCCATAGAAGATGCCAGTGCCATGGATAACTTTTCTGTTCTGTCTACTTCAAGGGCCAGTTGATATTTGGTCTCACACCGATAAAAAAGACAACAGACAAATGCCATCTGTTGTAATTCTTCAATATAATCCTCTGATGGATGTTCTAGAACCAGTTTTTCTGTATTAATTAATTTGTGCAACAGTTTACTGCTCAGTTTGATGCCCTGCGGTAGATGGGTGGCGCTTGACCTTACGATATGCCCTTTCTGTATTGAATGAATCAACAGCTTCTTTAATCTTGGAAGTCATATCAGCAGCTTTGACAGAGGCAACCCGGAGGCACTGCATAACAACACTCTGCATGACGCCAACCCCTCCACCCTTTTGAATGGCACATATATCACCATTTGTGTTTAGTGTAACAGTCATTCTACCTTTCATTATGGCCTCTTCGGAATGAGATGGATCTATCACCACAACATTATCAGCAGTGAAGAATGCAAATGTCACAGCTATAGGAAGATGATGAACAATCAACGGAAGTGGATCACGCACCTCGGGCGGGTATACTGTTAATTCTTGCCCATCTTCTCCGCCAACACTACACTCTGGCCTCCTAAAGGTGAGAAGGGCTGACAAAGCTGCAATATTTGCAGCATCAACAAGATTCCCTCCATTATCTAGTATGTGAAGATCAATACGAATGGACCACACTAATTTACCAGCAAGGACACAAAGTGATTCAGTATCTATTGCCCTGCTTTCCCTCAAACCACGGTCTATTATACGTCCCAATTCCACAGCAGATTCTCCAGGACGGCCGGGCTCAAATGAAGGATCAGCCATGGGAGAAAACTCAGTAAAAATAGCAAGTGATCCTTCATTTGGCCTGTCTTTATAAGGTTGAACTAATTGTGAAGTCACAAACCCCATAACATGAGTTTGGCCAAGCTGCACTTCGGAAGAACCATCTTCTCGGCCAAACTTAATGGTTAGACGACGGTAGTCAAAAGGACGGCGGCCATCAACACGGAGATCAGAAAGCAATGCTGTCTCAATAAATTTCTTCTCATTCACAGTCATGTTCCAACTATTAGCTAATCTCTGCTCCATTTCTTTCTTAACACACTATACCAGCGACTGGGAAGGCTTCAAGAACTGAATTCCTCAGTGAAAAATTGAAAATTGCTTGTATTTTGAGAAGAAAGAATGGTGTTTGGAGGTATACAAGTTAGGTTTGTGTTTATTTTTGCTTCAAAAAAT

*S.maritima*39645

AAATCGATCAAGCTTCAATTGAAGCATTATGACAGGGAAGGAGAAAGAGAAAGAGAGGGAGCAAGAGAGACAGACAGAGGCGGAGGGAGAAAGAGCAAGAGAAGCGGGATGGGGTTATATTGCAAATGAAACAAATCAGGTTCGTTGCTCAAAATCAATCTGGGAAAGAAGAAAATGGACGAGGGAGATAGCGCGAATTTAGAGTTAGAGGAGGAGATTGAGGAGTTAGCAAAGAAGCTAATAGAGTTACAAAAGGGCTCTAAGATGAAAGGCCTTGAAATACAAAACTGCATCAACTTTGATAGGCAAGCTTCTTTGCTTCAGAAACAACTCGAAAAATACAACGAAATGCAGGGTGAAAACTGTGTTAAGGAGACAGCAGAAGGTGCTGCAGTTACCCTGCCTTTCAATTCTACAAGAGAGGGAAAGATGAAAGGTCATGGATCAAGTCACCGGACTGCAAGGTTCACTGATGTGGAGCTTTTGAAAAGAAAAGTGGAGGGGTTATCAAAGGGAAAGTTGTTGAAGAGAATGGAAGAGGAATATGGATATATGTTATCTGCTGCAGCTAACTATACTGTCCAAAATTCTGCTTCGACCTCCAAACAATATGAGCTACCGGATTCATCTTCCTTTTCATATCAGCAATCCTCACAGCTATGTTCACTCTTTGGTGATTGCCAATCTTTGTTATATGCTTCTGAGTTGCCTCATACAAATAAGTCGTAAAATTTTTTTTTTATCACTTGTCAATGGATATTAAAGGGATTCTGAGCTTATTTTGATTCTAAGTATCATTGGTTATGGCCGCTATAACTTGACGATGTTTCCTGCAGTATATACTCATGGAGGCAGTTTGTACAGTACAATGTTTTCCAAAAATTATTCGAGTACCCTGCTAAGTATCGCCCTCCCATCTATTTGTTGTATTATTAAGATTCTGATTGGATTTGGAGCAAAACCTTGGTTTT

*S.maritima*36703

TTGAACTGGAAATGATGGTATGGACTCCGTACATATAACTCATTCGAAGAACATCGAACCAATACAAGGTTTGGCTAATATACACATATAAGTTGAGAAGGGACAAATAGGATAATTTCTAGCCTAAACTTCAACATATGCCTGTAATGTCAATAATACGAATCCAAATGTTAAGGAATGAGTTCATAACCACATTAATTTATAGACCGTGGTATTAATTAATAACCACATTCATTCACTGATCCTCCCAAATTACCACCAGCTCTCGGAGTAAATCATTGATCAATTTTTTGACAACAGCCAACTATACATGGGAGTATGGGACAAACCCTTCATAATCGTAAATTTGAAATCGCCAGCTTTCTTGTAGGCAATCCAAGTGCATCCTTAATCTTGTAGTTGTACCACTTCTTGTCTTGGCTCTTCAAGTAGGGGACTATGTGCTTTTTCATATCATCATGTGAGATTCCGAGATGATTCCCAGCTACACTTACATACTTAACTCTTCCAGCATCATCAAGGGTCTTTAATCCAATCCAATCTTCAGTGTATAACTTTGTCTCCTGAGGAGTTAACAGAGGTTTGAAAGCACCATCTGGATAGTACCCGAACCATGAGGTTTCTTTAGGTACCAGAACTGTGTCATGCTCAAACATGATCAGCACCAAATTTTCTAAGCTGCTAAAACGCTCCTTGTATGTTGAATTTCTTTCCTCAGGAATTTCATTGTTTAGTTTTGGAAGAAACCTACACTTTTTCAAGTAGCCCTCCATATTATTTGGGATTTTAAGATAACCACTGGGTGCCAAATGAGCCTGAATGTAATCTGAGTAAATCTCAGCCTTTATAAGCGTATCTGCGAGTATACAGAAGATACCAGTGCCACAAAGAGGAACTGAAGCAATGCCAGCGTGAGGACCTCCCATTGAGACAAAGTTCCTAACCGGAGGCCCTCCTTCACAAAACTCCACAACACCTCGCCCAATCAAGTTACCCTGGGACAAACCAACTATGTTGTAACCATTACTCAATTCTTTCATTTGCTTCACCTTGTCACACACAACTTGCGCCTGGTCCTCAAGAGTCATAAACCAAGAATCCCAGGATCCATTACCAATTTCCACACAATGTCCTTTTGATGCGGACCACTCACTTAAGAGCTTTGTAAACTGCTTCATTCCACGATTTGAACATTGATCTCCAATTCCATGGAGTACAATGAAGGGAAGTGAGTGAGACAAAGAAACAAAGATGAAGGAGAAGACGAAGGTGAAAAGGATTGCAGAAGATTTCATGCTTTCTTCCCAGAAATTTTGGGAATTTATTTGGTGTTTGGTTTGAGCAAGAAGTAGTTCTGCTTTCTCCCTCAACAATAATTTTGCACGGGAGTTAATCAGATCAACAAACAAGTCACAGGGGAGAGGCCTTTATTATTCCAGTTGTTGTTTCAGATGTTGTCATTGACTAGTAGACGAGAGTACTGGAATTCTGGAAACTTACACAACTTAATCCAACCATTGTCATTGCAAATGGAGTCTTTCACAAGGAAAAGCAATTTAAATGGAATGGCTAATATTGTAATTTTGTTTGGAATCA

*S.maritima*23484

AGCTCAAAATATCAGTTCTGTTTTAAGTAACAGAATTGATAACTGAGCAAGGAAAGGTAATTTGGATTATAAAATTTTGCTTTAATAAAAATTCCTTAAACAGTGAAAAAAATAGGCAAAGATACAAAAAAGTTTATAAGAAACAACAATCTTGTATTTATTTGTTATTTTATTTTATTTTATTTTTTCAAAAAGCACTTGAGTACTCGAGTTGCAATGTACAGTACACAGCCTTAGGCAGGGAAAAAATTGTCAATATGTACAAGGGAAAAAGATAGGGAATATAAAGTCATCTTCAAAAAAGATACGTAGTAAATAAAAAAAGATTTACAATCAATCTTTAAATTTTGTACAATTTTTCTTCTCCAATGATCCATCTTGTTAAATGGCTAGGGTCTCCAGCCAGAGGTCTACACATAGCAGAAATGCCAAGATCAGCAGCAATTTCACCATAATCCCATAACGCAGCCTCCATTTCAACTTCAACGGCGGCATCATCAACATTTTCAATCATGTTTCCATGCAGAACATCAGCATAATGCTGCTCTATTTCAATGTCATTTTCAAAGGGTTCCATGGCAGGACCCTCACAGAGCTTGGCATAGTGCTTGTATATCTCACTATTTTCATCAAGATCACCAAGCAAAGTTCCACCAAACCAACCATTGGCTTCATCCACCGATGACATAGCAAGATAACTTTGAAATATATCCTCCTCATTACAATCATCGACTTCAGAAAGCAAATTGAGGTCACATAAATCATTAGACTTGTTGTCATCTTTTAAGGCATCATCTGTGCTTGTGCCAGAGGGCACCTTCAGGGGAGAAGCATTTAGTTCGTCTTTCCTCAGTCCAAAAAGCCAATTTGGACTCTTAAGTTGTATTTCAATTGCATCAGGTGCCACTCCAGAAGCTCCTGCAATGCCTGCAAGCCTTTGGCCTGGTTCACAGCAGAGCCTCACGTCTTTTATCAAGCTGCATGTCCTCTCAATGATTTTATCAAAAGAAGTCAATTTGATCCGAAAGAAGTCCTCTCTACAAGCAAACACGGGCTCCAGTGATGTTTTTACTCCTAGAGGGTTGGCATCAGTTAAGCGCCTATCAGAGATGGCATCGTCTCCAATTCCAACAACATGAAGATAATAGTCAGAATCCAGCTCCCACAGAGCAGGTCTTCCTTCTTTAGGTCTGAAGTAACCCAAAAATAAGTTTATTGCATCCTGCTTTTCTCCATCAGTATAAGCATTGCTGTAGTATCTCTTTATAGAGTTGAGAAACTCCCGCGATTGAGTAGTAGCTTTCCATTTCCCCTGCTTGTCTGGAAATACTGTATTGTGAGCTGCAGAACCACCATATTGTTGTGCAAGAGCATCACCCATACTCCAGTACATATCCATGAGCGCTGTAGCAATGCTACTATCGGGATCCACTTTAGGCTCAACTGACATTTGCATTGCCTGAAGCTGACGACCCAATGCTTCTAGACCATAAGCATATTGTGCAACATTCGTGCGGTCTAAGCAATCAATGCAGTTAGTCCGCAGAACTCCACTCTGAAAACGAGGTTCAATACCATTACAAAAATCTTTCTTCAAAAGCTTGCCAGCATCTAAAACTCGCTCTTTATTCAAAGAAGAATTAAGCAATTCATTGGTGCTTGTACTTCTTGAAAGAGATCCTGAATTGGTTCTATGATCGCTCAAAGAAGCATCCTTAGAACTCAGATTACTGGCTATGTTTAACTGATTAGCTCTCGTTTTGACAACAGGCTTCTTCCCACTGTAATAAAATCCAGTCCTATCAAGTGCTTGCCTTGCTACACCAGCTAAAACTCCCAAAACATTGGCAGTCTTCGTCTTTGCAAATTTATGGAAGTCCCAGTGAATAAAATGAAGATACATTTCCTCAGAAAAAATCTCATTCAAATATGTAACTGCATTTGCAAACTCACGTCGAAGCATCATTTCTCTAGGCCTTTTCTCAACAGTCTTTATTAAGTTAAGCACGATTATCGGATTGCCATATCTCTCCACAAGGTCTTCAAAATGCAATCTGGTAGCCTCATAAGTAGGATCATATCTCTGCAAAACTATATCTGGCTTTGGACTAAATCTTGAAGCCTCTTGCGACCAGAAAAGAGGAATTGAGCCCCGCATCTGCACAACAGAACTCATTTTCCCCTTGTATGAACCACTGTCTTCATCAAGCACAATTTGTTCTGTTTCAACATCATTTGCTACCCTTCCCCTATCGTTTACTCCCCTTTTTAAGTACCTAGTTCCTGCAAAATGACGAGACCGTCTAGAAACCAAAGCAACACTAAAATCACGTCCAAATATTGACATCCTCATCTGCTTAAAGTTCCCATGCACCAGTGCAACAGTCCATATATTGTTTTTGCATCTAGTACGGACAGCTTCAGTAAGATAAGAATTCCACACAAATCTGCTGTCATAATAAATTCCAACTGAATCCTTGAACTGCACATTCTTTTGTAAGCTTTGCATTATAGGATATGTATAGCTGTAAAAGAAATCCTTGGTCAAGTCAACACTAGACAGAAGCTTCTTGTACCTCGACTCAGTTTTAGAGTATGCAGCATCAGTCTGAACAGAGGCATGAGGGATCGTAATCATCTGACTCTCATCAATAGCATAAATAGCATGCCCACAAATACACCCAATTTGCCGTCGTTTTGTGACCAAAATCAAATAATAAGACTCCAGAAACTTAATACAACCTGCAATACCATAAACTTTAGCAACAAAATTCAATCCTCCAGTCGCTCGATTTCCCTCGGCGATCCGTTGCAACAAGTTCTTCACCTCTTGTGATGAATACACAACAGGATCTTCACTAATGTTCAATTCTGACGGTTCACATCGATCAATCTTCAATACACGGAAGAACTTCTTGTTACGATCACTTCCAATTAGATAGAATCTCGCACGAGTTTCATAAAGCCTGAATTTCTCAAGCAAATAAGAGTTTGGATCAACTTCAGAGTCGTTGGAAGGATGAGTTTTGCCAGCCGATTGAGAGGAAAATGCAGGATTGGGAACCGAATTGTTCTCCGGTTTAGTCACCATTTGTGCCCAAAATATTGTTCACAAATCAGCTCAACTGATAAATTGCAGGAACGATGATCTTCAACAGGAAAAAAAAAAAAAAAAAAGCAGTTTTCAGATCAAAAGAATAGGAGAGAGAAAGAAGAGAGAG

*S.maritima*1034

CGGCAGAGATTTGATACCGTCGAAATAAGTACTGACTACTGCATGATTGTGATTTACATAATGTTTAGTAATATATAAACTAAGTAATATCGCCAGTGTACGGTTATAGGGATGATTATCTACAACAATGGTTGAAGTTGCTGATGTGTTCATTATATATCTAAAACTTCGTATTTCTGGTGTTGGAAACAAAATATTTCTCCTGGATATTCCTGCGACTTGAACTTTTTGAAATTTTTGTAAACTGCATTTTCCAGCCATGTTCAAGTATTCATACATCTATGTTATTGTATTCTTGGACCTAATCAGCATTAGCCTCATCATACCAGTATGGGGCACTCACTTACGTTCTTTAGGCGCTAGTCACTTTCATATAGCCTTACTTGGTTCTACATATTCGTTTCTACAATTCCTATCTGGCACACCAATTGGTGCACTCAGTGATCATTATGGTCGAAAGATTGTACTCGTTGTTACAATATGCATTTGTGCCGTAGCTTATTTCCTTTTGGGCTGTGTGAAATCGTTAATATTCATAGTACTAATTCGTGTACTTCAAGGATGTTTGAAACACTCACAACTTTTGTGCAAGACATTAGTTAATGACCAAGTACCTGCTGACCAACAGACTGCAGTTTATGGAAGAATGAATGGATTTTCATCACTCTCATTTGTTATTGGACCAATAATTGGTGGTCATCTAATGGAAAAAAGTGAAGGATTTTATAGCCTGGCATGCTATACTTCCATAATATTTCTAATCAACGCTTTAGTCGTTTATTTTACGGTCCCTAACACAACTGTACCAAAGAAAGAAAGAAAGAGTGCTTCACCATTTAGTGATTTGATGGAAGTTAATTGGAAGGAATGTTGGCCTGCTTTTTCGCTGAAAATGTTAGGAGCTGCAGCGTTGTTTGCATATTTTAGTTCAGTTGGTCTTGCAATGAATGAAAAGTTCAACTTATCCCCATCAGAAGCTGGTTATACTGGTGCTTTACAAGGACTTACTGGTGGCATAACTGGGTTTGCAGCTGGCAAAATAGAAAACCTCATATTTCCATCTAAAAACCCATTTACCAAATGTTTTTATTCCTTCATAATGTTGGGTATAGGATTTGTTAGTTTAGCCTTAGCACCAAATTTAATTATATTTATGGCAGCTATGATTCCAATCAGCGCTTCAAGTACACTCATAAGAGGATTTAACAATGAAATATTGTATGAACAGTCTTCATCTGACCACAAAGGTCTAGTCGCTGGTGCAGGAGCTAGTTCTGCAGCAATAGCAAGATTCCTTGCACCAATTATATGTGGATTTACAATGGACATGTTTGGGAATAACTCTGGATTTTTGTTGTCTGCATTGTTTTCATTTACTGGAGCAGTGGTGTCAATATGTTTAACCAAGAGATCAAAAGCTCATATTGAATAAGTTAGAAAGTGTGTGACAAACAGCAATGCTTTCTAACTATTGCAAATTATAAATATTTGGTTTGCATTTGAAAAACAAAAGAAAAAAACAAAGTACTGGTTTTCAAATTAAA

*S.maritima*34425

CTTTTTGTAAACATTCAAAACCCTTCAAACGCAAGAGAAAGAAAAAGAGAGACCCTTCTTCTTTCTTTTTGGGTTTAAAGTCCTCATCATCATCATTCGTTGATACCTGGATTAGATCCACAACTTAAATATTGACAACTTAACCTCTCTTCTTTTTTTCTTTTTTTTTTATTTTCCAATGTCAGTGAATTTCCTCTGACCAAACTATTTTTTTTTTCTATACAAGTTTCTGAGGGTGGTAAAAGATGCAAACTTGCTGATTATTTAGTGTAAATTTGAGTGAGTCAATACATGATGTTATACTAAATACTAATTTTTTTTGGATTGAAGATTTGAAATTTTCTCATGTCTGGAAATTCAAGTTCATCACCCCGTGTTCGGTTGATGCCTGGTTGCATTTTCATTACTGCAGCATTATCATCGAGATTTTAAGGAAGAGAAATGAGGATGAGGAACAAATACAAGAAACCAACCACTTTGCATTGCAGTGCAGGGAACAGATGTTCATTGCCTGTTGCAGTGTGTAGTGTGGTTGCTTGCTTATTTCTACTGCAGCTTTATACTCTTCTTTCCCAAGAAGATGACTTCACGTTGGATAAACATATACGTACTAGTAAATACATTCGTGAGCTTGAAGAAGTTGATGAGGAGAACATCCATATGCCTCCACCAAGGAAACGTTCTCCACGTGCTATCAAACGGAAACCTAAGAAGGCCACTACCCTTGTTGAGGAGTTTCTTGATGAATCTTCTCAAATCCGGCATCTTTTTTTTCCTGGCATGAAAACTGCAATTGATCCAAACAAGGGGAATGACAGCTTCTACTACTATCCTGGAAGAATATGGTTAGACACTGAGGGAAATCCAATCCAAGCTCATGGTGGTGGCGTTCTATATGATGATCGATCAAAGACTTACTACTGGTATGGAGAATATAAGGATGGGCCAACGTATCATGCGCACAAAAAAGCAGCAGCACGAGTTGATGTCATTGGAGTTGGTTGCTACTCATCCAAAGACTTATGGACATGGAAGAATGAAGGGATCGTGCTTGTAGCTGAAGAAAATGATGAGACTCACGACCTTCACAAATCCAATGTGCTTGAGAGACCGAAAGTTCTGTACAATGAGAAAACAGGAAAGTATGTTATGTGGATGCATATCGATGATACAAATTATACTAAAGCCCATGTCGGAGTGGCTGTCAGTGATTATCCTTATGGTCCTTTTGACTATCTGTATAGTAAAAATCCCCATGGCTTTGATAGCAGAGACATGACAGTCTTTAGGGATGATGATGGTGTTGCATATCTCATCTATTCCTCAGTAGAGAACAGCGAACTTCATGTCAGCCCTCTTACTTCTGATTATCTTGATGTCACAAACGTTGTGAGGCCAGTTCTCATTGGCCATCACCGTGAAGCCCCAGCTGTTTTCAAGCATCAGGGAACTTATTACATGATCACATCAGGTTGCACTGGTTGGGCCCCCAATGAAGCAATGGCCCATGCATCCGAGTCCATGATGGGGCCATGGGAGACTATGGGAAATCCATGTATAGGAGGAAATAAAGTATTCAGAGAAACAACATATTTTGCTCAGAGCACATATGTGCTGCCTCTGAAGGGGCTTCCAGATTACTATATATTTATGGCTGATCGATGGAACCCATCAGATTTGAGGGACTCACGGTATGTTTGGCTGCCTCTGAAGGTTGCAGGGGCAGTCGACCGGCCTCTTGATTATGACTTTGGGTTTCCATTATGGCCTAGAGTGTCAATTTTCTGGCACAAGAAATGGAGACTTCCTTTCAAATGGAGCACGTAATTAGATTGTACGTAACATAGTTGAGTTGTCCAATTCTTTATTCGATCGATTCTCTTTACTATCCTGTTGTAACAATAATATTTTTCCTCACAGTTTTAAATTGATTTTTTAGTTATAGCGTAGTAGTAATAAATTAGTGAACTCATGTTTATATGAATTAAGCGAAACTGTTCACTTCTGCTCTATAGAGGAACTAATGAGATGAGATGTTTGAGGTTTTCAGATATTGAAGCTGCTTACTATTTACTAGTCAAAAGATTCATT

*S.maritima*20865

GTAATAACATTTTGGTCAACAGGATTACATACATACATATATTCATTAACACCATCCAACAAAACAAAGAGAATTATTAGTAGAAAAATTCAAAGCAATAACAAGAGTTTTGATCCTTCAATCAAGCACTTAAGAGAACACCCTCAGTTTCCTTCCTCTTACGCAACTTCCTCTGCTTCACACGCCTCTGAATACTCTTCACCACCAACACGATCAAACGAATGACATTCAGAGCACTTGCTATTATCAGCACCAAAAACAGTATCATCATAACAACAACAGCAGAACCTGGCTGATAGTATCGACTCTCGTTCGAGAGCATCGATCCCATGTAGTTTAGACTCAAAAATCCAAAAGCAGTGTACATCAACTGTATCACAAGCCAAGCACAAAACTTGTTGCTTAAGGGCAAGCCACCTAAGAGTAACAAGATGACTGTAACAGCAGGCATAAATGAAGCCGAGCTGACCCATAAGTGATCGTCTATGACCGGTCCATTAGTCTTCATATAGATGATGGGCAATGCATCAATCAAGGCTACAATGGCCGCGGCTATTAGTATTTCACCTTTCATATCTTCCAATTGTTTAGTTATACCATCGAAGGTGAAAATCGTTTTCCATGATTTCTTAGGCCTTGTTGATGGTCTAGGAGCTTTTTCTTGGGTGCTAAGTAGTTTTGATGGTTGAATTTGTTTGGTTGGAAGAGATGATGAGTTGTTCTTTGTGGAGTTTTGAACATTGGATTGTAGGAGTAAGTTGTGTACTTCAAGGCCTTTCAAGTCTTTAGGCGAATTTTCGATCAAATCTAAGGCTGTTAGACCATTGTTGTTCGCTGCATTTAGTTCAATACCTGGTGTGGCTAGCAAGTATTTGGTGATCTCCAAATTCTTGTACAGAGCAGCCAAATGCAAGACAGTATTACCATCATCATTTCTTGCATTGAGAAAGTTGCTACCTTCACCCTTCAACACTTCCACCAACACTTGAAAAGCATCCAAATTTTCGTACTTGATACACAAATGCAAAACACTTTCCACTCTATCCACCTGAGACCGAGTTGATTCAGGACACGCCTGAGCCAGCTCGGTCACTACCTCAACTCTGACCCCCTTCATTATAGCCAAGTGAAGGGGTAACTTCCCATCCTTGCTTCGAGCCAGGCAGGTGCCTTGACTCACGCGCAAAAGCTCGCGGACAATCTCAACGTGGCCCTTGGCGCAAGCAACGTGGAGGGGCGTGTGTCCAAGTGAGTCATTCTGAGTGGCAAGCCGAGGGTTGTGACTCAATAGCGCCTTTGCAAACTCATGGTGCCTGCGCAGGATTGCTACGTGCAAAGGCGTATCTTGGAAGAAAGTCGGAGAAATTCGATCAAGAATCAAGGGATCTTCTTTGATTAATGCATTCAAGTTTGATACATTTCCTTCAAGAGCAGCATCATAAAGCTTCTTTTCCATCATCATTATCTCTCTTTTTTGTATTATAAG

*S.maritima*44753

GCATTTCTAGCCAACTGCACAATCTGAATTAGAAATGAAAAAAATACGTAGGTCACTCTGTTTGTTTGGTCTTCCATGGAATCCACCATATGCAGCTTGTCTCCTTGGAGAGAATATCCCATCTATCTTAAAGAGGCAATGGCACGCACAGTAGGTTGCACCACGCTCATCCAAACTCTGCACTTTTCATATCCTTTGTAATCTCCTCCATCCATGTCACTCACTCTTCAATCTTCCTTAGCTCCTGGATGTCGAGTTGAAGACAATGTCTGACCCCCTCCGTGTTTTAAAAAATGAATGAAGAGCACTCCAGAAAGTATCATTGGAGAACAGCTCATGCTTCCTTGAGTGTGAAGCATCATCAAGCTTCCCTGAACTTCTATCTACCAAAATTGGTTCTTCACTGGATTGTTTAGTTGGTGTCTTCACAGATGAATTCCTCAAAGATTTGCACCAGTTGATTACCCTTGTAATGAACCCTGGACTAGCTTTGGAATCATCAGAAATGTTACTTTTGGCATCCTCATTCAATGCAGCACCAGAAGAAGGATTTACAGGCTGTTGAACATTGTCAGTTACAGCATTGCTGGAAGAAGAGTGTACTCGGTGTTGAACATGATATTGATTGGTTGGATTAGTCTCTGTTTCTGAGTTAGCAGCAGGCATGTCCAATTCAACAGCGGCCTTATTTGTCTTCTCATCATCTGTGTCAGCACCAAACAAATTTGTCCACAATCTTTTGAATAGCCCAACATCACTCTTAGAATTTGGTTCTTCCTTACGAACCCCAGAATTATCAACTCCTTCTATATTAAATTTTAACTTATTATCTGGAGTCTCTTTAATAGCCTTCTCTTCCACAGTTCTCCCTGTAAAATCAATATGAGGGAGCACGGACAACTGTGGAACATCCACCATTTTAGTTATTGTGTTAGTCTGATCAGTTGCAGCTTTGGTAACTTCAATGTCTTGGTTTTGCTTCTCACTCATGGACTCAACAAAACTATCAGTCTTCTTAACAGCTTCTTGAACTCTTTGAGATGCAGGACGTAAGAAGAACTGACCATCAACACAAGTATCATCAACCTTAATAATGTGGGGCATTGACTGCAGAAAGCGAGAAAATTTTTTATGGCCAAAGAAGTCCTTATCAATAGGCACTTTATTATTTTTCAGCTCAAGTCTAAAGTCTGTTATGGATATTCCATTTGGATATGCACTAAGAATACGACGTATCTGCCTCAAAACTGACAATGGCACAGAACGATGCCTTACTGACAACTCAGCATCAGTTGAATTACTAGCCACCGCAGAGCTCTGTGGCTCAGGTACATTAAATGGATCTTCAAGCAGGCCTTTGTAATGGCCATACCAAGAATTGAAGGGGCCATCTGGTGGCTGATTTAAAAGCCTACCAGAGAGGTTTTCCCCTTTAACTAAATCATTCCAATGCCACATAATGCTTGCAGCACTGCAGAGGACACCACGAGCACTTTCTTTACCAGCAAGCAGTATATTATAGTTGTTCATCCTCAATTTGTGTAATATACCGGCAAAATCCCGGTCACTCGATATCAAAAAGATATGTGCAGGTGGAGGATTTTGAGATACCCAATACATAAGGTCCACAAGAAGGGACCTATCAGCACTGTTTTTTCCACCTTGAGGAATGTGAACAAGATTGACGCCAGTACTAGACAAAGCTTCTTGATTAGGTTTCGAAAGGCAAAAGACATCTCCAAAAGCAGTAATTTGAAGAGGACCTTTAATACCATTAGCTCTAACAGCAGAAGTAATTGAATGAGTAATTTTGAATACATTCACACCATTTGGTACTTGGCAATTCTCAATATCCCACCACACTGATACTCTCACTGTCTTGCTTTCCTCATCGTGCCGCCGAGATGGAGGAAATGATGATGGTGATGAAGCTGATGATGATGATGATGAATAATTGGCTGAATATGATGATGATGATGATGATGA

*S.maritima*43401

CGATAGCACAAAGAGAGGCGAATTTCACATTCTGATTACTGAACACAACAAAGGTTGTATTGGCATATATAAGACATGAATCCATTTGATACAAACACAAATCAGAAAGAGATCAAACCCCTTTACAACAACACCCATTTATAAGCATACATAAATAGTATGAGCAAACGCAAGTACACAACACAACCAAGGCAACATTACAAACAAGCAAATGAAAAGAATATGTGTTAGTGAATGCAATCATTCATATTACTCATCTTCGCCTCTCCAAAAATTCAAAGCCTGAATCTTGTTCTTACTTTGCTTAGACAAAGGTAGTGATATATGCACAAATCCAAGAGCTTTTCTCTTGGAGAAAAAATTGCTCTTTCCTTCACTACCTTCTTTCTCTCTTCTCTTTGGTGGAGACACCTCTCCTCTATGTCTTCGTAACATTTGGTTCAATGACACCCTTTGCCTCCCTCTTCCACCACCATCGCCATGACCTCCTCGTTCTTCATGTCCATGCCTACCAGTACCAACCTCTAAATCATCCCTCAACACAAGTTTCCTGCTCAATATGTCTCCTATCGAAGTTGTTGTCTGCCTTGCAACCGGCTTATTACACTCGTTTCTTGTGAATTCAAACTCGTTTGAATCGAGCAACCTTGAATCCCTTCTAGGATTTGTAGGAAAAGAGTTCCTAAAACCATCCAATTCTTTTCTCTTAAACATGTTTTCAGCTTTCTTAGCTTTGTCTGTCAATGCAGAATACTCCTCTACTGATAGACTAATTCTACCTGGCTTTTCCTGTGTGAACTCATTGCTCGACAAAGCTCTGATCTCAGCTAATGCAAGTGACTCAGTAGCTTTAGCAGCATCTTCAAGTTTCTTCGCAACAACCCACCTCATTTCGATCACTTTAAGGCCGGTTTTTGTCTGCTCAACATCTGACATTGCTTTCAGAACTTCTGATCTTGCTGCTTCCGCTGTTTTCATAAACTGGTCTGCCTCATAATTCAGCTGATGAAGCTCTCGTGAGATCGCTAATGGGTTAACAACCGTTGCTCCACCCATGCTTACAGTATGCTCAGTTTTCATTTCCTGAGCTTTTTCCACTGAGGCCTTCTCTGTTTCCAGTTTCTTACTGAGTGCCTCGACAGAAGCACGGAGTTCTGCAAGGTCATTTGTGTTCTGAAACAGGTTGTGCTTTGCCTGCTTCAGTTCCGTCATTATCAGTCCAGCAGCAGATGACGGGAGTGAAGGCTCATTACCACTAAGTGGTGGATGTTCTTCATGACTCTTGTTTGTGGAATCAATAGGATATTTGTTGACATAGTCTTTGTTAGGAGTTTGCATTTTCCAGTCAGAGGTTAGGGTTGGAATTGAAGTGTGATGATCAGGAGTTTCTTTCGGAACTTTGAGCTTCAACTCTTCTACTAGCCTTTTAGTAAACTCTAGCTCTTTCAAGATTTCATGGGTCTCTTGTTCTTTCCTCTGCAGATCTTTTAACATAGCCGCTGCTCGATCATCTAGTTTATGAACATCTGACTCTTCAAGATTATGATGAGGATGATCAGTAGCAGTAGCAGCATCAACAGCAAGAATGTGTTGTGGAATCCAAAACCCACGTCCACCAAACCGAATCACAGCTTCTTCAACCGACCCGAATGGCGGAGAAGTATCCACCATCACTCTCGGGCTTGAATTACCCATCATTTCATGCACGGTGTATGACCCGTCGTCATCTGACCCGTAGTCTGAAGTTTGGGTTGCTGAGTCTACTGCAACCCCTTTTCCTGCCATTTTTAACTACCTATTTTTCAAATATAGGGAGGAAATTGATAGAATAACAGAGAATGAAGGGAAGAAAGAAAAAATGGTGAGATTTTAATCAAGGGACATTGAAAATGTGTACACGTAAATAAGGGATATTCATATTATAGTAGGAAGAAGAAAGGAGTAATTAGAGGAAGGAAAACAAGTAAACATGGTAATATTCTAGGTTAGAGGTTGATATGGTTTG

*S.maritima*33718

TCTTCATCATCTTCATCATCATCCCCATCATCTGCGTCATCTCCAGACATCTCATCATCATCATCAAGATTTTCTTGCCCATCCGCTTGAATTTCAAACCTGATTCCCACACTTTCAACCCCATTCTCAAGGCCCCTTCCATCCACTGGTGTGTCATGCATGTAATCATCTTCATTAGGAGCAAAGCCACCATCAAGATCCTGGTCATGCTCCATATCATCAGTTACAGCCTCGGAAAAACCAAATGCATGCCCATCATTAAAAGAATCACCGCGGACATCAGTGGCAGAGTTATCATTCTCTTGAGAAGCAGCTTCCATAGCTTGAACGCCATCACCACTATTGTCAGTTCTCTCAAGATCAGAAGGCTTGGCAGAATTGTCATGCCTACCAGCATTAGCATCAGCAGAATGGATATGCTCCTTAGTTACCAGTTCTAATGCTTTAACTAGTCCAGTGACGACATTAAGTGAGTCGGCCGAGTCAAGGTCAAGCACTTGCAGAGTTCGGGTAAGTGATTTGACTAGACCAACATCTATGAAAGTGGCAGATGCTTCTGAGGCGACATAAGAACCAGTTGGGGATCGAGCACCAAGTACCTCGTTAAGAAGATCAACAAAGGCTACAATATCATTGCCAGGTGAAATGAAACCCTCGGCTGATTCAACAAAGTCGCTGAAAACTGAATTAACCTCTGTAAAAATTCTTCTCCTAGCCTCAGCAGAACGCACACATGCAGCTACTAAAAATTGATTAGCCCTGCTTGCAAACTTCTGTTTCCAATCACCATCTAATTTCTTTTCTTTCTTGGCTTTCTTACAATATGGAACAAACTTATGCAGAAGATGGTGAAACAATCCCCCAGAATTGTGACCAGAAGTACCTCGTTGTGGTAGACCCCTGCAGCTGCTTAGTTCAACATCCTTCCTTAACAATACATGAACAGAAGCAGCATATGTGACAAGAATCTCTGTAAGAAGCTTCAGGATAAAAACGATTTTGGCCAAGGAAGCAGAAACATCTTGATTTTGGCCTTCTTTTTCTTCAGACTCGCTTGCAACAGCCTTTCCTTTCCCTTTACTTGCAGCCACATCAATGTCCATGTTAGTTGTTGAAAGTGAGCCAGAGGCTTCAGGTAAGGAATCATCTTTCTTAGGGGGCTCAAACATTATAACTGTATCTAAAAGAAGCTCTATCACATTGACAAAGCTATGAGGAGGTTTTTTGTGAGCCTTGAAACTCTTGGACTCCTGAAGTTTCCCTTGTCCACCTCCCAAAGCCATAGGTGCAACAGCTCCAGTAGCAGGCTTTACATCAGTACCCAATGACTTTTCCTTCTCCTTGTCCTTGGATTTATCCTTATCACGATCTTTCAGCAAGACAATATATGGCCTGTCACCAACCATCTCAACTTGACAAACCGACTGAGCAGCTCGCATGAACACCACTGGATCTCTAGAAATTACAGGTGACAGATTTACCAAAAAGTTGCGAACAGTAAGCCTTCCACTTGACTGTCTGTTTGTTGCAGTAACAATGTTGTGGCGAATTTCAGACTCCATTGCCTGCTGAAGAGTCTGAGGATCTTCAAGGATATGACGAATGATATTAGCAGCAATATTGTCAAAACCAGAGAATAGGCTGCTAGTTGGTAAATTCAGAAGCAAAGGAAGACCCCCAGAATCAAGAAAACTAACAGCAACAGTGTGGGTTCTAGTAAGTGTAGAACAAAGCTGGAGGACAGCATGCATAGTTTCAGAGGGAAATTGCTTCCTTATACAATTACAAGCAATCTCAACCAGCCTCTTCTGCTGGTCTACATCAACATTCTGCTGTGATGGCTCCAGTGCCTTGTTTTGCTTATCCTCATCAATAACAATCGAGGCCTGCTGGCTAGTCTCCGTGTTCCTTTTCAACACCTCTACAACCTCAGGATTCAGCTTCTGTTCAACTTGTAGCAAGCGATCTACAGCAAGAAACGCAGCAGTTACCCATTTTGGAACAAGTGCATCTGTCCCACTAGATGATCCGGCATCCCACTGCCACAATACATCAGACACAAGCTCTACCAAACCATTTTTCAGACAAGTTTCTCGAGATGCTGCATCTTCATGAAGAATCAAAGCAAGAACATGAAATAGGGCAGATAGCATAGAACTATTCCCTCGATCAGTAACAGAATTACAATGCTTTATCTGCTCAATAATAAAGGTAATGACATTGGGTCTGTATTGACCATCATTCTGGGAGCACATTGTCATGAGCAAGTCTCGCACTGGAAAAGCAAGAGATTCCTTTGATTGCAAAAGCTTCACGCAAGTCAACAGAAGCTCATCAATAGGCGGGAGTTTTGCCATCTCTTCTTCAAACTGCTGACTATTTTCACTGGCAGCTTCCTCTTTTGAGTCTGATCCTGAACTCCCAAGAGACATGGCAAGAGCACGAGCAAGTTCATCATCCTCAGGAACTTCCTCTTGATGAGTGAACAACCACTCCATTGCTAACTCAACACTGTTAGCTCCAACTTGCCTAAGCGCTTCTTCAGCCCTGGCCCTTGAAAATCCCATTTCTACAATTGTTGAAATAGCTGCTTCATTGGGAGGTGGACCTGGTAAACGACTGCTACTAGTAGTACTTCTACTGACATTTTTTACCTCAACCCCTGAATAAATATGACGGATAATAGAGATGATTGAAGTAATGAAAGCATAGCTGCAGTCAACAAATTGTGGGTCGGTCCATACAGGAAGCACAGCCTTCAGAACCATTGACTGTAGAACCTTCACAAATGTCTCAGCATCTGGAGGGAACGGAGTATTGTTATTTGGCAAAGGTTGCACAAGCAAATGTTTTGTAAAGGGAGCTAATATATATGATGAGGTCACTAAATGATCCATCAGCTTCCCATAGCTAGCAAGAGGACCATACAGCCATGAATGCTCGGGCTCTTCCTTTTTATCATGCTTCAAATTGCCATCATCAGTTTCCATAGGAGATGAAGGAACAGTAAACAACAACTGGCTTGTAGCTTCAAACGTAGTCAAAATTGACTGAATAACACCTTGCCCGTATAAACAATTTAGTAGCACTGGATTACAGGAATCATGCCTGTCCATTAAGATGCCATCAACAAAATCCACAACCTTGCCAAAATAGCGGCACTTGCTAGATACTGATGCTTCAGATCCAGAAGGACTCATATGACCTCCAAAATTCATGTGTCCCAGGCAGATAGAAGCGAAGGAAGAAACTACTGCTTTTGAAGAAGGTGATATATTGATGGCGTCATCACGTCGCCGAGAAGGTAGCAGCATTGACTTCCCCAACTCTTGAAGCAAATGCATGATGTGGGATGCAAGTGATTTAACCATTTCACAACAAGATGTATAATGACTCCTCTGTTTGTCTTCCTCCTTTCTACTAACACTATCTGTGTCTAAACCACCAGACTCACGTGACTGCTGACTGCTTCCCAGCCGTAGATTCAACCCATCAGATGTACTTCTCTGAACACCACTTGCACGACCAAGATCTCTATAAAGATTAATAAGATCAAAAAACTGGGATTCCACACTCCATCCTGACATCCTCCTCCTGAACAATGGATCAAGCAACTGCCGAAAAGACGTTACTCTCTGTTCTTCAGCTCCACTAGAGCTTGAAACCTGCTGTGGTTCTGATGCAGAAGCAGGTCCTGCGCCTTCCTGTACTTCAACTTTCGCATCCTCGAGTAATGCAATCTGCCATAGAATTTCGCGATGCACTTTCCCAATATCCTCCAGTACATCTTTACTTTCACCACCAAACTCTGTAAGTAAAGCAGAAATCCACCTGCTTTCCTTTGCAGCAGCAAGAAACAGAAGAAACTCAACAAGAAATAAAGATGAGAAAACTTCATGATCAAGTTTTGTCTTAGAATCTAACAAAAACGACCCTGTGACCAGACTAAAACCAGTCAAAGCTTTCTTCAGGTGATCTCTTAAGAATGAACAGAAAGCACGAGCCAACGGGGCTGAGTGATGCTGGGTAAATCCCTTGAACACCATAGTGCTGTGCAAAGCAATAGCCATGCCCTCAGAAGATTGAGTAATACTAGGCCGCAACAAAAGCTTCAATAAAGCATCAATACCAGACTTCTCCACAAACAACCTACAAGTTTCTGAGTTCTCCATAGTCCTATGAACCAACACCATTACATGGAAGACACTTAAATTAATGAAATGTTCATCTGTAATACCCTCCCCAGCTGAATCTGAGGAAGGGACTACACTAGAAGAGCTTTCACTTCCGCTTCCTTTATCTCCAGACTCCAATTCCATAATTTTACCCTCATTTACCTTCTCTGAAGACCCAGAAGAACTTCCCTCTCCAAAAGCAGATATTTTATTGATAATCTCAATAATAATATCAACCCCAGTGCTTCTAAGTGAAGATACATGACGCAGCAACTCTTCCACACCATTAGCTAAAGGAACAATAGCTTCATTCATAGCTACAACATATTTCTTGGATGTGAAAATTTCAAGAAGAAAGCGCAATGCAGAAGCTTCTTTCACAGCCTCCAAACCTTTTGTATTGAGGCATATGGCACCCAAACCACTAGGCACACAAGTGAGAGCCTTTGAAGAAGGAAGTACACCAGCAACAACTGAAGATAAAAATGCATCAGGAAGACCCATTTCATGTAGTATGGCAAAACAAGTGGGATCTTTATGGAGAATTTCACTCATAACAGTAACAGCTGAGCAGTAAATGTCGCCACCAAACTTCTCAACATTACCAAATATTAATGACAATGTGGATGGCAATGTACTATCATGAGAACTCTGGGAGCTGGATGCATTTAAAGGAGCATATGTTGCTGATCCAAGAGCCTTCAACAGAGCCCTAATGAGCCTTTTCTGACCATATAAATAATCCTCAGTTGAAACATCGGCAATAACCATCAAATTATCATCTGACTTTCCAGTACCTATAACTCTCTGAACTTCTATCTGCAATCTCTGGGCAAGAAGTTCAACCCCTCCAGAATCTTTCAGAAGTGTAACAGCTGCATTACTGTAGTCCATAAGCTTCTGAAGCGTTTTAACGGCATAGCAGACAAGGTGCATATGTGCAGGTTCAGAATCCTCCAAAAGAGGCAAAAATGTTGGGACCATTCCTGAGCCACGTATGATGCTGCCAGAGCTTGATGTAGATATCACATGCAGCAGATAGAATTGCAGGAGAGCCTCGACGAAAGCAAGAGAAGATGGATCATTAGAATTCTTCAGTGACGAGATTGCTTTTTGAAGTACATTCAGAAGAATCATCCGATTTGCTCCAGCAAAATTAATGCTTGAACCACTTAACATCCTAGCCCGATCATGAGATGACGAATATGCTGCCAACTGAGCTCCCAATGCAAGCATAGCAAGCGTTCTAACAGTACCAGATATGGTTTCCTCAGATCGAACTATCTTGATCAACTCATTAGTATATTCAGGCTCGTTAGCAAAAAACGACACAAGCTCATCATTTGCATCATTAGACTGCACAAGCACAATAAATGCTAGAAGACAAATTCTACTATACAATCTGCCCGACTTTGGAGAACGGAAGGCATGTGCATATCGGATTCTTGTGAGCAACGAAAATCTCTGTTCAGGGGGCACATTGTTCTGATCAATGCATTGCTTAAGCAGCAAAAGATCATCCTCTTTCCGTAAATGAAGGTCTGGCATATGGATCATGGTCGAACTAGAAGGCTTCACATCACTACTGCTTTCTCCACTTCTTTGGGGACTAACTCCATGCAATTCAAAATACAATGTAGAGCCAAGACGAGTTTGAGACTTCTCATCTCTGTCTGGTGCATCTGAAGGAAATAAAGATAGCCCCTCTTCTTGACTCTTTTCATTGGCCACAACACATGAATACAGACCAAGACCCTCTTCCTTGCTCCCCCATCCCTGGGCTAGAGATAACAGGCAACTATTAATTGAGCCAAGTCCAATCATCTTCCCACTAACATGCAGTTTTGAGGGAGGTATTTTCACTAGAGCAGAAAGAGTCTCTAAAGTTGCCACAAGAACCTCAGGATCTGTCGATGCAAGCAAGAGCTTGAAATGCTCTAGACCATCAAATGAACTCTTATTGTGACAATTCTCTAGGATAATTTGCATCACCCTCAAGATCTGTAAAACTGCATGTTTTGGAAATGAAGCATCATCTTCTGTAATTGTATCTGATAGGAGGAGATCATTCCTCTTAGCTATATATTCCTTGAAATATGTATCAAAGTGATGAAACAACGGCCTCCAATGATGAAAATTCCCCTTGTTGTACTCCCAACGAAAACCAGACAGAGGAATTGCCAAGTCTTGCAGTGGACTCTGAATAACTTTGTCAATAAAAGCTTTAATCTTAGGAGGCGTTTCAGAATCAAGTTGAATAGAAGGTCCAAACGATCCTTCGCTACCGAAAAGTTGCCGAAGCCTTGAAGGTAAAGTCGATCTTAAAGTCGCCATTTTTTTTTTGTAATCTCAAACTTTTTTGATTAACTTCTCAAATTCTTCGAATAAGTCAAACGAAGAGTACCAAAGAAAGAGAGGAGAGAGATGACGAAGAAGAAGAAGAGGATGCATACACATCCGACGTTTCGTTTCTTTCGACTATACAAGAAAGAGAGAGAAAGAAATATGGAGAGAGAAAGAGAGAATTCCAAAGCTAAGAAATGTGAAGGGAAAAAAT

*S.maritima*30120

CCGAAATAGAAAGTTACAAGAATGACTTAGTTTAAGGGTAAAATTGAGAAATTTAAATAATATGAGGTACAATTTAGAAAACACAAAATCTTGGAAGTACAGCTTAGAAAATCCAAATATCTTTTAAACCTACTCCTAAAGAAACAAAAAAAATCCCTCCCAGAAAGAAGCATTTTTTATGTGCATAATCATACCAAGTTGAAATATATATAAATGGTTCCAAAGGTTTTGACATCAAAGAATACAAAGTTAATTACATGCACCTTAATCACCAAGAATAGAGAGTGGCTGCCGTATACTAAGCTCAGCTTTTAGCTCTGGGTATTGCTTCAGATCATGAGACGCGCTTAGTATTTTCAGTGCAGCATCATGAGCATTTTGAATTATAGCTCCATCAACTTCAAGTCTAGTAATAGGAAAATCCGGAAGGTGTCCTGATTGTTTTTTACCAAGCAAGTCACCAGGTCCACGTAGAAGCAGGTCAACTTTTGCCAGATGGAAACCATCTGCTGATTGCCCCAACACATTCAAGCGAGCCAAACTATTAGCAGTTGACCCAACTAATATACATTTGGATTTCCGAGCACCACGTCCAACTCGACCTCGGAGTTGGTGCAACTGTGCCATCCCAAATCTGTCAGCATTAATAACCACCATCATAGAGGCATCTGGCACATCAATCCCTGTCTCTATAACTTGAGTAGAAAGAAGAATCTGAGTTTCTCCATCTCTAAAACATTTTAGAGCTTTTTCCTTCTCCTCTCCCTTCATTCTCCCATGTAATAACCCACACTGATAATTGGAGAACTTGGCACATATCGTAGCAAAATCTGCTGAAGCAGCACGGAGCTGGGGAAGTTGCTCAGATTGATCAATTATTGGGTATACAAGATAAACTTTGCCTCCGGTTTCCAATTCACGGAACATCATCTTATAAACCTGCTCAAAACCATTATCATCACCTTCGACAATACATGTCTCAATAGGCAATCTTTCTGGAGGCAAATCTGTTATCTGTGTTAAGGACATGTCTCCATATAAAGCCAAAGCAAGACTCCTTGGGATTGGGGTAGCCGTCATTGCAAGTACATGAGGTGCCATCTGGAACTCACCTTTCAAGGTACTACCAAAAGCAGCCGAACCATTCTCAGAAGTTGATGCACAACATAACTTACTATTGAATCTTCCTCTCTGAATTACACCAAAACGATGTTGCTCATCTATCACTGCAAGTCGCAAAGCAAGAAACTCCACACTCTCAGCTATCAAACTATGGGTTCCAATTACTAAAGAAATAGTTCCTGCTTGGAGTTCATTGCGTATCATTCGTGCTTGTTTTGTTGGAGTTGAACCCGTTAGAAGAGCAATAGTAGGTCTGCATGCAGACTCCAAATTCTTCAACATATTTAATAAATCCTCATAGTGCTGGATAGCGAGCAACTCAGTGGGAACCATGTAAGCTGCCTGATATCCTGAGCCAATAACTTCCATACATGACAAAAATGCAACAACTGTCTTCCCACAGCCCACATCACCCTGCAGCAGCCGACTCATGGGAACTGGTCGCTTCAGGTCCAAAATAATTTCTGACACTGCACTTAGTTGACTTTTAGTTAATGAGAAAGGAAGTGCTTCTAAAATACTCCTGGTGAGGTCACACCACTCCTCAGAAATAATAGAACTTTGTCCTATATTTCTGTATCTTTCAAGCAATCCTTCTTTTTCTATTCTTGACCCAAGACCTTCAAGCATTTGGTACATGCGGCCCAACTGAAGGTAGAAGAACTCATCAAATATAAACCTTTTGCGAGCCAAATCAGCATCATTTAGATCCTTAGGCTTGTGAATCGCTATATATGCGTCACAAAGCCCCAAAAGCCCAATGTCTGCTGTGATATCTCGAGGTATAGGATCAACATTAGCAGGCAATGACTGCAAGGCTCTCATAATAATGTCCCTGAAAAACTGAGGTTTGAGGCTTCCCTTCGAAGAGTATATTGGATGTGGTCTACCCTTCACTGGAACAGATGGATCATGCTCATTATCAATCACATCCAAGCTATATTCTCTCATTTCATAGTGGTCTTCTTTAGTGGTGCCTTTCACCTTGCCACTAACACACACGATTTCACCTTCATTATGCTTTTTCTCAAGACACCGAAGAAAACCTTGGCTAGTGAAACGAGTGCCACGGAAAAATTTCTTCAGATGTAAATAAATTAGCCTTTTTCCTTCACCATTTTTGCAACCCTGTTCAAGATCATCATTTTTCTTATAATCAACCTCACCATGCACAATGATCTCGAAAATTGAAAGGGAGTAGCTAGCTTTAATAGCCCTGGCGGACAAGATCTTCCCAACAAAAATCAAGTACTGGCCATCCACAATACCCGTATGTGAGTTTTGTAGATCAGCATAGGTACGAGGAAAGTGGTGCAACAATTTGCGCAACGTATGGAAATCACATTCTTCCAGCTGACGAGAGTGTCTTTTGCTCAACCCCTGAATGAAGTTAATAGAAGTATCAAGATTCACCTGACATAATTTTTCCTGTTCAAGAATCATAGGCGGAAAAGAATTTTCAGAGCCTAGAACTTGAGACTGCTGATCTTCCAGATCATTCTGATGTTGGATTGATGGTGGTAGAGAAGCTTCTTCACTTGAGGAGCTCATATCTGGAAGCTCACTATTAAGAGGACTCATTTGCGTAAGAATCTCTGGAATCATCCATTTCCCATCTGTCAATATAGGTCGAAATTCGTCAGAGCTTAGAGCTGGAGAATACGTCTTTACTTGAGATTGCCAAGGTGAGTCGTTATATAGCTCCAATACAGGTGAACTTCCCACAGTGATGGATGGAAACTTTTTGCGTGTGAGAGAAACATCAAAGGTTGCTAAATCATCAAGGTTGCTGTAACCCATCATAACAGATACCTTATCAATAAACTTAGACCGATCAGCTATGCTTCCAACACCATAACCATCCACTTCCTCGAGTAACTTTCCCGACACCTTATGCTTTGATCTGTAGCAGAATTTCGGTATCTTTGAAGCCAAAATCTGATTATATCTCATCCTCTTTGTCAAAAATTTGTAATAACCTCCTTCAGCATGAAAGAAATTTGCGCTTCTCATATAATATTCTGAGAAACTAGAAGAAATCATCTTGAATTTACGTATCGCTTGAAATATGAGAGGTTTCTGCAAGGAGTAATGGAGATAGAACCGTAGGTTGAAGGAAAGAGCATTGAACTCGTTGTAAAGCAAAGCTATAAGCCTAATAATAGGCTTAATAGCCTATGGCTATTCAAGTATAATATGCAAACAGAGAGTGAACGACACTTGGTGAAACTTTGAAG

*S.maritima*602380

CAGCGTCAGATGCTTCAAAACTAGCATAATTTATGAATGCAAATCCTTTCGAATTGCCTGTATCAGGATCCCTCATTATCTTTGGGGTTTGTAGAATAACACCGAAAGCAGAGAAAGTGTCATATAGCAATTTCTCATCAACTTCTGAATCCAAATTACCAATGAATACATTGGCACCAACATCAAGGTTCTTTTGATGAGCAGAAGCCTTATTCACGCGTATTGGTTTTCCATACAATTTTATCATGTTCATTATTTTAATAGCATAATCGGCATCATCTTCAGCAAGAAATTCAACAAAACCATAACCTTGATGTGACTGTGTCACTCGATCTTTTGGCATATGAACATTAACCACTGGACCGGCTTGAACAAACAACTCCCACATGAGAGTATCCGAGACTTTTTCATCAAGACCACCAACATAAATCGTTGCATCTTGATTTCTTTCTGCTATAGGTCCCGCGGCCATCGTATAACTTCAAAATTGCATTTAACGACCGGCAAAATTATAAAAACTAAAACGTTATCGAACATATAAAATTGAAAAGTTGTATACAGAT

*S.maritima*40612

TTTTTTAGTACTCAAATAGAAGATGCATATTTTCCATTGCTTATATGAAATAGTTAGAGTTACAGCTTACAACAAAACAAATTTCTCTCCTCGCTTCTTGCCATGACTAAACTAATCTGCTATATAGTTGTATTCAAAACTCAATGAAATTGACATTTGCGAATTGTGAATTTGCGAGTCACATTTCAGCCTCATGTTGCCATTTAACTGAAGAGAAAATAAGCAAAATTTACATACTCAAGCTCTACCTACTCACACTATAGTCTCATACTCTATTATCTCCTACTGAATAGAATTCCTCAAAATCCAGGCAACGATGTGAGGTCCACGATCCCCCTCGGAAGGTCCGCTATCTTCTTAAGCAGAGCAAGCCTATTATTTCTAACCTGCTCATCTTCCACCATGACAAATACGTTGTCGAAGAAATCAGCAAGTGGTTTTACTAATTCTGAAGAAGCTTCAACAAATTCATCAATTTCAATACCATCATATACTTTGTTATTCACCGACAAGAACGCACTCCACAAAGCTCTCTCTTCCTTTGTCTCAAAAGCAGCTTCATTGACCTCCGTGTTGACATCGAGGTCCTTTCCTCGGACAATCCTTGTGGGCCGAGAATATGCTTCAACAACCTTTGGGAGGAGATCATGTTTGAACATGGCTTCCATCTTTACTGCTGATTTTGCGGCCAGACATGGCCAACTACCACGCTCTAAGAGGACAGACCGGACAACTTCTGGATTTATACCCTTGTCAACCAGATATTGTTCCAGTCTTCGTGTTACAAATTGTTGCACATCATCTATAACGTTGCTATCAACTTTTAAAGGCTGGACCTCAGCAGCTAGCTCCAAAGCACTTCGCAAGTTTATATCTGTATTATTTTCCACCAATAGTTGAACAAGGCCATATGAAATTCTCCGCAGACCAAAGGGATCATTTGTTGAACTAGGCTGACAACCTGCCCCAAATAAACCAACAATGCTGTCCAGCCTGTCAGCTAATGCAAGAGCATTTCCAGCATCTGACTTAGGAAGTATATCCCCTGAATTCCTAGGGAGTGTAATTTCAAATATAGCCTCAGCAACCTGGAATGAGTAGCCATCTCTTAAAGCATAATGCCGACCCATTATGCCTGCCAAAGAAGTAAATTCAGTCACAACAGCAGTAGAAAGATCTGACATAGCAAGGGAGGCAGCTTCTTGAACAGTCTGAATTTGCTCTGATTCAAAACCCAAGGATGACGCTAATTTGGTAACAATTGTCCCTACACGTGTCATTTTATCTAGCATTGTTCCAAGCTTCTCTTGGAAAAGAATGCCATTAAGTTGATCCCGAAATTCTGAAAACTTCTTTCTTGTGTCAGTCTCATAGAAGAACTTTGCATCTTCATAACGTGCTCTGAGAACTGCCTCATTTCCTTTCCTCACAACATCCACATTAATGTCCCCGTTTGCTACCGCTATAAAATATGGCAACAAGCTTCCGTTCTTATCAGCAATTCCAAAGTATTTCTGATGCTTTTGCATAACCATTGTTAGTAGGTCTTTTGGAAGCATTAGGAAAGATTCATCAAATTTTCCCAGTATTGCGGTAGGTGCCTCAATCAGATTCACAACCTCATCCAATAAACCACTAGGCATAATAACTTGTCCGTTCACAGTTTCAGCTAAAGCAGCTGAATGCTCTGAGATCAATTTTTTACGCTCCTCAATATCTACGGAAATCCCAGCATTATTCATCACATCAACGTAAGATTCTGCATTTTCCACCTCAATAGTTGCAAAAGGCGTATTACGAAGACCATAAGATATATTTCCACTGCAAACTCCTGCATAAGTAAATGGAATGATAACATCACCGTGAAGAGCCATAATCCAACGAATTGGCCTACTGAACAATACCTGAGAATTCCAACGCATAGATTTTGGGAATGACAACTTAGCAATGGTACCAGGGAGATCTTCAGCAAAGACCTCCAAAGAAAATCTGGAGGGCTCCATGACACGAGCATATATATACTGAGTTTTCCCATCTACTTTTTCGTATATGGAGTCCAGCGAAGTATTGTATCTACGGCAAAAACCCTCAACAGCCTTTGTTGGATTCCCTTGGTTGTCAAATGCCTTAGAAACTGGGGGTCCTCGAACCTCAACCTCATTTTCTAGTTGCCTGGCACACAAGCCATCAATGTAGACCACCAACCTTCGAGGTGTACCAAATGCTTGCACTTCATGATGACTCAATCTTTGCTTTTCCAGCAATTGCACAATGAGATTTTTCAGCTGTTGTGTTGCATAAGCCACATCTTGTGATGGCAATTCTTCTGTACCAATTTCCAGGACAAACATCCTTGGCTCTACAGATGCCTTTTCTACTGCAGACTGAAGGGCCTCACTTGGAGAAGTTAGAGAACCAAGCTCAGCAGAAATGCCTAAAGGATGGCCAAGAGACTCCCTCGTCTTCATCCATAATTGTGCACATTGACGAGCTAAACTTCGCATTCGGCCAAAATAACGAGCACGTTCAGTTACACCAACAAAGCCTCTAGAATCCAGGATATTGAAAGCATGAGATGTCTTCAGAAGCTGATCATACGCAGGTATTGCTAGGCCTAAGTCAAGCAAATGACGAGCTTCCTTGTCGAAAAAATCAAAATGCTTCTGGATATGCTCGACAGTGGCATGCTCAAGATAATAAGCACTCATTTCCTTCTCGTTCTCTGAAAACAACTCTCCATATGTGATCCCATCAGCATACTTAATTTTCTTGAAATGATCAACTTCCTGAAGCAACATGAGGATTCTCTCAAGACCATAAGTGATTTCAACAGACACTGGAAACAATTGAACACCTCCAGCCTGTTGGAAGTAGGTAAATTGAGTGATCTCCATCCCATCCATCCATACTTCCCAACCCAATCCCCAAGCACCAAGAACCGGACTTTCCCAGTTGTCTTCCACAAAACGAATATCATGCTCATTGACATTAACACCTAACGCAGATAGGCTCCGGATGAAGAGGTCTTGAGAATTTCCTGGATCTGGCTTCAATATAACCTGAAATTGTGTGTGACGTTGAAGACGATTTGGATTTTCCCCATAGCGACTATCATCTGGTCGGATGCTAGGCTCTACATAAGCAACATTCCACGGCTCTGGACCAAGGACTCTCAAATATGTCAGTGGATTCATTGTCCCAGCTCCAACCTCAGTATTGCTGCATTGCATAACTGTACATCCAACAGAAGCCCAATATTCCTGAAGACGCTGAATAGCTTGTTGAAAAGTGGGAACAGAAGATGAACTAATAACTGAATTGGGTTCACCATTGTTGGAACAATTCTGCTGCAAAATAGAAGCGCCAGAACTTGACGACGCAAAAAAGCGACGATTAAGAGGTTTAAAGCAAAAATGGCGAGTTGGAGAGATAATAAAAGAGAAGTTCTGGGGTTTATAGTTATGGGGTTTTAGCACAGAGATGACTAGTGGAAGTGTGAGTATTCCCATTTTCTGTGCTTACTTTTTTGAGGAATTTGGAAATTGCAAGAAGAAAAGAATGAAGGGGAAAATGAGTTCAATTGCTTTGTTACTGGGTATTATTGTTTTCCTCCATTAGAATCAAATGTTGGTTCTTTTGTAAGCTATTAAACAGAAGAATGGTTTTGGGTTTTAGGGGAGAAGAAAAAGAAAGATAATGAGGGAGAGGTGCACCACGTTTGGG

*S.maritima*44113

GGCCGATCACTAGTTATGTCCACAAAAGAAAGATTACATTCTATCTAGGATTTTTTTTGTATTTTTCAAGTAGTGGTATATTTTCCTAAATATATATTCAATTTCATTTTTCGTCACTGTAACTAATCCTCTTCTACTCATTCGAATGACAACCTTAAACCCCTCATCAGCCACAACACACCTTGAGGAAGAAGAGTTCAAACAGTTAGAAGATTCTCTTCCAAAAGAAATATGGTTAGGTAATCCAATGCACCTTTATCAAGGTTTTTGGTGCCTAAATTTTGTTTTTAGGGGCATACTCTCATTTCAAACCCACTTTAGGGCTCGTGATACAGATTTCTTATTAGCAAGTCTACCAAAAACCGGAACAACATGGTTAAAGTCTCTCCTTTATACCGTTATCAATCGTGAAAATTTACCTAAAAATTGTTCTCAACACCCTCTTCATACAAAAAACCCACATGAACTCGTCCTCCAACTTGATTTGAATCAACCAGACGTAATAACTCAACTCTCCTCACCTCGATTGTTTGCAACCCATGTTCCATATACATCTCTACCAGACTCAATCAAGACCTCAAAATGTCGAATTGTTTACATCTGTCGAAATTCTTTAGATACTTTTGTGTCACTAT

*S.maritima*44113

GGCCGATCACTAGTTATGTCCACAAAAGAAAGATTACATTCTATCTAGGATTTTTTTTGTATTTTTCAAGTAGTGGTATATTTTCCTAAATATATATTCAATTTCATTTTTCGTCACTGTAACTAATCCTCTTCTACTCATTCGAATGACAACCTTAAACCCCTCATCAGCCACAACACACCTTGAGGAAGAAGAGTTCAAACAGTTAGAAGATTCTCTTCCAAAAGAAATATGGTTAGGTAATCCAATGCACCTTTATCAAGGTTTTTGGTGCCTAAGTTTTGTTTTTAGAGGCATACTCTCATTTCAAACCAACTTTGACGCTCATGATACAGATATCTTATTAGCTAGTTTGCCCAAAACCGGCACAACATGGTTAAAATCCCTCCTTTACACAATTGTCAACCGTGACCACTTATCGAAAAATTGTTCTGAACACCCTCTTAACACCAAAAACCCCCACGAGCTCGTTGTGAACCCGGACTTGATTCAACCACAAGAAATAGCTCAACTCCCCTCACCTAGATTGTTTGGAACCCATGTTCCATATTTATCTCTACCAGACTCAATCAAGAGCTCAAAATGTCGAATTGTTTATGTTTGTCGAAATCCTCTAGACACTTTCGTTTCATCATGGCATTATTTCCTTAAATTTGATACAAGTAAGGAGACTAAGCCAACTAAGGAAATGATGGAGGAGCATTTGAGTATGTATACCAAAGGGATATCCCCATTTGGTCCTCATGATGATCATGTGCTAGGGTATTGGAAGGAGAGCATGCAAAACCCAAAAAAGGTGTTCTTTGTGGAATATGAAGGGTTGAAGAAGGAACCAAGTGCTCATTTGAGAAGGCTAGCTAAATTTTTAGGCTATCCATTTTCAACAGAAGAAAAAAAGGGCAATGTTGTAGACGAGATTATACAACTTTGTAGCATTGAAAGTTTGAAACAAATGGAGGTGAACAAGAGTGGAAAATATTTTGGTTGGGCTGAGAACAAGTATTTGTTTAGGAAAGGAGAAGTTGGAGATTGGACCAATTACTTTACTCCTTCAATGGCTAAGCAATTTGATGAGATGCAAGAGAACCTTAAGAAAGCTGGCTTTTCTTCTCAATTCAATCGAAGTTAAAGGTCCAAAATATTCAAATTTAGTCAAAAGAAATGAAGGAATAGAGTGAGGCTTGTAAATTCTTAATTTGTATAACGAATAAATAAAACAACCTTTTCTTAATTAAGC

*S.maritima*1382797

GTCATGTGAGACCAAATCTCCGAGCAGTCTGAAAGCAGCATCAGCAACAGAAGAGATGTTGCTGAACTTAGGGAAACCATTTACAAGGCAGCTCCACACTGTTTGCCAACCTCGGGAAATCTTATTTGAGATAGAAGATGACTTGAGTGATCCATTCTCCAAAGACAATAGGGGCAGAAGCAGCAAAAAGAGGTGATGCAGACTCCGCAGTGTCCACAGCAAGCCATCATAAGAGTGCTCCACATTAGCATCAGTCAAGATTCTGTCCAAACTGGAGCATATGCCATCAAACCAGTATCCAAAGAGATCCTTT

*S.maritima*44979

CAGAAGCCCAAGCACGATTCTACTTCAGAATATAAGTTCCGGTTTTCCCATTATCACTAACACTTCTAAAAGATAAGAATGGAGATTGCAGAAATAACAAACTTAACATGGAATTGCATAAATGGCCAAAATTTTCAAGTCTCATACTAATACACATGTATGACATTAAAAGTCAGGCTGGTAATCTACGAAGGCACTGTGGTTGACAAAATCTTGAAGGGGTTGATCACAAGTTCGTAAGTCAGATGCTTTTTTATTTTGACGGCCCCTTCTTTGAAGATCCTTTGTATGGTGATTTCTTGAAAGATTTTTTCTTCGGGTTCAATTTGCTAGAGCTTGAAGATCCCTTATCTCGCTTGTTCTTTGACTGTTGCTTCTCCTTCTGCAAATTGATATTTTTAACAGCCTTGTTGACATTTAGTGAATCATGGGAATTTCTGGAGATGAGCTTGTTCAGGATCTTTTCAGTTTGTTCCCTTTCTTGTGAGCCCATACCTTTTCCTTGCGCAACAGGCAGAAACTTTCGATGTTTCCCTTTGTTTTTCAGTGGCTTTTCTCCAGGCAATTTTTTGTCAAATTTCCCAATACTGGCGGTTGACGTTGCTGCTGCACCAGCTACCTCTTCTAGCTCAGTTTTGCTGAGTTTCTTCGGAGCAGCATGGCTCCCACTTATGGGCAAAGATGTAGCAGCTAGTTGGACATGGCTTGGCAAGGCACCACTCTTTGATGCATCCTTAAGATTCTTCATACGATTCTTCTCTTGTTTCTCTACTCTTTGCTTCTTGTCAGTTTTTCGCTTTGCAAAAGGATCCACTCCAGGCTCATCAGTTGGCTTGGCAACAATAATGGGCACATCATCTTCATCATTAGCACGATCATAACCGTAGGTGCGCTTCCAAGTACCGGTCTTTTCATCATATGCAATTTTGTCTTTCTTGCGATTCTTAATGCCCTTCTTCTCAGCAAACAACTCCCATTTTGTTTTTGGCTTTGGTCTTGGGAGATGCTTCTGCCGCGGAAGTCTAGTGGTTGGAGGGGGCAAAGTGACAATGGGTCCATCCACATCTTCAGTTGCAGGCAAATTGAAAAGTGAATCTGCAAGTGCTTGTACTAATTCTGTTCCCTTTTCCAAACATTCCTTCACCAATTCTTCCCTTGAAGATGGAATAGAAGAGAAATGGTGATTAGGGTTGTAAGCCAAAAGATTTCCCAAATCAATTTGAAAACCTTTATCTGTTTCAATCGCACCCATGTTTTTCGCAAACTTAAGAGCTCAAAATTTTGGGGATTTTTTTTTTCTGCTTCACTTTACTTGATGAGAACAGAATACTTT

*S.maritima*42692

TTTTTGCGGAGAAACTACTAGTATCTCAAATTGATTAACATGCTAAGGAGAGAAAACAATCACCCTTCAGAATAAGCTAATAAGAGAAACTTATTCAAAATGTATTTTCACTTGTATGACAAATTGATACAAAAGCCTCTACTCAACTGGAATTTCTAAGTGTCACTAAAATGAAGCAACAATGAAGTGTACAAGGCATTTTAAAAATAATGAACTCAGTCACAGAGCACCAAAATTTGTAGAGTCTAAAACCTAAAACTAACTGAAACAACAAGCTGTAGAAGAACATGAGATACTCTTGCAATGTCACAGGCAAGTTTGCAAGAACATCGTAAAAAATATACATACCGGAAGAGGGTAGTGGGTATAGCAAATTCGCAATCTCAAATTCTTCAAGGCAATGGTGGAGCTTCATTAGAGTCATTGATGATACATGGAAGCAAAATTTAGACATAGCTGATGATCTCTAGCATCTCCCCAGCCACCCCAACCTTTGAACCCGGCTTTATAACCTGCTTGATGTTCGTAAAAAAACACACTCCACTCTGGGCTAATGCTCACGACTCTGTCATCATTCCGAACTTCAATATTCACAGAACCATTGTCTATACAAGCATCCGAATCCCCTTGACCTTGATGCAATCCACATTTCCCAAAGTGAATAGCACTACTTGTAGGTCCTCGTAATGTATACACAGGACTCCCAAACGAAGGGTAGACCTGAGACCACATAGTTATATCCCAATTGTATTCATCAAAGAAGCAAAATTCTTCAGCTTTCTTGTGGATTTTCCGCCACACAGTTCGATTAAAAGAGTACCCAATATTCCCCATTCTCTCAGCAATCAATGAAGAAATACCTTCTCCTCTAGTTTTCACATCCCAGGGAGCTAAATTCACTGCATAACAGCTCGGACACTTCTTGGGTAACAGCTCTATAAGAAGTTGGAGATTCCTATAAGCATTGGGAAATATAAAGTGGTCTTCTTCAATGAAAAGTATATGACCTAAGTGATGCTTAGTCTCCTTCAATCCATCCCACACCGTGTTCATCATCCACCACCAATGATGCTTCAACGATACAATCTTCGGGGACCGATGATTCCCATACTGATCAGGGTTCCCCTCACACTTCTTTTCATTTGCATCTTCCTTTTCCTTACAATCCTGAGGAGAAACACCTGGGAAGCTATCGGGAAACAAATGAGGCGAAAACGGAGCAAAAATCTGCTTAACCTGACAAAACCTAATCCCCTCCACAATCCTATTCATCTCATCAAAATAACCATCATGACTAACTATAAGTAGAGTCTCATTAATTCCTTCTACATGAGACAAGCTCTCAACAACCACTTTAAGATACTGTGGCCTATTATGCACATACAACACAATCACAACATGATCCTTAGGCAGACTTGGAAACAGATCCAAGTTCCGCGGTGGCAAATCGTTCCGTTCCTCCAACAATTTCGACAGTTCATTCTGTTCCGGCAAAGCAATGCTTTCCCGTCTACGCAACGAAACGGAAACTATATGATCATGATCATCATGTATGTCCTCATTAGACCCAGTTGCATAATTAGAATCAGAATTAGTTCTATTCAGTGAAACAGACAACAAAAGCAGTGAAATCAAAACTACTGAAATCAAGATTAATCTCCCAAATACACCAACTTTATAAGGTGGTTTTTTCCCAGAAGCCATAGCAGTTGAAACTGACATTGCTCAATACAACATAACTTCCTCCCAAAAAAAAACAACACAGACCCAATATCACAAAAGATGAAATTAATTAATAGGGCAAAAAACTAAAATCACCCATCTAAAAACAAAAGTAATCAAACATAAAGCAGCAAATAAACTTTTAAACTGTTAACTTACTAAAGGTTAAATTGAATGAAATCACCCAAATTCCAAAACCCTTGTTTGGATTCCTTTGAAGTCGAGTTCAATCAGCAATTTGAAGTGAGAAGAACCCCAGAAACCCCAAAAGCTTGGATTTTGATCAGGGCTTTGGTTGAATTCAAGTAAGTGAGTAATTAAAGATGAGATTTTTGGAAGAGAAAAGAAGTGATTTTGACAATAATTTCTGGAGAAAAAACAAAGGGGAAGAGATCTAAGAAGGGTAATTGAAGGTGGGATTTGTAGAATTTGGGAGGAAGTTGTTAGAAGAGTAAGAAGATTAATGGACTAAGAAGAAGAAGAAAGAGGAAGAGACGATAGACGACTTTGAAGTAAAGACTTTTCTTTGGTTTGGTCAGTGTTTCAGAAAAGACTTTTCCTGGTTTTTTTTTAGGTGTTCCATCTAGTTTTCAGGTGAGAAAG

*S.maritima*34359

CCCTGAGCTGTAACTCCTCTATATGCAAGACTTATTTGCACCCAAAAGCAGAGCACCAAGAAAAGAATCAAGAGAGAGAAACAAAGAAAGAAGAAAAGCATCACATTTCAAATTTTTGTGGGGTTTTGAAGCTTGAATCTTTCTTTCTATCTAATATATATTTTTATCAAGGTTTCATTGAAATCAATTAAAGGGTATATTTCTGCTTTGAAGATGGCAAGCTTTGGAGGAACAACTCAGAAATGCAAAGCTTGTGAAAAGACTGTATATTTGGTTGATGAACTTACAGCTGATGGTAAAGTTTTTCATAAAGCTTGCTTCAGATGTCATCACTGTAAAAGCACTCTCAAGCTTCATAATTATAGCTCCTTCGAAGGGGTGTTATATTGCAAGCCTCACTTTGATCAACTTTTCAAGATGACTGGAAGTTTAGACAAGAGTTTTGAAGCTGCTCCTAGAGTTGTAAGAGATAGATCTTCTGAGCAGGGTCAAACTAGCATCAAAGTCTCTAGCATGTTTGGTGGAACACAGGAGAAGTGTGTCTCTTGCAAGAAAACTGTTTACCCGATTGAAAAGGTGGCAGTTGATGGCGATTCATATCACAAGGGGTGTTTTAGGTGCTGCCACGGAGGATGTGTTATAAGTCCGTCAAATTATGTAGCGCACGAGCATCGCCTGTACTGTCGTCACCACCATTCCCAGCTGTTCAAACAGAAGGGTAATTTCAGTCAATTGGCAACTCAACATCATGCTAATGGAGGTTCTAGTGGAACTACCAATGGAGTTGCAAATGGAGGCTCTAATGGGACTACAAATGAAGTTGCAAATGCGGTTGCAAATGGAGCTTCTAATGGGGTTTCGAATGGAGATGCTAATGGGGTTACAAATGGTGATACTAATGGAGTAGCCGATGGGGTGACTGAGAACACACTGGCCTAACCTTGGCTAGTTAAGTAGAAAATGCGCTTGTCTTCATAGTGGAGTCTTTGTTCAAAAGATGATACCTTTGTAATTCCTGAGTCATTATTGTAATTCCTGAGTCATTATAACTTGCTGAGTGTATTGCCGAGAGAATATTCATGCGCCATTTTTATACATACTCAAAAAAAACAACTTATACTAGTG

*S.maritima*39814

TGCCAAAATCAAAATCAAATATCGATATTATAAATGAGATTTAACTTGAGTTGCTTTATGTCATTGACGAATTGTAGAACATACATGGTATAGGACCAGAGAAAATATAAAATTAGTTATGTTAAAGCTTGCTCCAAAACTGTTGAAAGGTGTGAGAGACTGGGAGTATCATAGCAAAGTATAAATGTGCTTATGAGATACTTTGGTAATAAGATACAAAAACGGAATGCGGAAATTCAGCATTCCCTTTGAAGGAACATTCCCTTTGAAGGAAGATCAAGAAAATTTTGGAACCTATATATTAACATAATTAGATTTAATCTACAACAGAATGTTTAAGCAGGCTTAAGATGCAATACCACTAGGCCTATGAGCTTTTCAAGTCCGACAAGGCATCCTTGGTTGTTGCGGAGATTACAGCATCAGATGCATTAGGCAAGTAATAGTATTTTCCTTGAGCCACTCTGGCTATCTCTTTAGCAAAGCCCGTAGATACAAATTTGTTTTCAGTGTCAATGACGAGCAGTGACATTCCTGCTTTGTATATTTTCCCAGCTACTTCAAGAATCTCATCCTTCAACTCTTTCGAAGAAGGCCGAGGGGTATTTGCAGAGGCTTCTTCGGGGTCTGTAGATCTTTTCAGTGATATGTTTGCTCTGCCGTCAGTTATAGCAACAATCATTATGCGTCCAACATCTCCACTTTTTTCTGCATTTAGTCCAACCCTCACAGCCGTTGTAAGACCGTGAGCTAGAGGAGAACCTCCACCACATGGAAGTCTCTCAAGACGATTTCTTGCCATTGAAATTGACCTGGAAGGAGGCAGAAGAACTTCTGCAGCATCCCCACGAAATGGAATGATGGAAACCTGATCTCTGCTTGTATAGCTCTCAGCTAACAACTTAAGTGCAGCACCCTTTGCATTCTGCATACGGTTTAACGCCATGCTACCACTAGCATCAACCACAAATATAACCAAAGCTCCAGCTTTTCTAGCCATTCTTTTTGCTCTCATGTCTGTCTTTTCCACAAAAACTTTCCTCCCCGTGTTCATATCCTTAGCCTTCCTCAATTTTTGATATGGAGCAGCAGCTCTAAGGGTTGCATCCACAGCTAATCTCTTGACAGGACCCTTTGGAAGCATTGGCTTAATGTAGCGTCCTCGATCCTCTGAAAATATGACATTCTTTGCCCTCCCAGCCTTTCCTTTGCGTCTCTGAGCTTGTTGAGCAAAGAAAAGAAGTTTCTCATCCACCAACCCTCCTTCTGCATCAAAGATAAACTCTTCTGGTAATTGGTCCTGCTGTTGCTCGTTCTCTTCATCATTCTCATCCTCTTGTTCTTCCTCCTCATTCTCATTCTGCTCCTCCGACGAATCCTGATTCTGTTGAGGGGGTGGGGGAGGAGGTGGCTGTTTATTCTGATCTTCTGGGGGGGTTTCCTGGATGATTGAACGAGGAAGAATTACAAGTTCAACAGCTTTCTTCAGGTCATCCACACTGACTTTTTCACGCCCTTCTAAAGCAGTGAGACACTTGGCAACACGCATAGCATACAACTCAGCTCTGTGGCCCTGGCAACCACCTCGCATTGCTTCCATAACCAAATACTTTATTTGCTCCTTGCTAATTGTAACATCTTTCAGGTACTCCCGTGCTAAAATGATCTGTGTTTTAGCAACTTCTGTTTCCTCCTCGACCATTTTAAATACATCATTACATTGTTCTTGAAATTGTGTTGCAACTCCAACCGCTGCAACACGGTCATCAAAATTCATTGGAAGATCTGCACTTAAATTTATTGCTATACGATCCAACAAATGCTCGCGAACAGCACCCTCTTCTGGGTTATAGGTGGCAATTAACAGGGGCTTGCAAGGGTGACGGAAGCTGATTCCTTCTCTTTCAACAATATTCACACCCTCCGTTAAAACATTCAGGAGAAGGTTACTGATCCCTTCATCCAGAAGGTTTATCTCATCAACGTACAGGACACCTCTATGAGCTTCAGCCAGAAGTCCAGGCTGAAAAACAGTAGTTCCCGTCTTCACAGATTCCTCAACATCAACAGACCCAATAAGCCTATCCTCTGTAACACCAAGAGGAATCTGTACAAAAGGAGATTTTACAACATTGGTCTTGATATTGCCAGATGAATCATATTTGACATGATCAGCCAAACCATCTTCCCACTCTTCTGGACAAGATGGATCAGCATTTGAAATGGAGCCCACAACTACATCAATAGGTGGAAGAATGGCATGTAGACCACGTGCCATAATCGTTTTAGCTGTCCCTCGCTTTCCTGAGATACCAATCCCTCCGATTTCACAGTCAATAGCTCCAAGCAGCAGCGCTGTTTTAATGGCATCCTGTCCAACAACAGCAGCAAGGGGGAAGAATTGGCGACCATAATTAGCACCTTTAGGGCTTTCAGATTCAGAAATTACGGTGCCATTGTCAGAATTTAGGGTTGTGGTGGCGCAAATTCTGAGCGACGGTGATGAACGGCGGAAGTTACGGCGGCGAATGAAGTTAGTAGAATTGAAGGAGAGAGAAAATGGAAGTTTGGAAGAAGAGTTTGAATTGTTGAAGAAAGTGAAATATGGTGATGAAGAAGCAAGCGCCATTATTGGAATGGGGGAAGAAGAAGAGGAGGAAGAAGATAGGGGGGAAGTTAAGTGTTCACTTATCTGCTTATGTGGAGTTTTGTTTTGT

*S.maritima*31847

CTTGAGTATATCTACAGTTTGCCCCAATTGCTATTAAAAAAAACAATGTTTAGAAAAAGGAGACTGTGGTGGGATGATGGTGGTGATTACATTACATTACATTTGTGATGAAATTTGAGCAAGTTTTAGCCTTTTGAGGAAAAAATTAAGTGGGGTTTTCATATTTTTTTTTTCTTTGGAGCTTTGAAGAAGGCAAATTACAATTGCAATTACAAAAAGTTGTTTTCTTTTGGGTTTTTTTTTAGTGTATTGGCTTAGTCTTTGTAGTAAAGTCAAGATTTTTTTCAGATTTCTGATACATATAATTTGTTTGGGGGTATTATTATTATTATTGAAGAAGGAGATATGCCTAAAAGAAACTTTCCCTGTAATAGTACTTGACAATTATTCTTCTTCATCACCAATTTCTAAAGTAATTCACAAGAAATAACCAGGATCAAACAAGGAGATTTAAGTGCTGCTTAATCAACTGTTCATTTTCGCCTTAGTTTTCAGTCGTTGGGACCACCCATTGCACTAAGTTGTCTAGAATCTTCTGTATCCGAGAATTCAAGTTTGAAAATGAGAGTTCCTGCTCAACAATATCAGGGCGCAAAGTTGTTGGATTTTTCATTCCAAGATCAAGATTCATGTTCAACTCAATCAACAGAACAATCTTATACCAGCAATGCTAGCTTTGGGGAAAATGATGTTAATACTCAAAGCTTGAGTTTTGCGCAGTCAGGTTACACTCATGGGAAACATGAGGGTGGCGACAGTAAATTCACCTTGCCAATGGGAACTCAAAACTACATCTTACCTCCTCAACAAACTGATTTCAACCAAGCGTTTTTTCAGCCTCAATTTGCTGTTACATTTCCTGATCAGTCATTCAATGGTGCAATGTCAACATATGGGCCTCAAGCTGTGATGATGGGAATTGTGCCTGCTCGTGTTCCTCTCCCTGACTATTCTGCAAGTGAGCCCATTTATGTCAATGCGAAGCAGTATCATGGTATCTTAAGAAGGAGACAACATCGTGCTAAGCTCGAAGCTCAAAATAAACTTGCCAAAGATAGAAAGCCTTATCTTCATGAATCCCGGCATCAACATGCACTAAAAAGGGCTCGGGGATCTGGTGGGCGGTTCCTTAACAAAAAACAGCTCGAAGAATCCAAGTCTGCTGCCCCAGATACTGAAGGGCATCTGATTTCAGGCTCAATTTCGCTCCATTTGGCTGGAAATACATCAGCGTCTAACATTCATCGACAAGAAAATCATGGAGAAGGCACCTCCACAACTTCTTGTTCAGATGTAACTAGTACCTCCACCAGTGATGATATCTACCATCCACTGGAATTCAGGTTCTCGGGTTACTCATCTTGCTTTCGGGAAACCAAGCAAGGTGGTAATATCAGAGGGAACTATTTGAAGTTCTAAATAAACCAGCATCGAGGTCAATACTTCTTCTGCTGGTCATTCCTTGAAAGGGAAGTCATCCTTGGCTAGTTGGCTCAGTTACATTCTGCTTTGTGTTCAGTTCTTTAGATCGACAAAACTTTAAACTTAGAGATGCAGTTCTTCTGCATTCGATTTATGGAATATGCGATGAACATGTATCATTTTTAATATTGGAGAACGAGGATCAATATTGATATTGTGATTACAAACTTTATTAACATGATTGTGG

*S.maritima*25945

CTACAAATTGTACAACAAAGATTCCAAAAAGTGGGATAAACGTGTCATACCTGTTTTCTATGAAGTTGATCCATCTCATGTTCGAAAGCAAAACGGGGTGTTTGGTAATGGGTTTCAGGAGCTTCTGAAACAGGCAGACAGGTTTAGTAAAGATGAGATTGCGATGTGGAAAGGTGCTTTTGAAGCTGTTGGTAATATTTCTGGTTATACATGCCCCATTGAAAGGGTGGTTGGCAATGAAGCTGAAGTTGTACAACTTTTAGTCCAGCAGGTCTGGAGTGAGGTGAAAAACACCCCAGAATATGTGGTGAAACTTGATGTTGGGTTGGATTATCGAGTTGATCATGTGGTGAATTTGTTAGATGTTCAGCAGAAGGACAGTGTTCGATTTCTTAATATCCATGGAACTCCAGGAATCGGCAAGACAACCTTGGCTAAGGCAGTTTACAATAAGCTTGCGGCAGACTTCAGCAAGCGTTGTTTCATTTCTAAAGTCAGGGAAAAATTGATGCAAAAACCCCAAGAAGAGGGCATTTTAAATATTCAGAATGAAATTATAAATGGTCTTTCTTTGGGAAATGCAAAGTCTGTTGATGATGGCAAAACAACAATAAAGAGAATAATTTGCCAAAATCGAGTCTTGTTGGTTTTAGATGATGTCTTTGACGCAAACCATTTTGATGATATTGGAATTCGAAGAGAATGGTTTGATGGTGGGAGCAGGATAATCATCACGAGTAGAAATGAAAATGCTTTACTTGAGTTGGCCCATAGTAATGAGCTATATGAAGCAGAAAAGCTAAATTCATCCGAATCACTAGAACTATTTAGCATA

*S.maritima*43442

TCTTAATTACTGTAAATAAACAAGTACTTATAGTTTAGTAACACTAATTATCTAGAAGAGTGACTAAAAAAAAGGAAATAAATATCCAAAGTCTCTTTTATTTTCTCTTTCCTCCTCCTTCTCCGATGTTGTTGGTGGTGCTGTCTCTCTTAAGGATGCAAGATTTTCACTTCCCATCATATTTAACCTTCTTCTGAATTTCCCCTCTTTCCTTCTTTACTCTGTTTTTCTGCTCTCACTTTTACTGTTGTACTTTGAAATTGATATTTTTTTATGAATTGCTTCTCCCTTTGTTTTCTTAATCAAAGAACTAGTAGATCTTGATTTTAGTTATAGAGATTGAACTGAATTGGGTAAAGATGCCTAGTAAAGATGGAAGTTTTGAGGATCGATTCGAGACCAATGGAGGGTCCAAGCCTCAGAACATGTTGTTTAGACAGCCTTGGTGGCGCGGCTTGAGTGATAACGGTGTTTCCCCTAACTCAGGTTCGAATGATTCGTCTGGTCGAACATCTAGTGGGGAACCAGTTAATGGTCCGGCCTTAACCGGCGCCTTTTCAATGCACATTAATGGGATGCAGGACACTGGAGCTAGTGGTTCTGGTGAGATCCAAACTGCATTGGCTCATCAATTTGATAGGGATAATGCACAAGCTCAGCAACCTCTTAAACAAGGGCCATCATCAATGCCACAAAATCTTGGTGATCAGTCAAACGCCCAAATGGAACTTGCTGGCCACTCAATTGTTTTGACTCCATACCCACTCCAAGATCCAACATATGGAGGAATCATGGCTTATGCACAAGCACAACAGATGAATCCTCAGCTGTATGGATTGCACCAGAGTAGAATGCCTCTTCCCCTTGCTATGGAGGAAGAGCCTGTTTATGTAAACGCCAAGCAATATCATGGGATTTTAAGGCGTAGGCAGTCACGTGCTAAGGCAGAGTTGGAAAAGAAAGTTATTAAATCTAGGAAGCCTTATCTTCATGAATCTCGTCACTTGCATGCTTTGAGGAGGGCAAGAGGTGCTGGTGGTCGTTTTCTTAATACAAAAAAGCTTGATGGAAACAATAACGATACTTCTTCAAATACAAATTCTGCTACCAACCTTCAAATGTATTCCGGTGGTTCAGCACGTTCTGAACACTTCACAACACCCTATAATGCTAATCAGCAAGAAGCAAGAGGGTTAATGATTTCAGACATGCACAAAGTGGAGTCACATCCCAGTGGCAACACTAACGTTCATGGGCTTTCGTCAGCATACCATTCATTTTCTGGTCCTAATGAAAAAAGAGACAACTACGGTCAAGGTGGACTGAAAATGAATGGTGTTGTTTCTGTCAAGTAGGACCCACTTTATGCTTGAAGGGACGGCTTCCACCTTATTGTCTGGTTGTAAGCAGTATTTTGGTGGCCAGTCATTTAAGGTGGACCATCCTTCAAGTATCTGGAGTCCCAGGCAACTCATTCTTGGCTCTGGTCTACGAGGTTACAGTGGTCGGGATGGAGAAGGAAAGACATGGTAGGAGTCTGCTGTCATCCTACCTGCTACTGTTTTTCCTTTCAAAATCAACCTATGCTTGTGTATAAATGTACTCAACTTGTAAAATTTCATGGATTGCTATTTATTTTTGTGTGTGGATCTCTCTGGTTTTTGACTTTAAAGAACTGTCGAACAACATTGGATAGTGGTGTGCCTCGTTTAGGTAGTGTAGATGTTGTATCTCTCGGTGTAATATAGCTTAGTGTAGTTTTGTAGGCTTTCAGCCTGCGAACGCCAAAAATAACGTAGTTTTGTAGGCTGTCAGCCTGCGAACACCGAAAATATCGACAACATGGATAAGCAAGATGAGTTT

*S.maritima*220459

TTTTTTTTTTATCCATTGCATACAAACTACACAATGGGATTCCAAATTAAGGCCATGGATAGTGAGCAAGAAGAACTTCAATTCCTAGGGTTTTATGGAATCTTCAAAGAATCACTTAAAATTATCTTAACATGGCCTAAAATCTTCCTTCAAATCACCCTTGCCTTTATTCTCCCTCATTCTTGCCTCCACTTAGCCAAAAATTTGGTTTCCAAGGTTATCATTAGTAACATCTTTCATAATTCCTCCACCTTACCCAACAACCAAGTTGGATCTCCAACATATGAACATGTTTCCAACTCAATCTATTCGGAATGGGCTACATATTGGATTATCAACGCAATCTACTTCATATTTTCTATTGTACTATCTCTGTTCGCCGTCTCCGCTACCATTTATGCAGTTGCTTGTATTTATACTGGTAAGGATGTTACGTTCAGAAAGGTCGTGAGTGTTGTTCCTAAGGTGTGGAAACGGCTAATGATCACGTTTTTGTGTAACTTCTGTTTAGTCATCGCATACATATTTGCTATTCTACTGGTTGTGGTTATGTACACATTTATATACTTTATGTTCTTTTATGATCCACAAAATAGTAGTGTGGCTAACGTAGCAAGGACCATAAGCATTACAACATCTATAGTAAGCATATTATTGATGATTGGGTTTTTCTACTTGGCCATGATATGGCAATTGGCTAATGTAGTCTCAGTCATGGAGCTTAATAATTGTGGGGTAAAAGCCTTGAAAAAGAGCAAGGAGTTAATCAAGGGTAAAATGGGAACTTCCTTTGCTATCTTTTTGGTCATGAACCTTGGTGCAACACCACTATATCTAATGATTCAACAATTAGCAACATATGGAGTAGTACAAAGAATTTGTTATGGAATTCTAAGTATAGGGTTGTGGACTTTTGTTTCCTTATTTGCCCTTGTTGTTCAAACAATTCTATACTTGGTTTGCAAATCATATCACCATGAAAGTATTGACAAGTCCTCTTTGGCTGGTCAACTTGATGTGTATCTTGGTGAATATGTTCCATTGAAGAGTGAAGATGCTATACAACTTGAGAAACTCCATGTATAGCTTGAGGGCAAGCCGACAAGGGTTATTAGTAATTTTACTTGTATAATTTGTATGTGAAATAATTTCATTTCGAGCTTATAATTTGGTTTATGAGATGTATGTATATGTTCGTATCTATATGTATCGTTTTTATTCATTTTCAAATAAAAAGACTAAAAG

*S.maritima*44671

GCGCAATTCCCACTTCACCAACGACAATTCCTCCTTCCTTCAATTCTCAATTCCTCCTTTCTCTCTCCTCCTTCCTCTTTCTCTTTCCTTCCTTCTTTCGTATTTAGATCGATTGGTGTCGGTGATCTCTCTATCTCTCCTCCCAAAGATCTCTCTCCTTCTTCTCTCTTCCTCCTTCTTCCTCACCCCCCTAATTCGCATCCCCAAATCCAGGAAGAAGGAGGAACATACATACAATCTACCAAACAAAAAAAAAAAAAACCCAAAAAAATTCATCATTATCACCATCGTCATCAATGGCAGGTGCATCGAGGAGATTGAGAGAGTTACAATCTCAAACAGGCAACAAAACATGCGTAGATTGTTCCCAAAAGAACCCACAATGGGCGTCCGTTTCCTACGGTGTTTTCATGTGTTTAGAATGTTCTGGAAAGCACAGAGGCCTCGGTGTTCATATCAGTTTCGTTAGATCTGTAACCATGGATTCTTGGTCTGAGATCCAGCTTAAAAAGATGGAGTCTGGTGGCAACGACAAGCTTAACGCCTTCTTCGCTCAGTACGGTATCCCTAAGGAGACCGACATCGTCGCTAAATACAATTCTAACGCCGCCTCGGTGTACCGGGATAAGATTTCCGCGATTGCCGAGGGGCGCTCTTGGCGGGACCCGCCTGTTGTTAAGGAATCCGTCAATGGAGGGAGTGGGGGTGGGAATCGAAAGCCGCCATTGATGGCGGGTGGTGGTAGTGGGAGGGGTAATGGTAGTGTAAAGTCGGCCGCGAGTGTGGGGTGGGATAATTGGGATAATGATGATTCGTATCGGTCGTCGGCAAATAACAATGATATGAGGAGGAATCAATCAGCTGGGGATTTTAGGCATAATGGGTATGGGAATGGGAGTGGACCGCACAAGTCGAAATCGTCTGATGATATGTATACAAGATCACAATTGGAGGCGTCTGCGGCGAATAAGGATAGTTTTTTTGCTAGGAAGCAGGCGGAGAACGAGTCCAGGCCCGAGGGGTTACCGCCTTCTCAAGGAGGGAAGTATGTTGGGTTTGGATCTAGTCCTAATCCTAATTCTATGTCTAGTAATAATGCTAATGCTCAAGGTGATGTTTTTTCAGTTGTTTCTCAGGGAATTGGGAAGTTGTCTTTGGTTGCTCAATCAGCTGCTAGTGTTGTCCAAGCAGGAACAAAGGAAATTTCTTCAAAGGTTCGTGAAGGTGGCTATGATCACAAGGTCAATGAAACAGTTAATGTCGTTACTGCAAAAACATCAGAGATCGGTCAGAGGACTTGGGGCATCATGAGAGGTGTTATGGCTATTGCGTCTCAGAAGGTTGAGGAATATACGAAGGAGACCGGAGTGAACTGGAACACTGACAACCCACATCAGACTGAAAATGAAAAGAATGGATACTATCAGAACTTTAGGCATGAATCACAGGGATGGAACAATGCTTCTGGTGCTGGGCAATCTTCTGGGAGGGAAAACAATAGCTCAGCATCTTGGGATGATTGGGACCAAAAGGATGACTGGAAGGAAGGTTCCAGAAAGGAAGGATCGTCCCATAGTAATAATGATGGTTGGGCTGGTTGGGATGATCCAAAAGATGATGGATTTGATGATTTCTACCAAAGCTCAACTAAAAAGAGTTCAGCTATCAATGGGAAATCCGATGCCAAATGGGGTGATGGTGGATTCCTATGAGGTTAGTCTAATGTGGGATTGTGCTATTTCTTCCGATATTTTGTTGCAAAGCTTTTATGTTTGTTTCTAGTCTATATGTGTAAAATTTGTCCACATGGGAGGTATTGTCCCTCGAAAAGAGAACTTATTGGTGACTCTAAGGCGGGTCAATATGGCTATTCTTCAAACTGCTTTGTTCGTTCAACGACTCTTGTCTATGATAAATTTATCTGCCCCTTTTATCCCTTTTGATTCACTTTAACTTGTATCATAGGAATCCTGGTTTATTGTTATTGGATTCAGGATTATCATTTGCTAAATTCTACTGCAACTTTAGATGGAGTAACTTATAGTCGACTTCTGCTTACTGAATTGAATCCCAGGGTTCACTCACAACAGTCGCAACGTTGCAGTTTGATGGGATTTGGAGACTTGGAGTAACAGTGCTTTTGAAAGCCAAATGTAGAATGCTATTTCAACCTGTTTGCTTGTTCACATGCAGTTTTTCTTGCACCTTGTTCATTGATAAACGTTTTATGCAATATGAATGGTGAAGTGCATACATACGCACTCAGACTTTACACATTGTTTGAATAGAAAGAAATGGAGAGAAAGTGAGGGAAGGGAGAGGGAGGGGATAAGCCCCCTTGTTTGGATAACGAATTGAGGGAAGGAACGACCCATATTCTCTCCTTTAATAAACATTGTCCCTACTAAATTGGAGAGATTTGGAGGGAAAATGGAGCTCCTCCTCCCCTCCCTTTTTTTCGCCTCTTCTCTTCCTTTCTCACCTAGCTTATCGTCCAAACAAAGGGTTATTGATGGAAGAGCAGTTAGTGGAGCTTTCTTGGTTTCATGTTATCACTTTACTTTGCTCTAACTTCTGGAGTTGTATCTCTGTTTGGGTTACAAACTTACCATTAATGTTGAGTTAATTAGTTTGTTTTGTTTATTTATGATGCTTTCGGACTTCCGGAGGGCCTATGTATGAGTTTATTTGCATTCTGTCTCTGAATCGAATTGGAAAACAGTCAAACATTGAAATGGAATC

*S.maritima*762928

CGCTAACCCGCAATCACGGTCCGTCGCAGCAGTGTTTCGCCCGGAGTACCTACCCATATAAATAAAAAAAAAGCATTTACAATGGAGTTCAAACAGTTTAAAGAATTTTCGACTGACATGATCGATTACGTGGGAAATTATTTAGAAAACATTCGAGATCGGAAAGTATTGTCATCGGTAAAACCTGGTTACTTAAGACCACTTTTACCGACCGAGGCGCCTAATGATCCCGAAAACTGGAAAGATGTGATGTCTGACGTCGAAAAGCTGATTATGCCTGGGGTTACTCATTGGCATTCTCCAAGATTTCACGCTTATTTCCCTACGGCAAACAGCTACCCGGCCATTGTAGCTGACATACTGAGCGATTCAATTGCTTGCATAGGATTTTCATGGATATCAAGCCCCGCGTGTACTGAATTGGAAGTGGTCATGATGGATTGGTTGGCTAAAATGATAGGCTTACCAGAAGTCTTTTTAGCCTCTGCTGGTCAGGGAGGAGGCGGAGTAATTCAGGGCACTGCTAGCGAAGCCACTTTGGTGGCGTTGCTGGGCGCTAAGGCGCGGATGCTGCAGATAATGAAACAGGCGGACCCAACAGTCGTGGACGCGGACATCGTTCCCAAGCTGGTCGCGTACTCGTCCGCGCTGTCCCACTCGTCCGTGGAGCGCGCTGGTCTGCTGGGCGGCGTGTTGCTCCGCGCCCTGGACACGGATGGCGAGCACAAACTCCGCGGCGACACCCTGCGAGATGCCATCGCCAAGGACCGCGCGGACGGCCTGATCCCGTTCTTTGTGGTGGCGACGCTGGGCACCACCAGCTGCTGTTCGTTCGACCGCCTGGACGAGATCGCCGAGGTGGGCAGCGCCGAGAGCATTTGGGTGCACGTGGACGCTGCGTACGCTGGATCTTCATTTGTTTGCCCGGAATATCGTCATTATATGAAAGGAGTAGAATCAGTCGATTCATTCAATTTCAATCCTCATAAGTGGCTTCAAGTTACTTTTGATTGTTCAGCAATGTGGCTAAGAAATCCAAATTACGTGATAAATGCTTTCAACGTAGATCCACTCTATTTGAAACATGAACTTGAAGGACAAATGCCGGATTACAGACATTGGCAAATA

*S.maritima*31904

AATTAAATTATAAATAAAATTTGCACCTTTATTTCTTATCTTTTACTTGTGGCTTTGATGTTAAGCTGTAAAAAATTATTTGATTAATAAGGAAATAAAAAGGACCCATTTTTATTTGTGGGTCATTATTTTCATTTCATTTCTTGCAATCCCCATTTACAAGTTTCAAACTTTATCTCAATTTTCTCTCTCCAAAAGTTTTTTTTTTTGGTGGGTGTAATTGTGCAGAAATTATATTTTCTAGATTACTTCCTTTTGCAGAAGATTGGTGCTTAGCCTCATTGTAATTTATCTGCAATGGGGAGATCAACTGGGAAGAAGAAAAAACAAGCGGGGGAGAAACCGACAGATTCGACCCAAAAGCCAACTAAAACAGCAGATAATAGTCCTAGAACATATGATAAGGATACAGAGGTCTTCATTGCCATGGCACAGGAGTTGAAGGATGAGGGTAATAGGTTGTTTCAGAAGAGGGATCCTGAAGGCGCTTTATTAAAGTATGAGAAGGCTCTTAAGTTGTTACCGAGAAATCACATTGATGTAGCTTATCTTCGGAGCAACACGGCTGCTTGCTACATGCAAATGGGACTTAGTGATTATCCTCGTGCTGTGCATGAATGTAATTTAGCCCTTGAAGTTGCTCCAAAGTACAGTAAGGCTCTTATAAAGAGGGCTAGGTGTTATGAGGGGCTGAATAGGGCGGAGTTGGCTTTAAGAGATGTTAATGCAGTTCTATCCGTTGAGCCAAACAACCTTACAGCCTTGGAAATAGCGGAAAGGCTAAAGCAGACTGTAGACAGCAGAGATGATAGGGTTTTTGATCATCCTGTTCCTGAGTATGTTGAATCTCCTAGAGCTCCAGCTCCACTCCAAGCTGTGAAAGAGACCTCAAAGAAGAGTAAGCACTCTAAATATGAAGAGAAGGCACAGGACGATGGAGTTTGCTCAGAGGATGTAAAGGAGAAACAAGTTGAATACAAGGACAAAAGAGCCAAGATTAAGGATGAAAAGAAAGCTAGGTCCGAGGATAAGAAAAAGAAAAGGTCGAAAACCGATGTAGAAAAGAAAGAAAAAGATCACCAGCAGCGAGAAGTTGAGGATCTGGTCGAGAAGAAAGCCGAAGACAAGGTGGTGGTTGAGGAGAAAGTGTCTTATGCAAATGGAGGTGAGCTGAAGAATAATGTGAAATTGGTATTTGGAGAGGATATTAGGTGGGCACAACTACCTGTAAATTGTAATTTCTTGAAATTGAGGGAGGTCATACAGGAGAGATTTCCAACCTCGAAGGCAATTCTTGTCAAATATAAAGATTACGAGGGTGACATGATCACTATTACTTCTACTGATGAGCTAAGATTGGCTGAAGGATCTGCAGATCCCCAAGGTTCCATAAAGATTTACATAGTAGAAGTTAGTCCAGAGCATGACCCCCTGTATGAGGAGTTAAGGGGGAAAGATGGACTTGAGACAGAAAATTGTTTATCAGGGAATGGAAGTGTTAGTAAAAAGGTGGATCAGGAGAAATTGTGCATTGAAGAATGGATTATCCCCTTTGCTCAATTGTTTAAGAATTATGTTGGATTCAGCTCTGATGCGTACTTGGATCTTCATGAGCTCGGGGTGAAGCTCTATACTGAGGCCATGGAAGAGACAGTGTGTTGTGAAGAAGCTCAGGTCCTTTTTGATTCAGCAGCTGAAAAGTTTCAAGAAATGGCAGCATTGGCACTGTTTAATTGGGGAAGCATTCACATGGCACGGGCAAGGAAGCGAGTGCATTTTGTAGAAGATTCAACGCAAGAAATTCTGTTCGAACAGGTAAAAATTGCCTATGATTGGGCTCAAGTGGAATATGCGAAAGCAGGGCAGAAATATGAAGAAGCGCTCAAGATAAAGCCTAATTTTTACGAAGCTCATCTAGCTCTAGCACTACAACAATTTGAGCAGGCAAAACTTGCTTGGTACTATGCAGTTGCAAACAATGTTGATATGGACACATGGTCGCCAACTGAAGTTCTAGATTTGTATAATAATGCTGAGGAAAACATAGAGAAAGGCATGCTAATGTGGGAGGAACTAGAACTGCAGAATCAAAATTCACTCTCCAACCCCAGCAATGGCTATGCTCAGATGCAGCGGATGGGTTTTGATAGTCTATTTAAAGATATACCAACTATTGACAGTGAAGAGCAAGCCATGAGTATCAGATCCCAGGTCAATGTTCTATGGGGTACCTTGCTTTATGAACGATCTATAATGGAGTATAAGTTAGGCCTGCCAGTGTGGCATGAATGTCTCGAGGTTGCTCTTGAAAAATTCGAACTTGCGGGAGCTTCTCAGACTGATTTAGCTGTAATGGTGAAGCATCACTGTTCCAATGATACTGGGCTAGAAGGTGTAGGTTTCAGGATTGATGAGATTGTACAAGCATGGAATGAAATGGATGATGCAAAAAGGTGGTACACTGGCATTCAATCATTTCGACTTGAACCATTACTGCGGAGAAGGGTGTCAAAAATATACTACGCACTTGACCAAGCATGATTAAGAAGTTTCTGTCAATTCAGAACTATGTCCCAGCACTCGAGTTACCTCTGAAGATCGAGTACCAAAATGTGCAGGTGTATATAAGTTTATATAGTGAGGTCGGATGCTTTTTTTAAAAAAAAAAAAACCGTGTGCACCAATCTACAAAACCCTTATTTGCAAAAAGGGAGGTTAGTTGCAATTTTGTTGTTTGTACATTTTCATACTTAATCCTGCGAGTAGAAGTAGCTGTTGCTCGTCACGTTGTGGTAGATTAGAAGCAGCTGTTGCAAAAAGGGAGATTAGAAGCAGCTGTTGCTCGTCACATTGTGGTAGCCTGTTGCCCGTCACATCTCCATTGCAAGAACCGAAAGGCTTTATATTACTTGATAGTGTTACTGTATTCATTTTCAGCAAGTGAAGCTTTCGTTTTTGTTTTTGAGCCGTAAGGTGTGGCTAATTGGCAATGCACACACCTTTCGATTCTGAATTCCAAGATCAAAAGCTCATAAAGATCACAACAAAGAAAAGGTTTGTAGTTATTGTAGTGTGTTTTTTTCGGTTCTCACTTTCGACCCTTGTTGTAGGAAGGGGACTTGAGAAGAGGTTTATTTTTGTAAAATTTAATTTTAAAGCTATGTATGATTATATAACTTGAAAGGTTTGTAACTGTATCAAGCATAGCATGATTGTCCATTACAC

*S.maritima*1698407

CAGGGTCAGGGCAGGTCCAGGGAGGGGCCAGCGCCAGGCAGGGCCAGGGCACAACCAGGGCAGGGTAAGGCAGGGCAACAGTACCAATGGGCCATGGCAGGGTGAGGTCAGCGCCAGGAGAGGACAGAACAGGCAGGGCCATGGTGTGGCCAGGGCAGGAAAGAGCCAAAGCAGGGCCAGGACAAATCAGGGCGAGGACATGTCCAAGGCCAGCTCAGGGCCAGAACAGGACCAGGACCGTGACCATTGGCAGGACCAGGGTCACGACAAGAGACAGGGCCAGAGCCAAGGCAGGGCCAGTGCAGGTTCAGGGAAGGTCCTGGCCAGGGCAGGGCAGGACCGGGACAG

*S.maritima*1271050

CGCTTTTTTGCCAAACGTTTGGCTTTGATTGCAGCAATTTTTTCAACAGACATAGCTTCCGAAAGAGATTTGATATTGTCAACATTGACAGCAGACTCTTTAGGTGCATCTAAACGTGCAGCCAACTGTTGTTTAACCCTTTGAACTTGTAAAGTATCTTCCATACGAGGTTTTTTAGCAACTGATTCTTGTACATCTTCCACAGCACTTCGTTTAACCTGAGTTGGAATTTCAAGAGGAGCACTGCGATCGATACTAGCAGATGTAGATAGTTCCCCATTTAGGTAAGCTAGGAGTTCCCTTCGATCTGGTCTTCTTACAACTGGTATATTTTCAGCAGCTGCTTGTCGCACATATACAGGATGCGTGAGTGTAACATTCTTTAATAAAAATAATAGACATTCCAATGTATAATACTCTTTTGGTGCACCATCTTTACCAGAACCATACATCAAGAAGTTTGTATTAACAGTTTTTGACCAAGATAAGTCACCAAATAATATGTGATTGTCCTTAATTATGATTTCTTTTTTGTTAATATTGTATGTCCTTAAGAAAGTTAAT

*S.maritima*37714

TAGCATTTCCCTCCATCTAACAAGTCTTCTGTTGTTTAAATTGGTGTCCTGATTTCATGAATGAATGTCATAGTATACACGAGGAATCATCAACAAAGAACAATATATACAAGGTGATCCACCATCAAATGGTTGATAACATGATAAATCGAAAGAAGAAAGAAGGGGCAAAAGTAGGAAGAGTGCCCTTAAGAAGAAAGCAGTTCCCATCAATCTAAAAATGTCGTCTATCAGCAGGTCATATACAAGCAGGCCGAAGATTGATTCTATGATCGAAAAAAAGATTCGAGTTAAGAAACTAAGATGACTAGTTGTATAGGAGATTGTAAGCTGGCATATCTTGTACCCTCTGTACATCTTCCAAAGACAATTCTCTTTCTAGTTGTGTACGTGTTGCGTGTAAAACATCATTGAATTGCATGTAGGAAGCATTCATAGCAAGCCATTGACCATCCATCATTTCAAAGGCTATACAGAATAGAACGTCAAAGGCCTCTTCATCTTCTCCTAGCATTTTAACGAAATTCCTTCCCGGAATGCATCTTGGTTTATCTGAGAATATATCCAACATCTGTATCAACATAAAAGTCACATTAATCCCAGCGACGGCAAAAGGGTATTCCCACGTCGCTCTCCTGCCATCTTGCTTGAACAATAGCCGGTGGAAAGAGGCAGGGTATGTTCGGGCAAAAAAGAGCAAATTCTCCAGTGATATGTAGCCACACCCCCTAAAGTCTGTGGATGGATTTGGGCCTTGCCAGCCCATATCTTTCCATTGCTCTGAGACCATACCTGTGAGAGGAACGTTTGGAAAGGCAGCATACCACAATGCTGTTAGCGATTCTTGATGATCAGGGCGACTTTCATCAAAGGGAAGATGACATCTGTCTCGCAGCCTTTGCAGCCTTTGTTCCTGCATAGAATTTAATTGGTAGTCAGGAAACTTGTCACTTCCATGACGCCTAAGACCTGAGCGATGGAGAATTCCGCCAAACCATGTTCGGGATCCAACCATAGCATTAGTAAAACATTGAGAAATCTGAGATATTAGGCTAGCAGAGTTATGCACCAATTCCAAGTCATTGTCATATCTCTTCTTTCTCCGATAAATCTCATCCTCATAAACATTACGAAGGGAGGAACAAGAAGGGATACATTGACGATGCGTAATTCTCATTTAGGAATAAAACCCTTCAAATTTAGGGATCTTCCAAACAGAAAGAATGAAAGGAGACAAAAAGTAGGGAAATAGAAAAGATTAAAAAGGGTATACCTAAAAAAACCATTGTTTTCAACCCATGCTCAACAACCAAAATTTCAAAG

*S.maritima*40017

TTTTGGGGGAAACCAACGATAAATATAAAGTTAGAAGCCCAGCGTTATTAGCCTTCCCTTTCCTCTTCTCTCTCCTTTCTTTCTGTTCTTCATCGTCAAAGATCTATTATTTTCTGGCGATTAATCCCTAAATTGGTATTTAACTGTTTGCTGATTATAATTGCCCGCCAAAATTAAAGTATTCAACTTTTGTTTGATGATATCAAGCTATAGGAGCAATTATCATTTTCTTATATACCAAACATGAATGAAAGAGATATGATGTGTAGCACCCAACGAGTTCTTGATTTAACAAGTGGACAGCAAGTCCAGAGTCATCTCCATCCTGAGCCCTGCATTATTATGGGAAGCACGTCTAATATTCCCCGGTCGCACGTAAATCAGGTGTTACCTCCATCTGGCAGTGTGAACAACTTTGATTTCCACCGCTTACCTGAGCATGTTGGTAGGCCAGCTTTTTATGCGATGCCACAGTATAATCAGCATAATCATCCAGCTACAAACCACTCCAACTTTATGAATCTGCACTTGGACCCTGCTTCTGGGGCTAGAGTGTTTCCTGTGCCAATTAGCCACGGATTTGCGGACCATTTACCTTCTTCTAGTAATTATGGAGCCAATGCGGTTCCCTTTAATGAACATGTGAGGAGCAATTACCCAGCAGAAGGTGTTAGAGATTTATGTAAGAGAAAAAATACTGATGGCATCCCAGGGAATATTCAGCATTTAGGTGCTTCTGCAGGACCCAGTAGTTCTTCAGGTGCTGCTATGGCTAACTGTCATTTTGATTCTGGGTCTGCTTATATGGATGCACTGCCCATGACTGATTACAGAGGTAATGGCGCTCCTCCAGTGATGGAAGTAGGAGCACGGAATAGGTCTGGTGGTGTTGGGCTCCATATGGATTCTGGTGTGCATAATTATAATAACTATTTACTTCAAGCAAATTATGGAGGTCAGTCGTTTCAGCCATCTAGTTGCACATGGGTGGATCAACAGTTAATAAGTAATGCTGGGAGTTTACCATGGAGTGGTCCTCATCCAATGATGTCTTATTTTCAAGGGAGCAATGTTCATGGAGGATCATCTGAGTTGGCAAATTCCAGTCGTTCTGGATATCTTGAAAGATCTAGTAATCATGGCTCTGCAAATTTCTTGCCCACGCCACCTGTTGTACCTCAGCACCACAGTCTTCATCCTTCACAGAATATTCAGGGAATGGGAGGTTATGGTGTCGCTTGCTATCCTCCTTTTCTTGCAGCTCCAACTAGAGTTCCCCCTAGTGATGGTATGCAGCATGATGTTTTGTCTTTATCCCAAGAAAGTGCAGAACAGGCAAATCGGCCGACAGGCCTTAGAATATATCGGTCTCAAGGAAGAAATGTTGTGCCTGATTTAGCTTCCAGACGCCGTGACCTTCCTCGTTTAAGGACTCTTCAAGCAGATAGTGTTGCACTTTTGGAATTCTCAAACTATTATGAAGTAGACTACAATGTCGAGAACTTCATTGATCATCATAGTGATATGCGTTTGGATATTGACGACATGTCTTATGAGGAGCTCCTTGCTTTGAGTGAGAGGATTGGAACAGTAAATACTGGGCTTACTGAGAAAGACATCATATGCCATCTAAAAACGAAACTATATAACTCTACTGGAGCTACTTTCAATTTAGAGGAATTACCTAGTATTGATCAAGACAGTGATTCTTGTATTATATGCCAGGATGAATACGAAGATAATGATAAGCTGGGAACTCTGGATTGTGGACACGATTACCATGTAGATTGCTTGAAGAAGTGGCTACTCTTGAAGAATGTGTGCCCAATTTGTAAATCTCGGGCCTTGGCTGTGAATCAAGGGAACAAATAAATGCTCAAAGTCGGGTATCTTGGATGAAGGAAATCGAAAATCTTCTCCTACTTATCTATTTGCTTTTAACTAAGTAGAACATATATCGTACATATATTTGGAGATGAACTTGTATAAAACCATTTATTCGGTGCACTTCTGTCATAAGAGGTGTGAGTTGCAGCCCTCATCAGCAACTTATCTGTAGTGTTCCTTCCCAAAATGTTGTGGAACTGTACAGACTTTTGTAAAAATCTATACTATTCTGTCATTAAAAATTTTCCCGTCTATCC

*S.maritima*6203

GGACAATTGGTGTTGCCGCCTTGGTCAAGGATTTTCCTATCAACCGGAATTGTGCAGCTATCTTTTCCTAAACACATCTGCTCAACAATCTTTTGTGTGTTGGGAGAATTGCAAGTGCCAAGGAGGAAGAAGCCACAAACACCACGTGCGTTGCCAAAGCTAGCAAACTCAACACGGCTAATAACCTTGTGTTTAGGGCAGTTGAGTATTGCCTTGGTCTTAGGGTCATCATCAACCGGAACAATGGCATCATGACCATCTCTTTTCCATGACATTACGCTGGGTGGCATGTCCTCTGTTATGTAGCTACAGATTACATCTCGATTCACTGTCTCTATTTGAACCGTGTCTATATTACCTCCTGCCTCATCGAAGATCACCAAGAAGTTGTCCTTAGGTTTCAAGTATGACCTTGGAATATGGTACTCGCTTTGAGAAGGTGCATTCCTTATTGTAAGAAAGCTCGACCAGTAACGTCCGATGCTTTTTCCATTGACCCAAACCACTCCTTTGGTCATGTTCTCCAATCTGATGGCTACAGGATCCTTTCCCTCTGGTTCATCAAAGTATGCCTTGTACCAAGTAAGAAATTCCCCATTGCCTTTAGCTGGGGTCCACTGAACCTTCTTTGCTCCATCCTCAGTGAAGTACTCGTGCTTCTCTCCATGCAAACCAAGCGTGTGATGCCACCCATTAGAAGAAAGATCGAGCTCTCCGGTACCTAAACCTTGAATTTCTAGGAATTTTAGCCCTGTCCATCTCCTTTCCATATAGCTTCCGCTATCTGGCATTCCCATTGTGTTGCTCAAAATTTCAATGGTGTTGACACCTTCCCTAAGCTTTACTGGTGATTGAAAAGTGAAGGCGGGACCTCTATGGAACCCATGTCCCGAACCTATGTAAACTCCATTGACAAATGCTTGCAAGGAATGACCAAGATGGTAAAGCTGTATGACTGGTTGGAGGGACTTTCTGAAGGGCAAGTCAACGCTGTCCAATTCTACACTAGTTTTGTACCACATATAGTCAGTTCTGTCCTCGGCAGTATGAAACTGCTCCATCGGCATCCTTGAGTAGATATTAGATTTAGTAGGGAAAATCTCTTTCGTCTTTTCCCATTTCAAATCCTTCATAGCCTTTTCTGATCTTACAAATTCCCTTGCACTATGTTGTGCGACCATAGAATCAGTGTTAAAGACAACAGTCTTGCAATCAGGTAAAACGCTGATAGACTTAGGTGGAAGAGTGAAGTCTTTGCCTTTGAATTTCACAGTTTCTGCTGTATGAGTGCGGTTGTTCCACAGGAAAGCCGCGCAAAGGTTTTGCTTGTGGTAGTAACGTGCCTCCAAATCGGGGCCAAAAACTTCGACATTATATTTCCCAAGAAAAATGGCCTTCTGGCATAGAGTTACAGCATAATGCAGATCCCTCAAGTGTCCCCATTTGGGAGCTCTTACCAATCCAAATTCATCAAGAGGAGCTTCATCATAGTAACGAGTGGTCGTAAAGGCAGCAGCTGTTCTTCCATAGTTAGTGCCACCATGATACATGTAATAGTTTGTATGGCTTCCATTCTTGGAGAACCAGCGAGCCACTGCAAAGGCGGTATCCTCAGCTGACCTTTGAGAAGGTACATCTCCGAAAACTCTGAACTGAGCTGTCCAGTTCTCCGTCCACAGAGATGGCTTGTTAGCTGCATTTGGACCTTCAAATGTTTCTCCACAATGTCTACCATTGCAAGCATTGATCACTTCTGGGGGTGCATCTTTAGATTTGCACATAAGCCATGGAACATCAGTTTTGAGGCCGACAGCCATTTGCCCTGCCCATGTGATGTATCTCTCTGCCGCATCATCAAAAGCTTCTCTTACGTGATCGTACTCATTCTCAATCTGAGACATAATAATAGGACCTCCTTGTGGAGCATATAACTTGCTATCCTTCATCAAATTTACTACCTTGGTGACCCATTTCTCCATGTTCGCCTTGTATGCTGGATTATCGGTT

*S.maritima*28679

CATCGCACGAGTCCTATATTAGTAAAGAAATAAAACAAAATATTGTTGCAACTTAAAAATCTACAAGGTAGTGCAATTTTCTTTAAGACTAAGGTGTCTAATCATATTAAACCAAGCATACATGTTCTTTCTACTGGTAATTTGCATTTGTTGCACGACTTGTGCCTCTTCGTGTACATATTATACAATGTACATGAAACATCCTGCAATCTTCTAACCTTCAAAACAAAAATGTCAGCTACATTCTAAGAAGATAGGTCAGTCTTAAATACCCCTCCCCACAAAAAAATAAAATAAAAAAATAAATAAAAATAAAAATAATGGGAAGTCCAAAATTGGTGAAGTTTTTCTAGTCAAAAACAACCCATAAAAACAGATTAATAATAAGGTGATTTATAAATTGAAAAAAATTTATAAATTTTGTCAAAAATCATAATTTTGAGTGGCAAAGGGTTGAAACAACTTCAGCCTTCAAAAGGCAAACTTCAGTCACCAGAATCAAACTAAGCCATAGAAGAGAGAAAGAAAGAGATACACAAAACCCAAATTACTTGTATTATTACTCAATTTTAGTATTATCATTGATTGATTCATTTGTTTTTTTTTTGTTAAATTTTAGTTTTTTTTTTGTTGAATTTTTTGTAAAAAAAGTTTCTTTCTTTTCTTTCTTGTTGAATTCTACCATGGATCCATTTGATTCAGTTTCGTTCATCATCGAGCTTCGTAAAGGTTTTGGGTAGTAGATGAGAAGGCTTTCCTCTTGTTCTGCTTTGGTATTCCGTATTTGTATATCTACTAGACTTCTCTCCATCTTTATAAAGAAATGAGCTTATCCAGTCAAGGTTCTCCGACAACCCAAACCAAGTTGAGTTCAGCTACTATGGGAACTTCTTTGAATCCAAATGCTGCCGAATTTGTTCCCTTTTCTCTCAGAACGACTCCTGGGACTTCATCAAAGCTTCCTGCTGCTGGATCGTCTGGAAAACGAGTATTAGACCGGTCAGAGTCCTCTATTTCAACCAATTCCGATGAAGAGGCAAGGCAGTATTGGCAGCAGCAACTACCTGATGACATCACTCCGGACTTTAAGGTCATGGGAGAGGATGAACATAATGGAGCAGGTAACCTTTCACTTGCTGGCCTGTCACTGCATGGTAGCAATGAAATCAATAGGTTCTCTGCTTCTTTGGGTAGTGGCTACAAGATGAATGAGGCTTTTGAGTTAGCTGCTCACCATTCTTCAAATGGGGGTGCATACAGTGATAAGATGGGGTATCCCTTTTCTGGATATGTAGAAGATCCATCGCCAACAGGTTATCTTCAGAGGACCAATAGTAAGCCTTGGGATACACAACTTCTCAATTCTGAGCATCTCTATGGGAATGGCAGGGATGGGAAATCTTTGGATGAAAATGTGAGATGTGGCAATCCTAATGATTTGACTGGTGAATCCTTAATAGAGAACAACGGTATGAATCCTGTAGAATTTCTAGCTTCGCAGTTTCCGGGTTTTGCTGCTGAAAGCCTGGCTGAGGTGTATTTTGCCAATGGTTATGACCTGAATTTGACTATTGAGATGCTAACTCAGCTTGAGCTTCAAGTTGATGGACCTTTTAATCAAAATATTAATTCGAAGAACTTGTCAACTCCAAACCTGACGTCAATGGATTTCCCTGCACTTCCTATTCCAGACAGTAAGACCAACCTGCGGAATTACACTGGAGAAGAACTCCAGCAGTCTGCTAATCCTTATGGATCCTCAGAGAAGGACAATATGCTTTTTTTTAGATCCAGTTCTTCCTTAAGTGCTAAGGGTGCAACTGATTTTGCTTCAGCAGTTCGTAAGGTAGCCCCCCAAGATTCTTCCTTGTGGAAGTATGACAGAAGTGGTTCAGGTGATGTTGCTGTTGGTTCTAGTAGAAGCTCTCATGCTCTATCTGGGTCTTACAACACTGGTGCTGGACGAAATATTTATAGCGATAGGTTGCAGGGTCGTAGCTCTGCTCGAGCTGCTCCTGTATGGCTTGAAACTGGAGATGCAGTTGCCAATTTATATTCTGAAGTCCGGGGAGAAGCACGTGATCATGCACGCATACGAAACATGTATCTCGAACAGGCTCGTGAGGCTTACCTTATGGGAAACAAAGCATTAGCCAAGGAACTTAGTGTTAAAGGACAGCTGCATAACATGCAGATGAAAGCAGCTCATAGCAAAGCTCAGGAATCAATATATCGTCAGAGAAACCCAAATGGTCTAGAACAGCAAGGTGGTGGCAGATATCAGGAGAAGATGATAGACCTGCATGGGCTCCATGTGAGTGAAGCTATTCATGTGCTGAAGCAGGAGCTGAATGCATTGAGGAATGCAGCAAGGTTGGCGGAGCAGCGGTTGCATGTTTATGTATGTGTTGGTACAGGACACCATACCCGAGGTTCTCGCACTCCTGCAAGACTTCCTATTGCTGTTCAGCGCTACTTGCTTGAAGAAGAGGGTCTAGAGTTCCATGAAGCGCAACCAGGGTTGCTTCGTATTGTGATATACTGAATTAGGGTAAGAAAACTTGTGTATGATTAGGGTTTTTGTTGTTGACACAGAAAGTCATCCATCACTCTTTTAAATCATCATTTTTGTGCATCTGTACATGGGAAGGAAAAAGAATAGTGCATATTCAGGAGAAAACGAAAAGGAAAAAAAAAGACGGAGGGGATAATCCCAAGGAGAAGTGAAAATGTTGATAGGTGAAGAGACGAGGATATGGAGTCAGGCATGTAACATTTTATTTAGTAGCAGCAACACATTATGGATGATAGTGGAATGTATCTTCGACTCTTTGCCTGTCGGTAAATCTTTTGTAACAACCACCCAAATCCCCCTTGTCCAAAAAAAAGTCGTTCAGAACTTTGTCATAGATTGATTCTGTATCTTAAACTTGTTTAGTTTCACAAACCCCCTCTCCTAGACTCCTAGTAGGTTACATGTATCCAAATAGAAGTCATTCAAATTAGCAAATCGAATTTATTATAGTACATGCCCAATTCTTGTCAA

*S.maritima*45135

ATAGCTTCTTCAGCAGATGGCCTTCGTCAATCTCTCTCTGTAACATAAACAAAAGGGATATGCTGAATCTTGGGCCAGTCGCTACCATTTGGGCGTTGACATCGTTCTGTCAATCCTTTACACCTTCTGATTGTGAGTTGGTTTAAGGTGGTGAGGTTGCGGATGGCTTCTGGTAGACTTTTCAATTTGGGACATTCAATTAATACCAGATTAGTGAGAGAGGTGAGACAACTTATCCATTCTGGTACTTCCTTCAATTCAAAATTCCAAAATAATTTCAAAGAACGGAGGTTTGACAAGTGTTGAAGCCCACTCGGAAGACCCACTAGTTTTTTAAGATCAATTAATTCCAAAGAACGAAGGCTTGTATTGAAGGCTATCCATGGCATACCCTTACCAATACCATTACCA

*S.maritima*32946

TTTTTTTTAAACCAGAACAAATGCAAAAATCTCCATTAACAAAGTTGAAATAATTCACAAAAATTAACTACCGATTGATCCATGTGATTACAGAGTTGGGTGGTGCAAAAAAAACATTAAATGGCAAGCTTTTATAATAAAAAAAAACTATCACTTCACTTGGTGCAAAAAAATGGCAAAGCTTTGTCATTGTTCCTAAGCATGGATCAATTCTTCCATGGAGTTCCCTTCCATCTATATCTCATACTATTCTCTATTTAACTTGAACAATTCCGGCACTGACTAGCTTTTGAACCTTGTTCATCACCCCTGGATTCTTCATATGCTCCTGTGCTGCCTTGGGGTTCTCCTGGAAGTCTATCAAAACCTGTCTCATTACAGGATCAGTGAGAATGTTTTGGATTTCTGGGTCCTGCATTGCTTTAGCCTGTCTCTCTTTGAGTTCATCAGGAGTCAAATCCCCACGGCTTGCTTTATTGATTTGATCAACACATCTTCTCACACCATCCAACAATTCCTGGTTATTGGGATCGTGTTTTATTCCTTCTTGATAAGTTTCTAATGCCTTCTCATGTTCCTTCATGAAGAACTGGACTGCAGCCTTTCTAGTGTATCCCTTGACAAAGGTAGGATCAAGCTCAATGCACTTCTCAGCATCTTTTAATGCTTCTGGCATTGCCCCCAACTTGATGTAGCACGCAGCTCTGTTGCTGTAACCCCTTGGATCCTTAGGATTTCTCTTAATAGCTTCAGCATAATGTTTCACTGCATCAGGATATTTCTGCTCCTTGAAATACTCATTTCCTTTCTCACGCTCTTCGTCAGCTATTTTTGGATCAAAATACTCTTGTTGCTCCAAATCTTTCTTTGCCTTTTCAGCATCATTCAACTTCTTAAGAGTGTCTGGATTACGGTGCTCAGTCAGAGCTTTGTGGAAAGCTTCAATAGCAGGATCATAGTCTTTCGAAGTCTTTGCCATTTTCGCCAAAGCAGTTCCTTTTCTGGTCAAGGCCCTGGCTACCATTTTGTAGTCTGATCGAAGCTCCCTGCCTCTTTCAACAGCTTTATCACAGTCCTTGATACACTCATCATACTTTCCCATCTCAAGATAAACAGCAGCTCTATTAGTCAAGAAAGAAATATCCTCATCATCCAATTCAATAGCTTTACTATAATGTTCAATTGCACTTTCAAATTCCTTCTTTTTATAAGCACCATTCCCAGCTTCCTTCTCCTTTTGTGCCTTTGCTTTCCTCTCCTTCTTCTCTCTCACCTCCTCATCCTCCTCCTCCACCATGGGCTCAGGTTCGGGCTCAGGTTTGGGTTCAGGCTTAGGCTTGGGCTCGGGCTTCCTGGTTTCCCTCTCAACTTTGAGCTCATCGGGCTGAACATCCATATCTTCAGGCCCACCCGACCTCAATTTAATGTTCAACAAAACCCCAAGTGCTTGCATAACCCTTTGATCCTTCAAATACATGTTCAAATTCTCAGGGTTCTTCTGAATTTCCTGCATCATCTTCACAAAATCGGGTTGCTGTAGATAAACCCGGGTTGACGGGTCAGCGGTAAGCTTAGCCCACATCTCCGGGCCGGAGAAAGCGTCCCCAAAAGGCGAAGCGGCGCTACTAGGCCTAGAACTGCGAGAGGCAGCAGCTTCAGCGTCGGAAAGCCCAGATTTAAGAGCTTCGTTATTTGGGTCAATTTCAAGGCCCTTTTTGTAAGCGGAAATAGCTTCGGAGAAATTATGGAGACCCAAATAAGCAGACCCTAAACGACTGTAACCCTTGGCCCAATCGGGCTTAAGGTCGACGGTTTTCTGGGCATCAGACAAAGCGTCGGAATATTGACCTAAAGAAGCGTAGGAAGCAGATCGATTGGAGTAAAGGACATGGTTAGAGGGCGCAAGAGTGATTGCTTCGGTGAAGTGTTTTATTGCTTCAGTGTAGTTTCCTGATGAAAACGCTGCGTTTCCTTTGGCTTTTGCTTCGTCTGCCATTGATGTGATTTTTAGAGAGAGAAAACGAAGATTTTAGAGAGAGAAAGATTGGGGTTTAGAGAGAAGAGAGTGGAAAAAGAGGGGTTAAATAATAGGGAAAGGGAAGAGGAGAAGACGAGAGAGTTCTGGGGAAGAGGTGAAAGGGTTCTGGAATGTTCTTTGGGTGACTTTGATTTTTGAGT

*S.maritima*53673

AAAAAAAAAAAAAAAAAATGGAAATTTCCTTCAATCTAAACCCTAATCAACTTCAATCATTCGATTCTCAAGGTTATTTGGTATTAGATTCATTTGCAAGCTCACATGAAATTGCAGCTATGAGAAGCAGAATGGAACAGTTACTCGATGAATTTGACTGCTCTACTCCTGTCATCTTCTCCACTAAAAATCATAGTCATGCCAAAGACGAATACTTCTTCGATAGTGCGGAGAAGGTCTCGTTTTTCTTCGAAGAAAAAGCATTTGATGACGATGGTAGATTAAAGCAGCCAAAACAACTTTCTATTAATAAAGTTGGTCATGCACTACATGACCTTGAACCAGTTTTCAAAGAGTTTTCCTGCTCAAAGAATTTCTCAAGTTTGCTCTCAAGTTTGCATTACAAAAGGCCAGTGGTCATTCAGTCAATGTACATTTTCAAGCAACCTGGCATTGGTGGTGAAGTGGTGCCACACCAAGACAATTCATTTCTTTATACCAATCCACCTTCATGCACAGGGTTTTGGCTCGCTTTAGAAGATGCCACAGTAATCAATGGCTGCCTTTGGGCCATCCCTGGTTCTCATAAAAATGGCCTTGTGAGGAGATTCATTAGAGATGATGAAGGGGTCCATTTTGACCATCCATCTCCATCATATGACCAGAATGATTTTGTTCCTATTGAAGTTAAAGCTGGTTCCTTGGTTATCATTCATGGTGACCTTATTCATCAAAGCTTTGAGAACCAGTCACCAAATTCAAGGCATGCATATAGCTTTCATGTGGTGGAGACGGATGGCTGCAAATGGGAAGAAGATAATTGGATTAGAAGAAAGCAGGATCCAGAGCCTATATATGTATCCTGATATATGTGGCTCCTCTACTGGCCAGTTCAATCAACACATCCCACACAGTTGCAAGTTTTACTCTGTGGCAACAGTTGCACCGTTTTAAATAGAATAAGGGATTTCGTCTGATAGACCTTTTATATGGCAATATGCCAGT

*S.maritima*25402

GTGGACAATCTCATCGTCGGTGCTGACTGCTGCCTCCTTCCTCTTTCCCTCTCTCTTACACCCTACCATGTTCCTTTACTTTACTCATATCTTCTTCTGATCTTCTTCCTTCTCATTTCATTCAAAATTCCCTCAAATCTCAATTTTCAATCCAAATTCAAACAACCCCTTTTTTTCCCAGATCCCAATAATGGGCCAAGAGGGTTTCACGGCCCAAAAACGGCCCACAGCAGGCGGCGGAACTCTCCCCACCACCACCTTATCCTCCTCCAATGGCCGCAACTCCCGCGGTGGTCCCCGCACTCGCCAAATAGCCAAAACCATGAACAACATCAAGATCACCATCTTATGTGGGTTCGTAACAATCCTTGTTCTTCGAGGCACTATAGGGCTGAATCTAAGTGGGTCCTCCATTGATGCGGAGAAGCAAGCTCTTGAGGAGGAAGCGAGACGGGTTATAGCTGAGATTCGGTCCGATGGCGCTGATGTTGATGATGAACCTCCCTTCAATCCCAACATAACCTATACATTTGGTCCAAAGATTGTGAATTGGGATCAACAGAGGAAAGAGTGGCTTAATTCGAATAAAGAGCACCCTAATTTTGTCAATGGCAAACCTCGGATGATGTTGGTAAGTGGTTCTTCACCTAACCCTTGTGATAACCCAATTGGGGATCATTATTTGTTAAAGAGTATTAAGAATAAGATTGATTATTGTAGAATTCATGGGATTGAAATTGTGTATAATATGGCTCATTTGGATAAGGATTTATCTGGGTATTGGTCTAAATTGCCGTTGATTAGACGGTTGATGTTGTCGCACCCCGAAATCGAGTGGATATGGTGGATGGATAGTGATGCATTGTTTACTGATATGGTGTTTGAGA

*S.maritima*346393

GCGCCCACGCGCACCCACGTCACGTCACACACCTGCAGTCACTACTGCGCTATGGGTCAGCCGATGTACTTGTCGCTGATGGCCACCACAGTCTGCCTGGTCACTTTGTTGGCCACGTGCCCCTCGTTGGCCGTCGGTTGGCCGGTCGATAGCAGTTTTTACAATGAGATCAAGCGCGTGGTGTACGGTGGTGACGAAAACGTCGACGACTCTGCTGCCGCCACTGAATCAACACCATTGCCACCACTGTCTTTACCGTTGTCGTCACAGGACATAAACAGGCCCGTGGAAGTAGAAGGATGGCCGAGGAGCCCGATACCTCGCCTCGGGCAAGTGAGCGGGGTGGCAATAAACACGGTTGGGCAGCCTGTGATTTTTCACCGGGGAAGCCGTGTCTGGGACGAAAATTCATTCAACGAAACGTTCCATTACCAACACATAGAAGAAGGCCCCATTTTAACAAACGCCATCGTCACTCTGAGTCCGAAAACCGGCGAGGTTTTATCTAGCTGGGGAGCAGGGTTCTTCTACATGCCCCACGGCATCACAATAGATCACCAGGGGAACGTATGGGTCACCGATGTGGCCATGCATCAAGTGTTTAAGTTTTCGCCCGGAGCGCCCAGAGCGTCATTAACCTTTGGCAAGAGATTCGAGAACGGCAAAGGATACAGGACATTGTGCCAACCTACTTCTGTGGCCATCGCTTCGACCGGTGACATTTTCATTGCTGACGGATACTGCAACTCTAGAGTTTTAAAGTTCACAAGCAGAGGAGAGTTGCTACGAGTTTTTCCTCACGCCAACGAATTTCTGAGCCTTCAAGTACCTCACAGCCTAGCGTTGCTCGAACAACACGACCTCGTCTGCATTGCTGACCGTGAAGATATGAGAGTCGTGTGTAGGGGTGCAGAGCTTTCGACAGAAAGCAAAGAACAAGCCCCGTTAACGATTCAACAGCCAGACTTGGGACGGGTGTACGCGATAACCAATCACGGCGGTTTGCTTTATGCCGTCAACGGACCTACATCACCCCTGATACCGGTACGAGGATTTACCATAAACCCTATGACCGAGAACATTGTCGATCATTGGACACTATCCAAAAATGCAGCTATCAATGTGCCGCACGACATAGCTATTTCGAAAGACGGTTCGTCGTTGTACGTAGCTTCCATCAGTCCGAACCGCATAATAAAATACACGATGACGTCAGTTACTCCATTGAAATCCGAATAATTCTCAAAACTCAAAATATTCTACACGAAAAACTACACAATAACAATATTTATTTTATTCC

*S.maritima*37367

TTTTTTTTACCTTAACACTGATTTCAATCGATTTGATATATGTCCAATTTACAGTTTAGCATGGTACAAAAATTTCAAAAAATCGGTACAAAAGAATAAGAGTTCATGTTTTTCTATCCCAAGATAGACACCCCAAGAACAATATTAAATCGCTAAATTAACAATTTTACATCGACTCAAACAACCCTATAATCAAATCAAGCCTTTGAGGTGCCCCCATGATCCCTCTCAACATGCTCAACAAGAGCAACAGGATCCCTAAATCCCCTGTTACATTTCGGGCATACGTCAATAGATAACTTCCTTACACCTGAGCGATTATTACTCTTTTCATGAGCTTTCTCTACATGTTCCACGAGCTTTGTAACTGACGAAAACCTTGCGTTACACTGTGGGCACACTTCTTTTCCCCCCGTCCCACTACTACTTGTGCTCGTGCTGCTTTTACTGCCTTGGCCTACCTGAAGTGTCTGATTAAAGTCAGTACTTAGCTTCGCCATGCCTGCTTCAGCAGAGGCTCGTGCTGATGAAGCTGCATTAACAAATCGGGACCATACTGATGATGTCGATTGTGCTGGAGCCCTATTAGGTCGTGGCACTTCTTTCCTGCTATTGCTGTTCATAAGTCCCATAAATGGGAATCCAGTGGACTTTGGAGGGCCTGAGCACTTGTGATCGGGCCCAAATCGATGTTTCAAGCAATGTTCCGTGTTACAGTCCTTGCATTTGACTGTATTAGAAAACACAAGGACTTCCTTGCAGCCAACAACAGGGCATTTCTTCTTCTTGGTTACTTTCTCGTAATTTGCTGGGTCACAGACAGTATTTACATGAGATTCCCAACTGATATTTGGGTCTTCATCAGGAATTAGGCGAACTCCTTTGGCACAAAGTGGGCAAATGACGACAGTAACACCTTGGTTGTCAGCGTGTGGACATTTGTGCTGCTTGTAGCTTCGATGCTCAAGGCAATATACCTGATCACAACGGTCGCAAGTAAACGGAAGAAAATCAATCTGCTTGCAACTATCAACTGAGCAATGTCTTCCCAAATTTGGGAATGCAGGAGTTCCCATAATCCAAATAAATTAAACCCCTTTTTTCAGAACCAAAATCAATTGTAATTTGAGTCAAACAGAGGGATTAAAGGAGGGATTTTTATATTTTTTGGGGTTTCTTTCGATTGAAGTGAAAAAAAAAACAGAGAAAATTGAAATTTTGGAAGAAGGTTTAAGGAAGAAGAAATAAAGTCGGCGAAAATCGGGATTTAGATTTAAGCAACGACGACGACCCGATTTCCGCGTGAAGGAGGGGATCTAGGAACAAGAAAAGTCGATAATGGACAAATACTTAACAACGAATCAAC

*S.maritima*35346

TGGAGGTGGAGGTAGCAAACAACGGCTAAGATGGACTGAGGAGCTTCACAACCGTTTTGTGGACGCCATTGCACAACTTGGTGGACCAGATAGGGCAACTCCAAAAGGAGTTTTAAGAGTGATGGGTGTACCTGGACTTACAATATATCATGTGAAGAGTCATTTACAGAAGTATCGCCTTGCAAAGTATTTGCCAGAGTCGCCTAGTGATGGTTCCAAGGATGAAAAGAAAGGCTCTTTAGATGGCCTGACAAACACGGATTCCTCCCAAGGATTGCAAATTAACGAGGCACTCAAAATGCAAATGGAGGTCCAGAAACGGCTTCATGAGCAGCTTGAGGTCCAAAGGCAGCTGCAAATGAGAATTGAAGCTCAGGGAAAGTATTTGCAGAAGATCATAGAGGAGCAGCAGAAACTGGGCAGTGTCCTAAAAGCTTCTGACTCACTGCCGACCAACGCTCAAGATGAGGAGAGAGGGAAGTCGTCAGATTCTCAGCCAGGTAAGGAAGCCTCCCCTGGGCCCACTTCTCCTGGGAAGAAGCAAAAGCTGGATGATTTGTCGACAAGCAACATCGATAACGTCTCGGATTCCTTAGAGAGCCCAAAGAAACCGTTCCTCGACCAGTGGGATGGGACTTTGTATGGCTCTGTTGCGGGGTTTGAGATTGATTTGGGATCAGAGTTTAAGAAGCAGGAAGACAGTGGACATGAACAAATAATTTCTTCGGAATTGGATTATGCTGGTAGTTCCAAGTAGGGCAGAAGGACATTTAGCAATTAGAGAGAGAGAGGACGTAATCCTGCATCATTCCGGTATGCAAACCTGTTTTGTACATAGTATTTATATACACATTTGTAACACTGTAATTTTACTTGCAGTTTAATTGTATGAATTGTCACTCCTTTGATGTTGAAGTGTTACATCTGTAGTTGAAAGTTTTCTGACTTTTGTACAACTCCCAAGAGTTCAATGACGTCCGAATTTTTCGACCAAGATACTTGGTATGAATGTTACACAAATTTCAGGAATTGCGACACTGTCTAGCAGCCCCAAGTCAAGGGAGT

*S.maritima*208405

CTCCTCTATTCAACTAAAGATCAATTCAAAGCTTTCTGATACTTTTCTCCCTTCAAGAGGAATCAGACAGGGTGATCCATTATCCCCATACATATTCATCATGTGTATGGAGTTTTTAACTAGAATGATTGAGCAAAGGTGTCAGAGAAATGAATGGAAACCCTTCAAATTCAGAGGGGGACAAACAAAGATTTCACATCTTATGTTTGCTGATGATATTCTATTATTTGGAGAAGCAAATGATACTACTCTCGAGGCTCTCACCAGCACCATTGATGAATTCTGTGATATGAGTGGCCAGAAGATAAATTGTACAAAGAGCAGGATCTATTTTTCCCCAAACACCCCCACTGCCATTAAGGATGATTTTGAACAAGCTCTTGACATTAATAGCACCACCTCCTTAGGCACATACTTGGGTTTTCCCCTCACCCACAAAAAACCAAAAAAGCAAGATTTACAATACATTATCCAAAAACTCCAAAATAAATTAGCATCCTGGAAAACCAATACACTGTCAAAAGCTGCTAGAAGTGTTCTCATCCAATCCACCTTACAAGCTTTACCAACCTATTCAATGCAATGCTTGGATTTACCCAAATCACTGCACATAAAAATTGACTCAATCACCTCAAAATTCTTTTGGGGGGACACTGTTAATAAAAGGAAAATCCATTGGGTAGGATGGGGGAAGTTATGCTCACCAAAAAACCAGGGTGGATTGGGGTTTCACAAGAGTGAAGACCTCAATAGAATTGCTTTAGCTAGAACATGTTGGCGGCTTGATCAGCATCAAAATTGGGCAGGAACAATCATTAGAGAGAAATATGTTACAACAAGAACAAACAATGCACCAGCTTCATTCAAAAATGGTTCTCATATATGGAAAGCTATTGGGAAAGGTTGGGAATTGTACAAACACAGTTTATGCTGGAATATTGGAGATGGCAAAACTTGCAATTTCTGGTTAGATAAGTGGTTTAATGAAGAGTCAATGAGAAGCAGCATCTCTGGCCCTCTAAACCAGCATGATGATCAACTTACTGTTGCAGAGGCTTTAGCTAACCCCTCATGTTTATCTTTCTCCCTTCCACCATCCTTGAACCATCAACTGAGCCTCCTCAACCTTTCTAATAACCCAGACACCTTGTATTGCAGCTGGTTCAGCAATGGGAAGTTTGATACTTCAAGGGCTACACATTTTATCTCATCTAGCTCAGAGGAAGACACAACTTGGATTTGGAAAGCTCCTGGGCACAATAAGATGAGACACTTCTTATGGCAACTGTGGCTCCAAAGATCACCAACAAACGCAAACATAGCCATCAGAATCCCTTCCCATAACCCCCACTGCACCTTTTGCCCCACAACACCAGAAAATGAAAACCACTTCTCCAGATCCTGCCCCAGAGCATTAGAGATCTGGTCTCTACTTTCTTACACCCCTCCCTCCACTTCTTTTGATACCTGGCTCAAACAAAATATAGCTGACACTACACCCTCATCCCTCCACATCCCATGGAATGTTGTATTTGTGTTCACAATCTGGCTACTTTGGAAGAGAAGAAATCTATGGGTGTTCTCTAGAAAAAATGTGAAAAAAGAGAAGTTACTACAGGACATTTCATGGTATGCATCAGAATGGTTCTACTCCCAACCTTCTGTTTCAACCAACCCATCCCCTCCTACACACACTTGGATACCCCCACCTCAGGGAATCCTCCAATGCAGTGTAGATGCCTCCTGGATCACAAAACATGACCCTGCAGCCATTAGCTTCATTTGTAGAAATTTTAAAGGAAGATGGATAGCAGGCGGCAGCAAACATGTTTATACCTCATCTCCAATTGAAGCTGAAATCATTGCAATCAAAGAATGCATCACCTGGTGTTCTTTAAATGCTATTAATAGAGTAGTGGTCCTCTCCGATAGCACCCAGGCTATTAATATGATCAAAGAACATTTCAATATTAAATGTAAATGGAGTAATATGATTGATGAATGCAGGCAGCACCTGACAAGCTCAACTGGGATTGAACTTCAGCACATCAAACGAGAAGGAAACAAGGCAGCTGACATTTTGGCAAAGACAACAAGGAAGCAAGCGAACTGGAATTTTAATTTATGTATTTTCAATCATCCTCCTAACTGTTGTTTAGATATTTTGGCTCATGATAGAGCAAGCATAGACATTATTGTACCTAATAGGCTGGGAAACTACACACCCACTGCCTCACTTAACTCTGATGTTGTTTAAGCTGCTTTAATATTAACTCCTTCCTTTCAAAAAAAAGAGAATGCATTAGTAGTGTTTATGAGTATTGTGTTTACAAGGACGACATACAACAAGGGGAAAAGGGTATAGAAATGCATGGCTTTGGCTTTCATACGTACTATTGG

*S.maritima*35358

AGGTTGCAGTGAGCCGAGATTGTTCCATTGCACTCCAGCCTGGGTGAGAGTGAGACTCTGTCTCAAAAAAGAAAAAAAAAAAAAGAAAGAAAAGAAGACCATATGCGTCCAGCAGTAGATCTTCCCACAGTTGATAGAAAATGGATGTGGCTTGTTTAAAAAAACGAAAATTTTTGCCCCATATCCTTCCAACATTTGAGACACAAACTCATTTACCAGCTCGAAATGACTCCTTAGTTCTGCCATAATACTCAGGTTTGGTTCCATGGAATGTGATGAAATTCCAGCCAAATGCAGAACCACCAACAACGTATGTTCCAGGAATTCGGTGACTGCCGTGAATTGGCTTTTCTTCAGGTAGTTGATCTGCAAGCTTCTGTCTACTGTGCTTCTCGCTGCTGCCAAAATCTGGATTTCCTGATACATTGTTGCATTTTGCTAGCGGCACATTTTCTCCTGCTGGCTGTCTGCCTAGGTCCACCATAAGCTTCATCTTTTCTTGGATTCTGCGGGCAGCTTCACAATTCTGAAAATCAGGGGCACGGAGAACCTCGAGGAAAAGAGGTCGAAGGTCTTTGAGAATAGCACGAAACTTAAAGTAAGGAGAACTTTGAAGAAGATAACCATGGTCACGAGGTCTCTTCTTTGAAGTATAATTAGTATTAATACTGTTATTAGTCTTGTTGTAATTGTTGTTTGAAAGAGAAGAAGGTTGTGGCGGGAGATTAGTTGTTTCTGGAATTTGATTTTGGTGGGTGTTATTTGTTGTTGGGGGTAGTATAGTAGAAGACGCCATTTTCAATCTGTCTTTCTTTCTTCTTCTTCGCCTTTGAATTCGGTGAAATTTTTCTCTCACTTTCTCAAAATTGTTCAAAAGAAAAAGGGTGTTTTGGGTCTTTTGTGATTTCTTTGGGGGGTCTTTAGGAATTCTCTTCTCACTTTCTATAA

*S.maritima*36480

AAACATTTCATATAAGGTGACTTGTATTAGAGTCAGGAGCTTGTGCCAAACATAGCCTTAAATCAGTGAACCCAACAAACAAAAATAAATTGCAGAAATAATAATCACTTCTCCACAAACAGCAAAAATATAACACCCGAAACTCAATTGTAACTTGTATCGGAAAAGGATAATCAAAAAAGCATCTCAAGCTCGTTCCACCTTTTAGTTTTCGAGTACAAGATATCAAAGGGCCTTATTGACCTAAAATCATAAATGTCACATGGCATGATTCCTCGAGCCAAAATTCATAAGCAAAATCGTTTCCTAGAACGTCTCTAATTGTCTACGACCTCAATAAGGGTTGGCTCATAGTCACTAGGAGCCTCGTACCCATGTAGATTCATCACAGTAGCAGCTACATTGGCAAGTCCACCACTATGATCCTTGCGGAATCGAACACCAGGAGCAAGACCTGGACCTCCAATTGCAATAGGTACCGGTTGTAATGTGTGAGATGTGAGAATCTGAACTTTTCCATCTTTAAGAATAGGCTGCCCTTTCTTGTCCCTCTTCACCATGTCTTCAGCATTTCCATGATCAGCAGTAACAACATAAATCCCACCAACTTGCTCAATTGCATCAAGGATCATCTTGACAGCTTCATCAGCAGCCTTACAAGCCACAACTGTGGCTTCTACATCACCAGTATGACCAACCATATCCCCATTAGGGATGTTGACACGAATCTGATGGAATTTGCGGCTCAAAATCATATCCCTTGCCTTTTCACCAATCTCCAAAGCCTTCATTTTAGGTTGCTCATTGAAACTAATGCCACTGTCACTAGGAATTTCCAAATATTCCTCCATTTCAGAGTTGAAGTACCCTGAGCGATTTCCATTCCAGAAAAAGGTAACATGTCCAAACTTGACAGTTTCACTGCAAGCAAATGTCTTAACACCATTGTAAGTAAGATATTCCCCAGACGTTCTCTCAATAAGTGGGGGAGAAACAAGGTAATGACTGGGAAGCTTCAACTCGCCATCATATTGAAGCATGCCAGCATAACGGATTTTTGGCACTCTCACTCTGTCAAACTTATCAAAGTTTTCATACTCAAGTGCTTTTGCAAGCATAACCATACGATCAGCTCGGAAATTGAATGTTACGACAGCATCTCCATCAACAATTGGACCGACAGGCTTTTCATTCTCATCAACAATAACAAAAGGTGGCAAATACTGGTCATTTGCCTTAGTAGTCTCCCTTAAATTCTTGACAGCCTCAATGGCATTCTTGAACTTGTATGGAGCTTCACCAAGCACTTGGGCATCCCATCCTCGTTTCACAACCTCCCAATCATTCTCATAGCGATCCATAGTAACATACATGCGGCCACCTCCAGATGCGATTTGGGCATCAATACCTTTTGCCCGTAAATCCGCCAGATCCTTCTCAAGAGTCTCAACAAAACCTATACTTGAACCATCCAACACATCACGCCCATCGGTAAGAATATGAACACGGATTTTCTTGGCACCACGTTCAGAAGTTCCTTTCAGTAACAACTGTATCTGGTCAAGGCGAGAATGAACACCTCCATCACTCAGCAACCCAATAAGGTGCAATGTGTTTGTTTCAAAACACTCTTTAATATAATTAAAACCTTCATCGTCATAAATTTTTCCAGAGGCAAGCGCAATGTCAACAAGCTTTGCCCCTTGAGCAAAAATACGTCCCGCGCCCAAAGCGTTGTGGCCAACTTCACTGTTACCCATATCATCTTCTGACGGAAGCCCAACAGCTGTACCATGAGCCTTGATCAAGGTCCATTTTTCCGGAGCACCCTCTTTAAGGGAATCCATGGTAGGAGTTTCAGCAACATGAATACAGTTAAATTTATCAGCATTAGCTTCACCCCAACCATCAAGTACCACCATAGCAATGATCTTATCCTTTGGAAGCTTTGGATGGTCTGCTAATTTCCATGAAAATTCTGAACTTCCCATTTTTTCAAATTGAAATGAAAATGAATTTTGAATAATTAGGTTGATGTTTTTTCTCAATTACAATTTTGGTTGGTTGATTGATTGTTGAGATTTATAGAGGTGGCTAGATCTAATTGCGCAGTAAAGCTTTAATATAAAATAATTAGGTAATTTGGGGTTTTGTTTTATTTTATTTTTATTTATGGTTTTCTTCTAGTCAAATCTAGCCAATCAAAGGTATTTTTAAATTAGCGGAAAATAAATATCTTCAAGTCTGTTTAAAAGCAAAAGCTACCGAATATCCCGGGTGTTCCTTATTCATTGAAAGGGCGTTTGGCAATTAGAATTTTAGAGTAAAAATATGATTTTGAAACCAATTTTGGAGCAAAAATGTACGAGTAGTTTAAAACTAACTTTAC

*S.maritima*863845

CAGCAGCACTATGCTCAGCACCAACAGCAACCATTTTCACTTGCTACCTCCCCATCCATGGTGACTGCCAAACAGTGACTGTCAGTCTGTCACCACAAGCGATTTGCTTGATCTTCATACCACCTAATGTCTTGATTGGCTGAGGAATAAGTAGATCGCTAAAGCTTCCATGACCCAATCTCCAAGAATCACCCCTTGTGCCCCACCGGAAAGAATCGATCTTCAGCATCTCCGTGGCTATAGGTTAGGTTGACAGCTTCAGGTTAGTCATGGATGAAGTTGTATCGCCACTATCAAACCACCCTCTTAT

*S.maritima*24751

CATCAACACTATAGTTTCAAAACACATGAACATTTGATAAATAACTGCCCCTACTAGTTACAAGAATATTAAATAATATGGAGGAGCATTAATCAACAAATATGGTATACATGGTATACAATAGGAAGTTGATATTGGCTGATTGATATTGACACTCCTTTTTTGCACGAGAAGCATTGAAAACTATACTTTTATAAGAAGAGTGTCAAATTCAATTTCTAGGAGTGTCAATATCAGTTTTCTTGTGCTTTGGCACTTGACAATGTTCGCAAGTCCAGGCAACATACTCGGTGACAATGAGTTGCACATGTTTAGATGGTTAGGGTATTTCTAATCTAAGAGATATACAATGTATACACATGTCAGGATACATTCGCAATGTCAATGAACAAATTGATCTATTCACAAGCTGAAACCAATTCTATGCAGCTTCAAGCTTACGACTCGTGCGATCTCTCACGGCTCCTGAATTCTGACTACAGCGGTCTTCAGTCGTACTTTTAAGCGTCAAATCTTCCATGGCAGCACAAAGAAAATCTGGGCTGGTTGAAGCCTTGGCATCCTCAAGTTTGGATGTTGAAACCACAAGATTTTCATCTTTTTCATCTCTAATTTGTGATTCAAGCTTATTTAGTTCATCCTCAATATCTTCATCCTCTACTGCTGCATACGATGTAGATTCTAAGGCATTGTGGATTTGTTGTTGTGAATCCATGAACTCATCAAGTTCTTGCAAACACGAGTGAACATCTTCGACTTCCGTTTTGTTTTCCTTGATAGCTTGAGCACCAAGCTGGATAGCTTCAGACACCTTCTTTGCAGATTCTGTGTCTGCAATAGCACGGAGAACTTCCTCAACTCGGTTACAGAATGATGCGACTTTTTCTCTACTCTCTGAAGCTAACTTCAACTGCCTTGCTTTCCTCAAAGCTAATTTCTTGTCTCCAGATTTCAAAGCAGCTAAAGCAGATGTTCTCAATCTTTTGTCTTGCTCTTCAATAACAGAAAGCTGTAGCTGAAGCTCTTCTAGAGTCCAAACCAATTGCAAAACCACAGAATCTACACTTGGAGTACTGGAACAAGGAGCAGAAGATAAAGATACTTTTACACCCTCTATCAGTTCTTTACTTTTTACCGAAAGGTATCTTGCTTTCCCTTGTCTTGACAAATAACTTAGCACGGCATGTGCTTCACTATGCTCCTTGAACTCACTTTCAAATCTCTTCATTGTTATGATGTATGAAGATGTCCAGTGGGTTTCTGATAATGCATTGATGATACTGGCAGCTTTATCCTCTAGCATCGGGGAAAGAATGAGACGATCATTGAGAACATCACTTAAATTTGTTGATCTAAAGGCACTAGGGAAGAACATTAATTTCTTGAGAAGCTGAGAAAGTTGTCCACTTTTTGGATCTCCAAGATCAGTCATCCGCAGAATTTGACCAGTTTTGTACATTTCAAGCAGAACATCATCAAGGCATAAAGGAGACAACCCACCTTTATTAAACCAGCAATTTTTCATCTGAGAACGAGAAAGAAAAAGAAGGTTAAATCGTTGAGCAATTTTGATAATCAAATCCCTCCAAAATTGAAATCGAGGTTCCCAATCAGACCTTTGGCCACTAAAAGCTTTAAATCGAGCAGTAGATATGATATCATCATCCCAATCTTGAACTTGCTCTCTTATGAATTTTACAACTTCCAAATCTTCAACCATTTTTACATGCATAAAAACAAGAAAGATATTTGAATGGGCTAGTTGCACACTAGCACCTTGCAGGCTTC

*S.maritima*38414

GGAATGGAGTATGATACAATAGAGATGAGAATGACTGTAAACTGTAAAGTGGTTGTACTACTTTGTCCTATAAAATTGTGATAAGAGATGGCAATGGAGTGGATATAAGCTGACATCCACATCCACTCGACCACCATGCACATATTCACTGATTCCACCCACTACCCATGTGGATATCCACTCGACCTAGTTCAAAATCTCACTAGGAGACTGTAGTAGTGTATAATCTCATTGGGAGCTGGTGTCATTATATATAGACACATAGTAGCAAAAACCTCAAAGCCTTACTCAAAATAAATTTACAGTAGGTTATGTATGTATCAATGACCTGATTCAGCAAGGCATGCCAATGCAGAGGTTTGTATTGGCAATACACAAACTGATCTGAAGATGCAGTCCTAGTCGACCCAGTTCTACCATCAGACTCCACGAACTGGGCAGTTCGATGAGATACTCCAAACTGAAGCATTTGCACGTCAAGTTTATCTAAACCTCTCTTGTACACCACAAAAGGAGCATTGTTGGTGGCTTGGTGCATCTGCGCCTCAAGGAACCTTGTTATGTCTTTTGCCCAAGTAAATGTCTGAATTCAGAAGATTAACAAGAATGACTACGAAGTAATCATCTCACTTTGGCAATTTTTCAAGTGATTAAACCAAGATTGACCGTCTCTCCTTATAGAATCATGTGTCGTACAAAATAAAATCATCCTCCTGATCAAATACATCAACACCAGAGGCAAACAAATCCAAGACATTGTAGATGTGATCAGTATCCGAATGAGAATTGTCTCCAGAAACAAATGCTGAAACCTCATCCTTTATCTTAATCCATGATGATCCTGCCTCTTTTACTAGCCCTCTCGATCTCATCAGCTTTCTAATATCACCTGCCTCTCTCCACTTCCCGTTTGCAGAGTAAGTGTTAGCTAAGGTAATAAGTGTTCCCCTACAATTAGGGTTGTTATTAAGAATCATTTCAGCAGCCCGTGTAGCACGCTCAACATCACCATGAATTCTACACGCCTGCAGTAGCGTTGACCAGATAACATCATCTACTTCAAGTCGCATCCCATTGATCATGTTCTCAGCTTCAACTAGCTTTCCAGCTCTGCATAATAGGTCAATCATGCAGCCATAGTGTTCTTTTGTGGGACTAATCTCAAACTTTTGACTCATCGAATTAAAATACTGATAACCCAAATCAACAAGTCCTGCATGACTACAAGCAGAAAGAACTCCAACAAATGTCACAGCATCTGGCCGCAAACCAGTTTGTAGCATTTTCTCAAACAAATCTATGGCTTCTAGACTGTCACCATGTTCAGCTAATCCGTTTACCATGGAAGTCCATGACACGACATCATCATTTCTTGTAGCATTGTATACCTCTGAAGCATCCTTTACGCTTCCACATTTGGAATACATAGTGATAAGTGCACTTTGTATCAAAGACATATGTTCTAATCCAACTTTCAAAATATCAGCATGTAGTTGTCTACCCTGTTCCAGAATTGCCATACTTCCAGAGACACTCAACATACTAGCAAAAGCAAATTCGGTAGGTTTTGGCCCTTCCCTCCTCATCCATAAGAAATACCTAAACGCTTCATTGCCATATCCAGCTTGAGAAAATCCGGCTATTATGCTGCTCCATGAGACAATATCTTTCCTAGTCATAGACTCAAAAACTGAGGATGCAGATTTAAATTGTCCACATCTTGAGTATAGTGTCATGACTGAATTTGCGACAGATAGAGCATACACGGAACCAACTTTCGTTACATGAGCATGTAGTTGTTCACCCCATCTGATTTTACCAAGATGGGCACAAGCAGTGATAACTGCTGCAAATGTGTACTCATTAGGTCTCACCTCAGAGTTCCGCAAGCGCAAGAACTGTTGAACAGCAATCTCTTCTTCACCCATCTGAACATATGAAGTAATGAGCGAGGTCCATCCAACCACATCAGGAATCTTTTTTCTTCCAAACAAACACATCCCATAGTCCAATTTCCTACATTTATTGTACATGGTAGCAAGGGTGTTCACAACGTAAGAGCTCAAATCAAAACCCTTCTTTATTGCTTGGGTATGTATTTCCTTTCCATGTTCAAGATCACATAAATCAGCACAGGCCTTCACAGCAATGGCAAAACTATATGCATCACAGTGCAAACCAGAGCACCACATATCAGAAAAATACAGCAACCCTTCCTTACAGTACCCAGCCTTAACCAATCCTGTTATGATGGCGGTCCACGAAACGACATTCTTCTCCGGCATCTCGTCGAAAACCTGACAACTCTCCGAAATATTGCCAGATTTTGCATACATGTCCAATAGTGAACTTCCCACAAACACAGAATCAACAAGACCATTCTTTACACTAACCCCATGCAATGATTTTCCATAAGTAAGACTATTATTTAGTCCACAAGCTTTCAGAGCTAAACTAAGCACAAACGGGTCAATTCTAACACAAGATTCAACCCACATTGTTGAAAACAAATGCAAAGCTTCGTCAAAGTTTGAATTTTTGACATAACCAGATATCACATTGGTCCAAGAAACAACATCTCTTTGTGGCATTTCATCGAACACCTTACGGGCATCCTTCAAATGACCATCACTAACAAGCTGTTTAATCTGGGAATTTATTTCCAACAAATTTGGAACTTGAGAATCAAGAAAATCAGCAGAAATGGCTGATGATCGAGCAAAGTATCTCCTGACATGGGGTTTTGTGAAGAGAATCATAACATCAATAAGCACCCAATAAACAAAAAATACTTAGTTTATTAGAGATAAATTGTTCATTT

*S.maritima*37533

AGAAAAAGTTTCCCTCATACCCTTTCTCCCTCCTCTTTTTCTCTCTCTCCACTTCCAAAACCCTTGTTTCTCTCTCTTCATCTCTGGTCAAAGACTTTGTTGATTTCAAGAACAAAAAACGATGTCGTTGAGGCCAAATGCTAGAACCGAAGTTCGCCGTAACCGCTACAAAGTATCGGTTGACGCCGATGAAGGTCGTCGGAGAAGAGAAGATAATATGGTGGAGATCCGAAAGAGTAAGAGAGAAGAAAGCTTGCTTAAGAAGCGTCGCGAAGGTTTTCAATCTCAGCAACAATTTTCTACTACTACTCTTCATGCTTCTACTGTTGACAAGAAGTTAGAGAGTCTTCCTACCATGGTAGCAGGGGTTTGTTCCGACAATAATCAAATGCAATTGGAAGCCACTACGCAATTCCGTAAATTGCTTTCAATAGAGAGAAGCCCACCAATTGAAGAAGTCATACAAGCTGGAGTTGTTCCCCGCTTTGTGGAGTTTCTTCAGAGGGAGGATTTTCCACAGCTTCAGTTTGAAGCTGCATGGGCTCTCACAAACATTGCGTCAGGGACTTCAGAAAACACCAAGGTGGTAATTGATCATGGAGCTGTTCCAATTTTTGTGAAGCTTCTTGCTTCTCCCAGTGATGATGTCCGTGAGCAGGCAGTCTGGGCATTGGGTAATGTTGCTGGTGACTCTCCTAGATGCCGGGATCTGGTTCTTAGCAGTGGTGCACTAATTCCACTGCTTGCCCAACTAAATGAGCATTCCAAGCTGTCCATGCTTAGAAATGCTACGTGGACTTTGTCCAATTTTTGTAGGGGAAAGCCACAACCACCCTTTGAGCAGGTGAAACCAGCTCTTCCTGCTCTTGAACGTCTTGTTCATTCAAATGATGAAGAAGTGTTGACAGATGCATGCTGGGCACTCTCTTACCTATCTGATGGACCAAATGACAAAATTCAAGCTGTGATTGAGGCAGGTGTATGCCCACGATTGGTTGAGCTCTTGATGCACCCAGCTCCATCAGTACTGATTCCTGCCCTTCGCACAGTTGGCAATATTGTCACTGGAGATGATGTCCAGACACAGTTTATTATCACTAGTGGTGCGTTGCCATGCCTTCTGAACCTGTTGACCCAGAATCACAAAAAGAGCATTAAAAAAGAAGCTTGTTGGACAATATCTAACATTACGGCTGGAAACAAGGCTCAGATTCAGGCTGTTATCGATGCTGGTTTGGTCGGCCCTTTAGTCAACTTGCTACAAACTGCAGAATTTGACATAAAAAAAGAAGCTGCTTGGGCAATATCTAATGCTACATCTGGTGGAGCTCCAGAACAAATAAAGTACCTTGTGAGTCAGGGGTGCATAAAGCCATTGTGTGATCTACTTGTATGTCCTGATCCAAGGATCATCACTGTCTGTCTAGAAGGGCTGGAGAACATTCTAAAAGTTGGTGAAGCAGAGAAGAATTTGGGCAACACTGGAGATGTTAATTATTATGCACAGTTGATCGATGATGCTGAGGGATTGGAAAAGATTGAAAATCTACAAAGTCATGATAACAATGAAATTTATGAAAAAGCAGTGAAAATTCTCGAGACATATTGGTTGGAGGAAGAGGAAGAGACTGCACCATCTGGTGATGCTGCTGAGGCTGGGGTCCCAATGTGGAACTCTGAAGTTCCTGCTGGCGGTTTTAAGTTTGGCTGAAAGGATTTTGGTGTTTGGATGCATGAAGTCGCAATACATTGAAGTTGACTATATGAATTTGGGCTCTAGCTTGATATCAGGTGGACTTTGCAGTCCAACTCTGCTCGGGCTGGGTTTGGTGATGACATCTTGCATGTGTCAAAAGTCCGGGTCGATGTCTGTGGTTAAGGTGCTGCCAGTGGGTCTGTCGGTAGTCACTGGTCTAGTAAAGACCAGATGGTCATTTTAAGGTGATATTGGTTAATGTGGTGCTTTTATCCTTAGTTGGGGGGGTGTTTGTGTCTATTAGTGGACAATTCATTGGTTTTGGGTTGATTTTGTATTTTTGGTTTTTTTGTTTTAATTGCGGGTGGATAGGTTGTTTTTTTAAGAGTGAAGGTGTTGTAGATGCCATTGTTGGCCCGTTGCATGTTCATTAGCGCCATCCTACAGTCGTAGATATTTGTTTGTCTGGCAGTTTATCCTTGACATATATAACCATTGTTTTTGTTTTGCCCAAAATACATCTAGTGAAATTGCTGATAACCAGTGTTTGTGTTTACACACCTACACCTATGTTTAGGGATAAAATGCATTGGAGGAGTGTGTTATGGTGCCAAGAGATGTCCGGGACCTAATGAAATGTCAACTTTTTTCGCAC

*S.maritima*1267768

GTTGAGTTGATATCTTCACTACCTTATTTTTGCAGTGAGACATCAGTAGATGGCTGCCTTTCTAAACTTCTCCGTGCACTTTTCTAGCATTTTAGTCTTCCTCCTCATCCTCGCTACATTGGTTACCTCCCATGGAAGCAACAACAAATATAAGCACTGCTCTACCATGATATCATGTGGGAAGCTTCAGAATGTTGGGTATCCTTTTTGGGGGCTTGGGAGGCCCAAATATTGTGGTCACCCAGCTTTGCAGCTCCATTGTCCTCATAACAAAAGCAACGAATATCCTGTCTTAAAAGGAGGTGTTGAATATTACTACGTACAAGGCATCAACAACA

*S.maritima*25131

CAAAAACTTTCAAATACAACTCCAAAATCCATTCACTTCAAATTTTTACCCAAAATCAAAATCAAAATTCTAATACTAATTATTATCCTGTCTTTTACTAATGCACTTCAAATTTATGAATTGTTGGTATGATACCATGATCCCTCCTTTTCAGACTGCTAATTTGATATGCACACCAACTGTTTGATCTTTGTTCTGTGAGAACATCACTTCAGAAATTCTTGTTGTTTGTAGTTTGTTTAGTAATATCTTCTTAAACTTAATTTGGGAGTTTTCTTAAGTTCAATGTTTAGGAAAACCTTGTTCAAAGTTCATAATTTGCTTACTTCCGTAACCCATCAAGCTCGTGGGCTTTCGCAGAAGCCTGTTCCTAATCAAGTTTCAAAGTACTTTCGTCAAGCAAAACTCATAGATTCAATCCGCCTTGCTCTTAGGCATGAATCTCCTGATGCTATTCTTCCATTGTTGGATGACACTAATTTGGACTCATTTGTAGTGACGAATGCTCTTCGTTGCGCTCCTTCCCCTGAGTCTGCTCTGTCCTTTGTTGAAGCCCTTAAAGGGGTGCCTAATTTTTGTCATACCCAACAAACTCTTCAAGCCATTGCTAAAATCTTAGCTAGATTAGGCCATACTCGTCAGCTGGAAAAGCTCATTGCTTCCATCAATGCTGGTGAATTCCCTAAGGTTCGGCCTATTAGTCTTGTGGATCAAATGCGGTGGTATGCTGCTGCTCAAGACATGGACTTGGTACTACATGTTTGGGAACAACTTCGTGCCCAAGGTACGCAAAACCCAAATGTTGAATCGTACAATATAGTTATGGGCCTTTATGTTTTGATGGGAAAAGATATGGAAGCTGTACAGACGTTTCGAAGTATAATGCAAGAAGGAGCTATTCCTAACTCGCGAACTTTTACTATTGTTATTGAGCACCTTGTGAATTCGGGGAAAGTAGATAATGCAAAAGAGGTATTTTATATGTTGCCACGGATGAGGCTTAAGCATACTCTTAGACAGTATTCACTTCTTATGGAGTCATTCTTGGAAACTCAGCAGTTTGAGGCTATGAAAAGCCTTGTTGGAGAAATGAAGATGCAAGGAGTATTACCTCCAAGATCAATGCTATTGCCATTGCAACGAATGTGTGAGGCAGGGTTTTTGGATGAGAGTGATGAGTTCATTAAAGAGATGATACCTGATAATAGAATTACAAACATAAAGTTATGTGTAGATGTCAAAAATGGCGATGATCAGGAAGATGATGATGAAGAAGAAGATTGCAGTAGTGAAGCTGACACTGGTAGTATTCAGTTGAAACCATGGTTGGATCCAAGTGCTTTAGCAAAAGCATTGGAGCATTGGAGAGATGTAGACATATGTGCTTTAGAAGATGCTAAACTTGTGTGGACTAGCCGCTTGGTTTGCAAGATGGTTAAGCAAATTAAATCACCTCAAACAGCATGGAAGTTCTTCTTGTGGGTTGCTAATCAACCTGGATTTGTGCATGATGTTCATACTTATTCAAGGATGATTACAAAAGCAGCAAGGTCCGGGAGGGTTGATCTGGCTGATGATCTATTGCTCAAACTGAAGAGTGAAGGAATTAAGTTGACCATCAGCACAGTCAGACAAATTATTGATTTTTATGGACTATTCAAGGAAGGAGATGCCGCCCAGAGGGTTTTACACGATGCTAAAGTTTTATGTGGCCATCTCTCTGATTTCGATACCATGCTTTTATATTCTTCACTTTTACGAACACTGGTCAAGTGTGGGAGAGATCATGATACCATAGATACTGTCAACAAGATGTTCTCTTTGGGAATCTACCCTGATGCTCAAACATTTTCTGGTTTGATGCATTACTTTGCAGTTCGGGGTGATTTTAGAACAGTGCAGACACTATTTCGGATGGTTAAGCAATGTGATATAGAGCCAGATGCTTACATGTATAAAACACTAGTCCGTGCTTACTGTAAACATGGAAGGGCTACTCTTGCTCTGAGGCTATTTGAAGACATGAGAAACACTGGGTTGGTTCCTGATGTTGAGACAAAATCATTGCTTGTGAAGAGTCTTTGGAAAGAAGGGAGGCTTAGAGATGCTTCCCTTGTTGAAGTGACTTGTGAAGAACACGATGTCGTCCTTCCGCTTCCTTCAACTAGTCAGATGTTCAATGTCAGCTCTGCAGATCTTAGAGCCATACACGACTTATACTGTGGCAGTTTTTAAGCCAATATTGGTTGATCGATCCTGTTGCGTGAGAAATTGAAAAATCATGCAACAGAGTGCCGTTTATTTATTTGCAGGTAACCTGTCGAATACTTGTTCCCTTTTGAAACTCATGTTATTACTTATTAGTTGATTGCATTTCACTTCAATATCTGAACTATGACTGCCTCATTTTTATTTCAGAATCATACTAGTAATTTATGGCAGTTTCAATATAAGAAACTGATCTTCTAAAGCACTGTAGTACAAAATAACATTATTTGTACACAAATTTCATGTCTTTATCCTAAAAGAAATAAATCAAATGTGAGACCAGTAATAT

*S.maritima*1936037

CAGCGATGTAAGATTTCCAACCTCAGGTGGGATACTCCCAGTGAGATTGTTCGAGAACAGCTGTAATGTCGATAGGTTGGTCAAGTTCCCAATGGTGGGAGGAATAGGTCCAGAGAGTAAGTTTGACGAAAGATCAAGCTGGATAAGATCATGCAGTTTTCCTATCTCTGGCGGTATTGTGCCAGTAAATCTGTTGTTGTACAGAAAAATGATCTGGACTTTTGAGAGTGCACCTATTTCTGGTGGAATAATTCCGCTGAAATTATTGTTCTGAAATTGCAGGGAGATCAATTCAGTCCAATTGCTGAAAAAATCAGAAGCGATTTTACCAGATAGCTTATTTTCGGATAAACCAAATTCTGATAATTTAGTAAGACTGCTCAAGGACAGTGGCAACTCCCCAGTGAGCGAATTATCAGCAGCAGCAAAATAAGTAAGGTTGGTACATAAGCCGAGCTCAGAAGGGATAGTAGAGTT

*S.maritima*19691

CTTGAGTTCCTACAACTTTTTTTCCCTCCTTATATAAACAAAACTTTAACCTCTTATTTAAGCAAAATTCTCATGCATATATCCCCAGAATTTTACTTCACTTCGCTTTTTTTGAATTCAAGGGATTATCCAAAACAGTGAGTGTTAATTACACATTAATATTACAAGGATAACCTGAGAAGCTAAGTTCCCTTTGATTATTCTTAAGGGTACGTACCCATTTTCCCTTGAGCTAAGAAACAACGTACGAAAATCGTTTGGTAAAAGATTTCTTGATCAAGCATCTCAAAATAGACGCAATCGTTGTTGTTGTTGGGAAGTTTTTAGCCTAGTATGGGTGGGTGTGTTTCAACTACACACTCTAGAGTTGCTCCACACAGGAAGAAGTACACGCGTAGGCTAAGAAACCGTCACAAGAAGATGAACTCAACCATGGGCGATCCTCCTATCAAAAGACGATCACATAGTGATGCAGGAAGCCGCATCTCTGTGAGTGAATTTGTTCAAATTGACTTTGAGAACGGAGCCACTACTACTTGCCGGAGATCTGAGGTTTCTAACAAATCGTTTCATCTAACCCAGGTGCATTGGAACCATGTCGATGAAAACGGAGTATGTCAGGAAGAAGCGTGGTATGATTCAATGAGCATATTAGAATCAGAATCTGATGACGATTTTATCAGTGTTTTCGGAGATGGGAAAATACTCAACATGTCAAACCCATGGTCAGGTGCACAGTTTTTGCGTCCCCAAGCAGGACTGGTGGTTCCAGGTGCAAATGGTGAGAAGGCATCACCAGGCTCTTGGTCTGCCATTTCACCCTCAATTTTCAGGGTCCGAGGCGAGAGCTACTTTAAAGATAAACAGAAACATGAGGCACCAGGATATTGTCCGTATACTCCCGTTGGTGCTGATATGTTTGCTTGCTCACAGAAGATTAGTCATATTGCTCAGCACCTTGAGCTTCCCTCAGTCCAACCAAACAGTAAATTACCGCCACTACTGGTTGTTAATATACAGATGCCTACGTATGCTCCCTCGATGTTCCTAGGTGAAAGCAATGGCGAAGGCATAAGCCTTGTGCTGTACTTCAAGCTATCTGAAAACGTTGATGAAGAAATTCCAGCCGAATTCATAGAGAGCATCAAGAGACTGGTGGACAATGAGACGGAAAAACTTAAGTCAGTAGTTCCATACCGAGAAAGACTTAAAATCTTGTCAGGCGTAGTAAATCCCGAGGATCTTCAGTTGAGTTCTACTGAAAAGAGACTTCTGAATGCTTACAAGGATAAACCTGTGCTAACACGTCCTCAGCATGAATTCTTCAAGGGACCCGACTATTTTGAAATCGACATAGATGTGCACAGGTTCAGCTTTGTATCGAGGAAAGCTTTGGAATCTCTAAGAGAACGGTTAAAAGCAGGAATTCTTGATCTCGGTTTGACTATTCAGGCTCAAAAGGTGGAGGAACTACCAGAAAAGGTGTTGTGTTGCATGAGATTGAACAAACTTGACTTTGTAAATCATGGCCAAATACCCACACTAATGACCCTCAACGATGATTGAATCGAGCAACCTTGTTGGGGAAGCCCTTCCAGAAATCGTGGAAACGAGGACAGTTGATTGACACATTCTTTGTATAGTTTGGTTATATGTATTGTTCATTGTAATTTTTTAGATTCAAGAGAGAAGGGTTATAAAAAGAAAAAAAATATATATATATATATACACACAC

*S.maritima*45062

ATACTTTAAAAAAATAAAGGAAAAAGAAAAATCCCCAGTTAACCTCTTTCTCACACAACAATTAAAATTTTTCTCTCTCCTCGTCTTTGTTCATTCCCCGTGTTCGTTGTTGTCGTTGATTCGAATCACCATAACCAAAATTTTCTTACATCTTCGTCATCAATTTTCAATCGTCATCATCAACTTATACATTTTTCTGCACTTTCGAAATCCCTAATTTATAACTCTCGAATTTTCGATTCCTTCGTATCAATCTACACTTCATATCTCTCTCTTTTCTTAAATTTTTGGGGAAAAAAACCCTAATATTGCGTTGTTAAGTTTCACTTGATCTTGAAGTTCGGAAACCCTAATTTCGACTTCAATTCTATTTTTAGGGGTTTTAATTTGATTTTGGTGTTCGTGATTATTGATTTGGGGAATTAGGGTTTAGGATTTGAAGTAGGTCTGATTTATTGATTCAGAAATTAAATAGTGGGTAAATGTTTGAGGCAGTATGCTGCTGATAGTTTGGAGAAATGCAAAGGTATCATGCTGCTAGCTGCACTAGTGCTGTCAATAACAACACTCCTCTTGGGGGCATTTCCAATAGAGATTCTACTGCTCGAGGCGGCGGAGGTGGCGGAGATTCGTCTTCTTTGTCTGCCAATTTCTCTTTAAATTCACGACGGGGTTTGCCGCTTGCTCCATATAGGCTGAAGTGCGAGAAGGAGCCACTCAATTCTCGCCTCGGGCCGCCTGACTTTCATCCGCAAACTTTAAATTGTCCAGAGGAGACACTCACCAAGGAATATGTGCAGTCTGGATACAGAGAAACTGTCGAGGGACTTGAGGAAGCGAAAGAGATCTCAGTTACACAAGTTAGTAATTTTACAAAGCCTATTGTTGGCAAATGCAAGGAGGCGATTAGAAAACGTTTAAGGGCCATCAATGAGTCCCGTGCACAGAAGCGCAAGGCTGGCCAAGTTTATGGGGAGCCACTGTCTGCTTCTTTGTTGAGTAAACCTGGTGTCTTCCCAGAGCTGAAGGCTTGTGGTGAAGACTTCAAGAGGAGGTGGATTGAGGGTTTATCTCAATACCACAAAAGCCTTCGGTCTTTGGCTGATCATGTTCCTCATGGCTATAGAAAGAAGTCTTTATTTGAGGTTCTTATCAGAAATAATGTCCCGTTGCTGAGAGCAACTTGGTTCATCAAAGTAACTTATCTTAATCAGATTCGACCTGGATCATCCAGTGTGTCCCCTGACCGCATTCAGTTGTCACGTACAGAGATTTGGACAAAAGATGTCACTGATTACTTGCAGTTTCTTCTGGATGAAATTTTTCCAAAGAATACTTCTCATTCCATTTCTCAAAATAGAGATAGATCACAACAAGCGTTATATGCTGGGTCGGTGCAACAAAAGTCTGATCCGGCATCACTACCCTTAGATTCTGAGGAGCCGTCACTGCATTTTAAGTGGTGGTATGTTGTCCGTATTCTTAATTGGCACCATGCAGAAGGGCTTATTTTTCCTTCTCAAGTTATCGATTGGGTGTTTCTGCAGTTTCAGGATAAAGATTTATTTAAGGTCATGCAGTTCCTCTTGCCAATCGTATATGGATTACTGGAAACCATTACTTCATGTCAGACTTATGTCAGAAAGCTAGTGGATATCACCCTGCGTTTTGTTAAGGAACCTTGCTCTGGCAGTTCTGATCGATTTGATAATTCTCGGAGAGCATATACCTATTCTTCTCTTGTTGAGATGCTTCAATATCTCATATTGTCTGTACCAGATGCCTTTGTTGGTCTGGATTGCTTTCCATTGCCCCAATGTTTGATGAGATCTGTTGAAAATGAGGGGTCCCTTCAGTCTAAGTTCCCGGTAGATGGTGAGCACTTGATTAATAGTCCTGCTGGTGAAAAAGCTCTTGACATCAAAAACCCGTCATCATGGTTTAGGCGTGTAGTTTCTTCGATTCAGAAACGAGCAACTTATCTGGCAAAGGCTGCTAGCCCAGGCTATTCTGGGCAGAATGTTGCTAAAGCTGTGCAGGCCTTAGATAGAACTCTTGTGCAAGGAGATATCCAAGAGGCATACAATTTCCTCTTTGAAGGTCTCTCTGATTCTTCCGTTCATGATAGCTGGTTCACAGAAGTAAGCCCTTGCTTACGAGCATCTTTGAAATGGATAAGTTATGTTAGTCCTCCATTACTTTATTCCGTGTTTCTTCTTTGCGAATGGTGTACCTGTGATTTTAGGGACTTTAGAACTGCACCCGTCCACGATGTTAAATTCACTGGGAGGAGAGATCTGTCACAAATTTATGTCGCAGTTAGGCTCTTGAAGCAGAAAGCAACGGAAATAAGAAAATCAGAGAAAGGAAAGAAAGGTGCTCAAAGAAGGGAAAATGGAAGTTTCGATGACAATAAGTTTGATTGGAGAAAACTAGAAAAGAGCTGCATTGAAACATCAGATTTTTTTGAGAGTCCTGGTCCCTTGCATGATGTCATAGTATGCTGGATGGATCAACATGAAACACAAAAAGGCGAGGGTTCTAAGCGTGTACAGTTGCTCCTCATTGAGCTGACACGTTCTGATATATTTTCTCCGTTAGCATATGCTAGACAGCTTTTAGTAAGTGGAATTATGGACAAAATTGGGCCTGTTGTTGATTCATCCAGACAGAAGAGACATTATCGGATCTTGAAACAGCTTTCAGCTTCCTGTATGCAAGACATCTTTGATGAAGCTCAGAATGCTGACAGGTCACTTCTTTCTGAAGCAATACAAATCTACTCAAATGAAAGGCGCCTTCTTATTCGTGGGCTGTTAAGTGATGAGTTAAAGCAAACTAGCTCTGCGAATATTGCTTCCAAAAAGCAGAAAGATCATTCAGTCTCGCAGAAGGATCATTCTTTCCCCCCTTCTTTATCAGACGTGTCTCATAGGAAAACCCTGAAGAATGACCCGGACCTTGAGAAACTGAAACTGTCTATTTTAGAAATATTGCTTCTTCCGAAGTTTTCTGCTGATGCTGGAGCTGATGAATCTCAATTAGGCCTAAAAAGGTCCGTCGGACTCATTTCCAGTAAGCTAGACATGTCAGAAGGAACACCTGGTTGTGAAGAATGTCGAAAGGCAAAGAGACAAAAATTAGGTGAGGAAAAGTATTCTTTTCTTCAGGGTGCCTCGTCCAACTCTTTAGATGATCAAGATAATTGGTGGGCGAAAAAGGAGCCAAAACCTTTTGATTCCTTCAAAGTTGATCAACCAGTTAAGCCAAGCAAGCAGGCCTCGAGAGGCAGACAGAAGTCTGGACGTAAAACGCAAAGCCTAAATCAACTAGCTGCAGCAAGAATTGAGGGAAGTCAGGGAGCCTCTACAAGCCATATATGTGATAATAAAGTTGGATGTCCTCATCACCGACCAGTTGCAGAAGGAGATGCTGCAAGATCTGCTGATGTGAACAGACCAATTAGTGGCACTGATATTGTTTCAATTGGTAAGATTTTAAATCGACTGAAGTTTGCTGAAAAGAGGAGCATCTCTGCTTGGGTAGTTAGTTCTGTGAAGCAAGTTGTTGAAGAAACCGAAAGGACGTCTACCAAAAGTGGTCAGCTTAATAGATCTGTATCTTCTGTGGATGAAAAAAGCTCACCTCGATGGAAGCTTGGTGAGGATGAGTTGTCTGTCATACTTTATTTGATGGACATGTGCTCTGAAGTACCATTGGCAATTAGATTCCTCATTTGGCTGTTGCCAAAGTCTGTTGGTAATCTCAGTACTCCTACACATGGTGGTAGGAATGTAATGATGCTGCCTAGGAATGTAGAAAGCCAGGCCTGTGAAGTGAAGGAGCCCTTTATCCTATCAGCTATTCGAAGGTACGAGAACATAATTGTTGCGGTGGATCTTGTTCCAGAAATTTTATCGAGTGCCATGAATCGTGCTGCTGCAGTTTTGGCCTCTAACACCAGACTTTCAGGTTCTCCAACCTTTTTGTATGCTAAGTATTTGCTGAAGAAATATAGCAATGTTAGCAGTGTAATAGAGTGGGAGAAAAGTTTTAAGGCAACATGTGATAAAAGGCTGCTTTCTGAACTTGAATCTGGAAAATTGCAAAATGGAGAATTTGGTTTTATTCCTGCTGGTGTTGAAGATCTTGATGATTTTATTCGCCAAAAAATTAGTGGTAATCGTTTGTCTAGAACTGGTATGAATATGAGGGAAATTGTGCAGAGATATGTTGATGAGGCAGTTCATCATCTTTTTGGCAAAGAGAGAAAGCTTTTCGGCCCTGGTGCTCATAAGAATGCTGGCATCGATAAATGGGATGACGGTTATCAAATTGCCCAGCAAATTATTATGGGGCTAATGGACTGCTTTAGGCAGACAGGTGGTGCTGCTCAAGAAGGGGATCCTTCTTTGGTGTCCTCTGCCGTTTCCGCAATTGTGGGCAATGTTGGGCCTGCTATAGCAAAAATGCCTGATTTCACCGCAGTCAGTAACCATTCTAACTTTCCAGCTCCAACAGGTTCGTTGAGTTTTGCTCGTCGAGTCTTGCGAATTCATATAAATTGTTTGTGCCTACTCAAGGAAGCCCTTGGGGAGAAGCATAGTCGTGCATTTGAGATAGCTCTTGCTACTGAAGCTTCTTCGGCTCTTGCTGGAGTCCTTTTGCCAGGCAAGGGTGCGCGAGCACAATTTCAGTTGTCTCCTGATGCTCATGATTCTGGAATGAACATGTCAAATGATGTACCCAATAATTCCACGAAAGTTGCTGTTGGTAGGGCAACAAAGATTGCTGCAGCTGTGTCAGCCCTTGTCATCGGGGCCATTATCCATGGGGTCTCTAGCTTGGAGAGAATGGTAACAGTCTTTAGACTTAGAGAAGGTTTGGATTTGGTGCAGTTTGTTAGGAGTTCTAGATCTCACGCTAATGGAAATGCTAGATCATCTGGGGCCCTCAAGGTAGAAAATGCATTTGAAGTTTATATACACTGGTTTAGGCTGCTTGTTGGAAATTGTAGAACAGTCTCTGATGGTGTTATTGTGGATCTTCTTGGGGAACCCTCTTTGATGGCTCTTTCAAGGATGCAGCGCACACTTCCTCTTAATTTGGTGTTACCTCCAGCTTATTCATTGTTTGCATTCATGATCTGGAGGCCGTTTATCTTCAGTAGCAGCACAGGAAGCCGTGAAGAGATCCCTCAGTTGCTTCAGTCTTTGACCATGGCTATAGGCGACGCAATAAAACATGCGCCATTTCGAGATATATGTCTGAGAAATACTCATGGGTTGTTTGATCTTGTGACTTCTGATACTACTGATGCTGAATTTGCATCCATTTTAGAGTTGAATGGTTCTGATAGAAATATTAAGGCGAAGGCCTTCATCCCTCTGCGTGCTAGGCTTTTTCTGAATGCATTGATCGACTGTAAATTGCCACATTCTGCGCCTTTCCAGGATGATGGTAATCGATTATCTGGGACCAATGATTTAAAGGCTCAACATGGAGAAAGTGAAACAAAGATTTCAGATAGGCTTGTACGTGCATTGGATACCTTGCAGCCTGCTAAATTTCATTGGCAATGGGTTGAGCTTAGGCTTTTCCTTAGTGAGCTGACCCTTATCGGTAAAATTGATTCCAACGATACACAATCAGTAGCTGAGGCTATCCGTTCTGTTTCACCCAACCCTGATAAGGGTGGTCCTTTAGAGAGTGAGAACAACTTCATTCCAATTGTCCTTACAAGGTTATTGGTGAGGCCAGATGCTGCTCCCCTTCTCTCAGAAGTAGTACATCTTTTTGGAAGGTCTTTGGAGGATTCAATGTTGTGGCATGTTAAATGGTTTCTAGAAGGACATGATGTGCTTTTTGGAAAAAAATCTATTAGGCAGCGACTTGTGAATTTTGCTGAAAGTAAAAGCTTTTCAACTAAGGCCCAGTTTTGGAAACCATGGGGTTGGTGCAATTATATTGATTCTTCTGGAAACAGGGGAGAGAAAAGAAGATTTGAGACTGTTTCTGTTGAAGAAGGTGAGTTGGTTGAAGAAGACAATAAAAAACATGCTAGAGGTGCCTCAAAGATGTCTGATCGTGAAGGTCATAACACTAACCAACAGTTTGTGACAGAGAAAGCACTTATTGAGTTAGTTCTTCCTTGCATTGATCGAAGTTCTGATGATTCTCGCAATCGATTTGCAAGTGAGCTAATTAAGCAGATGAATAATATTGAGCATCAAATAAGTGGAGTTACTTCTGGATCTAGTAAACAGTCTGGATCATCTCCTTCTGGTAGTGAAGGCTCTATAACAAAATCTAGTACTAGAAAGAGCATGAAGGGAAGTAGCCCTGGTTTAGCCCGTCGAGCAGCAGCTGTGGCAGATTCAACTCCATCTTCACCTTTTGCTCTCCGGGCATCAGTTTCATTGCGGTTGCAGCTCCTTGTGAGACTGCTTCCCAATATTTGTGCGGATGGGGATTTGTCTTCTCGCAGTTCAAGATTCATGCTTGCTTCAGTTATACTTCGTCTTCTTGGAAGTAGGGTTGTCTATGAAGATATGGACCTGTCTCTCTACCCTGGGCGAGGGTCGCCACTGAAGGAAGCCGAGTTACAGATGGAGACTGGTGCTGATGTTTCTTTGAATTTATCTGGCAAAAACTTATTTGATTGGTTATTGCTAGTATTACATGCATTGCTGAGTAGTTCGCAACCCAGTTGGCTCAGATTAAAATCTCCCACCAAGACAACCACAGTGTCCCCTAAAGATTTTGCTGGATTTGATCGTGAAGTCTTAGAGAATTTACAGAGTGAATTGGATCGGATGCATTTGCCAGATACAGTAAAGCGGCGCATCCAAGCTGCCATGCCAGTTGTTGTACCTAATCTAAGAAATTTTATTTCTTGTCAGCCTCCACCTCTTTCATCTACTGCTCTTGCTTCACTTCAGCCTAGTGTCGTATTTCCTTCTAACCAAGCAAGCCCACTACCAAAGAATTCAGTGCCATCTGTACGCACAGCCACAGCTGCTGCCATGCTGGGAAAATCAAAATCGGTAGCGTCACAACCTGATAATGAATTGGAGGTTGATCCGTGGACGGTTTTGGAAGATGGGGCGGGTTCAGGCCCATCTTCAAGTAATACTGCAGTTATGGGCGGTGGTGACTATGCTAACCTTAGAGCATCTAGTTGGCTCAAAGGGGCCGTCAGAGTTAGACGGACTGATCTCACATATATTGGTTCTGTGGATGATGATAGCTGATCCCAATTAACATTGCATTACATTGTCTCTTAAATGAGCAGATCCTTCAGATATGGAACCCCTTGGAATATATGGCGAAGTCAAAGGGTGGATTTCATTCATTACTGTTCTTTACCCTTGGTTTAGCTGAAGGGCCAGATGGGAGACACTGTTACTTTTGTAGCCTGGGGGCAGAGGATTCGGGCGCTAATTCTTAGATTCTATTCCTGCCTGTTGCGGCTGCTCATCTGAAGTCTGCCTCATCAACTAACTGATGATCGCCACCGCCATGAGCATCGGTCGGAGGCTTGTATATATTTAGATATAATGGTCACCTTGATTGGTTGAGCTCAGTAAATCCCTGCTCTGTAGTGCTTTTTACTAATCATTGTCATATTCTTCACCCCTACCTCTGATGCCCTGGGTAGCTATTTATATTGTAAAATGTAGCTGGGGCAACAGAGTATTTTGTTTAGACTTCAAAACTCAGCATTGGAGAAATGCTATTGTAGATGATGGGTCTGAATATGGCTATTTGGCTAACTTGTTAGATATTAAACAGTGTTAGCCCTTCACCAGCGAAATTGCGGCCGCTCTTCAGTCTCCTTTTTTTTCCTTTCTATTCAATGTGGATATTGGGTAATTATTTTCGGTAATGATTGTATGCAAGACTGCTCCAAAGGGGTTTATTGATAGCTGCACTTTTGGGGCATATTGTGATGTGTTCAAGTACCACATTGTCGTTTTTGTATTCTAATTAGTCAATTGTTTATGTAGCTTTGGTCAAATCTTTTCTAATCAAATTTACACCCTTTGTTTTTAGGGTTTTTTTTTTCGTACTA

*S.maritima*37040

TGGAATGGAATGGAATGGAATGGTGAAATGAAATGTGAGCTGAGATTGTGCCACTGCATTCCAGCCTGGGTGACAGAGTGAGATTCTGTCTCAAAAAAAAAAAAAAAAGAAAGAAAAAAAAAGAAACTATTTCACCCATTTTGTTAGCTTTATTGTGCAAATTTCTGACAGTATTTATTTTATTTATATAAATACTTATATATTTTGTGGTGAGTATGAAAGTGGGATGGTATGGAGGTGGACGAGATTCAAACTCAAAATCAGCAGCAACAGCAACAACAGCAGCCACCGCCGCAGCAGCAACAGCCGCCTTGCAAGTATGCTAGGGTTGGCTCGAATGGCCGTGTTGATAGCAACGGTGGGACCCACGATGGTCACACTGAAGCAGTAGTTGCCGCCACAACGGCGGCAACTACTATGATGGTAGGTAATAATGTTAATAGTACTAGTAATAGTATTAGTAATAATACGGGTAGTAGATTTGGGAGGCAGTGGCAGCATTCGTCAAGGATAATAAGGGTTTCGAGGGCGTCGGGTGGTAAGGACAGGCATAGTAAGGTTTGGACAGCGAAAGGGCCGAGAGACAGGCGTGTTCGGTTGTCGGTAACAACAGCGATTCAGTTCTATGATCTGCAGGACAGGCTGGGGTATGATCAGCCTAGTAAGGCGGTTGAGTGGCTGATCAAGGCGGCTTCCGAGGCGATTAATGAGCTGCCTTGTTTGAATACAGCGAGCTTCCCACCTGAGACTTCGCCTAGTAGGCAAGCGAGCGGTGAAGAAGAGAGGAAGACACCGTTAGCAATATGTGGTGTGTTGGATAATATAGTTGATAATGCCGAAGGACAGCAGCAACAGCAACAGTTGGGGAGTAAATCAGGGTGTAGTAGTAACAACTCCGAGACGAGTAAAGGGTCTGGAGGTTTGTCTTTGTCGAGGTCGGAGATTCGGGTTAAGGCTAGAGAGAGGGCTAGAGAAAGGACTGCCAAAGATAAAGAACCATCTTCTTCTTTGGTTAATGTTGCTTCACATCAACAGCAAAATGTGAGTTCTATGGCGCAGCAAACGTCGTCGTTTACACAGCTTTTGACTGCCGGTATGAGCAGTCTGAGTGAGAGCAACAAACACTCTAGAACCCATAGCCACAACACCAACGCCACCGCCGCTACCACCACCAACAACAACCTTCAATGGTTGTCATCTAGAAATAGTAACAATACATCTCAGATGGCAGATTATTTTGGGACAGGACTAATAGGGCTTGGGAATAGCAGTACATCGTCACTTCCACAAGTGATGACAATTGCATCATTTGGGTCAGAAAATCATCATCATCATAATCAGATCAATCATCACCAACACCAACACCCGCATTTCCCCTTCTTGTCGGACCACCAAAACCTCTTGGAAGTCGCAAATGCGGCCTCGAATGGTAATGTCAGTGGTAATGGTGGCGGTGGTGGTGGTGATTATAACTTGAATTTCAGTATATCATCAACATCAACTACTACCTCTGGTTTCAATAATAGGGGGACCCTTCAGTCCAATTCGCAGTCATTGTTTCCTTACTTTCAAAGGTTTTCTGACGGATCAAGTTTGCCCTTCTTCCTTGGCAATCCTTCTGCTTCTTCGCCAAATGTCGAAAACCACCACCAACAGCAACAGCAACAGCAACAGGTGTGGCAGTGATGTCATCATCGGCAACAGCAGTGTTGATAGGGCGCAACAAGTCATTATCAAGTCCATCATCGCCCTCTACTGCTACTGCTGCTGCTATGAGCTTGAGCTTGAGCTTAAACCGGAAGTTTAATTGCAGAACAATAAACAAAAGAATCTGCTGTGAGGTGGCCATGCGATGGGAGTCAAATTCGACTTCAATGGAGGAAGAGGATTTGAAGAGGAAGATGGAAAAGGTTGGTTCGAAAGTAAAAGTGAAAGCTCCAATGAAGGTGTATCATGTGGCTAAGTTACCTGACCTGGAATTGACCCCTGATATGGTAGGTGTTATTAAACAGTATGTTGGGTTTTGGAAAGGAAAACATATTTCTCCCAATTATCCTTTCAAAGTTGAGTTTCTTGTTCAGGGCCAAGGCCGTGCTGGTGGCCCTCTTAAATTCTTCGTTCATCTCAAGGAAGATGAATTCCAAATCATTTCCTAACCCTAATTTTTATTAATACTTTACTATACTATACTTGCGGAGTATTAATCAATTCTACTTCTAGATGCTGTTTATTATATATATGTGCTTCTATCATTACCATTATCATTATCAATATATATGCTTGTTAATTACATTCACTTATTGTTACTACTGTTACTGTAATTGTATGTAACTGCCTTGTGACTTGCTTCGATTTTGGATGGGGCAGACTTGTTGTAATTATGGTCATGTTTGTGTTATGTGTGG

*S.maritima*40198

TTGTTCGTCGTTGTTATAGTTTTTGCCAAAACCAATCCTGCATATGATCGAGTTTGCTAGTGTTGTTGCCAATCCACTCAAATTTGCGAGCTTGGATTCCAAGGAGAGGGAAGAAATGACGCAAATCATTTGACGAACTTCCTCTTCTCGAATCCAGGAAAATGTTTGCACTCTTTTTGAGCTAAAGAGATGTACGACACATATTTTCCTCATTTCTCTATAATACTCATTGTAGGGTGTAAAAGTCAAATCTAAACCATTGTAGGACAGTTTTTGTTGGCTAACGAAGGATGGCCTACTACAAAACACATGATCTTGGGTTTTCATCACTTCTTTGGCTAATTTTGCTGAAGAAACTACAATTGTTGATATACAGCCAAGTTGTAAGTAAATGAGAGGACCATATTTTTTAGAAAGTTGGAAGCAATAAAAGTGTGTGTTTATGGGATCAAATTGATGAAGGTTACCGATGATTGGGAGTCCTTTCGGTCCAGGAGGACGATGAACTAGTGACAACGGCGTTCTTTCACCTTCCTGTTTTTTGCAGGATTTTGAAGGCACCACCAAGAACAGAACAAAGATTGCTGCAAGAAACGATAGTAGTAGCAATACCAGCAACATGATTGATGATGGCGCGTGTCTTTACGCACTCCTCTGGTTATGAGCGAAGTAGATTTCTACAAGAATTGGAAAGATGAATTATAGGTATGCTGTAATCTGTAATTCTGTTGCCCTGTATCGGTGGATTATGTTTTATGTGTGGTTTATCTTAACTTCTGAAATTCTGAAGTAGTAGCCATTATCTATATCAGTATTTTTGACTACTTTTGTATTAGACGTAACCTATTACAGGTTACATCCAACTATCCAAGAGA

*S.maritima*29592

GAGCAAGTAGAATCTGAAAAATCAAGAATTAAGAAGGGAAATAGAATCTGAAATGAAAGAATCTGAAAGAGACAGGAGGTTTCAGAGAATGGGGATGGAGCAAGAGCAACAACAAGAGCACCAGCAGCTTCTATTGCATCATCATGACATTCATGAGCATGGAAACGTTGCAGAAAATAAATCAGAATCAAAGTTTATGAAGATGAAGAGTGCAATAGTTGGAACAGAGATATGGGATGAACTTAACAAAATATGGAAGATAGCAGGACCTTCGATATTTTGTCGTTTCGCAATGTTTTCACTAACTATCATCACTCAATCTTTAGCTGGTCATTTGAGTGATCTTGATCTTGCTGCTTTCTCCATTTCCACCACTCTCCTCATTGCCATCAGCTTCGGCTTCCTGATAGGAATGGCAACTGCATTGGAGACATTATGTGGGCAAGCCTATGGAGCTAGACAATACCACATGTTAGGCATCTACTTGCAGCGCTCATGTGTACTTCTCTTTGGTTGCGCGGTTTTTTTGCTTCCCATGTTCATTTTCGCGTCTCCTCTACTTAAGCTCCTTGGTCAATCAGCTCAAGTGGCAGAACGGACTGGCTTAGTGGCCAGATGGTTGATCCCCATGCACCTCAGCTTTGTATTTCAGTTTTCATTGCAGAGATATTTGCAGAGTCAGTTAAAAACAGCTATCATTGCTTGGTCTTCTGCTGTGGCTTTGGCTGTCCATGCCATTGCTAGCTGGTTTTTTGTATATAAGCTTGGTGTTGGTGTAATAGGAGCTGCCCTTATTCTTGATTTCTCCTGGTGGCTTTCAGTTTTTATAATGTATGGGTATGTTGTTTATGGGGGATGTCCTATGACATGGAATGGCTATTCGTGGCAAGCCTTTACTGGATTGTGGGACTTCTTCAAGTTATCCTTGGCTTCCGGTGTGATGGTCTTGTTGGAGAATATGTATTATCGGTTGCTAATTGTAGCATCTGGGGATGCGGGAAACTCACATGTTGCCGTTGATGCTCTCTCCATATGTGTGACTTTGTTTGGCTGGGAATCCATGGTTCCCCTCGGAATTCTTGCTGCCACTGGCGTACGAGTAGCCATTGAGTTGGGAGCAGGAAATGTCAGTGGTGCCAAATTTGCAGCGAAAGTCTGCATGCTGACATCTTTAGTAATTGGAGTCATATTCTTCTTATTGGCTATATCTATCCCTGACCGCTTGGCAATGATATTCACCCCTAACTCATCTGTGATCTCTGTAGTTCGTGAACTAGCTTTTCTCTTGGGCTCTACAATTCTCTTAAATAGCATTCCTCCAGTCCTTTTAGGTGATTGAATTTCATAGAAAGGAGAAAAAAGCCCTTCAGGTTGACTTGTTCGTAGTGTATAACAGCACCATATGATTTTCCTCAGGCATAGCAGTTGGATCAGGTTGGCAAGTGTTGGCAGCATGCATAAATATTGGAAGCTATTACTTGATTGGCTTTCCTCTTGGGATTCTTATGGGACGAGTGCTAAATTTTGGATACATGGGAATTTGGGCTGGAATGCTTGGTGGGAGTGTGGCACAGATACTGCTTTTGGCTGTTGTTGCAATCAAATTTGAATGGGAGAAAGAGGTTCCGAAAGCTTGTGTTAAACATTTTTTGAAGAGTAAAGCTGTGGATGACTAAGCAATTTGATCTACTACATATGTTATGTCAACTTAAGAAGCAATTGCCAAGAGTCACTTGTTTATTTGATCGTCTGCAAAGGCCTTTTCTTGCTACATTTTTTTGCTTGCAGAGAGCTCAAGATAACCCATTGTCGCTGTTGGGGAGATACAACGATTGCTAAACTCCTTTCAGCTTTTAGACATTACAAAGAAAAATTTCTCTGGTTTTAGCCACCATTTCATGGACTTATTATTAATCTTTTTAATCTTTTGATCTTAAACTTAAAATTGCATCTATACAACATGTGTTATAGGCTTATAGCCATAGGTTACTTGAAACTGGTTGTAGTGTTATAATGGTAAAATTAATGGGTAAATTGTAAAACACTGCTTAAATAGAATGGATTGAATGAAGG

*S.maritima*29358

CCCTTCCCTTATATAATAGAGATGATGAGCAAAGAAGTCGACGATACACCGTCGTACATGACATCCGAACCTAATATGACGCTGTATCTTCCACCATCTGTGCTCACAACACGTGTCAACTGGGACATATTCTTGAGTTTTAAAGGACAGGACACTCGTCAAACATTCACAGGACCATTATATGATGCCCTGAAGTCAAAGGATTTAAGACCTTTCATGAATGATGAGAGCATGGAGGGAGGTGATAAAGTGCAACCAAACCTAGATGAAGTTATGCAAGACTCGGCTCTTGTAGTTGTCATCATATCACCAAACTATGCTGATTCACATTGGTGCCTCAAAGAACTTTCTAGTATCTACAAATTGTACAACAAAGATTCTAAGAAGTGGGAGAAGCGGGTCATACCTGTTTTCTACAAAGTTGATCCATCTAATGTTCGGAAGCAAAGAGGGGTGTTTGGTGATGGATTTCAGAAACTTCTGAATGACTCCTCAAAAAGAATTAGTGAAGAGGAGACTAAAATGTGGAGAAATGCTTTGGAAGCTGTTGGTAATATTTCTGGCTATACATGCCCTATTCAAAGTGATGATCATGCTGAAAATATACGGCGTATCGTCCAACAGGTGTGGAGTGAGTTAAAGAACACCCCAGAATATGTGGCAAAACTTGATGTTGGTTTGGATTCTCGTGTCAATAAAGTGGTGGACTTTTTAAATCTTGGTCAGCCAGATGTTCGGTTTCTGAATATTCATGGAACTCCAGGAATTGGCAAGACAACCTTGGCTAAAGCCATCTACAATAAGCTTGTCGTGAAATTCGAAAACCGCTGTTTCATCTCTAATGTCAGGGAAAGATTGACGCATGAAAGTGGCATTTTAAGTGTTCAAAATGATCTTATAAAAGGTCTCTCAGCAGGAAAGGTACAAGCTGTGCAGCATGATGGGAAAGAAACATTAAAGAGAATAATTAACCAGAATAGGGTTTTATTGGTATTAGATGATGTCTTTGAGGCAAAACAACTTGACGATCTCGGCATTGTCAGAAAATGGTTTACTGAAGGGAGCAGGATTGTCATTACTAGTAGAAATGTAAATGCGTTGCAGAAATTGGAGGGTAATAATGAGCATGAATTTTATGAAGCAAAGGAACTAGATGAATTGGAATCACTAGAACTATTTAGCATACATGCTTTTGGAAGAAGGAAGCCTACACCTGAATTCCAACATATCTCAAAGAAAATTGTTTCTCTAACCGGTGGACTTCCTTTGGCATTGGAAATCATTGGTTCCTCATTGGTTAAGAAGACCAAAAAAGAGTGGGATGATGCTCTAACTAAATTAGAATGCATTGATAGGCCACAAAATCTTCAACAAGTTTTAAAAATCAGCTATGATGGTCTGGAGGACCAAGAGAAAACTGTCTTTCTTGACATTTCATGTTTGTTGCTTCAAATTGATACGAAGAAAGAGGACATAGTGGATGTTTTAGAGAGTTGTGGCTTAAGGGCTGAGTTAGCGATTGATGATCTTGTCAGGAAATCACTTTTGAAGATAACAAAGCGAAACGCATTGTGGATGCATGATCAAATACGGGACATGGGAAGGAACATTGTCATAGAGGAAAACTCAGAGAATGCAGGGAAGCGCAGTAGGCTTTGGGACTCTATGGACACATTGCGTATTCTGCGTCAAAAAAAGGTGACGGAAGCTGTGCGAGGTATTGTTCTAAATTTTAAAAAGCCACCAGATTTGAGTAATGAAATGCTATATTGGTTGAACTTTCGAAGGCATCCCAGTATCAAAAGTTTCAGCAGGTATCTGGTAGAAAAGTCTAAGGAACTTCCATCATGTCAGAAACCAAATGAGGATGATGATGTCATTCTTCATTCAAGTTGGTTTAAGGAAATGAAGCACATGAAATTACTTCGAATGGATTATGCCAAATTACGAGGAAGCTATAAATACATGCCTTCTGAGCTAAGGTGGTTGCAATGGAAAGGCTGTCCTCAAAAGACTCTTCCTGTTAATATTTCAGAAGATCTTCGTGTGCTTGATCTTTCAGGTAGCAATATTGAACAATTGTGGATCCAAGATTATTCATGGTGGAATTCCAACACGGTGGTTTGGAACTTGGTGGTGCTAAATCTCTCCTACTGTCCCCGTCTTACTGTTCTTCCTAATATGTCTGGGTACAAACACTTGAAAAAACTTAACCTTGAACGGTGTGTAGGATTGACTAACATACACAAGTCAATAGGAAACATGACTTCTCTAATTTACTTAAATCTTCGAGGTTGCATAAACTTGGTCGAGTTTCCTAGAGATGTAACGGGGCTGCAAGGTCTTAAGGAGCTTATTCTATCTGACTGCTCAAAATTCAGCGAACTTCCTGAACAAGTTCAGAGCATGACATCTTTAAGCCGACTATGCCTCGATGGAACAGCTATTACTGAGCTCCCTGAAACTATATCTAACCTTACACAGCTTGAAGTGCTCAAGTTAAACAATTGCAAACAATTAACATGGCTTCCCGACTGCATTGGATATCTGACTTTTTTGAAGGAGCTTTCTCTTGCTCATACTAATGTGAATGATCTGCCTTGGTCTGTTGGATCATTGACTCACCTAGAGATTTTAGACTTAGTGGGCTGCTCCTCTTTTGAAACACTTCCTGATTCTGTGGAAAAGCTCAAAGCCTTGACTGCGCTGCGCCTTAATTCCAGTAAAATCAGTCAACTTCCTGCATCTGTGTGCTCTCTATCCTATCTAAGGGTCCTATCACTAGAAAGATGCCGGTCTCTTATCGAGCTACCTATCTTATTAGGAGGGTTAGCTTCCATTCTTGAGCTTGTGCTAGACCATACCCCTATTAAAGTACTGCCTGATGAGATATGTTCCTTAAAATCCATGGAGAAGCTTCAGATGGATAATTGCAACTTGCTTGAAAAACTTCCAAAAAAAATAGGGCAACTATCAAATCTTTCTGTTCTTTTGGTTGAAGGTGCTGCCATAACTAAACTTCCAAAGTCCATAGGATATATGGAAAATCTTAAATATTTAAAGTTGAACAGGTGCAAAAAGCTAAGCAGCCTACCAGATTCCTTCGGAAAGTTGGGGTCTTTGCGCACACTTATGATGGAGGAAACTGCCGTGACATTTTTGCCTGAAACCTTTGGGTTGCTCAAGGGCTTAAGGATGTTAAAAATGAAGAAGTCTCGGAATGACCCTGCTGAACTTAGTTTGCAGCTTCCTACTTCTTTCTGCAAGTTAACTAATTTGGAAGAATTTTATGCTGGGGCATGTGGAATCTCTGGTGAAATTAAAGATAACTTTGCTGAATTGTCTAAGCTGGAAATTTTGGATCTAGGGTTCAATAATTTTTATAGCCTTCCTTCCACACTTATAGGTCTCTCTTTTCTAAAAAAGCTAGTTTTGCGCAATTGCAAAGAGCTTAAGACCCTGCCTGGTCTCCCTTCTACTTTGGAGGAATTAAATGCTGCTGACTGTGTTGCACTTGAAAGAATATGTGATCTGTCAAACCTCGAAAGATTACAAGAACTACGACTATCTAATTGCAGCAAAATTGTAGATGTTCCGGGTCTTCAATGCTTCAAGTCACTT

*S.maritima*39187

GAGGTATCAAAGTCAAAAACTAGAACTGTAATTATTTTTTACTTGTGTTTACAAAACAACTTTTTAGGCAGTGTATTATTTCTACTACTATATGCTAAAGAACTTATTTACTACCTTCCTAATTTGAAGATGTTTCGGTAAACTTCCTGTAATTGAATCATCACAAACATACATGATGTCACGTCTTAGCCGGTAAACAAAATCGACAACAATCTCATTATGTGTGCTTTTGACTATTCACAAGCGTGTTGCATCTCAGTCTCAACATAAGAGGCTTTGAACTATATCTAGTGTGAGTCTTGCTCCAAGCAAATGGAAGTTACAGGCTTCTGAGGAAGATATTTTATCCAGATAGGAAAGGAAGATGAGCAAGTGTAGTTGGCAGGGAAATATTGAGGTTGTGAATTGTCAAATACGCTGCAATTCCTGCTGCATAACCAGCAAAAGCAAAACCACTCACCTTCCTAAAGTACCAAAAGAAATCGACCTTTTCCATGCCCATAAAGGCAACTCCAGCAGCAGATCCAATAATGAGCATGGAACCACCAGTACCAGCACAATATGCAACCAACTGCCAAAATTCAGAGTCTTTTGGAAAAGAAGAAACGTCATACATCCCCATTGTAGCTGCAACAATAGGAACATTATCTATAATTGCTGAAATAACCCCTATTGCACTGGCAATCAATTCACTACTTGGTACATGGGCATCCAAGTAATTTGCCAATTCACGAAGGATTCCTGCTGCTTCCAAGCTGCTAACAGACAACAGGATCCCTAGGAAGAAAAGAGCTCCTTGAGTATCAATTCGAGACAAAGCTTGAGGTACTTTCAATTTTTGTCTTTCAGATTCACCATAGTGAATAGCATCTGTCAGTAGCCAGAGGACACCAAGCCCTAAAAGCATACCCATATACGGGGGAAGACCAGTCATGCTTTTGAATACTGGAACAAAGACCAAAGCACCAATACCCACTGCAAAAACAAGCTGCCCTCGAGGTGCCATCTGCTCAGATGCCAAGACACTGGAAGAAGATTCTGGTTCCTTTCCATTGACTTCACTTGTAAGAGACATAAGTGCCAATGGAACAGCCAAAGATACTGCAGAAGGTATAATCAAGTCCTTCATTGTTGGCAGAGTGGATATCTGACCATGTATCCAGAGCATAGTAGTGGTAACATCACCAATGGGAGTCCAAGCACCCCCAGCATTTGCAGCTATGACAACAACAGCTCCCAGTATCTTGCGGTACTCGGATGGGGGAACTAGTTTCCTCAACAAAGAAACCATGACTATTGTAGACGTAAGGTTGTCAAGAATTGCACTCAGAAAAAAGGTTACAAAACCAACCACCCATAGGAGAGTACGCGGTTTACGTGTGGTGATATTCTCAGTAACAAGCTTAAATCCTTGATGAGCATCAACTATTTCAACAATTGTCATTGCACCTAGCAGAAAAAATACAATTTGACTGACTTCGGCAGTGGCATGCTCTAGCTCTGACACAGCTACCTCCGTAGATGGAGCCCCTAAGCATCTTACGACCCACAGGCTAACTGCCATGATTAATCCTACTCCACTTTTGTTAAAGGCCAGCGATTCCTCAAATACTATCCCAACGTATCCTATTCCGAATAGCAAAATCATAGCAAGATCCTGATTAGCAGCAACCCAAGAATGGTTAATTGTTGCTGCACCAGTCAAAGCAGCACCGAAAATCGCAAGAGCTTTCAGCAAGTCAGTCTTTGGCTGATAATTGGCTTCTAAATCTTGGGAGCTTGTTTCATCAACTGAACATAAAGGGTCACATTCTCCTGACGTTGGTGTCAGATCCTGAAATTGAGTTGGAGAAGAATTTTTGGCCTTATCTTCAGCTCTAACAATCAGACTTTCCCTAAACAAATTTCTTCGACATTTGTTGTTCCCGAGGTTCGAACTCGAGTTCGCCGGAAAACTAACAGTAAACGGTGAAGTTCGACGGTGCAGTAACGGCGGCGTGAAGTACGAAGTTAGAAAAGGGTGTTTGACTGACGGCCGTGAAGTAGACAAGGAGAGAGGAGACGCCATTAGAGAGAGACAGAGACCACACAGAGCAACAATTAGAGTTGGAAAAACTGTAATTGCCAACTTTTCTCAACTACTAATGTGTAATTTAGTTATTATTGTTTGTTTTGTT

*S.maritima*32301

CTCGTTTCTCTCTCTTATGTCTCCTACCTTGAGACGAGATTTTCTTCCTCATGGGTCGGATTTCAATTTGCTTTCCCAAATTTTAGTTTCTTCTAATATCCTTCAACGGTGAATTGGTACATTTAGCCTATAGAAAAGCTGTTTAAAAGTGTATTTAGATTTAGTGATGCAGAAGAGTGCGAATAATCGCGAGTTGTCAGCTATTGGTTCTATGCCTTGGTGGATTGCTGCAGGATCACAAGATGAACAAATGAATTCATCTAATTTCAATCAAATTGATTGGTCTGATGGTGGTCACCAGAAACCACGAACTTTTTCGAATTCAGACCACGTTATTGGTGGAAAAATTGGGAAAGAAAAGCAAGGATCTTTCAATCTTTCCTTTTCAACTGATGAGAGCAAGAACTCTTTTAAAGAAAACAATGCTATCAATCATCAAACAAGCAGTTTCCAGCAATTCTCTCCTCCCCAGAAGAGTTATTTTGAACTTGGACTTGGTCAGCCAGCAGTCTATGCAAATTATCCTGTAGTAGATCAATGCCAGGGACTATTTGGTCCTTATGGAACTCAGCCTGTGGGACGTCTAATGCTTCCATTTAGTGTGACAACTGATGATGGACCTATTTATGTAAATCCAAAACAGTATCATGGGATTTTACGACGCCGGAAAATAAGAGCAAAGGAAGGATTAAAATACCGGTCCGCCAGAGAGAAGCCGTACATTCACGAATCGCGACACCGCCATGCATGCCGTCGAGTCAGGGGTGCTGGTGGACGCTTCCTCAACACCAAGAATCTTGACAATGAAAAGAACAAACTGCAGGAAGCTAAGGCTGGATGTGATCAGTTAACCTTGCCTTCCTCGCACTCATCAAGCTCTGAAGTTCTTCAGTCAGAAGGTGGAAACTTGAACTCCTCGAAGGAAGTAGTCTCCTCTGACTGCAAAAGCTCAAACTTTTCTGAAGTCACTAGTGTCTATTACAGCAAAGAGTTTGATTGCTTCGGAATCAATCCTGGCCGTCCTTTTTCCTTCCACCCTTTACCAAATATGGAAGGTAACAGGCACGACAATAACTGGGTTACGGCAAGTGATGGATGCTGTAACTTCTTCAGAGTTTGAGCGAGAAAAAAGAGTACTAAAAAGGACGGTTGCTTAAGTTCTGAAACACTAGTTTCCGAATCAGTGACTGTCCTGTACTCTAGCCATTACAATGGAGTTTAGGGTGCATATCTGTACGAAGCTCCTTGTAGTGGTCTCCCTTGCTCTCTCTTTCCCATTTGGTTACATTATCGAATGGCAAGTCATCCTTTGGCTATTGAAGGTCATCATCGTTGTTGCGCAATCGGGACTTGCGAGCTGTGTGGCTTTAGAGTGATTGCAGTAACAATAGGCCTTCTTGTATTATATTAAAAAAAAAACCTGATACTTATTTGCTTCAATTTGTGATAGGCTTGGTAGTCTTAACTGTCAAGTCATAACATGAGGTTTGATTGTATGATAAACTTATTTTTTTCAAATGATGCTTGGATTGTGCTTTGATCTTTGATTGTTATGTGTACAAGTGTAAAACAATGCTACTTTGACTATGTAATATAACTTTTGCTTGAA

*S.maritima*13778

CTTGTATTAATCTTGTTTCTTAATTCTGTATTCAACCTTCTATTTCTGCTGTTGCGATTCTTTCTCCCTAATTCAGCTGTAGGAGATTCCATGTCGACAGAGCTTTTACATGATGATCTACTTGCAGAGATTCTTATAAGGTTACCAGCAGAATCTTTGCTCAAGATCAAATGCGTTTGCAAGTCTTGGTATTCCCTTATCAGCAGTTCCAGGTTCATCTTGGCCCATGTCTGTCACAACAGATCGAAAAACCCTCGTATCCTTTTGCGAAGTTTTAACAAGGCAGACAAGAAGGTCAGTTATAAACTGTGCCATGATAACGAGTATTTGGATGGTATTATGACTATTGATCCCCCATTCATGAGGCAACGTAATGATTTTTTAAGGATGGTGGGTTGTATAAATGGACTTGTATGCTTGTCAGATGACACTGTTGAAGTGACGGATAGTGTGATATTATGGAATCCGGTGATTAGGAGATTTCTACCTCTTCCCAAACTTGAATTAAATGTTGACTCAACTGATTTGGGCCGATCAGTTTTTGGTTTCGGCTATGATTCTACTAACAATGATTACAAGGTTATCAAGATTGTATACCGTAAGAATCCAGATTTTGAAGCCCGCCAAGTTGAGGCTTCAATTGCAATTTATAGACTAAGCTCATGTTGTTGGGAAGTCAATGGGTCTGCTTCAGTCCCTTTGCTTGATTCCCGACAAGCTTATGTAAATGGAGTTATCCATTGGTTGGCTTATAATAAGCTAGTTGTGGGGTTTGCTGTGGAAAGTGAGGCATTTAGTGACACCATGCTGCCTGAAACCTTGCAAAATGCCAATATTAGTGATTTAGCAATCGCCTCATGGTGTGACTTGCTCTCTGTGTTTCAAAATGGGTTTTGGTCTGGCAGACTTTGTTTATGGGTTATGAAAGATTATGGCGTGGCTCAATCATGGGTTAAACAGTTTGTGATTGAATCCTACGTGATGGTGAGAAGTCTTAGAAGGAATGGCTGCGTTATACTCGAAAATATTGGTGGTAAGCTAGTTTTATACAACTCAAAGACCAATCAGTTTGAGGAGTTCAAAATCCATGTCGAGGGTTCTATAAGAGGTTTTCATATGAAATCATACGTAGAGAGCCTAGTTCTATCAGATCGGTTGGATGCAAAATCTATTCCCTAAAAATCGAGTATGTATACTTAACTTTAGTTTGCAATTAATTGCAGGATTCCCTCCCGTCTCCTCTCTTTTTCCGGTTTGTGCGTGGATGCATGTGCCTTTGTTTCCAATTGTGCTTGTAGATTATAGTTTCTCTATCCTTGTATAAATTCTGTATAAATTCCGGGATACTCATGAACCTAGAATTTCTCTTCTACCTTCGATGAGGTGAAACTCTGGAAGTAGTTAAACTGGGAGATTTTCAATTTCACTGACCCTGTATTACAAAAACTCATTTCATGCTGTAAGTATAAACTGTAATGCTCCAAATGTATCAGCTCACATTGCAAGTTAATCTGATGCTAGAATTCTGTTGTACATGTGATTGGATTGATGATTTCGGC

*S.maritima*44824

TTTTGTATAAACAAAAAAAAAAAACCCAAAAAAAAATTAAAAAATCCCAAAATTCATATGAAGAAGCAACCTTTTTTCCTGTAGAAGAAGGGGAAAAAAAAAGAAGATAAGAGGTTGAAATGTTGGTGCAGAAAAGAATAGATTTTATGGCACAACCTTTAAGCATCATGATTATGAGTGGAGATAATTTTTGCTCTGTTTCTTTCTTCTTCTTCAACCTTCTTCCATTCTTTCTTCTGTGAAATGGGATTTTAGCTTCTGAAACTATGATGCGAGCTATGCCCTACAATTTGCAAGGCAAGGGTGTGGTAGAGGTTTCAGGTTTAATTTCACAAATCTCTTCTTCAGTTCCTCCAAAGTGGAAAAATATAGACAAAATTCAACAACAAAATCAACAATTTGCAGCAACAACAAGAGCATCGTTTGAAGACGAAGAAGAGTTTGAAGAAGAATTACAAGTAGTTAGTAAAATCTCAGGTGTTAAAAGAAGTAGTTCAATTGACAGCGAACCCACATCTACTCTGGATACTACCACTAGGAGAAGCCCTAGTCCTCCTACTTCTACCTCAACTTCTACCCTTTCCTCATCTTTCAACAACAACAATACCAAAGATAATAGTATTGCTAACAATACTGCCGCAACAACAACAACAACAAGTGCTGGTGCGGCTGCGGCTGCGTGTGCCGGTGTGGCGAACCCTGGAGCATGTGTGAGGAAAGAGGATTGGGGTTCGGAGCTCCACCATCATCAAAGATCTGATGAGAATGAGAATGTTGCCACTGCCGCTGTCGCTACGGTTGGAGTTGGTGTTGGGGGTGATAGATTGGTGGAGAGTCAGAATACAACAAGTGGTGGATTGGAAGATTGGGATACAATGTTTCCAACAGGTGAAGGAGCTTTGCTTCCTTGGATCATAGGAGATGCTGAGGACCCTAGTTTGGGTTTGAAGCATCTTTTGCAATCTGCTTCTACTCATGTTGTTGATTATGAAGGGAATGCTGGTTTAGGTGTTGTTGATCAGGGTCCTGGTTTTGATGCTCATTCTCATAGTCATAGTCCTCTTGCTGTTGTTGGGTCTCAGGCGACCGAGGCCGGTTCCAATTCCGGGTTTCTTGGGTCTGAATTTGGGAATAATGGAAAGATTGGGAACTTGATTTCGCCGAATTGTTCAACTGGGGTTATGGATAATACAAAGGTTAGCTGTTCTAACAATGTGTCAAATAGTCTGCTTTTGGGTTCAATTCCTGCTAGTTTTTCTCAGCAATATGAGTTTGGTGATGAGAAGCCACAGATTTATAACCCACAGTTGGTGATGAACCAACAACAGGCTCAGAGCCTTGCTAACCCTAGCTTCTTGATGCCACCATTGGGGTACTGTCAGTTGGACCAACATTTGGGGTTTCAGCCACCCAATAAGCGGCATAACCCCGGCGTTGTTTTGGACCCGAATCTTGGTGTGAAGAACCCTTTTATTGATCAGGGTCATGACCTGTTGTTGAGGAAACAGCAGCAACATCAGGTTGGTGGGTATCAGCAGCTGCCCTTGGGGTTGCCACCGCAGCTGGTGCCTCCCCATCTGCAGCAGAAGCCGATGATGTCAACGAAGCAAAATAACCACCAGCATCAGCAGCAGCATTTCCCATTGCCTATGCTTCAGCAACAGCAGCAGCAAGAGCAGTTCATAAAAGAAACGCTCTATAAGGCGGCAGACCTGATACAAACTGGGAATTTCTCACTCGCGCAAGAGATATTGGCGCGGCTCAATCACCCGCTTTCCCTCCCTGCAAAGCCCCTCGATAGGGCGGCTTTGTATGTGAAGGAGGCTCTACAAATGCTCCTTATGATGGGCAACCCAGTTGCAGCTCCTCCGTCGTCTAAGAACCTCACCCCTTACGATGTTGTTCATAAGATGAATGCTTATAAGGTGTTTTCTGAGGTCTCTCCTATTACTCAATTTATGAATTTTACTTGCACACAGGCTATTCTTGAGGCTCTTGATGATTCTGATGCTATTCATATCATTGACTTTGATATTGGTTGTGGTGCTCAATGGGCTTCCTTTATTCAAGAGCTTCCCTTGAGGAAAAGGGGTGTTCCGTCCCTCAAAATTACTGCCATTGTTTCGCTTTCTACCACTCAACCCTTTGAAATCAGCCTCATTCGTGAAAATCTTGTGCAATTTGCTAATGATATTGGTGTTCCTTTCGAGCTTCAAGTTGTTAATTTAGACTCGTTTGATCCATCTTCATGCTCCATGCCTAATTTCAGAATCTCTGAGGAAGAGGCTATTGCTGTTAGTATCCCGGTTTGGTCATCATCCAATCGGCCTTCCATCCTCCCACCGGTCCTTGAATTTATTAAGCAGTGCTCCCCCAAAATCATCGTCTCTTTGGATAGAGGCTTTGATCGCTATGATGTTCCATTCCCCCAACATCTTGTTTATGCCCTTGACTCCTGCACCAATTTACTGGAGTCACTAGATGGTCATGTAGCCTCAGATATCGTAAGCAAGGTTGAAAAGTTTTTCGTCCAGCCTAGGATTGAGAACACCATATTGGGACGCGTCCATTTTTCTGAAAAGATGCCACATTGGAAGAATCTGTTTGCCTCAGCTGGCTTTTCGCCTTTGCAATTCAGCAATTTCACTGAAACTCAGGCGGATTACGTGGTGAAGAGAACTCCGGGGAGAGGATTTCACGTGGAGAAGAGGCAGGCGTCTTTGGTACTAAGTTGGCAGAGACGGGAACTTGTGGCAGCTTTGGCGTGGAGATGTTGAGGTGAACATGACGCCGTATCCATCAAGCTAGGAGGTTTGCTTCTCCATATTGGTCACATTGCATTTCCAGAGGTTTTTTTATAAATTATGTGATACATGATATTGTCAATAACTAGAAGCTATGGTGATGATGATGATGACACCTCGCTTTGTGGCTTTAAACTGTTTATTATCTCCTTGCTTGTGAGCATCCCTCACTCATCGTGCCTTTCAGGTCGGCGTATTATTTTGCTATGTACAACCAAACAATTAACTTCAAAATTCAGTAGCTTTTATGTCTTTCGACAATCTAATTGTTAAGTATCATATTTTTCTGTCCATGATTTGATTCACTGTCAAATTTGCTCAGCTGCTGAAAATGTCTTATTCACATGTCTGTTATCTATGTGATTACATCCGAGGATTAGAAATGAAGGAAACGATATTGTTGGTCCATGTATGCACTTACCGTGCTT

*S.maritima*717777

TGTCAGGTAAATATCGCATCCGGTCAACTGATATTTATATCACAACGGTAATCCGGCGTACATAAATTCTTCTATATAACATTCGTTAGCGCCTCGTAGAATTTTGACGAATCCGTTGTCACCCCAGTACGTATTGAACGAGTTGGCTGCCAACCAGTACGGCGTGCCGTTTTCTTCTCCCCAGCCTAGGATTTTCACCGCTTGAGTGGTGACGTATTTTCCAGAGTTGAATGCATACACGCCCGATTGATAGTTGACAAAGTCTTGATACATGTAAAATGACGCCGTTATTGGACCGTTTTCGTAAATTTCTTTCATCGCTGTATATCCCGGGATACGATATTGCGTTCCTTTATGGTTGTCTTTCACGTATCTCGTCCCATAATCGGGGTTGTAACATTGAACCTTGCACTCTGGAGTGAAAAATGTTTTATTCGAGCACGGGTTTTCTACTGCAGTTTCCGTATGCTGGCACGGTTCAATCGTGTACGGTTGACATCCCTCGTCCGAACCGTATTCACCCCCGGAAACCAATCCGTGTCTTCTGTAGAAGTCCCAAGATTCAAAATGGCTGCCACCCGAACAGCCATCTCCGCACAGGTAGCAGCAGGACAATATCTGTTGTGCCGACAGTTTTGGTTTTATCGTCCCATTTGAATGGATGCAGATCCGGTCACTTACAGCTGACGCCACGGAAATAGCGTAGCTTGACTTGCAGTTACCTTGGTCGTATATCTGTCCAATGGACGGACACATGTACCATTTTGCCCGGGCGTCGAAGCTTTTCGGCAGGTCCAAAGGATCCCAAAACCCATTGTTCTTAGTTTTCATGGCGCCAGCTGGAGAATGTACATTGTCTAAAAAATCATGTTTATCGTGTAAAGATGCGCATCGTTCACTGATAACGATTATGCACAGCAAC

*S.maritima*35742

ATTGAATTGAGAATTCCAAATTTTCCAAATCACATTTGATTTCAACTTTCTCTCTTTTTTCTTTCGGTTGCATATTATAATTGCTGTTACTCACTGTTTTTTTTCCATTCTTCCTTTCACTGTACTCCACCATCATATTTTTCTCATGAACAACAACAAATTACTGCAATTTACTCTCAGTTTTTGTTCCTTTCATTATCCTCAATTTGCTTCTTGATTCTCTTTAATGGTGTTGAGAGCCTAATTTCGACAAAAATTGGAGTAATTTTGGTTCATTCCTAACGCAAATTTTCAGCATTCAGGTATTAGGCTGATGGTGGTTGGAGAATTTAGTTTATAGATTCACAATGTCTCATTTTAAAAAGGGTGTAGATGCTGCCTTTTCAGGTGCTGGAACAAAAGTCGGTCTAGAGACCTGGTGTGTCGAGAATCTGAGCTTGGTTCCGGTACCGAAGTCATCTCATGGGAAATTTTATTCAGGGAGTGCATATTTGCTTTTAAATACAATACTATTGAAAGATGGAATACTTGAACATGACGTACACTACTGGATTGGAAAGGATGCAAATGAGGTGGATTCACTGATGGTATCAGATAAAGCTCTTGAATTAGATGTTGCCTTGGGATCTTGCACCGTGCAATACCAAGAAAAACAAGGTCAAGAGTCTGCAAAGTTCTTGTCATACTTCAAACCTTGTATTATTCCATGTGAAGGAGTTTATACATCAGGACCAGAGAGGTCAGCTAGAGGCGCATACCATGTCACTTTGTTAGCATGCAAGGGTGAACATGCCATTTCGGTTAAACAAGTGCCTTTTTCACGGTCATCTTTGAACCATGATGATGTATTTATTCTAGATACAGAATCAAAAATTTATCTCTTTAGTGGTCATAACTCTAGCATACAAGAAAGAGCAAAGGCTTTGGAAGTCATCAAGTATATCAAGGAAGAAAAGCATGGTGGGAAATGTCTGGTAGCTACAATAGAGGATGGGAAGTTTGTTGGTGATTCAGATGCAGGGGAATTCTGGAGCTTTTTTGGGGGCTATGCTCCTATTTCTCGTGACTCATCTTCCTTTTTCAAGAAACAACATGATGCTCCTTTTGTGAAGTTATTTTGGATAAACACCCAGGGTAAACTGTGTGAAACTATGAGTGTTCTACTTAGTAGGGAATTGCTAGCTAGGGAAAAATGCTACATGTTGGATTGTGATGCAGAGGTATATGCTTGGATGGGAAGCTGTACTACAATCACAGAGCGTAAAACATCAATAGCTGCAATAGAAGATTTCCTCAGATCCCAAGGCAGGTCAACCAGGAGCCTTGTTACTGTCTTGACAGAAGGCTCAGAAACTCCCAAATTTAAGTCATACTTTGTTGGCTGGCCTCAAACAGATAACCCCAAACTTTATGACGAAGGCAAAGGGAAAGTAGCAGCAATTTTCAAGCAACAAGGATATGATGTAAAGGAGATTCCTGAAGAATGTTGCGAACCATTAATCAATTGCAGGGGAAAGCTTGAAGTTTTTAAGGTAAATGGTGATCAACTGTCTATTGTGCCTTCTGAGGAAGATGTAAAGCTTTTTAGCGGGGACTGCTATATCATGAAGTATACCTATCTTGGTAATCAAAGGGATGAAAGCATACTATATGCCTGGCTTGGCATTGCGAGCGTCAAGGAAGATAGAGTTGATGCAATCTCCCATATGGTTGATATGGCTAATTCCACAAAAGGAGCTCCTGTTTTGGCACAAATTTTTGAGGGTGAGGAGCCAAATCAATTTTTCTGGATCATGCGGAGACTTATAGTTTTGAAGGGGGGTATTTCCACCAGATACAGAGCTTCCATAGCCGTGAAAGGCTTTGCTGACAACAGTTATGATGCCAGAAGCACAGCGCTTTTTCGTGTTCAGGGATCAAGTTCAGAGTATATGCAAGCCATTCAAGTTGACTTGGTTGCAGGTTCCTTGAATTCATCGTATTGTTATATCTTACAAACTGAAGCTACTGTTTTTTCCTGGATTGGAAATTTAACTTCAAGCAGAGACCATGATCTTCTTGACAGGATGTTGGAACTGCTCAATCCAGCTTGGCAGCCTAAATCTTTGAGAGAAGGTGGTGAACCTGATGAATTCTGGATGGCCCTTGGTGGAAAAACTGAGTATCCAAGAGAAAAGAAAATCAGAAAGCATATAGAAGATCCACATTTATTTGTGTGCTATTGCTCAGAAGATGATTTCAAGGTGAAAGAGGTCTTCAGCTTTGCACAGGATGATTTGACCACAGAGGATGTCTTCATTCTAGACTGCTACACTGAAATCTATGTGTGGATAGGTTCCCAGTCAAATGTTCAATCCAAGCAACAAGCATTTCAATGTGGCCTGAAATATCTTGAAGGAGACGTTTTGGCCCAGGGACTATCGTTGGAGACCCCATTGTACATAGTTACAGAGAGATATGAACCAGAATTCTTTACTTGTTTTTTTGATTGGGATATATCAAAGTTGCATATGGATGGAAATTCATTTGAGAGAAAGCTTGCATTACTAAGGGGGGGAAGGAAGCTGGAGACACCTCCAAGAAATGCATGGAGGGCATATTCCTCAGAACTTCCCCGAAGTGGTTCAAGAAGCAAATCTGTAACTTCCAATGAAAAACCTAGTAATGCTTCTCCATTGCAGTCAACTTCTTTGAATGGTGGGGCATTTTCTAGCCCAGATTCAATTTCTGAGAAGCTTTTGTCAGTACAATCCAAGAACCAGAGCAGTCCTGTTGATTTCAATAAACTGGACACAGACTTTTCTGCTGAGAATGACAATCTTTCACTAATTGCTGAAACTGACAGTGATGATGCTGACATGTTGATCTATCCTTATGAACGCCTAAAAGTGACTTCAAATGAGCCAGTTTTAGATGTAGACGTGACCAAGCGCGAGGCGTACTTGTCCAAATTGGAGTTCCAGGATATGTTTAAGATGACAAAGAGAGCCTTCTACCAGTTACCAAAATGGAAGCAGGACAAATTAAAGCGCTCTGTCAATCTTTTCTAATGTCATTCATGAAACCGTGTTATGATAATTGAAACCGGGCGAACGAAGAGGCAGAACTTCCCGAGCACCTTCTACTTCGTCTGATGGTACGATCAAGGTGTCTCTAACTTGACTCGCTATGAAAGCTTGAAATGTTTAACTCTGAACTGATTATCGTGCTCTACTCTTACTGTAATACTCTTTACCGTCATAATTCTTTCTTGTTCATTGCTTCAGCTGTTCCGTAGAAACTATCTAGGACATCAGAGCTCCCTTGTTCAGAGATTGGATCTGTATTATCATACGTGTGTCTTCTTCATACACTATTCTTCTACACATTGCTGCTGTTTTTGTTAAATTTGGATGGAATTCTGATTTCGTACTATTTCTTCGCTTCAATTGAAAATTTTACCATTACCTTTTTCACCCGTGAGAATGGTTACCTTCTTTTTTTGCAATTTATTCCCGTTAATGAAAGTTAAAGTGTAGTGTAGTCATTTCAA

*S.maritima*558268

AAGACCCGTCTGGTCGGAAGTGTTCATCAGCCATTTCGTCCTTCACCCCTAAGCCCGTTCTCGCCATCCGCGTTCTCACTGCGCCGCTTCAAAGGCCGCCGGGCGGTGGTACGTTATGTCTTCGTCGTCCTCTACATCTTCCTCGTACGTGGTCGTCAAACAGGAGAACGAATCTGGCAGTCAAGCAGTTCCTGTAGTCAACATGGCCCCCATACCAGCCTATAAGCGTAGAAGAAATGAAAAACCTGGAAAAGGTCTACGTCATTTTGCCATGCGTGTATGTGAAAAAGTACGTAGTAAAATGTTGACTACTTATAATGAAGTAGCAGACGAACTTGTGGCTGAGTATCCAGAGGGTAGTAATGCTGAACAATATGACCAAAAAAATGTGAGAAGACGAGTGTATGATGCTTTAAATGTTTTAATGGCCATGAATATTATATCAAAAGAAAAGAAAGAAATCAAATGGATAGGATTACCAGTGACTTCATATCAGGAGTCTACTACACTAACTCGAGAACGAGAAGATTTAATGGCACGGCTCAATGAAAAACAAGCATTTTTGCATGAATTAATTGTTCAACAAGTCACATTTAAACAGCTCGTTGAAAAAAATAAAAAGTATGAACAGATTCATGGCCGGCCGTCTTCTGCGTCAGTTCTTAGTTTGCCATTCATTGCCATTAAAGCTGATCCTGGAACTGTAATTGATTGCAGTATTTCTCAAGACAAAAAAGAATATTTGTTTGGATTTAGTAACAGTTATGAAATTGTAGAAGATATGGATTTGTTAAAAAGCATGAACCTTTCATTAGGTCTCAATACAGGAACAAGTACTGCAGATCAATTAGAACAAGCAAAAACATGCGTACCAAAGAAATTACATGAATTTTTATCACGAATAGCCGTTGAATCTTCAGCTTTAGATGTAGTAGAAGAACAGCCAAAAAAAAAGTTCAAGTCTAGCTAAAGTGAGGTGACTTTTGCATTGATCAATGCCTAATAATTATCAGAAATTTAATTAAAAATCACTCTATTTTTACAAAATATTATCTTAAGTTATGATTAATGTAAGTTAGGCCACATTGCTTGTTGATGCAT

*S.maritima*40430

TCTCAGATTTTCACTAATATTAAAGTTCCATTTATTTACAAAAATAAATATCGATATGAATTGAAAATTATACTGTGGCTTAGGTGATTACCTAGTTGCATTAGCATTTAGATGACGAGGAACATTTACGAGAATTTAGAGAGTGGTGTTGTACAAAAATCACACAAAAACTATAAAAACAAAACCAACACAAGAGATGACCAATAGCTCGATACTAATTTGTACAAAATTTTAGTACCCAGTGCTATATATATATTTACTCTGAACCTCAAAGGGACTATTGACATTAAATATTGCAATACAAATGGTATACTGCTACTTCCCTTCGTGGCTTCATGTATTTACTGGTCAACAACACTGAAACGAGCGAGCGCATGCCAATGCTGCACGCTCCTCAAACATTCTCTTCTTTATTCACAACAACTTCCGCCGATGCATCATGCATACACAAATGAAGACATGCCATATTGGATGATGCGGGTGCAAGCTGCAGGATTACATTAACCACTCCAAAAAAATTGAGAGGCCAAATTTCCCCAAAGCTGCCAGACATAAATTCAATTCCTAACTTCTCTGAAAGTATGCTAGGTTTCATCAAGTGCAATATCAGCGGGATCTCCAATCCATGGTTTCCCTTCTCTCCATCTAAATTTTATCCTTAACTTACCGTTAGCATCAACACCTACCACCTCTCCCTTGCTTGCATGAGTTTCCATGCCCCATCCCCATCGTGGGGCCACAAGCCCTTCTCTAATCTTCACCTTGTCCCCAACCCTGAAAGCCCTAATTTTTTCCATTTCTGATGCCTTGCAAAGCCAATGTCTTTCCATGAAGCAGAATGCCACCCAAAGTTGTCCGTCCTCCATGCGATGTACTACACCAATACTTGAAGGATTTACTTCTCCCCATTGGTGAGTTGGAATTGTTACTGATTCTTTAACCTTAACCCAGTCTCCAATATAGATCTCCTCTTCTTCAACCACCTCGATTTCAGATGGGTCCAGCATCCAAGCTTTTGAACCAGCTGGTGTATAAATTCTAAGCTTTCCGTCTGCATCAATTGTTGTTATTGTCCCAATACTACCATGACTATGACCTGACCACCCAAACCTTGGCTGCTTGATCGATAATTTCACTCTAACCTTTTGTCCAAGCACTAACTTATCAGCTTTCTCAAGATGAGAGATGGGACCAGTCCATTTTTCTTGCTCTCCACAAAATCCAACAGAAACAGTCCCATCCCATTCATCACTGTCATATCCTAGTCCCTGTACAACCCCTATACTGCCAGGCCCGATAGATTTCCAATTTCCAGCACCATCCTTCAATCTTACCCACTCACCTACTTCAAACACTGCTTCAACTTCCAAGTCAGCAGGATCTCCTCTCCATAGCCCCAGCAAGCCAAAGAAAGCTACCCTTACTTCTCCATCAGCATGAACACTGGTAATAATGCCTCGGGAATTTGGGTTAGCACCTCTCCAGCCCCATCTTGGCTCTGCTAGTCCAGCACGGAATTTAATATACTGCCCAACCTTAAGATTAGGGATCTTCTCCATATCTGTATCATGAGCATGCCATTTTCCTTTACGGAAACAACAAGCCAGCTCTAAATAACCATTCTCTTGTACACTGTGGACAACTGCTACACCTTCCTTGCCTATACTGCTCCAATCATAGCTTGGTCTAGTTCCTAGGATAGGTTTAGATCGCACCCAATCACCCACTTCAAAACCAGATAGACATGCAGCATCTCCAGGAGAAACTCTCCACAGACTGGATCTCCCAGTTACTTTCACGTTTAATGTTCCATCCATGTCAATTCTCACAATTTTCCCAACAGTAGCTGGAGACTCATCAGACCACCCAAGCCGTGGCGCAGAAATAGATTGCATCACATGAATCTCTTGCCCAACTTCAAAAGGAGCTACCTTCTCCATATCAGTCACAGAACAGCAGAAGAGCTTGCTTCTAAAGCAAAAGGCAACACCCATGTCACCATCTTCCTCCAGGCTATTGATTATTCCAATGCTATTTTTGTTGACATCTTCCCATCCATATTTGGGAGAGGAAACAGAAGCTTTAACTCTAACCCAGTCCCTTACCTTAAAATCTTCCACTTTTTCCATATCAGCTGCATCAGCTTGCCAAGGAATTGGTCGGTTGGGTATCTCAATTATCAACAGCCCATCACTCTCTACCTCACTTATTCTTCCCACACTGTGATGAGTCTCACCACCCCAAGCATATCTTGGTTCTGCAACTGAGCGTTTTACACATACACGATCACCAATCCTAAAAGGCTCAACATGTTCAACCTCCTCTGGCTCACAATGCCACGGATTTGGAAGATAGCTTAGATCTAGGAACAGACTACTATCAGGTCTGATACAGTATACGACACCAATGCTGCCTGGTGTCACAGCACCTAAGCCATGTTTTGCTGTTGTAAGAGATGGACGGATACGAACCCAGTCACCAACTTTGTATTCTTCTACTCTTTCCATCTCTGCAGGATCAGCCTTCCATCCCCTGGATGCCCCTGGAAACCCAACCCGCAATATCCCATCATCATCAACACATAGTACAGTTCCTACGCTGTCACGTGAGTGGCCCCGCCATCCAAACCTTGGTTCCTTGACATCCTCTTTAAGTTTCACATGCTGTCCTCTGTCTAACGGAATTACTTTTATAACTTCATTCACCACAACACGAGCCTCTCCAGTGCAAAATGATATGACCAGATGATCCTTGTCAGGGATACTCTGTACAAAACCAACACTTTTTTGTTTTGCACCTTGCCAACCATACTTAGGAGCAGTTATGCTTCTCCTAAATTTTACCCAATCACCAACATCATATATCGTAGGAGAGAGTTGAACCCCCTTATCCAGAAGTGCCTCCATCAAATCTTCAGAAATCCATTCTCGAGGGAGTACCTCTAGAAAGTCTTTCAGGGTTTTACCACTGTGAGTCCTGAGATCAACAGCAGCATCCGGATACTTAAGCATATGAATAATCCATTGAAGGTTTTCACGAATCATCTTAGCTGCATCAGCTGCTATGTGGAAAGCATTATCACCTTCATCATCCTGAAAGTTGTAGTCAGCTCCAGCTGAGAGTAGCAGGCCTATACAAGAATTTGCACCTCGAGCCAGAGCCACATGAAGAGGTATTGTATTGTGCACATTTAGAATGTTGACATCAACTCCAGCATCCAAAATTATCTGCATCAGCTCCAGATCATCAGCCATAGCAGCTGTATGCAAAGCAGTTCTCTGTTGAGCATCTTGAGCAGTTGGGTCAGCTCCAGCAGTAAGCAATAACCGAACCAATTCTCTTCCCTCTTTTTCGTGGTCTTTCTTAGCAGCTGCAGCCATACACAAGGCTGTACCAACTGGGCTCAATATATTTATTGCAACAGCTATCTCCTCAATGGAAGCAACTTCCACCCACCTTCTCACAACAGCTACATTCCATGTTGCTACACAAAGGTGCAAAGGTGTCATGTTCTTTAAATTCGGAACACCCATTGATCTAGAACCCCCGTGTTCCAATATGATCAGAGCACAGTCAGTATATTTCTTTGCAACAGCTCTGTGCAGAACAGATTCACCTTCATCATCAACAGCATTAGGATCAGCGCCCGCTAATAATAGTTCACGCATGCAATCAGGTTGGCCATGGTATGCACAGACATGAGCTACAGAAGGGCCAGATCCTTCTCTCAATCTAGATCTCACATTAGCATTTCTATCTATAAGGGCACGAACACACTCCGGAGATCCAGCTGCCAAAGCAAAGACAAGTGGAGGATCTCCATCTTTATCAAGAATATCTACATCAGCTTCCTTGTACTCCAAAATAGCCTGAACAAGTTCAACACTCCCTCTTCTACAACCAAGATGGAGAGCAGTTTGCCCATCATTGTTCTGTGCCTCTAGCAGTGAAGACACTGCATTGCTACCAGCTCCAGATGCAGCCTTTGATAGAAAATTTCTTACACCATTCAAATCCCCTTCCGAAACTAGTCGATGTAGTTGATTGGGGTTATATGGATAGACCTCCAAGACAGATGTTGGGGACGGCTCAATCAAGTTTGTACTAGCACATGCCTCTAAATCATTGTCAGGACTTGCAGGAGGGCTTCGAGGTAGTGCTTGTAAGTGACGAAGAAATATGGCTAGCATTGCACTGAAAGTGGGACGTTTAGAAGGTTTGAACTGTAGGCACTCCCCAATCATCTTCCACAACTCCCTTGGTATCCCGACACCAACTACACTAGCATATTGAGGAGGCAATCTTCGAGCCTTAACAACAGTACGATAAATTTCCTCTGCACTCAAACCTGCCCAAGGAATGGTCCCTGTGCACATTTCCACCAAGGTACACCCAAAACTCCATGCATCTGACTCTGCAGATATCCCTATAGCCTCCTCCCAGAACAAGTTCAAGGATTTTCTGACAGGCTCCCATGCTTCCGGAGCTGTATAATGTGGACTGAGCATCGTGCAGTCCATACAAGAATGGATTATTGATGAATCCCCTTCTGCACGAGCTTTTCGGCATATTGGCTTTTTCAAAATGGACGGTAGTCCATAGTCAGAAACCACAGCATGACCAGCCGCATCCAGAAGAATATTAGATGGTTTCAAATTCATACAAACAACACCAGCGGCATGAAGTTCAGCTACACCTCGGGCAATATCTGCTCCATATCTGAGGATTTGCTCAAGTGTCAACCTCCCTTCGTTCCGCAACATAGCAGATTGAAGAGAGCCATAACATCTATCCATTACAAGACATAGATAATTATCAACCCCTCTTGAAACACCATGGAAGGTGCAAACATTTCTACACCACATAGCACCTTGCCTTAAACCCTCTAATTGAGTTTGCACCCACATCACATCCATTTCTTCCGTAATCGCCACCTTCCTCACGGCCACCTTATGTCGACACCGTCCCCTACACCGGACCCCTCCCGCCAGCGTAGCGGCCCACATTTCCACCCCTGACCTCTTCCCTTCCCCTATCCTCTGCAAAAACTTCAGCTCCCCATGATTAGCAAGCTCAATCACACCGCCAACCGAGCACCCGCCCGAGCTCGACGCACTCGAACTCCGTCTCCGATCCTCCTCAAACTCCTCAAACTCATCATTGTCAACGTCACCATCACCGTCGCTGACGTCATCGTCGGAGGACGAGTTTCCGGTGGCGGTAACAGCAAGGAGAGCGAAATTCTTCCGAAGAGCCGATACGGAGTTTCCCACTAGGGTTACATGGCGGCAACGAGGGCACGAGAGTGAGTTGTTCAACGAAGACGAAAACATCCTCGAAAGACACTCCTTGCAGAATCCATGGCCGCAATGGAGCAACAATGGAGCTCGCTCCTCATCGTTGTACCGCGTGTGACACACTGAACAACACGGCACCTTCATCTTCTTCTTCGAATCGAACAAACAACAAACTCCACAACTAAACAATCGTAATATCGAAGATTCCACTTTCACAACAATAAAACCACTCGAATCAAAATCGAAGAGAGAGATCTAGAGTGAGGAATTCGAGGTTTCAAGAAGTAGATCCATGGCGGAAAAATGGAGGAAAATAAATTAAAAATGAAAAATGAAAAAGAAAAAGAAAAAATAAAAAGAAAGGAAAAACAATAAAGTGAGGAGAGAGAAAATAGGGATTTGGTGGTATTTTTACTTACTTTGGTGTTGTTGTGTGATCAAGTACTTGAATTGAATTGATCGAAGTGGTAGAGAAACAGAAAATTAACAAAGTTTAG

*S.maritima*333049

TTTGGGATACTGCTGGCCAAGAACGTTTTAGGACAATCACGACAGCTTATTACCGAGGTGCCATGGGCATTATGTTAGTATATGATGTCACCAATGAAAAATCATTCGACAATATCAAAAATTGGATACGTAATATAGAGGAAAATGCATCAGCTGATGTAGAAAAAATGCTGCTAGGGAACAAATGTGAACTTAATTCAGGGCGTCAGGTATCAAAAGAACGTGGTGAACAATTAGCAGTAGAGTATGGTATAAAATTCATGGAGACATCAGCAAAGGCTAGTGTCAATGTTGAAGAGGCTTTTCACACTCTGGCCAGAGACATCAAGACAAAAACAGAAAAGAAACTGGAAGCATCCAATCCTCCAAAAGGTGGTGTACAGTCTAGTGGTGGTCACCATTTAAGAGATAATAATGGAACAAAGAAACTTGGTCAAGGTGGTGGAAGTGCAGCTGCACTCAATTGGTTGTCATCAAGATGTACAGTGCTCTGACCACGATTAGCATATTATCATGGTTCAATTAAAAAACAACAAATGTACACCTTGAATACCGTTGTCAACATGACCGTTAATTAAAAAAATTATTATATTATCTTTTTCTAATACTAATAATTGTTTTTAATGGAAACTGAATCTCTCCGGTATTATGTATTTCTTAATTATATGAAAATACGAACTTCTTTCATTTTTTT

*S.maritima*1466

AATTTTATTGACTAAAGTGATCAAAACAGTGAGAAAATAAAAGCCTACATTAATTAATTGGTTTTACTGAGAAGGAAGAAAGAAATACAGTACTTTAATTAATAGAGGAAGTGTTTATTAATTATATCCAACAACACTTTAATGGTTCAGTTGTACCATAAAAATCTTGCACTGAATCATCATCTTCACTGCCAAGAAAGTGAAACATCTTTGTGTCTAGTGAGTAAAACATCACACTCTTCCCTCGAAGCTTCGGGAAGTAGATTCTGTTTCTTATCTTTGTATCTCCTGTAAAGAAACTCAAAGTTGAACCTCGACTAATGAACAAACTATGATATCCTAAGTTAACCATTTTAACCCAACACACATTCAACCAGTCAAATTGTAAAACTTGAACCTGTTTTCCCATCTTATCAGCATAAACCACACATATTTCCCCTGCACACTCTACCAGATGCAGATTATAACCAATCTCACTGTCTGCAAACTCATCAACTATAGTCAGACGGCTGTTGTAAACAAACCATGTTGGTTCCCAATCTTTTACTATTTCAAATTCTCCAAGACATCCATAAACATCCACAAAATAAAATGCTCCTTCATAAAATATTGGAGGACTAAATAGAGTACCTGAAAATACATTGGTTTCGAATTCAGCATAATACTCCTGCCAGGTTTTATCCCCTGGTTGAAGGTAGTAGATGACCAGCATATCAGCGGGAGTATTGGCCATTACAACTGTCGAACAATCAGAGGAAGTAGGATCGGTTGAGAATCCAACACTCATAAAATCTAATAATTCATCAAAAGCAGGTAAATCACCCCAGAACTCGGTATACAGATTATAGAACTTCAATGATCGTGAGGAATGTGGACCAACCAAGGCAAGCAACCATCCATTCTTGGAAAAATGTATCGAAAAAGGATCTGTAAATAGTCGCGGTAGGCTTCTAGAACATGTGTTATATTGACATGGATCGATTAATTCACATGATCTATCGTTGTTCTTACTAAACATGAACAAGGGGAGGACATTGTTGGTTCCCCATTGTGGTGCAGGGCATACTGATAGGAGTGTTTTGCAACTAGCTCGGAATTTCCAATATTCAAACAGATGCATGCACTTTGAAATCAATTCTACCATGTCTATAGGAAGAACATCGAGTATGCTATCTTCAATACTTACTTTGTGGTCCAACAACTCAAGGCCCCCACTTTTCATGAAGTCATCCGATTGATTAGAAACATCATCTCCTATCTTGGTTCTTTTTTCTTTCAAGTCTTCTAATGACAATAATCTTAGGAAAAACGAACAGATAGACCATCATATCAATTAGTAAAACATACAAAAA

*S.maritima*233789

GTTCAACAGCTTGTAAGGGGGAAAAAATACTTGCTTGTCCTTGATGATGTCTGGGATCAAAACCAAACCATATGGACTTCTTTACAAAATTCGTTGCGAAGAATTGGAGGTGCCACGGGAAGCATGATTTTAGTTACAACCCGCAACAAAGACATTGTAAAAACAGCAGAAAGCCGATATATGCATCAACTAACTGGATTAACTGAAGAAGAAAGCTGGGCTTTCTTTGTGAAGAAGGCATTTCCAAAAGGCACGGATACATATGATCAAAGACTAGAGGAGATTGGGAAAGGGATAGTGAAAAAATGCAAGGGATTGCCATTAGCAATAAATGCTATAGGAGGCTTACTACGAACAAAAGAAGATCCAAGTGAGTGGGAAAATATTGGAAGCAGCACATTGTGGAATCTTCCCCAAGAGAACAATGATATACTACCTTCATTGTTACTGAGTTTCAACAATTTGTCTTCTCCTTCCTTAAAACAATGTTTTGCTTATTGTGCTATTTACCCAAAGGAAGCACTCATTGATCGAGAAGATTTAATTAATCTGTGGAATGCTCAAGGTTTCCTTCATTCCCAAGAAGAAGGAAGAAATTTGACACCTGAGGAACTTGGAGGAAAATATGTAGATATTTTGTTAAACAATTCACTCTTACAGGCAGAGACACGGCGTTCTTGCTCAGGAGATATCACAGAATTTCGAATGCATTATTTGGTGCATGATATAGCTCTTTACACGTCTAGACATGATTGGTTGATTTGGAAGGGGGCAGACAAGAACAACAATCCTAAGGGTGGTCTACACCTAGCCTTATTTCCTGAAAATGAAGACAGGATCTCAGAATCTCCAGTAGAAAGGATGGCATGGTTGCGGACACTACATTCTTCAATGAACTTACCTAAGCATTTTTTGGTGCATACCAAAAACTTGCGTGTCCTAAAATTAGCTTCAACTGGCCTGAAGAATGTGCCTCGTGCTATTGGTCGGCTCAATCATTTAAGGTATGTAGACCTATCACATAATCCAATAGTCACACTGCCTGAGTCCATTACAAGCCTTTACCATCTGCAAACATTCAGACTTTTTGGTTACAAGCTCATGGAGTTACCTCAAAAGTTGTATAGGTTAGTCAACTTAAGACACCTACATCTTACCTCCCGCTCATGGTGCCTACCTAGAGGAATTGAGCAGTTAAGTGCAATCCAGACATTACCAATGCTTGAGTTGAATGAAGGCTCTGGGTGGGAAATTGGTGAATTGGGAACTCTGGGTGACATTAAAGGCCTCTTATCTATATCTGGACTAGAACATGTGAAGGGCCGGGGAGAAGCTGAGAAAGCTGGCATTTGCAAAAAAGTCGGCATTTCAGAGATGCAACTAATTTGGGGCTGGGGAAGGTCTAGCAATCACCTAGATGTGCTAGATGCTCTTCAACCTCCCCCAAATCTTAAATTGCTCATGATAACAGGTTATGATGGTTCAAATTTTCCATCTTGGATGGTGAGTATGATGAATTTCCGTGAAGTTGGTAGACCAAGACCATTTTACAATCTGGTGCATATTGAATTACTATATTGCAGGAGTTGCCAACAACTCCCTGCATTCGGCCATCTACCATGCCTTAGTGATTTGTCTATGTACTCTATGTGGTCTGTCACATCTATGGGGGATGAATTTTATCACACTACTATCGATGTTGAGGAGCAGGCTCCAACTGTCTTCCCAGCACTGAGAAAACTTAATATATTCAGCTTTGAAAACTTAACAAAATGGGAACCACCAACAAGTCTAGAGTTGGGAGTAACTACACCAATCACAGCTGCATTTCCCTTGCTTGAAATGTTAAGAATTGAAGATTGTGGAAAACTAGTCCAAACTCCAACCACCTTCCCTTCTCTCAAGCATTTGCGGGTTGACAAGAGTCTCGGGGGCCTAGCATTGTATAATGTTATAAGTAATAGCAGTAATGCACTTACCTCAGTTAACATTAATGAGGCTCCAGAGCTATCTAGTCTACCTGACAAGCTGATTGATTGCACAAGTTTAGAGGAATTGACCTTACGGTGTTGTCCTGCTCTACAATCTTTGCCCAAGGGACTGGGAACTCAACTTACTTCACTGCTTCGATTGTCCATTGTTGAATGTGCAGTATTGAGAGAGATCCCAGCCAGCATGGGGGATTGCCTCTCTTTAAAGAAACTGTGCATACGTGACTGTCATATACTAGAAGGTGTACCAGATTTGAGTCAGTTAATTCATATTGAAGAAATCGAGATAAATGGATGCAAGAAACTTACTAACCTGCCAAGGGGCATTCAACTTCTTCCCCACCTCAACTCTTTGTCCCTTGGAGGGTTTAAGTCCCTAGACATAGCATCCACCAGCTCAGTGACATCTCAACTTCCATTCCCAGCACTGAAACTGTTGAGGATTATCAACGATGACTGTGCAGAGCAAGTACCAGATTGGATAGGGAATCTTACATCGCTGCAGAGACTGGAACTCAAGTGGTGCAGGAATTTGAGACATTTGCCATCACAAGATGTCATCCTGAAGATGACCAGATTGAGGGCATTGATAATAAAATCCTGCCCACTTTTGAAAGAAAGTTTTGCGACAGAAGATGGACCAGAATGGTACAAAATCTCCCACATCCGCCTCATTAGAGTCAATTCAAAGACAATTCAAGAATAATCTTCATAAGTTCGATCATCCTAATTCCTTCTTTTTCATATTTACTTCTTTTTCATAGCTGCCACAAAAAGCAAGGTTATTTATTAACAATAAATAATTTTTTAAAAAATAATGATTCCTTCGGAAAACATGCATAGAGCTATACAACACTACATGATATTAGACATACATTATGTCCACCAAGTAAAG

*S.maritima*26161

GTTTACCTTTTTAACTCAACTCTTCAATAAAGTTAACATTTTGGTGCTTTAGAATGCACCAAGGCCACAAAGAACTTTCACTACCAAAAATCCAAAAAACACACGTTATTGTGTGGGTCATAAGCAATCACTCATCAACTTGTTCCAAGCCATCAAAAATCAAAATCCAATACATACAAAATACCCTTGCACAAAGCAATACAACTATCAAAAAGAGTAGCAACCCTTTTTTCCTCTCTTATCCACTCCAACCATGGCTACCACATCCACCACCACCACTTCCCTCTTCTACTCCCCTTGTTCCTCCTCTTCCATGAAACACTCTCCTTCACCTCCCTCCCACCTCTCTTTCCCAACCCCTCGACGTCGTACCCTCCCACCAATCACAGCCTCTGGCCCCGACATCATAGGCGATTTCGGGGCCAGGGATCCATTCCCAGCTGAACTAGAGAGCCAATTCGGCGAAAAAGTCCTAGGAAACATGAGCACAGAACACAAGATACTCATTCCACAAGCATCAGCATTGTCACTCTCACAACAAACATGTGTCCCTGTTTCACCCAATGATCCTCCTTTGTCTAAGGCTGATGCTAAGCAACTCCTGTTTAAGGTACTCGGATGGCGGTTACTCGAAGACGAGACCGAGACCGAAGGCGGAACAAAGACAGTCCTTAAGCTGCATTGCTTGTGGAAGTTGAAGGACTCAGAGGCAGGTGCTGAGTTGCAAAAAAGACTGTCAAATGTTGCACAAAGTGCTGGTCATCATAATGCACTTGTATTGAAACAAGATAGTAATATTGTAACTGCTCAATTATGGACTCAACAAATTGGTGGATTAAGTATTAATGATTTTATTGTTGCTGCTAAAATTGATGAGGTTAAGATTTCTGATCTTCTTCCTAAGAAGAGATTTTGGGCTTAATTAATTACTAGTGTTTTTTTTTTTGTTTTTGTTTTTGATGGTGTAGTAGAAAAATGGTGTTGTGGAATTGATGTTTGTTATCATTTTTATCACATATTTTCTCAACAATACATAGTGTGATAGTGGAGTTTGAGTTTATCTTCAATTTTACAGAGTTCTTTGGATTGCACTATCCTTTCCATATCTGCAAATATTGGCCACATATCCTTTCTATATCTATGTTGAAATAGAGTAGTTGTGTTTTTTTTTTTAATTCCCAC

*S.maritima*41508

CTAGAGTCTACAAAGACCAAACTAATGATGTGCATCTTCGAGTCTTTGCACAAACCCCAAACAAAAACACGAGTCATCTTCTCCGCTGTAAAGCTCCCTTTTTTCTCTGTCACGAGAATGTTTATATTTGACTAACCACTTCTTCATCCATTCATGGCTTCTCTTCCTCTGTTTCTCGTCAACTCTTCATCACCCATTTTCCATGTAACTTCAAATCATTCAAAAATCCAATCTTTTAACTCAAAACCCCATGTTCCATTTTCATGAATTTTCCCCATTATCAAAAACCCCAGATTCCATCTATGTGTTTCTCTCTTTTCAGATCATCAAAACCTAAGAAACCGGACTCTAATTCCAAAAGAAAATACCCAAATTCAAATACCCCAGTTGTAATCTCTAGCTCAAGTAGAAGCCAAACTAATAGCAGAAGCAATGATTTCAACACTTTTGATAACTCAAAATCATCTTCAATTTCAAGCAGAGCTTCTTTAGCAAGTCTCAAAGATTCACTTCCTGAAAATCCTCATATTTATGATCTATCTGAAATTCGCTCTGCTACTAAGAATTTCATGTTAAAACCCACTTCTTCATCTTCATCTTCTTCTTCAGCTTCATGGAAATGTTTGATTGATAAGAAACAATCAATGGTTACTCAACGAAAGCTCCGACGACCCATTGACAATTCGGAGCTCCGAAAGATACTTTCGATTCTATGTAAGTCTCATCATGCTAGTTTGGTGAAGCTTCATGGTGCTTCAGTTAGTGGGAATTATATTTATCTTGTTTATGAGTTTATTAATGGTGCTAGTTTGAGTGATTGTTTGAGGAACCCTAGAAACCCTAATTTTACTGTTTTGTCTAATTGGCTTTCTAGGGTTCATATTGTTGCTGGGATTGCTGATGGGTTGGATTATATGCATAATTGTGCTGGTTTTAAAAATGGGTTTATTCATAATCATATAAAAAGTAGCTCAATTATTGTGTGTGATGAACCTGCTTTAGTTGCTAAAATTTGTCATTTTGGAACTGCTCAATTATGTGGGGAAGTTCCAGAGGCAATCAAAGAGGAAGATGATGAGGGTTTGGATAATTCGGTTCTAAAACGGGTGGGTAGTGCGGTTGGGAAGTTTGTAGGGACTAGAGGGTATATGTCGCCCGAGTTTCAGACAACCGGCATTCCCACATTGAAATCGGATGTGTTTGCTTTCGGGGTGGTGATCTTAGAGTCGTTGTCAGGGGAAGAGCCTGTGAAGTTGAAGACTGATTCAGAAAGGGGCGTGTTTCATAAGGTTTCGTTGATTGAAACAGCTAGAGAAGCTGTGGAGGGTGGGACTGGAAAGCTGAGGCAATGGGTTGATAGAAGGTTGAAAGATTCGTATCCGGTTGAGGTTGCTGAGAGGTTGACTCGGGTGGCGTTAGAGTGTGTTCAGGAAGATCCTGGCAAAAGGCCAGATATGGGCAGAGTTGCTGGTTGGATATCTAAGTTGTTTTTGGAGTCCAAGACTTGGGCTGAGAGTATTGGTTTTCCTGATGAAATCACTGTTTCATTGGCACCCAGATGATACTTGAGGTTTTAATGTGACACTGACATTCAGAATGTTCTTCTGTAAGTAGCTGACTTTCAGAATGTTTTGTTTTGTAATTAGCTGATTCTTTGAGGTGTGCTATCAGTTTATTGTAGGCTGTAGAGTATAGTGTAGTAAAAAACGTACAATAGCATACAAAACATAGTATGGTGCTTGAAGCCATGTCTCTTCTTATTGAGATCCTCAAGACACTAAGACAGCATGATGTTTCCTCATAAGCTCCACCTGCATCCCATCTCTCGTCAATAGTGTGAAGCTACTACCAAGTACCAAGGCAGAATGTTGCATTTCTCGGAAATGGACTTAAGTTAGCCCATCCTCGCATGCTTAAGACAGCATTCAAGCCATGTTTGTTTCTGCTAAGCAATGTGAGGTTATCATGGATTAGCTTATGACGCTTTCTTCATCAAGGTCTCTCATTTATGCCAAATGTTGATATGTGGGTGAAGTCCAGTTTGTGTTTGAGAATGCACATGAGTTGGATAATGTTTCATGACATGTGATGGTAACATTGTTGTGTGCTTGGAATGGTCCCAATTCGGAATTCATGTCTGGACAGTTTTCATCGAAGGTTATCTCTGCAGATTTTCAAATGAGGTGTGGGAGGTGGATCCATAGGTTCAGTCATGTACATGTTGGTGAAGGCGAACTAATTGCTTATCATGTTATGCGAGGCAGCAAAGGGATTTAGATGATGCTGCCAGAATCTTCAACGGATGCTAGATGTGAACCTATTAGTGTATATGATAAGCGGGATTTATAATTCAGGCTTCAATGAGTATCTCGTAGAAGAAAGCTTTCGCCTCACCATGAACGAGGACACCGGACACAGGTTAAACACATTGCTGCTCTGAACACCGGACACAGGGTAAACACATTACTGGCGAACAGCGGCACCTTACAATCTGGGATTTGATGGATCATCCATGAACAAGATGTTTTCTAAGGTTTGAATACTGGGTTTATTACATGCATACAGCATACTTATCAGTTATGTCTACTGCATTTTCAGATAATTAAAAATAATACCGTATTCATGTGCAAATGATTTGTTTGTGATTGTATCACTGTTAATCTGCCAATTGCAGAACACCTTACCTAAAAGAAATGTAAATTGTAAAGAGTTTAACTCATGTAATGAGATATGATTTGCTTGCGCACTG

*S.maritima*41732

ACCTTCATCATCATCATCATCATTCGCTTCGTTCTTCAGTTTTTGCTTCTCACTGTAATTCAGCCATGCCTCGCAACAAGGGAAAGGGTGGTAAACGTAAGGAGGACAAGTGGACATTGAAGTCAACTTCTGGTGGATCATCAATGATGGAAAGTGCATCAACAAATGAGACAGAAGCCATTATTGGTGCATTTAGTGGTTTAAATATATCTCATCAGAGTATGCAAAGTAAGAGTGCAATTGCACCAGGACAAAAGGTTGTTTGGAAGCCTAAATCATATGGAACTTTGAGTGGTGTTAAAGGAGACGAAGTTGAAGCTCAAAGTAAGGCAAAGGAAGGTGAATTTTCTTCAAGTGTAAAGAACAGTTCCCAGATTAGTACATTATTCAACGGGAAACTGTTGGAGAACTTTACTGTTGACAACTCTACTTATTCACATGCACAAATAAGAGCTACCTTTTATCCGAAATTTGAAAATGAAAAATCTGATCAAGAGATTAGAACGAGGATGATTGAAATGATTTCCAATGGCCTTGCCACCTTAGAGGTATCGCTCAAACATTCTGGATCTCTCTTTATGTACGCAGGACATGAAGGTGGAGCATATGCAAAGAACAGCTTTGGAAATATATACACTGCAGTTGGTGTTTTTGTTCTTGGAAAGATGTTCCGTGAGGCTTGGGGAACCCAAGCAAGTAAAAAGCAGGCAGAATTTAATGGCTTTCTTGAGAGAAATCGCATGTGCATATCAATGGAGTTGGTAACTGCTGTGTTGGGTGACCATGGTCAGCGTCCTCGTGACGATTATGTTGTTGTGACTGCTGTCACAGAATTAGGAGCTGGTAAACCCAAGTTCTATTCTACTGCTGAAGTAATTGCTTTTTGTCGGAAATGGCGGCTACCAACAAATCATGTCTGGTTGTTCTCAACAAGGAAGTCAGTGACATCGTTTTTTGCAGCATTTGATGCTTTATGTGAAGAAGGAACAGCAACTCCAGTATGCAAAGCATTGGATGAAGTCGCAGATATCTCTATCCCAGGATCAAAAGATCACGTTAAGGTGCAAGGTGAAATTCTGGAAGGTCTAGTTGCTCGTATTGTAAGCCCAGAAAGCTCAAAACGGTTGCAGGAAGTTTTGAGGGACTGCTCTCCTTCAGCAGTAGAAGAGGCTGGGCTCCACTTGGGTCCAACACTACGGGAGATTTGTGCTGCAAATCGGTCTGATGAGAAGCAGCAAATTAGAGCTCTTCTAGATGAAATTGGTTCCTCCTTTTGCCCCAGTTTTGTAGATTGGTTGGGAAAAGAGGCAGATGATGTTAGTTCCAGGACTGCTGATAGATCTATTGTCACTAAGTTTTTGCAAGCTCATCCGGCAGATTATTCAACTAAAAAGTTGCAGGAGATGATTCGTTTGATGAAAGAAAAGCGGTTGCCAGCTGCCTTCAAAAGTTACTTTAATTTTCACAAAGTGGATGCCATGTCTAAGGACAATATACATTTTAAGATGGTCATACATGTCCACAGTGATTCTGCGTTTAGGCGATACCAGAAAGAAATGAGGCACAATCCAGCTTTATGGCCATTATATCGAGGATTTTTTGTTGACATAAATTTATTTAAGGGAAACAAGCAAAACACTGATGAAGCTGGGAAAATCTTGAGTGATCTAAACAATTTAAATGTTAGTGATGATGCATCTGGAGGTGATTCACTTGCTGATGAGGATGCTAACTTAATGGTCAAATTGAAATTCCTCACTTACAAGCTACGAACTTTTTTGATCCGGAATGGCTTATCAATTCTGTTCAAAGATGGCCCTGCTGCTTATAAGGCATACTATTTGAGACAGATGAAGATTTGGGGCACCTCTGCAGCAAAGCAAAAAGAATTGAGCAAGCTGCTAGATGAATGGGCTGTTTACATAAGACGAAAGTGTGGCAACAAACAGCTGAGGTCTGATGTTTACCTCAGTGAAGCAGAGCCTTTCCTTGAACAGTATGCGAAACGGTCCCCTGCAAATCAAGCACTCATTGGTGCAGCTGGAAATTTAGTGAGATCTGAAGATTTCTTGGCTATTGTTGGAGCCATTGATGAAGAGGGTGACCTTGAGAAAGAGCGTGAGATAGAATCTACCCCATCATCTTTTAGCACTAAAGCAGCTGGGAGAAAAGAAGGCCTAATTGTGTTCTTTCCAGGAATACCGGGTTGTGCTAAGTCTGCACTTTGTAGGGAACTGCTGAATGCTTCAAGTGCTTTTGGAGATGATCGTCATGTCCAAAGTTTGATGGGTGATCTCAATAAAAAACGATACTGGCAGAAAGTTGCCGATCTGTGTAAAAGCAAGTCTGACTGCATAATGCTTGCTGACAAAAATGCACCAAATGAGGAAGTATGGAAACAGATTGAAGGCATGAGTCGATTTAGTGGTGCAGCTGCTGTTCCAGTTGTACCTGATTCTGAAGGCTCTGATTCAAATCCTTTTTCTTTGGATGCGTTATCTGTTTTTATGCTGCGTGTGATTCAGCGAGTTAATCATCCGGGGAATCTTGACAAGGCATCGCCAAATGCAGGCTATGTACTTCTTATGTTTTACCACCTGTATGAGGGAAAGAGCCGCAGAGAGTTTGAAGCCGAGTTGATTGAACGTTTTGGCTCAGTTGTGAAAATGCCTTTGCTCAAACCTGAAAGGGCTAGTTTACCTTTTCCTGTGAAATCTATGTTGGAGGAAGGGATAAATCTATACAAACTACATTCAAACCGGCATGGAAGATTGGAGCCAACTAAAGGGTCATATGCGAATGACTGGGTGAAGTGGGAGAAGCAGATGCGAGCGACTTTAATGAGTCATTTTGAGTATCTCAACTCGATTCAGGTTCCATTTGAGAGTGCTGTTAACAGTGTGTTAGAGCAGCTTAAAACAATCACGAAAGGCGACTATATCGCTCCCAGTACCGAAAAGAAGCGGCTTGGAGCAATTGTTTATGCTGCTGTCAGTTTGCCTATTACTGAAATTCGTCATGTCGTCAATGATATTGCTGTCAACAACTCCAAGGTGGAAGTTTTCTTCAATGACAAGAATTTGGCAGACACCCTTAGGAAGGCTCATGTCACTCTTGCTCATAAGAGAAGTCATGGTGTCATAGCAGTGGCTAACTATGGTCAATTCCTAAACCGTAAGGTTGATGTTGAATTGACTGCTTTGTTGCTATCTGATAACTTGGCTGCGTTCGAAGCACGTCTTGGCTCAGTTGATGGTGAAACGATCAATTCCAAGAACGAATGGCCTCATGTTACGTTGTGGACAGCACCAGGAGTTCCAGCCAAAGAAGCAAGTACCTTGCCCAGGTTGGTGTCAGAAGGGAAAGCATCTCGTTTTGAAATTGATCCACCTGCTGTTATAGACGGTGAAGTGGAATTCTTCTAGTTTATCTCCTTTTATATTAGTTTGATCAAAGAATTTGTTCTTTGAGCTTTTGCCTTGTGAATAGTTTTCTTCCTGCGGCGTACACATGTAGTGTGAATGTTGAGGGCTAGCTGAAATTTTTGGTAGGAATATGAAAGGTTTTGTATTTAGTTGAATCAATGGAATAAAGAAATTAAAGAATCTCTGGCCTGCTTTGGTACTTCCATGGTTCAAGAGATAGGGCGAGCCGAAGAAGCAGGTAAAAACTTTTAGAAAAGGGCAACAATGCCTGTAATTGAAGATAGATGGGTAGTTTGGCTAAAGCAACCATCTGTTTAAAACTTGTATTAATGTTGCAATTGACTCAAACGCAGCCAAGTCAAGGCACGTCTTACGTTCAAGTCAAGGCATATTGCTCCCAAAAACTATAGCAAGTTCATTGGATTTTCTTTCGAATCAACAATACTAGCTTTAGAAGCCATGTTCAGAGTACAAAAATGTGAATGCAACATTTCACAGTAGATGAGGTGTATAGGAATATAGGATTATGGATTTTAGTTGGTTTATGGCG

*S.maritima*43555

CGGCCATGATCCATAGATCTCAACTGCTCGAGCATAAATTCGACCACAAAAGGATCAGAGAGGTCCATATACACTTCAAAATCGAGCCAAAGCCACCATAACCAGAGTAAACTCCATGGCTTATGGTCCTCGTCAATCTATTTGTACTTCCACTTCCGAGTTCTACCAAACATAGTCTAAAAGACTCCCAACTTTTCACTAACTACCCTCGTTATCTGAATATACAAGGGATATGAAGAAGTAACAAGTTACTCAGATGACGGGGATATATACCGGATTGCCATCACTCACTGAAATCACCCGGAATCTGATATTCGTGCACTGTATGCAAACCAGTTAAAACTAAAGCAGATTCTTCAAATGAATAAACAGAAATGAGATAGAAAATGTTGATAAAATCCCTAACTTCCAACCAAAAACAGGATATTTGGTAAGCTCAAAGTGACATCAAAACAGAAGCTGTAAGAGCGCGACTGAGATCTTATTGACACCTGAAATTACATCCCAGCATTTCGCTAATGTTTCCCTATTTCCAAGTTCCAAGACTCCCAACGTTTGTATGTTTCCATAGAATCAGTCCGTCCTTAACATGAAACTACAGAATCAGATGTCATTATGCCAACACCGTTAATGATTAATCTGAGCACGTTCAGCCTCAGTAAATCCTTCTAACTATTGATAAAAAAAAATAATGGAAAACCAAATAAGGGAAATGTGATTTGGTGATGAGGTTCCAAAAATCTAATCCTAGTTGAATAATTTTAAAGCTAGGGGAAAACCTCCTGCACTTCACCATCAGCTAGAGCAACACCGAAATTTGCTTGAGCATCAGCATCAGAATTAAGTTCCCTTAAAACATGAGAGATCTTGAAAGAAGAGAACTGTTTCTTCAGTTTCGCCGCCTCTGCATGCAAGTTCGACATGTTTTCATTCTTGACTTTCCATAGACCCTGAAGCTGCATGCAGACAAGTTTGCTGTCACCCTGCGCTTCAATACCTGTATAACCCATCTCAAGAGCTTTCTTTAATCCTAAAATAATAGCTCGATACTCAGCAGCATTGCAGGTTGCAGTTCCCAACCCTTGACGTATTCTACAAATCAAGTTTCCACTAAGGGTTCGAAGCACCACTCCAGCACCAGCTTTTCCAGGATTTCCTTTTGAAGCACCATCAAAATGAAGAATACAGGTTTGAGTATCGGAGGGCACGTGGTCAGACTTCTGAAGCTTGCTCAATGGATCAGAAGTGGTTGAAAGCAACCCCACATCATCCTCATGAGGCCTCTTCTGTGGTAAATCTATGCTGGATGTTTCACCTGTGGCAGATGCTGGTTCCTAAAGAGCAATGGAAACACAT

*S.maritima*44432

CTTCCCTCAACCAACGCAGAAGTGAAACCTAACACAAGGAAAAATAAAGCAAACTTTACTCATTCAACGAAGGCGGAAGAGAAGCCAGAAGGTCATCAAAAGAAATGGAGAAAATTACAAAGAGAAGAATCTGATGCATTACAAAGGGGAGCTCTTGATAAATTTTTTAAGAGAAGAGTAGGGGAGCCTTCGGATGAATTGGATCTTGACAATAGAGGAAGAAGAGTAAGCTCAGGCGATGATTCATTCAGCAAGAGGATGTCATGAATTCCAGCAATAACAATGGCGACGACAACAACATAGCAGTGATGGGTAGGAAGGAGAAAAGGAAGGCGATGAAGAAGATGAAAAGGAAGCAATTAAGGAAGGAGATAGCGATCAAGGATAAACAAGAGGAAGAAGCTAGATTAAATGATCCTCAAGAGCAAATTAAAATGGCTCGGTTGCAGCAAGAGGAGGCGGAGAGAATGGAGAAAGAGCGTATCGAGTTTGAGGAGAGAGAAAAACAGTTTCTTCAAGCTTTGGAGCTCAAGAAGTTGCAGGAGGAAGAACAACTCAAGCAATGCAAAGATTCTCTCAATATAATGGTTAATGAAGATAATGCTGAAGAGAAAAATGAAGATGATGGTTGGGAATATGTAGAAGAAGGGAGGGCAGAAATAATTTGGAAAGGAAATGAAATAATCGTTAAGAAGAAAAAGGTCAAAGTGCCCAAGAGATCTCTGGAAATTCAAAAAGAAAACAAGGAAAGTGAAAGACCAATCTCAAACCCTCTTGCCCCCCAATCTGAAGCCTATGAAAATCATAGGAACGCACAAGATATTCTACAAAGTGTTGCACAACAAGTTCCTAATTTTGGAACTGGCAGGATAAAGAACATTGCCCTTTCCATCTCAAAACTGGGGCATGTCGCTTTGGAACACGCTGTAGCAGAGTTCACTTCTATCCAGATAAAGCTTGTACATTACTGCTGAAAAATATGTACCATGGGCCAGGCCTTGCATGGGAGCAGGATGAAGGGCTTGAGGTCTGTTATTGAAGCCATTGGGTGGTTCCTCACCTGGGGAATTCTATAGGTTCTCAAGATGGATAACTGAAGCTTCAGCACATTACTATCATGATGGTTTTCTTTCCATTGCTTTCAGCCACACGTTGCATTGTCAGAACTTTTAAATTATATCTTAGAAAGTTGAAGTATACGGATGAGGAGGCTGAACAGTCTTTTGAAGACTTCTACGAGGATGTTCACACAGAATTTCTGAAATTTGGAGAAATTGTGAACTTCAAGGTGTGCAGAAATGGATCTTCCCATTTACGGGGTAATGTTTATGTTCATTATAAGTCACTGGAATCAGCTGTGTTCGCATACCAGTCAATTAATGGTCGCTATTTTGCTGGCAAGCAGCCAGCTCATAATCAAATGTTAGACCAAAGCTGTCACAACTGAATTGGTTGAAGGATTCTATTGCTAGAAAAATTTCAAAGCGTCTTCTACATCTTTTGATAATAGAGCATAAATTAGGGGCAAGCTCCCTATTCTCAATGTTGAAATACACAGTTAACCTGTGAATTTGTTAATGTGACAAGATGGAGGGTTGCTATCTGTGGGGAGTATATGAAGTCAAAGCTCAAGACTTGTTCTCGGGGATCAGCTTGCAATTTTATTCATTGTTTCCGAAATCCTTGTGGAGACTATGAATGGGCTGATTGGGACAAACCTGCCCCACGGTGCTGGCTGAAAAGTATGGCTGCTTTATTTGGATACAGTGGAAACAGATTCACAGATGGCGATAGTCCTGGCTATCACTCAAGAAGTTCAAGGTTCAGGGCTTTAGGCAGCACTCATGAAGATGATGGTCACAGGAGACGGAGCCTCTGGAGGCACAAAGATGATGACAGAAAAGCCAACAATTGTGCTGAAGTGATACAAGGAAGAAATGACATGCATCAAGATCATCATTTTACCAAGCACCGAAAAACTGATGATGGTGATAGATCGAATGGGAGCACTGGTGGTCAGAGATCTTTCATTGGCTCGAAAAGAAGCTCGAAGAAGAGGGGAAGAGAACCTGATCCAATTGAACAAGACAGCAAGTGTGGAGAAGGTGATGCTATATCTGCCAAAAATGATTCTGTAGAGAGAGGAGTAAGTGACAGATACCTTGGTCACAATAGTAGAAGCTCAAATCAGCAGAAGAGAGCTGCAGAGTCAGCTCATGAACATTGCCAGAAAAAAGATGATACACATGCGCAGGAGAATACTTGTGCTGAAAAGCATTATAACAGATTCCATAAACACAAAGGAAAATATTTCCTGCAGGAAAGGGAAGGGTTGGATTCCTCAAACATTGAGGCCCATCATGACAGTCTGGATAGCGAGGAAGATGAACACTATAGGCATAGTAGGAAAAACCGGAGAAGGATGAAGAATGAAGCAGATTTATCTAGCACTGCTTCTGATGAGGATGGGCTTTGCAGGGTTAAGGACAGGAGCCAAAAAACCAGGAGAAAAAGTTCAAAGTCTAAAAGCAAGAGAAGTGTCACAATTAGGAGCGATGAAGATGGTTTGGACTGTTGTGAAGATAGGCAACATAGATTTGTTGAGAAAAACTCGAGATTGAAAACAAATATCAAAGAGAGTTTTGATGGTAGAGATAGACTTGGTAGGGGTGAAAAGAGGCAAAGAAAAGTTTTAAGCCTAAAAGATCAGTCAGTCACAGACATGGAGAGTGACAGAGATGAGGCCAGGCGTCATAGACACCGTAAAAAGCACTCAAGACAGTAAACATGATGCAGACTCGTCAGAAAGTGCTTTTGAGAGTGATAGATCTGGCAGGTATGAGAGAAGGCCCCAAAAGAGAAGTACAAGTTTCAAGTCCTGTAAAGAGTCAGACGTTACTGACTTAGCGAGCAGTAGAGAAAAGGGTGTTGGGAAAGAGAGACGTCGTAGACATAGGAGTAGTGCCAAACATCATGTGAAGGTGCGTGGCTCGTCAAAAATTGATTCTAATGCAGATAGCCGGTTCAAGGTCAGAGACACGGCTGAAGACAAGGATGAAGCAGATGGTGGCAATTTTGATACTTATGTCAGATCCAGAAGTTCTTGTGCTGATCTAGACCAGAATCATCCTGTGCAGGAGAGTATTGATTATCAAGGAGGGATATTATCGAGATTTAGTGACACTGAGAAGTGCAATGACTCAATTGATGGGGTTTTGGTATGTCGAAGGGATATCAAAAGGTGTGGCAAAGATCATATGTTACAGAGTAATAAAAGCGATGAAGGGGCTAATGTGTCAACAGAGGCGGAGACTTGTTCAGTGCCCACCATTTACCAAGACTTTGATGCTGACACAGAGGCTGAACATGAAAAGGCATGCCGCCATGCGGCTTTAAAGAAATTGGAAGAGAAAAAAAAGAACATGGATAGTCAGGCTGTCCATGGCAGGGAGTCTGTGGAAAGCTTCAGATTTTGCTCCCATGAACATACAGTTGCAATGGAATCTGGTTCAAGTAAATTGGGAACAAATGTTGCTTCAATACAAAGGAGAAGGATTTAATTGTCATGCAAAGTGCTTCTATATCTATACAAAGTAAATGTGTCACGACTTGTAACAAGTAGTAGTTCTCAAAGCTAACTATGGTTTTGAACTTATCAGTGCTGTAGTTTCCTTTGAATGTTGTACATTTGTTTGTGTGTGTTTTGGTGCTTGAAAACCTGAAGCATCAAGCAGATAATCCCCGATAATGACTACCAGAGGACTTATCATAATTTAATTATTTGACATAGTTGCTAAATACAGCAGTTCTCTCTGTAGCAAAATAATAGAGAGCAAGTTTTAGAAGATTGGGAGTCTTTACTGAGTTTGGTAAGTAAGTGAGTGAGTAGTCCTTTAACTCAAAAGTTTGTTTTCAAGTTGATCATCGC

*S.maritima*44459

CCAAGTCCAGCCATACCCCTTTCTACCCCAAAGACGCACCAAAATCCCCAAATGGAGAAAGAAAGGTACGATGAGAAAGGAAACAGGCAGCATGAGGATGAACTATTCTCATAAATCGCAGGCTCTATAATCATGTGCTCTCAACACCAAAACTAGGACCAAAAAAATTGGGTGATCACACACTAATTGAAGATCGTTCATGACTCATGAATTCCTCCATCCTTCACCAGCATAGTTGGCTTGGTCACCGAGCTCTGCTTCAATCCGAAGCAACTGATTATACTTGCATAGACGCTCCCCTCTGCAAGGTGCGCCAGCTTTGAACTGACCCGTAACAAGACCAACAGCCAAGTCAGCAATGAAAGTGTCTTCAGT

*S.maritima*565601

AAAAAAACAGAGTGAAAACTGAAAACCAAAGTGAGGAAACCACATTGAAAGTGAGAGTAATGGCTAACAATGGTGCAAATTCATCCACACAACCTTCGATTCCCATTTTTAAAGGGGATAAATATCATCTTTGGAGTCTCAAAATGAAGACTATGTTCAAATCTCAAGAGTTATGGGATTTGGTAGAAAACGGTTATGAGGAACCGGACCCGGCACCGTCGGCGCCTAATCAACAATTGAAGGAGAATCGCAAGAAAGATGCAAAAGCGTTATTCTTCATTCAATCAGCCTTAGATGATGAAATTTTTCCCCGAATTACATCAGCCACAACTGCACATGAAGCTTGGGAGATACTCAAACAAGAGTATTTGGGTGATCAAAGAGTGATTAAGGTACGTTTGCAAACTCTTCGAGTTGCTTTCGCTGAATTATCCATGGGAGAAAGTGAGTCTGTTCAAACTTATCTCTCAAGGGTAACTGAGATTGTTAGCCAAATGAGATCTTATGGAGAGAGCATCACTAATGAAATTGTTGTTTGCAAAGTCTTGAGAACTTTGAATGGAAAATTTGACCATGTTGTCCCTGCAATTGAAGAATCAAAGGACTTATCTACCTATACCTTTGATGAATTAATGAGTTCTTTACTAGCACATGAGGCTAGATTTAAAAAATCTAGTGCCAAGGTGGAAGAGAAAGCCTTTCAAGCAAAAGGGGAGTCTTCATTTAGAGGCAAGTCTGAAAATTTCGGCGGTCGTGGAAATGGCAGAGGCGGTTTCCGTGGTCGTGGTCGCGGTCGCGGTAGCAGAGGACGTGGCCAATATGTTGATCGTGGGCAATATGGTGATCGTTCACAAAAGAGCTCCCTTCAATGCCAATATTGCAAGAAGTTTGGCCATAAGGATTCTGATTGTTGGACAAAGCAAAGAGATGACAAGCAAGCAAACTTTGCTGAAAATGTTGACAATGAGTGTAAACTTTTTGTGGTGCAATCATCACAAGAAACCATTAACGAAGGAGTATGGTTCGTGGACAGTGGATGCTCAAACCATATGTGTGGTATGAAGACAATGTTTAAGGAGTTGGATGAATCGAAGAAGGGTGAAGTTCGCATTGGAGATAACAAGCTTATGCAAGTTCATGGAAAAGGAGTCATTGGCATCAAAACTGATCAGGGTAATGTTAAGCTTCTAAATGATGTTCAATATGTTCCAAATTTAGCTTGCAATTTATTAAGTGTTGGACAATTATTGAGAAGTGGTTATTCTGTCTTATTTGACAATGGTTATTGTTTAATTCATGACAAGAAAAGTGGTGCACTTGTAGTTAGCATACCAATGACACAA

*S.maritima*44770

AGCTTTTCCAAATACATTCATTCCCTCTGTTCTATTCTATTCTATGGAGAAACATGACTCCCTAAAATCAGACAATACAGCGGTGCATTTGATCCTGAGTTTACCTGTGAACCTATTAAACCATTAACCATGTAATGTTTTGTCACACATATTGTTCTACATCTACACACAGATATTGTAGCTCAACCGCATGTCCCGCTTGCGCTAATTTTTTGTGATTCTAACCAACTTGCACTTGCAACTCCTTGTTATTCAGAGTACAATGGAATCAAGATAAACAGAATATGGAGATGAAAGAAATTTCTTACTAATTTTCACTATATTCCTTTGTGGTGGTGGTAGCATGTGTACAACAGAAGCTCTTTTTACTACAAATCAACAAATATATATATTTATATAGATACATCAATCTCAAAATAGTGAAATTATGATGCAGGGAGAGACTGCCAAAAGAATTTCCGAAAAATAAGCTGTGGGGCTTTCTGCCTCGGTCTAGATCACAAGAATTTGCTGTTCACTCCAGCACGATAAAGTTGCAACCTCTTACCAGAAGATTCAATGCCCAACTTTGTATTCGCTGGTGCAAATAGCACATCACCTACTCTGATTTTCTCCATTGAGAAAGTAGTTCTTACATATCCACTCCCTTCAGTCACCAAAAATAATGACGGACCAGCCATTGCAGGAAACTGTGTTGAAGTTCCTTCAGGGAGATCACAATGATCCACCTCAAACTCTTCAAATGGAGGACGATATCTTTTTGTGCGTCCATTGGTACCCTCCACTTTGAAGCCTCTTAGTATATTAGGACGGCCCTGTTTGTATGTGAGCATCTCACACAAAGTCTTAACATCCCTCTTCTTGGGAGTTAGACCAGCCCGAACAACATTATCTGACGTTGCCATACACTCAATACAATCACCAGATACATAGGCATGAAGTTCATTTGCCCCCAAGCACAAGGCTTCACCAGGATTCAGCGTTACAAAGTTCATAAGAAAAGCAGCTAGCACACCCACATCGCCTGGATATTCTTTCTCAAGCCGCAAGACTAACTTCTCTTCCGCTGTTAGCTCATCCTTTTTCTTCAAGCGACTAATTAACTGTGACAACACTTCAGCAATTACATTCTCACTTGCTGACATGATGAGGGTAAATAGTTGTTTCAGAGCAACTTTAACGGCATCCTCTCCATCTTCCGCGGTGATATTCAACACTTGATTCACAACCGCGGCACCAACCAACTGTGAGATTTCTGGGACACCTTTAAGAATCTTTTGGAGATCCTCAAGACCAACAAATCCACAAAGGATCTCAAATTTGGTAATAGCAAGAGCCATTTCTGGCTTATGATTGTCATCCTTGTATACACTTGGGTCCTTTATGTGCAATTTCTCTGCTAATTCCTTACAAGGATGAGCTTGTATTGACAACGTTTTCGCAACCGACAACACCTTAAACAAAAATGGAAGCTCATAACCCCATTTCTCCACAACTTTTCTACCAAGCACATTAGGGTTCTTCTCAATCCAAGACTTAAGAGTATCACCTTGTAAATCCCCACATCTTCCATTCAAAACAACAAACCCATGATTTCCATTTGCCAAAACTTTACCCTTTTTAATCACATGAGATGATCCAGATTCATGGGTTCCCATCCAAAATTCAGCATAGGGTTTGTGGGGATCCAATAAATTAGCGTCGGAATTCGCGCTATACAATCTATAAACCTCAGAATCACTCCCAATTTTGCCCCAATCATAGTTTTTTACTGAACATTTAAGTCTTTGAAGCTTTTCTTCATTCCAGAAAACCCCATTAACCGCCATTATTGCCTTGCTCTTCTTCTTCAACAATGAAAATAAACAAATCACAACAAAAATCAAGGAAATGGGTAGTAAAGTCAAGTATGAAACATCATCAGCAAACTCTGTACTCATTTGGAAAAAAAAAAAAACAACCCAATTTCCTGGAGGAGAGAGGAAAAAAAAACTCCTCCTCCAACCAAAACCCAGAAATCAAATTGAAAAAAAGATTGAATTATGAAAAGAAAGATTGAAACCCAACAAATGTAGATGGTTTAGTGAAGTGAAAGAGGGGAACAAAAAATTGTTTATTATAGAGAGAAACAAAATGATGAGAAGAATGTATAATTGAGAAAATGGAAATTGTGTGTCATTTCTAAAACAAAGGTAACACCTTTATATAAAGGGTCAAGAAATTGATTGGAAAAAATAAAAAGAATTTAAAGATTGAGATCCAAATTGATGATGGTATCGTAGGAGAGAGAAAGAGAAGAGAAGAAGAGTAGTTTGAAAGGTATAAATTTGGGGACAAAAATTAAAGATGGGTATTTCTGTTTTGCAAAAAGAAGATTATAATTTGAGAAATTAGTTTGAGATTAAAGGAATTTGGAGAGAGAAAATGTGAGATTATTGGAAAATGGAAAATTGAGGTTGAAGAAGAAGAGGATGATGATGAACCGGAACAA

*S.maritima*44550

CAGATGATTCCAGTACTTTCTCATCCTCCTCAACTTGAACATCTTCATCCTCATCTTCGTCATTTTCTGTAAATACAAAATGTTCGTTCAGATATTTCCCACTTCACATTCTGCAAGCACAGTCAGCCCAACGTGCACAGAGACATGAACATCTATGTATGGTTCAGCATTGTACTGAAAACTCTCATGTTTTATCTTTAACAGAATGCCCTGGCATGGTTTCCTGATCCATCAGGCAATGCATTTCTGATCTGGAGGGCCACCATCAAGATGTGGCCAAATATTGAAAAGACCTTTTGCTTCCCATATCACTGGAGGCTTGTGCAGCCTCTCTCTGGACTTTGGCAGCTGTCTCCCCCATCCTGCCACAGATCTGATTCCCAGGAACAGGCTTGGTGTCCTGTCACAGTTCGCATTTCAAACCTCATTCTTTCTCTTAGGAGAGGACAAACTTGTCCCACAGTCCTCTATGCATCAGAAGATTTCAAGCCTCCAAGTGGCTTCTGCTGTGTTATTCAGGGACATTCTATCCATGGGGAGTGCTCCAGTCTGAAGCACTTCCTACCACCAAATGCCCCCACATCAAGTGCCTTCTCCAACACCACATGGAGAGGGGCTTCATCTCATTTTGAAAAGCATTCGTAAGTGTTCCCATATTTGGATGCTTCAGACCCTTGCAAGAGACAATTTGTCTGCCTTTGCAGATGGAGAGAGAGAAACTCTGGAAAGATAAATCACTCACTCACCGACACTTACTAAGAACATTGCCAAAAAGACAGCCTGGGAACCTTCATTCTTAGCCCAGAGCTCTTTTCACTCCAACAAGCGCCCTCCCATCACAGCCTCCTTCCTGTCCTTTAAAACTAGATAGATGCTGCCTCTTGCTCCAAAGACCACCTTCCATCAAGGAAGGAGGGACACTTGCAATACTGTGACCTCCAAACCCATGGGTTTCCCATCTCTGTTCTTACCCAGGAAGTCCTGGTCATGTCATGGCCACATATGTGTAGTAGAAAAAAACCCCACTGATACAACTGTCATTGTGAAAGTATGGAGGTCTGGAGCCTCTCATAAGCCTGGGGTTTTGGGTCATCAGGGCCTATGGCCACCTTACCTGGGCTGAGCTTTTGGACAAGGTGCTGTGCCAGTCTACACCCCTCAGCCAGCTGTTCTTGGAGGTCCTGCCCCTGGGACTTGTCCGGCTCATCCGGAGTGAGGAGGGCCTGGAGATGCTGATTCAATGAGCGGGAGGCATCTCTCCCTTCCCGTAACTTCTCCCTTAACTGGGTCAGCTCTCGTTCCTGAGAGTGAACCAGGACTTTATATTGCCTAAGGTGAGACGGTAGAGAAAATTTAAGAGTGGAAAGGGTTGAGTGATCCGCTCAAATATTGCAACAGAGATTTCTGAGACAATGTCCTCAAGGAGACCTCCAAGCAGAAGGTCAGCACATGTTGAAAGGAATGTCTGTGGCCAAGAGAAAGAATAGAAAATGGTTTACAGGCTTCCTCTGTATCAGAGAGGGCTCCTGCAAGATCCTCGATGATGTTCCATTCATCTTTCCCTTCTGTAAACAAAAGTAGGTGTCTTCCTAATTCCGTTTCAAAAAGACATCCTTTCAGTTCCTCACTCTGGCCATGGACATTTCCATGTGAAAATACACATAGTGCATCTTGCGGCCACTAGATACAAAGCCATGTACAGAAATGAGGCCAGGTGCAGATGGGGCGAATTGAAAAGATGAAAGAAGAAAAGAATGACAGGGTCGAGAAGGCAACATTGATTGAGTGAAAGAATGAGAAGACGCAGTCAGTCAGAAGGTGATTCTCACTAAGGGTAAGTGGGGTGGTGATGGCACACCATTTTGAGTATACTGAATGCTGCTGTGTGGTTCACACTCCTTTGGTTAATTTTGTGTTATGTAAATTTCACATCAACAATTACTTGTTTGAAAAAGAGAAAACAAGGCTCTGAGAAACAACTGCAACCCATAAATTTTTATTATCCTTCTTCTCTGTTTGATAAATATTTGTGTGTAGCGAGCCTGCCATGGCAATTCCTGCCCTTCCCCTGGCCCAGCTTAGCTCTTACGTCTCCCCACCGAGCTGCTGTACTTCAGAGATTTACACACCTGCCCCCCTGCCTGCCCCCATGGGGTCCCCTCACCTGAGCTCCTCAGCTTGCTTGAGCTGCTCTGCAAGCTTCTCCTCCTTGAACTGTCGCTCATTCCTCAGCATAGATTTTATGAGGTCTTTGCACTCTTCATATTCTGAGAAAAGACAGACACGCCTGCCTCAGTGGAAGGCTGGACATGCTGCTGTGGTCATTGCCTACAGGGCAGGAGCCAGGTCCATCCCAAGGACAAAACTCTCCCCAGTACCAGGGTCTAGACAGGGATTTCCACATCTTTACTCTTCAGTCTCCTGACTTTCTGGCATCTGATCCTCCAAAATTTAGAGATGAAGAAAGAGAACCTCAAGGGCACATCAAGGAAGTTGACAAGATGATTCAACCACAACGAAGTGGAGTCAGAACTCACAGCCCCTGAGGTCTGACTCTGAATGCGGGGCCACTTTCCCAAGCCTTGCAGCCTCTCCTCTAAAACACTGCACTGGGGCATGAAGTAGTGATTTCTTGTACAGTCGGGAAGGCCCCTAGGACTATGGGACTGATGGTTTCCCTTTTACTGGGAATTTCAAGGACAAGTATGCGAAAGAT

*S.maritima*45135

AAATTAAATGACATCCAACTCAAAATTAAAAATATCGATTGACCTAGAAAAGGCCTTTGCATATATATATTTTTATATAATCATAATTCAACAACCAAAACAAGTTGCAAATGCATAATGGCTAAAGGAAAACCTAAGGAAAGAAAACTACATCATTTGCAGCAGCATCTGGAAAACGAGCTGCATATGATAATGCTTCCATGGCCTTCAGCTTGATAGTAGATGGGATTTGATGAAAACTTAAACCCACCCAGAAATCCACAATGACATAATTACTGAGCCACATTAACGCTTGCAGATTGTCTAGCTAGTGTAGAAGTTGTTAACCACATTGGACGGCCCAAAATTGCGCTTCCATGGACAGAAAGATATGCTGCATTGAGTCGGTCAACATCGGGTGTAGCCAAAGGATCAAAGAGCTCCTCCCTCTGCTCCATTCCTGGAGCAACACCAGCTGCAGCAGCCATATCTTGAACACCATCAGCCTGTTGAGGATATTGAGCATACCCACCATATGCATTGTATGCGTATAAGGATGGATCTTGAACAGCACCATATGCATAGGCATCATAACCTTGCCCATATCCATAGTATGCAGCCCATTGATTAGGATCCATTTGCTGACCCCATGCAGCAGGGTCCTGTTTAGCAGTTGGGCTCCTTCCCCACAAAACACGAACTACTTGCTGACCAATCATGGCTCCTTGAAGTTTCTGTATAGCTTCTTCAGCAGATGGCCTATACCAAGCTGCAAACTGCACAAAACCACATCCCTTACTAACTGGGATCTTGACATAGTCGAATCTCTTCAAAATGCAAGCAACGCTGTCTCAGCTCTTCTTCTGTTACATACATTTTGGGTCCAAATTCCCAATATATATCTATCTTCGTCAATCTCTCTCTGTAACATAAACAAAAGGGATATGCTGAATCTTGGGCCAGTCGCTACCATTTGGGCGTTGACATCGTTCTGTCAATCCTTTACACCTTCTGATTGTGAGTTGGTTTAAGGTGGTGAGGTTGCGGATGGCTTCTGGTAGACTTTTCAATTTGGGACATTCAATTAATACCAGATTAGTGAGAGAGGTGAGACAACTTATCCATTCTGGTACTTCCTTCAATTCAAAATTCCAAAATAATTTCAAAGAACGGAGGTTTGACAAGTGTTGAAGCCCACTCGGAAGACCCACTAGTTTTTTAAGATCAATTAATTCCAAAGAACGAAGGCTTGTATTGAAGGCTATCCATGGCATACCCTTACCAATACCATTACCA

*S.maritima*36986

TGATGATCAAATGCGGACTAATCAACAAGTTATATTGATTTTTTCAAGTGACGCAAAAATATATGCAGAATTATAGAAAACATGTATCAAACATATAGCAGAACTATAGGGTCATAATAACATATAGGCATATACTTTCTTTCAATCCAAAACACATTTGACAAAATATTACAGCCATAGAAATGGAGAGCTGAGGTCAATCTACCATCTAAGAGCGAGGCTACTATTCAGAGCTGCCACTAGTATACTTCTGAAACATATGTTACCCTTGCATTACTTGAGGCCCTTGGAAAGTTTAATAATTGCTTCACAAACTTCAGCTCATTGCTAGCCGCAAACTTTTTCTTGCAGGGAAGGGGAAGGATTACCATCTTTTCCAAGGTGACAGAATTTTTGAGCAAAAATTCCGCTAGTTGAAGCAGGCTAGGACAAGGTATTGTGTAGCCACGGATGTCAACAGTTTTCAGTTGATGCATCAAACAAGAAGGGAGCTCCGGGGACAGCGCATTGGTGTCATGTTTTCTATCCGAAATATGGTGTGAGTCATAGTACACTACAAGTTCTTCCAAGTGAGGTGAACTTCTCAAAACTCTACATATGCCTAAGAGTTGATCACCAAGAAGCAAGGACTTAATATGTAACCGTTTCCATCTTGTTTGAGGAAAATCTTGCTTCTTAAATATTGACATCTCAACCATGGCAAGTGATAATTTTATTAACGATGGCAAGTGGACTTGACGTAGCTCATCGATTCGACAACATTGAAGGTTCAGTGTAACAAGAAACTGACTTGTGAACACACGCATTGGCAAAATAGAACCGTCATTCTCAACAGTACTTGTAGTATCTCCGTCCACGAAATGTTGAACGATTAACTCCTTGACTTGTCTGTCTACTGCCACCCTTAACCATGTTAAAATTTCATGGACAACTCCATATTCACCAACATCGTGGAAATTAAGCTTAAATTTATCAATAGGGGTCTTTTTATGAACGATTAGCACATTGCGCACAAAATTGGCGAAACGAGAATTATACTGGTCGTAATTAGATATATCGGATTCAGACCAATCAGGCCAAAAGTCAGACTCATAAAATTCAAGGGAAGGAAGCGAAGTCCAAAGATCACCAAATCTTCGAATTAAGACGGTTCTAACAGCATCAACAATTGGCAAAAAGCTAAGAATGTGTACCAGAATTTCATCGGGTAATTCGCTTAACCTATCTCTCCCTGAAGAAACCCTTTGCTCTTTTGAAATGGAATTCATGATTTTTGAGGTGGTAGTTCTAATTGGTGTAGTTGAATTCCTGAGTAGTTAGTAATTTTGATTTTGGGTTAAATGTTGAACTGAAGTAAAGTAGATTAAGCGTTGGTCAAAGACCCATTGAAAATTGAAAATTGAAATTCTTCTGAGTTGCTTGAATACTTTCGCAAAACAAGAAATTGGAAGGGTTGTG

*S.maritima*173919

ATTTTTTTTTTACTTTTGAATAAAAAGATAACATCAAGTAAAGGAAATAATTATGTTTAAAATAATAATAGAATTCTTCTTTTTTTCAACATGCTTAAGCTAAAATGAACTGTACTTATTTTAAGTTGCCCTATGTATTCAATGGGTATCCTTTTAAATATTTAACATCCTAATAATCGTCAATGTCATAGAGTTCGTTTGGATCCAAATCATCACATTGCATACTTTGGAAATCAACTTTATAACGTAATAATCCTCCTATGCCACCAAATCCTCTTACAAATTGTGATCCTTCTTGTGATTTATCTGTGATTATTTCTAAGGTAGCTCCGAAAATTTTATAATTGTTTGCTAACCATTCCAAAAGTGGCTGACACTCAACTAATTCCAATTCGACTCCGGTATCTTTTTCTGTGAAATGTGATTTATCTTTTTCTTGTTCTGGGGTCAAATGTAATATTTTTTCTTCAGCTGTTGTGTGGTTTTTTAGGACATATCTCTGAATGTCTAGGTTCTCCCAACAAATTAATGTTTCAACAGATCCAAGTTCTAGAGCTTTTAAAGTATCCTCAACACCAAAACAGTATTTTCCAGTATCTTGACTGATTTCATCAAAATATCGTCCTATTAATTTTTTTTCTTGAATAAACTTAACATTTTGTAATGATTCAGCAGCTAATTCAATCGCTTGATTAAAACCATTTTCACCACCATACGATACATCAACTAATTTAATAATTTTAGCTTGTAACCGAGGATCAAACATATCAGATTGACTAAGTTCTGTTTTAAAGTCTGCACTACCCGCCAATATTAATCCAGCTATATTAGGTTTGTCATTTGTAATAAACAGCTGAGTGGCTACCTCTGCTACTTTACGTACATAATTGTGTCGTTTTTCCATACGTAGACGGGCAAAACGTAAAGCAGATTGACCTCCTCTACCATGCTTTTTCGGTAAGTCAACAGTAAATTTATGAAGAACCTCTCTGGTGTTTCCTTGTAAAGTACCAAATAGTGCACCATTACCATCCATCACAATGAAACCAAATTTATTGTCATCTGCCAATAGTGCTGTAAGCGCTTCTGTATGAAACTTATTATCACAAAGGTACAAAGAAGTATTAATAGGCTTGAATGGCTCAAAATCAATATTGACTTTCTTTTCTTTGCCTTCCTCAGTTACAATGGTTCCACAATACACCACTAATCCATTAGGTGGCACTTTAGTATAAAGCTTTAATCTTTGCTGTACTGATGTAATAGCACCCAATACTGACAATCTGTTTACACGTGACTTGATGTTTGATGCAGTACCATATTCATCAGCCAACATCTTACTCACTCTGGAAATCTGATCCTTAGGGGGTATGATTAACGAAATCATACTTGTTCCATTACCTCTGGCCATCTCTAGACTCTTGATGAGCTTCTTGATCTTCCATATCTCTACGTTGCGATCCGAAGTGGTCTCCTCGCCGTTGGTGGTCATTTCGGGTGAGTGCGAAGAGTACAACGAGAGAACGACACGGGAAACTGAGAAAACGCACACACGGCGGACGGACTGACGCGCGACTTTACGTCAACAACAGTCTTCTGCAGGTTTTTACCGCTGTCCCCGACCAGAGCAGCGACAACTGACAGACAATGTGACAATGGTGTGCGATAAAATATATATTGTTGTACACCGTCGTATTAAATATTTTATTTTTTTAAATTTGTTCAATTAAAATGTGTGACTCGGCGAAGTAGGTTAGGCAGGCGCG

*S.maritima*36986

TGATGATCAAATGCGGACTAATCAACAAGTTATATTGATTTTTTCAAGTGACGCAAAAATATATGCAGAATTATAGAAAACATGTATCAAACATATAGCAGAACTATAGGGTCATAATAACATATAGGCATATACTTTCTTTCAATCCAAAACACATTTGACAAAATATTACAGCCATAGAAATGGAGAGCTGAGGTCAATCTACCATCTAAGAGCGAGGCTACTATTCAGAGCTGCCACTAGTATACTTCTGAAACATATGTTACCCTTGCATTACTTGAGGCCCTTGGAAAGTTTAATAATTGCTTCACAAACTTCAGCTCATTGCTAGCCGCAAACTTTTTCTTGCAGGGAAGGGGAAGGATTACCATCTTTTCCAAGGTGACAGAATTTTTGAGCAAAAATTCCGCTAGTTGAAGCAGGCTAGGACAAGGTATTGTGTAGCCACGGATGTCAACAGTTTTCAGTTGATGCATCAAACAAGAAGGGAGCTCCGGGGACAGCGCATTGGTGTCATGTTTTCTATCCGAAATATGGTGTGAGTCATAGTACACTACAAGTTCTTCCAAGTGAGGTGAACTTCTCAAAACTCTACATATGCCTAAGAGTTGATCACCAAGAAGCAAGGACTTAATATGTAACCGTTTCCATCTTGTTTGAGGAAAATCTTGCTTCTTAAATATTGACATCTCAAATTTTCTGTCTGAGTATTTGAATACCTCTGCATCACAGAACTTGTTCATATATTCATAAAAAACATGTATGATACAGCAAGACTTGATATTGACTTCACGAGTGGAAGAAACGTCAACCACTTGCATACAATCTCTACTTGTAGAATAAATATCCAAAATTTTCAAGTTGGGGCATTCAAGCAAACCATTGTAGGAAGATAGCATTAACTTGTTAATGTTTGGAGCAGTAAAACGCAGCTCGGTCAGTGAATATACACGATGCAGATGCAATTCTTGTAAAGAAGGGCTTCCTAATACAATTTTATTGAAAGTCTCATTACTCACCTCAACCATGGCAAGTGATAATTTTATTAACGATGGCAAGTGGACTTGACGTAGCTCATCGATTCGACAACATTGAAGGTTCAGTGTAACAAGAAACTGACTTGTGAACACACGCATTGGCAAAATAGAACCGTCATTCTCAACAGTACTTGTAGTATCTCCGTCCACGAAATGTTGAACGATTAACTCCTTGACTTGTCTGTCTACTGCCACCCTTAACCATGTTAAAATTTCATGGACAACTCCATATTCACCAACATCGTGGAAATTAAGCTTAAATTTATCAATAGGGGTCTTTTTATGAACGATTAGCACATTGCGCACAAAATTGGCGAAACGAGAATTATACTGGTCGTAATTAGATATATCGGATTCAGACCAATCAGGCCAAAAGTCAGACTCATAAAATTCAAGGGAAGGAAGCGAAGTCCAAAGATCACCAAATCTTCGAATTAAGACGGTTCTAACAGCATCAACAATTGGCAAAAAGCTAAGAATGTGTACCAGAATTTCATCGGGTAATTCGCTTAACCTATCTCTCCCTGAAGAAACCCTTTGCTCTTTTGAAATGGAATTCATGATTTTTGAGGTGGTAGTTCTAATTGGTGTAGTTGAATTCCTGAGTAGTTAGTAATTTTGATTTTGGGTTAAATGTTGAACTGAAGTAAAGTAGATTAAGCGTTGGTCAAAGACCCATTGAAAATTGAAAATTGAAATTCTTCTGAGTTGCTTGAATACTTTCGCAAAACAAGAAATTGGAAGGGTTGTG

*S.maritima*30865

GTATGTTTCTACAAGTACTAACAAAAGGAAAATAAGAAAACAATATTCAATTATTCATCCCAGAAACACAAGCGAGCAGAAACCCTAACAATTCCATCCCCCAAAAAATGCAATTTCAGCACCTAATTCAATCTCCTCGAACTTGATCTCCAAACAAAGTTAAGCTCAAATCCCTAGTTTCTTCTCCAATTTGTTGTAATTATGGAGATTTTAAAGGATTATCCTTCAGAAATCGAAATTGGAAGTTCAATTGAAACGTTTCAGAAGGCAATGGATTCACAGCAGCGAATTTTCCATACTCAGATCGATCAACTTCGCAGCATCGTTGTTACTCAGTGCAAACTTACTGGCGTCAATCCTCTTTCTCAAGAAATGGCTGCTGGTGCTCTAGATATTAGCATTGGGAAAAGGCCTAGAGATCTTTTGAATCCGAAGGCTTTAAAATATATGCAAGCAGTATTTTCTATAAAAGATGCTATCACTAAGAAGGAGTCTCGTGAGATCGGTGCGCTGTTTGGTCTAACGGTTACCCAGGTTAGGGAGTATTTTGCTGGTCAGCGTTCTAGGGTGAGGAAGATGGTTCGGTTATCTAGAGAAAAAGCCATTAGGGTGAGCGCAGAAAAGGATTTGCAGGATAATGTGTCTACCGATTCTGACCTTATGCTACCCATAGATCCTACACCATTGAGCTCCATTTCAAATGAGGAAGAAGCACCTTCTTGTTCAAACCAGGATGAAATTTTGCCGGGATTAGGAGAGTCTGAAAGAAAATTTGTTGATAACATATTCAGCTCAATGTGTAAAGAAGAAACCTTTTCTGGTCAGGTGAATTTGATGGAGTGGATTTTGCAGATAGAAAATCCTTCAATCTTGTGCTGGTTTTTGACAAAAGGTGGTTCGATGATTTTAGCTACGTGGTTGAGTCAAGCTGCTATAGAAGAACAAACAAGTGTCGTTTCTGCTGTTCTCAAGGTTTTTTGTCATTTGCCCTTGAATAAAGCTCTTCCTCCTCATATGTCAGCCATACTCCAAGGTGTCAACAAATTGCGGTTCTACCGAATACCAGACATCTCAAACAGGGCAAGAGTTCTCTTGTCGAAGTGGAGCAAAATGTTTGCTAAAAGCCAAGCTTTGAAGAAACCAAATGGAATTAGAGCTGCTGGTGCTCCCCAGCAAGAATCTGACCTAACACGAAGGATTGGTGAACTTGTTGGAGATGATTCATGGCAGTCATCTGTTGATTACTCTGATGATATGTTAGTTCCCTATTATGTGGATCCTGATGATACCAGGAAAGTGGAAACTCTGGAACCGGTGAAACTGCTCACTGCTTCAGAAGAATCTAATAAGAAGCTTATCCTAGGGACATCTGCTGCTCATAATAAAGAACGGAGAAAGGTTCAGCTTGTTGAACAACCGGGCCAAAAAGGAGCTAGTAAAAGCCAGCCTGTCAAAGCAGTGCTTGCTAATCAAAGGCGTCCTATCACTGCTGATGAAATTCAAAAGGCAAAATTGCGTGCACAATATATGCAGAGCAACAAAATAAAAACTGAAGGTCCAAAAAAACCTTCTTTACTGACTAATGATTTACTTTCAGCATCAGAAGCTTACCTTCGACCAAAGTTGGAAGCACAGAAAAAAGCATGGTTGCTTGCTCCAAAAAAATCGGTTGATTCTGCTTCTGATGAGAAGCCAGTGTGTGATGAGAAGCCAGTTTCTGAACCAAAGGAGACTTTGCTGGAAAAGTGTCGGAGGGTTCAAATTCCTTGGTCGGCACCTCCAGAAATTCAGCTCATAACAGAAGTAAGCTGTGGAGAAAGCAGCAAAGAAATGGAGGTTCAGAGGAACCGAAATCACCGGGAAAAAGAAAGCGTTTACCGGACTCTTCAAGAGGTCCCACCAAACCCAAAAGATCCATGGGATACCGAGTTGGATTTTGATGACTCCTTGACCCTGGAAATACCCATCGATCAGTTGCCTGATGCTGATTCCATGGATGCTTCAGTTGATGGTCATGGTCATGTACCTGAAACTCAGAGCTCGAATCCCTCCACTTCATCACAGTCCGAACCTGATCTGGAGCTCCTTGCAGTTTTGCTCAAAAACCCGGAATTGGTATTTGCTTTGACTTCAGGCCAAGCAAGTGGGTTGTCGAGTGAGGATACGGTTAAACTACTTGACTTGCTCAAGTCAAGTGGTGGGGCAGCATTGTTAAACGGACAAACTGTTGAGAATCCAATGGAGGCAAAAGTTGAAGTTTCTCTTCCTTCCCCTACCCCGACAAGAAACGTTGAAGTCTCTCTTCCATCTCCGACTCCACCGAGCAGATTTGAAGTTTCTCTTCCCTCCCCGACACCATCTACCAATCTTGGGCCGGTGCGTGAAAGTGTATTTATATCGTCAAGTTTCTTGCAATGGATTCGGGAAGTTTTAATGGGTATTATTATTGCTTGTGCATTGTTCATCCCTCAATTCACATTCTCACTATCTTCACGAACTAATTAACCCCTCATTATGCCTTCACCCTAGCAAATGCTCTTTAGTGTTGAAATTTAGTCAAAATAAGAAACCACGCTCATCTCATCATTTGCCTCAAAATAGACAATTGGACCCTTATGTATTCATTTTTGTATGAGATAATATCCTCTATGTGGGTGGTGTGG

*S.maritima*1952088

GAATATTCAAGTTAGTATTGAGGGTTTTGTTCGATCCAAAGTTTCACCTGTTCCTAAAGGAGAGTTTCCAAATGCAATGGAACCAGTACCGCAAGGTGGGGTTGATATCGTTCTATATGATGCAGATGCAGAACACGAAATAGTTATCGAAGGAAGATAGTTTTTTTTCTTTTGCTGATATAGTAGCTACAAACTAGTTGGGTACGTGTAGAATTAAGATCAAATTCACTAGTTTTTGGGTATTTTGTAATGTTTAGGCAGTTTAAATGTAAGCATGCTAATGTACTCAACTACTAATTTCTAG

*S.maritima*275107

ATAAAAACACTTTACCTATAGAGGAAAACATATTAAGAATTACTTTGACTTTTCCTCAGAAACCATGCAAGCAAGAAGAGAGTGGAGTGAAATATTTAAAGTGTTGAGAGAAAAAAACCCACCAACCTAGAATTCTGTATCCAGTGAAAATATCCTTCAAAAATGAAGAAGAAAAGACTTTCCTAGATGATCAAAAGGTCATGGATTTCATCAATATCAGACCTCTCCTACAAGAAAAGCTAAAGAGTGTTCTTCAATCTGAAAGAAAAGGACATTAATGAGCAGTAAGTAATCACCTGAAGGTACAAAACTC

*S.maritima*926096

TACTCGTGAGTTTTAAATATGTTATCCGTTTATCTTAAAGTTAGATATCTAAAGAATGTAAGACCTATGTTAACAAAAAATTTTGTTTTCTTCTGCAACTGCTCCATATGGTTTTTGAGTGGTTCCTGCTTTTGACTGAGAACAAGGAAATGTGGAATTGTGCACAGGAATGAAATGATGGCCAGGCTTTCACTGGGTTATACACTGTCATCAGTAATAGTTCCTGACATCTCTCTCTTTCCTTAAGAGCTTTTTTCTATTCAAGAAAACAGAAATGGCATAGCCTTTTTAGTTTGCTCTTTGATTTGAGTCAGACAAATT

*S.maritima*20392

CACGGAGTTTGGTAGGTAGGCAACTAATAAGGAGAAACAAGCAATTTCTGCTTTTATTTGTGCTAATTAAGTTGCTAATTAAGCTAATGCATGTGTGTGTGATATTATCTACGCATGTGCTGGCAATAGTTTGTTAGGAAGAATCTTCCACCTCATTTCTTCTAGTGGATGGAAATGATGTAATAACGGCTTCCCTTATATATGGTGTCGTGGCCCTCTATGTAAGGTTGGGATAGATTTAATACAACAGAAAGTTTGTTGCCTACATTCTCTGAATTTCTTCAACAATTGATTGCAATCATTCTCAATCATCATCTCATCATTTTCTCATTGTTTTTGCATCTTTCGACTAAGAACATTTTAGTCATCATGGTATCAGAGCCATCAGTTGTTTGCTGAAATAGAGACACCAAAATCATCCCCTATAACCTTCTTCTACATCTTCTCCTTCTTCTTCTACATCTTCTTCTTCTTCCTCTGTTCTTGCTTTCTTCATGGCTTCGCGACTTACCTTTGCTGATATTCAAAACCCACTCTTCTTACACCCTTCTGATAATGGTTCCTCTGTTAATGTTGAAAAACTTCAAGGCGCAGCTGATTACAGATCTTGGAGGCGAAGTATGGAGGTTGCTCTAGCTTCGAAGAGGAAAATGGGGTTTGTCACAGGGCACACCATCAAAGACACCACTGATGAGGTCAAATCTGAACTCTGGGAGCTCTGCAATAATCTGGTAATTGCTTGGCTTCATAACAATGTTTCTTCTTCTATTAAGAAAACCATTCTTTACATTACTTCAGCTTCTGAGGTCTGGAAAATTCTTGAAAAACGTTTTGCTCAATCTAATGGTTCACGAAAGTATAAACTTAACAAAGATTTGTATGAGGTTAAACAGCAATCTGTTTCTGTGAATGAGTTTTACACAAAATTATCTGTTATTTGGGAAGAAATTGAGTCTATGAACTTTCTTCCACCTGTTACCTCTCCTAATGCTGAGGTTAAAAAACTTATTGAAGCTGTCATTAACTACAGAGAAGAATCCAAACTCTTCCAGTTTTTGAATGGATTGAATGAGTCTTATAGTGCTCAGAGAAGCCAAATACTGATGATGAATCCATTACCTACTGTAGAGAATGCCTGTGCATTAATACAACAGGAAGAATCTCAGAGAGAGGTGTTACACTCACCTAATCTTGAGAGTGAAATGTCAGCAATGTATAGCAAGCATACAAACAGTGATAGGCTGTGTAAAGCTTGCGGTCAGAAGGGTCACACATATGAGAGATGTTGGACCATTGTAGGTTACCCAAAGTGGCACTCAAAACATGGTAAAATCATGCCTAAGATGGGATACAAAGACCAGGCCGGTGGTGTATCAAGCTCTAAATGGGCTCAGAACAAGGTTTCTGCTACTCAAACCCCCAAAATGGCAGCTAATGTTCATGGTGCCTCCTCATCTAGTGAACAAGGCTTGCTGTTCACTCAACAACAACTAGAACAACTTGCAAAATTATTCCCTCATATGGGATCCTCAAGTTACAAGGCTTGTGAAACAGATGAGGAGTTAGACTCGCATTTTTCTGGAATGATATCATGCTTTAATGTGAAGGGTTTGGCTAATGAATGGATCATAGATTCTGGGGCATCAGACCATATGACCCCAGAATTGACTAAACTTCATCATCTCACACAAGTTGCAACCACTCTGGAGATCAATTTACCAACTGGAAATATAGCTCCTGTAACACACATTGGCAAGGTGGACCTGGGGTCAGGACTTGTGCTGGCTAATGTGCTCTGTGTTCCTGCATTCAAACACAACCTGTTATCAGTTCAGAAACTGATTAAAGACAACAAGTGTGAGGTAAAATTTTTCCCTAATCACTGTGAGATTGTTGATACAAAAACTAAACAATTGAGGGGGAGAGGAATAGCAAAAAATGGCTTGTATTATTTGGTTAATGCAAATAGTAGTGCCCCAATCTGTTTGAATGGAAGTGCAGGAATACCTAAGTCTGATGCCTTCACCCTGTGGCATAACAGACTAGGACATGCCTCCACCTCTAAATTGATGCACATTGACTGTGTTAAATCACATCTAAAACATGCTGCTGATGGTGTATGCTTGACTTGTCCCATGGCCAAGTTCACCAAGCTACCATTCACTTTGAGTACTTCTCATGCATCTGTGCCTTTTGAATTAATCCATATGGACATCTGGGGTCCATATAGGGTTTACACAAAAAATAAATACAAGTATTTCCTCACCATTGTTGATGATCACAGTAGATCTACTTGGGTGTATCTGCTTGAATTCAAATCTCAGTCCTTGAGTACCCTTGAAACCTTTCTCAATTATAGCAAGAATCACTTCAATAAGTCTATAAAATTTCTAAGGTCAGATAATGCCCTTGAATTCCATGATACACCTTCTCAAATGTTCTTCTCTGCTCATGGTATTATTCACCAAACTTCTTGTGTCAATAGGCCCCAACAAAATGCAAGGGTCGAGAGAAAACATAGACACATCCTTGAAATTTCTAGAGCTTTAAGATTTCAAGCTGGTTTACCACTCAAATATTGGGGTGATTGTGTTCTAACTGCTGCTCACATCATAAATAGACTACCCATTGAAATCCTCAAACACAAAACTCCATATGAAGCACTCCACAAAAAACCTCCAAAATATTCTCATTTGAAGGTATTTGGTTGCTTGGCTTTTGCTAGTAATCCTAGTGTTTCTCTTGATAAATTCTCTCCTAGAGGTGTGCCCTGTGTCTTTCTTGGATACCCAGCTACACAAAAAGGATACACACTCCTCAACTTGCTCACTATGACTACATTTGTTTCAAGGGATGTCTTGTTTCATGAAACCATATTCCCATTTAATCCTTCTTCACCTAATTCTTACCTAAAACCTCTTCCTGCACCTCAACCTATTACTACACAGCCTGCAGCTGTTGATTATATCCTCAACAATATTGAACTTGAGGACACAGACTTAGAAACAGAACACACTGATAATGCTCCCTTCAATCCACAAAGAGCTACCTCACCTGATGCTACTCCACAAATACCTACTGTCACCCCCTCACCTCTTTCTTCACCACAACCTGAGCCTATTCCTAGAAGGTCAAACAGAACTCATAACCCTCCATCTTGGCTTCAAGATTATGTCACTCCTCAGTCCATCTCAAACCTTGCTTCTGCTACTATCAAACCTCAATTCTACTCCTTCATGTCCACTCTCACCTCTCAAACAGAACCTACCTCTTTCAAAGTGGCTGTTACACAAGCACAGTGGGTTAAGGCTATGAATACTGAACTTGAGGCTTTAGAACTCAATGATACTTGGGATATCACTCCACTTCCCCAAGGCAAAACAGCCATTGGTTGCAAATGGTTATATAAGATCAAGTACAAACAAGATGGTTCAATTGAAAGGTACAAGTCTAGGCTTGTTATACTTGGGTGTAGGCAGAAATTTGGAGAAGATTATGGGGAGACTTTTGCCCCTGTAGCTAAAATGGCTACAGTCAGAACAGTATTGGCAGTGGCTGCCCTCATGAACTGGTATACATTCCAAATGGATGTGACCAATGCTTTTTTGCATGGAGACTTATATGAGAATGTTTATATGAAGTTTCCTCAAGGCTACACTGGGCTTGGCAGTAGAATCATTCTTAATTCTGTCCCAGATCCTTCTCACCTGGTGTGTAAACTGAAAAAATCACTATATGGCCTCAAGCAAGCACCAAGGCAATGGTTCTCAAAGTTGTCTACCACTCTTCTCAAATTTGGTTATACACAATCTAAGGCTGATTACAGTCTCTTCATCAAATCTGATGCAACCACTATCACTCTAATTCTCATATATGTTGATGACTTATTGGTGGCTGGAAATTGCACTCAAAGCATCAACTCTCTCAAGCAATTCCTCTCTCACAACTTCCACATGAAAGATCTTGGCTCTCTTACATATTTCCTTGGGCTGGAAATTGATAGAACAAGTGATGGTTTCTTTGTCTCTCAAAAGAAATATGCAATGGACCTTCTCAAAGAGTATGGCATGCTTCATGCTAAACCACTTTCACTCCCTATGGATACTCATCTGAAACTCACTCCTGATAAGGGTGAACCTCTCCCTTCTCCAACTCCATACCAGCAATTACTTGGTAAACTGATTTACCTAACTGTCACTAGGCCTGATATTGCCTTCACAGTGCAGCTTTTGAGTCAATTTATGCATCAGCCAACCTCCATTCATATGCAAACAGCCAAAAGGTTACTCAGATATCTTGCTGGCACTTATTCTCAAGGTATCCTCTTAGCAACACAGTCTGCTGCTGCACTTACAGCCTACTGTGACTCAGACTGGGCAAGTTGCCCTACTTCTAGAAGGTCAACCTCAGGCTACTGCATCTTGCTTGGTCACTCACCCATCTCTTGGAAAACAAAGAAACAATCTGTGGTGGCTCGATCCTCTGCTGAAGCTGAGTACAGGGCAATGGCTCTCACTACATGTGAAGTGTCATGGCTGCACACCCTACTGCAAGACTTGGGCATATCCAACTTGCCTCCAACTATACTCAGATGTGATAATCAGGCAGCAATTGCCATTGCGGCTAATCCTGTACTACATGAAAGAACCAAGCATTTGGAAGTCGATTGTCACTTTATCCGGGATAAGATTAAAGCTGGAATCATTAAGACTGAACATGTCTCATCTCAAGAACAAGTTGCCGACATTCTGACCAAGGTTCTACCTGTGAAACAACATCAAAATCTGCTTGTCAAGCTGGGAGCTTCACCTTCTTTGGCTCACTCTCAGCTTGAAGGGGAGTAATAAGGGGAAAACAAGCATCTGTTTTTGTTTGTTACTTAGCTGCTAATTATCTTTAATTCAGTATTAGCTTTTAATCAATCCTCCATGTACTGATTACATGTGAACATCAGAACATGTGACTAGTGGTGGTGGTTTGTTAGGTAGGAATCTTCCACCTCACTCATTCATTCATTGAATGTATTGATAAAATAAGGAAGTGTAGGTGTCTATTTTGTCTTCTTGTGCCTTTATATATGGAGGTTGTACTGTCGGTGAACGGTGGGATTCAATTAATCAAAAAATTCCTTCATC

*S.maritima*347872

CTTTGATTTTTTTTTTTTTTTGTACGTTTCCTCCTCTCCTCGTACAGAATTTTTGACACACTGAAAATCTGTGCCTTGAGCTCCTCATTTCTAATACTTTGGAATAAGATCTTCCTTCAATTCGGGCCGCATTTCATCACAACAGCGGAACTAGAACATCGGAAGTCGTATATATATTCAGGAGATTACATCCAATGTTAGATAGTTATATGCCAATCAATCAAAATGACCATTGTGCTAAATGTCTTGTGGGAACCATATATAACACATCTTGGATGGATTCTCCCATCCTGGCCTGAAATGCGCAGCAAATTATCAATATCATCACACTAACTAACTCATCAACATGTTGTCTTGTCTCGATCTCCAAAAGATCGAAAACTAGAGAAGACTGGTGGATCTGTGGATGGATTGCACTATATGTAAACCTCCCAAAACTTCACAAGTTGATTTTTACAGCATTTCATGCATCATGCATCCGTTTTATATCTC

*S.maritima*26301

ATCATCATCATCATCATCATCACACAACAAAGATAATACTAAGCAACATTCCAAAGAAATGTCAAGGGCTTCCCTCAATCCTAAACCTAGTGGCCACTTCTCGGGCCCCGTTGAAGGAGCCGTGGCTGGTGCCGGGCCACGGCACCAGCATCGGTTTTCCTCATCGGGGCCGCTTACGGGTCTGCTTCAACACAAGAAGTTGAACGAAAAGAAGAAGAAGAAGATGATGAAGCAGAAGCTTTACAAACTGAAACATTAATTCTAGGGTTTCCAATTAATGGCGGGAATTTCAAATTAGAGAAATTGGGGGTTGATTTTAGGGGAAAACAACGGATATGAGGATGAGGAGAGATTGATAACGAAGAAAGAGAAGTCGAAATTGCCATTTTTGTGCAAAAGATTAAGCAGAATTACAAGAAAATTTGAAGTGATTAATAGTTACGAGTAGTTGTTTATCAAAGTTAAGTATCAAAGTAAGCAAAATTAATTTGATCGTGTAATCGTG

*S.maritima*32405

TTACAAAAAGTATAACCAAGTTGCAAAAATTTAACACCTACTGCAACTGAATTCAGTTATGGGTAGAATTGGTACAAAGCTACGTAACTTTCAATAGAAAAGTCACTCTACACAAATCGACAGCCAATATACAAGAGATTGTAGATGGTAATGGGAGCAAGTATGTAATCCAAGATTAAAGGGGGTAGGAGCAAGCCTTCGAAAATTCGCCCCCGCCAAAAAATAAGATGCAAATACTAAAATTAGCTGTCAAAAAAAAAAAAGAAAGAAAAGAATTTCCCACTTTTTGAAGTTGTGAAAGAACAAACTTTTTGAGGTTAGATGTTAGAGATAGAAGATGGGTTTTTATGGGGAATTTTGAAATGAACTTAAATAATAATGGTGGGGTTAGACAAAGGAAGAACAAAGGGATCTAAGTAGAATCACCAGAAGAATAAACTTGATATTTAAATTGATACGTATTAATTTTTATTTTATTTTTTTATACAAGATGAAGTGATGTTTAAAATTAAATGAAAAATGTTTCCAGTTTTTTGAGGAAGAAAGAAAGAAAATAATGGAGAAGATTGATTTGAGCAAAAACAAAGGTATCAAAGTCGGGCCGTCGCGTAACACAAACACAAACGAATCTTTTCTTTTTCTTTTTTTTTTTTTCTGGAAATGGGGAAGAAATTAGCGCCCTTTTGTTTGTTGCTGTTGTTGTTTTTGATTGAAGTTTCTTTGGTTTTTGCTAGTGGTCGAAATATTCAGCAAGTGAATATATGGCCAATGCCAAAATATTTTAGCAATGGTGATAAAGTGCTTTATATAAGCAATGATTTTGAGCTGAAAACTAAGTACGGTGATAAATTAGGGATTTTGAAAGATGGTTTTTTGAGAATTAAGGATATTGTAACCTTAGATCATGTCATTGATGCTAATTCTTCGCGGTTGGACCAATCAGCTTTCATCAAAGGAATTAATGTGGTTATTCAATCACCAAATGATGAGCTGCAATATGGGGTTGACGAATCATACAACTTAACTGTCCCTGCTTCTGGGAAGCCTTCCTATGCACACATTCAGGCAAACTCAGTGTATGGAGCTTTACATGGACTCCAGACATTCAGCCAGCTTTGTGTTTATAACTTCAAATCTAGGCTAATAGAAATTAGTATGGTTCCATGGAAAGTTTTTGATCAACCTAGATTTGCTTATCGTGGGTTGCTGATAGATACATCGCGACACTATCTACCTGTGCCTGTGATAAAGAAAGTCATTGATTCCATGTCATACGCAAAGCTGAATGTGCTGCATTGGCATATTGTAGATTCACAGTCTTTTCCTTTAGAGATTCCTTCATATCCAAAGCTTTGGGATGGTGCATATTCTACGTCGGAACGATATGCAATGGCTGATGCTGCCGAAATTGTGAGATATGCTCAAAGACGGGGAGTGCATGTACTTGCTGAACTTGATGTTCCTGGGCATGCTGGTTCATGGGGTGTTGGTTATCCTTTGCTGTGGCCATCAGCAAGCTGTAGGGAGCCACTTGATGTGAGCAACGAATTTACCTTCCAAGTTATAGATGGGATTCTTTCAGATTTCAGCAAGGTCTTCAAATATAAATTTGTTCATTTAGGAGGTGATGAAGTTGATACAGGTTGTTGGGAATCAACTCATCGTGTGAGTAGATGGTTGAACACACACAAAATGATCGGCTCTCAAGCTTACCAATACTTTGTTCTGAGAGCTCAAAAAATTGCATTATCCCATGGATACGAGATCATCAACTGGGAGGAAACCTTCAACAAATTTGGTGACAAATTGGATCGTAAGACCGTGGTCCATAACTGGCTAGGGGGTGGGGTTGCCGAGAAGGTGACTGCAGCTGGATTAAGGTGCATTGTAAGCAACCAAGACAAGTGGTATTTGGACCATTTAGACACCACATGGGATGAGTTCTACATGAATGAACCTCTGAATAATATTACAAATCCCAAGCAGCAAAATTTAGTTATGGGAGGTGAAGTTTGCATGTGGGGTGAGCACATTGATGCGTCTGATATCGAACAAACCATATGGCCGCGTGCTGCTGCAGCTGCAGAGCGGTTGTGGACATCTTTTGACAAGATAGCTAGGAATCCAAGACAAGTAACTCGAAGATTAGCGCACTTCAGGTGTCTGCTAAATCAAAGGGGAGTTGCAGCTGCTCCATTGGCTGGCCCTGGTAGGAGTGCTCCTGATGAACCGGGCTCATGTTATGTTCAGTAATGTAACGAATAAACTTGTCATACTCATCAAGTATAGCGAAACATAAACATCATTATAGGTTACCTTGTGAATAATTTATCAATCAAAATAGGCATTGTTGCGTTCACTAGAAACTAGATATTAAGCCTTGATTCCCCTCAAAAAAAAAAAAGATATTAAGCCTTGATCATCTATGTCTTATTCCTAGAACTGATATGACCAATTGTCATATATGTTATGTTGGCGAATTTTGCTATAAGAGAGATTCAGATTTGATTTCGGA

*S.maritima*32882

TGAAATCGAAGTTTTATAATTGAATAGAATATAGAATAGAATCTCTTACAAGTATTATTAATTTGAATAATAAAAGTGGAAATTAATAAAATCAACTACAGCAGAAATACAAATGTGAATAAGTAGTCGTCAAATAAATATGATAATTAAGCAACTAACATAAAGTTATTAATAATGTAACGTAAATGTCATTGCAACTGCTAGGCCACCAACAAGAATTGTAGCTAGACTCAATTGTGTGCCCGAGGCTCCATTTTTCCCTGCTGAAGCGTCTGCTCCTTCCGGGGACATCCCCGCTGGTGATGACGAAGAAGGTCCTAAGCTCGGTCCTTCAGTCGAAAGTCCACCACTAGATTCTGGTGCTCCTGCTGGTGCTCCTACAGGTATTCCCACAGCTGAACATAAGCTAGGATCAGGAGCTTGAAGCTTGCAAGCAGTAGGCATTTTAAGAGCTCTCTTCATATCCAACTCCAAACCAAAATCCTTACTTTTTCCAAGCAATTGACACAAACAAATTGGGTTTGAATCCAACATTCCAGCAATTTCTGGGCAACAATTTTTATCAGGTTTTGTTAAATTACTCCCTTTTTCAACATATGTCAAACAATCTGACATATTATACAATGCAGTCATGCAATCATCAGCCGTTGATGCTCCGGGCCCCGGAGCGGGCGCTGCTGATTGCGCCTTTACTTTAAAACTTCCTACTGAAATCAACACCATTATCACAACTACCCACATGCTCATTTTCGATGAAATCCCCATTTTCCTCTGTTTTTTTTTGTTGGGGGGGGGTTTGCTCTGTTTTTACTCTGTTTTTTTGGAGGGAATTCCTCTGTTTTTGCTCTGTTTTTTTTGGTGAGTTTTTTTTTAGAGAGGTGGGAGTGTAAATGATGTGGGTGTGGATTTATGAATGTGGGTGTATGTTGGTATTTTTAAAGGGAGTATATTACTAATTTATTAAGAAAAATATGGTGTGAATTTAAGGAAATTTGATTGATGAAAGGTTTAATAAATGTGAAACCTAACTACTACTAACTATCTTAATTGTTTTGGAATTCAATGTGCTCACCTCACTCTTGCGGTATTTTGCCTCTTCTATCAAAGCCAAAAGATGCCGTGATTATGTCTAACTTTTCC

*S.maritima*42135

ATTTAATTAAATAAAGCTTCAATCTTTCAATTCCATATTTTCCCAGATTCTTTATTTTGTTTAGTTGTACTTGGAATTGGTGAAATATAATTATTTATATATAAATCACTAGTTTATAAAATTGGTGATAAAAAAACAAGGATTTCTTCTGGTTTTTTTTGTTGTTTTGAGAAGAGGACTTCTTACCCTCAGAAGTTCTCTCATCCACTGTGGCATCCTTATCTCCATATCACTAAAATCTTACAATCACACACTCTTCTATCTTTCTCTTTCTATCTCCTCTTACACAAATTCGTCTATTTTTAACCCTTTATTATATTTCTCCAATTTCTTGCTTTCTCTACTTTCTTCCCAATTTGTGAACAAATTGGGGGAAAAAAGGGAAGTCATTGGTTTTTGATTCTGGGGTTTGGAGCTAACTTCTTGAATTTCATGAATTAAACCCCAATTCTTGATTTGGGGGAAAATTGAGTGTTAAAGTCTTGATCTTTTATCGAAATTAGTATCTGGGTTTTGCTTAATTCGTTGGAATTTGGGTTGAAATGGGATTAATTGAAGAATTTTGGGGGATTTTAGAGAAACCCACATCAATTAGAGGTGGATTGATAGTAGAGACTATGTTGTTTATAATTCCAATTTGGACTGCCTTTCTTCTTGGTATTGTAATTGGATGGATTTGGAGACCAAAGTGGTGGCAAACTTTAGATAAGAACAAGTTTGAATCTTTGATGAACAAAATAGTGGAATTTTCAGCTCCTTCTTCTCCTTCAAAAGGGTTTCCTTCTATTCAGAGTTTTACTCCTTTTTCCATGAAATTCTCCAGCTCCAAAGGTCAAGCAACTGGGGATGATATTGGTGCTAATAAAGAGATTGTTTCTTTGCAATCTAGTTTTGGTGATTCCAGTTGCAGGTCATCAGAGCTAAATGGTGAGGGGCATCCTGGTGTAAATAGAGGTGATTTGGTACATCTTTATAAACTTGTTGAAGAGAAAGATGGAGGTCCTTCTTGGATTCAAATGATGGACAAATCCACCCCAACCATGCGCTATCGAGCTTGGCGGCGAGACCCTGAGACTGGTCCCCCTCAATATCGTAGTAGTACTATCTTTGAAGATGCAACACCCGAGATGGTGAGAGACTTCTTTTGGGATGATGATTTTCGTCCAAAGTGGGACACCATGCTCATACAATCAAACATTATTGAAGAGTCTCGCACCACTGGAACCATGACTGTGCAATGGGTTCGAAAGTTTCCATTTTTCTGCAGTGATCGAGAGTACATCATTGGCCGTAGGATATGGGAGTCCGACAGAACATATTACTGTGTGACAAAGGGAGTACCTAATACTGTGCCAAGAAAGGAGAAGCCAAGACGTGTGGACCTGTATTATTCAAGCTGGTGCATTCGCCCAGCGGAATCAAGAAGAGGGAATGGGCAGATGACTGCATGTGAAGTGCTTCTCTTTCATCATGAAGACATGGGTATTCCATGGGAATTAGCTAAACTTGGTGTCCGACAAGGAATGTGGGGAGCTGTTAAGAAGATCGACCCTGGTCTACGGGCTTACCAGAAAGTAAGAGCTGCTGGTGGGCCACTCTCACCCACAGCATCCATTGCCCAAATCACCACAAAAGTGGACTCAAACGATTTGCAATCTATGTTGAACGGTGATTATAATCAGTCAGTGACCGAAGTAGCTCCTTCAGAGGAAAAACAACAAAAAGGGATGAATGTGCCAAAGCTCCTCATCTTTGGTGGTGCAGTTGCTTTTGCATGTAGTCTTGACCGAGGGCTTCTAACAAAGGCAGTGATCTTTGGAGTTGCACGAAGACTCGGAAGAATAGGAGGAAAGTTATAACTAGAGGTCCATCATCAAAATATAGAACACCCTTGCATACTGGAGTCGTAACCTTCGTGTCACTGCACCCATGTAGGAAAACAACCCGAAACAACCCTTGAAATTAAAAAGTGAAATTCAGAAATGTAAGCTTCACCACTTGACAAGGCGAAAATCATAAGGAGTTTTGCATGTTGTAACAGTTGTGTAGTTGAAGAAAGCAGATCATTAAGCTGTATATCTGCAAAAGATTTGCTCTTTCTTTCTTTCTTCAATTTATTGCTGCTAGATGAGTAGCCATTTCTTATTTACTTAAGCCTCAATAAATTTTCATTTCATTATTTAATAATTTACAGGAATATTCCAGTGAATAATTATTCTTCATAAATTTTTGTGGGTGTCATTTTGATTTTCTCTGAATTCTGTGAGATATGATATTGATTGGAAAATTTGTCTCAGGGATAATAA

*S.maritima*12177

TTTAATTTTGTTTTTCTTTTTTGTTGAAAATTCATGAATTTTCTCGTTTGTTGATCAATTCTCTTCATCAATCTCTCAAAATTTGCAGCTTTGCTTCAATTATGGAAGATCCTACTATTCAGTTACACCCTCTTCAGAATTCCAGGAAGAGGAAGTTTGCAAGTAGTGGTAGCAGTAGCAATTCGTCAATGGTGGAACCTGATTTGATTGAAATTCCACCTCCTGCTACTTGGAAATCAAAACCCCAGAAACAGAAATCGGTTGCGGGTTGCGAGCTGATTGTTCTTGATGATACTGAAGATACTGATGATGTTATTGTTATTGATGAGAAAATTGCTCCCAAATCTAAAGGAAAGAAACCTATGCATAACTATCTTTCAGGAGATAGCATTCCAGCCAACAATGGACCTCTTTCTGCATCAGTGAAAGATTCTATGAAAGGCGCTTCTAGTTCCGGTGTATCTTTCATTGATGACAGCTTTGATTTTGCTGGGATGGATCTTGCTGGGAGTCATTATGATTCAGAGTGTGCATTGCTGCAAGCCCATTTTGATTCTATTGACATTCCATCTGGAGTAGAGGCGACAGTCCCTTGGTGGGCATTCTCTCCTGAAATCATTGAACCTCCTGTTATTCCAAGTAGTTCTGCTGCAATTACCAATCAAAGCAACTCTTTTGTTGATACTTCTGAAGTGGATGGGTTTCAATCATGGGCATACTCCCCAGGGATCGTTGAACATTCTGTTATTCCAAGTAGTTCTGTTGGAATTAGCAATCATAGTAACTCTGTTGTCAAAACTTCTGAAGTGGATAGGTTTCAACCATTGGGTAAAGTGAAACCCGTATCGTTTAAAAAGAAAACTGCATCCAAAAAGTTTTTGCATGAATCAAAGCATCCTTTTTCTGGCCATGCAACTTATCCTATGCCCTATGCCCAAACTAGTGGTATGTTTCCGCCACACAATGGGTGGCCATCGGTTCCTGGTCAGGGCCCCAATTTTGCTTTTGACCACGATCCTTTTGGAAGTAAAGGTTTCCCAGGTTGGCAAAATGCTTCTCATGTTTATCCCAGTCCGGTGCCATTCTTGTCTTCTGTAGCAAGCTCTAATTCCATTAGTAAACTATCCAATGGCAATATGCATGTTCCTCAATTTCTTGATCCTGTAAGGCTTGATGATGGTATTTCTTCACAAATTTCACCACCTTTTTCCCAAGAACAAGGAGATGGTAGTGAAAATTCCATTGTTAAGAAGCTGGAAGTTTTTAAGAAATTTGATACTTTAGAAGATCATTCAGATCATTTTTTCTTGAAAAATAAAGAATCTGGTAGTCAGGCCAAAAACAGCTGGTTGAGAAAATTAGCGGATGAATGGAAAATGCTGGAAAGAGATTTGCCAGGTACAATATATGTCAGGGTTTATGAAGCAAGGATGGATTTGTTGAGGGCTGTAATTGTGGGAGCGGATGGTACTCCCTATCATGACGGTCTCTTCTTCTTTGATGTCCATTTCCCACCCACATATCCACATAAGGCACCTTCCGTGAACTACCATGCACATGGCCTTCGACTTAACCCTAATTTGTATAATTGTGGAAAAGTATGCCTAAGTCTACTAGGCACTTGGAGTGGTAGTGGTGTAGAGAATTGGCTTCCTGGCAAGTCAAATATGCTACAAGTGCTCCTTTCAATACAAGGGCTCATCTTAAACGCTGAGCCTTACTATAATGAGCCTGGTTTTGCAAACTCTAAAGGTAACCAACATGGCCGACAGCAGTCTAAGAATTACAGTGAAAACACGTTTCTCTTGTCGCTCAAAACTATGCTCTACACAATGCGAAATCCTCCAAAGCATTTTGAGGATTTGGTTTTTGGCCATTTCTATACGCGTGCGCATGATATACTGGGAGCATGTAGAGCATATCTGTGTGGTGTTGAGGTTGGGAGTTATGAAAAAAGCAAACCAGCTGCCCAGTCAAAGGGCTATAACTGTTCACAGTTGCTCTTAAAGAATTTACCAACTTATATCAAATCACTTGTTGAAGCTTTTACAAAGATTGGTGTTAAAGACTGCGAGAAATTTTTAGTCCCTGTTGAAAACGTTGTTAACCAACCAGCCAGAGGTAGTTCTGCGTCTCGTGCAAAGGGGCGCAAGTTTTTGGCATCATACGTGCCGCCATGGAACCATGTATAGGGGCTGAAATGGAAATGCAGCATTGGCAAACCCTTTTAATGAAGACATGCTGCTTGATCAAAATTATCTTGGTATATATATTCATACTAATCCTTGACTTATGCTAGTATTTTGATGACATTGTATATAGGCGGAAGTAAATGCTGAAGCTTTTGACTAGATTAATTGAAAGAGCAAGAAGGGTTTCATAGAGCTTCTTGTTTTTTGTAATCAACATTTTTTAGCGGATGTCGCCTTTAATCGATTTTTTATACATCAGTTAATCAATGATTTTGATTTAATTACTAAGTTACTATTGCTATTGAGCTGTGTTGTGCTGTGCTCAGGCTCGGGTAGTTGTTTGTG

*S.maritima*118987

CATTAGTCTCATCTCTTTTTGTTGCTATATATAGGTATTTGGAATATGGAAGCACTTCAGCCTACGAGGAGTAGAGCAAGTGTTGATACATTCTGATTGTAAGGATGAAGGTTTTGTCAACTCAGATGAATATAATGACGAAGTGATTACTAATAATAAGATAACTTGGCAGCCAACATCGGAAGCTTGGGAGTTCTTTGATAAAAATGCTGAAAATGGTGTTGCAAAAGCAAAATGCAAGCATTGTAATGCAATACGTTCGGCTACCGTTGGAAGTAGTACTTCGCATTTGCTTAAATATGCAAAGGAAGGCTTGCCCCGATGACATTTGAGGCTTGCACCTTTCATAACACACGTCCTTCCGCAGCTACACTGTTGCAGAAAATTAGCTGCTTGAAATTCTCAAGAATAGTAGAATCCACAACAAGTGAATTAAAGTAATTTTCAATTTCACCATTAGCTTCTGAAATCGCATGATTTGAGTGAGCTTGGAATGCACCAGCCTCATCATATGCAGATATTGTGCTCACATTTCTACAAGCTCTACCAACAAGATTCAAATTCTGCTGCTGCGGCCCATCTTTTTCATCAAACAGGACATTTGTCCCATTTTACAGTTTTTCATCCATTTCCAATCAGCATCTTGTGTGTTGGAATTAGTAACACTTGCACCATTGTTTCCACTAAGAAATCCATTACCAAATAATACTTCTGAAGTGCTATCGAGTCCACCAAGTTTCCTGACTAAATGCTGCACACGTGCATTGTGTAATTCACATCCTTGTCCTACATCATTCAGTTTTGGGGGAAAGCGGCGCACATTCAAGTCTATCATCTTCATCACAACTAGTGTTGCTATCACAGGAAGCAGCAGAAAGGAACTCAGCTGATAACATCAAAATCTCTTCCTCTAAGGAGTAGCGACGGTAGAGAATATCTTCCTCATAATGTGGAGGTGATACAGAGGGTGCAGAGGATGATATTAACTTCTTCAAACCTTTAACGGTGGTCAATGCCGCACCACTGAGACTAGCATTTATCTCCTCCTCACCATACTGTACAATTGTAGCAGAAGTGGCTGAGTTACAGTCTGGATTTTGCAGATATGTTTTCCCTTTGGTGAAATGGAACTTCAGATTATCCTCATCTAAAGCCTTTGCAGTTATTCTAAGCTTCTTTTCCCCATTATAGCCACATTTTCCAATAAAAGAAGCAACGGAACTATTAGTTTCTTGAGCATTTCTATTACTATCATCTCCAGATATCGTAATAGAGTCTGATTCATATTCCATGCTTCCAGAACACTCTCCAGAATGTCTCCAACTAGATATGTCTCCTAAATTGATGTTACCAAAAGTTGGGCTACTCTTATTTTCATCAACCCATTTCTCAGATCCTTGATCTATCCAACCCTTGAATTCCTCCAGCCATAATTTAGAACGTTCTTTCTTCATCAACTCAATTCTGTTAATTAAAGTAGTGAGTTCACCTTCATTATCAGAAACATCCTCCTCCCCACCTTGAATATCATTACATGTCACTAAATCTTGCTCATAGACCATATTCATCCCAGGTTCATCACTTTCTATACAAGCAAGACGAGAGAATTTCTTCCTTTTTCCATTTATAAACCCTTCCACATCATACTCTTCCCTAGCAGGACAGTAAAATCCATAGCTTGTAGGCCGTTTTTGCCTCCTTGCAATGATTATCTGCCTTTTCCAGAACTCACTTGTATTCATTTTTTTATCATCCAATTTCATTTGCTCAGGATAAAGAAAAAAGCTGAAAACTTGAGGCCGATACCATCGAGTACAACAAAGAGGATTCCCTTCAAGCCAAATATTCTTAATAGATGCAAGGTTTGAAAAAACCTCTAGCTCCACATAGTTGGAGATAACGTTGTAAGAAACATCAAGTTCTTCAATAGATTTCAATTTCTCAATCCCTCGCAATGTAGTCAATGCATTATTCCTTAAAACAAGTTTTAATATATGGCTTGTGACCTCACTCAATGATGCAACTGATCGAAGTTGATTAAATCCGAGATCAAGATGTCTCAATCTTGTGCACTTCCGAAGATTATCTGCTTTTGCAAACTTATTTCTGCTTACATCAAGGGTTTCAACAGCTGGAAGAAGCTGCAAGGACTCATCCATAAGAATCAAACCATTGCATGCACAAGATATAAACGATAGCCGGTTCCATTGGGAAGATTCCTTAATGTCGGCAATTCTACTAGCAAACACGTGGCGCAATGCATCAGTAGAATTATGACAAATAAGTTTCTCCAAAGTGTTTGTCAACTGAAGCAGACCCTTGGCAGCGGAAGTGGAAAGATCACAACCACGAAGCTCCAAAACCTTCAATCTTGCAAAAGTAAGCAACGACAACGGGGTGGGATCCCTAGAAGGGAATGGGGGCAAGACTGATACAATTTTAAGCGACGTAAGACGGCATAAAATCCTCCGAAGCTGTTCCAAAGCACGGTAATCACCGAGATCGGAGACGTAAGCACGAAGATAATCAACGGGAGCTCCAGCGATTAGACCCTCCAATTCAGAAAGTGATTCTAATCTTGATTGGACGTAGTGTAATCCCACTGGATTCAGCTTCAGAATTAGGGTTCCTTCCATTAATCCGCTGGTGTTATTCTCCACGAATTTTACTAGAGATTCAAGGTAACGATCTCCTGTTACAACCTCCATTGATTCAAACAATTCTTCTGAAGGTTTGTTTGTTTGTTTGTT

*S.maritima*12826

CTTTAAATCAATGGCCCAAACAAACAAAACCACCATCATTTCTTCACCCAAAAGCATATCATTTCTCCATAAAATTTAAAAAAGAAAAGATAAGATAACTCATCCACCCAACTACACCTCCATTCAATTCAAACACCATGACCAAAATCCTACACTCCCAAATTTTCAATTCACCATTTTTCCAAACTTCAATCTCAAAACCTCAATCTCATTCACCAATTTCACCATCAATTTCAATTCTTTCTCCAATTCGAAGCACCAATTTTAAAATCCAACAAAACAACAATGATAATGAAAATCTTCAATCACCTTCATCAATCACACCCCCAGAAACTGTTGAAATTAGGTTTCGAAGAGGTTCTAGAAGACGGCGAAAACAACAACAAGATGAACAAAATTCTGGCGATGAATCAACGATGAAGATGAGTGAAAAAACCCCAATTAAGAAAAATTGGGAAGAAATGAGTGTGTCTGAAAAAGCAATAGAATTGTATATGGGTGAAAAGGGGTTTCTTTTTTGGGTTAATAAATTTGCTTATGCTTCGATTTTTATTATAATTGGAGGTTGGATATTGTTTAGATTTGTGGGTCCTTCTTTGAATCTTTATCAATTGGATACACCTCCACTTTCTCCTGAATCTGTGTTTAAGGGTTCTTGATTTTGAGTTTTGTTTGATTAATGGAAATTATATTATATTATAATGAGAAATTTGGTGTATTAATGATTTGTGTTTGTGTTTGTGTTTTTGGGTTATATGACGTTTGACAGGACAAAAGGGCGTGATGG

*S.maritima*41751

CTATTAGCAAGGTTGTCCAAAGCCCACTTTAAGTCTTAACCCAGCCCAAAAAAGTCTAATCATTCCAGAAACTCCTCAACTCGGCGAAAACAATAGTCACATAAAAAAGAGGAGAAAGAAAACAAAGCTCCAAAAAACAATAAACCACAAAAATGGCGATTGCTTCAGCATCTTCTTCATTGTTGAAAGCTCTTCTCGATCCAAAGAAGAACTTCTTAGCTGCTATGCACAAGAGCTCTGTTGATCATCGTCTTCGCAAATATGGTTTGAGATTTGATGATTTGTATGATCCAATGGAAGATCTGGACATCAAAGAAGCCTTAGATAGACTTCCTCGTGAGATTGTTGATGCTCGTAATCAGCGTCTTAAGCGTGCTATTGATCTTTCTATGAAGCATGAGTATCTCTCTGATGAACTTCAGGCAATGCAAACACCTTTCAGGAGCTACATGCAAGATATGCTGGCTCTTGTGAAACGTGAAAGAGAAGAGCGTGAATCTTTGGGAGCTTTGCCTCTGTACCAGCGAACACTTCCTTAAGCTTTTCATTGCAATTTGCAGCTGGCTGATTTATGAACTATTGCATATTGGGGGGTTTTTGAGGTTATTCTGTTCTGTAAGAAAGGCAGTTAGTATAATAGCAATGAGAGACTTGCTTGTTTCTCTCCCTTTTGTGCACAATTCATCTTTTGTTTTACTCTTGATCTTGCTCATTCATAAGCATTATTTTGGATTAGGTGATGCTTCAAATGATCAATAATGTACTCAAATTATGCTTTAAAACTTGAATACATAGTCAATTGACTTTGTGTTATTTCATCTACAGAGAATAAGTTTCCTTAACTGGCGACAGCAACAGTTC

*S.maritima*45469

CCAAGGCTATCAAAATGGGAGATAGTGTGACACTCAAAGGTGCATCCAATTAGAGAGAGGTGTGTAAGCCTTGAGAATGTAGGAGGAAGGTGATGGGGGATATATCCATTGGAGATATAGAGGTGGGTGAGCTGTGGTAGCTCCCCAATGGAATCTGGTATTGTCCCTTTATACATCCAGTTCCCTAAATCCAAATCCAGAACTCGGAGGGATGAATGTAATCCATAGTTATTATAATCACGAATCCATAGTGTTGGATCCCCTTTGAATTGGAGGTATCCATGAGTGGAGGTTTTCTGAGTTGCCAATTCCTCTGTAATTATTGGGATTTCTGTTATTTGAGTTTTGAGCTCCTTCCAGGTTTTACGAATGTGCGTCTTCTTAACAGAAATGATAATGGAGTCATTCAAATAAGTTGGCAGCGTTTCTAGTCTAAATTCTTGAGGAAGGTATAGAAGTCGCAAATTGGAGAGCTTGGTGATACTCCAAGGAAGGCATCTTAATCTGTAGCAACAATCTAGAAAGAGAGATGCAAGGAACTTCAAGTCACCCAAATTTGCAGGAAGTTCTTCCGGTATCTCATCACCATAGATGATTAAAGTTGTGAGGTTGGACAGCTTTGTTACACTCTCAATAACATAGTTCTCACACTCATAGAGAGAGAGAGATTTAAGGTTGTACAGGCCACCCAAATGTGGTGGTAATTCTTCCAGTTCACTGATGAATAAAGTTGTGAGGTTAGACAGCTTTGCTACACTTTCACATAATTTTTTGTGACACGGCCTGAGATAGAGATATGTAAGGTTGTATAAGTCACCGAAATTAGGGGGTAAATCTTCCAGTTTATCACAACCATACAACTCCAAGTGATGCAAATTGTGGAGCCTTGAGAGGGTGTTAGGCAGGCACTTGAGATGACATCCATGCCGGATAGAGAGGTGAGTGAGCTGTGACAGCTCCCCAATGGAATCTCCTATTCTCTCTCCTTTATAACCCCAAAGATACAACTCCAAGGATCGCACTGATGACACAATATCAGGGTTACTTTGAATCCAAACCCCTGGATCATCCTCCAAATACAGGAACCCATCATCATATAACCTCCCAACCTCAACCGTCTTCCTTCTCACCACCGACCACTCCTCATATTGAACAAGAGATGGCTGAATAATTTCCATTTCCCAGCACAAGCAACACCACCACGCTCCCCTCCCTTAGTTAATTAATTAATTTAATAAAGATTAGCAGATTGCCAAATTGCTGTTTGGAGGATTTATTTTAATGAAAACCCAATCAAACAACCAAAAACTCTCATTTAAATACAGAAAGAAAATCAATCTCAACCTCAATCTTTTAGTCTTTGGAAATCAACTAGTATACCACACCTCAGCCAACAATGTTTCTAAAAAAAA

*S.maritima*14478

CTCAAACTCAAACCTTAAAAAAAAAATCACACACCTCAAACTTTCTATCTCATCATTTATTTATCTTCCAAATTTCCAGTTAAGAAAGATGAATTCAAAGCCATTTAAAGGTTCAAATGTGTTTATGTCAAGAAATTTAGTTCCTCCTGAAATCTTTGATTCTTTAATTGATGCCCTAAATCACAATGGTGCTATTGTCTTCCATTGTTGCGATCCTTCTCGCAACTCCTCCAACGATTTTCATGTCATCTCTTCTCCTCAACATGAAAAATTCGAGGATTTGCGAGCTAAAGGATGCAATTTACTCGGTCCACAATGTGTTCTGTCATGTGCAAAAGAGAGGCGAGTGCTTCCTAAGCTAGGATTTGTTTGTTGTCTTGCCATGGATGGTGTTAAAGTGCTTGCATCTGGCTTCGGAACAGAGGAAAGGGAAAGAATAGGAAAGCTTGTGATTTCTATGAGTGGAAGCATTCTTACTAAGCCTTCCTCAGATGTTAGCTTTGTTGTTGTGAAGGATGTGTTGGCTACAAAGTACAAGTGGGCTGTCAGTAAAGAGAAGCATATAGTAACTACAAATTGGTTGAATCAGTGTTGGATTGAGCATCGTCTTGTTCCTGTGGAGTCATTTCGAGTTCTCCCTTTCTTAGGATTAACTATTTGTGTTACTAGAATCCCTGGAGATGAGCGGAAGGAAATGGAGAAGCTCATCATACAACATGGTGGAAAATACTCTGCTGAACTCACCAAAGTCTGCACACATCTAATTTGTGAAGTTCCTGAAGGTGACAAATATAAGGTTGCTATGAGATGGGGAAATATCTGTATTGTTACTCGAAAATGGTTTGATCAGTCAATTTCTAGAAGAGTATGTCTCAACGAGGATGCCTATCCAGTTCAGAGTGGTTCGATATCTTCAGTTTCTGCCTTAAGGAATAAATTAATAGCCAAGTCTTGCCAGGACAAGTGTACAGGGAAGTCACAATCTAGTTTGTCATCAGCACCCACTGATAAATATTTCCAAGCTGTTCCATCTTTGGGGATTACAGAAACGGATCTGGATCTTGCTTTTTCACAGAACTTATCTTCTGCATGTCAGAGTGGCTCTGCTTTCGTCAACGAGGATGCTGAAAGACAGCCTGATCCATCTGCTAATGACAAGGGGCTGCAAGATTGTGTTGCTGAAGATTCAGAAGATGATGACAATGACTTGTACCTGTCAGATTGCAGAATTTGTCTGGTTGGCTTTAGTGCTTATGATATGCGAAAACTAGTTAATCTTGTGCGTAAAGGTGGGGGGTCCCGATACATGACATGCAGTGAGAGGCTCACACATATAGTTGTTGGAGAGCCATCAGATGCCGAAAAGCGGGAATTAAGAGGCTTGGCTGCTCATGGTGTCATACGTGTTGTTAGACGGAACTGGCTTGAAGATTGTAATCGTGAAAAGAAAGAGGTCCCTGTAATTCAGAAGCACATTGCATTTGATTTACTTCTTTCCAAAGATATGATGAACCTGTCAAGTAAAGCACCAGCTCCTGTCGTGAGCAGCTCCAAACACAAGAATAGCTCAAGCATTCTTTTGAACTCACCAATAGGTTCAACTTCGGGAACTACAGATACAGTGACTGAGATCTCACTGGAGAAAATTAATCTCACAGTTGAAAAAGGAAAGATTTCTCCTAAAAACTTAAAACTTTCTGCAGTTAATAGCAACAACACAAGTCAAAGGAAGATCCAGCGCAGTGATAAATTACAAAATGTCAAGCCTATAAAAGTCTTTACTGGGAAACGTTTTTGTTTCTCACATTCTTTCCCTGATGACCGGAAAGACGAAGTTATTCAATGGATAAATGAAGGTCAAGGTGAAGTGGTTGATGATCCTATCAAAATGGATGCGGATTTCACTGTTGAATGCCACGGTGTGATTACTAAATCAACCAACACTTCTCAAACTACTTATGTGTCAAGTCACTGGATTCGGTCTTGCTTAGAGGATGGAAGCTTGCTTGAGGTTGATAGTCACATACTTTACTCTCCACTTCAGTGTACAGTTCCTTTGCCTGGTTTTGCTGGCTTACGCTTCTGTGTATCACAATATGTGGACAAAGAGCGATTGCTTTTGCGCAACTTGTGCTTCGTCCTTGGTGCTAAATTTGCAGAGAGGCTTACCAGGAAGGTTACACATCTTTTATGTAAATTTGCCAATGGTCCCAAGTATGAAGCTGCCTGCAAATGGGGAGCACATCCTGTTAGGTGTGAGTGGCTTTATGAATGTGTTAGGAAGAATGACATAGTTTTGCTAGATGACTTCCATCCAGAACAAGTTCCTCAAGATCCTGAGTCAGGGCTATGCACTGTTACACAATATCCTACACAAGCTTCTAAACTAATCTCTATGGATGGTTCATCACAGCTCTCAAGTCAGTCTCAGGACTTGCGAAACGCATGCAAATTGCAATCTGGTAAAAGGAAAAGGCTATTGGATTTAGACAGACAATCAGATCAAGATTCTCAAAAGCATAAGAATGCTGTAGCTGTTGAGACTCATGATAATGCTAAAGAAAATGATCATAGTGTTCCTGATGTTGCTGCTGCTATAGAGGACTTGTTGGAGCAAACAAGCAAGATTCAGGATCTAAAGCCACCAGTTGATTCAGAGTGTGACAGAAGTATGTTTTCATCTGACTGTCCAATTCTTGTTAGAGACGATCCAGGCTCTCACTCCGCATTCGGAACTTCTAAGCTTTGGTTAAACAGTTTAGAGAAGGACAATCACGATGCTTCTAGAGATCAGAGCGCAGGCATTCATGATGGTTTTAGCGAAACACAGACAGAGTCGGTCGGTTATGAAGATGATCTATCTGGTAGACAAGAGATTATAAACAGAGTTCTGACCAGAAGTAGCATGACTTAAATTGCTCAGAAGCTTTATGAAAAGGAATGGAGCATTGGCTATATTTCAGCGGGGAGAGAGGATTAAATTGTCGCCTCATGGTATGCATTGGTTTACAGCTGGATTAAGTTTCTCAGCATTCTTTAGAGTGCTGGCTTGATAAATGATAAGGCAATCTACATTTCTACCTTAGACAAAAACTATCAGCAAGTGACAGTAGAACTTTGCAGCAATTGCATCCTGTGACCCTGTGAGCATTGCTTGAAAGCTGGTTGGACCTTGAAGGGGCTGTGCCAAACTGACAACTTAGAGAGAAATGCTACACAATATAACAGCACAAACAACCAGGTGTCAGTTTGCTCCTTTATGTTCCATCTCTCGAAGGACAATTTCCTTAGCTCGAGTGGTGTTCTGTTCTTTGATAGAACGATTTGATTTTTGGCTAATGGTGAGTACAGTTCACAAGAAATCTCCATCTGTCATTTGAGATATTCACATGTAACTGTGATACAAGTATGCTTCTGTTTTGTTGATGGGGAGGAAGTGTTTTAGCTGAAAAGAAAGCATCAAAATAGCATGGTTTAACCTTTAACAGTTTAGAAAACCAATATTCTGATATATGGCACTTCGTTTTTACAGTACTTTTTAGTCAAGGAAATTTTTGTATAGCTTGCATATAGATGTAGTGTTGTGCTGTAAATTTTTGCATTTTTACCTTGTTTAAAAGGAAAGTAAACTCCATTTTTTTTTTTCCGTAATGCAGAAAGGAAACTGAGG

*S.maritima*43199

TTTGGCGATTCAACTTTATTATGAGTAGTCGATGGTGTTTAGTAGTATTGTTAAAGAGGTAATAAAAATATAAAATTGAGTTCCAGAGTTGGAAGGGAGAATGATATACTAATGTGTAACCAGAGAACTTTAATATTATGTTATACTCCCATATGATGAGATGATATACATTGCTTGCGATATTACATTCGAGCTTAACAATTATTATGCACTGAATTTTACAAATAACAATGTAGTAACCAAGTAATAAAGTGGTTAAGTGCTTGATTAATTAGTACACAATGAACACAAACTATCTTCTACAAGGGAAGCAACCAGACTGTTCTAGGAAGACAGTAGCAAAAGGTCGAAAATGGATTAATGCCGATGTAAAGTGATGATGGATTAGAAATAGAATCATCATTCATGGTCATCAAGTAGAAACCAACTGATTTTAGTGTTAACCTATGATTGGATTCACTCTGATCGAATCAAGTTTCTTAACCAAAAGCTTGACAGTGTCGTCCTTCCTTGCATCAAGCAAATCCCTTTTGAGACTGCCGTAAACAGAACCAGGAGGTCTAATGCCAAGATCAACCATTTCTTCGAAAAACCTACACGCATCATCCAATTGTTTTTTGTGCCGAAATGAATTAATCAGTGTAACAAACATTTGCATTCCTGGTAAAACCCCCTTGGCCTTCATCTTATCCCAAACCTTTACTGCCATATTCAGTTCTCCTTTACTACAATACATTCTTATAATCATCTCATATGTACTCGACGTGGGATCACAGCCTTTATCATTACCCATCTTCAAGAACACCGAGTATGCCTCCTCTTCTTTCCCGGCCTTTATCAAGTGATGAAGAATTATATCATATGTTCTTGAATTCGGACCAACCCCACATTTCCTCATCTCATCAATCACTCTAAAAGCATCATCAAATCTGTTAGACCAACAATACGACCCAACCACTGCATTATAAGTAGGTGATTCAGGTACAACACCATTAGCTTTCGCCATCTCAAAAAACTTCAACGCTTCCCCAAGCCTTTTATCCGAGCCTAACCCATTGATCAAGATACAATAGATATGTTGACTAGGCTCAACACCCTTAGCTCCCATCTCACCCAACATCTCCACTGCTTTCGTGACTTGTCTCGATTTACAGTATGCATTCATGATAATCCCATAAGTAATGACATCAGGCTCAAACCCAACTTCCTTCATTTCCCTGTAAACTTCATCAAGCTTCATCAAATTCTTCTGTTCACCCCAACCTTCCAACAGTATAGTATACGATTTTTTATCAGGCACAAATCTTCTGTTTTTCATTTTATCAAACAGTTGGTGTGCACACTCAACTTGCCTAGATTTACACATTGTGTCAAGGAAATTGTTGAAATCTACCACATCCGCCGTCATCCCAAACTGTTCTATCCTCTCAAACGCCGTGATTGCATCATCAACCCTCTTAGCTCGAGCATACCTTCTTACAATAAGCGAAAACGTGTACTTCATCAAAATTCCTTTACTTTTCATTGAATCAACCAACACCCAAATCATTTTAAACTGTTTGATTTTCCCCAGTGAATCAATCAATGCATTGTAAACTTCATTTGTGTGCTGAAACCCCTTTTTCTTTTCTGCCCACCTGAAAAACCCAAGAGCTAAAACTCCAGAATTACTTAACTTCTTCAAAACATCTAAAACCAAAATGGGTGTAATTTCTTCAACCCCACTTATATCTAGAGTTGATTCAATGGTGGAATCTTTTGGTAGTGATAATAATAATTTGCAGATTTTATCCGAATCTTTGATTATTTGATTGGGAATTTTAGAGGAGTTGGGTTCCTCTGTTTCTTTCTTAATTTGGGTTTTCATTGAAGACCCATGAATCAACCTGTAAAATAATTCTGGGTTTGGGTTAATTGATAAGTTTGTTGATGATAAGTATGGTGTTTTGATATTAGTTGCAGAAGAAATGAGCTTTGTTAATGGGAGTAATCGACGAACTAAAATACGAGAAAACCCCATGATAATGAAGCTAGCTTTGTGAGAAAATCAGTAGCAAATACGATGTAAACTGATG

*S.maritima*46987

CAGTTCTTCATCTTCATCTCCATCTCTCGATCGCTCTTATCTCATCAATCATCATCAATGTTTCGTTTCATCAAATAATCAGCTGAAAGCTCGAAACAAACCAGGATATTTTCGCGCAGGAGCACGCATATGTATGATTTTGGAGCACGTAAATGAGTAGCTTATACATATGTTTGACTGTGAATTGCATCTTGCAGTATGGATCGATAAATCAAGTTAATTTCTAATAAGGGTATTGTAGAGCCAATAGGAACTTGGAACTTTAGGGAGTAACACCTTTGACAATTTTTTTGGGACAAAATCAAGGTACACCAACTTAATTCAACTTTACTTTATTCAAACTTCTTTAGCTCTGGCGCCCATTTTGATAGAAAGTTTGATCAAGAAAATCAATATCCAAAACTCAAAATTGTACAAAAATGTTCCCCCGTATATATGAACTATGAAAATTACTACACAGACCAGAGCAGTCAAGACTCAAGTGATCACGTTTTGTGTTATCGATCCTCACATTGCTTTAAACATCCTCAGATCCATTAATTTCCTTGTCTGCAATCACTGCACCGTCTGGGATCTCCAGCTTCTCTCCAGACTTGGCACTAATGGTCACTTTTCCCTTTAGAGCAACTCCAGCACCGAACCATACATCGCCTGCCACCTTCAAGCTATCTAGCTCAATGATGCTGGGGATAGACTTGAATCGGCTTAAGAAGTTGCTGACCTTTTTATACTCTGGCCCGAGCTCAATGGTTGGGTTTGCAGGGTTGTTCCTAGCTGGGTTTCTTATCACAAATCCATCGGCAACAGTGTACAGATCCGACTGGACAAGGAGCAAATCTGAAGTCGCCTTCACTGGAAGAAACCGTGATCGTGGCACATTCATACCAATAGCATTATCAAAAAACTTGATTGCAGCACCAGCAGCAGTTTCCAGTTGAAGAACCTTAATTCCATCAACTTCCTTGGGGTTTGGTATGATTTCCATCTTAAGTGCATCAGCTTCAACAAGCCTTTTAATTGCATTCAAGTTCACCCACAGGTTGTTGGTGTTAAAAATCTTGAACTTCTCAATCGATTTGAATTCACTGACATGCTCATCTGGAACTTGGGCAATTTCTAGAAGCTGGACCTTCCCTTCATAGGAAATAAGAGTACCACCTTTAACATCAGCCAAAGTTTTGGGTGTAACCTCCATGCAGTACTCATTCTTGTTTTGCACCAAGTGACTTAAGATCTTCAAATCAACAATAGCCCCCAAATTGTCAGAGTTGGCAACAAAGATGTATTCTTTGCCCTGTGACAAAAACAAGTCAAGTTTTCCACTGTTCTTCAAGGATGGGAACACATCACCATGACCAGGAGGATACCATCCATCTCTGCCAGTTTGACCTTTGCATGGCAATGGCGAGAAATCATCAACAACCAAACGAGGGTATTGGCTCTGATTGAATGTATGAATCTCAATGTTGGAATTGGTGTACTTCTCAACAATCTTGAGTGTATCGTCATGTGTGTTGAATGAATTCATCAAAACCAGAGGTACTTTGCATCCATACTTGCTGTTGAGGTTCTCAATTTGCATGACAATAAGATCCAAAAAGGTCAGGCCATTACGGACTTCAATGACAGACTTAGGACCAGTACAACCCATGGTTGTTCCAAGACCTCCGTTGAGCTTCAACACCACAAGTTTATCCAACAGCTCCCTAGTCTTTGCCTCATCTTCAGGTGGTGGTGCTACAGTTTCGTAAGGAACAACAATATCATCAGTAGGAGTCTTGATTTTGCTCCAATCAACATGCTGAGCTTCTCCACTAAGGTATCGACCTACGAGGCTGATGAATCCAGATTTCTCATTCTCACTGATTTGGCTAAGGCCAGAAACAGCAGAGCTAAGCTTCTGGAGCTTTTCAGCTTCAGTTGGGTTGAGAGTTGCAGCAGAAGCCATTGATAGAGGTTGTGAAGGTTCGAATAACAGAGAAGAGAGAAGAGAGAGAAAGGGAGAAAAGAGGAGAGAGAGTGGTGAATGGTGATGATGAAATTGAAGTGTGAGGGATTTAAAATGAGGGAAAATGATCCAAGAAATACGGTTTTTGTCCTAAAC

*S.maritima*36827

GCCATTTTTTCAATCTTCATTCAAATACTCAAATCAAAATAATCAAATACTCAATTTTTCATTTTTTATTTTCATTCAAATTCGAACTTATTTTTCATTCATCCCTTAACTCCGGTTCAAACCGCGAAATTTCACATTTTCACCATTAATTTTGTTTTCATTCAAACATAAACACACTTTAATGGCGCCTTTCTTCATTTTCATTCTACTACTACAAATTCTCTCACTTCTCCTTCCTCCATTTTCACCCACTTCTTCTTCTTCTCTTCCTCTTTCTCTTTCGCTTATCACTTTCTCTCTCTCTAACTAGTACTCTCTTCGTTCTTCAACAATGGCGAATCAAGAGAACAACTGTATGAGAGTCACTCGACTTGCGTCCAAGCGAAAAGCTGCTGAAGCTATGCCTGAGAATCATCATCAACAATACCAACCTCCTAAAAAGAGGGTTGTTTTGGGTGAAATTTCGTCTAATGTTTTAGGGAATTCTGCCCTCAAATCTTGCTCTGAACCTCGAACTAGAAAGCGTTCCAGGAAAGCTGATAAACCTTCTGCTAATCAAAAAACTCCTGAAGAAGATGTTGTGGGTGTTAACAACACTGTTACTGTTGATGATATTGATGCTGCTTCTGATGATCCTCAAATGTGTGCTCCTTATGTTACTGGGATTTATGATTATCTTCGTAATATGGAGAAAGAAGAGAAGAGAAGGCCAATGGCGGATTACATGCAGAAGATTCAAAAAGATGTGACTGCAAATATGAGAGGAATTTTGGTTGATTGGTTAGTTGAACTTGCTGAAGAATTCAGGGTTGTTTCAGACACACTCTACTTAACAATCTCTCACATCGATCGATATTTGTCTTTCAAACCAATTAGTCGTCAAAAGCTTCAACTTCTCGGAGTTTCTGCTATGTTAATTGCATCGAAATATGAAGAAATAGATCCAAAACCTGTGGAAAAATTCTGTGATATGACTGAAAATACCTATACTAAATATGAGGTTGTTGAAATGGAGGCTAATCTGCTTAAAACCCTCAATTATGAGATGGGAAATCCGACCGTGAAAACGTTCCTTAGGCGGTTCACGCGTGCTGCTCAAGGAAATGCCAAAAATCCAAATCTTCAGTTGGAGTTCTTGGGGTATTACTTAGCCGAGCTCAGTTTGCTAGATTTCGGCATTGTGAAGTTCTTACCTTCTATGGTAGCAGCATCAGTTGTTTTTCTTGCTAAGTTAACAGCCCATCCCAAGAAGCACCCATGGAGCTCAAAGTTGCAACTTTACACTGGATATAACGCATCGGATCTAAAAGAATGTGTTCTTCGGATCCAAGACTTGCAACTAGATAGAAGAGGAGCTGCATTATCAGCAGTAAAAAACAAATACAAGCAGCATAAGTACAAATGCGTTTCAACAATGAGATCTCCATCAGAAATACCGGCTTCTTACTTTGAGGATGTTTGTGAGAGATAGATCTTTGTGAAACTCGGTTTTGCTTCTTTTTTGCCATAGATAGAGTTTGGATGGTTATCTGCAGAATGATGCTACCTTGAATTAGCTATATGGGTATATTTTGTGCAAATACAGTAATTCAGCTGTTAATCTTGAATTGTCAATCATCTTTGACGCGATTTGGAAGACATTTTGGTGCTGTGTGCTTGTATAGTTGGCAGAGCTGATGAAGTAGGAAGATTATTTCTGTGCAGTAGCCAGTATATAGGCATGTAACTTAGGCAGAATTAGAATGTTTTTGTAGATGATTAGTCTAGAATGCAATTGCAATTGAATGAAATATTCACTATGGATGCTTCTGATTAATAATAAGACTTCAATTTTTT

*S.maritima*20428

AGCAGCAGCAGCAGCAGCAACAATCCTCTGTTTCATGGCTTCATAATCCTTCAGAAAACAGTAGAAGTCCAAGCCCTAGTCCTGCATTTTCTGGGTTTTCTCGTTTTGGAGAAGTAGGAGGAAGAGGAGTGACGTTTACAGCAGGACAAAGGCAAGAATTAGAGAGACAAACAATGATATTCAAGTATATGATGTCTTCCATTCCTGTTCCTCCTGATCTTCTTATTTCTTTTGCTAAATACCCTTCTACAATTGGAACACCACCCTCTGTGGGGAGAAAAACAGGGGTAGAAATGAGATATGGAGTGAACAGAGGAGGAGGAGATCCAGAGCCATGGAGGTGTAGAAGAACAGATGGTAAAAAATGGAGATGTTCAAGAGATGTTGCACCTGATCAAAAATATTGTGAAAGACACTCTCACAAAGGTCGTCCTTCTCGTTCAAGAAAGCCTGTGGAAACACCTTCATCAACAACCACCTCACTAACATCTACATCACCTTCATTATCATCGTCTCCGTTATCGTTATCGTCATCGTCATCCTCTCTTCATCCTGTTACTACGCTTATTACCCCTTCTGTTTCTACTTGTAACCCTGTTATATTCACCCCTTTTTCAATCCCTAAACCTTCTTCCTCTTCCTTTGACTTGCCTTCTCCTCCTCCCCAATTCAACCCCCTCCCCAGGTACCCTGATTGGTTCATGAAGAAGGAAGTTGCACCTAATGCTTCAGCTTCATCTTGTTTGTTCCCACAACATATAGGAGGTGGTGGTGAGTCAAGAGCTACTGTAGATGATCACCAAAGTTGCCTCCTTTCTAGGCCTTTTATTGATGCTTGGTCTTTAGGAGATCGCATCGATCCCAAGGATGCTACTAATAATAATAACAATGGCATTCACAATGAAAACAATAGCATCACCGCAAACAAATGTCCTGCCTCAACTTCCCCCCACAATTCCATTTTGCCTCTCACATTGTCTATGTCAGCTGGGATTGACTCTGATTATCATCATAGCTCAGACGACCACTCCAAAGACAATCCTGCTGAGATTAGGTCATCGACATCATCGCATCACCATCAGCCGTTGAATTGGATGAGTTCATGGATAAGCTCTCCACCGGGTGGTCCGTTGGCAGAGGCACTGTGTCTTGGGATATCTGGACAGGGAAACGGGATAATGGCGAGTCATTCGGATGAGTTAGTGTCACCTCATGGACATAGCACAGGCTCTAGTACAACAACTTTGGGATAACATCATCAGTGTGCTCTCAATATGTTCACCTTTTTTTTCATATGGTACCTTAAGATTATGATATTATGTAGTACTCTATCATGTTGTTAGCATTGTGGTGGCATATCCCACATATCGATGATTCTGCAGTAGCTAGATATCTGTTAATTCTTTGTAGGTTGGAGCAACAACAAAACACAACACTGGTTTGAAGAGTTTTG

*S.maritima*27150

AGCTTAAAGCTCTCTCTCTTCCATTGAAACGAACTCTTAAGTTCGGTCTCTATTGAAGGATACTCTGAAATCTCCATAGAAGCAGCCTTTTTGTGCTTTCTATCTCTTCCATTGTAGCAGCTTTTGTGATCTTTCTCTCTCATTGAAGCGGCTTCTAAGTTCTATAGTTATATATTGATGGAGGACAAACCGAATGGAGTGGAGGATGCCGATGAGAAAGTGGATGAGGATGATGGGATTATTGAGGAATCTTTTTCAGAGGGTTCTAGTGAGGAGGATGGAGAGCTGGAATTGGAAGGAACTGGATCATTACTTGAGGCCCCACTAACTGATAACGAGATATTAGAATTGGTTAATGAACTATTTGAAGCAGAGAGTAAGGCAGCAGAAGCACAAGAAGCGCTTGAAGACGAGTCTTTGCAAAAGGTTGAGCAGGAAGTGAGAGAAGAGCTTGCTGCAAATCTACAAGGTGATGAGCTAAAGAAGGCTGTGGAAGAGGAAATGGTGGCACTTAGAGAAGAGTGGGAAGAAACGCTTGACGACCTCGAGAAAGAGAGTGCTCACATATTGGAACAACTTGATGGTGCCAACATTGACCTACCAAGCATCTATAAATGGATCGAGAGAGAGGCTCCAAATGGCTGTTATACTGAAGCCTGGAAAAAGAGGACACACTGGGCAGGAACTGAGGCTGTTAGTGATGCTGTAGACTCCAAAAATAATGCTGAAATGCATCTTCAGACCCTGAGGCCTGTAAAGAAGCACCGTGGTAAAACGTTGGAGGAAGGTGCTAGTGGATATCTGGCAAAAAAAATTTATGCTACTGACAGAGGTGAGGGTTCAACTGAAAATCCTGTGGTAGAGTGGAACTCTTTTACCAAATTATTATCAGATCAGCCTTCCAAAGAAGATGCTGCCTTTGGTAGCAAGCAATGGGCTTCAGTATACTTGGCGAGTACACCTCAGCAGGCTGCGGCCATGGGGCTTAAATTTCCCGGGGTGGATGAGGTTGAGGAGATTGATGACATTGATGATAATCCAAGCGACCCATTTGTTGCTGGTGCTATTGCAAATGAAAAGGAGTTGGATCTTTCTGAAGAGCAAAGGAAAAATTATAAAAAGGTTAAAGAGGAAGATGATTTGAGTTATGATCAAAAGCTTCAACTTCACTTGAAGCGAAGGAGGCATAGAAAAAGGAGCAGACAGAAGGCTATGGGGCACTCTGGGCACTCTGAAAGTGTATTGAGTGATGATGAAGAAGCAACCTACCAAAATACAAAAGTTGCTAAGCTTGCTAGTTGTGGACTGCCTGTTGATTCTCAAGAGGAAGAAAATAAATCTAATAATTATTCTTGTGGTTCCAAGCGCTTATGTGATGATGAAGTATTGGACCATGAAGCTAAGAGAAGTCGGACTAATATTATGGAAAGTGATAATGAAGCCCACAATTCTAACCATATAAATAATGACAACATCGTAGAAGAACCATTTGGTCAAGAAAATGGAGATGGGTCAATGAAGGTTGATGCATCTTCAGAAAATGTGTATGAGGTGTTCTCCTGCACTGTTTGCAGTAAAGTGGCAATGGAGATACATGAACATCCTCTTCTTAAAGTAATTGTTTGTGGGCATTGCAAGTGTGTTATTGAGGCTAAAATGAGGGATCTCCAGGGCTTGGAAACTTATTGTGGATGGTGCGGAAGCAGTAGTGATTTGTTAGGTTGTAAATCGTGTAAACTTTTATTTTGTACTTCATGTATAAAGAAGAATATTGGGGTGGAATGCCTGCCAGAAGATATGTCATTGGGATGGCAATGTTGTTCTTGTTCTCCCGATCTGCTGCAGCAGTTGACCTTGGAACTCGAGAAAGCAGTTGGCTCTCAAGATTCAGCAGTTTCAAGTTCTGACAGTGATTCGGATGATGAGATAGGTGCTTCTATCAGCAAGTCAGGTAAAAAGAAAATTCGAAGGATTCTGGATGATACTGAACTTGGAGAGGAGACAAAGAGGAAAATCGCTATGGAAAAGGAACGTCAGGAACGGCTCAAGTCCCTCAAAGCCCAGTTCTCAGCAATACCCATGATGATGAATGCTTCCACTTATAATGGAAGTTTACCTGAAGGTGCTACTTCTGAAGTTCTCGGCGATATCTCAACTGGTTTTATCGTTAATGTTGTGAGGGACAAAGGTGAGGATGCTGTCAGGATTCCTCCAAGTATCTCTGCCAAACTGAAAACACACCAGGTAGCTGGTATTCGCTTTATGTGGGAGAACATCATTCAGTCTATCAGAAATGTTAAGGCTGGTGACAAAGGCCTTGGTTGTATTTTAGCTCATACGATGGGTCTTGGCAAAACTTTCCAGGTTATAGCCTTCTTGTACACTGCAATGAGAAGTGTTAATTTAGGTCTAAAAACTGCGCTAGTTGTAACACCAGTTAATGTGCTGCACAATTGGCGGCAGGAGTTTAGAAAGTGGCAGCCCAAAGAATTCAAGCGTCTTAATGTTTACATGCTAGAAGATAAGTCAAGGGAGCAACAGAGAGCAGAGTTGCTAAAGAAGTGGAGAAGAAAGGGTGGAGTGTTCTTGATTGGTTATACCTCATTTAGAAATTTATCCCTTGGAAAGAACATGAAAGATCGTAATTCAGCCAAAGAAATATGCTCTGCCCTTCAGGAAGGACCTGACATACTTGTTTGTGATGAGGCCCATATTATTAAGAACACCAAGGCAGAAATTACTCAAGCTTTAAAGCTTGTTAAATGCCAGAGAAGGATTGCATTGACTGGATCCCCTCTTCAGAATAATCTTATGGAATATTACTGTATGGTTGATTTTGTAAGAGAAGGATTTCTGGGAAGTATGCATGAATTTCGCAATCGGTTTCAGAACCCTATAGAGTATGGCCAACATGCAAATTCGACTGCTAAAGATGTAAGAATTATGAATCAGAGGTCACACATTTTATATGAACAACTAAAAGGGTTTGTCCAGAGAATGGACATGAGTGTGGTGAAAAAGGACTTGCCTCCTAAGACTGTGTTTGTAGTAGCCGTGAAGCCTTCTTCACTGCAGAGGAAATTATACAAACGGTTTCTTGATGTTCATGGATTTACTGGTCACAAGGCTTATAATGAAGGGATCAGGAAAAATTTCTTTGCTGCATACCAAACCTTGGCTCAGATTTGCAATCATCCTTGGATTCTGCAACTGATGAAAGATAGAGTATATGTAAAGCAAGAAGAGAGTCCAGATGATAGCGCCAGTGATGAAGTTATTGATTCCAATTTGGGGACCGGAGAAAAAGCAAAGGCAAAAATTGATAAAGGCTTCCTTCATGAGGGTTGGTGGAACAATCTACTCAATCAGTATTCTTACCAAGAGATTGAGTATAGTGGCAAAATGGTTTTGTTGCTGGATATATTGACCATGTGCACTGAAGTTGGTGATAAAGCACTAGTTTTTAGCCAGAGCATAGCAGCTCTGGATCTGATTGAATCCTATCTATCTAAATTATATCGGCTGGGTAAGAAGGGGAAAAAATGGAAACAAGGAAAGGATTGGTATAGAATTGATGGGAAGACAGAAGGCTCTGAAAGACAGAGGCTTGTTGATTTATTTAACAATCCCCAGAATGAAAGAGTGAAATGCACGCTTATATCAACAAGAGCCGGAGCTTTGGGGATTAACCTTTATGCTGCCAATCGTGTAATTATTATTGATGGATCCTGGAATCCTACACACGATCTTCAGGCTATATATCGAGCTTGGAGATATGGCCAGAAAAAGCCTGTGTATGCTTATCGATTGGTGGCGCATGGGACTTTGGAGGAAAAGATTTACAAACGTCAGGTGAAAAAGGAAGGCCTTGCTGCGAGGGTGGTCGATAGGCAACAAGTGCTCAGGAGTATGTCTAAAGAAGAGATCTCCAATCTCTTTAACTTTAGTGATGATGAGAACCTTGACGTGTTGCCTGAAACGACAGAGAAAAAAGGGCGTATGAACGATACAGATCATTGTAGAAAGTTGAAAAATTCGTTGAATTCTTCTGATTGTCCCAGAAACTGCTCTTCTGACAAATTGATGGAAAAGTTGCTTAATAAGCATCGGCTAAGATGGATTATGAATTTCCATGAGCATGAGACACTGCTACAAGAGAATGAAGAGGAGAAACTTACAAAAGAAGAACAAGATATGGCGTGGGAAGTATTCAGAAGAAGTGTGAATATGGAAGAAGGGCAAAGGGCTCCTGTTGACATTGAATGGCAGGAAGTGAGTAGGGTCGTGGTTGATGAGTCAACATTTGAGCAAAAACAAAGTAGTCAAATCCCATCACCTGTTTTACAAGAGAGAAGCACGTTGAATCCACTGGCTAATGTTCCCTCAAGGTACAGTATGCCAGTACGGAAATGCACCAACATATCCCATATGTATACTCTGAAGTTTCAAGGAACGAAAGCGGGTTGCAGTAGTGTTTGTGGAGAGTGTGGCCAAGACATAAGCTGGGGGGATCTTAACAAGTGATAAAGGTTAGTTTTGATTTTTTTCGATGGATCTGGCTAGGGTGATTGCCCATTTTGTTTATGGCTTGATCCTAGTAAGCTAATTGTTGCCTTATGTATAGGAACATGTCACTACACCCAATTACCCAAGTAGATTGTAAAAGTCACATTGATTCCCCCCATCTTCTTTTGACATCGGTAGGTTTAACTCCTGATTTTGCAATATACTAGGATCAAACAGAGTTACAACGTTCAATGTATAGCAAACAATGTCAGCCATTTTTTGGGATCACAGTATGATATAAAATCAGCATTTTG

*S.maritima*27747

TTTAAAGGTGATAAGAGATGCAAGAAGTGCTACTATAAGAAGGGATGATATAAACCTAGTTATATTACTTTCAAGTGCAACATCATCATCATCATCATCAAGTGCTGGAAGGAAAGCCAAAGTGTATTGGATCTGGGTTAATGAAGCAAGAAAGATCCGACCCATTTGGAGATGATGAAAATAATAGGGGTTCAAAAATGGCGAAAAGAGTTGAGGATTTGGGTTTGAGATCTGAGAATGCACTTCCAAAGCAGCAAATGCTAAGCTTTTCAACTTCTCCTAAACCCCAACAACTCAACTTTGTATGTGGTAAAGATGTTGAATTAATTGCTGATAAAAATGCCCAAAATCTTGTCTTATCTTATTTTCAGCAACCACCTCAATCTTCTTCTGCTTATTCCAGAACTCCTACTGGCTTTAATTCTGGAAACATGAATGGGAGCATGCATGGGTGTTTTACAGGGATCAGAGGACCATTTACACCAGCTCAATGGATTGAGTTGGAACATCAGGCTATGATTTACAAGTACTTAACTGCTAATGTACCTGTTCCTCCTAATTTGCTCCTTCCGATTCGAAAAGCCTTAAGCTCTTCTGGGTTTCCTGGCTTCTCTATTGGATCTTATTCTCCCCATTCATATGGATGGGGTGCTTTCCATCTAGGATTTTCTGGCAGCACTGATCCTGAACCTGGGAGGTGTCGCCGGACTGATGGAAAGAAATGGCGGTGCTCTAGGGACGCAGTTCCCGACCAGAAATATTGTGAAAGACATATCAACAGAGGTCGCCATCGTTCAAGAAAGCCTGTGGAAGGCCACACTGGCCAAGCTGCCTCTGGACCCACTAACACGAAGGTGGTTCCAGCGATTTCTGCTCCTATGTCGTCGCTGGTAACATCCAATGGTGGTGCCACCAACAGTGTCACGATTGCGCATCAACAACACAAGATCAAAGGGTTGCAGCAGCAAAGTGGTGCCACCAATACTAATGCTGATTCATTTGTTAACCGCTTCCATGATGAGCAAGGTCTCTCTGTGATGCCTCAAGCCATCAATTTGAAAACCAATGACAGCCCGTTTTCAATTGCGAAACAACACATTCCTCTGGATGAATCCTCTCAACCAGAATTTGGAATTGTATCTACTGACTCTCTCCTCAACCCTACGCAGAAAAGCGCATATTTGGGTTGTAAAAGCTACAATTCTCCTATATTGGAATTCAATGGTCAGCAAACTCAAGATCAATACCCACTTCGACATTTCATGGACACTTGGCCCAAAGATAATTCTAACAGGCCCACCATCCCATGGTCTGAAGACTTGAAATCAGATTGGACTCAGCTCTCAATGTCGATTCCCATGTCATCAGCTGAGTTCTCATCAAACTCTTCACCCAATCAAGAGAAAATCGCTACTGTTTCACCATTGAGTTTGTCTCGTGAGATTGACCCATCTCTGATGGGTTTGGGTGTGGGGACGATCATGGGTGAAACAGGTATGAAGCAAAATGCCTGGATACCAATTTCATGGGGGAATACTACAATGGGAGGTCCTCTAGGGGAGGTACTTAATAATACAAGTAATGTGACACCAGCCTTAAATCTCAAGGGTGAAGGGTGTGATGGGAGCCCTCAGTTTGGGTCATCCCCTACTGGTGTTCTGCAAAAGTCTACTTTTGTTTCTGTTTCAAATAGTAGCTCTGCTGGTAGTCCGAGGGGTGAAAATAAGAAAGCCCCCGAGAGAGCTAGTCTATGCGAAGATATCCTTGGTTCAGCAGCTCATGCTAGTTCTACATTCATCCCTTCAATGTAAACTATCGGAAGAGCTCGACGAGGTAAGAGAGGAGAGTGATTCCGGAGATTATGTGACTTTCTTCATATTTATGAAAAAAAAATGTTCGAAAACAAGTCTTACTGGTAAGGCCTGTTTAAGAATTAAAATATGCTTAATCTGTTATTTTAATTGAGTTTTGCTGAACAGTTGTGGGAGGTTCAAAACTTAGAGCAGGTGTTTATAGCTCAACCTATTTTTTGGCTATGAGTTGTGAACTTGTGACGACCTGTTCGTCGAATGATCACAGTGTATTGTTTGGCTGTTGTCATTACTCTGTATCTATCAATGACCATTTTGTATCTCCTTCATTCTCCATTTGGAGGGTTAATCCAAACTTTCAAGAATTTTAATTGAGAGAGAGTTGGATTTGAAATCAAAGCAATTTCATCTTGGACTA

*S.maritima*27747

GTCAAAAAAAAAAAAAAAAAGATTCAATTTTTATGGTGTTTTGAGTAATGGGTTCAAATGGGGTTTTAGTTTCTGCAAATGGGTGTTCTTCAGATTATGCAGCAAAGAGGAAATGGTGTGGATCTAATAATGATGAAGTTGTTTGGAAACAAAACTCAAGTTTTGGTAAATTAAGTGGTGATTTCTGTGATAATGACCAAAATATGCTTAGCTTTTCTTCTCCAAAGTTGAGTGACTTTTCTGCAAAAGATAATGGTGAATTAGAGAGTAAGAACTTACTACAAAATTTTCCCACATTGCAAAATTTTCAGCACTCAAATTCATTTTACAGTAAAAATGCAGGGTATTCTTGTAATATTGTGGGTATGAATGCAACAAGTATGTACTTCATGAATGGGGTTAAAGGAGCATTTACATCATCTCAGTGGTTTGAGCTTGAGCAACAGGCTTTGATCTACAAGTACATTCTTGCGAATGAGCCTATACCACCTAATTTGCTGATTCCTATACGAAGAGCTTTTGAATTAGCTGGGTTTTCTGGCTTCCCTACTGGGCTTCTGAGACCTAATTTATTTGGATGGGGTCCTTTCCATCTTGGGTTGGCAAACAGCACTGATCCGGAGGTAGGTCGGTGTCGTAGGACAGATGGGAAGAAATGGCGGTGCTCGAGAGATGCAGTACCTGATCAAAAGTACTGTGAACGGCACATCAACAGAGGCCGCCATCGTTCAAGAAAGCCTGTGGAAGGCCATCCCGTCTCTGGAGGCACCACTAACAAAAGTAGCAACTCCAATACTAGTACCTCTAATGTCATGAATATGACCTCGTCTTCATCTGCATTAGTGGTGCCCGGAGGTGGGCCAGCCAACAGCTTTGCTATTAACCTCAGCCAAAGCAAGGACTTACAGTTACAGCCTGTAACTTCAAATCATTTGCCTTCTTCCCTGGTTCACAGGTCTATGAACTCAATGTTGAATAAAACAAACAGCGATCAGAGGATGCAAAACACAACTGGACTAGACTTTTTGTCCCCTAGAATTAGCCAGAATTCGCGAGCTGCTGAAAGCACATTTTCCATTGCTGAATCAGCTGCTGCCGATTTCCCATTTCTCACATCTGATGCCACCATCTTAAACACCACACAGGGGTGCTCTTATGCATCAAGAACTTGCGTACAATCCCAACATTCACCTCACCTCTTCACTGAGGATGATCCATCAAGCAATTTATGGCCAAAGTTAGAGACACAAACAAACACGACAACCAAGCTATCAATCTCAATACCACCAGCTGATTTCATGTCTTCAACCTCCTCTCCAACTGAAGAAAGGATGGCCCTCTCTCCTTTGCGATTATCTCGGGAACTTGACCCTGCAAAAGTGCATTCAAGTTTGGGTTTGGATACTGTATCAAATGAGCAAAGTCCCATTTCTTGGGAAGCTTCCTTAGGAGGGCCACTTGGAGAAGTCCTGAAGACCACTACGAGTAACAGCTTGGGCGACCAAAGCAAGAGTACATCAGCTCTTACCCTCTTATCTAAGGCCTCAGAAGGTAGCTCTCAATTGGCTTCATCTCCAACAGATGTGCTTCAGAGGACTGCACTCGGCTATTTCTCTAACAGCAGTGCAGGGAGTAGCCCTCGAGCAGAAAATGGAAGAGCCCTCGATTGTGGGATCCTTAGTGGGGATCTTATGGGATTCACTCTCGGGAGTTCCTCTTCTTTGAGTAGGTGCAGCAGGAGATGAAAAGCAGATACACTTATCTGTCTTTATATCCGCTATAACTCAGAAATCTGTTGTATAATGATGTTTTGTAGTCTATATTATGTATTACTTCGGCTTTGTAGAATTTCTTGACTTTGAAATTATCTCAAGGGAAAATTTAAGCCTCTTGGGGAGTTGTTTCATATTGCAATGCAGTCTGTTATTTTCACCTTGCATTGTATGACTACTTGGTAGAGAGTTTTGTGAAGCATAGAGCCAATTGGCTGTTCGGCTCAGCTGTACCTGGTAGGCTATCAGGTCTTGTAAGTGTAATTTTTCTTAATACTGTAATTGTTTTACAATGACTGAAGAATAGTCTGTATTCTTGTGTTTAACATGCA

*S.maritima*27683

ATTGTATACAATTATAAAATATATTATGACAACCAATATTTAAGTGAAACGGCAACTTTTTGAGAAACAGAATTACGTAAGTTTATTATTCTAAAAATGCTTCATTGTCACAACTAACGGATCATATAAGCGTAGGTACTTAATAATAGTAATAACATAGGGTATATGGAGCTCAATACTGTTGAAACATTCAACAATATAAGAGTAAACAAATTAGGAATTGCATTCAAGGGAATATCTTCCTCGAAAACCACATAAAACAGTTGTACATATTATTATAATTACGAGTACTTTCAATAGGTAAGTGTTGGTAACAAGTCCAATAATAATCAGTCATGTAATCATGTTGTAATCTGCAGGAGTTTCTTTACATGAAACCAGTCAATCTGAGAAGCAGAGAAGCAACTGTCCATGCACAGTACAGCAAGAAAGCGTACACAAATACTTGTAGACCAGTGTGTGCAATGTATAATTTTTCCCATTTTTTCCTCGCCGCAGGCAATGTATGGATATCATCTTTTACAAGGTAAACTCTTAAGCCGAGACAATGTGCCTGACTAAAGTATTCCCAGGATAACTGATTTATGTCAAAATTGAATATATCTCGATCTTGACTATCGAGAGTATTCCACAACGCCAGCACTCGATCATTAGTGAACGTCCATTCTTTGTCTGAGAAATAGGCTAATATATCACGTACTTTGTCAATCTTCTTGTAAATTTTCATCAATATTGGTTTTTGTCCAGCGATAACAGCTAATGTGTCCAGTAAATATCCGGGTACGATATGGCAAACAAAGTTTAATAATGCATACAAATATGGATTGTTGGTGGGCCAGAACGAGTAGTACCAGATTGCTCGGATTGTGGGCCAGTAAATTCCATGACGTCTATTCATCTCGATGAAATCATTCCACTTAATTGGTTTTTGCCCTGCACTTGAGCACGTGAATATCGGTATTCCGTCGTCTTTTTTATTAGTTGCAGTTTCTTTTGTTGAACATATAAGTGCGTTAACAACCATGTCTACTGGAATCATATCAGTAACCATATTTGAATCACCAAAGTATGTATGAAGAACTCCTGTTCCAGCTCCAACTATGAGTCCTGTAGGACCATAAACATTATCTATCCACCCCCTCACTGGTTCTCGGTAAGTTGCAATCACAACAGTGGGACGGAAAACTAATATTGGAAGATCTTGCGCTTCTCTTCGGATGACGTCTTCTGCCAAAGATTTGGTAAATGCATAAGTATTTGGTAAATCGCCAATGATTTCTTTAGTCATGTCTTGAAGCGTTTGATCGTCTTTAGAAGAAATCAATTTTAATACATCTTCATAGTCAGCAATAGGGTCGTAAAATTTTTCCTCGACGTGCAATCGATTACAGTTTGAGTATGCTGTGGAAACGTGTGCCATGACCTTTAAGTTGGTTATTTTCCTGGCCAAACTTAGTAATTCTCTTGTTCCAGAAACATTTATGGTCATAGCTACACGGATATGTTCATCAAAACGCACAGTAGCTGCTCCATGGAAAATTATGTTCACTTCATTCATCAATGTATTTCGACTACTTACGCTTAATCCTAGACCCGGCAAACTGCAGTCACCGGCTACTCCAGAAACCTTATGGTAATACTTGGGTACCTCATGTTTTAATCTTTTAAAAAGCCTGTCTTCAAATATATCTTCCAATCGTTCATCGACGTTTTTGCCTTTCTTAGACCGGATCAGCAAATAAATATGTTTGATGTGAGGACATGTTCTGAGCAGTTTTTCCACTAAAACCTTGCCCATAAAACCTGTACCACCGGTTAAAAATATTACTCCATCCCGGAAAAACTCTTGTACTGGACTTCCGATCTCAGACTCATCAACTTCACGAGGTTCACTAAAGCTCCTCTCGCCCAAAAGCTCCAGGGGATCCAAGTAGTATTTAGAATTTATTATATCTTTGATAGGCACCAACGGTGCACTATCTTGCTTTTTCTCGATGTACTTCCGATGTAAATCGAGAGCCATATTGTGGAATTGTAGATTTAATAACTCTTTTAAAACGACGGTCGACGATGAACACAGGCACTCTGCAGATAGAAACTACTTGAACGAAGTACCGCGACGG

*S.maritima*29250

ATGCGATTCATGCCTGAGTAAAAATTCAGTTCCCAATTTACTATTGAATTGTCAAATGGGAAGTCCTACATTCTCTTCTCTACAATACAACTACACAAGATCCATCACTGTACTACCCTACTACAGTACCTCTCTTTTTTCCCCTCGACATCAAAGAATTGTAGAACAAACTGCATATCTTTCAGCAGAGACCTGTCAAATTTCCTCGGCAGTGGAAAACTTCTTTGATCTGTCTGTCACTCTGTACACGGACATTTGAGGTGAAATGAAAAGTCGACTAGTAGACTGCTGAGTCCTGACTTTGCATGCAAATGTGAAAATTGCCTATAATGATATAGGAAACTGCTCGTAAGCATTAGATTTCTCAGACCAAAAACGTCCTTCTATTCAAGATCTGTAATGTTTGATCCACGGATAAACTTGAAATACCGAAAGTCAACAAGTAATCTGAACATTTTGGATTGTCTAGCAAGCTGGATTTCAAAACATCGACCTCATTTCTATCTAGATTCAATACCTTAAGCAAATCTCTAATGCATAGAAAAAAGGCTTCTGGGCGAACACATAAGGCTTTAAACGCGTAGAACATCCCCTGCATATCAGCCTTTACCTGTTCAGCTCCCATAACCGAAAAGCGAACACCAAGAGAGAGAAGACTGCGAACTATAAAATGATCAAGCCCATCTGCAATACTTCTCCAAGTATCCAAGAAATCACTCGCATTAAGATGTTCTTTCATTAGAAGAAGCAGGCTTCTCAAATTATCCAGTGCTTCCACAAGATCATCAGATATAGTCACGTCCTTCACACTAACTTGCTCAAATTGCTCCATGTTATGAAAATAATAGAGTGAATGGTTTTCAAACTGCTGAAGAGCATGCGTGATTAATTCCATAACCCAAGTTGTTTCCATTTCAACTAGGCTCTCAATCTCTTCTTCAAAGAAGCAACTGCCATTAACATCAAAGTTTAGTCCTTTTTCAGCAAGTTTCAGCTCCAAAAGATCAACATCATCACTCCATTCTTTCAATTTGCATTCAGAAAACCTGGCTGCATTGATCAATTGACAGACTTTAACTACATTTTCTTCAGCATCCTGAGCAATATTGCCAGCGCTCTTATGCCTTGAAAGTAAAATTTTAAAGAAGTGCCAGAGGAACTTGCAAGGCGCTGATTTAATGAATTGTATTCGACGCGCACTACCAGGTAAAGTCTGGCAGCGCTGGATCATTTCCCAAGCTTTCTTAAGAGCAAACTCTGCAACTAAAGGAGCTTTGTGATCTTCTCTAGTAGAAAGAAAGTACTGGTTATCATCTTTCTCTAGTTTATGGTCAGACGACCAAGCTCGTTCATCCTTCAGTTCTGCCTTTAGTTTTTTCCATGCATCCTTAAGTTCTACCTTAGCCCAAATACGGAGCCAATCAGGTCTATCGCAGAATAAAGACAATGCTGACATACTCCTTGCTGAACCAGGGAGAAGATATGTGTCTGAACTGATAAATGACTGCATCTGTTTATCGAATCTAACAATTTCATCAATCAAGTGAAGCCAGGAGGATATCGCATTTGATTTAGCATCTTTTTCTTTGTATCTTTCAGCAAGAACTGGAAACACTTTTCTTCCCAAGAATCCAGAGAGAAACTGCACCATTGCAGACACCCACGCCTCCTTAGCACTACAACTTACTAGCCTTGCCTCATCTATTAGAGGCTGCAAAACATCATCCACTCCATCACTAAAATCACGTATAATCTTATGAACAAGAGCAAACATGAACTCAGGCTGATCAACCCATTTGAGCAAATGAAGCTCAATCTTCGATGCTAGCGAAGACACTAATTCGTCAATAGCCCAAAGCCCAAGAGTTTCTTCCATCTTTTTTCCTAACAAAAAATGGTGCCGTCCTTCCCTCTTCAATTGTACGTGCTGCAATGCACAAAGTGCAAGAAAGCTCTGGGAATAACTCTGTTTTTTTCCTCCTTGCATAAGAATTAGGGGATTTGGGATTCCTATAGCCTCCCCATCTTGTGTTCTTGATGTCAAAGTTTTGGGAGGCCATCCAAGAGAAGCCAGAAGACTGCGGTGATCAGCAATTACCTGAGGCCTAAGCACACCCAAAGTTCTATCTACTCTGGCATCGACAGCCTTCAAGAGGTTGTGCCACTTAGGATGATCTTTCAGTACACACAACAGAATTTCTTCAATGTTGCTGATCGCCTTCACAGCCCTATGGACATTGTCTAACTTTGCACAAAAATCTCCTGAGATCGATGGACTCGGATCAGATCTTGAGAAAATATATCCAGTGCATTTATTCAGAGCTGATAATACAGCATCCTCAAGATCTCCAACCAGTGTTTCCAACTTCAGCGCAACCCCTGCATACTCACGTATGCTTTCAATTCGCCGTAATTCCTTCGCAATTTTTGGCAAATCATTCCCAATAATCTTCTCAATTTTCCTACTTTGTGCCCTGTTTCCACAATTAGAAGAAATGATGCTAAGATTCTCCAAATTAAGTATAAAATCATTGAGAGAAGATTTGGCGCGAATTGAACGAGAAGTTGAAGAAACTAACAAACTTCCAAGGGTTGTGTGGAGATTTTGGAGATGGGTTTGAAGATGGGTGCATTTGGAATTGAGTTGAATTGACAAATCTTGAAACTTTGAAGGGTTTAAAAGGGGATTAAATTGTTGATTGTTGATAAACCCTAATGTTTTTTCTGGAATTTCATTGAGTTTGGGAAGAACATCTTCATCATTCAAACTTTCCATTTTTTTATTTTTTTTTTGGGGGAGATTTTTTCACACTTGACTTGCTTCCCTGCGA

*S.maritima*38649

TTTCGATTACAACTTTGATTCAAGTTAGTTTACACACTTGTTTTAGTACAACTTAATTTGCGATGGAAATAACATACCATTACATTGCTAGATTCTTTAAAATTGTTCCATCCAAAAATAAAATTTATGGCAAAATATTACGTAGTACTTTAATTCTTGGCAATAAGTAATCAAAAAATCTCCAGTATATTCACAATTCACAAACCTTTCTCCTTGATTAGTTACTAGGAATTTGCTGCTACTGTAAAAGGATGAGCAGCTCGAACTTGATTCTCATGGAGCAAGCATACTGAAAGTCCTTCAATATTTCCAGAAAATATCAGACCATTGACGGACTCGTGTTTTATCCCAATTCGCTCTAAACGGAACCATTCCTATAATTCTAAACTCTGATTTCGCACACCCAGATTTTGGTGGGTGATATCGGTCAAAACTATTTTCTATGTACACTGGGTTTTACAATTCTATTACCATTAAGGTATCACATTCTCTCTACATGATCGCTGGTTTTTGCCGTGTCTTGCTGAATAGTACATCTCCACTCAGGACTGAGCCGATGAACTTCTCTACTTCTTCAGCGTTTAAACTCCCTGTATATGCTGCATACCTCCCTTTCCTTGGCTTGTAGGCTACCAAAAAGCTATTTGATGACTTGAATCCAGATTTTTCGAAAGCATCCAGGAAAGATTGTTGCTTTGCCGCATCTACCAGAGTGTATGAGATGGAATCCTTGGAATCAGAAAGACTTCTTCTCCTTGATAACGATTTCTGAGAAACCATTGACAAAATTGATTCAAGTTTGTCTTTTGCTTTTGATGATCTAAAACCACCAATTATGCAAACAGGGTTTTCATCTCCACAAAAAGCATGGTAGTTCGATGTTGTCAAAAGCGGTACTTTTTTGCTAGATGATTCACTCTCAGACTTCTTGGTCTGACTTGAAGCAATTTTGTTCTTCTTTTCAAAACTGCCAAGCAATGCACCAAGTTCCTGAACAGCTGACTCTAAATCTCTCACAGATATCCCAGCTTTTAGAATTTGTTTTTCACCATTTGACAACCATCCCACTATAGCTGGAAGTGCGTTAACTCCTAATCTCCTGACCTCTGGATCAGAAACATCATGAACCTGTGTGTCATAGAAGACAAATCGTTTGCGATACAAACCACTGAGAGTACGCCAGATCACTGGGGTATCTTTCTTTGTTGAAAGAAGCATGACTCGAGGTAACTTTTCCCCAGCACCAAACAAAGGCTGAAAGTTATCCAGGCTAACACGTCTTGAAAATCTCGGCAAATGTTCCTGACAGGAACTTTTCAAGCTTTTGACATTCAAGTCATCATTATACTCCACAAACGAACCACTCTCGCTAGTTATGTAGGAATATACAAACAATCTGGGTGCTCTACGAGGATGTATTCCATGTTTCTTGCAGAAAGAAGATTCATTCTCACAGTTTAATTTACCAACTTTCACAGCTCCATTCAATGAGTTATTAATCTCCTCCAACATTGATTCATACTGCTGGACTGTCATCATTGAGGGAGTGTAAGATAGCAGAAGCCAAGTAATGCCCTTTCCATACATCTCATTCTCAAATACTTGTGAACTCACAGCTCGAACAGACTTGGTGGGCCTCTTGAAACCAGATTGTGATCCAGATCCAGCAGAACCACCAAAACCACCAAAACGAGAACCACTGCCAGCATCACCACCAAAAAAGTTTGAAAAAATATCATTGATACCAAAACCAAATGGATTTCCACCACCAGATCCACCAAAGGAAAAGGAGTACGACCGACCACCCCCACCCATATTCTGCCACTGTCCTCCTGACCCACCACTTGTGAAGTGGGAGTAGCCACCATAATCTCCTGTACTCCCACCGTCAAAGGATGGACCACCTCTCTCATCACCATATTGGTCATAGTTTTTCCTCTTTTCTTCATCAGATAGAATTTCATAAGCATTATTGATCTCCTCGAATTTTGCTTGAGCACCTTTGTTCTTGTTCTTGTCAGGATGATATTGTAAAGAAAGCTTGTGGAAAGCCTTCTTAATATCACGTTGACTAGCATTCAGATCAACACCTAGAACCTTATAAGGGTCTAACCCCTTTTTAGCTTCAGCAAAAAGGATAATTAATGATACCACAGTAATTAGAACGAGCTTCAATCGGACACCCATTATTGAATTTTCCAGGACTTTTATGTGAATTTCAGTTTCTTTTCTTTAGTCAGCGGGAGAAAGTGAGGAACAACAGAAAATAAAAATAGAAGTTAAATAAAGTTTGGAGAAGATTGTGGAAAGTATAAA

*S.maritima*37948

TAAAAAAAAAAAAAAAAAACCCTTTATTCAATTTCCATAAAAAGTTAAGTAATTTGGTTGTTTCCTTTTTGTAGTTTGTAACCAAAATAAGCAAAAGAAAAGATGTTAAGTATGAGAAAGAATAGTTCCAATAATGAGTTAGGCGTTTCAAGCAAGAAATTCCAACAAGCTAAAGATGAACAAGTCGTTGTAGTTGAGTTGGGTCTTAAACTTGAGCAAGATCACAACCGTTCATTTCAGTTTAATCATGATCATCATGGTGATGGTGATGGAAGTTGTTGTGGGCCCGCCTATAACATGATTAGTAACAACAACAACAACAATACTCCTACTATATACAGTAGTAGTGTTGCTGCTTCTACTACTCCAACAATTGCTGTTTCTTCTTCTACTCCTTCAACATCAACAACAACAGGATTACTGCAACCTTTTGATGTTTCTTCTGCTTATTATTACTCTTCTGCTAATTATCCCTTCTTCAAATCCTCAGGTGGTATGGCAACAACAACAATGGGGTTTCCATTTACAGCAACACAATGGAAAGAATTGGAAAGACAAGCAATGATTTACAAGTACATGATGGCTTCTCTTCCTGTTCCTCCTGATCTTCTTTATTCCTCTTTCCCCACCAATTTCTCCACTTCCACTTTTGCCCCTGCTTCTTATTTTTTGGGAAAGTATGGGAGTGGCATAAGCATGAGATGGAGTGGAAATAGCAAAGATGCAGAGCCAGGGAGATGCAGGAGGACTGATGGTAAGAAATGGAGGTGCTCAAGGGATGTGGCGCCTAACCAAAAATATTGCGAGCGTCACATGCATAGAGGACGTCCCCGTTCAAGAAAGCATGTGGAACTTCAACAACAACAACAAAATCATCATTCTTCTTCTATCTCAAACATTTCCTTTAACAACAATAGCAAAGACACAGACAATAGTACCCCTACGAAGAAGACTCGTTTGAATAATACTCTACTCACCTCTAACAACAATCATGGTGTTGGATCAAAAGTTCCCTTTTTTGATCATTCTTTGTGTCCTAACAACAATAACAACAACAACCCCCCTTTTGGTTATTCTAGGAACTTGGGTTGGAGTGATGAGCAACAATGGAATCAATTGATGCAGGCTAACATGAATGCCTCCGTTTTTCAGCACGAATTTGTAGGGGAACCTTTGAGTTTGAATTCACATGGTGGATTTAATGATCATAAATCGGATGATATGGTGTTGTTAGAGAGGCCTTTGATTGATGCTTGGTCAAATGGTAATAAACTTTCACTTTCTTGTTCAAATGGAGGGGATAATAATAATGTTAATGCCAATGGTATTGATGAGGAAATGTGCCAAATTCATATGGGCCTTGGGCTTATTGACTCATGGGTTGGGTCCACAACCACCACACCTGGTGGCCCATTAGCTGAAGTTCTAAGGCCTGGTAATCCAGCAATGTCGGATGGTGGTGAAGGCGAGCTCATCACACCACCGGCAACTGCTGTCTCATCTCCTTCTGGGGTCCTTCAGAAAGCCTTTGCTACATTTTCTTCTGATAGCAGTGGGAGTACTAGCCCTAACACTTGCCATAACTCCAAGGCATATTCTGATATGCCGTTCCACTGGTTGAATTAGTATGTTTGTTTTTTCGGCTTGAAGCTTCTTTTGAAGTTGTGTAAAGCTTCAAAGAGAAATGGTAAATGAGGATTGAGGAATCTATCCTGGATTAGTTGGATGTGTTTTTTGCAACTAATCAGTTGGAAATTTTGTGCTTTTGTATGTTTTTTCTTGGTTTTTTTTTATGTATGTGTATGAGATCCAACTGTGTAGTACTTTTGCTATTTTGCTGTCGGAAGAGTATAAAATATGGATGCAACTATTGTTGTTTTCGCTGTAAATTGTTCCTGCGTCTTTAGAATACACATGATTTTTCAGACTTCAGTCATGTTGAGCGCAGCATT

*S.maritima*35644

AAGAACAGATGGGAAGAAATGGAGGTGCTCAAAAGAAGCATACCCTGACTCTAAGTATTGCGAGCGCCACATGCACCGTGGCCGCAACCGTTCAAGAAAGCATGTGGAATCCCAGTCTCTCTCTGCGTCCTCTTCGTCATTGTCTTCTTCAGTGTCTGCTGCTGCCCTGACTCTGAACCAGACCCAGAGTGCTCCTTTGAAGTTTAATGCTTTTGCTGATGCTAGTAACTCTGCATCAAATTCTATTTCTAATCATTCGCAATTTCAATTGGACTCTATTCCTTATGCTATTCCTACCAAACACTACTCCAGGCACCTCCAGGGAGTTAAACCTGAGGCTGGAGAACACAGTTCCTTCTCTCAAAGCTCAGGTGGCAACAGATCTCTGCTGATGGACTCGAATTTGGACAATGCATGGCCCTCTCTGTCACCCCAGCTGTCTTCATTCTCTCTTTCAAAACCAGTGGACAATTCCTCAATGCATAATGATTACCGCCATTCTTTTATCAACAGTGAGTTTGGCCAGCCACAGCCAGTGAAACAAGGCAGTCAATCTCTTCACCCTTTCTTTGAGGAGTGGCCAAAAAACAGAGAGGCATGGTCTGGTTTGGAAAATGAGAGTGCCAACCAGGTCTCTTTCTCCACGACACAACTCTCCATTTCTTTACCAATGGCTACATCGGACTTCTCTAACAGTCCACGATCCCCTTAAGAACCTTGAAACAAGCACTGGTTCTTTCAAGCCAATACAAATCCAGCATCTTAGGCTCCTTTTCTGTAATTTATGTCAAACAACTCTTGCTAACTTGGTTTTCCTGATTTGCTTGCTAATATGTATTAGATTATAAAATGTTGTAAAAGCCTTTACACATGAGGTGCTATAGAGAGGGTGACAGGCATGCAGATAAGGAGAAATTCAACTAGTGAGGCTTGAAGCTAAGAATCAAATAACTTCCTTCTGTAACCGTCACTGTTTGGAAGCAGCGTCTGAGGGTACATATGAAGGCAGAAATGCGTAGTATTCAACTCTGACTTGATCGGCTGCTAGTTTATGATTCTCGTTTGAAGCAGAGTCTGCAAATGTTGTTTCTGATCATAAAGAGTATTTTCCTGAGACTATTTCATTTGTTTGTAGCAACGGTTCATGTTTTCGTTGTTTATTTGGACTAAAAAATTGTATCAATGGTTAAAGTTGAAAACCATGATCAATCAAGCATCTTATATTAATATTAAGAGGGGTATCTATTTGATGCCCTTTTATTGTTTCTGTTTTAATTTTTCATTTGGCGTTATAACTCTCATCTTGTAAGCTGTTTGAGGGTTGATTAGAGGAACTTTGAATCATTG

*S.maritima*35644

ACAACAACAACAACATCAACAACAACAGCAAGAAATTGCAGCTCTTCCTTTGTTTGTGTCATCTGATTCTCAACCCAATAAAATCTCCACCACTCATTTGTCTTCTTCTCCTTCTGCTCCTACTGCTATTCCCACCAGAGGAAGATATTCATTCAGCTTGGTACAATGGCAAGAGTTAGAACTACAAGCATTGATATACAGACACATAGTAGCAGGTGCATCTGTCCCTCCTGAGCTTCTTCAGCTTGTGAAGAAGAGCCTTCTTGCTTCTTCTTCTTCTCCTTATTATCTTCATCATTACTGCCCTCATTTTCAGCCTGCTGCTGCTTTGTTGCAAGCAGGGTATTGGGGGAAAGGAGCAATGGATCCAGAACCAGGGAGATGTAGGCGAACGGACGGGAAGAAATGGAGGTGTTCGAGGGATGTGGTGGCTGGTCACAAGTATTGCGAGCGCCACATGCACCGTGGCCGCAATCGTTCAAGAAAGCCTGTGGAATTTCCTGCTGCTGCTACTGCTACTGCTTCCGCTAATAATGCTGCGCTTGCTGGGTCACTCAGCAATAACAACAATGCTTCTCCTGCTTTTGCTGCTAGATCTTCTTCTGTTGCCGCTTCTGCTAATGAAACCCCGTTTTCGCTGTCTAGATCAGGAGTTTCTTCTTTTGATGCTTTCCAACTTAACAGGTCATCTGACAAGAAAACCATGTTTGGACCTCAATCATCAGTTTCTGGGGATGGTGGGAGATCTAACAACAACAATAACAGTAACAACAGGGTATTAATGCACTTATTTGATGACTGGCCAAGATCTGATGATGCCGAGGGAAACAATTTGTCATCGACAAGCCTCTCGATATCGACCCCAGGAAACCCGACATCAGATTTCTTGAGGCTTTCAACCGGGAATGGGGACGAACTAGGTCAGAGGGACAGGAATGTAAATGGAAGCGAAGACAGGGATAGAGGTCAGCAAATGAGTTGGGGTAATGGTGGCGGGTGGGGAGGGAATTCAATGGGAGGAGGACCGTTGGCTGAAGCATTGAGATCGTCAAGCAACTCATCTCCTACTAGTGTTCTACATCAGTTACCTCGTTGCTCGGCTCCTGACACTAGCTTTGTCAGTGCCTAAAAATGAATTCGACTTCACATCTTTTTTTTTTTGGTTTCGGAGAATAAAAATAAAGAGTTCCTTTGAAATTATGAGGGGATAAATGTTTAGACTTATTGTTTCCCTTTGTTTGCTTCTAACTGTTAAACACTAATCTTACCCCACATACTGCAGGGGTGTTATGTTATTGCTGACCACTTGAATGGTGATCCTTGTAGCAAAGCCATGGCATTTGTACTTGTTTTTTGTTTGTTATTTCTGTCAAACCATAACTATTTCCATATGACAATGTTGTACATTTTAAAGACATCTTTCATTTGGACACTACAATATTACTAGCGAGTGTAGCGACCAAATTTTGAAGTTACTGCTAAGCATAC

*S.maritima*38793

GTCCGTTCATTTTCACAAACAAAAAAAACAACCAACCATTGTTTGTAATGACTAATGAATACATTTGTGGTCATTAAGCAATTTCATTTCACCATCTCCTTGATCAATGAATCAAATTAGAGGTCAAAATTCCAACTTTTGCAACGATGCAAAGGCCAAACTATCAAACAAAGACAATACAAGAACGAACTCACTGATTTCTTTTCATTCCAAAGGAAAAAAAAAAAAAAAAAAGGAAACAAGGAAGTTGAAAACCAAGGACAGAATTATGCCTAGCAAAGTGACTTAGCTGCTTCAATGACGGCTTCTTTTGTGATACCCAACTCCTTGTATATTGTTGGGGCAGGGGCACTGGCGCCAAAACGATCAATGCCAATTGCCTTGCCCTTAGTGCCAACATACTTCAACCACCCGAATGTTGAACCAGCTTCAATACTAACCCTAGCTGTCACGTCTTCAGGCAAAACACTTGCTTTGTATGACTCAGGTTGAGCATCGAAAAGCTCCCATGAAACAAATGACACAACTCTAACAGCCTTTCCATCCTTTCTAAGTTCATCAGCAGCTTTAGCAGCAATCTCTAATTCGGAACCAGTTCCCATCAAGATAACATCAGGTTTGTTCCCATTTGAATTGTCAGAAATGATGTAACCACCCTTTGCAACTCCTTCGACTGAAGTTCCGGGAAGCTGTGGAAGCTTTTGCCTAGAAAGAGCAAGAATTGAAGGTGTTTTCTTGTTTAGTACTGCTACTTTGTAGGCACCAGCAGTTTCAGTACCATCTGCAGGACGGAGCATGAAAACATTTGGCATGGCTCGGAAACTGGCCAAATGCTCAATGGGCTGATGGGTTGGCCCATCTTCCCCAAGCCCGATAGAGTCATGGGTCATAACAAAGATGACTCCAGACTGAGACAAGGCGGCCATCCTTATGGAGGCTCTCATGTAATCCGTGAAGACAAAGAAAGTTGCGCAGTAGGGGATGAAGCCTGGGCTGTGGAGAGCAATGCCATTGCAGATAGATCCCATTCCATGCTCCCGAACACCAAATCGTAGGTTCCTTTCCTCAGGGGTATCCTTCTGGAAATTGCCAAACATTTTCAACAATGTCATATTTGAGGAAGCAAGGTCAGCACTTCCACCAAGAAAACCTGGGAGAACTTGGGCAAGCCCGTTCAGACATTGTTGAGACAAGTTTCTGGTAGCATCAGCTGCACTCTCTGGGGTGTATGTCGGAAGTGACTTCTCCCAGCCAGAAGGTAATTCCCCAGTGATAATAGATTTTAACTCTGCAGCATCATCCATGTACTTCTTTTCATACTCGGCAAACTTAGCATTCCATTCAGCTTCAAGACTTGCTCCTTCAGGGGTGTGGCGGCTCCAATGTTTCTTAACATCTTCAGGAACATGGAATGGCTCAAATGGCCAACCAAGATTGCTCCTTGTTGCTTCCACTTCCTTAGCTCCCAAGGCACTCCCATGGACACTGTATGAGTTTGCCTTGTTGGGGGATCCAAAACCAATAGTTGTAGTCACCTTAATTAAGGTTGGCTTGTCCTTAACAGCCTTAGCTTCTTTGATTGCAGCGCGGATCTCATCATAGCCATTATTGCCATTCTTGACCCAAATCACATGCCATCCAAGAGCTTCAAATCGTTTGTCAACATCCTCAGTGAATGCGATATCAGTGCTACCGTCAATAGATATATGATTATCATCGTAGAATGCAATAAGCTTTCCAAGTCCCCAATGCCCTGCAAGCGAACAGGCTTCCTGAGAAACACCCTCCATCTGACAACCGTCCCCAAGTATAGCATAAGTGTAGTGATCAACAATTTCCATATCAGGCTTGTTGAAACGAGCAGCCAAGTGCTTCTCTGCAAGTGCCAGCCCAACAGCATTGGCAATACCCTGACCAAGAGGACCAGTAGTGACTTCAACTCCAGGAGTCTCGAAATTTTCAGGATGACCAGGGGTTCTGCTTTCCCACTGACGGAAACTCTTCAAATCTTCTTCAGTGACACTGTCATAGCCAGCCAAATGAAGAAGAGCATACTGAAGCATACACCCATGACCAGCAGACAAAACAAAGCGATCACGGTTAAACCAATACGGGTTTTTGGGGTTATACTTCATGATTTCATCGTACAAAATGTGACCCATCGGAGCACAACCCATGGGCAAACCAGGGTGACCC

*S.maritima*38542

CTTACACTTGTGCAACATTTTCATACACACAATCCTTTTCTTCCTTCTTAAATCTTTTCATCAAAATATAAATAAATTAAATAAATAAAAAATGGAGCCTTTTTCTACTTATGAAGGATACTCTAAAGGGTATAATCAGAAACGCAAAAAAGAGGAAAACAGGTGGAGGGTAGAAACAGAGATAAAACAGGGGGAAACAGGGGAAGATGAACCACAGCCATTGATCAATCTTGGTCTTGGGATTGGTGGGTCCACCTCCACTAGCTTCAGAGTTTATGACAGAGAAAAAGACAAAAGCTTTGGCAGGGTCATGAAGGTGGAGGTGAAGCAAGAGAAACATGATGATGATGAACATGAATATGATCAAGTGGTGGAGAGTGTAGGGTGCAGGAAATGTTCAGTGGTCAGGTTTACAGAGGATCAGAAAGAAGAGTTTAAGAGACAATTGTTAATATTTAAATATATGGTTTGGGGTTTACCTGTTCCCTTTCAGCTTCTTCTTCCTATTTGGAGAACTTTTTCATCTTCTTCTGTTCCTCCTCATTTTTACCTCTACACAACTTCACCTCCTTTTTTGTTAGCATATTTCCGGGGTGGGTTTAATCACTTGCAGATGACGGATCCTGAGCCAGGGAGATGCAGAAGGACAGATGGCAAGAAGTGGAGGTGCCATCAATCTGCAATTCCTAATGAGAAATACTGTGAGAAACACATGCATCGCGGTAGAAAACGTTCAAGAAAGCTTGTGGAAGCCCCTCCATATCCCCGAACCCCCAATCTAATTAACTTAAACCGAAACGATTATCCTATTAGAGACGACATCAGGCCTACATTCCTCGGAAATCCAGTTCAGAATGGACCAACTAAGGGTGTGTTGAGAGGTCCAGGCTTGATCAATAGTACTGCAAAGAGCAAGAACCATGAACAAAATAATGAGGCGAAGCGTGATATTGCTGTGAATCAAAAGATTGCTGTCTTCAACCAAGCCTCGGGATTGGATTTCTCGCCCAAAAGTGTTCTTCAAAATGATATGGGAGTCAAGTGCGCGAGAGTTGAAAGTTGCAAGAACAGCCCAGAATCATCCAAATCAAGGTGTAAGAGGACTGATGGGAAAAAGTGGCAGTGCAAGAAGGAAGTTATTCCAGATCGTAAGTATTGTGCTCAACACATCAACCGAGGCAAAAACCGCTCAGGCTCAGCAAAGCCATTTACAGGCTCATCAGCCTCTGGATCAAAATCCCAGTCTGCAGCTCGTAGTTCTACTTCGAACACCTATTTCTCAGGGACAGATCATGCCACGGGGGGCTTGGGCCTGGACACTAATCTAAAGATCTCGCTTCCGACTAGCTTGGCCCCAAATGGTAGCAGTACCTCAACCTCAAGCAGCGAGGCTACTGAAAGTGACGAGGGCACTGATGTCTCGGACATTATTGTCCTTTCCCCTTGAACGTTTTAAGGGTAGCTGTGTCCTGTGTCTACATCAAAAATGGTTTTGGACATATAATATAATGTATGTATGTATATACTAGTAGTAGTATATATTGACATAATTGTTGTAGCATGTCACCACAATTGTGTTTACTTCCAGCATTTTTGATTTTAAGGTACTCGTACTAAGGATGACTCAACTCTTGCGTCTCGACGACTCGACCTCATGGTGCATGATTTTGGTAACCCTTTATGATCAGATGTGGAAATAGCGGAGTAATATATTATGATTGTATTAACAAAGGTCAATTGATTCAGATAAAGAAAATTCAGTGGTGTTTT

*S.maritima*36182

ATGTGATGTGTTCTTGTTACATGAACTTCCAAAAAATGTTTTTACATTGTAGAAGAAACAAATTTGTGCATATAAATTCAAGAAACGTGTGGAATACTGGAATTCTATTTCATTACGTAGATGAGAGATACCATAATTAGTTATTTTTGTCATTCATATGATCATATGTATGTACAAATTGTAACATTGTTTTTCTACATGACCAATAATTTACATTGGTATAAACGATTACGGCATATAATCAGTATTCATCGTGCTCACTAAGCCGCTTTGCAAGAACATCAACTGGCAGTGAGCCTAGAGGACCTGGGTTTTCGGCATCCTCAAATGGGTCATCCTCAGAATCTACTTCAAACTCGTCTTCTTCATCGGTGGAATAATTATCAAACTCTTCGTCATCTTTGAAAAATGCGTCTTTTGATTCTGAATTCGACATGCTACCCTCACCTGAATGAACTAGAGCAGGATACGAGTGCTCAGTAGCTTGTGTCATCCTGTCGAATTCCTCCACCCTTTCTAGAAGCTCGTCTGGACCCAGATCAGTCTCGTCTAATATAGCTGCTTTGTTTGACTCTAGTGCGCGTTGCCATAGTGACATCATTCTTGGGCTTGAGATCACTTTTTTAGTTTCACCTGAATTAGAGTTGTCAAAACCATTTACAGAAGCACTTGAAGTCAACAAAGATAGAGTGTCTGGGTACATAGACTTCCACTCATGTCCCCTCCACATCAGTATCTGTTCATCGTCAAAAGACAATAGTACACAGGGAACCAGCTCCTTTAGCTTTGCTCCAATCTTTTTGTAGTCACTAGGTTCTAATCCTCGACAATCAATTTTGACAAGTTCACTTCCTTCAAAGGCTTGTCTCACTTCATTTACTAGATTGATGTAGACTCCATTTTTTGCTAGTTTACATATGGCCAGAAGGCTTTTCCCTTTTTGTCTAAACTCATCAGCCTTCTGTTTAGTCAAGCCTTCAGGAGCATCTTGAATAAGTTTTGGATAAACCGGGGCAGCTGGCTTCCAAAGCATTATAGGATATTTTGGGCGAGTGCGGTAGTTATAATTCCGGCCACGGAAAAGGTATGCTATACCACCAGTACGATAGATAATCTTACCCCCTGCTTTCTCCTCGATACATCTGCAGACATTATCCATGTCAACAGTTGGAACACCCTTGCATTTCACTTTACACACCGGCCGTCTCTTCCAATGAGAATGTATCAACTCCAACATATTGTGTGTTAGCCCATCTCTCCCAAGATTAACTTGACGATTATCCTTGATCATAGGTTGAACAAGAGCCTTAATCTCAGCTCTAGACAAAGGTTCCCCAAGAATCTCCTTTCTACTCCTTCCATCACTAGAATACTCCCCAAGTTTCCAAGGACTTTTCATATCAAACTTCTTAACATAAGGATTAGAAGAATCAAACAAAGGAACATTCCTCTTTTTCAGCAATTTCTCAGCTTTACTCTTCAGTGGAGCTTTACCAGTCCAAGGACGGGGCATAGAAGGAGGAGCAAAGGGCAAAAAGGGGGGTTCCCTAATAGCTAAAGGTTTGGCTTTCGGGGTTTCGGAGTAACTGAATTTGAACTCAAATGGAGCTCCGGGGAGAAGGTAGGAAACCCCGGAATCACCAATGACAATGGTTCGGTCATCATCGGATTGGGATTCAAAATCTTCTTGAACGGTGGAAGGAGGGGAGATGAATTGTGGGGTGGTGGGAGAAATGGGTTTGTAGTATTTGGTTTTGTTGTGGGGGATTTTGGTGAAAGCAGGGTGGTTTTGGGGAGATGGGGTTTTGGAATTTTTGGGTTTAGGAGGGTATTTGGGAGGTGGGATTGGAGGAGAGAAGGAAGGTGGTGATTGTTGAGTGGTGGTTTTGGGGGGTTTTGGTAATGAGGTGAAGAGGTTGTGGTTTGGAAGTGAAGCTACAATTGCCATTGAAGAAGATTTGAAGATGATTGTAAGAAGTTGTGGTTGTATCTTGTTTGTTAAGTGCCTTTTTTGTTT

*S.maritima*647644

CGTGAGGTGGGTTTGATTTCAATACATAGCATAAAAATGAGTTTTCTCCTTTAAATATAACTAGTTGGTGAAAGCTGTGGAATGTTATTTTGAAATCCTAGGATTTGTAATTTGTTTATGGTGTAATACTGACAGGCCGTACAATGCCAGGAAGATGAATGTGCGTTAATGTTGCTGGAACATGGCACTGATCCAAATATTCCAGATGAGTATGGAAATACCGCTCTACACTATGCTATCTACAATGAAGATAAATTAATGGCCAAAGCACTGCTCTTATACGGTGCTGATATCGAATCAAAAAACAAGGTATAGATCTACCAATTTTATCTTCAAAATACTGAAATGCATTCGTGTTAACATTGACCTGTGTAAGGGCCAGTTTTCCGTATTTGGAAGCTCAAGCATAACCTGAATGAAAATATTTTGAAATGACCTAATTATCTAAGACTTCATTTTAAATATTGTTACTTTCAAAGAAG

*S.maritima*45408

GAGTGAGTTCTCATGAGATCTAGTAGTTTAAAAGGCTGTGGAACCTCTTTCCTCTCTCTGTCTTGTTCCAACTTCTGCCATATGAAACATGTCATTGCCGCTTGGATTTCCGGTGTGGTTAGGAGGGGCCTGATCAGTGTGGGCCTGGTCAGTGGACCTAGGTCAGTGAGGACTATTTAGTGGGATAGTGGTCAGCAGGGGTCTGCTTAGAGAGGGTCTCATTAGTGGGGTCTAGTAGTGGGGTTTTGGTGAGTGGGGACCTATTGGCTGCCAGTTGTTTGGTGTCTGGTCAGTGCAAACCTGGGCTGTGGGGCTTGATCAGTGGAGACCTGGTCAGCTGGGGCTTAGTGCTGGCCTGGTCAGCATGGGCTGGGGCACTGGTGACCAGGTCAAGGGGTGCTATTCAGTGGAGGACTGGGCACATGGGACCTAGTCAGCAGACCCTGGTGGGCGTGTCCTCATCAGTGAGGCCCTTGTCAGTGGGGCCCTGGTCAGGGCAGCCTTGTCAGTGGGACCTAATCTGTAGTGTCCTGGTCAGAGAGGACTTGGTCAGTGGTGACTTTTGTAGCACTGGTCTACAGGGTGACCTGGTCAGCGGGGATCTCAGCATTTGGTGCCAGTTCAGTGGGGTCTACTCACTAGGGTCCCAGTCAGGGGCATCTGGTGACCTTAGGCCTGGTTATTAGGGGCCTGATCAGTGGCAACCTGTTCCCTGGAGGCCTGGTCAGTGGGGCCTCATCTTTGGGGCCAGGGAATGAGGTCATGATCAGTGGAACCTGATCAGTGAGGCCTTGTCAATAATGACCTAGTCAGTGAGGACTTGTCAGTAAGGACTTGGTCC

*S.maritima*647287

GAAGAGAAACTTGAAGATCAGGCTCTGATAATGAATCCAGAGGGCAATGGGCGATGTTGAAGGCTGGCAAGCAGGGGAGTGACATGATCAGATTTGGATTTTAAAGGTAATTTTGGGTGCAGTGTGGAGCATAAGCAGGACAGGCAAGGCTGGCAAGAAGGAACCAGTTAAGAGGCTGTTTTTGATCTGGGACAGAGAGAGGGTGATGACTGATCTGGGGTTGGAGAAGAAAGCACATGTTTGAGAGGGCTGTGGAAGATGGAATCGGGGAGACTCTGCCAGCAGAACATGTGGGCAAAGCGCCGATGAGCTGTTCTGGAGCACGGGGCCCAGCACAGGGTGAGAGGCAAGATGCCTGTGGGGAAATCCAGGAGATAGTTAAACACAGGCAAGGGGCTGGAGCTCAGGAGAGGCTTGGTCTGGAAGGAAAAAGTTGAAGTTCATCACAACACAGGTGGTGGTTGTCAACACTGTCTAGAAGGAGTGTACAGAAAGAGAAAAGGATGGTTTGAGGACAGAGCCCTGAGGAATGAAGAGGGGCACGCAAAGGAGCCTGAGAAGGAATGGTCAGAGAGGTGGGAGGAGAACCAGAGCCGACTGCATGACAGAGGGGGGCAGTGGTTCCACCAGGAAGAAGCCGTCAGCGGCATGGGCAGCGGCAGATGGGCCACGCAAGGTGAGCACTGGCAAGGGGCCTTAGGGTTTGCCA

*S.maritima*2539489

AAAGTGAAGAAAAGTTAAAAGAGCTTAAGAGAACTGTGGAATACCATCCAGTGGAATATCATATACATTTTAGGAATTCCAGAAAAAGAAAAGACAGAGAAAGGGGAAAGAGAATATTTCTTAAAATATAATGGGTTAAAACTTCCCAAATTTGATAGATATAAATCTGCATATTCAAGAAGCTCAGGAAACTTTAAGATAAGCTCAGAGATCCATAGTGAGAAACACCATCAAATTGGCATAAATACCAAACAAAAACCAAAAGAGAATACTGAAAGCAGCAGGAGAGAACCAACTCATTATGTATGGAAGAACTTCAATAAGAGAAACAGTTGTATTTTC

*S.maritima*1490218

CAAATTATGAAGCAAAATGCTGATACAAAAAGAAAAGATGAGAAGTTTGAAATAGGAGATGAGGTGTTTCTCAAGTTACAACCTTATCGTCAAAACTCACTAGCTAAAAGACCCTTTGAAAAACTTTCAGCTAGATTCTATGGTCCTTTCAAGATTTTACGACAGATTGGTCGAGTGGCATATCATAGGAGCTGCCTGCCACTTGCTAGATTAATCTGGTCTTCCATATTTCCCAGTTGAAGCGAGCTGTGGGAGAATCACCAGTAACTATACCATGACATGGAACTGATTGTTGAGCCAGAAGAGCTGC

*S.maritima*29634

AAGCACCCCATCTCATTCTCATTTCAGCTCTTACATTAACATATTCACTTTGATAAAATGGCGGGGCTTGGTAAGAATGGCAATACTAAATCTTCATCTTCATCTTCTTCATCTTCAAAGGGCAGAAACAATGTGAAGATACTTCCACCTGAAGAGTGGTTCAAGAAGATGAAAGAAGATCTTCTAGATGATGGAGGATTACTGGGAAGTGTGATGGGAGGTCTTATGAGGAACACTGTCAGGATGACTTGTGAAGCTCATGCTCCTAGATTTGGTGCTCTTCCTTCTCTTCTTGGCAGAAATCTTGCTTACATCCAGCTCTTAGTGGTAGATGCAGATAGAAACATCCAGGGGAAGCTGTATATGACTGGCTCACAAGTTGATCCTAATTCTCCAAAACTCATTGCAAGTTCAAGAAAGTTGTTCAAAGATGCTATCCCATGCTTGCTGAAGGATACTGAGAATTGGGTCTTCTCCCGCTCATTGGTGTTCAAAGGATATAATGGTGTGATTGATGAGGCAGTCAAATTCTCTGAATATGATCCTGTTGGTGCGCCAGATGGTCTGCTAGAACCACCATCCATGGGATCATCTGGTATTCGCTGTGCAGAGGTGTTCGTGTTTGTTGCTGGGAACTTTGAAGTGGATGAGGATGGTCTGTGCTCTCGTGAAGCAGTGAAGGAACTTCTGCAAAATCTCAATTCAGAAGAGGGCATCTCCAAACTGCGTGCCTTTGATTGTGTTGCTGCAAGTGCTTTGGTCCCAAAGAAGAAGTTGAAGGAACTCAAGGATACACATCTGAATTCGACTGCCTGAGATATATATGAATCATGTGATGTCTATAACTTTCTCCTAACATGAACTATCAAACTGAATTCCTGTAATTAATAATCTAAGGATGAAAATGTGTAAGCAACTCTAGTATGACTAGTATTTGGGTACTTCATTGCTTTGGATGCGAATCTGCGATGCAAAGTTGTTAACTAAGCTCTAGCTTCATGCCTGTGGCATGCCTAATCAAATTTCTCAACTTTCTGAGACAACAACTGTTCTGAATTATGGGCTGCTTGCTTCTGCAATGTATATATCTCGT

*S.maritima*655047

CTAAGTTTCTATTCAAAGATGAGTATAGGAAGAGGGTGTTCTTCAACTACGAAAAACGCATCAGATTAAGAAGCCCACCAGAAAAGGTCTTTGAGTACTTTGCTTCAGTCCGATCTCCAAGTGGTGAATACTTGATGGCACCAGGGGACTTGATGAGGGCAGTTGTTCCAGTTTTTCCTCCTTCAGAATCAAACTATGTTAGAGGAGGGTTTTTGATGGGGGAGCACTCCCCTGGAGAGCTACAATGTGCTCCTTCCGAGTTTTTCATGCTCTTTGATACCAACAACGATGGCTTCATATCCTTTCCTGAGTACATATTTTTGGTCACCCTACTTAGCATACCAGAATCAAGCTTTTCTGTGGCTTTTAAGATGTTTGATTTAGACCATAATGGAGAAATAGACAAGGAGGAGTTCAAGAAGGTTATGGATTTGATGCGTACATACAACAGACA

*S.maritima*28805

AAAAAAAAAAAAAAAGGGAAAAACTACTAGAAAAGGTTGTATTGTATCACTGCAGGCTTAATCATAAACATGTACTAGCTCTACACTAAAACCTAAACCGTAAAATTAGGTGGCACATTTTTTTTTTTTTTTGACAAAGCCCAATAGTTGTAGATGACGGCTTAGCCAGTAATAGAGATACAACAATTAATTAAACATACATTGCGAGGATAGCTAGTACGTGTTTTGTGGATCAAACCTTCTCCTTCTTTCACGCGTGTAAAAAGGAGTCTCATCGATTGTTCTTCTCACATATTCAATGAACGGTTGGGGAGTCTGAGGTGACCCTTCAGTTTGGGGAGAAAGACTGGTACGCACAGGAGTCAGAGGTCTAGCAACAGTTTGGCCACGCGGAGCAACAGAAGACTGAGTTTTTCTAGGTCTTTCTAGGATAAATATAAAAATAATAGCAGTCAGCATCAAGATTGTAAGCCCTGCAATTCCTGCCCATACCGTTGTATCAATAGCTGAAGCCTTTTCCTCTCCTGATTCATATTTTACTTTCACAGCTACTTTTTGGCCATTGACTGGGAGAAAAAATGTAACCACGTCCTCGAAACACTTTTGCTTTAGCAGTTCAACCTCATATAGTGCACGACCACCAATGCCAGAACCTTCTTTAAACAAGGTCCTAATCATCAACAGATCACGGTTCTTCCAATCGATGCTAACATCTGTATTACCCACTATGGTAATGATGCTTCTGTTGTAGCTTCGTGTCATATTCAGCTGGAAAGAGTCCTTTCCCATCTCCACGATTGAAAATCCACCAACAAAGAGTGCAGATGCGGAGCCTGACACATCATTAGATTCTTTAAGTTTAGCATGAATGGAAATGAAAACTTCTTGTTTTGTGTCCGTCAATTTAGGTACAGTATGCTCTAAATGTTCGGGAGAGAAAGGAAAAAAAAGGCAATATGAATCTCCAGAGCTAAGATCCTTCCATGGCTTTGCATACCCAACAAAAGTAGGCTCCACTTGACATTCGTAAGGAAGCTCAACATTTCTGCTATGAGTTCCAAATCCATGGTGACTGAATTTCACTAAAAATTTATATCCTCCACGAATAGGAGGAATGTTTGTTAGAGTGTCAGTTGGAGCATCAACTAATATGAGATCACCATTCAGCACTTTTATGGTACTCTGAAGTTTCAAGCTTGAATCTTGAAATGTCACAGAGGTTGTACCAGTGCCAACTGCCACAGCATTACCCAAATGTGGGTCCACAGAAACAACACTTTCATCGCCACTCGACCATCGACCTGATGCTTGATCATTTAGACCTTCTACGCTAAAGTTGACATTACTTCCAAGCTGAAGGATTGGATCTTGTGGATATATATGAGCTCCCACAGAAATCATGACATAGTCTATCTTTCGTGAATCACCATTAACTCTCACAGCAACAAGAGCTCTACCATGCTGTCTTGCCTTGATTATAATACTTCCATTCTTCCCATAAGCATTATCAATAGCAAGAATGTCCTTAAAGTTACTTTCAATGTCAAAAATCACCACATCATGAGCTTCAAAGAAGGGATTTCCTAAATTGTCATAGAAATAAACAGGAAGTTCAAGTTCAGCACCAACAGCAAGGTGGAATATAGACTTCCTCTGAAATCTGATTTGAGCGACCTCAGCCACCCTCACACAGGAGGCAATTTCAATTCTTCCAGTACTAAGGTCTTTTGCCTGGATACAAGCAAGACTGTTGCTGTCTCTTGTTATGATTCGATTTTCAGATATAGAAATAACACCCTTTTGCAATTCAATGTCACTTTGACCAGAACTTCCCAATACAGAGTAGGCAGTTCTTGCTTGACTTTGCTTAATCCTAGTGCTGGGTGATTTTGAATATGAAGGTAAAACCTCTGATGTAGTATAATGTGGAGGCAGAATCCATGTTATAGGAATCCCACGAGCGAGGGGAGGATCAGGCACAACCCTTACGATTGCTGACGCACTATAATTCCTCGAGTACAACAAGAAACCAGGAGATGAAAAATCACAAGTAAACGTAAGAGCAATTTTGCTTCTTCCACCCGCCCTCCCATAAAGAACCTTAATGAAGTTGATTTCGTGTTCATGCTTATAATCGAGTTTGAAATAGTCATCACTCATATGCTCAGCAAAGTGAAAGCTTAGTACCTTTTCATTCTCAATAGTCCAGTTGTAATTCTTGCAAAGCTCATAAAATGAAAACAAGTCTCCCTCAATTAAGAAAGGAAAAATGGGCATCTCACGCCCTACAGCTAGTTGCTCACTCTGCACATTCAAGATGGCATCTGATGGAATCCCTACTCTGACCTTACCATGCGCTTGACAAATGACTGCCCGTCCATTCCCATAAAAAGTGGCAACTATGGTAGCATTCCCCAGAGTGATTGCAGATAGTTGTCCCGCGAAAGCATGAACTGCCACGGCGCTGTCATTCATGCTTGTGTACTCAACAGAGACTTCCATTCTTGGTCCGCCCTTCACAGTAAGCATGTAAGATGCACCAGGAACCAAGAAAATATCGTTGGGGTGTATAATGGGTGTGGCATAAACTTCAATTGTGACGATTTGACTGGATATTTCTTTCCCTGACTGTTGTTTAGAAGTAACATATAAAGTGGTTACTCCAGCCAATCTGCCATATATTGTAAACTTGGATGCACTAAAATATCCATCACCAACTGTTGTTAAATTATCATCATCTACAAGGTCAACAATTTGGTCCTTGATGTGCACTTCTATATTCATGAATGCAAACTGTGAGGACTCAAATGTATTCCCATCATCCGTTCCAGCAATAAGATCAACAGAGGTTAAGCTGCCCTCCATCAGGCTTATCCCTTTCCCAGATACAATCTTAAGCCAGTCCACCTCTGCAACTTTGACCACTGAAGCAGCTGTGAGTGGAAGAGACAGCCCTATATCCACAACTTTTACCACTGCAGTTCCCACATCTCTAGGAACCAGCATTAATTGTAAGCAAGGTAAGCTTGATGCAGGCTGCATAACTTCTAGAACTCGGGAATTATCCACATTAACATCCAGAAAACAACTTCCTCCAATAACTGAAAGGTTCATCTTCGCATCAGGATTGAAGAAAAGCAAGTTGAATTCTGGGAATAACTGTAGCGTAGGGACAACCTGAAGACGAACAGCATCCCGAAGAACATTCTCTGAAGAATACGATAATGATGGAGCGCGATAACTGATATAATTTTGAGCCTCAGCATGAACAATGCACTGTCCTGATGCATTCTCCAGGTGTAAAAATCTTTCCCAGCTAAAAATGGACATCCGAGAAATATTATTTTCATCCCAAGAAGCCAGCTCCTGACAGTTGTTCAGTTCCATTCTTAAGGGAATAGAGGATGAGTTAGCAAAGGCATTTCCAGAATTACTAATTCCAACTGCTGATAATCTGATGGTCTGTCCATTTGCTACAGTGATAGGCATGGAGCGAATGCGACCAGGATCACGGTCAGCTTCAACGGCATTTTGTATGATGTCAATTTTGTTCACAGGTTCATCAGCTAGAAGAACAATTGAAGATGGAAAGCTACACTCAAGTCGCAATTTAGCCTCTGCAACCACTGGCAGCAGATGGTCATCTCCGATCATATTCCCACGTTTGAAAACAAGTGTGAAGGTACCAAGCACTTCACACAAAATAATATATCTGTTTCCTTCTGAAGTTTGGACCTGGTGAACTAGAGCCCCATCTTTAGGAAGGGCATAAACGTCATCAAGAACTTCAACAGTTTGAACATAATCAACTTCTTTTCTCCATCTCTCGGGTCCTCCAATGAGTAAAACATCGAAATGAGATCCAGGCGCAAGATATAAACAATCTAATGTTTTCAGTCCACCATCCATTACTCCCTCCAAATCAAACCAGTAACCACCAAACTTATTGCCATCTCCGGCTTGATGTATAATTAGTGGAGAAAACGATGCAATTGGAGCAGATGCCTGTAACAATATTTGCCTGCCAAAGTCATTATTTACATGATTAGAAAAAGTTGCATGCAAAACAGCTCGACCAGCACTGGCAGCATAAAGATGGATCCAGGAACAAGCAGGGCCAGATTTAGCTCGTTCATTCATCTTGAGATCAGTCTTCAGAGATGGCTCACTTGTAGAATTGACAATTGAGAACAAATCACTTCCAATAGACCACTTTATGCATAAACTAAAAGCATCGCAGCGATAGAAAAAATCACCATTTGATGCTTTCATTGACACTGCAGCCTGTAGATGTGACCCTACTACAGCCTCAACAGGGAAGTCTGGCAGCATAACCATTGAGGACGGTAGAACAACTTCAATGCTCACCTCATCAAAATTGAGATCATCAAAAATGGAAAAAACTTTGACATTGACTTTACCAGGCCGTTTGGCCTGAACAACACCAGTAGCAGATACAGATACAATAGTATTATCAGAGGAAAGCCACCTATAATCAGCAGGTGTAATAGCACAACCTCCCACAGCATTTAGCTCTGCTTCCTGATAAATATTAGGTGACCAAGGGAGCAGAATAGATGACAATGAAGTATTCATTTTTTCAACCCTCACTTGATCACAGACCATAACATCTTGCACAACCTTCAGAACCTCCTTAGCATCAAATTGATCAGTAGAATAAGTTAAAGAAGCTGACAGCTTTCCAAGTCCCTCTACAGTTGCCTCCAAAATTCTTGAATACTGCCAGCCATACTTGACTGCAATAGTGTCTTGAATGGGAAAATTTTTCCAATTTGGTTGATCATAGTACAATTTCACATTCTCATTCTTCGTTACATAGATTACACCTACTTTAGGCCCTCGGGAGAAAACTTTCAGTTCAATAAGATATCGTCGATCAGAGACGACATACCAACGAGCATTTGGGATTGTAGGTTTTAGCCCCTCGATAGGATCACCAGATTTAGACAGTGGAAGCAAATATAAAGATATATAATCAGGCAGCACAACATTAAGAGATGACATCTGGACATGTCCAGCAACCCTAGTATCCTCCACTACAATACTAGTTTCACCTAGTTGTAATGCACGCACTTGACCCATCATAGAATCCACTTGAACAACGGAAGTGTTTAGAGCAGACCACTGGTGAAACGGAGATGGCAGAGGGACATCTTGAGGAATGTTTTCACTAACAACTTTAAGGGTGTACTGCATTGTAGCACCAACAAGAACAAGAACAGGGGAAGGCGGATCCAGTGACATAGCTTCTGCTACAGTCAAAACAATCTTATCTCTCATATCATCAACTTGTGGCTCAAATAATTGAACAGAAACACTCTCATGCCCAATGTCAACTCCCTTCACCACAAAAAGGTCTGAGAAAACACCACTTTTTTCTAGTTCTATCTGAATGTTTAGATCACCACAAAATCCACTGCAGTCACTTAAAGGAGAATCACGCAAAGGGACATGAACAAGGTGATGTGATGACCCGTCACGCACAGGCATCAGTTGCCACAAAAACCTCAAGCCCACTAAAGAGGAGAAAACATTATCTTCGCTATCAAAAGCACGGACCCGGAGAGTCGCAAGTCCGTCTAAATCAAGTTTAACAGAGCTATGGAATATCTGGATCTTGGAGAACATATCAATATACACCTTACAGCGAACTACTGTTCCTGTGTGTACATCAGCAGCATAAACAGCTGTCTCCTTTCGACCAGTATAAGGAGCAATTGATCGTAGGCGAGCACTTGTGGAACAATGGTTACTTTCATTGAATTCAGGCACAACAGACAATATATCATGATGATCCCACGACCATTTGAAGCAACCATCAGTGGCAAGCATACGATACTCGACAGGATGAGTCAATTTAGGGGGCAAAAGAATGTTAACATCAGCAATGTGAGGTCCAGTAGAAGAATGAACACAAAAAGTATTTCCATTGAACAACAACACGAAAATGAAAACAAGAACATTCAAATAAACAAGCATCATTCTCAGGTGGAAATAAACACACAAACAAGCATCATTCGCAACAATTGATGGATGTTAATGGTGATAAACAATTGAGAATTGAGAAAATGAAATGTGTAAATGGTGGTAAGGAAGGAGGAGTTGGGTATTTGGTTTTAAGCGTAAAAAGAAATTTCTAGGAGTAGAAGTACAACTGTAGAATTGATTCCGTATTTCCC

*S.maritima*28378

CAAAATTGGCGCACAAAGTCCGAATTCCCTCCCCATTTGAAAAAAACACTTTTACAATTTACAAACCATGCTCAAATAACCACCATCATCCGCCATTAAAACCACTATGACCACCGCCAAAACCCTAACCCTAAAATCCTCAGTACTCTATTCTCATTCTCAAACCCCTCTTTCTCTCTCGTCTCTTCCACAAATTCCCCCAAATTTCCCTCTCTCAAAACTAATCCCTAAAACTCGCTTCATCATCGATGGTTTCCGAAACTCCGGCGAATTCTCCATCTCCTATTTTCTCTCTCATTTTCACGCTGATCACTACAATGGCCTCACTTCATCGTGGTCTAAAGGCATCATATATTGCTCCGAAACTACTGCTAAATTCGTCGTTAAAGTTATCGGCGTTTCGGAGAATTTCGTCGCTTCTTTACCTATGAATGAAACCGTCATAATCGATGATTGTGAGGTAACTTTAATTGACGCTAATCATTGCCCTGGTGCTGTTCAATTCTTGTTTAAAGTTCCTGTTAATGGTGGTACATTTGAGCGTTATGTTCATACTGGTGATTTTAGGTATTGTAGTGATATGAAATTGATTTCTGTGTTGAATGATTATATTGGCTGTGATGCTGTTTTTCTCGATACGACGTATTGTGATCCGAAATTTGTGTTTCCGTCGCAAGAGGAGTCTATTGATCACGTGGTTAGTGTGATAGAGAGGGTTGGGATGGAGAATAGGAATGTTAGGAAGAGTGTTTTGTTTCTTGTTGCGACGTATGTTATTGGAAAGGAGAGGATTTTATTGGAGATTTCGCGGAGGTTGGGGTGTAAGGTGTGTGTAGATAGTAGGAAAAAGGGTGTTTTAAGGGTGCTAGGGTATGAGGATGAGGGCGTGTTTACGGAGGATGAGTCGGCTAGTGATGTTCATGTTGTTAGCTGGAATGTGTTAGGTGAGACTTGGCCGTATTTTCGACCTAATTTTGGGAAGATGAAGGAAATTATGGCTGAGAAGGGGTATAAGAAGGTTGTGGGATTTGTTCCTACTGGGTGGACATATGAGGTGAAGAAAAACAAGTTTGCTGTAAGGACGAAGGACTCATTTGAGATTCATCTAGTGCCTTATAGTGAGCATTCTAACTACAATGAACTTAGAGAATATGTGAAGTTTTTGAAACCAAAGCGTGTAATTCCAACTGTAGGATCTGATGTTGAGAATGTGGACAGTAAACATGCTATTAAAATGAAAAAGCATTTTGCTGGATTGGTGGATGAGATGGCAAATAAGCATGAGTTTTTGACGGGATTTTACCGTGTACCCCAGGGAGAAGGAAAATGTGATGTAGATGATCAGGAAACTGAGTTGCACAAAGGTGCTGAACAAGTAAAAGAAGAGTTTGATGTGTGTGAGATAAAGCATGATCATACATTTGAGGCTGGTTCCACGCATTCTGTAGCTTCTGTGCTTGAAGCTGGTACGAAAAATTTAACCTCCTTGAGTGATAAGGAAAAAAATAATATCATGCAAGAGCTCCAGGACTGTTTGCCTAGCTGGGTTTCTGAAGATCAAATGTTAGAACTCATCAAATTATATGGTAGAAGTATTGTTGATGCTGTTTCTCATTTTTATGAACATGAAACAGAATTTCATGAGCAGAGTGGAAATCCTCTTCCTTCATCTCAATCAAATGCAACAAGTGATTGCACTACATTTGTAAATCCTAGTTTTGAGGACAGTAGTCTTCAGACATCAGTAAAATCACCTAAGAAAAACCTTAAATTTCCACCGTTGAAGCAGTCAGTTGCTAGTAGTGTTTCCCCTGGTAAAAGGAAGAGAAATCCAGAGAATAAGCAAAAGAAGAAGGGAAAAGTTAGTCTTAATCCGGAGCCTGGTGGAATTAAGCAATCTACTATAACTAAGTTTTTCTGTAAAAGTAGCAGTGTTGGGAAGGGAAGCATCAGTGCCCCTCTGTCTGGGTCTAACACTGAAGATAAGAGATTATCCCCTGTACGTCCAGCTATACATTACAAGGAAGAGTTGACACAATTTATTCAGATTATTGATGGTAGTGAATCTATGAAAGATTATGCTACTGAATTGTTAGAAAAGGCAGAAGGAAATGTTGATTTGGCACTGGACATGTATTACAGTAATTCTGAAGCTCAATCTGGTGGTGTTCAGAAAACGGTAGATGTTAGTAACAAATTACAAGCATGCGAAGGTCCAGATGCTGGCCTCTCCAGCCAAGTGAAAGAACCGTCTGAAGCTGGCAACAAAATTTATCAATTTGGAAAAGATTCGTTAGCTGACAAAATTGCTGTTAGCTATATATCTCTACCACCAGAAAAGTATTCTCCAATAGAACATGCTTGCTGGAAACAAGGTCAGCCTGCTCCATATATACACTTGGCACGGGCTTTTGATCTTGTTGAAAGTGAAAAGGGAAAAATTAAAGCTACCTCTATGCTTTCCAATATGTTTAGAAGTTTGCTAGCTTTGTCTCCTGAGGATGTTCTACCTGCTGTGTATCTTTGCACAAATAAGATTGCTGGTGACCATGAAAATATGGAGCTAAACATTGGTGGAAGTTTGGTTACTTCTGCTCTTGAAGAGGCATGTAAAACCAATAGAGCTAAGATAAAAGAAATGTACAACACTTACGGTGATCTTGGTGATGTTGCTCAAGAGTGTCGGCAAACACAATCACTGCTTCGTCCTCCTTCACCACTTTTGATTCGAGATGTATTTTCTGTACTTCGAGAAATTAGTGTGCAGACAGGTAGTGGAAGCACTAGGAGGAGGAAAAGCCTAATTGTTGGTTTGATGAGGTCTTGCAGAGAGAAGGAAGTGAAGTTTATTGTCCGAACGTTGGTGAGGAACTTACGAATTGGAGCCATGATGAGGACAGTTCTTCCTGCACTGGCTCAAGCAGTTGCTCTGAACTGTTATAATGAAGGAGCAGTTAAAAATTTAAAGGAATTGCTTCAGGGCCTTTCTGCTGCTGTCATTGAAGCTTATAATATTCTTCCAGACTTGGATTTATTGGTTCCCTCTCTTATGAGCAAAGGCATAGAATTCTCCACCGCTACGTTGACTATGGTGCCCGGCATTCCTATCAGGCCCATGCTTGCTAAAATAACAAATGGTGTTGCGCAAGCATTAAAGCTATTTCAAAAGAAAGCTTTCACCTGTGAGTATAAGTATGATGGTCAACGTGCCCAAATCCACAAACTTGATGATGGTTCTGTGCGTATTTTTTCACGAAAAGGGGTTGAGACAACATCAAAATTCCCAGACTTGGTGCATATTATTCAGGAATCGTGTTCACCTTCTGCATTGTCTTTCATCATTGATGCAGAGGTTGTTGGAATTGACCGCAAGAATGAACTTAAATATATGTCCTTCCAAGAACTATCTGCCCGTGAGAGAGGGAGCAAAAATTCCTTGGTTTCATTGGATGGAATAAAGGTTGACATCTGTGTCCTTGCATTTGATGCCATGTTCGCCAATGGAGAGAAGTTGTTGGCTTTCCCACTTCGGCAAAGACGGCAGTTTATGAAAGATCTCTTCATAGGGGAAAAGCCAGGTTTTTTTGAATATGTAAAGGAGATAACTGTAGAAGCAGATGAAGCTCGTCCTGACAGTGAATCCTCTTTGACAAAGATTAATGCTTTCCTTGAAGATTCATTTCAGTCCTCTTGTGAAGGAATTATGGTTAAAACACTGGATGTTGATGCTGGTTACTTTGCTTCAAAACGTGCAGATTCATGGTTGAAGGTAAAGAGAGATTATATAGATGGAATGGGGGACTCCCTTGATTTAGTCCCAATTGGCGCTTGGCATGGCAATGGGAGAAAGGCAGGATGGTATAGTCCATTTCTTATGGCTTGTTACAATCCTGATTCTGAGGAATTCGAAAGTTTATGCCGGGTAATGACAGGGCTATGTGATTCATTCTACAAAGAGATGAAAGACTTTTTTTCTGGAGACAATATAATGACCAAGAAGCCCCTATATTACCGAACAGATGAAGTTCCTGATATGTGGTTTTCAGCTGAATTGGTCTGGGAGATAAGAGGTGCGGAACTCACCGTATCACCTGTTCACAAAGCTGCCGTTGGAATTGTTCATCCTTCACGAGGGATTTCTATGAGGTTTCCAAGATTTCTCCGTTCTCTCTCAGACAGACGACCAGAAGACTGCAGTACCTCAACAGATGTTGCTGAAATGTTTCATTCTCAGATTCGGAAAATGGATTTAATTGGTGTTCATTAGAACTTTTTTCATAAAATTTAGTGTAAATATAAATTATGCACTTCCTTGTACAATATGAACTTCAAGTAATCCTCTATGTAATTGTAATCTTTTGCTAGTTAAACATGTCTGACTAATTTGGCTCCAAGTA

*S.maritima*30069

TTATTACTAAAATATTCAATCTTGCTCTTATTTACGAGATTGCCATTTTGATGCATTTTGCTAAACACATCTTTTGAGTAACTTGGCCAAGCAAAGCTTCTGTTCTTCAGAATTCAACTCAAAAAAGTAAAACTTCAAATCAGAGATTCCTAAATTAGGGCAAATGACACATTGATTGAAAAATTGTTCTACTGCAAAAAATCTTCAAGATTAATCAGAAATAGGAGGGAAGGAGAGCGAAAATGGTGGTGATTTATAAGGAGAAAACTGTTTCTCGGAAAAATAAGGTTGCAGAAGGTTGTGGAACTAGTAAAAATGTTGGTATGAGTAAATTCAAGGTTTATTCGGAGAAGAAGAAGTTCAATGATGGAAATGGCGATAATGATGATCCAAAGACTTCGAGAGCGTCGCTAGTTTCAAGTAAGGGGAGTGTGATGACTAGTGCTAAGATGAAAGTTGGCAAAAGTGGTGCTCCGATTGCTCGAAGCAGCAATGACTCTCTTGACAAGTTAAGGCTTAGAAGGAAAGCATTAGCGGATGTTAGCAACACGCGGGGGCAATCATCTAGATTTGAGAAGCCTAGTGGTACAAGGAGTTCACTTACTTTTGGCACCTATACCACTACCACTTTGTCTAATCGATCTCTTCCAGGAAAAATTAAGGCAAATATAACTGGCGGCATCACAACCGTAAACACTTCTATGAAAGCCGGCAACCAGGCCCTGAGTACATCATCAAATATCCGCAGGGTCCAAGTGAACAAAACCATTGAAAACACTACTAGGAAGTCTTTACCAGTAATGAAGAAACCTATTCAGTTAGATTCTGGTAAAGTACAGGGAGATAACAGAAGCACGGAGAAGTCTGCAAGAAAAACTGGTATCCCAGTGAAAACGAGAAATGGTGGAAAACTAATTCCTCAAGTGAGCACTCTCAAAGGAAAAGGTTTGAAAGAAAAATTGAATGACGGCTTCACACTAAAAGGTTCGATAAATCATATAGATCATGATGCTATTGGAGGGGTAAGAAAATTAGTCAAGCCAATTGCAAGGACCAATTTACAAATGCCCAATTACAGAAAGAACCTTAAATCCAAGAGCATCTGTAGTTTGCCTAAATCTCTTGGTACCACTTCTACCCAAGAAGTGAAGGTGGCCACTTCTACTGAAGCAAAACCTTCTGAGCCTATTGGTTCCCATGAAGAAGTTACTGAAGAATCAATCACAGCAGAAGGCATTGATAACATGAAATCACAGATTCCAGAATTGACAGAGACCACAGCCACTAAAAAATCTGGTCGCAGGAAATCGTATACTTATTCATTAATATCAAGATCAAAGTTACTAAAAATGAGGAATGAAGAAAAGGAACATGAACTGTTGCCGAACATTGATGATGAGGGTAATCCTCTTGAAGTTGCAGAGTATGTGGATGATATCTATGAGTATTATTGGATGATGGAGGCACAGAGTAACCCTGTGGAAAATTGTATGATGAACCAAACAGATGCTACACCACGGATGCGAGGCATATTGGTTAATTGGCTTGTAGAGGTACACCTCAAATTTGATCTGATGCCAGAAACACTTTATTTGTCAGTAGCCCTGCTTGACCGATATCTCTCATTAGTCACATTAAAGAAAAAACAACTTCAGTTGGTTGGTCTTGTGTCTCTTTTGCTTGCATCAAAGTATGAGGACTACTGGCACCCACGGGTCAAAGACTTGATCAGCATCTCAGCAGAGTCATACACCCGGAAACAAATGCTTGAAATGGAAAAGAGCATTCTCAAGAAGCTAAAATTTCGGTTGAATCTACCCACAGCTTATGTCTTCATGCTAAGGTTTCTTAAGGCTTCTCAGTCAGACAAAAAGCTTGAACATCTCGCATTTTACCTCATTGAGTTGTGCTTGGTTGATTATGACGCGTTGAGGTTCAGGCCATCATTTTTAAGTGCGTCAGCAATCTATGTTGCTCGTTGTACCCTGAACAAGGTTCCAGCATGGACGTCATTGCTTGGAAAGCATGCACGTTACGAGGAACATCAGATTAGGGATTGTGCAGATATGATCCTGCGAATCCACAAAGCTGCAAGTACAAGTGTCCTGAAGGTCACCTATGACAAGTATATGCAGCCTGAGTTTGGGAGTGCTGCAGCTGTAAGACATTTGGACAGTCTTCCTAGTCTATGAAACTCGTCTACATGAATCAAACCTCTTGCGATTTTTTTTAAGGCGGTGGAGTTTGGGCTGCATTCATAGTTCAGATTATATGTAAGAATTAGTGAATTCTGTACTCCTTAACATACAAGGTATATTGATCATTGTAGCGATGTCAGGTTGTGTCTGTACCATGGTGTATAATTTGCATTGTTTTGTTTGTGTACATTTCACGACTAGTGATATACAACATAATCGTTCAGTACTTACACAGCTCAATATTGTATTTGTAATTTTCCGAGGA

*S.maritima*10708

TTCATGATCCTTGGTTTAATAAGGACACTGCATTCCCTTTGACAGAAAGAGATCGTCTTGGACTTCGTGGTCTACTTCCACCTCGTATTGTATCTTTTGAACAACAGTATGCCCGTTTCATGGAATCCTATAGATCGTTGGAAAGAAATACACAGGGGCAGCCAGAAGGAGCTATAGCACTGGCAAAATGGAGGATTTTGAACAGACTGCATGACAGGAATGAGACATTGTATTACAGAGTCCTTATTGATAATATCAAAGACTTCGCTCCTATTATATATACTCCAACTGTAGGGCTGGTGTGTCAAAACTACTCTGGTTTGTTCAGACGTCCACGTGGAATGTATTTCAGTGCCAAGGATAAGGGAGAGATGATGTCCATGATCTATAATTGGCCAGCTCAACACGTTGATATGATAGTCGTCACAGATGGCAGCCGAATTCTTGGCTTGGGAGACCTTGGCGTTCAGGGAATCGGCATACCTATCGGTAAACTTGATGTATATGTTGCTGCTGCAGGAATGAACCCACAGCGGGTACTTCCCATTATGCTTGATGTGGGGACCAACAACCAAACGCTTCTTGAAGATCCTCTCTACTTAGGTCTCAGACAACCTAGGCTAGAAGGGGAAGAGTATCTAGCTATACTTGATGAATTTATGGAAGCTATCCATACACGTTGGCCCAAAGCCATTGTGCAGTTTGAAGACTTCCAAATGAAATGGGCTTTTGAAACACTGGAAAGATATCGTAAGAAGGTTTGCATGTTTAATGATGACATTCAGGGAACTGCCGGTGTTGCCTTGGCTGGGCTGCTGGGTGCTGTCAGAGCACAAGGTCGTCCATTGACTGATTTTGTGAAACAGAAAATTGTTGTGGCTGGAGCAGGGAGTGCAGGGCTTGGTGTCCTTAACATGGCAGTGCAGGCAGTTTCCAGGATGGCTGGGGCAAATGGGATCCCTCCCAAGCACCAGTTTTACCTAATTGATAAAGATGGTCTTGTCACTAAAGAGAGAAAGAATTTAGACCCAGCTGCTGCACCTTTTGCAAAAAATCCAGAAGAAACTGAGGGGCTTAGGGAGGGAGCTAATCTCGTTGAAGTGGTTAGAAAAGTCAAGCCAGATGTGCTTCTTGGTTTGTCTGGTGTTGGTGGTATCTTCGATGAAGAGGTTTTGAAAGCCATGCGAGAATCCGACAGTCCTAGACCTTCTATCTTTGCCATGTCAAATCCTACACTCAAAGCTGAATGTACACCTGCTGATGCTTTCAAATATGCTGGAGAAAACATTGTTTTTGCGAGTGGAAGTCCGTTTCAGAATGTAGATCTTGGGAATGGAAAAGTGGGTCATGTCAATCAAGCAAATAACATGTACTTGTTTCCAGGGATTGGGTTGGGAGCTTTGCTGTCTGGTGCTCGCATAATTACTGACGAAATGTTGCAAGCTGCCGCTGAATGTCTTGCTTCCTACATCACAGATGAGGAAATTCGTAGAGGTGTTTTGTATCCATCTATTGGAAGTATAAGAGAAATTACAGCAGAAGTTGGAGCTGCTGTGCTGCGAGAAGCTGTTGCTGAAGACTTGGCTGAAGGCCATGGTGAAGTGGGGCGAAAAGAGCTCAGATCTATGTCAAAGGAAGCAAGTCTGGAATATGTCAAGCGCAGTATGTGGTTTCCAGTCTACAGCCCTCTTGTTCACGATAAATGAGGCTCTGACAATTACTTCTATCATGCGCTGCCTCCGCTGGGCAGTTTGTTTCTCCCTGGAATCAGTAAAAACTGGCTCAGAAGACAGGCGTATTGTTTCAACAATTAACAGAAAAATTAGAGTTTAGGAAACCTGTGAAAATTTTGCCAGCTTCATTTTTTTTGTATTGAAACTATTGAAATTAGAAACCTGGAATTTGTAGAATTACTTCTCTAGGGAATGATCTATTTTGGGGTCCAATATCCTAGAGATTGTTTGTATGCAGATGCATTCTATTGTATTCTATCACATCTAACATCTAATAAAGTCACTTGTAAAAAATTGGGTGTTCAAATGTTTTTTT

*S.maritima*25890

CAAAAAAAAAGAAAAGAAGAAGAAGAAGAAGAAAAAGTTGAGGAGAAATATGATGATGATGAACAGCAAGAATTAGACTGGAAAACAGATGAAGAGTTCAAGAAATTTATGGGAAATCCTTCAATTGAAGCTGCAATTAAGTTAGAGAAGAAAAGGGCTGATAGAAAGCTTAAAGAACTTGATGCTGAGAGTAATAGTGATAATCCAATTGTTGGGGTTTTCAGTAAAATTGCTCGTGAGAATTTGTTGAGAGAGAAAGAGAGGTTGGAGAAAGTTGAACAAGCTTTTAAGGCACTTGATCTTAACAAATTGAAGAATTGCTTTGGATTCGACACGTTCTTTGCGACTGATGTACGAAGGTTTGGGGATGGAGGGATTTTTGTAGGGAATTTGAGAAAGCCAATTGAGGAAGTTATGCCAAAATTGGAGAAGAAACTTTCTGAGGCAGCAGGAACAGAGGTTATGTTATGGTTTATGGAAGAGAAGAAGGATGATATTACAAAACAGGTATGTTTGGTGCAACCAAAATCAGAAATTGATCTCCAGTTTGAATCAACTAAGCTGAGCACTCCTTGGGGGTATCTTAGTGCTATAGCTTTGGGTGTTACAACTTTTGGAACCATTGCTTTGACGAGTGGCTTCTTTGTTAAGCCTGGTGCAACATTTGATGACTACTTGGCTAATGTTGTCCCTCTTTTTGGTGGTTTCCTGACCATCTTGGGTGTTTCTGAGATTGCCACTAGGCTGACAGCAAATCGTTATGGGGTGAAGCTCAGCCCATCATTTCTTGTTCCATCAAATTGGACGGGGTGTTTAGGAGTAATGAATAACTACGAGTCCCTCCTTCCCAATAAGAAGGCTCTTTTTGATATTCCTGTGGCTCGTACAGCCAGTGCATACTTGACTTCCCTAGGACTTGCTGTTGCTGCCTTTGTATCAGACGGTAGCTTCAACGGAGGGGATAATGCACTGTTTGTGAGGCCTCAATTTTTCGAGAACAATCCTTTGTTTTCTTTCATCCAATATGTGATTGGACCTTATGCTGATGAGCTTGGAAATGTACTGCCTAATGCGGTGGAAGGAGTGGGGGTACCTGTTGATCCTCTTGCATTTGCTGGACTTTTAGGAATGGTAGTGACTTCACTAAACTTGTTACCCTGTGGAAGACTTGAAGGGGGTAGAATTGCACAGGCCATGTTTGGAAGGAACACAGCGACATTGCTGTCATTCACCACTTCTTTGCTGCTTGGCATTGGAGGTTTAAGTGGCAGTGTTATCTGTCTTGCGTGGGGATTGTTTGCTACCTTCTTCCGAGGAGGAGAAGAAATTCCTGCTAAGGATGAAATAACACCGTTAGGTGATGATCGATATGCTTGGGGTGTCGTCCTCTTCCTTGTGTGCTTCTTGACTCTCTTTCCGAATGGAGGAGGGACCTTCTCCAGCTCCTTCTTCAGTGACCCATTTTTCAGAGGCAATTTGTAGATATGGTGATCTTGGCTAATAACTGTATATTCCTTAGGTCTTAACTAATGTTATTTTTCTCTTCCCTTTGTATATTTTCTTGATCCAAAATTTTGTAAATCAAACGATTTTGGATGCTTTTGCTTCTTCAGTCTTCTGTATGGGCTGCCTCTTAAAATCAACGGAGTAAAAGAGCTATTGGGTTTCTAATTTATCAATTTGAGATACAAACTATTAAACTAGACATCTCAAGTGACATTTATGTAAATAAATTGAAAAAAACTTGCCAAATTACTCTCATTTTATGAATAAAATTAAT

*S.maritima*36451

ATTTAAGTACTCAAAATGAAAACGACAGTTTTGCCAAGTGTGTGACATGAATTCCATTTCACTCTGTAAAAAAAAAGTATGTAGAAAAAAAAGACACATCTTGAGCTACCATTCATGTTTCTATATGCAAAAGAAGATGACAGACAATTAAGTGTACAAACGCTGCGTATCACAAAATTTTTGATTAGCAACTGCAAGTCCAAAACTGCAGCTCATTCATTGCAAAAAGAAAAAACTCTTTTCTTAAACAAACATGCCAAATCCACTACATATTCTCTCATCTGCCTAGAATTGCTGTCAAGCTTCAGTAACCCCCGGCTTTTCTTGGATAGCTTAGTTGCAATATCTAGCACCAAGCTGACCTTGTCTCTACAGCTTGTTAACACTTGCAAGACCATAGCTGAATATGTCGGATGCGATACCAGTCGGTTAAGTAAAATCAAAGCCTCTCGCATAAGTAATGTTCTCTCTTTGGATGATTCTGGAGCAGGGCAAGACTCCAATGCTTCAGCATCAATTTCTGACACCAGTACTTGCAGAACTAGTCCCAGAAAGTTTGTTGATGTTGCCTCTCTTTTGGGAAGTTTGTAGAACAGGAAAATTTCCAGACCACATTTCCCAGAAGAAGCCAAAAAAGATAGCACAACTATCGCATTTCGACGAAGGTTAAGATCCTTCGCAACATACTCAGAGCAAAGCACACATCCGGCCAATCCCTCAAGAACAGCACGAGACTTTTGACAGTCCAAAACTTTTCCATCACCATTTGAAGATTGTGCAGGACTGTGCGCTTCTTTACAACTTGAACAAAAAGCTGCTAGTACATTTGGACAGTTCAGAAGTAGATACAACAGATGTACAGCTTCTTTACGGAAAAACGACCCAGCTTCCTTTCTCAACAGCAAACTAACAGCTTCAAAAACAAGTGACCCACCAAACTTTCCTCTATCAGACCAAGGATCCGTCTTCATGAGAATTATATTCATGATGATAACTGCTTCCACCCTTGCCTGCTTGTCAGTCAATTTAACGGCAAGTTGACACACTGACGTAAATAGATGAAGCCAGTCTACATAACAAGTCAAGGATAGAGTTTCATTTTCTGCATGGCATGCCTCGTCTCCACTAATATCACACACACCACCACCTTTTCCCACTTCAGAATCAAGAATATGTGTAGCATCAGAGCCAGGGTAACACTTTTCCACCTTGATATTATCCCTTCTAGTGCGCGTTCTTTCAATACTCAGAACATGAATCAGAGAAATATGCAATATATGCAAAGATCTATGCACAACAAGAGCTTGTACATTCTCAAGGGTACATAGATCAAGTACTGCTTCCAAGAAGGTGTTTATATGAATTACATTCTCAGTAAGCTCCATCAACAGGAAGTATAGCTGTGATACCCTGGCAACTTCAGCAGAATGTAGATCTAGGAACGTTTCAGGCAATGGTGCACGAGTAAACCACTCACTCTTCATCTTGGAGGTCATATCCGGACCCAAACAACCAAAAAGATCAAAAAAGTCACTTGCACATGCCTCAAGTAACTTAGCAGCAAAATTTCTTCCTAAATGACCTCTTACAGGAGACCAGATTGATAGCAGTTCTGAATGATATCTGTATGTAGTACTGCCACTAGTGCTTGGTTTTAGTGATTCATCTACTGCTTCTGTTTGAATGCCAGTACTTCTTTTCTCTTGCTCAGCAGCAACATTAACACACTCAAAAGTAGGCGAAATCCCAGTCCTAGAAAAATCTTTCGCATCACTCCTGGATTTCTTGTTGGAATGTATAAAGTTGAGCTGTTTCTCCTTCTCTCTTTGCTTCTTAAGCTCAGAACACTCTTGTTTCAAATCCAAAAGCTCCTTAGACACACCACTTAACTCCTTCTTAAGGCGTTCAATTTCGCGGTGTTGATCAGTCAAAAGAGGAGGAGGTGGTGAAGAAACATCTTGAGAACGAGTAGAAGTGGAAGGGTTTCGCTGCGAAAGTTGTCGAGGAGGTGAGTGACTATGAACGAAAGTAGTTTGCGGAAGTTGTGGAGGATTTGAATTGAAAAGAAGTTGGTGTTGTTGTTGTTGAAGATGA

*S.maritima*10995

CCCAAACAAAGCACAACCTCTAAATTTTTTTCACAATTTCAGTTATTCCACGTGTCCTCTTTTACACCCACTAACTGAAACAAAACAGAAAATTGTTTGCTAGAGCCTCTTCAGTCGCTCCTTAACTCCCCTGTTCTTTGCTCTGTTTTTCTGAAAAGCTTCAACACTTTCCCCTCAAAAAATTTAAGAGGGAGAAAAAAAATCGAAGTTTAACCCAAAAAAATTTCTCAAACCCTTTTCAATTCTTCTATCAAAGAACAATACCCATAATTTAGTTTCCCCAATTTCTCACTAAAAATCACTTCTTTTCTTCTTTATACTCTGTTTTTCAAAACAGAGTATTCAAAAATGTCTTCAATTGTTTCCTCAGCATTCACTTTACCATCATCTAAAACTGATTTCCTCTCATCAATCAATCAAAAACAGTACATTCTCCACTCTTTCCTCCCTAAAAAAACCAGGCAATTCAACTCAAGAACAACCAAAAATGGCAAAATCAAATGTGTTGTAGCTGGAAATGGGCTTTTCACTCAAACTTCACCAGAAGTTCGACGAATTGTCCCTGAAAACAAACCAAATCTCCCAAGTGTAAAAATTGTGTATGTAGTGTTAGAAGCTCAGTATCAGTCTTCGCTTTCGGCCGCGGTCCGAACCCTCAACAAGACCTCAAATTTTGCAAATTTTGAGGTTGTGGGTTACTTAGTTGAGGAACTTAGAGACAAAGAAACATACCAAAGCTTCTGCAAAGATCTTGAAGATGCCAACATTTTTATTGGATCATTGATTTTTGTGGAGGAGCTAGCAATCAAGGTGAAGGATGCTGTGGAAAAGGAGAGGGAAAGAATGGATGCAGTTCTTGTGTTCCCTTCAATGCCAGAGGTAATGAGGCTCAATAAATTGGGTTCTTTTAGTATGTCTCAATTAGGGCAATCAAAAAGCCCATTTTTTCAGCTTTTTAAGAAGAAGAAACAAGGTGCTGGATTTGCTGATAGTATGCTAAAATTAGTAAGAACATTGCCTAAAGTACTGAAATATTTGCCTAGTGATAAGGCTCAAGATGCTAGGCTATATATCCTAAGTTTGCAGTTTTGGTTAGGTGGGTCCCCTGAGAATTTGATTAATTTTGTTAAAATGATATCTGGGTCATATGTTCCTGCATTGAAAAGTGTGAAGATTGAGTATTCTGACCCAGTTTTATTCTTAGATAGTGGGGTTTGGCACCCTCTAGCTCCTTGTATGTATGATGATGTGAAGGAGTATTTGAATTGGTATGGAACTAGGAAAGATGCAAGTGCTAAGCTTAAGAGCAAAAATGCACCTGTAATTGGGTTGGTTCTTCAGAGGAGTCATATTGTAACTGGTGATGAGTCTCACTATGTTGCTGTGATTATGGAGTTGGAGGCAAGAGGGGCTAAGGTTATCCCTATTTTCGCTGGGGGGCTCGATTTTTCGGGGCCTGTTGAGAAGTTTTTCATTGATCCAATCACTAAAAAGCCTATGGTGAACTCTGTGATATCACTCACTGGGTTTGCTCTTGTTGGTGGGCCGGCACGACAGGATCATCCTCGGGCTGTTGAGGCCTTGATGAAGCTTGATGTGCCTTACGTTGTTGCTGTGCCTTTGGTGTTTCAGACTACTGAAGAGTGGCTGAATAGTACCTTGGGTTTGCACCCTATTCAGGTTGCTTTGCAGGTTGCTCTTCCTGAGCTTGATGGTGGCATGGAACCTATTGTTTTCGCTGGACGTGACCCGAGAACAGGAAAATCACATGCTCTTCACAAGAGAGTTGAGCAGCTCTGCACAAGAGCAATCAAGTGGGGTGAATTGAAGAGGAAGACAAAGGCTGAGAAGAAGCTGGCTATTACAGTTTTCAGCTTCCCTCCAGACAAAGGAAATGTTGGAACTGCGGCATACCTAAATGTGTTTGCGTCCATTTATTCTGTCCTAGAAGACCTAAAAAAGGATGGTTACAACATTGAAGGTCTTCCAGAGAATGCTGAAGCCCTGATTGAAGATGTGATCCATGACAAAGAGGCGCAGTTCAATAGCCCAAATCTCAACGTTGCATACAAAATGAGTGTCAAGGAGTACCAGAAACTAACTCCTTATGCAACCCTTTTGGAAGAGAACTGGGGAAAAGCCCCAGGAAATCTGAACTCTGATGGAGAGAACCTCTTGGTGTATGGAAAACAATATGGGAATGTCTTCATCGGAGTTCAGCCTACTTTCGGATATGAGGGTGATCCTATGAGGCTGCTTTTCTCTAAATCTGCTAGCCCTCATCATGGGTTTGCTGCGTATTACTCATTTGTTGAGAAAATATTCGGAGCTGATGCTGTTCTTCATTTCGGAACTCATGGCTCCCTAGAATTCATGCCAGGAAAGCAAGTGGGAATGAGTGATGCTTGTTTCCCGGACAGTCTCATTGGAAACATTCCAAATGTATACTATTATGCAGCTAATAACCCTTCTGAAGCAACAATAGCTAAACGTCGTAGTTATGCAAACACTATCAGCTATCTGACTCCACCAGCAGAAAATGCAGGACTTTACAAGGGTCTTAAACAGCTGAGCGAGCTCATTTCTTCGTACCAATCTCTTAAAGACACTGGCAGAGGCCAACAGATTGTGAGTTCCATCGTCAGCACTGCTAGGCAATGCAATCTTGACAAAGATGTCGATCTTCCTGAGGATGGGGTAGAGATCTCCGCAAAAGAAAGAGATCTTGTAGTTGGAAAAGTCTACTCTAAGATCATGGAGATTGAGTCTCGACTTCTGCCTTGTGGGCTCCATGTGATCGGTGAGCCTCCTTCAGCCTTGGAAGCAGTGGCAACTTTGGTAAATATTGCTGCTTTGGACCGTCCTGAAGAGGGAATTTTCGCCCTCCCTTCTATACTTGCTGAGACCGTTGGAAGGAGTATTGAAGATGTGTACAGAGGAAGTGACAAGGGAATCCTGAAAGATGTTGAATTACTAAGGCAAATTACTGATGCATCACGAGGAGCTGTCTCTGCATTTGTTGAAAAAACAACCAATGACAAGGGTCAAGTTGTGAATGTCAATGATAGATTGACTTCAATCCTTGGGTTCGGATTGAACGAACCATGGATCCAGTACTTGTCAAACACTAAGTTTTACACAGCTGATAGGGAGAAACTTAGGGTTCTTTTTGCATTCCTAGGAGAGTGCTTAAAGCTTGTTGTGGCTGATAATGAGTTAGGAAGTTTAAAACAAGCTCTTGAAGGAAAGTTTGTGGAGCCCGGTCCTGGTGGAGACCCGATTAGGAACCCAAAAGTTCTGCCTACTGGGAAAAACATCCATGCTTTGGACCCACAAGCTATCCCTACTACTGCAGCCATGCAGAGTGCTATGGTGGTTGTGGATAGACTGCTCGAGCGTCAGAAGGCTGATAACGGAGGGAAGTTTCCTGAAACGGTTGCACTTGTGTTGTGGGGAACTGATAACATTAAGACTTATGGAGAGTCACTTGGTCAGGTTCTGTGGATGATTGGGGTGAGACCAATCGCCGACGCCTTTGGTAGAGTCAACCGCGTTGAACCTGTCAGTCTTGAGGAGCTTGGAAGGCCTAGGATTGATGTCGTTGTCAACTGCTCAGGTGTCTTCAGAGACTTGTTTATCAACCAGATGAACCTTCTTGACCGTGCGATTAAGATGGTAGCAGAGCTTGATGAGCCAGAGGACCAGAATTATGTGAGAAAGCATGCAATGGAGCAAGCCAAAACACTTGGTGTTGACGTTAGAGAGGCTGCTACTAGGATTTTTTCGAATGCTTCAGGCTCCTACTCCTCAAATATCAACCTTGCAGTCGAGAATTCTTCATGGAATGATGAGAAGCAATTGCAAGACATGTACTTGAGTAGAAAGTCATTTGCCTTCGACAGTGATGCACCTGGTGTTGGAATGACTGAGAAGCGACAGGTGTTTGAGATGGCTCTTGCAACCGCAGATGCTACTTTCCAGAATCTTGATTCTTCAGAGATCTCTCTGACTGATGTCAGCCACTACTTTGATTCGGACCCCACAAATCTTGTGCAGGGTCTCAGGAAAGATGGGAAGAAGCCAAGTGCTTACATTGCTGATACTACAACAGCAAATGCTCAGGTGCGTACTCTATCTGAGACAGTTCGTCTGGATGCAAGAACAAAGCTGTTGAACCCCAAGTGGTATGAAGGAATGCTGTCAACTGGCTATGAGGGTGTACGTGAGATTGAGAAGCGACTCACCAACACTGTTGGATGGAGTGCAACCTCAGGACAAGTGGACAACTGGGTGTATGAAGAGGCAAACACGACATTTATTAAAGACGAGGAGATGCTGCAAAAGCTGATGAACACCAACCCAAACTCTTTCAGAAAGTTGTTGCAGACCTTCTTAGAAGCCAATGGAAGGGGCTACTGGGAAACTTCGGAGGAAAACATTGAGAAATTGAGGCAGCTGTATTCTGAAGTTGAAGACAAGATTGAAGGTGTTGATAGGTAAATTATGTAAAATTGTTACAATAAGACTGAAATGTGAATATGCGGAAAATCAACATTGTCCAACTTATTTCAAGCCTTGAATTGACTCTGAAAACTTTATACAAAGCTTCAGTGTAGCACTTCATCGTCTGTTTATATCAGGTTTTGATCTGTCAATGTAATGTAACCTTGATAGAATACAAACTTTCTTTTTTCTCTATTTGCTACTCGAAATGTCCAAATTATAATCTCGTTATCCC

*S.maritima*29081

TTGTAAAAACATTTCAAGAGATACACTTGCTAAATGTGTGTTTCTCTGAAAATATACAATGTATGACACAAATCTCGGGAATGAGCATAACAAGCAAAATGTTCCATTCAAACAACAGATGTAATAAAATTTGTTAACAGCTGACAATCAAAAGTAAGAAAACCTCATTAACTACACACAATTCTTCAGTCATACCTACAGACTCTATACTACTTGTACTACCTACGCTCGCTTCAAGTGCAAACCACCCTGATCTCCACAATACTCTATCAATTCTCGCCATCAGCAAATTTCACCATAGAAATATCCTACTTTACAAGTTGACAGTTTAAGAAGCTTGAGTATATATAGATGCCTTTTCCATTGCAAGGAATGTGTACAAATATAAATGAATAGTTCAGCTATGCGAGAATATTACAGCACAACTTTTTCCAGTCACAAGGTTTGTCACGGAACTGACATCAAATTCTACGACCTCTGACAGCAGATATTGGAATTGCATATACAAACATTTCTATACAAACAAACACAGAGTTCAACATCATTGGATCTCCAAATCCTTGTCAATGGATATATGAGGTTCACTATCAGAATTGCTGTCACTTTTTTTACGTTTAGATGATTTTTCACTTAGAAACCGAACTAAGCTACCTCTTGTCCTTGGAACTGATCTGGTTCCATCACCTGAATGTTCTGCAGGACTAGTCTTTGCCATATTATCCAAAATGGATTTTGTGAGTGTTTCAAATCGGCGTCTAAAAGTAGGATATCTTGAGATTGACTTTGTGATCCCTCGAAGTCTTCGAGCCAATGCCTCAAGTTCTGCTCTTTTGCCTTCATATTCAGGGGTGCTTACAATCCAACTACTTCGAAGTCTCTCCGGATCCCCACCAAGTAAAACCAATTTACCAAGATAATCTTCTAATGCAGGTGAGAGATTTTCTGCTTTGATCTGTTCCTTCATGATCAAAAGTGGGTTGAAAAACCAATCAAAAAATGTATCTTTTGGTCTGTTTGTGCTAGTTATTTCAGTTACATTATCACTTAATAGCAAACCAGCAGAATTAGACTTTGCAGATTTGATAAGCATAAGTAATAAGGAGTAAGCAGGTAAACCAATGCTAATACCACCATGGCTATCTTTGCCAGACTTTGCATCTTCTACGTCCTTCACAGATATTATCCCTTCAGAGACCAGTCTTTCACCATGGCGACGACACTCTTTAAACAAGTTATCTAGTAGCTCAATTGGCTTGAGGTCAACCATAGGACGCTTCACTGAACCGTTTTGAGAGGGCACCTTCTGGAAAGAATTAGGCCTTGAGAATGAAGGAGCACGGGATTGAGGTTGTAAAGATGCGTCCCTCTTCCGGTAAGAAGGCCTTGGAAAACAAGTTCCCTCTGGCATGTCAAGGACATCATTGCTGTACTCATCATAAATGGCAAGGGCAGCCAGAATATAGTGAAGCCCCATCACGAAAGAGGACTCCTGATAAGCAACAACCCCTGCGTAAGCACCTAGGAAGACACTGAAAACCATGGAACCCAATATTGCCCCAATAACAGCTAATGGCCAGAGAATAATAACAAGGCCAGCAAAAGGGACACATAATGTCTCAAGGAAAGGACCTTCACGTCCAATCAGGTCATGTACCAGACGCTTCCAACCTTTGAAGAGCATGTATGGGCCTTTGAACAAAGCAATAACTGAGATAACAGGTACATCAACTAAAAGACCAAGAACACCAGCCAAAATTGCTCCAGGAAGATAAAGTATTCTAATTTCATAATACTTTGCATCTGGAGGACCACGATCTCGCAGGTCATCCATTATTGAAAAGTACGAATGAAAACAAACATCTTTGAAATCTCTGACAACAGTAAAGCAACCCTGAATAGTACCCCAAGTTCCGTCATAGATAGAATGATAAAAACCGTTTTCCTTTCCTTCTCCGACAGCATCAAAAGTAGCAAATACTGGTGAAAGAAAGCCATATGCTGCACCTCCTATAATGCTGGCAATAATGCCAACCAGTGGCCAGAGAATCAAAAGAACAAAGACTAAAGGGCAAAGCACAAGCTTCAAAACCGGTCCTACTTGCTTAGCTCTTACAATACAATAATATGTCCAAAAAAAGTGTGCTGGCCAAAGACCCAAGATGACTGCACTGTTCCCCACTGTCATAATTAAGCACACCAAAGGGCAAAACAAAACTCCTTTGATGGTGCCCAGAAGAAGAAGACCAATAAAAAAAGGAAGAAAGTAAATAAACTGCCATATAGAAGCCCAAACACCAGTAGGAGGTTCCATTATTGTTTTGAAGCATAAAACAATTCAGATCTCACAAGAAAGATGAAAACCCCACATCAGTATTCAGTGCACTGTTTTATAAAATACCCAATTTCCCAAAAATGGAAGGGAAAAAAAAAGTTAAATTTGTAAATAAACTTGAAAATGTTTGTTCGACAAAATGGGAAACAACAAGAAAGTACGTGATGAATTTGATGATGTTAACGGTTGGACAACTGCAATTGTCTTGTCTTAGAGTGAAGGAAGGTTGTGGAAAAGAGCAGTTGCATTAATCTTTTTGCAGATTTAAAGCAAGGAATATAAACCCAACCTTGATTTATCAACTTTGCCACCAAAAAAAAC

*S.maritima*31247

AAAGGAGTATATATTTTTCATGATTCAACTTTACAGGCCATTTTTTCCTATAAAAGAAGCTTAAACATTTGTGTCCCTCTCTCTAACTCTCACCTTTGCTTATTAACACTGCTTACCCCATCAGTCACTTGACTAACCTTCTCTTATGGTCTCACCACACGTTCTCCCTCCCTATTGTATGGCCACAGTTCATCACGACACCAGCACTAGCCCAAAATTTTTCCACTTCAGTGCAACATTAAAAGAAATGCTAATGAAAATGTTGGACTGCATGAGTTCAATCAATGGATGAGAATAAGAAAGAGGCAGTGATAGATTGTTTTGCCATGGCTACAACCTAGAATTTTGGGTCACAGAACATGCTACCATACCCCCACACACTTAGATCAATGTGATCAACAGGGAAACCAAAGATAGTGAAGATACAATGTTGTCTTCCATGTTTGCCTCTTGCTCCATTCTTGGAACAGCTACAAAATAACGCTCCACCCTAAAACTGAATTCCTCATAAAATTAAGAGCTGATTATATGTAAGGGAGAGAGTGATTATGAATATCTTGGGTGGCTAAGGTAGTAGTTTGGGCTATGTTCAAATGTGGGAGCAGAAGGATGTAATGAACTCATATCAAGGGAAAGGGTGCTTCCGTACTTGAGGTAGTGCATAATAGTTATATGGAAACCCTTTCAACTAGACTGTGCGTTCTTTGAAGACATTTTTACATGGGAGAATTTTGCAGATATTGGGGATGTGTCTTTAAGTTATGTCATCAAGTTCTCTTTTCAGTTTTTACACAAGTCTGTATTATAGGATCAGTGGTATGTGCATTCGTAATACACCTTGAAAATTTGATCAAAACATTGTGTCTTTATCTGCAAATTTTTCAGAGGAGGAAAAGGTGAATCGTTGCTTCTCACCACAGCTTGATAGCAAAGATTTGGACATTAGTGATGGTTCCAAACCTTCTCCTGCTGTGAATATGTCATCTGATATGGCTTCAGTGTCTTGCATCACAAGCAATGGTTCATCTACTGGCGAAGGGAATTATTCATCGGCTACTAATATTCAAAGAACCTTTGAGAGGGTTTCAACTAGTGATGCATTGTGCCCATCTTGCAAGCAACTTCTATTTCGTCCTGCTGTACTCAATTGTGGCCACGTATATTGCGAAGCTTGTATAGTAATACCAGAAGATGGAATAATCATATGTAAAGTTTGTGAGTACCCACATCCAAGTGACTGTCCAAATGTTTGCTTGGCAATCAATAATTTTTTGGAGGAGAGATTCCCGAAAGAATATGCAGCACGAAGAGAAAATGATGAACGGAAACGAGAACGCTTTTATCATAAGAGCCCATCGTCATGTTCTGGCTCAAAGAAATCTGCCAAAAATATCTGCTGCATACCACCTAGGCATGGTGAAGAATTCAAACCAGGGTCAAAGGAACATTGTCGTAAAATCCATCCAGGAGCTGGTTGTGACTACTGTGGAATGTATCCTATAGTTGGGGATAGATACAGATGCCTAAATTGCAAGGAGAAAATGGGATTTGACCTTTGTGGAGCTTGCTATAATACACGGTCCAAGCGACCAGGTCGATTCAATCAGCAACATACTTCAGAGCACAGGTTTGAACTTATCCAGCCACGGGATGCGTCATCGAATATGATGTTGCGCATTGTTGGTGGATATCTTGTAAATAGTTCTGGTGAACCTGTTCTCGCAATAGGTGCAATCGAAGATCCGCACGATGGTTGGGTTGCTGTTGAATCTCTGGATGAAAATCTTGACGAGCTTGATAACAGTGAAGCAAATGATGGTTCTGTTGAGATTGAGTTTAGTGATGCTGTGGAAGATTCTGGTGATCATGATCGTGATGGTAGGGAGAATCCACCGTTTTAAACCATGATATTGCAGCTCGATGTATTGAATATGCATCTTCTCAGTTCCAAATCCTTAAAATGAGGATAAAGACGTTGATGGCTTTTTTGTTTATCACCCTGTTTGGCTATACAAGTAATTTGTTTATTTTTTAACGTTCTGTGATGTAAATATTTGGAGTAGGCAGGTTATCGGTTTAGTGTTCATGTTATATTGGAATGTTGGTATTTATCTCACAAAGGAATGTTCAATAATTATTGTTGTCGGGATTGACA

*S.maritima*29081

CGCGTATTATGACATTTTTATGGTTGAAACTTGGAACACTAGCCAAGTAGCCAAACAATATAATTCAACAAAAAAACTGTATGCACTAAAACTGACAATTGGTATTCTGAGGTCCAGAGTTTCTTTACAAACTTCAAAAGTGCCATCGATGTCAAAAACATATTGTCAAGAGCAGAAATTTATTTAGTACCTTCGAGCCAATGCCTCAAGTTCTGCTCTTTTGCCTTCATATTCAGGGGTGCTTACAATCCAACTACTTCGAAGTCTCTCCGGATCCCCACCAAGTAAAACCAATTTACCAAGATAATCTTCTAATGCAGGTGAGAGATTTTCTGCTTTGATCTGTTCCTTCATGATCAAAAGTGGGTTGAAAAACCAATCAAAAAATGTATCTTTTGGTCTGTTTGTGCTAGTTATTTCAGTTACATTATCACTTAATAGCAAACCAGCAGAATTAGACTTTGCAGATTTGATAAGCATAAGTAATAAGGAGTAAGCAGGTAAACCAATGCTAATACCACCATGGCTATCTTTGCCAGACTTTGCATCTTCTACGTCCTTCACAGATATTATCCCTTCAGAGACCAGTCTTTCACCATGGCGACGACACTCTTTAAACAAGTTATCTAGTAGCTCAATTGGCTTGAGGTCAACCATAGGACGCTTCACTGAACCGTTTTGAGAGGGCACCTTCTGGAAAGAATTAGGCCTTGAGAATGAAGGAGCACGGGATTGAGGTTGTAAAGATGCGTCCCTCTTCCGGTAAGAAGGCCTTGGAAAACAAGTTCCCTCTGGCATGTCAAGGACATCATTGCTGTACTCATCATAAATGGCAAGGGCAGCCAGAATATAGTGAAGCCCCATCACGAAAGAGGACTCCTGATAAGCAACAACCCCTGCGTAAGCACCTAGGAAGACACTGAAAACCATGGAACCCAATATTGCCCCAATAACAGCTAATGGCCAGAGAATAATAACAAGGCCAGCAAAAGGGACACATAATGTCTCAAGGAAAGGACCTTCACGTCCAATCAGGTCATGTACCAGACGCTTCCAACCTTTGAAGAGCATGTATGGGCCTTTGAACAAAGCAATAACTGAGATAACAGGTACATCAACTAAAAGACCAAGAACACCAGCCAAAATTGCTCCAGGAAGATAAAGTATTCTAATTTCATAATACTTTGCATCTGGAGGACCACGATCTCGCAGGTCATCCATTATTGAAAAGTACGAATGAAAACAAACATCTTTGAAATCTCTGACAACAGTAAAGCAACCCTGAATAGTACCCCAAGTTCCGTCATAGATAGAATGATAAAAACCGTTTTCCTTTCCTTCTCCGACAGCATCAAAAGTAGCAAATACTGGTGAAAGAAAGCCATATGCTGCACCTCCTATAATGCTGGCAATAATGCCAACCAGTGGCCAGAGAATCAAAAGAACAAAGACTAAAGGGCAAAGCACAAGCTTCAAAACCGGTCCTACTTGCTTAGCTCTTACAATACAATAATATGTCCAAAAAAAGTGTGCTGGCCAAAGACCCAAGATGACTGCACTGTTCCCCACTGTCATAATTAAGCACACCAAAGGGCAAAACAAAACTCCTTTGATGGTGCCCAGAAGAAGAAGACCAATAAAAAAAGGAAGAAAGTAAATAAACTGCCATATAGAAGCCCAAACACCAGTAGGAGGTTCCATTATTGTTTTGAAGCATAAAACAATTCAGATCTCACAAGAAAGATGAAAACCCCACATCAGTATTCAGTGCACTGTTTTATAAAATACCCAATTTCCCAAAAATGGAAGGGAAAAAAAAAGTTAAATTTGTAAATAAACTTGAAAATGTTTGTTCGACAAAATGGGAAACAACAAGAAAGTACGTGATGAATTTGATGATGTTAACGGTTGGACAACTGCAATTGTCTTGTCTTAGAGTGAAGGAAGGTTGTGGAAAAGAGCAGTTGCATTAATCTTTTTGCAGATTTAAAGCAAGGAATATAAACCCAACCTTGATTTATCAACTTTGCCACCAAAAAAAAC

*S.maritima*864747

CAAATAGTGGGTGTTATTGATGAGAGACCCAAAGATATTAAAACATTGGACATGTACAAAAATAAAAATCGGTGGAAACGAGTAGGTGGCGATAGTGACATTGAAGAAGATTCTGCGAAAATTGACGTTGATCAAGTCAACAATTATGTGCGACAAATTAATAAATCCACGTTAAATGATCTAATAAAAGAGAAAAACGAAGAGTCAATAGAAAATGGTATCCCTATTAGACGTGACCGAAAAACTGGTAAAAAACGAGATCTCAAAAGAGAAAGAGAAGAAGAAAAACAAAAAGAAGAAATATTAAAAGAGCATAAAGCAAAATATGCTATATGGGGAAAAGGTGTAAAACAAGTAAAGGATGCTCAGGAACAACACCAGTCTGATTTACATGAAATGCAAAAACCATTAGCAAGATACGCAGATGATCAAGACCTGGAGAATTTGTTGAAGTCTAAAATCAGAGACGGGGATCCAATGCTCGAATATATAACAAAAAATAAAGGATCAGGTGAAGATTCTCCTGATGGTGTGAGACATGAAAAAAGAGAGTTTCGAGGGTTTTGTCCGCCAAATAGGTTTGGCATACGTCCAGGAATTGCTTGGGATGGTGTTGATAGATCAAATGGTTATGAACAGAAATGGCTATTACAACTAAATTCTCAGAAAGCTGTGGAAGATGAAGCATACAAATG

*S.maritima*1272171

TCATTTTTAACTTAACTTTTGTGTTTTGTTTTTTGTTTGTATTTATGTCTTCCCCAAATGTCCTCTTTTTTTATGCAAACGTAGAGAAGGCAGACACCTAGAATTCCATCCTAGAATTAAGACGTGACAATAAACTTTGATTTTGTAAGTGCATTGTACGCTGACCTAATAGAAGATGAAAAGTGGAAAAATTTGCACAATGAGTTCAAGAACTCTGAGGAAAAGTATTCAAACAGTCTAGTTAAGAAAACCTTATTAAGTGGAAATTTCATACCAAACAAGGCTGCACAGCTTGGAAAAGCCCTGCACTTAAAATTAACTTCAGCCGGTGATGTGCTGAGGCTCTTGACTCAAGTACAGGCACACGGGCTGGCTATGATTTGGAGAAACATGGTAAGTCATATGAATGTTAGTTGATAACTAATTTTATTGTTCATGTGCGGAGGACATAGGGTCAAATGTGCCCCAAGAAGACCTCGAGAAAATATATGTG

*S.maritima*218708

AAAAAAAAAAAAAAAAAAAGACCTAATCTATCAGCAAATCAGCAAACAATGGATGATTACTCAGTCTTGAAAAAAAGCCAATCAGGCAATCATCTCCAGCTCTTCGCCTCTTCCAGCATTAACAATGAATCATTTTGCTTCAGACTCTGCGAGAGACCGCTCTCAATTCAATCCTACTGAGGTAGTAATTTGACAGTGAAACACTTCTAACTCAGCTATTGCAATGTTGATTGGATGCCCATGCAACATAGATAATTGGAGGATCTACATTTAAATTTCTCCAAGCAAATAACCCTGAACTGCTGCAAGCCTGCAAGTACTTTTCTCAGCTGTCATCCAATCTGATATGATCAAATGCTGCACATATCATAACCTTTTCACACCCGCCAACAGATCCGCCTTCAGCAGTGATCACTGTGAAATATGTATGTCTCGGCTTTTCTGCATGTTTCTTGTTCATCATGTTGCACATAATAATTAATCCTTTTCAACATCCATAGTAGCTCATGATTCATTCAACATAGGCTGCTACCAGATGCAATAATACTCTTCTGAACAAGAATCGACATTAACCTCATCATTCCTTCACATCGAGAGGCTCCAATGAACCCCAAGTCTTGCCAACCTTCACCTTTACTTGCAGAGGAACTAGAAGAGAAACAGCAGTTTCCATACACAGCTTCAGCAATGACCCAGCATCATTTAACACGGACGGATCAACTTCAAGAACCAATTCATCATGCACCTGCAATAGAATACGGCAGTGTCCCTTGAGCATGGAAAACTTTGTTGCAAGAGCATCATTAGAGCTATGGGTACCAATTCCAGCAATGACAGTGTAGATGTTGATCATTGCTATCTTTATTATGTCAGCTGCAGAACCCTGACAAATAGAATTCACAGCTTGTCTCTGAGCTTTAGCTTTCTCCTGCCAGTCGCCAATATGTATTTTTGATAAGAAGCGCTTTCGGCCCTTGAGAGTCTGCACGTATCCGCGCTGACGGCAAAATGCAACTGCTTCTTGAAGCCAAGAAGCAAGGCCAGGGAATGAATTTTTGAAACTTTGAATTTTCACTTTAGCTTCATCTGCACTGCAATTTAACTGCTCAGCAAGAGTATTAGCACCCATACCATAAAGAATGCCATAGACTAGCCTTTTTGTTTGGTCACGCTCTTGAGCGCTCACATAAGATTCAAGCTTTCCTGTCCATTTAGATGCTATCAAGGTGAAGGCATCGCCATGAGGATTACTGAGTAATTCAATTAGTGAAGGATCCTCAGAGAAGTGAGCCATCAACCTCAGTTCTATCTGGGAATAATCTGCTGTTAAGAGTAGCCAATTTTCCTCAGTCGGAACAAAAAAGTTTCGGGCATTGATTTTATGATCCATAACATCATGATCACAATGAGCATCACCATTTTGATGCATTCTGAATTCTACCATATGTTCAACACACTGAAGATTAGGTTCCTCCATTGAAAGTCTTCCTGTGGCTGTAGAAGTCTGGAGCCAACGACCATGCAATGTATACCTTTGTGTCCTCATAGAAAGCTTTGCTAAAGAACAAATTGATCCCAACGTACTATTCAGGAGCTTTGCTAATGTTCGATGCTCCTTAATCACTGGAACAATAGGGTGCTCAAGCCTTAACAAGTCCAAACATTGTTTGTCCGTACTTGCATGATGTTTTCCTTTGTTGTGTCCCTCAGGTATTGGAAGCTTCAAGTGTCCATAGAGCACATTTGCAATATCTGCAGGCATGTACAGTGAAAATGTCATGCCAGCAAGAGTGTAAGCTTCCTTCTCCAGGATCTTAAGCTTTTTACTTAAAACATGTCGTGCCTTGAGGCAACCTTCCATATCAACGCCTATCCCCAGATCCTCCATGTCTGCAAGTACATGAACCAAAGGAAGTTCAATGCCTAAAAGTGCTTCAACAAGTTCTTCAGAAACGAGCAATTTCCAAAGAACACAACTCAGAGCTCGTATTTGAGCAGCACGACGACAGCAACCATTATGTGCAGCTCTTCGCATCTGATTTTTCCACCTGCCACTCTGGTTAGCTGCGGCCGCAGCCTCACTGGATAAACGCTTCTTAACTTCCTTCTCGAGAATTGGGTTAGAGCTCCTCTCCTCGTCAGGCCACAGGATCCAAGCTACGATACAGATGTCAATAGTATCTTGCAGAATAACCTGGGGCAGCCGACAAAATGAGCCATCAAGAAATTCAATGTCTGTACTTTTAATAGTCTGACTCAGGCTGTTTTGGTTCAGTGACAGAATGGCATTTTTCAAGACCTGAACTTGAATTTTTAAGTTCCAAGTAAATTTCCGCACACCAGTCCTCCCAAGTAACCTACCAATCCTATTCCACCGAATATTTTCCATGACCAACAGAATGTTAGCATCAACTGACTTTACTTGCCCAGACAATTCATTGCTGCATGAATCAAAATTGCTGTTCTGCAAAATCTTTACAAGATTGATATAATACACAGGTGAATCTTCCCAGCAAATGGCAAGGCCATGTACTTCATAAGAAAACATTGAATCCACTTCAGACTTTTTGTTGAAGTAAAGATCAAAACAAAAATCTCCAACTGACTCCCAGAGATCCAAGAGAGCATCCAAACTACTTGCGGTAGCAATATTAACAGGACCCTTGTCCCTACATTGTTGCTTAGTACCTCGACTGCAGTCATTGCCTCTCTCCTGTTGTTGGTCTTCCTTATTTACAGCTCCAACACACATCACGGTTTCATTAGGTGTCTTGCTTGCAACTGAAACACAATCTTTAACTGTTGTAGAGGAAGCTCGAATTTCACAAGCACCAGGGACGGGATTAACTGTCTTTCCTTCAGCAACAGTTTCTATGCCAGTGACTAAAGACTTGTCTGCCCTTCCAGCATCAGCATATGGAGCACCCTCATCCCCCTGTCTTTCATGAGGATAAAAGCTGCTGTTACTATTTTCTCCAGAAGAGGAGGTCAAGGCTTCATTGCCATCAAGGACATTAGTGGCAGGTAATAAGACAGGATGAGAAAGTTGTGGAACATTCACTCCAAGCGATTGGAATGCCGAGAATGCAGCAACTCTCGCCTCCTCAGCTTTTACAAGGACAATTTTCCTTGCAGCATTTTTAATCTTCTTTGCTATTCCCAACTGAAC

*S.maritima*41484

TCATCATCATCATCACCATCATAAAAAAAGTGGAAAGTTGAAAAAAGGGGGAAAAAATCCATCTCCACAACCTGGGATTTCCTACATTCCTGATTCACAATTTGGGCCTAATAATAGTAATTATCCTTTTCCTTTTGATTCTTATGCTACTAATTCTTATACTAATGAAGTTCCTAATCCTTATTATTATTCTGCTAATCCTAATTTTAATTCTTATTATCCGGAAAATGGTTACAGTGATTATAATAATAATAACTACAATAATTATAATTATAATTACAATGGTAGCAATTTGTATGGTTCAACGACTTCTTATACAACTTACTATATGAAGAAGTCTTCTACCCGAATTCCATCCGTTATTTACGAGGAACCTAAACAAACTCCTGTTTATACATTTTCTGATCACCCTGCTCAGTCAAGTTATCCGAATAACCCGAGTTTTGGGTTTTCAATGACTCCGGATTATAGGGGTAGTAATGTGCAGCAGCAGCAACCACCGCAGCAGCCAAGGCGGCCGCCAACGCCGCCTTCGCCAAAGGTGTCTGCTTGGGATTATTTTAACCCATTTGAGACATTGGAGGGTGGGGGTTATCCGGGGTTTATGGTGCAGAATAGTTATGCAATTGGGTCGAATACGAGTAGTCCAGATTCGAGGGAGGTTAGGGAGAGGGAAGGGATTCCGGAGTTGGAGGAGGAGACTGAGAGTGAAGTTAGTAAGGAGCCTTATTATTATGATAAGAAGAAAGTTACAGGAGAATTTAGCTTTCCCAAAACTAAGAACAAAACTGTTCAATTTGAAGATGGGATTGGGAGGAACAAGAAGAGTGGTGGTAATTTTGATGAGGGTACTTCGAAAGGCGTGAGGTCGAGGGTTCCTTTGCAGCAGTCATATAGGGAGGAGGTGCGAAACTCAAAGCCAGCAGTGGAAGTGCCACACCCCAAGTCAGCGGTGGAGGTGCCACACCCTAAGTCAGCAGTGGAGGTGCCGGAGAGGGAGGTGGAGACTGTCGAGGTGAGAGATGTTACAGGAAGAAGCAGTAGCAATAGTAGTCCTGAGACTGAGATTGAGACTAGCATGTCTAGTTTAGAGGATTTGTCTGTTAAGAAGAAAGGGGTTAGCTTTGATATGGATGGTAGTGGGCATGGAGGGGGAGGGGGAGTTTCATCTCGTGATATTGGATCATCTAAGCCAAGTAGCTCGACGACATTGTCAGCTCACGGCACTCGTGATCTTAAAGAGGTTGTAAGGGAAATCAAGGATGAATTTGAGGCTGCTACTGATTATGGGAAAGAGGTCTCTGTGTTGCTCGAAGTGGAGAAGGAGCCATACAAGTCCAGGTCCACGTTTTTGAAAGTGATTCTTGCTAGGATGTGCTCTACATCACTCTCATCTTCATTTTCTCCATCTTCCCCACTTGGAGGACATCCTGGTAATGGTGACAGTAATTTTGACTCTCGGAGCCTTGCCTCAACTTTGGAGAAGCTATATGTATGGGAGAAGAAACTATACAAGGAAGTCAAGGGCGTTGTGCTGGTTTTAGGATGAGGAGAAGCTTAGGGTCATGTATGAGAAGCAGTGCAAGAAGTTAAAAACTCTGGATGATAATGGAGCTGAGACAAGCAAGATTGAAGCCACTCAGGTTTCAATTAGGAAGCTGCTGACCAGGCTTAATGTTAGTGTCAAAGCTGTAGATTCTGTATCTAGAAGGATACATGAATTAAGGGATAAAGAATTGCAGCCTCGAATGAAGGAATTGATTTATGGATTGAGAAAGATGTGGAAGCTGATGCTTAAATGTCACCAGAAGCAATTCCATGCAATCTTAGAAAGTAAAACCCGTGCTCTGAAAGCAAACACTGGACTGAGACGAGATTCCAGTGTTACAGCTACAATTCAGCTTGAAGCGGAACTGCGAAAATGGTGTCACCGTTTTAACAACTGGGTTGAAATGCAACAGTCCTATGCTGAGACTTTAAATGGGTGGCTTGAAAAGTGCATTCATTATGAACCCGAAGTGACACCAGATGGTGTCATGCCTTTTTCGCCTGCCCGTATCGGTGCTCCACCGGTGTTTGTAATATGCCATGATTGGAAACAGGCAATGGAAAGAGTATCCGGGTCGGAGGTAAAAACGGCAATGAATGACTTTGCTACAAGCTTGCATCAGTTGTGGGAGAGGCAAGATGAAGAGCAACGCCGAAGGGTGAAAGCCGAGAACATGTACAAGGATTTTGAGAAACAAATCCGGGCAATGAGAATGGACCGGCAAAAGAAGGGGCATGAGCATGATGATTCCTTGTCAGACAAAAATAGTCTCTCAATGGTCACTTCAGAAAGTGGGATTTCACCCCTTGATGATCTAAAAGTGGATTTGGACTCTATGAAACAGAGGGTAAGGGAAGAGAGAGCAGGCCACAAGGAAGCTGTCAAACTCGTCCACGATGCAGTATCACGCTCCATACAAGCTGGTTTGATTCCAATATTTGAGTCGTTGGAGAGTTTTACCTCGGAAGCATCTAAAGCTTTTGATGATGTCAGGATAGAACATGGCAGTAGAAGTTGACAAGGAATTGTGTACCAATAGAGAGAACATAGGGTAATTTAGGTTAGAAAGTTTGAGAAAAGAGAAATGTAGATGTTAGGCGTCGAAAGAAAGCAATAGTACAGCAATTGGTTAGTGCTAGTTTGCTAGGGATTTCTGAGAAGTTGCAGGAGTTCATTGTGAGCTTCGTTTCTGGTTCTGCAATAGCATAGAGGTTAGCATCTCTTGTACAGAATTTTGTTTATTAATTTCATTCGTTCAGATCGAAAGTACTAGTGTCGAAACGTGAAATATATGTAACATTGTATTGGTAATGAGTTGAAATTAGAGTTAAACATCAGCATTCAGAGTTTTTGACTCTGCCATCTTTGAACTGTATTATTT

*S.maritima*8922

TTGAGATATCCCTGACGCTGGCTATATTCATAATTTCATACCAGTAAAATTTCCTACATTACAACATTACTTCTTTCTTTCGAATCCACTCTTGCGTTGCTCCCTCTCTCTTCACACTCTCACTCCTCCATTAAAGCGTATTCTGAGTTCTCTATTGAAGCGGTTTCTGCACTTCCTTTCCTCCATTGAAGCAACTTTTGAGATATTTGTTCATAAACCTCCAATGGTAGGACGTTCTCTCCCTAGGCTCAGGGCATGCGTCTCTCCCGCAGTGTCGCCGGGGAGGACCTGTCAAAGTCTCGAACAAATCGACCCGATTCATTTTGATGCGGAGTCGGGTTCCGTTGCTCTTTTAAATGCTTCTCAGCTCCCATCACCTAAGCCTGCTTTGCAGCTTGTTAAGCGCATTATCAGCAGCAGGTTAGTCACTAGCTCTGACTTCTTCCGCAAATTGAATCAAACCCGTGTAAAGTTGAAGTTCGATAGTAGCGTTGCATTTGATTATTGGATTCAAGGTTTGTGTCAGATGAAGAAAGCTGAGGAAGCTTTCAAGTGTTTTAAGTTGATGAATAAGTTGGGTTTTTTGCCTAAGGTTGAAACTTGTAATCATTTGTTGAGCTTGTTTCTTAAAATGAATTCTACTCACTTGGCTTGGGTTTTGTATGCGGAGATGTTTAGGCTCAAAATTAGTTCTAATACTCATACCTTTAATATTATGATCAATGTGCTTTGCAAGGAAGGCAAATTTAAGAAGGTGATGGAATTTCTAGATCTTATGGAGATTAGTGGTGTTAAGCCTAATGTTGTTACATATAATACGATTATCAATGGGTATTGTTCGAGGGGTAAAATTGAAAGTGCTAGGAATATATTGAGTGAGATGAAAGTTAGAGGGGTTTATCCGGATTCATACACATATGGTTCGATTATTAATGGGTTATGCAAGGTAGGGAGACTTGATGAAGCTGTGGAGTTCTTTGATAGAATGGTGGAGGCTGGGTTGGCACCTTCTGCTGTGGCTTACAATTGCTTGATTGATGGTTACTGCAATAAAGGGGATCTAGAGAAGGCGTTCTATTATAGGGATGAGATGGTTAGAAAGGGTATATCACCTACTTTGCCAACTTATAATATGCTGATACATTCATTGTTTATGGAAGGTAAGGATACTACTGCTGATAGCTTGATAAAGGAGATAGAAGAGAAGGGTATGAAGAAGGATATTATTACTTACAACATTCTGATCAATGGGTATTGCCAGAGTGGAAATGTAAAGAAGGCTTTTGATCAATATGATGAGATGTTAGTTGAAAGGATTCAGCCTACAAGGATTACTTACACATCCCTTATCTATGTCTTGAGCAAATCAAAGAGAATGAAGGAGGCCGCTGATCTGTTTGAAAAGGTAACCCGAAATGGTTTGACTCCAGATCTTGTTATGTTCAATGCTCTGATTAATGGTTATTGTGCCAATGGGAATATTGAGAAAGCCTTTTCAACCATGAGGGAGATGGACAGAAGAAAAGTTTGTCCTGATGAAATCACTTACAATACTTTAATGCAGGGTCTTTGTCGGGATGGTAAGGTTGAAGAAGCTCGAAAACTTATGGATGAAATGAAGGAAAGGGGGATTTCACCAGATTTTGTTAGTTATAACACACTTATAAGTGGGTATAGCAAAAGAGGCGATGTGAAAGATGCTTTTAAGGTTCGAGATGAAATGTTTGATATAGGTTTTAATCCGACTCTTCTTACTTATAATGCCTTAATTCAGGGTTTGTGTAAGAATAAGGAAGGTCCCCTTGCTGAGGAGCTCCTGAAAGAAATGGTTGATCGTGGGATTACTCCTAATGACAACACTTATATCTCTTTGGTCGAGACTATTGGTGATGTAGAAACTACCAGTGAAAACAGCCCGCCATAGAAAGCAGTTATATAGAGTTCTTAGCTTTGCAACAGTTCTTGGAGGACAAGGATGGAGCAGGGGAAACAAGGTATCTGAAAACTTTGGAAAGGCAGCTGGTATTCCTTTTACAGAGGAGTTCTTGTACCAGATATATCCAACAAAACTCAATGTTTGAGAACTTAAGAAGTTGAAACTTGGAAGAATAAGCTACTTGATATAAAGTTGAAATTTGTTGTTTGTGCTCAGAAGAAGCAAGGTTATCTATCATATTCTTTATTTGTCACCACCTTAAATCTCAGCTGTTCTAATCTGCTCTTGCTGCTGTTGCAATTGCAACCTCAATTCCTCTAGGGTTGATGGGAGAGTGGATTGATTCCCTTTGGAGAACTATGAGATGCATTAAAGAGGCAAAAGGTCTAAGCTAGCAAATGAAGAGAAAAAGATGGAGGGGGTTGTTTCAACAGGATATAACGAAGATTAGGGGTAGGGAATAGGGATATCAGGAGAGCTAGGACTTGTCCTGCAGAGGGTCATCAAGCATTCCAATAAGGCAAACAAGTGAACACTGAACAGAAAGACTTCCCCCAAGTAAGGTCGTTGAAGATTTGCTCGCTGCATGGAATGGTAAGGCCACCCATTGGATGATTGTACCCGAATTCCTCTTCAGCTCGACGAAGCAATGCTTGGAAAGATGCATTGCTCAGGAATGACAAGGGTATTACATGTCTCTTCTTTTCTTCATCTCCTACGTAAACAGCAATGTGACCTTTTGGTACTGATTTTTGGTTGTCTTTGCTAAAAGACGATTGTCGATGTAGGATTTGCTTTGTGTTTGAGATCACTGAATACAATCTAAGATAACCCATAATATTTGTTTTCTTTATGTCTTGCTTTCTTTGAAGGTAATACTTAGTTTGAAAAATG

*S.maritima*45073

ATTTGACAGAAAAATCTAAAGAATCTCACCCCAAAAACCCCGTTTGAGTTTTGACCAAGCAAAAGATTCCAATTTTCCAAGTTGTATAATTCCAAAACCCCAACTTTCCACTCTCACTCTCTCGCCTCACTCGCTCACTCACCCACCATCATCAATGGCGATTGATAGACCAAAAGCAGAAAGACCAACATTAAAAGTCTCAAACATCCCACTAACAGCCACAGCACAAGACCTCTTAGACTTTCTTGAATCAAACATTGGTAAAGATTCAATCTTTGCCATTGAAATCTTCACTGAGAAGAAGAACTGGAAATCCAGAGGTCATGGAAGAGTACAGTTCGAAACCCTTGATGCAAAATCAAAAGCAATTCAATTTTTTTCTCAAAGTAGTCTCCTTTTTCAAGGGAATTTGCTTTCGTTTTCACCTTCTCATGAAGATGTTGTTGTTAGACCAATTGACCCTACTCAAAGGTCCAGTTGTGGGGATTTGTATGTGGGGTTGATGGAAAGCGAGGATAAAATGTTTGTTGTTGATACTTTTGCTGATGTTAAGTTCTGGGTTATGCCTGATAGAAATTATATTGAATTTCATGTTGAAAAAGATGGGATTTTTTATAAAATGGAGGTTCAATTGGATGATATTTTTGAGGCTTTTGGATGTTATTTTGATCCTGATGCTCCTGCTATTGTTCTCAAGCTCAAGTATGCACCAAAAATATACCAGAAGTTCACAGGGGCCAACTTACAGACAAGGTTCAGTGCTGACCGCTATCACATTTGCAAAGAAGATCTTGAGTTTCTATGGGTTCGTACAACTGATATTTCAGATAATAGTGCTATTGGACTCTCTTCTGCAATCTGTTGGGAAGTTGAAAATGGGGCATTGGGTCTAGATTTTTTTTCCACCATTCCTTTCTATAGGAAAACTTTGGACAGCTTGATATTGGAGGAATACGATGACGATTCTCCTATGCAACCTCTTATTCCTCTAGTAAACCGTGAGAACATTTCAAAATTAGATTATGAAGTCCTTTTCCAAATCAACTCTCTAGTCCACACCCTTAAAATTAGTCTTGCTTCTGTAACGACGGAGCTGATTGATTATCTCAGTTCTTTGAGCTTAGACACTGCTCTTATGATTCTTCAAAAGCTGCATAAGTGGAGCACCACGTGTTATGAGCCGCTTTCATTTCTTAAGACTGAGGTACAAGCTCTAGAAAGTGCATGGGATAATCGTTTATCAACAAAAAGTCGCTCGACTAATGATAAGATAATGAAATGTCATAGGGCTCTAATCACCCCTACAAAAATATGTCTCTTGGGTCCTGAACTGGAGGCCTCAAACTATGTTGTCAAGAACTTTGCTGCATACGCCTCAGATTTCATGAGAGTTACTTTTGTAGACGAAGATTGGGGTAAGTTACACCCAATGGTTGTATCAGCTAGTACTGAGCACGGTTTTCTCTCAAAGCCTTTCAGAACAAATATATGTCACCGCATATTGTCTGTTCTTCGAGAAGGGATTGTGATTGGGAATAAAAGATTTGAATTTCTTGCCTTTTCAGCTAGTCAACTCAGGTCAAATTCTGTATGGATGTTTGCTTCCAATGATAAAGTGAAGGCTGGTGATATACGTGAATGGATGGGTAGCTTTAATAAGATCCGTAGTGTTTCAAAATGTGCAGCCAGGATGGGGCAATTGTTCAGTGCTTCTTGGCAGACATTGGTTGTTCCTGTACAGGATGTGGAAGTTATTCCTGATATTGAAATGAATTCTGATGGAATTGAATACTGCTTCTCTGATGGTATTGGAAAGATCTCCCTCGCTTTTGCTAGACAAGTTGCTCAAAAGTGCGGGGAAGAGCAGACTCCCTCTGCGTTTCAGATTCGTTATGGTGGTTATAAAGGTGTGGTAGCTGTAGACAGGAACTCCTTCCGAAAGCTGTCTCTGCGTAGTAGCATGCTTAAGTTTGAATCAAAAAATAGAATGCTTAACGTCACCAAGTGTTGCAAGACCCAACCATGTTACTTAAATAGGGAAATTGTGACTCTTTTGACCACCTTGGGAGTTGAGGACAATAGCTTTGAAGCATTGCTGCACGTACACTTGCTTCTTCTTGGGAGTATGCTGACAAGTAGGGATGCAGCTTTGGCTGTGCTTGAGGGCACAGATATGGGTGAAAGTAAAACTCTTGCTAAAATGCTACGTCATGGTTATGAACCAAATACAGAACCTTACCTCTTGATGATGCTTCGAGCATTCTTGGAAAGCCAATTGTCTGATTTGAGAGGAAGATGTCGCGTTTTTGTTCCAAAGGGCCGTGTTTTACTTGGATGCTTAGACGAAACAGGGATATTAGACTATGGCCAGGTGTTTGTCCGTTTAACCCTGAAGAATACTGAACTTCAAAATGTAGATCCAAGTCTCTTCCACAAAGTGGACGAGAAAACTGCAATTATTACTGGGAAATTGGTTGTCACCAAGAATCCTTGCTTGCACCCTGGTGATGTCAGAGTACTTGAGGCTGTTTATGAGGTGGCTTTAGAGGAAAAGGGACTAACTGACTGTTTAATCTTCCCGCAGAAAGGAGAAAGGCCTCATCCAAATGAGTGCTCAGGTGGCGATTTGGATGGTGATTTATATTTTGTAAGCTGGGACGAGAAACTGATACCTCCAAAAACTGTAGCCCCCATGGATTATACAGCAAGCAGGCCTCGCAACATGGACCATGACGTAACTATGGAGGAGATCCAACGCTTCTTTGTAGATTATATGATCAACGATACGCTGGGAACTATCTCAACTGCACACTTGATCCTTGCAGACCGTGAGCCTGAAAAGGCCCTTAGTCCTAAATGTCTCCGTTTGGCTAGCCTGCATTCCATGGCAGTCGACTTTGCAAAGTCTGGAGCCCCAGCTGAGATGCCTAGAGTTTTGAAACCAAGGGAGTATCCTGATTTTATGGAGAGGTGGGACAAACCCTCTTACAAGTCTCAAGGGCCTCTTGGCAAACTTTACCGTATCGTTGTCGCTACAACCATCCCAAAATCAGATTTCAGTTGGTCAGAAGAAACTGCTCGAGCTGCTTATGATCCTGACCTAGAAGTAGCTGACTTTGAGGCCTTCATTGATGCTGCAATAAAGTGCAAGGAGATGTACATAGATAAAATGACTGCTTTGTTGATGTTCTATGGAGCAACTAGTGAGGATGAGATATTAACGGGCAACTTAAGGAGCAAATCTGCATTCTTACAGCGTGATAACAGGAGGTATAGAGAGCTGACAGACAGAATACTTGTCTCTGTAAAAAGTTTGCAGAAAGAAGCAAAAGGATGGTTCGAGAATAGCTGTGGGGTAGAGGACCGTCGAACCATGGCTTCAGCATGGTACCATGTTACTTATCACCCATCTTATTGCCATGACAGTAAGAATTGCCTGGGCTTTCCATGGACTGTCAGTGATGTTCTGCTTTCGATAAAAGCTGCAAACCGGAAACAATCCTGAAACCCTTCTTCAGTCCCTTTTATTTTCTAAGTTGTATCATAAAACCATGAATTCCTATCTGTAGTTATCAGTGCGGCAGTGCCACATTGTTTAATTATTTCAAGTATCTGATTAAGAGTATGTTTGGATAGATAAGTTGGAGGGAAAAGAAGGGGAAGGAAAATAGAGGAAGGGAAGGGAAGAGAAAGTGAAGGTCTATTTTTCCTTCCAAATCTCTCCAACTTTGGGAGGATAGTGTTTATTAAAGGAGGAGGGAAAATAGTTCCCTCCATCTCCTTCCCCTTCCTTCCTCCAACTTATTGTCCAAATAAGAGATCTTGTCCCCCTCCCTTTCCCTTCCCTCACTTTCTCTCCATTTCTTTCTATTCAAACAATGTGTAAAGTCTGAGTGCGTATGTATGCACTTCACCATTCATATTGCATAAAACGTTTATCAATGAACAAGGTGCAAGAAAAACTGCATGTGAACAAGCAAACAGGTTGAAATAGCATTCTACATTTGGCTTTCAAAAGCACTGTTACTCCAAGTCTCCAAATCCCATCAAACTGCAACGTTGCGACTGTTGTGAGTGAACCCTGGGATTCAATTCAGTAAGCAGAAGTCGACTATAAGTTACTCCATCTAAAGTTGCAGTAGAATTTAGCAAATGATAATCCTGAATCCAATAACAATAAACCAGGATTCCTATGATACAAGTTAAAGTGAATCAAAAGGGATAAAAGGGGCAGATAAATTTATCATAGACAAGAGTCGTTGAACGAACAAAGCAGTTTGAAGAATAGCCATATTGACCCGCCTTAGAGTCACCAATAAGTTCTCTTTTCGAGGGACAATACCTCCCATGTGGACAAATTTTACACATATAGACTAGAAACAAACATAAAAGCTTTGCAACAAAATATCGGAAGAAATAGCACAATCCCACATTAGACTAACCTCATAGGAATCCACCATCACCCCATTTGGCATCGGATTTCCCATTGATAGCTGAACTCTTTTTAGTTGAGCTTTGGTAGAAATCATCAAATCCATCATCTTTTGGATCATCCCAACCAGCCCAACCATCATTATTACTATGGGACGATCCTTCCTTTCTGGAACCTTCCTTCCAGTCATCCTTTTGGTCCCAATCATCCCAAGATGCTGAGCTATTGTTTTCCCTCCCAGAAGATTGCCCAGCACCAGAAGCATTGTTCCATCCCTGTGATTCATGCCTAAAGTTCTGATAGTATCCATTCTTTTCATTTTCAGTCTGATGTGGGTTGTCAGTGTTCCAGTTCACTCCGGTCTCCTTCGTATATTCCTCAACCTTCTGAGACGCAATAGCCATAACACCTCTCATGATGCCCCAAGTCCTCTGACCGATCTCTGATGTTTTTGCAGTAACGACATTAACTGTTTCATTGACCTTGTGATCATAGCCACCTTCACGAACCTTTGAAGAAATTTCCTTTGTTCCTGCTTGGACAACACTAGCAGCTGATTGAGCAACCAAAGACAACTTCCCAATTCCCTGAGAAACAACTGAAAAAACATCACCTTGAGCATTAGCATTATTACTAGACATAGAATTAGGATTAGGACTAGATCCAAACCCAACATACTTCCCTCCTTGAGAAGGCGGTAACCCCTCGGGCCTGGACTCGTTCTCCGCCTGCTTCCTAGCAAAAAAACTATCCTTATTCGCCGCAGACGCCTCCAATTGTGATCTTGTATACATATCATCAGACGATTTCGACTTGTGCGGTCCACTCCCATTCCCATACCCATTATGCCTAAAATCCCCAGCTGATTGATTCCTCCTCATATCATTGTTATTTGCCGACGACCGATACGAATCATCATTATCCCAATTATCCCACCCCACACTCGCGGCCGACTTTACACTACCATTACCCCTCCCACTACCACCACCCGCCATCAATGGCGGCTTTCGATTCCCACCCCCACTCCCTCCATTGACGGATTCCTTAACAACAGGCGGGTCCCGCCAAGAGCGCCCCTCGGCAATCGCGGAAATCTTATCCCGGTACACCGAGGCGGCGTTAGAATTGTATTTAGCGACGATGTCGGTCTCCTTAGGGATACCGTACTGAGCGAAGAAGGCGTTAAGCTTGTCGTTGCCACCAGACTCCATCTTTTTAAGCTGGATCTCAGACCAAGAATCCATGGTTACAGATCTAACGAAACTGATATGAACACCGAGGCCTCTGTGCTTTCCAGAACATTCTAAACACATGAAAACACCGTAGGAAACGGACGCCCATTGTGGGTTCTTTTGGGAACAATCTACGCATGTTTTGTTGCCTGTTTGAGATTGTAACTCTCTCAATCTCCTCGATGCACCTGCCATTGATGACGATGGTGATAATGATGAATTTTTTTGGGTTTTTTTTTTTTTTGTTTGGTAGATTGTATGTATGTTCCTCCTTCTTCCTGGATTTGGGGATGCGAATTAGGGGGGTGAGGAAGAAGGAGGAAGAGAGAAGAAGGAGAGAGATCTTTGGGAGGAGAGATAGAGAGATCACCGACACCAATCGATCTAAATACGAAAGAAGGAAGGAAAGAGAAAGAGGAAGGAGGAGAGAGAAAGGAGGAATTGAGAATTGAAGGAAGGAGGAATTGTCGTTGGTGAAGTGGGAATTGCGCTGTTT

*S.maritima*15309

TCATCCCCCCAGAAAAAACCCAAAAAAAATTGCTCACTCACTCACTTATCTCCCCTTTCTCTCTCTTCTCAACTTCAACTTGTGTTTGTCCACTGATTTCTCTCAGATCTAGGGTTTCGTTCTGAAACCCTAGAATTTCCCCCCTTTCAGTTTTTCCATATTTACTTTTAACTTCCCTCTTTCTTCGCTCGTCAGCAATGGCGACTCCATTCATCACTGCTCCTCAGGTGGGTTCATACTTTGTAACTCAGTATTATTCGATGCTTCAACAAAGACCAGAGTTTGTTCATCAGCTTTACTCTGATTCTAGCACTATGCTTCGTATTGATGGCAACACTCGTGAGGCTGCCACTGCAATGCTGCAAATTCATGCGCTTGTTATGTCGATGCGTTTTACGGCGATTGAGATAAAAACGGCACATTCTCTTGAATCTTGGAATGGTGGAGTAGTTGTGATGGTCACAGGGTCTCTTCAGATCAGGGATTTTAGTGGGAAAAAGAAGTTTGCTCAGACTTTTTTTTTGGCTCCTCAGGAGAAGGGTTTTTTTGTTCTCAATGATATTTTTCACATCATTGAGGAGGATCAAGTTCATCCATATCCAGCTTCACTTATAGGGCAAACCAATACTAATGTAAAGTTGAATGCTCCTTCATCCATACCAGAGGCAGTGCCAAACTACATGATGAGTGGAGCAATGCAGGCCAGGGAATACATGCCTCCTGCTGACGTCAAAGAAAACGGTACTGTTGACAAGTTTTCAATGCCTGAGCAAAGATTGCAGCAAGCTCCTGTAGTTGAAAGTATCCTGGAAGATAATTCTCGAGAACAGACAAATGGTTTTCTTGAGAGCCCGATGAACCCTATACAAGAAATCCCACCAGCACAGATAGAGGAGCCTGTTGAGGAGCCACACAAGCACACCTATGCTTCTGTTTTGCGTGCTGCCAAAGCACAGTCTGCATCATCTGTTGCGCCTTCTACTTCGTCTAACAAGGCTGTGGCTCCTGCTTCAGAATGGCAGCATTCTCCACAACAGAATGATCAGCAATCTGCTGCCCAACAATCCCAACAACAGTCTAATTCGGCACAGGTTGCAAATGAAAGCTGGGCACCAGATGTGGGTGATGACGTTTCAGCTGCTGATGATAGAGGTGAAATAAAGTCTGTCTACGTGCGAAACTTGTCTCCAACTATATCAGCTTCTGAAATAGAAGAGGAGTTCGTGAAATTTGGTCAACTTACTGCTGAAGGGGTAGCCATCCGAAGACTGAAGGACACTGATGTTTGTTATGCATTCGTTGAATTCGAAGATATTGCTTCTGTCCAGAGTGCAATTAAGGCTGGTTCAGTACAAATAGCTGGACGTCAAATATACATAGAAGAGAGAAGAGCAAACAGCAGCTTTGCACGTGGAAGGAGAGGGAGAGGCAGGGCTTCATATCAACCTGATATTTCGAGGGGAAGGTTTGGTGGTCGTTCTTATAGCAGGGGTGGTGGTCAAGTTGGAGGTGAGTTCAACAGACCGAGAGGAAATGGCTATTATAGGCAAACAAGCCGCCAAGACAAAGGGTATTCAGGGAACCAAGTATGATGAAATGGATAAACCATACCAGAGTGAAAATTAGGGTGACGGTGAAAAGAAATAGTGAAAATGTTAGTAATTCAGTTTAGTTTTGGCAAGTTAAAAACATAAATATATATATTCAATATTTTTGCAGTTGTAGCATTTGAAGAAATGCATTCTTTGTTAGTGGCTTCTGTGCATTTTTTTTGGTCGGTTTTCGCCATTCTGCTCACTTTTTTTTGTCAAGAGCAAAAATGCAAAATTGTAACTTGAATGCCATCTTCTGTCAAAGCATTCATTTTCATCAACCTTTGTTGAACTTATTGCATAAAGGTGTGCTCTGATGACATTGTAGTACCTAAACTGAATTGTGATGGGACCAAACATGAACTGTATTGGAC

*S.maritima*26853

AGGCTGATGAAAATTGCAATATTTTCGAAAATTTAATCCGAATCCAACCAAACCTTTTGGATCAGTTAATAAAATACAATACATATTCTATAAATCATGACCAAATACTGAGTAACTTACTGTATAATCTTATAAAATCTAATTTTGGGACGGAGGAAGTACTACGGAGGGGTGACTAGCTAACATCACCTCCCCGCGAACATAATCAACATCCACAAAATCTACAGTAATGGCACTCAGAAAATGCAGAATTTGCACCTAATAAATAGATTAGATTGTCTTAACATGCTAATAATAATAAAACAGATTGTTTACATCCTTAATGGCTCAATGCCAAAATACACCCGAATAGTCGATTGTTTGGAATGAGGCACATCTTGCTCATATCTGGAATTAGTCGAAGTTTAGTACGATGTGTAGTATAGTACGAGGGGGTACCTTAAGAAAAATGCCAACATCAATGATCATCCTCCAAAGTTTCTTCAACATTATTTATTTCACAACTTATGGAGGGGTGGCGATTTTATTAGTGATTACTCATTGATCACCTTCAAAGGATTCTCATAAGAAGCCTTTTCATTCTTCATAATACAATGGAAAGATTTCGCTTGATCCTCCTCAACGATCTCTCTCAACAAAGCGTCTTCAATCTTCTCTGCTTGCCATTTCGATGGATCATCCACCTGCTCTTCACTGAATATCATACAGGAAACCCATTCTTTCCAAGTTGTTCTACCATGCTTGTCTTCCTCCAATGTACCGTTAACTAAGAGTTGATAGACATACACGATCTTCTCTTGACCAGGCCTAAAAGCTCGAGCAATGGCCTGCTTTTGCTTCGAGGGATTCCATTCGGAGTCCAGTAACACCACTCTAGAGGCAGCTGTCAAAGTAATACCTTCAGCGCAAGCATTGATTGATGCAAGTAATACCTTTGATGGCCCTCCACGTTCCTCAAACTTATCCATAACTCTTCCTCTCTCAAAGAGCTCAAGATCCCCTTGAAGGACGAGAACTTCATGACCCTTCCTCCATCCGTAAATCCTCTCAAAAAGCTCGAGGAAGAGGTTTATCGGAGCTATATTGTGGCAAAAGATCAAAACTTTCTCTCTTCTTATTATAGACCGTTGAACTAGTCCGAGGACAAACTTCACCTTTGATCCTTTTGTGGGGTCATGCCTAAACTGATCAAGGCTCTGAAGCTCCTCTGCACTAAGGAACTTGTTTGCACAAACTGCGGATTTAAGCAACCATGGATGAATCGACCCTAGGGTGATCATGAGCTCGAGCTCCAAGGGAAATCCTCGAGCCACATTCATGTGTTGGTGAAGTCTAACAAGGATTTCATGTTGCAATGGAGTTGGCTTCATCATAAGAGTATAACTCTGAAGGCCAGGCAGATTCTCAGAGCTTCCGCCTTCATACACATCAATAAATTTATTTGTCATGTTCTTCAGCATGTTAAGACCATCAGCCCTCTCATCCAAGTCAACCGAGTTTATCTTCTTTGCAATGATATCCATAAATACCTTCCTTGCTCGGTTCTCTATGGAAGTTCGAGTTTTATTCTGCCCCTTGATTTTCTTCTTGTACTTTGGATCTAACACCCGAAGCACTTCATTCACAAACTTTGGCCTTGCCAAACATAGGGTGTTGAAATACTCTCCAAAGTTGTTCTGAAACAAAGTTCCCGACAGCAAAATCCTGAATTCAGTGTCAACTCTCATCAGTGCTTTTCTCAGCCTCGACTTTGTGCTTCTTGGATTGTGCCCTTCATCAAGTATCAAGATCCCGGGACTTTCCCTCAGAATTTGACCCATGTACCTACGATGAGCATACTTTGAGTCTTCTCGCATCAGTGATAAAAACGAAGTATAACCCATGAGAAGAACACTAGGGTGAGCATGCCACTTCTGTATTTTCTCCAAGCAATCAAGAACATGCATGATATCTCCATTCGGAACCACAGTTGATGAAGCAGTTCCGATTTTCTGCATGTAGATTCTATCCCTGTATGTTCTTCTTCCATGGATTTGATACACTGGAACTGGGAATTCCCACTTTATGATTTCTTTGTACCAAGTGTAGAGTGTTGTTTTCGGTGCAAGAACTAAAGGCCTCTTTCCAGGGAATAGCTTCAAGTAACTCACAAGAAATGCAATGATGAGAAATGTTTTTCCAGCACCAGGAGTATGCGAAATTGCACAGCCACCCACTTTCTTGCCTGATGGTTCCATTTCTGAAGGTATCAAAGATCCTGCAAGATTCCTCCAAAGGAATTCGAACGCTTTCTTTTGATGTTCATGCAACTTCTTCCGTATGTTTGGGATGAGACCCCAGACATTGTCGTTTGATTCAGACACGATAGTGTCGTAGGCAGAAGGATTGTAAATAAGATTGGCACATGCCTCATCAAGCAGCTTATGCTCTGTATCATCCTTCTCAATCTTGTCATTTCTTTCCTGTTTTGCTGAAAATCCTGATTGTCGCACAAATGAAGGTGTAACATCTCTGATTTCAGTGATTACATAGCCACATTTTCGACATAGAATCCCGACCTCATCATCTATGGTAAACACATGCTGACAAGATCCATCATTTTTGAAGAATTTCTCCGGTTTTTCATCACCATCTCCGCTCTTTGGTTTCTCCTCATCAAGAAAATATGCTGAAGCTAAACAAAGTTCCATTTCTCGAAACAGCATATCAGTTTCTGACTCAGCTTCTTCTTCCTCCTCGGGAGCTGGAGCGTCATCAGAAGCCTTCTTTTCATACATAGATGAAGCTCCTTGAAGGTTTTTCCATGCTTCAGTAGGAATCGGTTGTTTATTCTCGATTGTCAACTGTATGTTCTTCATGTAAGTTGATATAAGGTCCTTGTAAGCAGCAATATCCAATGTTTTCTTTTTTCTAGACGGATCCTCATTGTTCTTATCGCTCCTGGAAGAAGGAAAGAATGAAGGACGCGGCTTTTTCTTCTGATATGATCTGTTACTCACACTCCTTCCTCGGTATAATGGTTCATCATCAAGAGCATCCAATTCTGAGATCGTCTTTGTGCGTGTTGCTGCTGTTCTTGTTCTTGTTACACGCGCTTTAAATCTTCTTGTGTAGAACTTCAAGGGGACATTATCCTCGTCAACATCAAGGGGATTAGGAAGGATCTCGTTTAGGAGATTACTTCGTTCCTTAGGCACTCGGTTGCTTTCTTCAGAGATAGCAACGATGGCAAGGTTGTCTTGCTTATTTTGTTTCGCCCTTTGAACCTTTGTTGCCCTTTTCTCTTCCTCATTATCGGCATTTTCCCCCTTATTTTCTTTCTCTCTTTTGGTATATTTCCGCTTCTTATCATTCTCTTTGTGCTCGCCATCATTCTTCTTCTCCCTTTCCTTTTTACTGTCTCGAACACGTTTCTCTTCTTTTGCCTCAAGTCCAAGTTTTGGAACATGAAGAGTTTCTCCCGGGTTTGCCTCACCAGCACCTTCGAGGGTTTTTTGAGGATCCTCTAAAGCAGCAGACTGACTTGTATCGTCTTCATTTAAAACTATAATGGCTAAATCATCCTGATTCCAGAACTTGTATCCGATTCTGAGTGCAGTAGTATTAGCTTCCTCAAGACCGATCTCACCCAAGTATCGCTCAGGCTGTATATTCCTACGCTTTGACCGCCTTAACTCCATAAGTTCATATTGTGCAAGTGCATCCACTTCCTCCTTTACCTCCCGAACAGGTGCTGCTGTACTATTTTTGTCAACAGGAACAAACGGAAAAACTTCTGAGAATGTGATTTCACCATCTTTTCTGAAGTTTACACCACTAAGATGGACTGCAGTTGAATAAGTACTGAAATTCAAGTCCAATATTTGGTAGACTATCTTCTTTTCTATCGAACGTACATCAAATACAGTCTGCTTCTGGACTGATGCAACCAGAAGCCAAGAAATATCAGCAGCAAACTTCGACAGGAATAACTTTGTCCTTGACAATGAAGTGCAGTCCTCCGCAAGGTCCCACCTATAATGCGTATTTTCAGACGGCTTTTGCTGGAGAACTTGAAAAATAGATAATTCATTGATCCCAACAGTTTCAATTTCCTTGCTTAGCTTTCTTCTGTCAGTTCCAGCATCGTCACTGATGTAGTAGAATTTGATGTAAAACTTGCAGTTGCATTCACTTCCATGTGGGGTTCTCTCAATCGAAGTTATTTTTGCATCAAACCATCCGGTGGGAGGTTCTTCAGATGATTCTCTCAGACTGTCATAATCATCAGATGATGAAAATGCAGAAACATCAGCACCAGGTCTTAGTACACAGGTGCAGTCGTTCATAGTTGCTCTTCTTGGTCGTAGCCTTAGTGTAGGAAGGAAAAACTTTTCCTCGTATAAATATCCGAAATCCAAAAAGTTAAAAGTCACCGTTCCAGCGCTGATTCTTAACCGATCCACATTCTTCCACGAGCCATGATAAAATACCTCAAAGGGATGGTCATCGAAAGGATGTAATGACTGATGAAGACCACGTTTCCTCATTGCGTCCACGTGATACAGTTAACACGAGGACCACAGAGTTATGTCAGCACAGCAGAAGACCTGGTAATCTCCTTTACTAAGTCCACTCTTTCACCTGAACATGATGAATTTACTCAATTCAGTACAAACACTAGATTTTACAACAGAAATGTGAAGAAACAGAGAATCAAAGGTAAAATTACACTTTCTAGTGAAGAACTACATACAAGTGAAGTAGTATGACTGAAGAGAGAGAGAAAATAAGCGGAGAAATCAATGGTAAAAATGAAAAAAGTGGCACAGAAATTGAAATTGAGATTCTGTAAAATGGTTGTCTTTTTAGTAAATTTGTGTGAAAATTAGTGAAAAAAAAAA

*S.maritima*42249

CTGATGTTAAAAAATTCCATAAAATTTTATCAGAATGAATGTTTCACAACAGTAAATGTCGTCTATTAAACTGCATCTTATGGGTTTCCATTGATAAATATCAGTAGTATACATTATATGACAGACAGTATATATACCAGAATACCCTCAAGGCCTCAACAAATCACCAACCATCTACGCAGATGAAGAGGTCTTCTGTTACTTCTTGTAGAGGTAGCATTTGTTGCGCAATACAATGTAAATAAAAGTAGATCTTTTGACTCTCGATATGTGAAGTGTCTTCGGCTAGAAAATGATAAATAGATCCTCCAACTTCAATCCAACTAGAACCTGCTTCTTTGTATACAAATTTCTCTTTCATTTCCCTTCTCAGCCTTCTCACCGAACTCCAATCGTGTGCTCTAGCATAAAGATTTGACAGAAGAATGTAAGCAGAGTCATTGTCAGGCTGCAGCTTAAGTAGTTCCTCTGCAGCAACTTCCCCGACTTCTAGATTACCAGCATTGTGACAGGCAGACAGCAGACTCTGCCACACCAGAGCATCAGGGCTAACAGGCATTTTGTCAATGAGTAGACGTGCTTCTTTTAAAAGACCAACTCGACCTAATAAGTCGACCATAGTAGCATAATGTTCTAGGCACGGGACTAATCCATGAAGCTCAAACATAGAATTGAAGTGAACCTTTGCTTCATTAACGAGTCCAGCATGACAGCATGACTGAAGGATTCCAAGATATGTTATTTCATCCGGCTTTATACCTAGCTCAGTCATCCTTTTAAGCAAGAAAATAACTTCATTGTAACAACCATGCTGAGCAAATCCCATAATCATAGCATTCCATGCAGCTAGATTATGGCTGGTTATATACATAAATATGTCCTGTGCATCAGCAACGCTTCCGCATTTGCAATAAAAATCAACCATAGCAGTCTCAACAAACTTATCACTTCCAAGTCCAGCTTTCAAAGCCAAACAATGAAAAGACTTTCCTAGGCCAAGATTTGTCAAAGAACTACAAGCTTTGAATACTATACTAAAAGTAATACTATCCATTTCAGGCCACACCCTCATTGCAAATTTGAAGCATTCCACAGCATCATCAGGGCAACCTGCAGTGATGGCAGCCGCTGCCATTACTTGGAGATGTCCAACATTAACTTCTTGTATTGCACATAATACCCTCTTTGCATGGTCCAAGTAAGAGCATTTACCATAGGAAGACATCAAACATGAAATCATTGAGTCATCAGATATCAATCCAGTTTTAATGATAAGAGAATGAACTTGCAACACAAGACCAGCAAAATCAGGACCTGTAACTGCTCCAAGAACACTAGCAAGGGTATACTGATCAAGCCGAACACAAAGACTACGCATTTGACAAAATACACGTAGCAGTGGAGCCAATGATCTATTTTCAGAATACCCCCCAACCAAAGAATTCCAAGATATAGAATCACGAAAATGTAATCCATCGAAAACAAGCCTAGCATCATCCATCTCTTTACACTTCCCATACATGGATATAAGAGCATTACCAACTGATATAACGGTCAAAAACCCAGCTTTGTGGCAAATTGAATGAACTTGCCTCCCAGCTATCAACAACTTAGGGTCCTCGATTGCTGATAATACATTCACAATAGTAAGCTCATTTGCTACCACATTATTCGACCGCATAAATTTAAAAGCCTCTAGAGCCTCCTCGCCATCACAAGAAGCCCCAATCCTAGCAGTCCACGATACAACATCCGGCTTAGGAATTTCCTCAAACACCTTAACCGCATCGAGTCTACTCCCACACCTAGAATACATATTCATCACCACATTACTCAAATGAAGTGAACCACCAAAAACCAACCCAATTTTAAGAGCAAACCCATGAATTTGCTCGCCTTCTCTAATCTCCCTTAAAGCCCCAACTACAGCGGTCAAACTAAACTCATTCAATTCCAACCCTAACCCTCTCATTCCCCTAAAAACCAATCTACCTTTCTCAAATTCACCATTCCAAACATACCCATTAATCATAGCAGTCGAAACAACAGAATCCACATCCAAACATTCCTCAAAACACTTCTCAGCAAATTCAAGATACCCACATTTCCCATATTTATGAATAAGTCCACTAACAACAAACCCACTAAAATTAAACCCCAACTTAATACAATTAACATGGGCAATAATAATTTTTTCTATAGAATCGCATGATTTAATCAATGAACTCAAAGTATACTTATCTGCCCTAAGCCCAAAATATGTCAGCTTATTATACAAAGCAAAAGAAGAAGATGAATGATGAAATTGGGCATACCCAGAAAGCAGCGCATTGAAAGCGACGATATCAGCGTTGTTACCGTCGATAAAGAAGCCATATGAGCGAGAAAAGTCGCCGAATTTGGAGAAGTGAGTGATGAGAGAAGTTGCGAGGTAAGTGTCAAGGAGATGTCCTGATTTGAGGAAAAGAGAGTAGGTTTGATCAGTGATGGAAGGCGAGTAGAGAGAAAGCGAACGCTTAAGAGCTTGAGAATGGAGGTTTTGAGCTATGAGGTCTTTAATGGAGAAGAGAGAGTGAAGGTGAGGGGTGTTGGAGAAGAACCTCTTCATGCACTGGGGTTTTGGAATACTTTGCTGGGGTTTTGGTAGGGTTTAGTTGTTTACTGGACATTGCCCTATTGTCTACCGTCAGTCCGTTACCAGCCTTACCAAAACCCCAGCAAAGTATTCCAAAACCCCAGTGCATGAAGAGGTTCTTCTCCAACACCCCTCACCTTCACTCTCTCTTCTCCATTAAAGACCTCATAGCTCAAAACCTCCATTCTCAAGCTCTTAAGCGTTCGCTTTCTCTCTACTCGCCTTCCATCACTGATCAAACCTACTCTCTTTTCCTCAAATCAGGACATCTCCTTGACACTTACCTCGCAACTTCTCTCATCACTCACTTCTCCAAATTCGGCGACTTTTCTCGCTCATATGGCTTCTTTATCGACGGTAACAACGCTGATATCGTCGCTTTCAATGCGCTGCTTTCTGGGTATGCCCAATTTCATCATTCATCTTCTTCTTTTGCTTTGTATAATAAGCTGACATATTTTGGGCTTAGGGCAGATAAGTATACTTTGAGTTCATTGATTAAATCATGCGATTCTATAGAAAAAATTATTATTGCCCATGTTAATTGTATTAAGTTGGGGTTTAATTTTAGTGGGTTTGTTGTTAGTGGACTTATTCATAAATATGGGAAATGTGGGTATCTTGAATTTGCTGAGAAGTGTTTTGAGGAATGTTTGGATGTGGATTCTGTTGTTTCGACTGCTATGATTAATGGGTATGTTTGGAATGGTGAATTTGAGAAAGGTAGATTGGTTTTTAGGGGAATGGGAGGGTTAGGGTTGGAATTGAATGAGTTTAGTTTGACCGCTGTAGTTGGGGCTTTGAGGGAGATTAGAGAAGGCGAGCAAATTCATGGGTTTGCTCTTAAAATTGGGTTGGTTTTTGGTGGTTCACTTCATTTGAGTAATGTGGTGATGAATATGTATTCTAGGTGTGGGAGTAGACTCGATGCGGTTAAGGTGTTTGAGGAAATTCCTAAGCCGGATGTTGTATCGTGGACTGCTAGGATTGGGGCTTCTTGTGATGGCGAGGAGGCTCTAGAGGCTTTTAAATTTATGCGGTCGAATAATGTGGTAGCAAATGAGCTTACTATTGTGAATGTATTATCAGCAATCGAGGACCCTAAGTTGTTGATAGCTGGGAGGCAAGTTCATTCAATTTGCCACAAAGCTGGGTTTTTGACCGTTATATCAGTTGGTAATGCTCTTATATCCATGTATGGGAAGTGTAAAGAGATGGATGATGCTAGGCTTGTTTTCGATGGATTACATTTTCGTGATTCTATATCTTGGAATTCTTTGGTTGGGGGGTATTCTGAAAATAGATCATTGGCTCCACTGCTACGTGTATTTTGTCAAATGCGTAGTCTTTGTGTTCGGCTTGATCAGTATACCCTTGCTAGTGTTCTTGGAGCAGTTACAGGTCCTGATTTTGCTGGTCTTGTGTTGCAAGTTCATTCTCTTATCATTAAAACTGGATTGATATCTGATGACTCAATGATTTCATGTTTGATGTCTTCCTATGGTAAATGCTCTTACTTGGACCATGCAAAGAGGGTATTATGTGCAATACAAGAAGTTAATGTTGGACATCTCCAAGTAATGGCAGCGGCTGCCATCACTGCAGGTTGCCCTGATGATGCTGTGGAATGCTTCAAATTTGCAATGAGGGTGTGGCCTGAAATGGATAGTATTACTTTTAGTATAGTATTCAAAGCTTGTAGTTCTTTGACAAATCTTGGCCTAGGAAAGTCTTTTCATTGTTTGGCTTTGAAAGCTGGACTTGGAAGTGATAAGTTTGTTGAGACTGCTATGGTTGATTTTTATTGCAAATGCGGAAGCGTTGCTGATGCACAGGACATATTTATGTATATAACCAGCCATAATCTAGCTGCATGGAATGCTATGATTATGGGATTTGCTCAGCATGGTTGTTACAATGAAGTTATTTTCTTGCTTAAAAGGATGACTGAGCTAGGTATAAAGCCGGATGAAATAACATATCTTGGAATCCTTCAGTCATGCTGTCATGCTGGACTCGTTAATGAAGCAAAGGTTCACTTCAATTCTATGTTTGAGCTTCATGGATTAGTCCCGTGCCTAGAACATTATGCTACTATGGTCGACTTATTAGGTCGAGTTGGTCTTTTAAAAGAAGCACGTCTACTCATTGACAAAATGCCTGTTAGCCCTGATGCTCTGGTGTGGCAGAGTCTGCTGTCTGCCTGTCACAATGCTGGTAATCTAGAAGTCGGGGAAGTTGCTGCAGAGGAACTACTTAAGCTGCAGCCTGACAATGACTCTGCTTACATTCTTCTGTCAAATCTTTATGCTAGAGCACACGATTGGAGTTCGGTGAGAAGGCTGAGAAGGGAAATGAAAGAGAAATTTGTATACAAAGAAGCAGGTTCTAGTTGGATTGAAGTTGGAGGATCTATTTATCATTTTCTAGCCGAAGACACTTCACATATCGAGAGTCAAAAGATCTACTTTTATTTACATTGTATTGCGCAACAAATGCTACCTCTACAAGAAGTAACAGAAGACCTCTTCATCTGCGTAGATGGTTGGTGATTTGTTGAGGCCTTGAGGGTATTCTGGTATATATACTGTCTGTCATATAATGTATACTACTGATATTTATCAATGGAAACCCATAAGATGCAGTTTAATAGACGACATTTACTGTTGTGAAACATTCATTCTGAT

*S.maritima*44452

TTACCGCCCAAAACCCCATAACACTAGCCAAATTGCGAAAAACCTATTTAGCTGCTACCTCATCTCCTTCACCTGCTATAAACCCAGAAATAGAAGAAAGAAATCCCCAATTTCATTGTAATCCCATTGAAGGGTTTAAGCTTCTTCTACTTCCATTTTCAATTGAAGTCCATAAAGAAAACTAAGCAAAGATAAATCTGTGAGATATGCTTTCCTGATCATTTCTGATTGAATACAATATCTGAAGCAGTATTGGCATGAGTGCATGGCTTTGTCTTGGTTGTCTCATCTTCATATCAATCCATGGCCGACTGCATGCTTAGGAAAGACGTTGCTTATGCATGGAGTTGGCAGAAATCTTTGCAAATCGTATACACTCAATTTTTCCTCTGTTTTTTGTGTAAAGGGCTTTTCCAAGTTATCTTACAGTTCAGAACAAGATCTTACAAGATCTGCTCATGTCTCTTCTGTTAGTCATGTGGAGGTTCCCTTGAAAGTTGCACAAGGGATTGAACAGGGAATATTTTCCCGTTCTGTCAATCCCAATGTTGCTGCAGTAAGTGAAGCTCCTTCAGATGGTAGAGTTATGCTCATTGATGGGACTTCTGTTATTTACAGAGCCTACTACAAGCTTTTAGCAAAATTGCACCATGGCTATCTTTCTAATGCTGATGGTAATGGAGATTGGGTTTTGACAATCTTTTCAGCTTTATCTCTTATAATTGATGTTCTTGCATTCAACCCTTCGCATGTGGCGGTGGTGTTTGATCACATTGGAAATTCATTTCAATCATTTGGTGAAACTACTATTTCCTCAAAACAAAGTCTAATGTTCAAAGGCACTAATTTCCGGCATACCATGTATCCGCAATATAAGAGCAACCGTGGTCCAACACCTGATACTGTGGTTCAAGGACTTCAGTACCTAAAAGCATCCATCAAGGCCATGTCAATTAAAGTAATTGAGGTGCCAGGGGTTGAAGCTGATGATGTTATAGGAACTCTTGCTTTAAGGAGTGTTGAGGCTGGTTACAAGGTTCGAGTTGTCTCACCAGATAAAGATTTCTTTCAAATTCTGTCACCGTCCTTGCGTCTTTTGCGAATCGCCCCGCGTGGAGATGAGATGGTCTCATTTGGCCTAGAGGATTTTGCCAAAAGATTTGGAACTCTTAGTCCTTCTCAATTTGTTGATGTTGTTGCCCTTGTTGGTGACAAAAGTGATAACATCCCAGGGGTGACAGGGATAGGGGAAGTCAATGCCGTTCAGTTGATAACAGAATTTGGTTCTCTAGAAGAATTGTTGTCTAATGTTGATAAAGTAAAACAAGACCATATAAGAGAGGCACTGATATCAAGTACTGATCTTGCTCTCCTTTGTAAAGAATTGGCAATGCTACGGTCTGATCTTCCTCATTACCTTGTCCCATTCGCAACATCTGATCTGATCTATCAAAAACCAGAGGATGATGGGGAGAAATTTACAACCTTGTTGAATGCAATAAGTGCTTATGCCGAAGGTTTCTCAGCAGATCCTATCATCAGAAGGGCGCTGTATTTGTGGAAGAAGTTGGAAAAACAGTAACTAAATTTGCAAAAGTTCGAAGCAGCTGGGTAGTTCAGAAGTAGCAGATACAAGAAGCGAGGAACTTGACACTTTCACTATGATTTTTTTGTTGTAAATAGACATGCTTTATCAAACTGCCCGCTATGGCAGTGTGTAACATACATCCACAACATGTACGACTAACTAATAGTAATGAACATTTTGATCTTTAAGAATCAAAATTTTTGGTACAATACATGTGCATGTTGCCCCTGAAGTTTTAACAGATACAAACGACACAAATGTATCTTCTATGATCTTGACAAGGAAAACCTATACAAATGTATTTCTCCACAACGTTGAGAGCATTCATACTGATATCAACTCTAGTTCACACATTGCTGTAGTTACTTAACGATACAATATAGCTAGCTTACAAGCATGGATGCATCTGTTTTCATATGAACTCTTGCAATTTTGAACAAACTGTCATTTACACAATAGAACCTTTTGACTTCGAATTTGAACCTAGTATAAACTTAGGGCACATAATCTACTTAATTACGCCATGTTTAAAATCCAAGCGCAGATGGGGCAGAGAGCTAGAAAGTAAAAACAAAAAAGGATGTTAAACTATGTATTGAGTTACAACATTGCTAAATTTCAGCTTCTGTGGTAGCAGTCTCAGTTTCTTCATTGTCAAGCTCCGTTTCAGCAGCAGACTCAGTTTCTTCTTTTGAAAGCTCTAGTTCAACATTAAAGCCAAATGAATTTCCAGGAGAGAAGGATTGTTTGAGTTCATCTTCGCTTTGAATTGTTTTCATTTCATTGTCTTTCAAAGAAAGATTCTCCTGCTCCACATAACCTCCCACAGAAGAGGTAACTATTTCCCGAATGACAAAATTTGTTACTCGTTCTTTACCAAGTATATCCAGAAGCAAGTTCTTTGGAACCTTCGCTGTTTTTCCTCCTTTTTGACGACGGAAACCAGGAATTGGTGGGGCTGAGCGAGCTAATTTTGACAAGACCACGTCAAAAGCCTTTTGTGTTTCTTCTCCACTCAAGTCCACTCTCAATTTGATCTTTTCTGCATCTTGTGATTCAACAGCTATGTCTACTGTTTTAAGTCTCAGTGTACTCCCCTCTGAATCTGCCAAAGATGTCTCCAAACCTGAACCCACTGCAGAAACTGGCTTCAAAACATCTCTGTGATCTCTTCCTCGTAGTTGGTGAACAAATGACAGACTATTGCCGGATTCATGTTGAATGGTACAACATGCTACAGAAAGCTTTCTTGGGGCAGTGTAGCAGCATGCAACATTGACAAGGGAGTTGCGAAGAGGATGAATAGTTGGTGTTGAACAAATTGGAGTTGTCAAATTTGCCATTGACATTTGCTTTTAGTTCAATTTATGTATGGAGTAGTCGATTTTAGGGGATTTTGAGAATGTTTTTCTGTGTTAATTTCCCTGAATTATCTTCTTCTCTATTTGTATTTGTGCAATTGTGGCAACCAATTTTCTCACTGTTCTTTTATGAGTGGTTTTAACTTCACATTTTTCACCGCCGTGAGGACGAGGCACAGAGCTTCTTTCCCA

*S.maritima*87746

GTTGGTTCAAAACCTCAGACATGCTTTCCCATTGAAGATTTTGTGGGAACTGGAAATCATATTGACCGTCACTACATATATTCTTCTCAAATTTATATTTTTCTATTTTCCTAGAAAACCAAAGTACCAGTATTTAAAGAAAAAAAATCAATTCTTTTTCTCCGACTTTCTTTCTTCCTTGTCTCTGCAACTCACGCCATCTCTTCTTCTGCTTCTTCACTGTTAGTCGTTACTGTGGTCAATTGTGATGAATTCTTGGAAATATTGGTCTGAACAAGAGGGGGTGTGGTTATCGGAGAATGAGGAAGTTGATTTCCAATCAATTGATGGCAATGGATGTAAAAATAAATTGGGTGAAAGTGGAACTAAACCGACTTTTAATGTTGAAAATTATCCACTAATCTCTAACACTCAAGCACTTGATAGATTGGATTTTATGGATTTGGGGTATGCTGATGATATTAGGAAGTCATATTCTATGTCTAGTGAGCCAAGTGGAGAGGTTTTTAGTGAAGAAAACGGCACTGATTATCCTCCTACTCCGTTTGGTGAAGAAAGGGAATCAGGCTCTAGAGTTCCAACTAATGTTCTGCAATCAGATAAACAAGATTTTTCACTTGTTGATTTGAAGTTAGGTAGTTTGGCAAATGGTGGAATTGCCAAAGATATTGAATTATCAAAATCTGATGTAGCTGAGACATCTATGATGTTGTCTCCTTTGGCAAAGAAAGCGCGGTTGACAACGTTGAATACGCGATCCTGCTGCTGTCAAGTTCTTGGCTGTAACAAGGATCTTAGCTCCTTCAAGAGCTACTACAGGCGGCATAAGGTCTGTGATGAACATACAAAGACTCCTACAGTTGTTGTGGATGGGATTGAGCAAAGATTTTGTCAGCAATGTAGCAGGTTTCACCTATTGGCTGAATTTGATGATATAAAACGTAGTTGTCGTAGACGTCTTGCTGCTCATAATAAGCGTCGAAGGAAGCCTCAGTTTGCTTCTGAATCAGGTACTGGAGTTATTGACTTTTCCTCGTCACTTGCATTTCCAGAAGTGCTGCCTAGTGTTTTCCTTGGTCCAAAGAAATATGAAGAAGGGACGGGATATTCCGTCAAGAGCTCAAATTTCCTTCTTCAAATGGGTTGTGTAGGAGAAAAACTGGATAAGGTCCCTGAAAATGTGAAGGGCAGTGTTTTTGGCATGGCAAAACCAACGATAGAAAATTCTTCTAGCATTCAGCAAGCTAGTCGTGCTCTCTCTCTTCTGTCAGCTCAATCACATAACATATCAAGCAATTTGCTGGAAACTAAAAGCATGGGCATCAACAAAAACAGCCATGCAGATTCGAATCCGGCGCATGCTCCTAACCACTCGATCGACATTTTTAAAGATTTGGCAGAAAGTGGGCCCCGTGAAATGTACTCAATGGAAGCTGACGAGGTAGGACCTACCACGATTAGTGCAAACAACGACAACACAGTTGGCATGCAGCTCCCTGGTGATGGCTTTTCAACCAAGTCAAGCTTAAGGGACGCTGATGGTCATCAGCATAAGGGTCGTGGCATCATCCTTAACTTGCTCCAATTGTCTTCGCATCTTCAAAGAGTTGAGTCGCAGAAGCACTCAACTGAAAGTGAAGCAGGATAAACAAAAGTATCTCCACAAAAATGGCTTTATGCCTTTTCTTGCTCTGAAGGGCTGTGCATGCATTAAGAGTCTTACAATCAAATTCAAACTTGCTATGCCCAAGAATGGTACCTTCCCGCCAGCAGTAATCAAGCGATGTTTGATATCAGAAGCAATACTAAGTGGGTGTGAACAATTATATTCTTTGTACATAGTCGGAACTTGTTTCACAATACCGTAAGGTACGCAAGCTGCCCACTGCAAAACAACTAAAACAATATTAAACGAAAAGAAGCGAGGTAGTGGTCTTGTTGCCACCAAGAATATGCTGCTGCTTGTAAAATGGCAGATGTTCTGAAACTTAAGTCTATCTGATCCTGTGTCACTCTTAGGTACTGTCACATGTAATTATATTAAATTTTTTGACTCCATTGTTTCTGTACTTCTGTTCTATATTCCCAATGCCTCTCAATTCAGTGTTCTTTGTTGTGACTTGTGAGGTGCATTATATTTTTCTGTAGCGGTCTCACAGACAAGGCGCTAGGTTTCACTTTCCTCATCAATTTTATTGGCCATTGTTCTTCGTTTCAATGGTTGTGGTTCTGCTACAAAAGAGGCCGGGAGGTCAATGGAATTGGTTGGGTCTAGACGTCTAGTGATGTTGGATTTGATCGAGTCCGTTTAGATTGTATTGGCTCATATTCGTATTTTAGCTTGTCTTGAAAAGTTTTATCCAAGACCATAAGTTTACCTGGGTGAGGTTGTTTTTCCACCCTGGCCCGCATGATTGGATCAGACAAGCATGATAGTGGTACATAAATATTTTGAATATTTCACCCAAAAATTTGAATATAATGGAAT

*S.maritima*95321

TTGTCAGCAATGTAGCAGGTTTCATTTGCTGGGTGAATTTGATGATAGTAAGCGAAGCTGTCGTAAACGACTTGCAGGCCACAATGAAAGGCGGAGGAAACCTCAAGTGGGCTTAAGTAGTAGAAGTGGGAGATCATTTCACTCATACACGGGAAGCAAGTTTCAGGGGTTTACACCGTCAGCATCTTATATTTGTCGAGACATTCTTTTGAGAGCCACAACCTCTGCAGAAAAATATGGTTCAAATGATTGGATTAAACATGTAAAGCTTGAAAATGTGACAGATTTTGTTCAAGAACAAGCGTATGATTGCATATATGGACAGCTTCAACCAAAATCTAATTTTCCCTCTTACCATTTTGAGAAACATTTCCCATTTGCTGACAACAAAGATGACACTGTCTCAAAAAGTCTTGTTCACGGGAATGTCACTCAACACGTACCTCAAACAGTTTCACCAAAAAGTGAAGACTTGACACTTTTGGACGCAGCATCAACCGTTAATGGATTACCAAGAACATCGGAGTCCGGTTGTGCTCTCTCTCTTCTGTCATCTCAACCACAGAATTCTTCAGGTCATTCATCACAAATGCCGGAACCTGGAGTTTTAATGATACCAAGCACTAGTCCACACTACAGTATTAATGAAGACTCTGAAAAAGTCTTTGGAGTTCAAAACAAAAATGCTTTGTGCATGATCAGTTCTATGGGAAGAAATCAACCAAGTTCAATGTTGACATATCTTAGTGATAATACCTCACATTCTGACATTGGAAATGCGATGCACCACAGTTCCAAATTTATGAACATCAAAGATCAATTGTCGTGTGATATTGGGACAACTATTGATTTGCTTCAATTGTCATCTCAGTTGCAGAGAGTGGAAAACCAGTGGCAACCTCACCCTCTCAAGCAGGAAACTGAAGGTTCTTGCTGCCTAAGAATGACATGAGGGACCATACAAGAGGATAATAATAATATGAATGCGGGCAAGGGGCAACAACCATCAGAGAACTTGGACCCTGCTAATGCAATCTTCACTACCGCTCAGCTAACTGCTTAGTACTTGTAGGCTTGTAGCTCATTTGTGGAGACGGATTAATATCTCTGCTCTGGAACTAGTATATATGTCCTAAGCTTTATAGAAATTTTTTATTCTATGATATTAGCTCATTTTTAGCCTTGAAAATTCTGAAATTTTGAAGAGCCTGATGCTACATTTGACTGGCGTTTATCTTCATCCTAATATTTGCAATCGTATATTCTTGTTTT

*S.maritima*347871

TCTAAAACAAACTTATCTCAGGTACTATTTTTTACTTTCTCTTGCTTAATTTCAATTCAATTCTATTTTTTAGTTTTGCATTGTTATTAATTTCTAATAAGAAATCAAATACAAGATAGAAAATGGAGGGATATAATCATGATGATGATCAACAAAGAGGTGGATCACTTCAAGCTAGTACTATTGTGTACTGCCAAGCAGACAATTGCACGGCTGACTTGACCGAGGCTAAGCGATATCATCGTCGTCACAAGGTTTGTGAGTTCCATGCCAAAGCTCCGGTGGTCATCGTTAACTCGATTCATCAACGATTTTGCCAACAATGTAGCAAGTTCCATGAACTATCAGAGTTTGATGACACAAAGAGAAGCTGCAGGAGGAGGCTAGCAGGGCACAACGAAAGGCGACGGAAGAACTCCTTTGATGTTTCTGCAGAGAGCTCTTCAGGCGGCTGAGTGAGGCCACCGGAGAAAGAGAGATAAACAGCGCAATCAGAGAAAGAATATGGATTGATTATTAGAGATAAAACCATAGATTAACCCCCTTGAATTGCTTGAATTGAGATAAGCTAATTAACCTTGATTGATGAGTTGATCATATTATAATGAAGGTGTAATTAATGGTTTTATCACTAATAATGATCCATTATTATTCTTCTGAATGCTCTCTATCTTCTGTCAAACTCATGC

*S.maritima*219639

CAAAAATAATTAAATAATTATTATTTGTGTAATAGTCAATTCCAAATAAAAAAAATTTAATTTATATATATTTTTTGTATGTAATTAAATTGTTTATTTTCTTATGCCGATTGTTTCTGAGTACTGCCTGCTTTGTTTTGAGCAATAACAAATTCTGGATAATCTATAAAACCATCAAAATCTGTGTCATCCATAGTTAATATAGGATCAATTAACTGCATTAATTCTTCATCTGTAAATATCTTTTCTTGGGGAGGTGCTCCACCCGCTGCGGGATCATGACCACCTTGAACATGCCAATGTATAAGTGACTTGATAAGTTCACATCCATCTAATTTATTATTGTTATCTGCATCATGCATTTTGAAATAATGGAATTGTAATTCTTGTTCAGACATTTTACTTGTGTCTAATGTTGGTAGATCCAAATGTTTTTTTATGTGCTCGCTTTCTTCTGCTAAGTTTGGATTATGTAACACTTGTTGTGCATGAGGATGATGTCCTTGGGAACCATGGCCATGACTCGGTGGTTGCTGTTGTTGTTGTTGTT

*S.maritima*307759

TCGACTCAAAAAAAAATATTTATAGTAACTATTGTTTGAAATCTTACACTATCCATATTTAAAGATAGTTAACTTTGCAAAATACCTATCCTAAGATAAGTTATTGATGCAATTCATCCTTAAGCACTTCCATTGATTTGGAAATTTCGGCTTTATAAACATAAGACCTTGCAACGAGCAAGAAAAGGATCAAATTCACAATATTAAGTATGACGAAAAACCCATAATAGTAATCAAGATGAGATTTATTAAGGTTGTTCAATATCCATCCTTGGTGCCCATGCTCTTCTGTTACTCGAGAAACAGTTGATAAAAGGAAGCTACTTAGGTAATTTCCAACACCAAGAGTTGTAGTTGAGTAGGAAGTCCCGAGGCTCTTCATTCCCTCAGGGGCTTGATCATAGAAGAACTCAAGCTTGGCTACCTCGAGAACTGCATCAGCAACCCCCATAAGCATAAACTGAGGGAGTAAAATGAATATTGTTAAGGGAACTTGTGCACCACTTTCAACAACTCCATGTTCCTTTGCAGCACTTAGTCGCGCTCTCTCTACTAAAGATGATATGATCATGATATTTAAATGGAGACTTAGGCCTAATCCCATTCTTTGAAGCAATGTAATTCCTCTAGGATTTTTGGTTAATTTTCTCATAAACCTCACAAAGACTTTGTCATATAGAATAACAAAAACTAGCATCGAAAAGGTTGTAAAACCTGTTAAACTTGCCGGGGGGATATTGAAGTTACCTATACTCCTATCCAAGGTGGTTCCTTGCTTAACAAAAAGGGTTTGTACTTGAGCTAACATGGTACTTGGTACGAATGTAGTGATCAAGATTGGAATCATTTTCAGCATTTGTTTCGTCTCCTCTACTTGTGTAACCGTACATAGCTTCCAGGGATCCTTTGTATTAGTTATCACTGCGGCTTTGTTAAGGAACCTCAAGTAAGATGTCAATTCAATTCTAAACTTTCCGTTCTTCGTATATTCATCCAAATCAAGCTCATACAGTTGCTTAGGATCACTAGGGAGTGGTGTTTTCCTATTGTTAAAAGCAGCCACTATAACCTTTGCCATCTTTGTGAAGGGACTTCCATTAGGCAATTTATGCCTATAAAATGGAGTTCCAGCTAAAAAGATGGATATCGATATCGCAAGCCCAATTGTTGGAAGCCCATAACCCAAAGCCCAGCCCACATTGTCTTGGATGTAAACTAGAACTGTGTTGGCAAAGAGTGTCCCAAAGAAAATACTAAACATCCACCAGTTGAAGAAAGAGAATTTATGGGCCTTCTCCTTGGGCTCAAATTCATCAAATTGGTCTGCTCCAATTGTTGAGATATTGGGCTTGGTCCCACCAGTACCAATTGCTAAGGTGTAGAGTGCTCCATAAAATACAGCTAGATGGATTGGTGAGGCCTTTTTGCATTGGGCCATTTGGGCTTCATTGCAAAGAGGTGGCTTCAGCCCAGGGAGTGAAACTGCTAGTGTTAATACTGACATTCCCGAGAGATAGATGGCGCATGAAATGAGAAAAGTCCAATATCGTCCAAAATGCGCATCGGCGACGTAAGCCCCCAAGATGGGAGTCATCCAAATAGTACCAACCCAATTAGTGACATTGTTAGCTGATTCCACAGTTCCTTGGTGAAGCTTCGTTGTCATATAC

*S.maritima*18003

TCGCACTCGCACTCGCACTCGCACCAACCCTCAGAAGAAGAAGAAGCCTCCACCCTCACCCTCACCACCACCACCACCAATTAGCCTTGTTTCTCTCATTGACTCCTACATTAAGGTGGGGAAGAGTCTGAAGCCTATCCTGAAACCTTTGTATTCATTGATATGTTCATCTTTCAGTTATGAAGTTATTGAAGGAAATCATGATAATGACGCTGTTAAAAATGTTTCCAACCTTACATTTGATGACACAGTCACTCTTTCTGGTATCTTGTTTAAGGAGCTACGAGAAAAGTTGATATGTAGCTCAGGTGATGCATCTGTGGTTGGAAATGTCAAATTGCGTAAATTTGATGTGAAGAATTCTGGGGAAGAATTGCTTCTTCTTGTAAGATGTTGTGTTCTCTTGGTAAGACTGCATCCATATGATCAGGTTCTCACAGAGCAATTCCAGGTTGTTGTTGCTTTACTCCGCAAGCTGTCTTTCCATGGTATACCTCTTGGAAAAGTGAGAGAATCTATACAGTACACCAAACTGCTTCCTTCTCACAGTACTTTTCGTGGTTGTAAAAATTCTTCTCTTGATATCAATGCGAAATCAGTTGACTTCAATGCCAAATCACCTGAGCCATACTTGAGTTTTATACAACAAATGCAAGAGGTCTATCTGGATGAGTTGTTTGCAGACAGATCATTCCTGAAGTGTATGGCAATTTCAGACTCTGTATCTTCCACTTGCCAAAATGTGTCTGTGCAATGCTCAATGCAGATTGAGAGTGTCCTTGAAATCATCGCTGCTCATTTTCTTTTGTCCATATCTGGAAAAGATGCTCTTCACACTATAACAGAAAGATTATATTGGACAGAAAAACACGTTCTTTCTTCTCCTAAAGTGAGCTTTCTTGTGGCTTCATCATTGGTTAATCATCCTGTCATGAAGTCTGCTCCAACACTTCTCTTTTCACACGTGATCTCTTTATTATCTGATGTTATCAGCATCCATGTCTTTCCTGAGAAGGTGGGCCAAAGGACAGAAGAATATCTATCTGCATTTCATGATTCGGTGATTTTGTATCGCGAATTTTTGTCTATGTTGATGCCTGATTATTGTAGCCCTGAGAGAATTGTGCGAAAACCCTTTGAGTTTTATTTCCACTTCGAGATTTATGATAGAATTAACATTGTGCTTCTTGATTTGGATAATGCATGGAGGGTACAAACTCGCACGATGTTTGACAAGACAAAATCTAGCATGGCAATTGATGCTATTTCTTACATAAAGACGAACATCTGTATTGTTGATGAATCTTACAGAAAGGAAACACTCTCATTCCTTAGCTCATTAATAACAAAAATTGTTTCTGTTGAAATGAATGAAGTTCACTTGCAACGTTTTAAGGAAATGTTTGTGCAAGATTTTTGCTTACTGGCTTCAATTTTAAAACTGATGAGCTGCTCTTTGTTGCAAGCTACTCGCAATGATAAACAGGGCTGTTTCAAGTCATTGTTGAAGAGAAATGCTTTTCCGATGATATTCTTCTCACAGTACAATTTCCATGTATCTATAGAAGTAAGACAATTCATAGTTGATGCCCTGAAGCCGCATCATTCAACATGTAAAGAATATGAGTTGATGTTTGTACATTTTGCTGGCCTTTTATCATTATGCATAGTCAGTGGATTGCGCATCCTGATGAATGGATGCTTATTTATAATGCTAGTTCTTATGAATGCTTTAGCTTTGGAGCTGGGCAGTTTAGAGTTTTTGGGGCTATTGGTTAACTCAGGAGCAGAGTTGCATACTTCTTTGTTTTCTCCGGCCACTGTCAATAGCTTTGTTACTACTGATTCTGATTCTGGTTCTTTTGCTATATCTCCAAAGTTCCTTAAAGTTGCATCAAGGTTTCAAAAAGAAAGACGGTTGCTGTTTAGGGACCAAACAGCATCTGAAAATCCAGTAATATCTGGTGATGAAACTTGCAACGGAGAGATTTTCCTCAAATGTTTGATAAAGAACAAGAAGGAGATTGACGAGTTGGCTGATTTTGTTGAGTGCAAGCCATGGAGAGACTACAGTGCTCGTCTCTCTTGTATAGAGAATATAAGAATCAGGAGAATACCAAAGAGTAATAGACGGCTACAGCAAGCAAAGATACAGGTCCGCAGTTCTGAGAGAATCGAAAATAAAAGAAAGCGAGGTTTTTGAGGTTTGAAAGTACAAAGGGTCTATAAGTTAGATGTATAAATTCCAACTTGTATAGGTATATGTATATATGTAGATGGTGAACAGGGGAGTTATTAAGGACTCAAAGGTGTCCATACAGGAGGTAGAATTCTGACCCTGTGTGCTCGTTTAGTGTCCATCATCATACTGAAGGAGGTCGAATTTTGAAACTATGAATGATTAATCTCTTTTTTATTCCCTTTTGTTGTTAATTTTCTTATGCTG

*S.maritima*44627

ACGGAAATTTGAATTCCGTAGCTAAGTTTTGCCAAAATATAAAAAAATAAACACAAACCCCACTCATCCTCACACATTCACACGCAAACCCAAAAAAAATTACAACCCTTGGTTCTTACTCCAGAGATTTGTTTCGCACAAACCCATCAACCCATCAATAATTTATCATCTTCGAAGCCGATTGATCTTCGTGGTTGCGGATTCATCTAAATTAGGTTTCGACGCCGAATTGTCTTCTCAAGTCATTTAATTGATCAGTTCATGCTTGGTGTTGATTGAGTACTGTGATGGACTTGAATGTCTCACCTCCGGAAGATGACGAAGAGTATTTCGAACACCATTTTGAGGAAGATGTGGCACGACACGATCATATAGAGTCATCGGTTCAGACCCTTCGGCGGGAGCGAGAAGAACGAAAGGAACGATTGAGGGGTACAGATGATAGACCTATGCGAGTAACCCGACCATTCGAGCGTGATGAAGGGCCTTACTTGAAGAAATTCAAATCTGATAGAAACAAGTTACCCCCTGGTTGGCTGGACTGTCCAAAATTTGGTCAGGAAATAGGCTTTATTGTCCCATCTAAAGTTCCACTTGATGAAACTTTTAATGACTGCATCATTCCTGGCAAAAGATATGCTTTCCGTCAAGTGTTTCATCTGCTTAGAGTGAAGAACATCAAACTTGGTATGATAGTTGATTTGACTAATACAGATCGATATTATTCAACGAATGACTTTAAGAAAGAAGGTTTGAAATATTTGAAGATTCGATGCCAGGGGCGTGATTCTGTACCAGATAATGAGTCTGTAAACATTTTTGTCTATGAGGTGATGCAATTTATTTTCAGTCAAAATGAGAGGCAACCGAAAAAGTATGTATTTGTGCATTGTACTCATGGCCATAATCGAACTGGTTACATGATAGTTCATTATCTGATGCGAACACAGCAAGCTAGCTCTGTCACTGACGCTATACAAAGATTTGCAGAAGCTAGGCCTCCAGGCATCTATAAACAAGATTATATCGATGCACTATATGCTTTTTATCATGAAACTAAAAGTGACGTAGTTGTATGCCCCTCTACTCCTGAGTGGAAGAGGTCGTCTGATTTTGATCTAAATGGTGAAGCTCTTCCTGAAGATGATGATGGAAATCCATATGCTTCCAGCAATGAAAAGCAAGAGAATGCTGTATTGATGACCAATGACGATGTTTTGGGGGATAAAATACCTGATCAACAAGAATATCAATTGCAGAGATTTTGCTGCGACATCCTTAAGACTACTACGGCGAGGGGACACCTACAGTTTCCAGGATCACATCCAGTGTCTCTTGACCGGGACAACTTACAACTGTTAAGGCAGCGCTATTACTATGCCACTTGGAAAGCTGACGGCACAAGATACATGTTGCTCCTTTGTCCTGATGGGGCTTACCTAGCTGATAGAAGTTTTAAATTTCGAAGGGTTCAGATGAGGTTTCCATGCAAAAATATGACCGAACTTCACCATTACACATTGCTTGATGGAGAAATGATTATTGATACTGTGAGTCCTCAGAAGCAGGAGAGGAGATACCTGATCTATGACTTGATTGCAATCAACCGTGTTTCTCTCGCTGAGAGGCCTTTCTATGAGCGTTGGAAACTGATTGAGAAAGAGGTAGTTGAGCCTCGTAATCAAGAGCGGAGGAACATGGACACAACTGGCAAGCCTTACAGATATGATCTAGAGCCATTTAGGGTTAGAAGGAAAGACTTTTGGTTGCTGTCAACTGTTGGGAAACTGTTAAAGGAATTCATTCCTAATCTGTCTCATGAAGCTGATGGTCTTGTTTTCCAGGGCTGGGATGACCCCTACGTTCCCAGGACGCATCAAGGTCTCCTGAAGTGGAAATACCCTGAAATGAACTCAGTCGATTTTCTTTTTGAGATAGGAGAAGATGGTGCTGAAAATCTTTATCTCTATGATCGAGAAAAAAGCGATTGATGGACAACAACAGGGTTACATTTGGAGAGGATGTGGATCCATCTCCATACTCCGGTAAGATAGTGGAATGCTCGTTTGATTCGGAGGAAAACTTGTGGGTTTGCATGCGAGTCAGGGCTGATAAAACAACTCCAAATGAGTTCAACACCTACCGGAAGGTTATGAAAAGTATAAAGGACAATATAACTGAAGAGGTGTTGTTGAATGAGATTGGAGAGATCATTCGCTTGCCCATGTATGCTGATAGGATTCAAAGTGATAGCCGAGCGCAGAGGAAAGGGAAACCACCACCTCAAAAAAAAGATGTAAGATGAAGTAGACAATCGGTGCTGCCAGCTTTGGTCATTGAATTTAGATGTTCTTGCAGCAATTGGTTGTGTAATTAGGCATAATGGTAGCTTGTATATTAATTACATAATTATTAGCTGAGGGCTTAAATCTTTGTACTTTGTAGTTAGATTAGGAGATGTAGTGGTAGGTTATTTGTGTATATTTTCAGCATAAGTTCGCTTTCTTCTGTCACTCTCTTTCCCTTTAGTTCCTTTTGTATCTGTGACTGTGTTGAGAAAAGAGAAGGCTAATCTTGCAAACGTTAACAATTGCGAAAAGTGTACCATCTTTTTCTCTAAGGTCCTAATGTGTTGAGGATCCAAGGTTAGTATTCTTGCAGAAATTTGAATGGATTGTTGTAATGCTACGTACTTGTATTAGTTATACACTATCTTGTATCAGTCTATTTTTTTTAGTTAATTAATG

*S.maritima*1740190

TTGCTGCCCAAAGCACTGAAACAATCAACCCCTGGCCCTTAGCCATTAAACCGTCATACAAACTCCATACTCTGATTCTCCCACTGTAGACATACCTACTGGATTATTGCTTTCTTTCTCTGTCTTTCATGAGGATATGCTGCAGCACCATACAAGTAAGTTCTCCTAATACATGCTTTGGACAGATCACCCTAGATCACCCTGCTGTTTAGTGCTTCTTTCTTTGGAATCCCAACTGGCCCCATCTTAGGACAGTTTGGGGCACTCCCTTGTGGGAACTCTTCTGCTGCCACTTCTGGGGTGATTCCAGCATAGGTTCGGAGGGACGAAACCCCTTCTTTCCCTTGGGTGGCAGAGAGGATGTGCTTGCCCTCTTCTGTTTGT

*S.maritima*45455

TGGATGAAATGATGCGTGGACAAAAGGGTGGAGAGAGTTAGCAATTTTGCGGGGTGATAAAATAAACACATTTACAAGAATGATAGAGGGGCTTATATGGTCCCCGAGGGGTGGTGATGGCAATAAACATGATGATAGCTGAGCCTCTGAGGAGTGAAGTTGAATATGAAGAAAAAACTGTAAATTTGATTTGGCGAATTGATTTTGCTTGGGCCTTGGAAAGAGATTCGAAGAAGCACTGCGTCGTCGACATGGCAAGGATTGATCGAGAATCTGCAATTGGCTCATCAAGAGCATTCTGTTATCATTGATATTATTAACACTGTTCATTTTTTTCTTCTCAAATGAAGCTCATAATTGTTGGTGGTCGAAATGTGTGAGGTAGCTACAGGTTTATGCAAACTAAACACAGATGCCTCCTTCCAGTGTTAGCAAATATTGGGTGAAGGACCTGTTCTAAGGGATGTTGTTGGTGATGTTCTGGTTGCTTGCTTCTCATCTATGAATGGCGGTTTTGATGTGGATGTTGGCGAAGCTATTGCTATGCAGAAGAGTCTTTTGATTGTCCTTGAGTCAGGGTTCCGACGGCTGGTGCTCGAAACTGATAACATTAAGCTGTTTCATCATATGAAAAAAGGCATTACGCCTCCGACTGCTTTTGGAAATGTCATCAAGGACATTATTAGGCTGACTAGCTAATAAGGAGTATTTGTCTTGCTCTTTCTCTTTTGTTAAAAGGTTAGGGAATGGTGTTGCTCATGAAGTCATGAGTTAGCTTTGTTAAGTAGTGAGTATTGGGTTAATGGGTTTGGCTTGAGGAGTATCCCCTGATGTTCATGGGGCAATTTGTAATGACCTTGAAAGCTTGATGCCTTAATAAATTTTTGTTTCCCTTTCAAAAAAAAATGTGCGAGGTTTCATCAAAGATGCGGAGGACTTCGCAGGTGAGAGTGAAGATGCGAATGATAGCGTTGTCGAAATGCAAGTTGAGCATAGTGTAAAATTAAGAATTTTGATAAGAGCAACTATAAATTTGATAGAACAGAAGCTTTT

*S.maritima*42159

ACAATCTTGGATTGTTGGACCAACCAAATTTACAAAAGCAATTTGTTTGTGTTGCTCCATGAAGCACATCCAAACACTTTTCATCTAGGACTAAAACAAGAAATTCAATTATACCAGTACAAGCTACAACATTCAGCTTGACTTAAAAATCAATTGTATGCCTGCAAGTCTGTAACCATAAAAAATAAAACAAAGACTTGGAAAAAGCAAGTCATTCTTTGCAAAAGAACCAATCAAATTCATCTTCATCTCCTCCACTTGCCTTGCATATTTTCTTCTGTCATCAAGTCTTTAACAAGAACTCGAGTCAACTGCGGGTCCAATTCCCAACTGTTGGGATGTTGATTGTTGATCAATAATCACATTATAAAATTCCAACCTTTGAGTTAGTTCATCATTAAGAGAACCAACCTCAGTAAATGGCTGACCTTTTAAACCTTTCACTGCACATTTTCACCATTCCCTTCTTCCTCATCCTCGCTACATTAGTCGCCCGCTTGCATCACAACAATCTTGTCAAACTACACGGGTGCACAACAAGACACAGTCGAGAGCTTCTCCTGGTATATGAGTTTATTCCCAATGGAACTGTCGCTGATCATCTTCATGGAAATCGAAAAGAATCAGGAATCCTTTCTTGGGATATGCGGATGAAGATTGCTACTGAAAGTGCAAGTGCATTGTCATATCTTCATGCTTCCGACATCATACATCGTGATGTGAAAACAAACAATATCCTCCTCGATAACAATTTCTCTGTCAAAGTAGCAGATTTTGGCCTCTCAAGGCTCTTTCCTTGTGATGTCTCCCATGTCTCAACAGTCCCACAGGGAACTCCAGGGTACCTTGACCCGCAATATCACAAAAACTACCAAGTGACGAGCAAAAGTGATGTTTACAGCTTTGGAGTTGTACTTTTGGAGTTATTGACAGGAAGAAGGTCGGTTGACAAGACAAGACCAAGTAAGGAGCAGAATTTGGTGGATTGGGCCCGACCAAAACTCAACGACAAGAAAAAGCTTTTGCAAATAATAGACCCTAGATTGGAGAATCAGTACTCCATGCGAGCAGCACAGAAAGCCTGCAGTTTAGCTTACTATTGTCTGAGCCAAAATCCGAAGGCAAGACCCTTAATGAGCGACGTGGTGGAGACTCTGGAACCTTTGCAAAGTAGTGGTGATGTTATGAATGAAGTTTCATCTGCATCCTCGTCGTTACCAGTTTCAGCCTTCAGAAGTAAGCGTCCTAGCCAATTTCCTATTAGGGGAGTGCCAGACTACCCAATGCGGCAAAGATTCCCTGGCAATGTTAGTGCGGCTGCTAGTTGTCGTTCCCCCAACCCAGCCTGTTCTCCCAGTGGTCCTGCACCTTGTCGAGTGAGATGACTAGTGTCTGTTTGACTAGTTGTGTACTATTACTAATGCCTACCTTACACGCAATCTCTAGCAAGTTTATTCCCGAGGTTGCGTAGTGTAATGTAAATGGTTTTGTTTTTCTTTCAATCTATTCTGTAGATGGCCATCATTTGTGTTCTTTTCTATCCTTTCTCCCCCCTGCATTTTGTTGTTAGTTGTGATCTAACCACTGGTGGTTTCAGTCGATTCTGTTTTTGGATAAATTGTGATTCCGGCCAGTGTTCCTCAGATTAGACTTAATTGTGGGATGTTTGTGAAGGTTGTATTACATATGGAGTTATCACTTGTTCAA

*S.maritima*1265026

ACTTGAACTTCATTGTGTATACCGTGCAAATGACCAGCTAATGCAATTACATTCTTGTAATACATTTCCCCGATTCTTCTCAACTCATCTACCGTGACATTGTTATTATAAGGCAGTGTTGCTAAATATTGTTGAGTATTTTCAATTTCATTTTTTAATATGTTTCCTTTCTCTTGTAATGATTTGATAAGCTCAGCAAAGTATTTGATATTAGACTCCATATCTGTTTGGACCATTGCTGGATGGTCATGATTACGTTGAGCAATTTCAAAATCTTGTAAACACTTATTAGTTTCATATTTTAACTTTTCAACAGATGTTTTTTGTTTATCTAAATTATGTTCTATAAACTGTAAATTTTTTTGACATAATGTTATATCTCCAGCGACTTCATTTGAACCTTTAGATGACATTCGTGCCAATTCAGTGCTCATTGTTTTTTGTTGCTTAAGAAAATCTTTTAAATTATTGACTAATTGTAAAATTTCATTAGGCACAGGCATTTCAAGAGCAGGCACAGCTTTATTTTGTGCTTGATTTAAATTGGAAGTTGTATCTAGGCCACCAAGACCACGAGAAACAGGAGTGGCGGAAGTTGTTACATTAGTAGTTGTTCCTAATGTCAATTGTGTACCAGAAAGAGTTGCAGTAGGTTTGGCATTCAACGCTGAAGTTCCAA

*S.maritima*32657

GTTAGAAATTGAGAAATTACGGAGTAAAAATTTGCCGAGTTTCAAACACCAAACCCCAAAACCCCGAACAAGTTTTCTTCTCCGTAGTCACTCACTTCTAGGGCTTGCACTCACTCATTCTGCTCAAACTCAAACACCTCCATTTGCAGCAACTCAACCTCAACCTTAACAATGGCGACTGATGCAGACATGAGTGGTTGGACTGACCTTCTTCATTCTTCCACTAAACTTCTCGAACAAGCTGCTCCTTCTGCTCAGTTTCCTCCTCTTCAGAGGAATTTGGATCAATTAGAAGCTTTATCGAAGAAGCTCAAGGCTAAAACCCTAAGTAGCGAAGCTCCTTCTCACTCTATTGCTGCTACTAGGTTGTTGGCTCGTGAGGGAATTAATGCAGAGCAACTCGCAAGAGATTTGAAGTCGTTTGAACTGAAGACTACATATGAGGATGTTTTCCCTGCTGAAGCAACTACTGTGGAAGAATATCTCCAACAGGTACATGAGATGGCAACGCTCTCAGCCATACAGGAAGCTCAGAAGGATAATCTGAGAAATTTCAATGACTATATGATGAACGTTTTGGAGGATGATTGGCAAAAGGAGAAAAGGGATTTTCTTCAAGCCTTGAGTCGTATTTCAGCGTTGCCACGCAAGAATACTGGAGAATCAACTAGTGCAATAACTCGTCCTGGTCAAATCATATCATTGGCTTCGACTCCTCAAGCCCACAGTGGTGCTTCTAGTATGGACATTGTCCCTCATGTTAATAAACCCATCGAGGAGAAAAAAGCTTCAGTTTATGCCGAGGTTGTGAGCAGCCTCAATAGTGCTAGGGAGCGTGCTTTATCATTTAAACCTGCTACAGCATTCAAGGCTGCTTTTGAGAGTTTGGGTATTGAGGCATCTGGGGGAAAATCAGTAGGCGTGCAGAAGATATGGCACCTTGTGCAGACATTGATGAGCGAGGATCTAAATGTCCAGCCAAACATTTCAAAGAAAATGTTATTGGTGAATGGTGCTAGACGACATTTGGAATGGGGACATGCCAAACATGTTATTGAAACAATACAAAATCACCCCGTACAGGCTGCACTTGGGGGTTCAGTTGGTAATCTACAAAGAATACATGCCTTTCTCCGGATCCGTTTACGAGATCATGGGGTTTTGGATTTTGATGCCACTGATTCTAGAAGGCAACCTCCTCTAGATACAACTTGGCAACAGATATACTTTTGTTTAAGAAGTGGGTATTATGATGAAGCAAAGAGTGTAGCTCAGTCATCTCGTGTTTCTCTTCAGTTTGCTTCTCAGCTTTCTGAATGGATATCTACTGGAGGTGTGGTATCCGCAAATACTGCGGCTGCAGCTTCTGAAGAATGTGAAAAGATCTTTAGAATGGGTGATCGTGGGGCTCGAGCAGTGTATGACAGAAAGAAATTGCTACTTTATGCTATGATTTCTGGATCTCGCCGACAGATTGACCGCTTGCTTAAAGAGTTACCAAATTTATTCAACACGATAGAGGACTTTTTATGGTTTCAGTTGTGTGCCATACGTGAGAGTTCTCATGGCGTCTCATCCTCAGTACTGAATGAGGGTTCTGTGCCATACACTTTGGAGGATCTACAAACATACCTTAACAAGTTTGAGTCATCATATTATACCAAGAGTGGGAAGGACCCTCTGATGTACCCTTATGTTTTGCTTTTGAGCATTCAGTTGTTACCTGCTGTCATTTACATGTCAAAAGAAATGGGTGAAGGTTTTAGTATTGATGCAGTCCACATAGCAATCACATTTGCAGATCATGGGGTTTTTTCAGAAGGTGCTGCTGCAGGACAGAGGCTGGGAGTGATGGATGCTTTTGCTGAAGTATCTAGCATGATAAAGCAGTATGGTTCTGCATACTTGAGAATAGAAAACCTTTCTATGGCACTGGAATATTACGCACAAGCAGCTGCTGCAATCGGAGGTGGCCAGTTATCATGGGTTGGAAGATGTACTTTAGATCAGCAAAGGCAGAGGAACATGATGCTGAAGCAGCTTCTTACAGAGTTGTTGTTACATGATAATGGAATATATCTCTTACTTGGATCTAGGGGTGCTGGGGAAGAAGCTGAACTGAGACGGTTTTTTACTGAAGCTGCTTCACGGCAACAATTTCTGCTTGAAGCTGCTCGGCAGTGTCAGGAAACCGGTTTGTATGACAAATCCATTGAAATTCAGAAGAGAGTTGGTGCATTTGGAATGGCTTTAGATACAATTAACAAGTGCTTATCTGAGGCAGTTTGCTCACTGTCACGTGGTAAATTGGATGGTGAGAGTCAGACGGCTGCACTCATCCATTCTGGCAATGAGATATTGGAGATGTGCAAGTATTATCCTGAGATCAGTGTAAAAGATAGAGAACATGTTATTAAACAAGAAACTGTCTTGAGGCAGCTTGAGGCAGTACTTTCAATTCACGAACATTCTCGGCAGGGCCGCTATATGGATGCATTACGAGATATTGCAAAGCTTCCTTTTCTTCCACTGGATCCACGTGCACCAGGGTTGACCATGGATTCGTTCCAGAATCTATCCCCTTATGTCCAGGCTTGTGTCCCTGATCTTTTAAGAGTTGCCTTAAGTTGTCTGGATTATGTGACAGATACTGATGGATCTCTTCGTGCTTTGAAAACAAAGATTGCAAATTTCTTGGCAAACAATCTGCATCGAAATTGGCCACGTGATTTGTATGAGAAGGTTGCAAGAAGCCTTTGAAGTAAAGCATTAGGGAAAGATTGGCACCTTGAACTGTGTTACCATTTGTTTACTTTCATCTGTCAGGTTCTGGTGCCAAGCGCTTGCCATACTGCTTGCACATTTTGTACAAATATGGAGTAAGAGCTTTTTTTTTCCCCTTCTTTTTGTTTCCCTCCCTCCTTGAATGCAATCATTTATACAAGGTCGGTGTGTAAAGCTGGTTATTTAAGCACCACCTTTATTTTATTGCCATCAAAAACTCTCCTGTAGCTAGTATGAACGGCTAGTAGAATCCTATGCAGCAATGCTATATCTTAGCTAATATAACTTATGTATGTCCTAGATTTCTGATATAGAGAAAATGAATTTTTCGATGTGTATCATCCTTATTCTTAGGTTTTTCTTGCATTCAGTTGATATTTTTTTTAGGCAATGCCATTTTAGTCGATACTATCTTGGTCCCTGGAAGTTATATGATCATAGATGCCATAGTGCAATGGTATGGTCTTGTCTCTTGTTCAAAGTCTTTTAAACACAAACACAAATTCTTTACTTGCAACTTTGATGTTAATACTCCGTATTTTATATTCGAC

*S.maritima*34109

GTTCATTTTACCGGTCCAGACCGGATATTGAACACCCACTCGCATTTCGCAAACAATCCGACCTCCTCCGGTCCTCCACGAGTCCGCGTGAGTTGAAAGCTAAACTAAAAGCCTTAAACCCCTGATTCTGATTCTGATTCTGATTCTTTGAAGTCTGTATTGCAAATTTGCAATTGCAACTTGCTCTCTCTTCTCTGTCATCTTCTTCAATTTCTGCACTACAATTCATAAAGGGTCTTAATTATGGGAAAGGACGCAAAGAAAAAGGAGCCTGCTGGAAAAGGTAAGGGAAAGCAAGCTGGTGGCAGCGATGAGGGCGCTTCAAAGGAGAAGGGTGGTGGAAAGGGAGACAAGCTTGGAACTTGCAGTTTTGTCAAAGCTAGGCATATACTATGTGAGAAACAAGGGAAAATTAATGAAGTATACCAAAAGCTGCAAGAAGGCTGGCTGGACACTGGGGATAAGGTTCCACCAGCTGAATTTGGAAAGCTGGCTGCTCAATATTCTGAATGTCCTTCGGGCAAAAAGGGTGGGGACCTAGGATGGTTCCCACGAGGAAAGATGGCTGGTCCATTTCAGGACGTAGCTTTCAGCACTGCTATCGGAGCCACCAGTGCACCTTTCAAATCTACGCATGGTTATCATATTGTATTGTGTGAAGGTCGAAGGGGCTCATGAGAAATGCCAGCATCACCTTTGCTACTATAGAAGATTATGATTCTGCTGCTGGCAATTGAGAATAACCATCTTTTTGTATGACTATGACTGCTCTTTGCTTGGTAAAGCTGAAGTTTTTTCTGTATGTTTGTGTGAACAGTTTGTGAATTGTGATCAATCATACATATTGGCTTGTGACTTGTTTCTCCATTTCAAGTACATAATCTTTTTTGTTGGATGGTATTTATTGATTCATTTCTAATCGTGGCTGTGGCTTGTGACTTGTGA

*S.maritima*88337

TGGCAATTAGATCTTCCCATTCTCAATATAACAACTGTAAATAATTCACTAAAACAACTTTGAAAATAAATAAAAACAATAAATATATATATATATATATCTAAAACAATACTATAATAATTAAAATATTTTGTGCTATTTCAAACAATAAGTAGCAATGTGCCATATTATTAATGATCGAGTATTCTCAGATTTCCAGACACTATCTTGTTCTCGAGAGTGGCATTTGAAGGGATGTCGATTCTATCTCCATGATTTGCAATTATAATAACAGTTCCTTTCAAAGAAACATTTCGACCAAATGTTACATCACCAGAAACTGTTAAATGATCCAATTCCAGTATGTCAGGAATAGTTGCAAAACGAGACAAAAAGTCTTTTACTTTTGCAAAATCTTTATCGCCCAGTTTAACTAACGGAGTTGTAGGAAACATTCTCAATGGACTCATTGACAATGATCCATTTCTCATGTGGTACAAGTTGCTCATCACCAATAAAAGATCAGAGGTTTTTTTAACTGGTAAGAAACGACTTCTTGGTACATTTATACCAAGACCACCATTAAAAGATTTCATAGCTGCACCAACTGCTGTTTCAAGTTGAATTATATTTAAATTGTTGTCCAATGTTTTATTATTGACTATAATTTCCAAATTCATGGATCCTTCGTTTAAAACTTTATCAATAGCATCAAGTTTGATCCATAAGTTGTTAGTGTTAAAAAACTTAAACTTTTTTACTGATTTAAATTCTTCAAGATGTTCTTTAGGAACTTGCGCTATTTCCAATAGTCTCAATTTATTCTCATACTGAATTAATGTACCACCTTTAACATCAGCTTTAGTTTTATTGGTAACTTCCATTACAAATTCAAGAGGATTAGTTCCACGGTCTAATAACATATTGAGGATATTTAGGTCAACAGTTGCACCTAAGTTGTCAATGTTTGAGATGAAACAATAATCCCGACCGCTATCAATAAACTTTTGTAATAATCCTGAGTTTTTAAAACTATCATAGAAATCTCCGTGACCTGGAGGATACCAAGCCTCAATGTTGCCTTCAATATCACAGTCTTGTGCAATTGGTAGAAGTGATTCTTTAGATACTCGAGGAAAACAGCTTTGGTTAAATGTATATATCTCTACTTGAAGTCCTTTATATTTTCGAATAATTTGTTCAGTATCTTTATCCGTATTAAAAGAGTTCATTAAAACCAATGGTACATTTACATTGTATTTTTTATTTAAGTGCTCAATTTGTTGAACCGTTAAATCAAGGAAGGTAAGATCATTTCTGACTTGTATAACAGACTTTGGACCACGGCATCCCATGGATGTACCAAGACCTCCATTAAGTTTAACTACCACCAATTTTTCAAGCTTAGAATGAATATCATCGGGGCTTGGTTTCGATAAAGTATCATAATCTCGTATAGCATCAGTAGGCAATTTTTCAATTTTGTCCCAATCAACTGATGGGCCAGATTCTTCCAGGAAACGTTGAAATAAGCGCGAAAACGCTGTAAATTGAGACTTTGCTAAATCTCTTTTGTCATCTGATGTTGTTTTAAGCAAAGCGGTCATTTCTTTTTCTAATTGTACCAATGAATCACGTTGAGTAACTTCTTTGAACTCTTTCAAGTCTGTACTTGAAGGTACACGAGAGTGTCCTTTTGGCTTAACGACATTTGTGGAAATATTTATTCCCGAGTATCCGACATTTGTTTCCTCGACCATTTTTTCTAACAGAATTTTACAATATTTATTTTACTTTACTCGAATACAACGTCAAACGAACATCCGTCACTATGAGCACAACATAAACTGGCCTTCCCTCACTCATAAAACCAGTTTTTATACAGCTAACTCATGTAGTTTTT

*S.maritima*966826

AATATTTAGTGAAGTCTAGTTGCTATTGATATAAAATTAATATTAACAATAAGTATGAATAAATATAAAAAATTAAAAATATAATTTTGTCCAGTAGTGGCAACAGTATTATAAGTCTATAATTCTTCCCTCCGTTTTTGTATTTTGTCTTCCAGTATTTTGTGTAGCTTTTGCACATTTCTACTCTCTTTTTTAATTTTTTTTTCAATATCATCATGTTCATTTCCAGCTGCTAGATCCACATTAAAATGTCGTAGTTTTAATAACCGTCGCTCATATTCTTTTAATTCATCATGCAGAAATATCAGTTCGTCTTTACTAAAATTTGTTTCTTGAGCTTTAATCCAGATTTCTTGTACTTTAGGTTCCACAAACTCTTCACTATTTGGACCGGTGAGTGATAGCCGATGAAGACGATCAAAGCTATCTTTTATATCACGTACTCGATTTTGTAAGTTTCTTTGTTTATGACTGAAATCTGTAAAATCTTGAGATTCGTGTAATGAATTGAAATCATCTTTTAATCCTTTTTTTGAAATCGCAATGGGTATATCATTTTGCATGTGTTCAATTTCATTTGTCAATGAATAAAATTCATCAATTTTATTTTGGTGGCTTAAAAATTCATCTTTCAGCGCTATTAGCTCAATATTTGTAAAACCAGATTGTTTAGCTTGGTCCCATAATTGATTTAATCGTTTATCATGTAATAAATATTTATCAACAACTTTAGATGAATCTGCAGAAACACGATCTTGAGTTTCAAAATGTGTATGATTAATTTGAGATTCATTCACACTATCATCAAAATGATAGCTTAACTTGTATCTTTCCAATATACCTAAGAGTTTTTTTCGGACAATAGATTCCATTTCCCCATCTTTATCTAAATTTTTGGTTTTGTAGATCTTAAGTTCTCGCTCGTCTTCATCATGAATTCGCAATTCTGATAATAATGATCTCAATTCCGGTTCTGAACATCTCTGTTTAGCTTTGCTCCAAACTAAATTTAGTTTATTAATTTTGAATGGTTTATTAAGTTGCCGAACATTGAATTCTGGTTCATTGTTTAAGGATAGTTTTTTTTTCAACGTTTCGTTTTCGTCTGAACGTGAATATGCACTTACATAATAGCATAGAAATAGAATAATTAAAACTGTTCTTTT

*S.maritima*638009
[truncated: 192,257 more chars]
