# Supplementary material for: Novel and conserved miRNAs in the halophyte Suaeda maritima identified by deep sequencing and computational predictions using the ESTs of two mangrove plants
Source: BMC Plant Biol. 2015 Dec 29;15:301. doi: 10.1186/s12870-015-0682-3 (PMC4696257; doi:10.1186/s12870-015-0682-3)
Supplement: Additional file 11: — Targets of the conserved miRNAs mentioned in Fig. 2 . (DOC 283 kb) [file 12870_2015_682_MOESM11_ESM.doc]

**Additional file 11**

**Novel and conserved miRNAs in the halophyte *Suaeda maritima* identified by deep sequencing and computational predictions using the ESTs of two mangrove plants**

Corresponding author E-mail: sachingharat113@gmail.com

| Sr.No. | miRNA name | Target ID | Inhibition type | Target function |
| --- | --- | --- | --- | --- |
| 1 | sma-miR399a | *S.maritima*29157 | Cleavage | uncharacterized protein LOC100216569 |
|  |  | *S.maritima*32186 | Cleavage | putative transmembrane protein |
|  |  | *S.maritima*37741 | Cleavage | protein phosphatase 2C 16 |
|  |  | *S.maritima*6020 | Cleavage | cation/H(+) antiporter 20 |
|  |  | *S.maritima*13022 | Cleavage | acidic ribosomal protein P40 |
|  |  | *S.maritima*33760 | Cleavage | sugar transporter ERD6-like 16 |
|  |  | *S.maritima*11130 | Translation | MORN (Membrane Occupation and Recognition Nexus) repeat-containing protein |
|  |  | *S.maritima*35771 | Translation | cationic amino acid transporter 2 |
|  |  | *S.maritima*35771 | Translation | ationic amino acid transporter 4 |
|  |  | *S.maritima*31043 | Translation | TATA box-binding protein-associated factor RNA polymerase I subunit B |
|  |  | *S.maritima*13729 | Cleavage | aromatic and neutral transporter 1 |
|  |  | *S.maritima*31909 | Cleavage | Os10g0494500 |
|  |  | *S.maritima*1239142 | Translation | hypothetical protein precursor |
| 2 | sma-miR172c | *S.maritima*41386 | Translation | 3&apos;-5&apos;-exoribonuclease CER7 protein |
|  |  | *S.maritima*39645 | Cleavage | PREDICTED: uncharacterized protein LOC101292477 |
|  |  | *S.maritima*36703 | Cleavage | palmitoyl protein thioesterase family protein |
|  |  | *S.maritima*23484 | Cleavage | phosphoinositide phosphatase-like protein |
|  |  | *S.maritima*1034 | Translation | inorganic phosphate transporter 4;3 |
|  |  | *S.maritima*34425 | Cleavage | Arabinanase/levansucrase/invertase |
|  |  | *S.maritima*20865 | Translation | ankyrin repeat-containing protein |
|  |  | *S.maritima*44753 | Cleavage | Os11g0167300 |
|  |  | *S.maritima*43401 | Translation | uncharacterized protein |
|  |  | *S.maritima*33718 | Cleavage | ubiquitin-protein ligase 1 |
|  |  | *S.maritima*30120 | Translation | DEAD/DEAH box RNA helicase family protein |
|  |  | *S.maritima*602380 | Cleavage | RNA recognition motif-containing protein |
|  |  | *S.maritima*40612 | Translation | glycyl-tRNA synthetase 2 |
|  |  | *S.maritima*44113 | Cleavage | desulfo-glucosinolate sulfotransferase 18 |
|  |  | *S.maritima*44113 | Cleavage | desulfo-glucosinolate sulfotransferase 18 |
|  |  | *S.maritima*1382797 | Translation | Os01g0106700 |
|  |  | *S.maritima*44979 | Translation | ribosome biogenesis regulatory protein-like protein |
| 3 | sma-miR398a | *S.maritima*42692 | Cleavage | SubName: Full=Putative uncharacterized protein |
|  |  | *S.maritima*34359 | Translation | PREDICTED: pollen-specific protein SF3 |
| 4 | sma-miR169b | *S.maritima*39814 | Cleavage | magnesium-chelatase subunit chlD |
|  |  | *S.maritima*31847 | Cleavage | SubName: Full=Nuclear transcription factor Y subunit A8; |
|  |  | *S.maritima*25945 | Translation | putative TIR-NBS-LRR class disease resistance protein |
|  |  | *S.maritima*43442 | Cleavage | uncharacterized protein LOC100789738 |
|  |  | *S.maritima*220459 | Translation | Os01g0800300 |
|  |  | *S.maritima*44671 | Cleavage | putative ADP-ribosylation factor GTPase-activating protein AGD6 |
| 5 | sma-miR408b | *S.maritima*762928 | Cleavage | tyrosine/DOPA decarboxylase 1 |
|  |  | *S.maritima*31904 | Cleavage | PREDICTED: uncharacterized protein LOC101492369 |
|  |  | *S.maritima*1698407 | Cleavage | cruciferin 3 |
| 6 | sma-miR159c | *S.maritima*1271050 | Cleavage | hypothetical protein EMIHUDRAFT_460924 |
|  |  | *S.maritima*37714 | Translation | ELMO/CED-12 family protein |
|  |  | *S.maritima*40017 | Translation | Os01g0667700 |
|  |  | *S.maritima*6203 | Translation | beta galactosidase 9 |
|  |  | *S.maritima*28679 | Translation | CTC-interacting domain 7 protein |
|  |  | *S.maritima*45135 | Cleavage | Os08g0224000 |
|  |  | *S.maritima*32946 | Cleavage | Sti1p |
|  |  | *S.maritima*53673 | Cleavage | phytanoyl-CoA 2-hydroxylase |
|  |  | *S.maritima*25402 | Cleavage | Os12g0149300 |
|  |  | *S.maritima*346393 | Translation | alpha-amidating enzyme precursor 2 (ISS) |
|  |  | *S.maritima*37367 | Cleavage | uncharacterized protein LOC100806459 |
| 7 | sma-miR167d | *S.maritima*35346 | Cleavage | Os06g0609500 |
|  |  | *S.maritima*208405 | Cleavage | Os07g0501200 |
|  |  | *S.maritima*35358 | Translation | uncharacterized protein LOC100279635 |
|  |  | *S.maritima*36480 | Translation | Os01g0817700 |
|  |  | *S.maritima*863845 | Translation | putative regulator of chromosome condensation (RCC1) family protein |
|  |  | *S.maritima*24751 | Cleavage | SNF7-like protein |
|  |  | *S.maritima*38414 | Translation | pentatricopeptide repeat-containing protein |
|  |  | *S.maritima*37533 | Cleavage | IMPORTIN ALPHA SUBUNIT (KARYOPHERIN) |
|  |  | *S.maritima*1267768 | Translation | Protein kinase family protein |
| 8 | sma-miR319a | *S.maritima*25131 | Cleavage | pentatricopeptide repeat-containing protein |
|  |  | *S.maritima*1936037 | Translation | Os10g0469000 |
|  |  | *S.maritima*19691 | Translation | uncharacterized protein |
|  |  | *S.maritima*45062 | Cleavage | transcriptional regulator MED12-like protein |
|  |  | *S.maritima*37040 | Cleavage | conserved hypothetical protein |
|  |  | *S.maritima*40198 | Cleavage | cytochrome P450 71A14 |
| 9 | sma-miR169a | *S.maritima*25945 | Translation | putative TIR-NBS-LRR class disease resistance protein |
|  |  | *S.maritima*220459 | Translation | Os01g0800300 |
|  |  | *S.maritima*31847 | Cleavage | SubName: Full=Nuclear transcription factor Y subunit A8; |
|  |  | *S.maritima*44671 | Cleavage | putative ADP-ribosylation factor GTPase-activating protein AGD6 |
|  |  | *S.maritima*29592 | Cleavage | MATE efflux family protein |
|  |  | *S.maritima*39814 | Cleavage | magnesium-chelatase subunit chlD |
|  |  | *S.maritima*29358 | Translation | Toll Interleukin1 receptor-nucleotide binding-Leu- rich repeat-type resistance protein |
|  |  | *S.maritima*43442 | Cleavage | uncharacterized protein LOC100789738 |
|  |  | *S.maritima*39187 | Translation | sodium:hydrogen antiporter 1 |
|  |  | *S.maritima*32301 | Cleavage | hypothetical protein SORBIDRAFT_01g013430 |
| 10 | sma-miR171b | *S.maritima*13778 | Cleavage | F-box/kelch-repeat protein At3g23880-like isoform X1 |
|  |  | *S.maritima*44824 | Cleavage | scarecrow-like protein 6 isoform X1 |
|  |  | *S.maritima*717777 | Cleavage | cathepsin B-5880 precursor |
|  |  | *S.maritima*35742 | Translation | villin-1 |
|  |  | *S.maritima*558268 | Cleavage | transcription factor Dp-1-like |
|  |  | *S.maritima*40430 | Cleavage | E3 ubiquitin-protein ligase KEG |
|  |  | *S.maritima*333049 | Cleavage | ras-related protein Rab-8A isoform X2 |
|  |  | *S.maritima*1466 | Cleavage | F-box protein At3g56470-like |
|  |  | *S.maritima*233789 | Cleavage | putative disease resistance protein RGA3 |
|  |  | *S.maritima*26161 | Cleavage | pterin-4-alpha-carbinolamine dehydratase |
|  |  | *S.maritima*41508 | Cleavage | putative leucine-rich repeat receptor-like serine/threonine-protein kinase |
| 11 | sma-miR159a (Homologous miRNA, ath-miR159a) | *S.maritima*41732 | Cleavage | RNAligase isoform 1 |
|  |  | *S.maritima*43555 | Cleavage | RNase H family protein, putative isoform 3 |
|  |  | *S.maritima*44432 | Cleavage | zinc finger CCCH domain-containing protein 5 isoform X2 |
|  |  | *S.maritima*44459 | Cleavage | cytosolic enolase 3-like |
|  |  | *S.maritima*565601 | Cleavage | Putative gag-pol polyprotein, identical |
|  |  | *S.maritima*45062 | Cleavage | mediator of RNA polymerase II transcription subunit 12 |
|  |  | *S.maritima*40017 | Cleavage | probable E3 ubiquitin-protein ligase HIP1 |
|  |  | *S.maritima*44770 | Translation | mannose-6-phosphate isomerase 1 |
|  |  | *S.maritima*44550 | Cleavage | neuroblastoma breakpoint family member 1-like |
|  |  | *S.maritima*45135 | Cleavage | disease resistance protein RGA2-like |
|  |  | *S.maritima*45135 | Cleavage | polyadenylate-binding protein RBP47B' |
|  |  | *S.maritima*36986 | Cleavage | f-box family protein |
|  |  | *S.maritima*173919 | Translation | eukaryotic peptide chain release factor subunit 1 isoform X1 |
|  |  | *S.maritima*36986 | Cleavage | f-box family protein |
|  |  | *S.maritima*30865 | Translation | homeobox protein LUMINIDEPENDENS isoform X2 |
|  |  | *S.maritima*1952088 | Cleavage | liprin-alpha-1 |
|  |  | *S.maritima*275107 | Cleavage | Leu zipper protein p40 |
|  |  | *S.maritima*926096 | Cleavage | endoplasmic reticulum resident protein 27 |
|  |  | *S.maritima*20392 | Cleavage | putative retroelement pol polyprotein |
|  |  | *S.maritima*347872 | Cleavage | hypothetical protein OsJ_19676 |
|  |  | *S.maritima*26301 | Translation | uncharacterized protein LOC104895350 |
|  |  | *S.maritima*32405 | Cleavage | beta-hexosaminidase 3 |
|  |  | *S.maritima*32882 | Cleavage | non-specific lipid transfer protein GPI-anchored 2 |
|  |  | *S.maritima*42135 | Cleavage | uncharacterized protein LOC104883331 |
|  |  | *S.maritima*121777 | Cleavage | probable ubiquitin-conjugating enzyme E2 26 |
|  |  | *S.maritima*118987 | Cleavage | Outer arm dynein light chain 1 protein, putative isoform 4 |
|  |  | *S.maritima*12826 | Cleavage | uncharacterized protein LOC104904627 |
|  |  | *S.maritima*41751 | Translation | cytochrome b-c1 complex subunit 7-2 |
|  |  | *S.maritima*45469 | Translation | protein SUPPRESSOR OF npr1-1, CONSTITUTIVE 1-like |
|  |  | *S.maritima*6203 | Translation | beta-galactosidase 14-like |
|  |  | *S.maritima*28679 | Translation | polyadenylate-binding protein-interacting protein 7 |
|  |  | *S.maritima*14478 | Translation | DNA topoisomerase 2-binding protein 1-A |
| 12 | sma-miR168a | *S.maritima*43199 | Translation | pentatricopeptide repeat-containing protein |
|  |  | *S.maritima*46987 | Cleavage | UTP-glucose-1-phosphate uridylyltransferase (predicted) |
|  |  | *S.maritima*36827 | Translation | cyclin-A2 |
| 13 | sma-miR396b | *S.maritima*20428 | Cleavage | growth-regulating factor 3-like |
|  |  | *S.maritima*27150 | Cleavage | protein CHROMATIN REMODELING 20 isoform X1 |
|  |  | *S.maritima*27747 | Cleavage | growth-regulating factor 2-like |
|  |  | *S.maritima*27747 | Cleavage | growth-regulating factor 1-like isoform X1 |
|  |  | *S.maritima*27683 | Translation | putative fatty acyl-CoA reductase CG5065 |
|  |  | *S.maritima*29250 | Cleavage | RINT1-like protein MAG2L |
|  |  | *S.maritima*38649 | Cleavage | dnaJ protein ERDJ3A |
|  |  | *S.maritima*30680 | Cleavage | hypothetical protein |
|  |  | *S.maritima*37948 | Cleavage | growth-regulating factor 7 |
|  |  | *S.maritima*35644 | Cleavage | growth-regulating factor 4-like |
|  |  | *S.maritima*35644 | Cleavage | growth-regulating factor 3 isoform X1 |
|  |  | *S.maritima*38793 | Cleavage | transketolase, chloroplastic-like |
|  |  | *S.maritima*38542 | Cleavage | growth-regulating factor 9 |
|  |  | *S.maritima*36182 | Translation | CRS2-associated factor 2, chloroplastic |
|  |  | *S.maritima*647644 | Translation | ANKRD26-like family C member ENSP00000349402-like |
|  |  | *S.maritima*45408 | Cleavage | activating transcription factor 7-interacting protein 1 |
|  |  | *S.maritima*647287 | Cleavage | aquaporin-7 |
|  |  | *S.maritima*2539489 | Translation | Transcription initiation factor IIB, partial |
|  |  | *S.maritima*1490218 | Cleavage | Ty3/gypsy retrotransposon protein |
|  |  | *S.maritima*29634 | Cleavage | uncharacterized protein LOC104891875 |
|  |  | *S.maritima*655047 | Cleavage | calcium uptake protein 1 homolog, mitochondrial-like, partial |
|  |  | *S.maritima*28805 | Cleavage | nuclear pore complex protein GP210 isoform X2 |
|  |  | *S.maritima*28378 | Cleavage | DNA ligase 1 isoform X1 |
|  |  | *S.maritima*30069 | Cleavage | putative cyclin-B3-1 isoform X1 |
|  |  | *S.maritima*10708 | Translation | NAD-dependent malic enzyme 59 kDa isoform, mitochondrial |
|  |  | *S.maritima*25890 | Cleavage | probable zinc metallopeptidase EGY3, chloroplastic isoform X1 |
|  |  | *S.maritima*36451 | Cleavage | uncharacterized protein LOC104893104 |
|  |  | *S.maritima*10995 | Translation | magnesium-chelatase subunit ChlH, chloroplastic |
|  |  | *S.maritima*29081 | Cleavage | uncharacterized membrane protein At3g27390-like |
|  |  | *S.maritima*31247 | Translation | E3 ubiquitin-protein ligase PRT1 |
|  |  | *S.maritima*29081 | Cleavage | uncharacterized membrane protein At3g27390-like |
|  |  | *S.maritima*864747 | Cleavage | BUD13 homolog isoform X1 |
|  |  | *S.maritima*1272171 | Translation | hypothetical protein SPRG_06936 |
|  |  | *S.maritima*218708 | Translation | helicase and polymerase-containing protein TEBICHI |
|  |  | *S.maritima*41484 | Cleavage | uncharacterized protein LOC104891002 |
|  |  | *S.maritima*8922 | Cleavage | pentatricopeptide repeat-containing protein At2g15630, mitochondrial |
|  |  | *S.maritima*45073 | Translation | RNA-dependent RNA polymerase 2 |
| 14 | sma-miR156a | *S.maritima*15309 | Cleavage | putative G3BP-like protein isoform X2 |
|  |  | *S.maritima*26853 | Cleavage | SNF2 domain-containing protein CLASSY 1-like |
|  |  | *S.maritima*42249 | Cleavage | pentatricopeptide repeat-containing protein At2g04860-like |
|  |  | *S.maritima*44452 | Cleavage | DNA polymerase I |
|  |  | *S.maritima*87746 | Cleavage | squamosa promoter-binding-like protein 6 isoform X1 |
|  |  | *S.maritima*95321 | Cleavage | LIGULELESS1 protein, putative |
|  |  | *S.maritima*347871 | Translation | squamosa promoter-binding-like protein 3 |
|  |  | *S.maritima*219639 | Cleavage | multiple coagulation factor deficiency protein 2 |
|  |  | *S.maritima*307759 | Cleavage | protein NRT1/ PTR FAMILY 5.2-like isoform X2 |
|  |  | *S.maritima*18003 | Cleavage | uncharacterized protein LOC104898647 isoform X2 |
|  |  | *S.maritima*44627 | Cleavage | mRNA-capping enzyme-like isoform X1 |
|  |  | *S.maritima*1740190 | Cleavage | mitotic-spindle organizing protein 2B isoform X2 |
|  |  | *S.maritima*45455 | Cleavage | putative ribonuclease H protein At1g65750 |
|  |  | *S.maritima*42159 | Cleavage | serine/threonine-protein kinase At5g01020 isoform X1 |
|  |  | *S.maritima*1265026 | Translation | nucleoporin p58/p45-like |
|  |  | *S.maritima*32657 | Cleavage | nuclear pore complex protein NUP93A-like |
|  |  | *S.maritima*34109 | Cleavage | peptidyl-prolyl cis-trans isomerase NIMA-interacting 4 |
|  |  | *S.maritima*88337 | Cleavage | UDP-glucose pyrophosphorylase 2-like isoform 2 |
|  |  | *S.maritima*966826 | Cleavage | alpha-2-macroglobulin receptor-associated protein |
|  |  | *S.maritima*638009 | Translation | kinesin heavy chain |
|  |  | *S.maritima*43446 | Cleavage | nuclear-pore anchor |
|  |  | *S.maritima*1008713 | Translation | cell division cycle protein 27 homolog, partial |
|  |  | *S.maritima*2405104 | Cleavage | uncharacterized protein Gm4549 |
|  |  | *S.maritima*42840 | Translation | vacuolar protein-sorting-associated protein 11 homolog |
| 15 | sma-miR166a | *S.maritima*19497 | Cleavage | uncharacterized protein LOC104904626 |
|  |  | *S.maritima*32700 | Cleavage | homeobox-leucine zipper protein REVOLUTA-like |
|  |  | *S.maritima*38793 | Cleavage | transketolase, chloroplastic-like |
|  |  | *S.maritima*40112 | Cleavage | PHD finger-containing |
|  |  | *S.maritima*40112 | Cleavage | PHD finger-containing |
|  |  | *S.maritima*22916 | Cleavage | cell division protein FtsX |
|  |  | *S.maritima*42150 | Cleavage | uncharacterized protein LOC104889847 |
| 16 | sma-miR164a | *S.maritima*955933 | Cleavage | metalloendopeptidase / zinc ion binding protein |
|  |  | *S.maritima*37044 | Translation | PHOSPHATIDYLINOSITOL TRANSFER PROTEIN |
|  |  | *S.maritima*33002 | Translation | uncharacterized protein LOC100191684 |
|  |  | *S.maritima*37419 | Translation | sphingoid phosphate phosphatase 1 |
|  |  | *S.maritima*32914 | Cleavage | 4-coumarate--CoA ligase-like 8 |
|  |  | *S.maritima*345325 | Translation | hypothetical protein COCSUDRAFT_40018 |
|  |  | *S.maritima*21367 | Cleavage | photosystem II subunit P-1 |
|  |  | *S.maritima*29598 | Translation | elongation factor family protein |
|  |  | *S.maritima*34412 | Cleavage | chaperone DnaJ domain-containing protein |
| 17 | sma-miR157a | *S.maritima*347871 | Cleavage | SubName: Full=SQUAMOSA promoter binding protein; |
|  |  | *S.maritima*87746 | Cleavage | hypothetical protein  POPTRDRAFT_769914 |
|  |  | *S.maritima*95321 | Cleavage | PREDICTED: uncharacterized protein LOC100777766 |
|  |  | *S.maritima*30137 | Cleavage | BRE domain-containing protein |
|  |  | *S.maritima*40686 | Cleavage | serine/threonine-protein kinase MHK |
|  |  | *S.maritima*70061 | Cleavage | Os03g0802150 |
|  |  | *S.maritima*43959 | Cleavage | Os05g0151400 |
|  |  | *S.maritima*11515 | Cleavage | CDPK-related kinase |
|  |  | *S.maritima*43687 | Cleavage | Os11g0657400 |
|  |  | *S.maritima*43687 | Cleavage | ASYNAPTIC 3 |
|  |  | *S.maritima*42755 | Cleavage | hydrolase, hydrolyzing O-glycosyl compounds precursor |
|  |  | *S.maritima*42274 | Cleavage | uncharacterized protein LOC100191883 |
|  |  | *S.maritima*31662 | Cleavage | F-box/LRR-repeat protein |
|  |  | *S.maritima*33247 | Cleavage | sulfate transporter 2 |
|  |  | *S.maritima*20302 | Cleavage | putative F-box/LRR-repeat protein |
|  |  | *S.maritima*1008713 | Translation | AGL294Wp |
|  |  | *S.maritima*40857 | Cleavage | SubName: Full=Uncharacterized protein |
|  |  | *S.maritima*15309 | Cleavage | uncharacterized protein LOC100795457 |
|  |  | *S.maritima*34447 | Cleavage | Os04g0608800 |
|  |  | *S.maritima*28203 | Cleavage | pentatricopeptide repeat-containing protein |
|  |  | *S.maritima*43631 | Cleavage | uncharacterized protein |
|  |  | *S.maritima*19554 | Cleavage | PREDICTED: uncharacterized protein LOC101244071 |
|  |  | *S.maritima*43351 | Cleavage | lipase class 3 family protein |
|  |  | *S.maritima*44043 | Translation | uncharacterized protein |
|  |  | *S.maritima*14465 | Translation | uncharacterized protein |
|  |  | *S.maritima*127226 | Cleavage | DNA mismatch repair protein Msh6-2 |
| 18 | sma-miR156b | *S.maritima*87746 | Translation | hypothetical protein POPTRDRAFT_769914 |
|  |  | *S.maritima*347871 | Cleavage | SubName: Full=SQUAMOSA promoter binding protein; |
|  |  | *S.maritima*95321 | Cleavage | PREDICTED: uncharacterized protein LOC100777766 |
|  |  | *S.maritima*43687 | Cleavage | Os11g0657400 |
|  |  | *S.maritima*43687 | Cleavage | ASYNAPTIC 3 |
|  |  | *S.maritima*43959 | Cleavage | Os05g0151400 |
|  |  | *S.maritima*34246 | Cleavage | TBP-associated factor 2 |
|  |  | *S.maritima*43631 | Cleavage | uncharacterized protein |
|  |  | *S.maritima*43351 | Cleavage | lipase class 3 family protein |
|  |  | *S.maritima*422746 | Cleavage | uncharacterized protein LOC100191883 |
|  |  | *S.maritima*20302 | Cleavage | putative F-box/LRR-repeat protein |
|  |  | *S.maritima*559117 | Cleavage | U6 snRNP-associated protein Lsm7 (predicted) |
|  |  | *S.maritima*768 | Cleavage | protein embryo defective 1895 |
|  |  | *S.maritima*386590 | Translation | Os07g0212400 |
|  |  | *S.maritima*44043 | Translation | uncharacterized protein |
|  |  | *S.maritima*39407 | Cleavage | patellin-1 |
|  |  | *S.maritima*38285 | Translation | apyrase 2 |
| 19 | sma-miR166m | *S.maritima*32700 | Cleavage | uncharacterized protein LOC100274567 |
|  |  | *S.maritima*38793 | Cleavage | transketolase TKL2 |
|  |  | *S.maritima*40112 | Cleavage | enhanced downy mildew 2 |
|  |  | *S.maritima*40112 | Cleavage | enhanced downy mildew 2 |
| 20 | sma-miR165a (Homologous miRNA, ath-miR165a) | *S.maritima*32700 | Cleavage | uncharacterized protein LOC100274567 |
|  |  | *S.maritima*38793 | Cleavage | transketolase TKL2 |
|  |  | *S.maritima*43565 | Cleavage | global transcription factor group E4 |
| 21 | sma-miR319c | *S.maritima*45328 | Translation | UV damaged DNA binding protein 1 |
|  |  | *S.maritima*37040 | Cleavage | conserved hypothetical protein |
|  |  | *S.maritima*19691 | Translation | uncharacterized protein |
|  |  | *S.maritima*28134 | Cleavage | cycloidea |
|  |  | *S.maritima*25131 | Cleavage | pentatricopeptide repeat-containing protein |
|  |  | *S.maritima*1936037 | Translation | Os10g0469000 |
| 22 | sma-miR166e (Homologous miRNA, bdi-miR166e) | *S.maritima*32700 | Cleavage | uncharacterized protein LOC100274567 |
| 23 | sma-miR166j-3p | *S.maritima*32700 | Cleavage | uncharacterized protein LOC100274567 |
|  |  | *S.maritima*38793 | Cleavage | transketolase TKL2 |
|  |  | *S.maritima*40112 | Cleavage | enhanced downy mildew 2 |
|  |  | *S.maritima*40112 | Cleavage | enhanced downy mildew 2 |
| 24 | sma-miR169g | *S.maritima*31847 | Cleavage | SubName: Full=Nuclear transcription factor Y subunit A8; |
|  |  | *S.maritima*43442 | Cleavage | uncharacterized protein LOC100789738 |
|  |  | *S.maritima*1339 | Cleavage | phosphoethanolamine N-methyltransferase 1 |
|  |  | *S.maritima*10265 | Cleavage | ADP-ribosylation factor 3 |
|  |  | *S.maritima*32301 | Cleavage | hypothetical protein SORBIDRAFT_01g013430 |
|  |  | *S.maritima*25945 | Translation | putative TIR-NBS-LRR class disease resistance protein |
|  |  | *S.maritima*29358 | Translation | Toll Interleukin1 receptor-nucleotide binding-Leu- rich repeat-type resistance protein |
|  |  | *S.maritima*14740 | Cleavage | PREDICTED: uncharacterized protein LOC101249172 |
| 25 | sma-miR159a (Homologous miRNA, pta-miR159a) | *S.maritima*37567 | Cleavage | uncharacterized protein |
|  |  | *S.maritima*1271050 | Cleavage | hypothetical protein EMIHUDRAFT_460924 |
|  |  | *S.maritima*220693 | Cleavage | peptidase C12, ubiquitin carboxyl-terminal hydrolase 2 |
|  |  | *S.maritima*32946 | Cleavage | Sti1p |
|  |  | *S.maritima*43182 | Cleavage | Os08g0152600 |
|  |  | *S.maritima*40017 | Cleavage | Os01g0667700 |
|  |  | *S.maritima*45135 | Cleavage | Os08g0224000 |
|  |  | *S.maritima*19691 | Translation | uncharacterized protein |
|  |  | *S.maritima*35210 | Cleavage | uncharacterized protein |
|  |  | *S.maritima*1502527 | Cleavage | uncharacterized protein LOC100502343 |
|  |  | *S.maritima*41595 | Translation | kelch repeat-containing protein |
|  |  | *S.maritima*471539 | Cleavage | uncharacterized protein LOC100277001 precursor |
|  |  | *S.maritima*45409 | Cleavage | ubiquitin-protein ligase 2 |
|  |  | *S.maritima*565601 | Cleavage | Os08g0125300 |
|  |  | *S.maritima*15946 | Cleavage | PREDICTED: uncharacterized protein LOC101223066 |
|  |  | *S.maritima*25402 | Cleavage | Os12g0149300 |
|  |  | *S.maritima*25432 | Cleavage | uncharacterized protein LOC100819752 |
|  |  | *S.maritima*45403 | Cleavage | zinc finger C-x8-C-x5-C-x3-H type family protein |
|  |  | *S.maritima*45411 | Cleavage | NB-ARC domain-containing disease resistance protein |
|  |  | *S.maritima*44351 | Cleavage | SH3 domain-containing protein |
|  |  | *S.maritima*14250 | Translation | hypothetical protein COCSUDRAFT_56672 |
|  |  | *S.maritima*35465 | Cleavage | Os02g0771700 |
| 26 | sma-miR157d | *S.maritima*347871 | Cleavage | SubName: Full=SQUAMOSA promoter binding protein; |
|  |  | *S.maritima*95321 | Translation | PREDICTED: uncharacterized protein LOC100777766 |
|  |  | *S.maritima*87746 | Translation | hypothetical protein POPTRDRAFT_769914 |
|  |  | *S.maritima*43959 | Cleavage | Os05g0151400 |
|  |  | *S.maritima*30137 | Cleavage | BRE domain-containing protein |
|  |  | *S.maritima*40686 | Cleavage | serine/threonine-protein kinase MHK |
|  |  | *S.maritima*422746 | Cleavage | uncharacterized protein LOC100191883 |
|  |  | *S.maritima*20302 | Translation | putative F-box/LRR-repeat protein |
|  |  | *S.maritima*37085 | Translation | FAF family protein |
|  |  | *S.maritima*44014 | Cleavage | FIP1 [V]-like protein |
|  |  | *S.maritima*8774 | Translation | protein trichome birefringence-like 11 |
|  |  | *S.maritima*11515 | Cleavage | CDPK-related kinase |
|  |  | *S.maritima*34246 | Cleavage | TBP-associated factor 2 |
|  |  | *S.maritima*44043 | Translation | uncharacterized protein |
|  |  | *S.maritima*1008713 | Cleavage | AGL294Wp |
|  |  | *S.maritima*559117 | Cleavage | U6 snRNP-associated protein Lsm7 (predicted) |
|  |  | *S.maritima*18847 | Cleavage | THO complex, subunit 5 |
| 27 | sma-miR166e (Homologous miRNA, osa-miR166e) | *S.maritima*32700 | Cleavage | uncharacterized protein LOC100274567 |
|  |  | *S.maritima*43126 | Cleavage | polynucleotide adenylyltransferase family protein |
|  |  | *S.maritima*40112 | Cleavage | enhanced downy mildew 2 |
|  |  | *S.maritima*40112 | Cleavage | enhanced downy mildew 2 |
|  |  | *S.maritima*38030 | Cleavage | probable folate-biopterin transporter 5 |
|  |  | *S.maritima*35677 | Translation | aspartyl protease family protein |
|  |  | *S.maritima*45147 | Cleavage | NAD(P)-binding Rossmann-fold superfamily protein |
|  |  | *S.maritima*35175 | Cleavage | Dbp10p |
| 28 | sma-miR165a (Homologous miRNA, aly-miR165a) | *S.maritima*32700 | Cleavage | uncharacterized protein LOC100274567 |
|  |  | *S.maritima*38793 | Cleavage | transketolase TKL2 |
|  |  | *S.maritima*43565 | Cleavage | global transcription factor group E4 |
| 29 | sma-miR166b | *S.maritima*32700 | Cleavage | uncharacterized protein LOC100274567 |
|  |  | *S.maritima*38793 | Cleavage | transketolase TKL2 |
|  |  | *S.maritima*40112 | Cleavage | enhanced downy mildew 2 |
|  |  | *S.maritima*40112 | Cleavage | enhanced downy mildew 2 |
| 30 | sma-miR166j | *S.maritima*32700 | Cleavage | uncharacterized protein LOC100274567 |
|  |  | *S.maritima*35384 | Cleavage | nuclear RNA polymerase D1B |
|  |  | *S.maritima*31694 | Cleavage | PREDICTED: pre-mRNA-processing factor 39-like |
|  |  | *S.maritima*42780 | Translation | BEL1-like homeodomain 5 |
